# *In vivo* prime editing rescues alternating hemiplegia of childhood in mice

**DOI:** 10.1016/j.cell.2025.06.038

**Published:** 2025-07-21

**Authors:** Alexander A. Sousa, Markus Terrey, Holt A. Sakai, Christine Q. Simmons, Elena Arystarkhova, Natalia S. Morsci, Laura C. Anderson, Jun Xie, Fabian Suri-Payer, Linda C. Laux, Emmanuel Roze, Sylvie Forlani, Guangping Gao, Simon Frost, Nina Frost, Kathleen J. Sweadner, Alfred L. George, Cathleen M. Lutz, David R. Liu

**Affiliations:** 1 Merkin Institute of Transformative Technologies in Healthcare, The Broad Institute of Massachusetts Institute of Technology and Harvard, Cambridge, MA 02142, USA; 2 Department of Chemistry and Chemical Biology, Harvard University, Cambridge, MA 02138, USA; 3 Howard Hughes Medical Institute, Harvard University, Cambridge, MA 02142, USA; 4 Rare Disease Translational Center, The Jackson Laboratory, Bar Harbor, ME 04609, USA; 5 Department of Pharmacology, Northwestern University Feinberg School of Medicine, Chicago, IL 60611, USA; 6 Department of Neurosurgery, Massachusetts General Hospital, Boston, MA 02129, USA; 7 Harvard Medical School, Boston, MA 02115, USA; 8 RARE Hope, Washington, DC, USA; 9 Department of Genetics & Cellular Medicine, Horae Gene Therapy Center, Li Weibo Institute for Rare Diseases Research, UMass Chan Medical School, University of Massachusetts, Worcester, MA 01605, USA; 10 Department of Microbiology, UMass Chan Medical School, University of Massachusetts, Worcester, MA 01605, USA; 11 Department of Molecular and Cellular Biology, Harvard University, Cambridge, MA 02138, USA; 12 Broad Institute of Massachusetts Institute of Technology and Harvard, Cambridge, MA 02142, USA; 13 Department of Pediatrics, Northwestern University Feinberg School of Medicine, Chicago, IL 60611, USA; 14 Ann & Robert H. Lurie Children’s Hospital of Chicago, Chicago, IL 60611, USA; 15 Sorbonne University, Inserm, CNRS, Paris Brain Institute, APHP, Institute of Neurology, 75005 Paris, France; 16 Sorbonne University, Inserm, CNRS, Paris Brain Institute, APHP, Hôpital de la Pitié Salpêtriè re, 75013 Paris, France; 17 JAX Center for Precision Genetics, The Jackson Laboratory, Bar Harbor, ME 04609, USA; 18 These authors contributed equally; 19 Lead contact

## Abstract

Alternating hemiplegia of childhood (AHC) is a neurodevelopmental disorder with no disease-modifying treatment. Mutations in *ATP1A3*, encoding an Na^+^/K^+^ ATPase subunit, cause 70% of AHC cases. Here, we present prime editing (PE) and base editing (BE) strategies to correct *ATP1A3* and *Atp1a3* mutations in human cells and in two AHC mouse models. We used PE and BE to correct five prevalent *ATP1A3* mutations with 43%–90% efficiency. AAV9-mediated *in vivo* PE corrects *Atp1a3* D801N and E815K in the CNS of two AHC mouse models, yielding up to 48% DNA correction and 73% mRNA correction in bulk brain cortex. *In vivo* PE rescued clinically relevant phenotypes, including restoration of ATPase activity; amelioration of paroxysmal spells, motor defects, and cognition deficits; and dramatic extension of animal lifespan. This work suggests a potential one-time PE treatment for AHC and establishes the ability of PE to rescue a neurological disease in animals.

## In brief

Prime editing and base editing in mouse models and human cells effectively correct mutations in a gene that causes a childhood neurodevelopmental disorder called alternating hemiplegia of childhood. *In vivo* prime editing in the brain results in significant improvements in neurological symptoms and lifespan, highlighting a potential one-time gene therapy approach for the disorder.

## Graphical Abstract

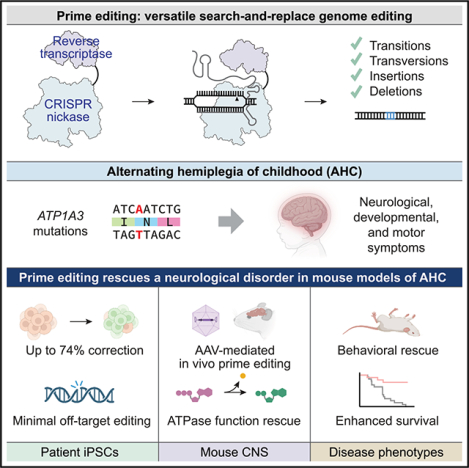

## INTRODUCTION

Alternating hemiplegia of childhood (AHC) is a neurodevelopmental disorder that presents within the first 18 months of life with recurrent paroxysmal attacks that include hemiplegia (paralysis affecting one side of the body), dystonia (painful involuntary muscle contractions), abnormal eye movements, and seizures. Patients with AHC also exhibit non-paroxysmal hypotonia (low muscle tone), developmental delay, and intellectual disability.^1^ AHC is exceptionally rare, with an estimated incidence of 1 in 1,000,000 individuals.^2^ No disease-modifying treatments for AHC exist.^1^

**Figure 1.**
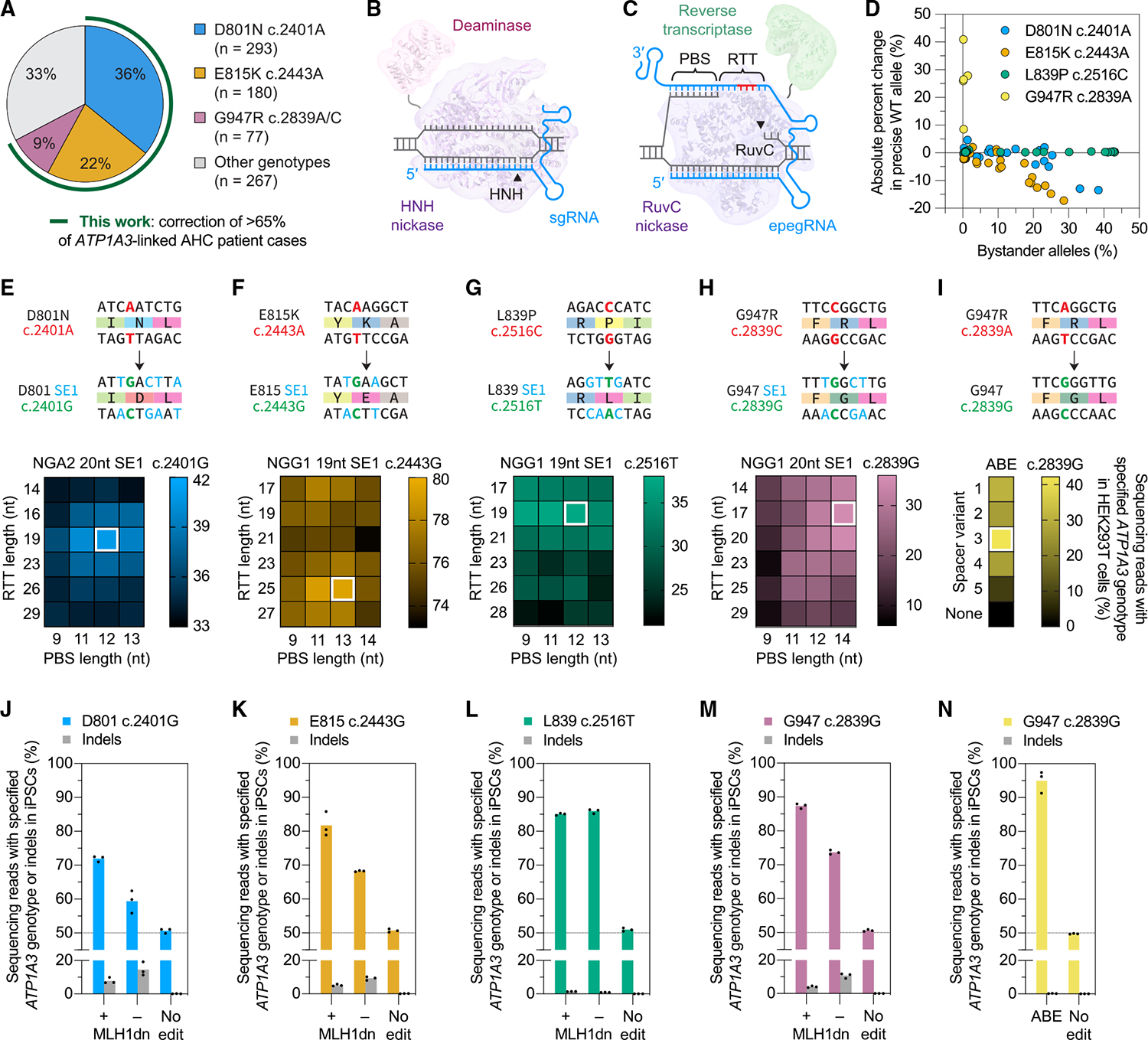
BE and PE efficiently correct AHC mutations in engineered HEK293T cells and iPSCs from patients with AHC (A) Frequency of common *ATP1A3* pathogenic variants in AHC cases.^6^ (B) Base editing by Cas9 HNH nickase fused to a ssDNA-deaminase (PDB: 6VPC) directs targeted deamination in a guide-RNA-programmed manner. (C) Prime editing by Cas9 RuvC nickase fused to a reverse transcriptase (PDB: 8WUT) directs precise editing in complex with an engineered prime editing guide RNA (epegRNA). The nicked target DNA strand hybridizes to the primer binding site (PBS), and the reverse transcription template (RTT) specifies the desired edit (red). (D) The absolute percent change in precise WT allele frequency versus cumulative bystander editing upon treatment with ABE (to correct D801N c.2401A, E815K c.2443A, or G947R c.2839A) or CBE (to correct L839P c.2516C) via transfection in HEK293T cells. Each dot represents the outcome of an sgRNA and deaminase variant paired with a PAM-compatible Cas9 domain. (E–I) Top: Correction of *ATP1A3* mutations (red) to the wild-type base (green) and co-installation of benign synonymous (silent) edits (blue). Bottom: Subset of optimization by plasmid transfection in HEK239T cells, highlighting optimal epegRNAs (white box). (J–N) Correction by RNA electroporation in patient-derived iPSCs, which start from ~50% wild-type genotype as the heterozygous baseline (dotted line). For (E)–(N), data represent the mean of *n* = 3 independent biological replicates. Dots show individual replicates. See also Figures S1, S2, S3, S4, and S5.

Approximately 70% of AHC cases are associated with pathogenic variants in *ATP1A3*, which encodes the catalytic α_3_ subunit of the neuronal Na^+^/K^+^ ATPase protein complex.^3–6^ Although more than 50 AHC-associated *ATP1A3* pathogenic variants have been reported,^7^ three—D801N, E815K, and G947R—account for more than 65% of *ATP1A3*-associated AHC cases, with approximate prevalences of 40%, 20%, and 10%, respectively^3–6,8–12^ (Figure 1A). Pathogenic *ATP1A3* variants are heterozygous, missense, and almost always *de novo*, with a notable absence of overt loss-of-function variants such as frameshifts or premature stop codons.^3–5,8–12^ Electrophysiological studies suggest that the D801N, E815K, and G947R mutants impair wild-type ATP1A3 activity.^13^ In mice, homozygosity for patient-derived pathogenic variants or complete *Atp1a3* knockout is lethal.^14–16^ Heterozygous Atp1a3^+/−^ knockout mice do not develop severe phenotypic defects in contrast to mice heterozygous for D801N, E815K, and G947R mutations.^14–23^ Although these observations support a dominant-negative disease mechanism for AHC-associated *ATP1A3* mutations, the absence of a definitive molecular mechanism^1,14,24,25^ and the challenge of selectively targeting dominant-negative protein variants hinders the development of conventional, non-genetic therapies. The current standard-of-care focuses on symptom management.^1^

Efforts to develop genetic therapies are ongoing, including a recent gene therapy study in which an *Atp1a3* D801N mouse model of AHC was treated with an AAV9-delivered *ATP1A3* transgene. Unfortunately, no improvement in clinically relevant behavioral deficits was reported,^24^ highlighting the need for more effective AHC therapeutic approaches.

Precision genome editing offers a promising therapeutic avenue to directly address the underlying cause of diseases, including those such as AHC that lack well-defined molecular mechanisms. Among these approaches, base editing (BE) and prime editing (PE) enable precise genome modifications in cells and living organisms without requiring double-strand breaks (DSBs) or donor DNA templates.^26–29^ By avoiding DSBs, BE and PE minimize undesirable outcomes such as uncontrolled insertions and deletions (indels),^26,27,29,30^ large deletions,^31,32^ chromosomal abnormalities,^31,33–36^ retrotransposon integration,^37^ and p53 upregulation.^38–40^ BE uses deaminases fused to programmable DNA-binding domains to introduce targeted point mutations such as C·G-to-T·A and A·T-to-G·C, which are installed using cytosine base editors^41^ (CBEs) and adenine base editors^42^ (ABEs), respectively (Figure 1B). PE combines a reverse transcriptase (RT), a programmable nickase DNA-binding domain, a PE guide RNA (pegRNA), and an optional nicking guide RNA (ngRNA) to install virtually any substitution, small deletion, or small insertion into the target site (Figure 1C).^43^ Since their initial development, BE and PE have rapidly improved,^26–28^ yielding current-generation base editors and prime editors capable of efficient and highly specific targeted gene correction in cells,^44,45^ animals,^29,46–73^ and human patients.^74–79^

Although BE and PE represent promising strategies for a one-time, permanent genome correction treatment for AHC, several key challenges must be addressed to validate this possibility. First, although the first PE clinical trial is underway as an *ex vivo* treatment for chronic granulomatous disease,^80^ safe and efficacious *in vivo* therapeutic PE, particularly in the central nervous system (CNS), remains underexplored. Second, given ATP1A3’s critical role in embryonic brain development,^81,82^ it was uncertain whether postnatal genome correction could effectively rescue AHC phenotypes. Finally, in order for a majority of AHC cases to be addressed, we would need to rapidly and systematically develop multiple correction strategies in parallel, testing the scalability of modern gene editing approaches. Motivated by these challenges, we pursued precision gene editing to address the root cause of AHC pathology and validate a new approach for potential treatment of this devastating disease.

Here, we report PE and BE correction of AHC mutations in human cells and in two mouse models of AHC. In engineered HEK293T cells and induced pluripotent stem cells (iPSCs) derived from patients with AHC, we developed PE and BE strategies that efficiently correct (40%–90% in treated iPSCs) five *ATP1A3* mutations that together cause more than 65% of *ATP1A3*-associated AHC cases. To investigate how gene editing can mitigate the pathology of AHC *in vivo*, we engineered PE strategies to efficiently correct the mouse *Atp1a3* D801N and E815K mutations—orthologous to the two most prevalent human pathogenic variants—in two AHC mouse models. Dual AAV-mediated delivery to the mouse CNS as a postnatal day 0 (P0) intracerebroventricular (ICV) injection at a dose below that of an FDA-approved AAV9 drug^83^ efficiently corrects the *Atp1a3* D801N and E815K mutations, rescues hippocampal Atp1a3 ATPase activity, ameliorates multiple behavioral defects, and greatly extends lifespan in *Atp1a3* D801N mice. Collectively, these findings establish efficient and precise genome editing strategies that correct the most prevalent AHC mutations, demonstrate durable multi-phenotype rescue of AHC in animals, and establish the ability of *in vivo* PE to rescue a neurological disorder. Our work suggests the feasibility of a one-time gene editing treatment that can mitigate the pathology of AHC through direct, efficient correction of a variety of AHC-causing mutations.

## RESULTS

### Bystander editing limits base editing of some ATP1A3 mutations

We sought to develop gene editing strategies to correct five *ATP1A3* mutations that collectively represent 65% of *ATP1A3*associated AHC cases: D801N c.2401A (“D801N”), E815K c.2443A (“E815K”), L839P c.2516C (“L839P”), G947R c.2839A (“G947R-A”), and G947R c.2839C (“G947R-C”). To optimize BE and PE correction approaches,^44,45^ we used PE to generate HEK293T cell lines with each pathogenic variant in endogenous *ATP1A3* (Table S1A). Four variants—D801N, E815K, L839P, and G947R-A—are transition mutations that can, in principle, be corrected with CBEs or ABEs. Although *in silico* prediction tools can predict BE bystander edits,^84^ we sought to thoroughly interrogate BE possibilities at these candidate sites through experiments in cultured cells. We targeted each transition mutation using a variety of single-guide RNAs (sgRNAs) and base editors comprising a protospacer adjacent motif (PAM)-compatible Cas9 HNH nickase and CBE or ABE deaminase (Figures 1D and S1A–S1D).

D801N, E815K, and L839P editing was efficient but resulted in substantial non-synonymous bystander editing of bases adjacent to the targeted mutations. Using engineered deaminases to vary activity and editing window size did not substantially reduce bystander editing (Figures S1A–S1C). In contrast, G947R-A ABE editing was efficient and precise (up to 40% correction and no observed bystander editing with sgRNA G947R BE-3 and ABE8e-SpCas9^85^), likely because the target adenosine is well-isolated from the nearest bystander A (Figures 1I and S1D). Given these results, we selected the G947R BE-3 and ABE8e-SpCas9 strategy to correct the G947R-A mutation and turned to PE, which does not induce bystander editing, for the other four targets.

### Developing PE strategies to correct AHC mutations

To develop PE strategies^86^ for the remaining mutations, we first determined the optimal engineered pegRNA (epegRNA)^87^ primer-binding site (PBS) and reverse transcription template (RTT) lengths across multiple protospacers (Figures S2A, S2B, S3A, S3B, S4A, S4B, S5A, and S5B) to maximize correction efficiency and minimize indels. We designed silent edit installation strategies to disrupt epegRNA PAMs and protospacers^43^ and create clusters of silent edits near the corrected mutation to increase cellular mismatch repair (MMR) evasion.^88^ We also hypothesized that 19-nt epegRNA spacers may be beneficial when (1) a 20-nt epegRNA spacer did not start with a 5′ G and (2) truncating that 20-nt epegRNA spacer to 19 nt would create a spacer with a genome-matched 5′ G. Efficient U6-driven spacer transcription requires a leading purine,^44,89^ and 5′ G spacer extensions can diminish SpCas9 nuclease activity,^90^ interfere with PE complex formation,^91^ and reduce PE efficiencies.^55^ For E815K and L839P, epegRNAs with 19-nt spacers and genome-matched 5′ Gs outperformed identical epegRNAs with 20-nt spacers extended to 21 nt by addition of a 5′ G across 77 of 79 tested designs (Figures S3A, S3B, S4A, and S4B). By thoroughly exploring these parameters, we identified epegRNAs capable of correcting all four remaining AHC mutations (Figures 1E–1H, S2B, S3B, S4B, and S5B).

Next, we evaluated ngRNAs for each mutation. For D801N, we tested ngRNAs with NGA PAMs against three variants of the epegRNA D801N NGA2 PBS12 RTT19 (Figure S2C). For E815K (Figure S3C), L839P (Figure S4C), and G947R-C (Figure S5C), we tested ngRNAs against the best-performing epegRNAs from each tested protospacer. For all mutations, we tested ngRNAs with epegRNA-ngRNA inter-nick distances of approximately 100 bp and, when possible, PE3b nicks to minimize indel byproducts.^43,44^ We identified ngRNAs that maximize PE correction of *ATP1A3* D801N, E815K, L839P, and G947R-C and minimize indels, offering up to 1.8-fold, 1.3-fold, 1.1-fold, and 1.6-fold improvements in pathogenic allele correction, respectively.

Although epegRNA and ngRNA optimization yielded E815K, L839P, and G947R-C strategies with greater than 55% correction efficiency, the best D801N approach only corrected 22% of pathogenic alleles (Figure S2C). To improve D801N editing, we tested our optimal epegRNA-ngRNA pair with PE6 variants, prioritizing RT variants PE6b–d for their dual-AAV size compatibility and their enhanced activity *in vitro* and *in vivo*.^52^ We did not use PE6e–g because their Cas-domain mutations have not yet been tested in combination with the SpCas9(VRQR)^92^ domain targeting D801N (Figure S2A). Although VRQR-PE6b–d did not substantially improve editing compared with VRQR-PEmax (Figure S2D), we selected VRQR-PE6c for its suitability in dual-AAV delivery systems, since VRQR-PEmax would need to be truncated to VRQR-PEmaxΔRNaseH,^53^ potentially introducing edit-dependent deficiencies.^52^ SSB/La prime editor fusions^93^ exceed dual-AAV9 size limits when packaged with epegRNA-ngRNA systems, precluding their use in our envisioned *in vivo* context.

To further enhance D801N correction, we evaluated the effect of adding “dead” sgRNAs (dsgRNAs), which have 5′ truncated 14- or 15-nt spacers and have been reported to improve PE by disrupting local chromatin to increase target site accessibility.^94^ We evaluated 23 dsgRNAs with epegRNA-dsgRNA distances of approximately 100 bp, targeting either strand of chromosomal DNA (specified as the epegRNA’s “PAM” or “non-PAM” strand). The best-performing dsgRNA, non-PAM − 44, resulted in 55% pathogenic allele correction, a 1.4-fold improvement over editing without a dsgRNA, demonstrating the utility of dsgRNAs for PE enhancement (Figure S2E). Collectively, these data demonstrate that systematic PE optimization enables efficient correction of the most common AHC-associated *ATP1A3* mutations in model HEK293T cells.

### PE corrects AHC mutations in iPSCs derived from patients with AHC

Motivated by this improvement in D801N correction efficiency, we next optimized E815K, L839P, and G947R-C editing approaches with PE6 variants and dsgRNAs in iPSCs derived from patients with AHC. We anticipated iPSCs would present a more challenging context for PE and a more relevant cell type for off-target analyses. Although we did not observe substantial improvements in E815K, L839P, and G947R-C correction using PE6b–d compared with PEmax, we selected PE6b based on the same rationale used in the selection of VRQR-PE6c for D801N (Figures S3D, S4D, and S5D). We tested small, focused panels of dsgRNAs with (e)pegRNA-dsgRNA distances of −60 to −30 and +60 to +30 bp (unless this range overlapped with a ngRNA), guided by the typical range of previously identified effective dsgRNAs (Figure S2E; *CFTR* F508del^86^). We identified E815K, L839P, and G947R-C dsgRNAs that yielded 1.3-fold, 2.1-fold, and 1.3-fold enhancements in pathogenic allele correction, respectively (Figures S3E, S4E, and S5E).

Under our final optimal conditions with MLH1dn supplementation,^88^ we observed pathogenic allele correction and indel rates of 43% and 8%, 63% and 5.2%, 70% and 1%, and 74% and 4% for D801N, E815K, L839P, and G947R-C, respectively, in patient-derived iPSC lines (Figures 1J–1M). Excluding MLH1dn supplementation, we observed pathogenic allele correction and indel rates of 18% and 15%, 36% and 9.3%, 71% and 1.0%, and 47% and 11% for D801N, E815K, L839P, and G947R-C, respectively, suggesting that MMR-directed reversion of DNA heteroduplex intermediates indeed reduces the editing efficiency and purity of several PE strategies (Figures 1J–1M). These observations imply that further optimization of silent edits will be required to support future *in vivo* strategies. We also observed efficient ABE correction of G947R-A in patient-derived iPSCs (90% pathogenic allele correction and 0.3% indels; Figure 1N). Together, these results from AHC-patient-derived iPSCs provide a proof-of-concept for efficient PE and ABE correction of mutations that account for a majority of known *ATP1A3*-associated AHC cases.

### Off-target editing analysis in iPSCs derived from patients with AHC

**Figure 2.**
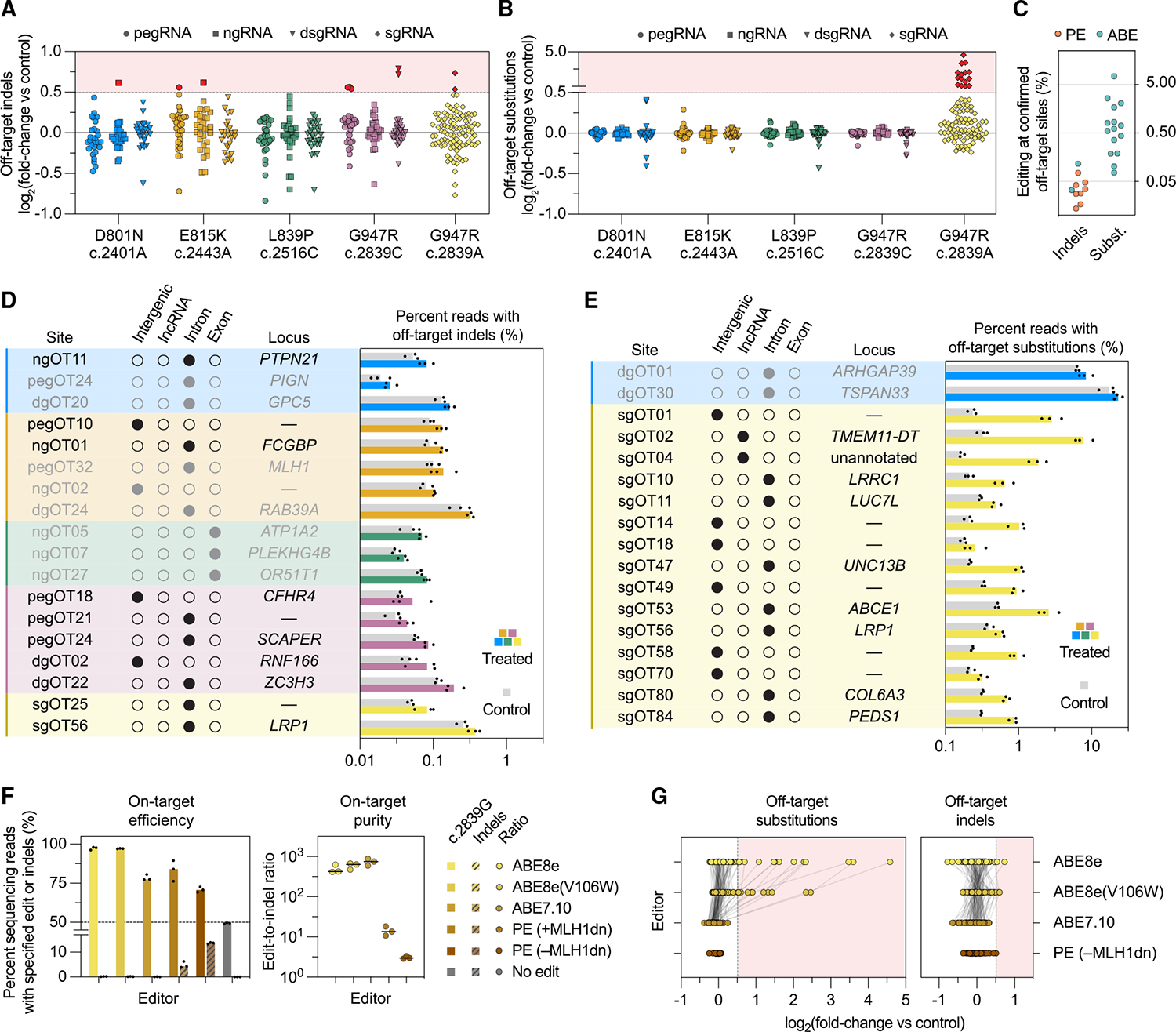
Off-target editing analysis in iPSCs derived from patients with AHC (A and B) Off-target indels (A) or substitutions (B) at 457 CIRCLE-seq nominated sites. The top 32 sites for the pegRNA, ngRNA, and dsgRNA for each PE correction strategy and the top 96 sites for the ABE correction strategy were assayed via rhAmpSeq in monoclonal iPSCs. Each dot represents editing at a nominated off-target site, shown as the ratio of editor-treated versus mock controls (*n* = 3 independent biological replicates). Sites in red were selected for further analysis. (C) Off-target editing at 24 top off-target loci. (D and E) (Left) Gene annotations of 24 top off-target sites (black text and filled circles) and 10 additional sites (gray text and filled circles) for indels (D) and substitutions (E). Off-target epegRNA, ngRNA, dsgRNA, and sgRNA sites are labeled with prefixes pegOT, ngOT, dgOT, and sgOT, respectively. (Right) Horizontal bars (colored as in A and B) represent the mean of *n* = 3 independent biological replicates for editor-treated (colored) or untreated (gray) samples. (F) Comparison of ABE and PE correction of G947R-A in RNA electroporation of iPSCs. (Left) On-target correction, starting from approximately 50% wild-type genotype (dotted line). (Right) Ratio of pathogenic allele correction:indels. (G) Substitutions (left) and indels (right) at CIRCLE-seq nominated sites, characterized as in (A) and (B). For ABE, connecting lines between the same off-target genomic loci are shown for different base editor conditions. All PE off-targets (for pegRNA, ngRNA, and dsgRNA) are shown together. Dots show individual replicates (D–F).

Next, we assessed the specificity of our optimized PE and ABE correction strategies by evaluating off-target editing in AHC patient-derived iPSCs. We performed CIRCLE-seq^95^ for all guide RNAs utilized in our optimized correction strategies (four epegRNAs, four ngRNAs, four dsgRNAs, and one ABE sgRNA). Notably, we observed guide-RNA-dependent cleavage at the on-target site for all four dsgRNAs tested (Table S2) despite their reported inactivity in cells,^94^ consistent with the high sensitivity of CIRCLE-seq in capturing low-frequency events *in vitro*. We applied pooled RNaseH-dependent amplification and sequencing (rhAmpSeq^96,97)^ at the top 96 CIRCLE-seq nominated off-target sites for each mutation, selecting the top 32 sites for each epegRNA, ngRNA, and dsgRNA and the top 96 sites for the G947R-A ABE sgRNA. For all amplifiable sites (457 of 480 nominations), we quantified indels (Figure 2A) and substitutions (Figure 2B) as the log_2_ (fold-change) upon editor treatment versus donor-matched mock controls.

Few changes at candidate loci were observed upon PE or ABE treatment relative to the donor-specific genetic background (Figure 2C). Of 24 confirmed off-targets, the majority of these validated off-targets (16 of 457 assayed sites) were consistent with guide-dependent deamination from ABE8e, a highly active deaminase.^85^ PE strategies displayed fewer genuine off-target loci (8 of 457 assayed sites) and lower levels of off-target editing, with no sites exceeding a 0.5% increase in indels or substitutions (Table S2). These observations are consistent with previous studies that have demonstrated PE’s substantially reduced off-target editing compared with alternative CRISPR-based technologies.^27,43,53,86,94,98–109^

To evaluate the potential physiological relevance of off-target editing, we analyzed the genomic contexts of confirmed off-target loci, as well as those of ten additional top off-target sites, binned by indels (Figure 2D) and substitutions (Figure 2E). Off-target activity primarily occurred in intergenic or intronic regions away from splice junctions. Two sites with ABE-induced off-target A·T-to-G·C editing were located in long non-coding RNAs (lncRNAs) of unannotated function. No off-target sites were found in UTRs, promoters, or exonic regions anticipated to affect protein expression. Although further long-term *in vivo* studies are needed to fully assess the safety of our approach and the possibility of lower frequency events, these off-target editing experiments collectively suggest that PE correction strategies for AHC-associated mutations introduce minimal off-target genomic alterations in patient-derived iPSCs under the tested conditions.

We next sought to improve our G947R-A correction strategy’s off-target profile with two approaches: (1) deaminase variants Tad8e(V106W)^85^ and Tad7.10^42^ with reduced Cas9-dependent and -independent off-target activity^45,85^ and (2) application of our G947R-C PE correction strategy—originally designed to target the c.2839C allele—to c.2839A. Both strategies were effective in maintaining on-target editing efficiency (Figure 2F, left) and edit-to-indel purity (Figure 2F, right). Off-target evaluation for each approach (Figure 2G) showed a notable reduction in the frequency of confirmed off-target sites, decreasing from 16/95 sites for ABE8e to 15/95 and 0/95 sites for ABE8e (V106W) and ABE7.10, respectively, and to 1/89 sites for PE. Our data demonstrate that using PE or alternative ABE deaminases can substantially reduce off-target editing while maintaining efficient on-target correction.

### PE efficiently corrects two AHC mouse model mutations in cultured cells

Encouraged by our efficient, precise PE and ABE correction of *ATP1A3* mutations in AHC-patient-derived iPSCs, we next examined how precision gene editing might mitigate AHC pathology *in vivo*. We targeted mouse orthologs of the two most common human *ATP1A3* variants, D801N and E815K, in recently reported mouse models of AHC, known as B6C3.*Atp1a3*^D801N/+^ and B6C3.*Atp1a3*^E815K/+^ (hereafter referred to as D801N and E815K mice, respectively).^110^ The D801N and E815K mice faithfully reproduce hallmark features of AHC and have a C3H hybrid background,^110^ which overcomes complications of general fragility and early survival defects observed in earlier AHC mouse models.^18,19,24^ Compared with B6C3.*Atp1a3*^+/+^ wild-type mice (hereafter referred to as WT mice), D801N and E815K mice display reduced lifespan and body weight, motor and cognitive defects, trigger-induced paroxysmal events, and impaired hippocampal Atp1a3 ATPase activity.^110^ Encouraged by these clinically relevant AHC phenotypes, we sought to establish how precision gene editing could treat AHC using the D801N and E815K mouse models.

Sequence divergence between human *ATP1A3* and mouse *Atp1a3* (90.8% coding sequence identity) complicates the direct transfer of optimized PE and BE strategies, as it can alter PAMs or result in mismatches between the guide RNAs and the target *Atp1a3* sequences present in the D801N and E815K mice (Figures S6A and S6B). For D801N (Figure 1J), sequence differences would change the epegRNA PAM from NGA to NGG and introduce six mismatches across the protospacer, PBS, and RTT (Figure S6A). The accompanying ngRNA and dsgRNA would have NGA and CCC PAMs, respectively, and additionally contain protospacer sequence mismatches. Given these differences, we developed a new mouse *Atp1a3* D801N PE correction strategy. First, we generated a monoclonal N2a cell line with the D801N c.2401A mutation in endogenous *Atp1a3* (Table S1B). Next, we selected the closest PE-suitable protospacer with an NGG PAM near the target mutation to evaluate a panel of corrective epegRNAs, from which we selected the *Atp1a3* D801N NGG1 20-nt SE1 PBS9 RTT23 epegRNA for further development (Figure S6C).

**Figure 3.**
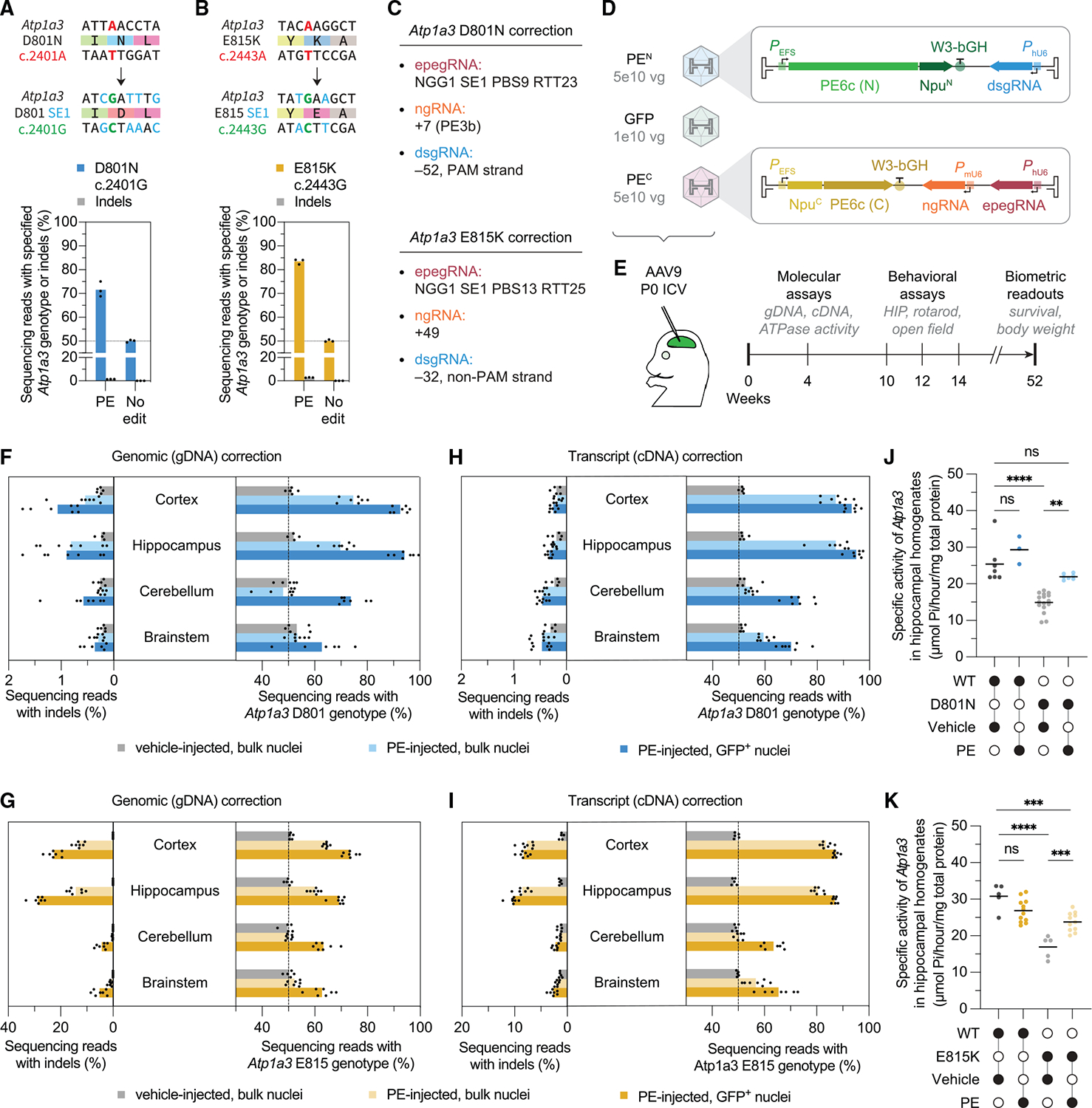
PE efficiently corrects *Atp1a3* D801N c.2401A *in vitro* and *in vivo* (A and B) Top: Correction of *Atp1a3* mutations (red) to the wild type nucleotide (green) with co-installation of benign synonymous (silent) edits (blue). Bottom: Correction with RNA electroporation in mouse fibroblasts (*n* = 3). Dots show individual replicates. (A) Correction in C57BL/6J Atp1a3 D801N c.2401A mouse primary fibroblasts. (B) Correction in E815K mouse primary fibroblasts. (C) D801N-PE-AAV9 (top) and E815K-PE-AAV9 (bottom) optimized components. (D) PE-AAV9 system encoding split-intein PE6c prime editor halves, epegRNA, ngRNA, and dsgRNA. Npu^N^ or Npu^C,^ *Nostoc punctiforme* intein N-terminal or C-terminal halves, respectively; *P*_EFS_, Promoter, elongation factor 1α short; *P*_hU6_, Promoter, human U6 polymerase III; *P*_mU6,_ Promoter, mouse U6 polymerase III; W3, minimized gamma portion of the woodchuck hepatitis virus post-transcriptional regulatory element; bGH, bovine growth hormone polyadenylation signal. (E) Mice were characterized by molecular, behavioral, and biometric readouts. (F–I) HTS quantification (*n* = 4–7) from gDNA (F and G) and cDNA (H and I) of bulk and GFP^+^ nuclei from D801N mice (F and H) or E815K mice (G and I) injected with PE-AAV9 (PE) or phosphate-buffer saline (PBS, vehicle). (J and K) Specific activity of Atp1a3 in hippocampal homogenates of PE- or vehicle-treated D801N mice (J, *n* =3–16) or E815K mice (K, *n* = 5–12). Two-way ANOVA with Tukey’s multiple comparisons. ns = not significant, *p < 0.05, **p < 0.01, ***p < 0.001, and ****p < 0.0001. For (F)–(K), dots show values from individual mice with tissue collected at P28. For (A), (B), and (F)–(I), samples start from ~50% wild-type genotype heterozygous baseline (dotted line). See also Figure S6.

Finally, we evaluated ngRNAs, PE6 variants, and dsgRNAs to enhance correction. Compared with PE without a ngRNA, we identified a +7 PE3b ngRNA that improved editing 1.4-fold and offered a very low indel rate of 0.2% (Figure S6D). Notably, this PE3b ngRNA targets a protospacer with a NAG PAM, which is weakly targetable by SpCas9^111^ but suitable at this target to enhance editing. In our evaluation of PE6 RT variants, PE6c improved D801N correction 1.2-fold over PEmax (Figure S6E). Finally, we assayed dsgRNAs and identified a −52 PAM dsgRNA that, compared with editing without a dsgRNA, enhanced pathogenic allele correction 1.3-fold to 47% with 0.3% indels (Figure S6F). As a final *in vitro* evaluation of our PE correction strategy with ngRNA, PE6, and dsgRNA enhancements, we tested our approach in C57BL/6J *Atp1a3* D801N c.2401A primary mouse fibroblasts. We observed 43% pathogenic allele correction and 1.6% indels (Figure 3A). These results demonstrate that ngRNAs with non-canonical PAMs can be used for SpCas9-based PE and that the combined use of an optimized ngRNA, PE6 variant, and dsgRNA can enhance correction of mouse D801N.

In contrast to the mouse *Atp1a3* D801N mutation, the mouse *Atp1a3* E815K site (Figure 1K) preserves the epegRNA and ngRNA PAMs from human *ATP1A3* (albeit with four spacer mismatches) but converts the dsgRNA PAM to GCC. To rapidly correct mouse E815K without additional extensive optimization, we modified our human pegRNA and ngRNA designs to accommodate sequence divergence and tested these new gRNAs with PE6 variants and dsgRNAs in E815K mouse primary fibroblasts. We observed up to 28% pathogenic allele correction with PE6c (Figure S6G) and identified a − 32 non-PAM dsgRNA that yielded a further 1.2-fold improvement, resulting in 34% pathogenic allele correction (Figure S6H). As a final *in vitro* evaluation, we tested the PE6c- and dsgRNA-enhanced approach in E815K mouse primary fibroblasts, achieving 67% pathogenic allele correction with 2.9% indels (Figure 3B). These results show that an optimized human E815K strategy can be quickly adapted to correct the mouse ortholog, supporting a streamlined cross-species PE workflow without full epegRNA-ngRNA redevelopment.

### Efficient *in vivo* prime editing rescues molecular defects in two AHC mouse models

Encouraged by efficient PE correction of D801N and E815K in mouse primary fibroblasts, we next sought to correct these mutations *in vivo*.^110^ We selected adeno-associated virus (AAV), a delivery vector used in several U.S. Food and Drug Administration (FDA)-approved drugs,^83,112–116^ and used the v1em dual-AAV system,^53^ which supports robust CNS PE by splitting prime editors across two AAVs to be reconstituted by intein *trans*-splicing upon co-transduction. Notably, our choice to use v1em (which uses a compact EF-1α short [EFS] promoter) and PE6c (515 bp shorter than PEmax from the originally reported v1em architecture^53^) provides space to accommodate epegRNA, ngRNA, and dsgRNA expression cassettes within each AAV’s ~4.8-kb packaging capacity^117,118^ (Figures 3C and 3D). To complete the design of our PE-AAV systems to correct D801N and E815K (hereafter referred to as D801N-PE-AAV9 and E815K-PE-AAV9, respectively), we selected the AAV9 capsid for its neurotropic transduction profile,^119^ its use in a FDA-approved drug^83^ to treat spinal muscular atrophy (SMA), its successful use as an intrathecally injected CNS-targeting vector in a clinical trial to treat SMA,^120^ and its ability to support robust PE and BE in the mouse CNS.^52,53,57,121^

Using ICV injection (Figures 3D and 3E), we treated P0 D801N and E815K mice with 1.0×10^11^ viral genomes (vg) of their corresponding PE-AAV9 systems (5×10^10^ vg of each AAV containing editor N-terminal or C-terminal halves) and 1.0×10^10^ vg of an EFS-eGFP-KASH (Klarsicht/ANC-1/Syne-1 homology domain) AAV9 vector to mark nuclei of AAV9-transduced cells.^53,57,121^ Because dystonia and seizures are complex neuropathological conditions involving elaborate neuronal networks, including those of the basal ganglia, thalamus, cortex, brainstem, and cerebellum,^122–126^ we isolated nuclei from the cortex, hippocampus, cerebellum, and brainstem regions to assess CNS editing at P28. We then used fluorescence-activated cell sorting (FACS) to sort bulk and GFP-positive (GFP^+^) nuclei (Figure S6I) and analyzed genotypes from gDNA and cDNA.

In D801N mouse gDNA, we observed efficient correction of pathogenic alleles in cortex (48% bulk and 85% GFP^+^ correction), hippocampus (37% bulk and 88% GFP^+^ correction), cerebellum (48% GFP^+^ correction), and brainstem (20% GFP^+^ correction) (Figure 3F). Notably, we observed very low levels (≤2%) of indels (Figure 3F). In E815K mouse gDNA, we also observed efficient correction in cortex (27% bulk and 46% GFP^+^ correction), hippocampus (20% bulk and 39% GFP^+^ correction), cerebellum (27% GFP^+^ correction), and brainstem (25% GFP^+^ correction), although we observed higher indel rates (up to 13% in bulk cortical nuclei) (Figure 3G), likely because a PE3 editing approach was used to correct E815K (Figure S3C), in contrast to the PE3b strategy used for D801N (Figure S6D). Consistent with previous AAV biodistribution studies^127–129^ and observations from our group^121,^ P0 ICV AAV9-mediated gene editing in bulk cerebellar and brainstem nuclei was inefficient (Figures 3F and 3G).

D801N and E815K cDNA analysis revealed even higher efficiencies of pathogenic allele correction in the cortex (73% and 68% bulk and 86% and 75% GFP^+^, respectively), hippocampus (73% and 62% bulk and 89% and 75% GFP^+^, respectively), cerebellum (4% and 3% bulk and 43% and 28% GFP^+,^ respectively), and brainstem (17% and 14% bulk and 38% and 31% GFP^+^, respectively) (Figures 3H and 3I). The elevated editing rates within bulk transcripts compared with bulk gDNA suggests that PE occurs more efficiently among *Atp1a3*-expressing cell types, which likely reflects the neurotropism of AAV9.^119^ Together, these results demonstrate that PE-AAV9 can efficiently correct the D801N and E815K mutations in the mouse CNS and suggests that PE is enriched in *Atp1a3*-expressing cells.

Notably, in D801N-PE-AAV9-treated D801N mice, we observed greater than 85% correction in gDNA from GFP^+^ cortical and hippocampal nuclei (Figure 3F), suggesting that nearly all fluorescent-marker-transduced cells exhibited D801N correction. Given the high editing levels, we speculate that variability in D801N correction arose mainly from differences in AAV transduction efficiency among identically treated mice. To better understand the relationship between the percentage of GFP^+^ nuclei (as a proxy for AAV transduction efficiency) and editing in bulk nuclei, we compared matched FACS and high-throughput sequencing (HTS) data from D801N mice (Figure S6J). In D801N cortical and hippocampal samples, we found a significant correlation between these variables (Pearson’s *r* = 0.82 and 0.78; *p* = 0.0236 and 0.0397, respectively), suggesting that AAV transduction efficiency was indeed a key determinant of PE correction efficiency in bulk nuclei. In E815K mice, which had lower rates of editing (Figure 3G), we did not observe such a correlation (Figure S6J), suggesting that other factors, such as editing efficiency and purity, may also influence correction variability. Together, these results suggest that optimized PE can be efficient enough—as in the D801N mice—that correction in CNS is primarily modulated by AAV9 biodistribution.

We sought to quantify the effect of our efficient *in vivo* genomic correction on Atp1a3 Na^+^K^+^-ATPase activity.^130^ Using hippocampal homogenates from the same P28 mice used for HTS analysis, we observed that vehicle-treated D801N and E815K mice had 59% and 55% Atp1a3 ATPase activity relative to vehicle-treated WT mice, respectively. However, ATPase activity of PE-AAV9-treated D801N and E815K mice was significantly (*p* = 0.0023 and 0.0002, respectively) improved to 87% and 77% of vehicle-treated WT mice ATPase activity, respectively (Figures 3J and 3K). Atp1a3 western blots of hippocampal homogenates (Figures S6K and S6L) showed no substantial change in Atp1a3 protein expression (Figures S6M and S6N). To assess whether genomic or transcriptional pathogenic allele correction in bulk hippocampal nuclei was predictive of Atp1a3 ATPase activity, we compared gDNA and cDNA HTS results to the ratio of Atp1a3 ATPase activity in PE-AAV9-treated mice and vehicle-treated WT mice (Figure S6O). Interestingly, for the highly efficient D801N correction, we found that transcriptional—but not genomic—pathogenic allele correction was significantly correlated with changes in ATPase activity (Pearson’s *r* = 0.81 and *p* = 0.0268). This difference may reflect that genomic changes capture editing in all transduced cell types, while transcriptional changes more precisely reflect editing in *Atp1a3*-expressing cells. No similar correlations were seen for E815K, though future dose-response studies may offer further insight. These results establish that PE correction of D801N and E815K restores Atp1a3 ATPase activity, a key AHC-related defect.

E815K mice show elevated serum neurofilament light chain (NFL),^110^ a neuron-specific cytoskeletal protein and biomarker of CNS damage, including neuronal injury^131–134^ and blood-brain-barrier disruption.^135^ Treatment with E815K-PE-AAV9 substantially reduced NFL levels compared with vehicle-treated controls (Figure S6P), indicating that PE treatment may mitigate CNS pathologies associated with elevated NFL.

### CNS PE improves survival and behavioral outcomes in AHC mice

**Figure 4.**
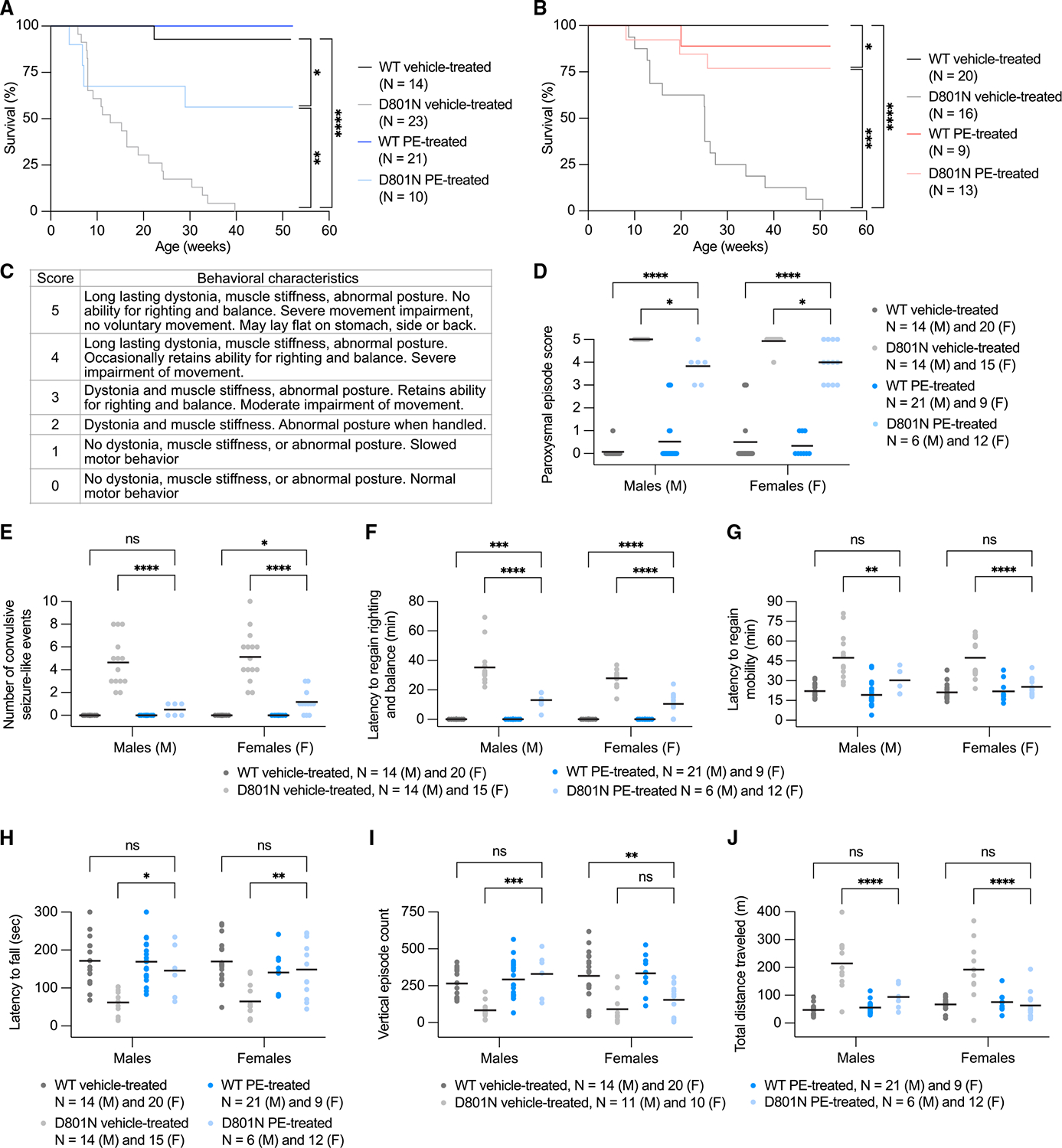
D801N-PE-AAV9 greatly improves survival and rescues multiple patient-relevant behavioral phenotypes in D801N mice (A and B) Kaplan-Meier survival curve for (A) males and (B) females for WT and D801N mice treated with PBS (“vehicle”) or D801N-PE-AAV9 (“PE”). (C) Hypothermia-induced paroxysmal episode assay (HIP assay). Hypothermia was induced by submersion in a 5 °C water bath for a body weight-normalized duration, and mice were observed for paroxysmal events and recovery. Mice were scored by blinded assessors according to standardized criteria on a scale from 0–5. (D–G) Results of HIP assay for WT and D801N mice treated with vehicle or PE for males (M, left) and females (F, right). Paroxysmal event scores (D) and number of convulsion-like events (E) were measured upon hypothermia induction. Recovery period was monitored for latency to regain righting/balance (F) and mobility (G). (H) Latency to fall off an accelerating rotarod was measured for D801N and WT mice treated with vehicle or PE for males (M, left) and females (F, right). (I and J) D801N and WT mice treated with vehicle or PE were subjected to open field testing to interrogate exploratory and/or locomotor activity in males (M, left) and females (F, right). (I) Vertical episode count. (J) Total distance traveled. For (D)–(J), each dot represents data for a single mouse. For (A), (B), and (D)–(J), the size of each cohort is displayed in the plot’s legend. For (A) and (B), Mantel-Cox test. For (D)–(J), two-way ANOVA followed by Tukey’s multiple comparisons test. ns = not significant, *p < 0.05, **p < 0.01, ***p < 0.001, and ****p < 0.0001.

**Figure 5.**
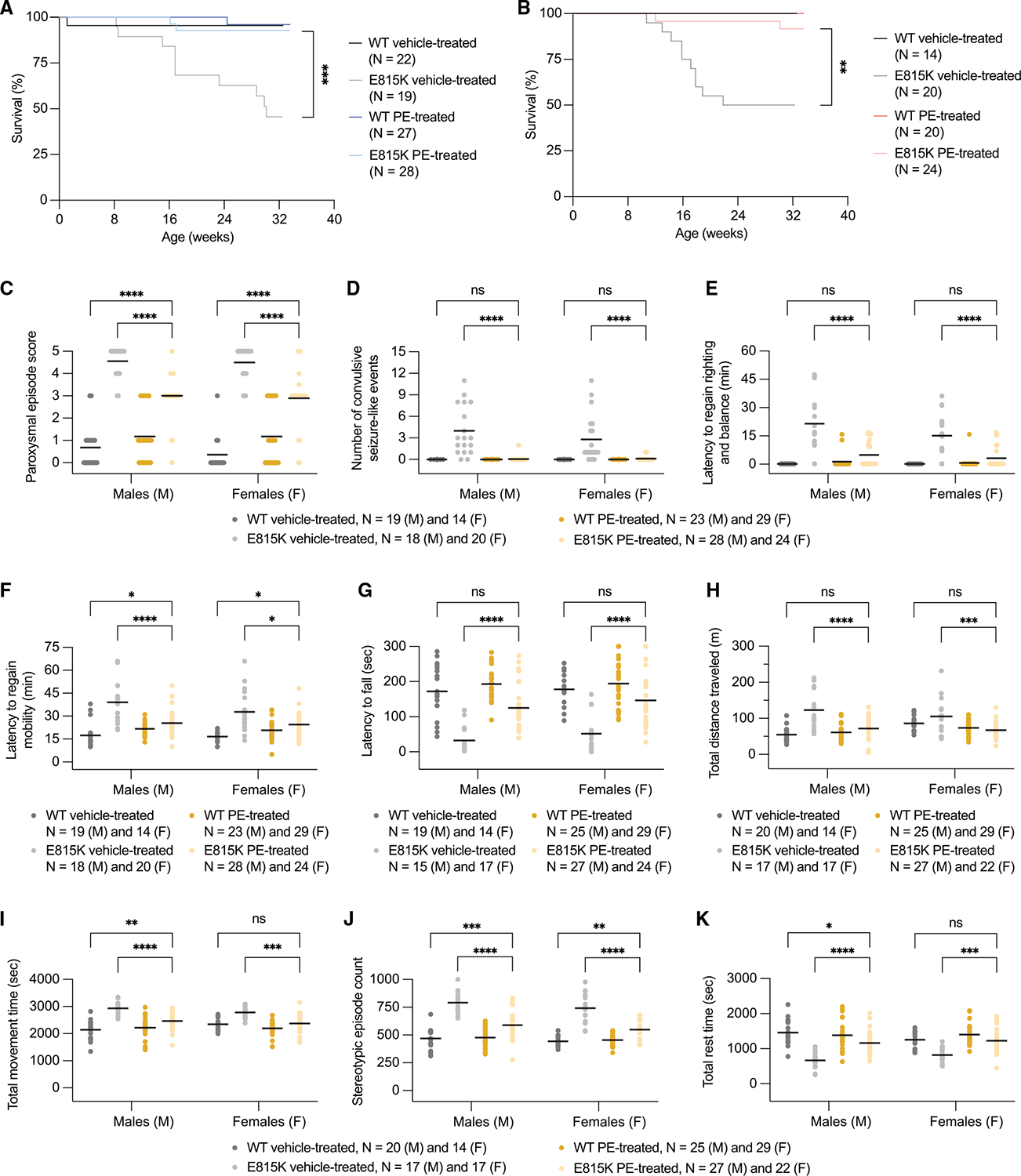
E815K-PE-AAV9 greatly improves survival and rescues multiple patient-relevant behavioral phenotypes in D801N mice (A and B) Kaplan-Meier survival curve for (A) males and (B) females for WT and E815K mice treated with phosphate-buffered saline (PBS, “vehicle”) or E815K-PEAAV9 (“PE”). (C–F) Results of HIP assay for WT and E815K mice treated with PBS (“vehicle”) or E815K-PE-AAV9 (“PE”) for males (M, left) and females (F, right). HIP assay scores (C) and number of convulsion-like events (D) were measured upon hypothermia induction. Recovery period was monitored for latency to regain righting/balance (E) and mobility (F). (G) Latency to fall off an accelerating rotarod was measured for E815K and WT mice treated with vehicle or PE for males (M, left) and females (F, right). (H–K) E815K and WT mice treated with vehicle or PE were subjected to open field testing to interrogate exploratory and/or locomotor activity in males (M, left) and females (F, right). (H) Total distance traveled. (I) Total movement time. (J) Stereotypic episode count. (K) Total rest time. For (A) and (B), Mantel-Cox test. For (C)–(K), each dot represents data for a single mouse. The size of each cohort is displayed in the plot’s legend. Two-way ANOVA followed by Tukey’s multiple comparisons test. ns = not significant, *p < 0.05, **p < 0.01, ***p < 0.001, and ****p < 0.0001.

Similar to patients with AHC, D801N and E815K mice develop spontaneous and stress-induced paroxysmal spells and show deficits in motor, behavioral, and neurophysiological activity.^110^ We observed long-term survival improvements in both cohorts of PE-treated AHC mice compared with their vehicle-treated counterparts. Among D801N-PE-AAV9-treated D801N male and female mice, 56% and 77%, respectively, survived to 52 weeks of age, whereas 0% of male and female vehicle-treated D801N mice survived beyond 35 and 51 weeks, with median survival times of 13 and 25 weeks, respectively (Figures 4A and 4B). Among E815K-PE-AAV9-treated E815K male and female mice, 93% and 92%, respectively, survived to 32 weeks of age, whereas 46% and 50% of male and female vehicle-treated E815K mice survived to 32 weeks, with median survival times of 30 and 27 weeks, respectively (Figures 5A and 5B). Although we observed an improvement in body weight of E815K-PE-AAV9-treated E815K mice compared with vehicle-treated E815K mice (Table S1C), D801N mice displayed no substantial improvement (Table S1D).

We assessed stress-induced paroxysmal episodes triggered by hypothermia, recording both the response and recovery phases (Figure 4C; Videos S1 and S2). Although the treatment did not prevent stress-induced dystonia, paroxysmal event severity in PE-AAV9-treated D801N and E815K mice was substantially reduced compared with vehicle-treated mutant mice (Figures 4D and 5C). Additionally, PE-treated mutant mice displayed a substantial reduction in convulsion-like event frequency (Figures 4E and 5D) and recovered the ability to regain balance and movement faster than vehicle-treated mutant mice (Figures 4F, 4G, 5E, and 5F).

AHC mice also exhibit motor coordination defects, which result in worse rotarod performance compared with vehicle-treated WT mice; these defects were rescued to levels of vehicle-treated WT mice levels upon PE treatment (Figures 4H and 5G). In addition, AHC mice exhibit exploratory and locomotor deficits in open-field tests, which are designed to serve as proxy assessments of cognitive function.^110^ Differences in deficits between D801N and E815K mice may reflect mutation-specific variability in patient cognitive impairments.^1,14^ D801N-PEAAV9 treatment increased vertical episode activity in male but not female D801N mice to WT-like levels and additionally decreased total distance traveled in both male and female D801N mice to WT-like levels (Figures 4I and 4J). In vehicle-treated E815K mice, we observed an increase in total distance traveled, total movement time, and stereotypic episode count as well as a reduction in rest time compared with vehicle-treated WT mice; these deficits were substantially improved following E815K-PE-AAV9 treatment (Figures 5H–5K).

## DISCUSSION

**Figure 6.**
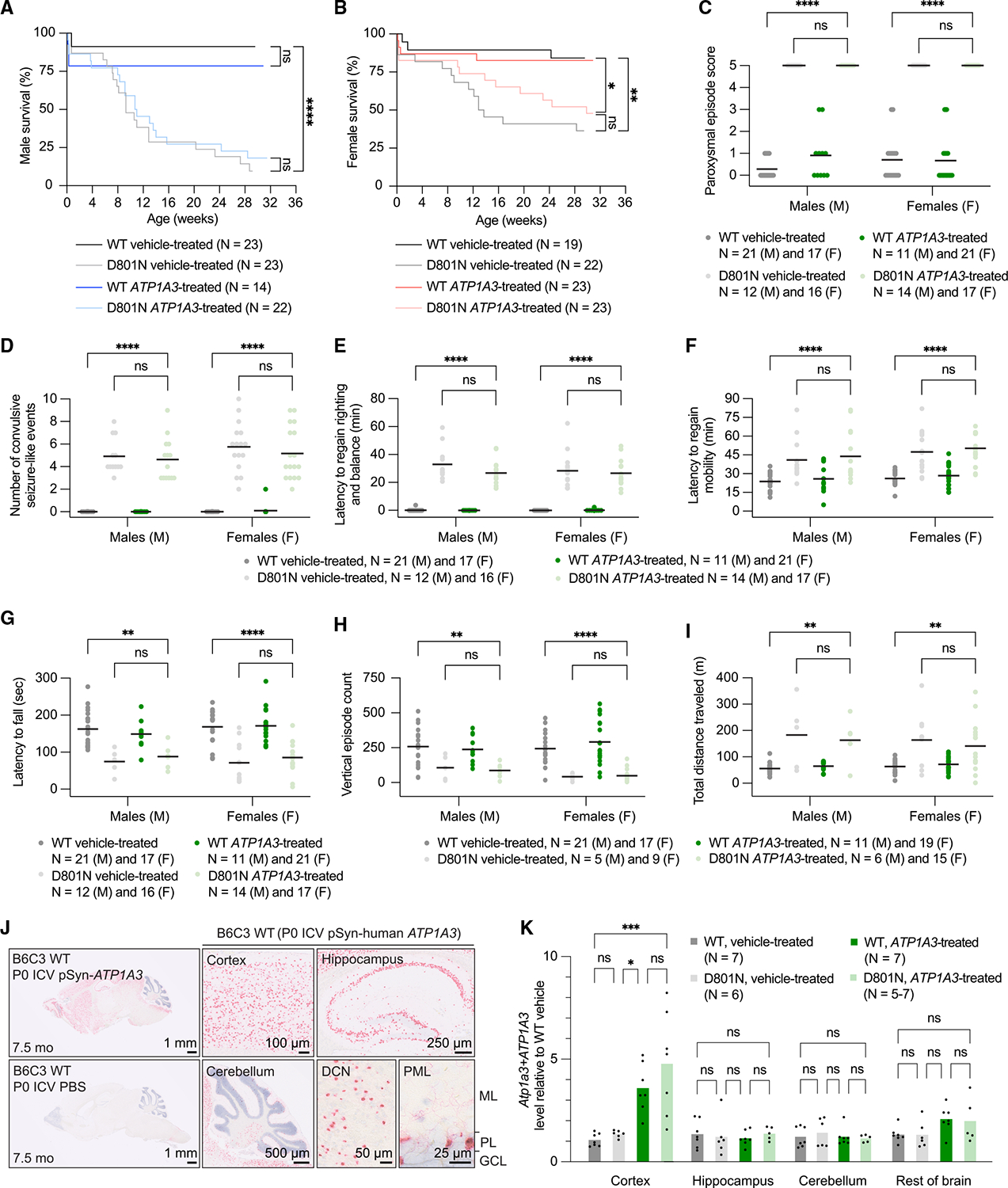
*ATP1A3* gene complementation does not rescue phenotypes in D801N mice (A–I) Survival and behavioral assays, as in Figure 4, for WT and D801N mice treated with PBS (“vehicle”) or *ATP1A3* gene therapy vector (*ATP1A3*-treated). (A and B) Kaplan-Meier survival curve. (C–F) HIP assay. (G) Rotarod assay. (H and I) Open-field assay. (J) RNA *in situ* hybridization of human *ATP1A3* mRNA (red) on parasagittal brain sections from ~30-week-old mice. WT mice were ICV injected with PBS or *ATP1A3* gene therapy vector at P0 (*n* = 2 per injection article). Sections are counterstained with hematoxylin. Higher magnifications of individual brain regions are shown with the indicated scale bars. ML, molecular cell layer; PL, Purkinje cell layer; GCL, granule cell layer; PML, paramedian lobe; and DCN, deep cerebellar nuclei. (K) *ATP1A3* gene expression analysis of different brain regions of vehicle- and *ATP1A3*-treated D801N mice at ~7 weeks of age. Quantitative RT-PCR (TaqMan) using probes that detect both human and mouse transcripts was normalized to the transcript levels of *Tbp*. Fold-change gene expression is relative to that of vehicle-treated WT mice per region of the brain. One-way ANOVA followed by Tukey’s multiple comparisons test. For (C)–(I) and (K), each dot represents a single mouse. For (A)–(I) and (K), cohort size is displayed in the plot’s legend. For (A) and (B), Mantel-Cox test. For (C)–(I) and (K), two-way ANOVA followed by Tukey’s multiple comparisons. ns = not significant, *p < 0.05, **p < 0.01, ***p < 0.001, and ****p < 0.0001.

In contrast to the benefits observed with PE, a parallel AAV9 gene therapy study that we conducted in D801N mice using a human synapsin^136,137^ promoter-driven *ATP1A3* transgene showed no improvement in disease-relevant defects (Figures 6A–6I; Table S1E). P0 ICV injection of 6.0×10^11^ vg/mouse resulted in broad and long-term *ATP1A3* expression in the CNS (Figure 6J), and we observed up to a 6-fold excess of healthy *ATP1A3* expression relative to pathogenic *Atp1a3* expression (Figure 6K). It remains possible that *ATP1A3* transgene expression was insufficient to overcome the putative dominant-negative effect of the pathogenic ATP1A3 variant and confer therapeutic benefit.

A previous gene addition therapy study in *Atp1a3* D801N *Mashl*^+/−^ mice also failed to meaningfully improve clinically relevant behavioral deficits.^24^ However, in contrast to our long-term gene therapy survival study (up to 30–32 weeks of age), that study reported a survival benefit in mice monitored only up to 10 weeks of age. Although transgene expression was not assessed in the previous study, preventing a direct comparison with our results, it is tempting to speculate that optimizing vectors to enhance *ATP1A3* expression could improve the therapeutic efficacy of future gene therapies. By contrast, precision gene editing of endogenous loci offers a promising approach for a one-time, permanent treatment of AHC. Gene correction by precision genome editing avoids the challenges of achieving sufficient transgene overexpression and complications arising from the loss of endogenous gene regulation and also partially circumvents the need to understand the molecular disease mechanism sufficiently to rationalize the safety and efficacy of gene addition.

Prior to our study, the limited understanding of AHC’s neurodevelopmental impact made it unclear whether a single postnatal intervention could result in durable, multi-phenotype rescue of AHC in adult mice. This work establishes that *in vivo* postnatal prime editing can dramatically mitigate AHC phenotypes and enhance survival, and the rescue of two models with distinct phenotypic profiles^110^ suggests a generalizable approach to correct *ATP1A3* pathogenic variants. Because we used P0 injections to achieve broad CNS transduction, additional research into later treatment windows is needed to define the temporal threshold for effective AHC gene editing and age-dependent phenotype reversibility. These investigations, along with evaluations of alternative routes of administration, dosages, and vector designs to either enhance therapeutic efficacy or further explore disease pathogenesis by identifying faulty neuronal circuits, investigating predominantly affected brain regions, or supporting histopathological analyses, will be the focus of future work.

We previously demonstrated that *in vivo* AAV-mediated BE requires 1–3 weeks to effect protein production from the edited target gene^138^ The therapeutic benefit we observed from the relatively slow-acting but permanent PE approach investigated here suggests that alternative, fast-acting but transient therapeutic modalities targeting *ATP1A3* mutations—such as an antisense oligonucleotide (ASO)^139^ or oligonucleotide-guided endogenous adenosine deaminases acting on RNA (ADARs)^140^—could potentially be used to treat AHC alone or in combination with gene editing; in a previous study, we successfully used such an approach to treat an SMA mouse model with an ASO and AAV9-delivered ABE.^57^

This study provides a proof-of-concept for *in vivo* prime editing to rescue a neurological disorder, and our data suggest that therapeutic *ATP1A3* PE could address the urgent unmet clinical need in AHC. Furthermore, our editing approaches may inform treatments for other *ATP1A3*-associated disorders.^6^ This work demonstrates that rapidly optimizing multiple correction agents in parallel may be feasible for diseases like AHC with many pathogenic variants, and developing gene editing tools capable of simultaneously modifying hundreds of bases or inserting gene-sized (>5 kb) cargos^141–144^ may also maximize patient eligibility for genome editing therapies.

### Limitations of the study

Further optimization is required for clinical PE-based AHC therapies. Our PE strategies for multiple pathogenic variants appear partially suppressed by MMR (Figures 1J–1M). Although MMR effects may differ *in vivo* with sustained editor expression, further refinement of these editing strategies is likely achievable. We streamlined initial epegRNA evaluations by testing only one MMR-evading silent edit design for most mutations; future high-throughput lentiviral screens^54,142,145–149^ could simultaneously evaluate thousands of epegRNAs to identify superior designs.

Further studies are needed to optimize the route of administration for a future AAV-based AHC PE therapy. Although some ongoing AAV9 gene therapy clinical trials use ICV injection,^150,151^ alternative strategies, such as AAV9 intrathecal delivery^120^ or intravenous administration of blood-brain-barrier-crossing AAV capsids,^152^ may also enable efficient and safe CNS transduction.

The D801N and E815K mice used lack “humanized” *ATP1A3* sequences, limiting direct translation of our *in vivo Atp1a3* gene editing findings to human AHC. However, our promising iPSC results support developing humanized mouse models, which would enable more comprehensive evaluation of *ATP1A3* editing strategies and their therapeutic potential.

## RESOURCE AVAILABILITY

### Lead contact

Please direct requests for resources and reagents to the lead contact, David R. Liu (drliu@fas.harvard.edu).

### Materials availability

Key plasmids used in this study are available from Addgene. Materials are available upon reasonable request to lead contact. Additional details are provided in the key resources table.

### Data and code availability

- Sequencing data have been deposited at the NCBI Sequence Read Archive database as BioProject ID PRJNA1211588 and are publicly available as of the date of publication. Accession numbers are listed in the key resources table.
- All original code is available in Methods S2.
- Any additional information required to reanalyze the data reported in this paper is available from the lead contact upon reasonable request.

## STAR★METHODS

### EXPERIMENTAL MODEL AND SUBJECT DETAILS

#### Mammalian cell culture conditions and isolation of primary cells

HEK293T (ATCC CRL-3216) and N2A (CCL-131) cells were purchased from American Type Culture Collection (ATCC) and cultured in Dulbecco’s modified Eagle’s medium with GlutaMAX (Thermo Fisher Scientific) supplemented with 10% fetal bovine serum (FBS; Thermo Fisher Scientific) at 37 °C with 5% CO_2_, as previously described.^44^

Primary mouse fibroblasts were isolated from 5 centimeter tail dissections, as previously described.^157^ Briefly, tail dissections were washed in 70% ethanol, air-dried, shaved, minced into sections smaller than 3 millimeters, and digested in a collagenase D and pronase solution at 37 °C for 90 minutes with horizontal shaking at 200 rpm. Following digestion, material was transferred to a culture dish by grinding tissue through a 70 μm cell strainer. The transferred cell suspension was spun down for 5 minutes at 500 × g and 4 °C twice, each time removing supernatant and resuspending pelleted material with growth media [Dulbecco’s modified Eagle’s medium with GlutaMAX (Thermo Fisher Scientific) supplemented with 20% fetal bovine serum (FBS; Thermo Fisher Scientific), 1% penicillin-streptomycin (Thermo Fisher Scientific)] supplemented with 250 ng/mL amphotericin B (MilliporeSigma). Cells were grown in amphotericin B-supplemented growth media at 37 °C with 5% CO_2_ and passaged twice before growing in growth media without amphotericin B. Following fibroblast line establishment, cells were grown in Dulbecco’s modified Eagle’s medium with GlutaMAX (Thermo Fisher Scientific) supplemented with 20% fetal bovine serum (FBS; Thermo Fisher Scientific) at 37 °C with 5% CO_2,_ as previously described for human primary fibroblasts.^44^

Patient-derived iPSC lines with heterozygous *ATP1A3* mutations were used for this study. Two lines were previously reported.^25^ Additional iPSC lines were reprogrammed from peripheral blood mononuclear cells (PBMC) from patients recruited using informed consent procedures approved by the Institutional Review Board of the Ann & Robert Lurie Children’s Hospital of Chicago or the Ethical Committee CPP Ile de France II (formerly CCPPRB de Paris-Necker). Briefly, PBMC were expanded in culture, and transduced by Sendai viral delivery of Yamanaka factors (Oct3/4, Sox2, Klf2, c-Myc). Approximately 2–3 weeks after transfection, nascent iPSC colonies were picked and replated on Matrigel-coated culture dishes using human embryonic stem cell media supplemented with ROCK inhibitor. For routine maintenance, iPSCs were cultured in B8 medium^158^ on Matrigel (Corning) coated tissue culture plates, passaged with 0.5 mM EDTA, then plated in media supplemented with 10 μM Y-27632 (Tocris). All iPSCs were incubated at 37 °C in hypoxic atmosphere with 5% CO_2_ and 5% O_2._ All iPSC lines were genotyped to verify the heterozygous mutation and screened by conventional karyotyping to exclude chromosomal aberrations.

All cell lines were verified to be free of mycoplasma and identity-authenticated by their suppliers.

#### Generation of monoclonal HEK293T and N2A cell lines

Monoclonal HEK293T and N2A cell lines were isolated by limiting dilution. Briefly, cells were transfected with the most efficient PE strategy to install a mutation of interest and subjected to limiting dilution as follows: 72 hours after transfection, cells were dissociated from adherent culture, counted, and plated across ten 96 well plates in 100 μL of media per well at a concentration of 0.5 cells per each well. Over 10 days, cells were grown and monitored for the development of monoclonal populations. Monoclonal cultures were expanded and genotyped by HTS. The isolated engineered HEK239T cell line *ATP1A3* genotypes were identified as D801N c.2401A (+/−/−), E815K c.2443A (+/+/−), L839P c.2516C (−/−/−), G947R c.2839C (−/−/−), and G947R c.2839A (−/−/−). The isolated N2A cell line *Atp1a3* genotype was identified as D801N c.2401A (−/−/−).

#### Mice

Mouse strains used for these studies have been previously described.^110^ Briefly, heterozygous B6C3.*Atp1a3*^D801N/+^ and B6C3.*Atp1a3*^E815K/+^ males were crossed to B6C3F1/J (JR #100010, The Jackson Laboratory, MGI:5654213) females every generation and the resulting progeny (B6C3.*Atp1a3*^+/+,^ B6C3.*Atp1a3*^D801N/+,^ and B6C3.*Atp1a3*^E815K/+^ mice) were subject to experimental testing. These strains are publicly available (B6C3.*Atp1a3*^D801N/+^ as MMRRC #071287 at The Jackson Laboratory, MGI:7461667; B6C3.*Atp1a3*^E815K/+^ as MMRRC #071376 at The Jackson Laboratory). Mice on study were genotyped at birth, wean age (~postnatal P28), and triple confirmed when they reached the expected study end point (e.g., necropsy for tissues collection) using the previously described genotyping primers.^110^ For simplicity, mice were given an abbreviated nomenclature throughout the manuscript with B6C3.*Atp1a3*^+/+,^ B6C3.*Atp1a3*^D801N/+,^ and B6C3.*Atp1a3*^E815K/+^ mice referred to as “WT mice”, “D801N mice”, and “E815K mice” respectively. The Jackson Laboratory Animal Care and Use Committee approved all mouse protocols.

### METHOD DETAILS

#### General molecular biology

For all general-purpose cloning experiments, nuclease-free water (Qiagen) was used, and primers were ordered from either Integrated DNA Technologies or Eton Biosciences. All plasmid construction was performed using isothermal assembly unless otherwise indicated below. Briefly, for isothermal assembly cloning, PCR was conducted using Phusion U Green Hot Start II DNA polymerase (Thermo Fisher Scientific). The resulting PCR products were purified using the QIAquick PCR Purification Kit (Qiagen), fragments were assembled using NEBuilder HiFi DNA Assembly Master Mix (New England Biolabs), and the assemblies were transformed into One Shot Mach1 cells (Thermo Fisher Scientific) before being plated on LB agar supplemented with 50 μg/mL carbenicillin.

Guide RNA expression plasmids (pegRNAs, epegRNAs, ngRNAs, dsgRNAs, and sgRNAs) were cloned using isothermal assembly and synthetic gene fragments, as previously described.^44^ Briefly, the template plasmid for any gRNA using the pU6-tevopreQ1-GG-acceptor (Addgene #174038) vector backbone was PCR-amplified using Phusion U Green Hot Start II DNA polymerase (Thermo Fisher Scientific) to generate linear dsDNA with isothermal assembly overhangs in the U6 promoter and origin of replication. PCR products were purified from a 1% agarose gel using the QIAquick Gel Extraction Kit (Qiagen). Synthetic gene fragments (purchased from Integrated DNA Technologies) with compatible overhangs were cloned into the purified PCR product using NEBuilder HiFi DNA Assembly Master Mix (New England Biolabs) and transformed into One Shot Mach1 cells (Thermo Fisher Scientific) before being plated on LB agar supplemented with 50 μg/mL carbenicillin.

All plasmids were purified using PureYield Plasmid MiniPrep Kits (Promega), Qiagen Plasmid Plus 96 MiniPrep Kits, or Qiagen Plasmid Plus Midi Kits (Qiagen). Plasmid DNA was eluted in nuclease-free water and quantified using Quant-iT dsDNA Assay Kits (Thermo Fisher Scientific) or a NanoDrop One UV-vis spectrophotometer (Thermo Fisher Scientific). Complete DNA and protein sequences of all previously unpublished base editors and prime editors used in this study are provided in Methods S1.

#### Chemically synthesized guide RNA generation

Chemically synthesized epegRNAs were ordered from Agilent and contained 2′ -O-methyl modifications at the first three nucleotides, 2′ -O-methyl modifications at the third-to-last and second-to-last nucleotides, 3′ -phosphorothioate linkages between the first three nucleotides, and 3′ -phosphonoacetate linkages between the last two nucleotides. Chemically synthesized ngRNAs were ordered from Synthego and contained 2′ -O-methyl modifications at the first three and last three nucleotides and 3′ -phosphorothioate linkages between the first three and last two nucleotides. Synthetic pegRNAs and dsgRNAs were ordered from Integrated DNA Technologies and contained 2′ -O-methyl modifications at the first three and last three nucleotides and phosphorothioate linkages between the three first and last three nucleotides.

#### Generation of *in vitro*-transcribed mRNAs

Prime editor, base editor, and MLH1dn mRNA were generated using *in vitro* transcription as previously described.^44^ Briefly, the prime editor, base editor, or MLH1dn transcripts were PCR amplified from a template plasmid using PCR primers that repair an inactive T7 (dT7) promoter and install a 119 nt poly(A) tail. PCR product was purified using QIAquick PCR purification kit (Qiagen) and then used as an *in vitro* transcription template with the HiScribe T7 high-yield RNA synthesis kit (New England Biolabs). *In vitro* transcription followed the manufacturer’s optional protocol to include CleanCap reagent AG (Trilink) and completely substitute N1-methylpseudouridine-5′ -triphosphate (Trilink) for uridine triphosphate. Reactions were incubated at 37 °C for 4 hours, DNase treated, lithium chloride precipitated, and reconstituted in nuclease-free water following HiScribe T7 high-yield RNA synthesis kit instructions.

#### HEK293T and N2A transfection

Transfections of HEK293T and N2A cells were performed as previously described.^44^ Briefly, HEK293T or N2A cells were plated at a density of 1.6–1.8 × 10^4^ cells per well of a 96 well plate (Corning) in complete culture media. Between 18–24 hours after seeding, cells were transfected at 70%–80% confluency with variable amounts of plasmid DNA and 0.5 μL of Lipofectamine 2000 (Thermo Fisher Scientific) diluted to a total volume of 10 μL with Opti-MEM (Thermo Fisher Scientific), following Lipofectamine 2000 manufacturer’s instructions. The following plasmids amounts were used in 96 well plate transfections: 200 ng of prime editor; 50 ng of pegRNA, epegRNA, or base editor sgRNA; 15 ng of ngRNA and/or dsgRNA (if included in experiment); and 100 ng of MLH1dn (if included in experiment). Transfected cells were incubated at 37 °C with 5% CO_2_ for 72 hours. See Table S3 for complete guide RNA sequence(s) for each editing condition.

#### Mouse primary fibroblasts nucleofection

Nucleofection of mouse primary fibroblasts was performed using the P2 4D-Nucleofector X Kit S (Lonza) in conjunction with a 4D-Nucleofector device (Lonza). Cells maintained in culture were dissociated with TrypLE (Thermo Fisher Scientific), and 200,000 cells per reaction were resuspended in 20 μL of complete P2 Nucleofector solution. The Nucleofector solution containing cells was supplemented with prime editing RNA reagents as follows: 1 μg of prime or base editor mRNA, 90 pmol of pegRNA or epegRNA (if applicable) and 60 pmol of ngRNA (if applicable). MLH1dn mRNA was not used in mouse primary fibroblast nucleofection experiments. In conditions with co-delivered ngRNA and dsgRNA, 30 pmol ngRNA and 30 pmol of dsgRNA was used. Negative control conditions for “No nick” or “No dsgRNA” utilized 60 pmol or 30 pmol of non-targeting sgRNA as needed. See Table S3 for complete guide RNA sequence(s) for each editing condition. The cell-RNA mixture was transferred to a Nucleofector 16-well strip and electroporated using the DS-150 program. Following nucleofection, 80 μL of pre-warmed growth media was added to each cuvette, and the resulting 100 μL cell mix was plated onto tissue culture plates. Cells were seeded onto 24-well tissue culture plates containing pre-warmed growth media and incubated at 37 °C with 5% CO_2_ for six days. On day four, cells were washed with PBS to remove dead cells and subsequently cultured in fresh growth media. Figure 3A shows correction in C57BL/6J Atp1a3 D801N c.2401A mouse primary fibroblasts, and Figure 3B shows correction in E815K mouse primary fibroblasts.

#### Electroporation of human iPSCs

Cells were cultured to 70%–80% confluence, washed with DPBS, and dissociated with Accutase (Thermo Fisher Scientific). The resulting cell suspensions were quantified and assessed for viability using a Vi-CELL automated cell counter (Beckman Coulter). Viable iPSC cells (2.5 × 10^6)^ were pelleted by centrifuging 4 min at 1000 rpm, washed with 5 ml electroporation buffer (MaxCyte, Inc.), then resuspended in 40 μl electroporation buffer (MaxCyte, Inc.).

Monoclonal iPSCs derived from patients with AHC with heterozygous *ATP1A3* mutations were electroporated to evaluate correction with chemically synthesized guide RNA component(s) and *in vitro* transcribed editor mRNA. For prime editing, cell suspensions were mixed with 5 μg PE mRNA, 5 μg MLH1dn mRNA, 450 pmol pegRNA (Integrated DNA Technologies), and 300 pmol ngRNA (Synthego). In conditions with co-delivered ngRNA and dsgRNA, 150 pmol ngRNA and 150 pmol of dsgRNA was used. For adenine base editing, cells were mixed with 3 μg ABE mRNA, and 150 pmol sgRNA (Synthego). Negative control conditions for “No nick” or “No dsgRNA” utilized 300 pmol or 150 pmol of non-targeting sgRNA as needed. Negative control (“No edit”) electroporations were performed without RNAs. Final optimized PE conditions in patient-derived cells (Figures 1J–1N) were assessed with and without MLH1dn mRNA supplementation (“MLH1dn +” and “MLH1dn −”, respectively). The mixed RNA-iPSC suspensions were transferred to an R-50×3 processing assembly and electroporated on a MaxCyte STX instrument (MaxCyte Inc.) using Optimization 8. Immediately after electroporation, cells were transferred to a tissue culture plate and recovered at 37 °C in 5% CO_2_ atmosphere for 20 minutes. After recovery, cells were plated on Matrigel coated 6-well plates in mTeSR1 media (StemCell Technologies) supplemented with 10 μM Y-27632 and incubated overnight at 37 °C in 5% CO_2_ and 5% O_2_ atmosphere. The following day, media was changed to mTeSR1 supplemented with 5 μM Y-27632. Cells were harvested 96 hours after electroporation for genomic DNA isolation.

MLH1dn IVT mRNA was also used to transiently suppress MMR to increase the editing signal of initial iPSC experiments (Figures S3D, S3E, S4D, S4E, S5D, S5E). In all PE6 variant experiments and the E815K c.2443A and G947R c.2839C dsgRNA experiments, we used shorter pegRNAs instead of epegRNAs due to more limited commercial availability of full length epegRNAs.

For iPSCs harboring the D801N c.2401A, E815K c.2443A, or G947R c.2839A mutations, experimental replicates are from three donors. For iPSCs harboring the L839P c.2516C mutation, experimental replicates are from one donor (using two different monoclonal lines: one line was edited twice, and the other line was edited once). For iPSCs harboring the G947R c.2839C mutation, experimental replicates are from two donors (using two different monoclonal lines from one donor and one monoclonal line from a second donor).

For Figure 1J, optimized PE correction of D801N c.2401A was performed with NGA2 20-nt SE1 PBS12 RTT19 epegRNA, +49 ngRNA, and −44 non-PAM dsgRNA VRQR-PE6c mRNA. For Figure 1K, optimized PE correction of E815K c.2443A was performed with NGG1 19-nt SE1 PBS13 RTT25 epegRNA, +49 ngRNA, and −42 PAM dsgRNA and PE6b mRNA. For Figure 1L, optimized PE correction of L839P c.2516C with NGG1 19-nt SE1 PBS12 RTT19 epegRNA, −1 ngRNA, and −36 PAM dsgRNA and PE6b mRNA. For Figure 1M, optimized PE correction of G947R c.2839C was performed with NGG1 20-nt SE1 PBS14 RTT17 epegRNA, −1 ngRNA, and −49 PAM dsgRNA and PE6b mRNA. For Figure 1N, optimized, ABE correction of G947R c.2839A was performed with G947R BE-3 sgRNA and ABE8e-SpCas9 mRNA. See Table S3 for complete guide RNA sequence(s) for each editing condition.

#### Genomic DNA preparation from cell culture

Genomic DNA (gDNA) was extracted from HEK293T, N2A, and primary fibroblast cell cultures using a custom lysis protocol followed by paramagnetic bead-based purification. For cell lysis, growth media was carefully removed from the culture plates, and lysis buffer (100 mM Tris-HCl pH 8.0, 200 mM NaCl, 5 mM EDTA, 0.05% SDS, 4.0 mg/mL proteinase K [New England Biolabs], and 12.5 mM DTT) was added at a volume of 100 μL per well for 96-well plates or 250 μL per well for 24-well plates. Plates containing lysis buffer were incubated at 55 °C with shaking for 20 hours. For gDNA extraction, the lysate was mixed at a 1:1 ratio with AMPure XP beads (Beckman Coulter Life Sciences), incubated for 5 minutes, and separated on a magnetic plate. The beads were washed three times with 70% ethanol with resuspension during each wash and were finally eluted in 50 μL of nuclease-free water.

For DNA isolation from iPSCs, cells were washed with DPBS, lysed in 10 mM Tris pH 7.4, 1% SDS, 10 mM EDTA, 100 mM NaCl, then incubated overnight at 55 °C with 0.2 μg/μl proteinase K (Promega). Final purification was performed by phenol-chloroform extraction followed by ethanol precipitation.

#### High-throughput sequencing and data analysis

High throughput sequencing (HTS) of genomic loci was performed as previously descrbied.^44^ Briefly, two rounds of PCR amplification were carried out using Phusion U Green Multiplex PCR Master Mix (Thermo Fisher Scientific). In the first round (PCR1), approximately 150 ng of genomic DNA was used as a template for amplification with primers containing Illumina adapter overhangs. The cycling conditions were as follows: 95 °C for 3 minutes; 27–30 cycles of 95 °C for 10 seconds, 58–61 °C (optimized experimentally) for 20 seconds, and 72 °C for 30 seconds; followed by 72 °C for 5 minutes. The second round (PCR2) used Illumina-barcoded forward and reverse primers to uniquely identify each sample. Using 1–2 μL of PCR1 as a template, PCR2 was carried out under the following conditions: 95 °C for 3 minutes; 7 cycles of 95 °C for 10 seconds, 61 °C for 20 seconds, and 72 °C for 30 seconds; followed by 72 °C for 5 minutes. PCR2 products from the same genomic locus were pooled and purified using the QIAquick Gel Extraction Kit (Qiagen) to remove primer dimers. The purified amplicons were quantified using the Qubit double-stranded DNA High Sensitivity Assay Kit (Thermo Fisher Scientific), and 4 nM libraries were sequenced on an Illumina MiSeq 300 v2 Kit with 200–300 cycles.

Sequencing reads were demultiplexed using MiSeq Reporter (Illumina) and amplicon sequences were aligned to a reference sequence using CRISPResso2^153^ as previously described.^44^ For prime editing experiments, the QWC parameter was set to the amplicon coordinates spanning 10 bp upstream of the nick position of the most 5′ epegRNA, pegRNA, ngRNA, or dsgRNA and 10 bp downstream of the nick position of the most 3′ epegRNA, pegRNA, ngRNA, or dsgRNA. If the 3′ flap generated by reverse transcription (RT) extended beyond these QWC bounds, the coordinate of the extended 3′ flap plus 10bp was used instead in place of the superseded QWC bound. For all CRISPResso2 analyses, the discard_indel_reads parameter was set as ‘TRUE’, the q parameter set as ‘30’, and, for base editing experiments, the parameter w was used in place of QWC and set to ‘20’. Additionally, in all CRISPResso2 runs, indels for a given sample were calculated as ((‘Discarded’ / ‘Reads_aligned_all_amplicons’) × 100) using the ‘CRISPRessoBatch_quantification_of_editing_frequency.txt” output file. To quantify single base genotypes, nucleotide frequency at a given position was calculated as ((frequency of specified point mutation from the ‘Nucleotide_percentage_summary.txt’ file) × (‘Reads aligned’/’Reads_aligned_ all_amplicons’)×100), where the ‘Reads aligned’ and ‘Reads_aligned_ all_amplicons’ values were collected from the ‘CRISPRessoBatch_quantification_of_editing_frequency.txt” output file. “Pathogenic allele correction” was determined for all cell lines (HEK293T models and iPSCs) accounting for their baseline genotype, listed below.

Because iPSCs derived from patients with AHC, primary fibroblasts isolated from D801N and E815K mice, and some HEK293T cell lines are heterozygous for pathogenic *ATP1A3* and *Atp1a3* mutations, all correction data are shown as “percent specified genotype” to account for the initial genotype of each cell line.

Based on HTS analysis of unedited engineered *ATP1A3* D801N c.2401A HEK293T cells, monoclonal *ATP1A3* D801N c.2401A HEK293T cells are presumed triploid for *ATP1A3* with two alleles bearing the D801N c.2401A mutation and one allele bearing the WT D801 c.2401G allele. As such, editing results from samples start from a baseline of approximately 33% “Precise WT (c.2401G)” genotype, which is displayed on plots as a dotted line.

Based on HTS analysis of unedited engineered *ATP1A3* E815K c.2443A HEK293T cells, monoclonal *ATP1A3* E815K c.2443A HEK293T cells are presumed triploid for *ATP1A3* with one allele bearing the E815K c.2443A mutation and two alleles bearing the WT E815K c.2443AG allele. As such, all samples start from a baseline of approximately 66% “Precise WT (c.2443G)” genotype, which is displayed on plots as a dotted line.

Based on HTS analysis of the unedited engineered *ATP1A3* L839P c.2516C HEK293T cells, monoclonal *ATP1A3* L839P c.2516C HEK293T cells are presumed homozygous for the *ATP1A3* L839P c.2516C allele. As such, all samples start from a baseline of approximately 0% “Precise WT (c.2516T)” genotype.

Based on HTS analysis of the unedited engineered *ATP1A3* G947R c.2839A HEK293T cells, monoclonal *ATP1A3* G947R c.2839A HEK293T cells are presumed homozygous for the *ATP1A3* G947R c.2839A allele. As such, all samples start from a baseline of approximately 0% “Precise WT (c.2839G)” genotype.

#### Analysis of base editing and quantification of alleles with bystander edits

For base editing, CRISPResso2 analysis was performed with discard_indel_reads set to ‘TRUE’, min_average_read_quality set to ‘30’, quantification_window_center set to ‘−10’, and quantification_window_size set to ‘20’. Indels for a given sample were calculated as (100% * ‘Discarded’ / ‘Reads_aligned_all_amplicons’) from the ‘CRISPResso_quantification_of_editing_frequency.txt’ output file. Product alleles were categorized using a custom python script (see Methods S2) as follows. The list of theoretical product alleles was constructed with every possible base editor-mediated deamination event within the protospacer sequence (A to G for adenine base editing, and C to T for cytosine base editing), along with combinations thereof. The percentages of reads corresponding to those product alleles were extracted from the ‘%Reads’ value in the ‘Alleles_frequency_table_around_sgRNA’ output file. Precise base editing correction to wild type (‘Precise WT’) was determined as the percentage of reads containing only the targeted deamination event, while bystander editing was calculated as the sum of reads containing any combination of undesired bystander events.

#### CIRCLE-seq *in vitro* nomination of cas9-mediated guide-dependent off-target sites

Genomic DNA was purified from HEK239T cell lines containing the edit-specific mutation of interest using the Puregene Kit (Qiagen) according to the manufacturer’s instructions. Off-target editing sites were nominated using CIRCLE-seq as described previously.^95^ Isolated genomic DNA was sheared with a Covaris LE220Rsc Focused Ultrasonicator in a 96-well plate (Covaris) to an average length of 300 bp (10 °C, 350 bp mode, 50 uL, 200 W, 25% duty factor, 50 cpb, 170 s), as confirmed by capillary electrophoresis (Agilent). The sheared DNA was subjected to enzymatic end-repair (New England Biolabs) and A-tailing (New England Biolabs), and subsequently ligated to a uracil-containing stem-loop adapter (New England Biolabs) following the manufacturer’s protocol. The adapter ligated DNA mixture was treated with Lambda Exonuclease (New England Biolabs) and *Escherichia coli* Exonuclease I (New England Biolabs) to remove unligated products. Treatment with USER enzyme (New England Biolabs) and T4 polynucleotide kinase (New England Biolabs) was performed according to the manufacturer’s directions. Circularization with T4 DNA ligase (New England Biolabs) was performed under dilute conditions to favor the intramolecular reaction (6.25 ng/uL, 16 °C, 16 h). Residual linear fragments were removed from the crude circularized DNA by treatment with Plasmid-Safe ATP-dependent DNase (Lucigen).

*In vitro* cleavage reactions were performed with 50 ng of circularized DNA (approx. 8000-fold genomic coverage), 90 nM *S. pyogenes* Cas9 protein (New England Biolabs) or Cas9-VRQR protein (purified as described previously^63^), Cas9 nuclease buffer (New England Biolabs) and 270 nM guide RNA (Integrated DNA Technologies) in a 50 μL reaction volume with incubation for 1 h at 37 °C. For base editing off-target nomination, Cas9 digestion reactions were performed with sgRNA and RNA-free negative control.

For prime editing off-target nomination, Cas9 digestion reactions were performed with pegRNA, ngRNA, dgRNA, and RNA-free negative control. Cleaved DNA products were treated with proteinase K (New England Biolabs, 5 uL of 4-fold dilution) to degrade Cas9, and the resulting mixture was A-tailed (New England Biolabs) and ligated with the NEBNext hairpin adapter (New England Biolabs) following the recommended manufacturer’s protocol. Sequencing libraries were prepared by USER enzyme treatment (New England Biolabs) and PCR barcoding with universal primers (NEBNext Multiplex oligos for Illumina) using KAPA HiFi HotStart uracil+ DNA polymerase (Roche). CIRCLE-seq libraries were sequenced on Illumina MiSeq or NextSeq2000 instruments (150 bp paired-end reads) to an average depth of >10 million paired reads per digested sample. Alignment to the human reference genome hg19 (GRCh37) assembly and off-target site analyses were performed using CHANGE-seq^156^ open-source analysis software and default recommended parameters.

#### Targeted amplicon sequencing by rhAmpSeq

Genomic sites nominated by CIRCLE-seq analysis were amplified from MaxCyte-electroporated patient iPSCs that were either treated with optimized prime editing systems (MLH1dn-free conditions; see Figure 1J–N) or mock control using the pooled rhAmpSeq system^96,97^ (Integrated DNA Technologies) for multiplexed targeted amplicon sequencing. For base editing off-target analysis, the top 96 nominated sites were selected for targeted amplicon sequencing. For prime editing off-target analysis, the top 32 nominated sites for each guide RNA (pegRNA, ngRNA, dgRNA) were selected for a total of 96 sites per prime editing strategy. The genomic coordinates used in rhAmpSeq panel design were generated from the output of the CHANGE-seq analysis software by adding a padded window of 25 nucleotides on either side of the target site (defined as the protospacer plus PAM sequence). Primer panels were generated according to the manufacturer’s instructions, with target inserts ranging from 120 to 265 bp in length.

HTS libraries were prepared following the recommended protocol. In brief, 50 ng of iPSC genomic DNA was used to template a PCR 1 reaction with rhAmpSeq primers (50 μM) under the following conditions: 95 °C for 10 min, 14 cycles of 95 °C for 15 sec, 61 °C for 8 min, followed by 99.5 °C for 15 min. Using 5.5 uL of a 1:20 dilution of the crude PCR 1 reaction as template, a PCR 2 reaction was performed using rhAmpSeq i5 and i7 unique dual indices as follows: 95 °C for 3 min, 24 cycles of 95 °C for 15 sec, 60 °C for 30 sec, and 72 °C for 30 sec, followed by 72 °C for 1 min. The resulting reactions were pooled, purified by paramagnetic bead selection (AMPureXP, Beckman Coulter), and sequenced with 300-bp single reads on an Illumina NextSeq2000 instrument. Sequencing of genomic loci that did not amplify during rhAmpSeq library preparation were subsequently assayed through arrayed amplicon sequencing using primers designed with National Center for Biotechnology Information Primer-BLAST. Primer sequences, genomic coordinates, and IDT rhAmpSeq panel IDs are listed in Table S2.

#### Quantification of off-target substitutions and indels at nominated sites

Reads were aligned to reference amplicon sequences generated from the off-target site genomic coordinates using Bowtie2 in local alignment mode with the flag “–very-fast-local” to permit alignment of untrimmed adapter sequences. Using the SAMtools software package, the resulting Sequence Alignment Map (SAM) file was converted to a sorted binary alignment map (BAM) and demultiplexed into individual fastq files, each corresponding to a single target amplicon. Adapter sequences were trimmed using CRISRPesso2 by setting trim_sequences to ‘TRUE’ and trimmomatic_options_string to ‘ILLUMINACLIP:adapters.fa:2:30:10 SLIDINGWINDOW:4:15 MINLEN:30’ with a file ‘adapter.fa’ included in the run directory containing an entry with sequence ‘AGATCG GAAGAGCACACGTCTGAACTCCAGTCA’ as previously reported for rhAmpSeq libraries,^97^ and the resulting sequencing reads were aligned to the reference genome amplicon sequence. For each off-target site, the quantification window was constructed generously to capture off-target events starting 10 bp upstream from the protospacer sequence and ending 10 bp downstream of the PAM sequence. Off-target indels were quantified by setting discard_indel_reads to ‘TRUE’ and min_average_read_quality to ‘30’ and are reported as the percentage of reads discarded versus all aligned reads: indels = (100% * ‘Discarded’ / ‘Reads_aligned_all_amplicons’) using data from the ‘CRISPResso_quantification_of_editing_frequency.txt’ output file. Off-target substitutions due to prime editing were quantified as substitutions = (100% * ‘Substitutions’ / ‘Reads_aligned_all_amplicons’). Off-target substitutions due to adenine base editing were quantified by identifying all reads containing A·T to G·C transitions within a window from position 4 to 10 of each protospacer (where the SpCas9 PAM spans positions 21–23), normalizing to all reads aligned. Custom code for quantification of off-target substitution and indels at nominated sites is included in Methods S2. Sites surpassing a 1.4-fold increase (0.5 log_2_ unit threshold) were selected for further analysis. Off-target editing in Figure 2C is calculated as the background-subtracted (unedited donor-matched control) indels or substitutions.

#### AAV production

Transfer vectors were prepared from v1em constructs from Davis et al.^53^ as previously described for PE6 editor variants.^52^ The transfer plasmid for the N-terminal half of the prime editor PE6c was cloned by restriction digestion and T4 ligation to install a human U6-driven dsgRNA cassette derived from a synthetic gene fragment (Integrated DNA Technologies) with compatible overhangs containing matched restriction sites. The transfer plasmid for the C-terminal half of the prime editor PE6c was cloned by restriction digestion and T4 ligation (New England Biolabs) to install a human U6-driven pegRNA cassette and mouse U6-driven ngRNA cassette derived from synthetic gene fragments (Integrated DNA Technologies) with compatible overhangs containing matched restriction sites. Full sequences of the assembled AAV9 transfer plasmids are provided in Methods S1.

For prime editing, recombinant AAV (rAAV) was produced via transient transfection of HEK 293 cells followed by CsCl gradient sedimentation, performed by the University of Massachusetts Medical School Viral Vector Core as described previously.^159^ Vector titers were quantified using ddPCR, and purity was evaluated through 4%–12% SDS-polyacrylamide gel electrophoresis and silver staining (Invitrogen). For gene therapy, the viral vector (pFB-PhSyn-hsaATP1A3nonopt-rBGpA) was produced by a triple-transfection method in HEK293 cells at the Vector Core at the University of North Carolina at Chapel Hill (UNC Vector Core).

#### Mouse cohorts

For each mouse therapeutic study (genome editing or gene therapy), mouse cohorts for molecular and behavioral efficacy were generated and the details are outlined below. All testing was done during daylight hours and performed using mice of either sex.

1. *Molecular biology cohort to assess in vivo editing efficiency and Atp1a3 function* (Figures 3F–3K). Mice were injected at birth (P0) and tissue samples were collected for molecular analysis at ~4 weeks of age (Postnatal day ~P28). Mice were not subjected to any behavioral testing.
2. *Behavioral cohort to assess efficiency of prime editing in vivo – body weight, HIP assay, rotarod, open field and survival monitoring cohort* (Figures 4 and 5). Mice were injected at birth (P0) and longitudinally subjected to body weights measurements, stress-induced paroxysmal episodes via hypothermia (HIP) at ~9–10 weeks of age, followed by rotarod testing at ~13–14 weeks of age, followed by open field testing at ~16–17 weeks of age, and monitored for survival defects until the study end (~52 weeks of age). Mice were not subjected to any other behavioral testing.
3. *Molecular biology cohort to assess gene therapy expression of human ATP1A3 in vivo* (Figures 6J and 6K). Mice were injected at birth (P0) and tissue samples from mice were collected for molecular analysis at ~7 weeks of age (Postnatal day ~P35). Mice were not subjected to any behavioral testing.
4. *Behavioral cohort to assess efficiency of gene therapy in vivo – body weight, HIP, rotarod, open field and survival monitoring cohort* (Figures 6A–6I). Mice were injected at birth (P0) and longitudinally subjected to body weights measurements, stress-induced paroxysmal episodes via hypothermia (HIP) at ~9–10 weeks of age, followed by rotarod testing at ~13–14 weeks of age, followed by open field testing at ~14–15 weeks of age, and monitored for survival defects until the study end (~30 weeks of age). Mice were not subjected to any other behavioral testing. Survival monitoring was not extended past 30 weeks of age since no positive indications of survival benefits were observed.

#### Intracerebroventricular injection

For neonatal ICV injection (targeting of brain ventricles located below the cortex), heterozygous B6C3.Atp1a3^D801N/+^ or B6C3.Atp1a3^E815K/+^ males were crossed to B6C3F1/J (JR #100010, The Jackson Laboratory, MGI:5654213) females (timed matings). B6C3F1/J females were singly housed 10–12 days post mating set up. The week of birth, females were checked daily for the birth of the litter. On the day of birth (postnatal day P0), the litter of mice was injected, and operators were blinded to genotype and the treatment (test article or vehicle). For gene editing studies, mice were bilaterally injected with PE-AAV9 (total dose: 1.1×10^11^ vg/mouse, 2.5μl per hemisphere). For gene therapy studies, mice were bilaterally injected with AAV9 ATP1A3 (pFB-PhSyn-hsaAT-P1A3nonopt-rBGpA, total dose: 6.0×10^11^ vg/mouse, 3μl per hemisphere). The equivalent volume of sterile PBS (ThermoFisher, #10010023) was bilaterally injected in pups to produce independent vehicle control group for each therapeutic study. Post-injection, pups were monitored daily for survival and the survival frequency was reduced to 2–3 times per week after 10–12 days post-injection.

#### Serum collection and neurofilament light chain analysis

Whole blood was collected via retro-orbital collection (RO) after applying a topical anesthetic (Proparacaine) on the eye. The collected blood was transferred into the serum collection tube (Microtainer Tube with serum separator), the tube was inverted a few times, kept at room temperature for 20–30 min, stored on wet-ice and then, centrifuged for 10 min at 14,000 × g (rcf) at 4 °C. The serum (located above the gel separator matrix) was collected and stored at −80 °C until further analysis. Serum samples were analyzed via Simoa HD-X analyzer (Simoa NF-Light v2 Advantage Kit #104073 by Quanterix) using 32x sample dilution with 2 technical replicates per sample to measure levels of neurofilament light chain (NFL).

#### Survival

In addition to the routine welfare check, animals were monitored (2–3 times per week) from birth (P0) until the defined study end. Because D801N mice undergo spontaneous deaths, mice were found dead without any prior indication of welfare and/or health concerns. In addition to spontaneous death, mice were monitored for their overall welfare and health. Mice that developed any welfare concerns, as part of regular animal husbandry (e.g., dermatitis, bite wounds), required study termination (humane euthanasia) prior to the anticipated study end date to comply with the institutional welfare regulations. One PE-AAV9-injected D801N mouse of the genome editing cohort required study termination at ~5–6 weeks of age due to bite wounds. One vehicle injected D801N mouse of the gene therapy cohort required study termination at ~9–10 weeks of age due to dermatitis. The study end of these two mice were not recorded/included as “death” (survival curve of D801N mice).

#### Body weight measurement

Individual body weights were measured using a countertop scale. A plastic tub was placed on the scale and tared. Mice were placed into the container and their weight was recorded in grams. The tub was wiped clean with ethanol between each cage of mice.

#### Necropsy and tissue collection

##### Molecular biology cohorts

At birth injected mice were euthanized by CO_2_ asphyxiation (~4 weeks of age for prime editing and ~7 weeks of age for gene therapy studies), brain tissues were harvested, dissected into cortex, hippocampus, cerebellum, brain stem and the remaining brain (‘rest of the brain’), frozen on dry-ice, and stored in −80° C freezer until use.

##### Assessment of biodistribution and expression of human ATP1A3 in aged B3C6 WT mice

Injected mice were euthanized by CO_2_ asphyxiation (~30 weeks of age), brain tissues harvested, fixed (24–48 hours) with 10% Neutral buffered formalin (NBF) for BaseScope and embedded in paraffin.

#### Rotarod performance test

Mice were habituated to the testing room one hour prior to testing with the rotarod on (Ugo Basile rotarod for Mouse). The apparatus was cleaned before and in between each mouse using 70% ethanol. Mice were tested for four consecutive trials of accelerating rotarod starting at 5 RPM and ramping up to 40 RPM over 300 seconds. Latency to fall for each of the four trials was recorded with a 45 second rest period between each trial. Day one is considered training day, followed by the same protocol on the subsequent day which is considered testing day. The latency from the testing day trials 2, 3, and 4 were averaged and reported as latency to fall for each mouse.

#### Open field test

Open field arenas used in this study were squares (40 × 40 × 40 cm) and made from clear Plexiglass. LED lights indirectly illuminated the arenas at ranges of 100–500 ± 20 lux. The chambers were vented and attenuated of sound. Mice were habituated to the testing room for 60 minutes. One mouse was placed in the center of each arena, and vertical and horizontal activity were recorded by beam breaks across two levels in infrared beams using the Fusion software (Omnitech Electronics). Mice were recorded for 60 minutes, and the arenas were cleaned with 70% ethanol between subjects within a single testing day. B6C3 D801N mice show defects in horizontal and vertical activity and E815K mice show defects in total distance traveled, total movement time, and stereotypic episode count, and rest time,^110^ and the readouts are listed below.

##### Total distance traveled

The total distance that the subject has traveled.

##### Vertical episode count

This is incremented by 1 each time the animal rears. The animal must go below the level of the vertical sensor for at least 1 second before the next rearing can be registered.

##### Total movement time

The length of time that the subject spent in activity. Activity is defined as a period in which ambulation and/or stereotypy occurred.

##### Total rest time

The length of time that the subject spent at rest. A resting period is defined as a period of inactivity greater than or equal to 1 second.

##### Stereotypic episode count

The number of beam breaks due to stereotypic activity. If the animal breaks the same beam (or set of beams) repeatedly then the monitor considers that the animal is exhibiting stereotypy. This typically happens during grooming or head bobbing.

#### Hypothermia-induced paroxysmal episode assay (HIP assay)

Hypothermia induced paroxysmal episodes were induced and scored as previously described.^110^ Briefly, a clear plastic box was filled with 5° C ± 0.2 °C cold water (monitored via thermometer). The body weight of each mouse was measured to determine the ‘duration in water’ required to accomplish a comparable body temperature reduction across mice. While restrained, the mouse was transferred into the water and then released in the water (start ‘duration in water’). Subsequently, the mouse was removed from the water bath, the rectal body temperature was immediately measured (‘0 minutes’), the mouse was placed in the ‘recovery station’ for the observation of paroxysmal episodes (‘dystonia-like’ and ‘convulsion-like’ episodes), the rectal body temperature was measured at 15-, 30- and 60-minutes post water exposure, and the entire ‘recovery period’ was video-recorded for each mouse (see Videos S1 and S2 as examples). The scoring of paroxysmal episodes (‘dystonia-like’ episodes) was performed using the previously developed scoring system.^110^ The video recordings were used to identify (i) the number of ‘convulsion-like’ episodes (reminiscent of tonic-clonic-like seizures; an episode needed to have a clear stop of at least 1 second before considering the start of a new episode), (ii) the time for mice to regain balance (time needed until paroxysmal episodes would no longer disable mice to righting and remain of their feet), and the time for mice to regain voluntary motor/mobility control (mice at this point have fully recovered and would show normal behavior e.g., moving, walking, grooming).

#### Nuclear isolation, sorting, nuclei acid extraction, and cDNA synthesis

Nuclei were isolated using following a modified version of the nuclei extraction protocol provided with Miltenyi Biotec Nuclei Extraction Buffer (Miltenyi Biotec, #130-128-024) and using a gentleMACS Octo Dissociator with Heaters (Miltenyi Biotec). All nuclei preparation steps were performed at 4 °C. Briefly, dissected brain tissue was resuspended in 2 mL complete Nuclei Extraction Buffer (Nuclei Extraction buffer supplemented with RNase Inhibitor, Murine (NEB) at a final concentration of 0.2 U/μL) and added to Miltenyi Biotec gentleMACS C tubes. The C tubes were transferred to gentleMACS Dissociator and the manufacturer default program “4C_nuclei_1” was run. Once the program was complete, homogenate was passed through a 100 μm MACS SmartStrainer into a 15mL falcon tube and 2 mL of additional complete Nuclei Extraction buffer was added to the strained homogenate’s volume. The 4 mL of strained homogenate was centrifuged at 500×g at 4 °C for 5 minutes and supernatant was removed upon completion. Pelleted material was resuspended in 4 mL ice-cold nuclei suspension buffer (PBS with 100 μg/μL albumin (NEB), 3.33 μM Vybrant DyeCycle Ruby Stain (ThermoFisher), and 0.2 U/μL RNase Inhibitor, Murine (NEB)) and centrifuged again at 500×g at 4 °C for 5 minutes. Supernatant was removed upon completion, pelleted material was resuspended in 1 mL ice-cold nuclei suspension buffer, passed through 35-μm cell strainer, followed by flow sorting using the Sony MA900 Cell Sorter (Sony Biotechnology) at the Broad Institute flow cytometry core. See Figure S6I for a representative fluorescence-activated cell sorting gating strategy. Gating was performed to identify nuclei based on forward and side scatter area, followed by selection of singlets using DyeCycle Ruby intensity. Sorting was then carried out based on GFP fluorescence, generating two populations: a “Bulk Nuclei” group, which included all nuclei regardless of GFP expression, and a “GFP-positive Nuclei” group, which consisted exclusively of GFP-positive nuclei. Nuclei were sorted into RLT Plus buffer from AllPrep DNA/RNA Mini (Qiagen) and DNA and RNA was purified according to the manufacturer’s protocol. Using kit-supplied random hexamers, cDNA was generated from RNA using SuperScript IV First-Strand Synthesis System with ezDNase enzyme (ThermoFisher) according to the manufacturer’s protocol. 10 μL of total cDNA synthesis output was used to template HTS PCR1 step.

#### Atp1a3 western blots

Crude homogenates of mouse hippocampal samples were made in 315 mM sucrose, 20 mM Tris, 1 mM EDTA, pH 7.4, containing Roche complete mini protease inhibitor cocktail (1 tablet/25 ml buffer). Protein concentrations were determined with a Lowry assay. Gel electrophoresis and western blots were performed using Invitrogen NuPage 4%–12% MES gels and transferred onto nitrocellulose membranes. Atp1a3 was detected with F1 antibody (1:300) (Santa Cruz Biotechnology, sc-374050) and anti-mouse IgG kappa chain BP-HRP binding protein (1:2,000) (Santa Cruz Biotechnology, sc-516102). Loading controls were with anti-GAPDH (1:2,000) (Cell Signaling Technology, #3683). Signals were developed with the WesternBright ECL reagent (Advansta) and measured with a GE Healthcare LAS 4000 imaging system and ImageQuant software.

#### Assay of Na,K-ATPase activity

The activity assay measured the hydrolysis of ATP in the test tube after pre-treatment of the samples with SDS at a final detergent: protein ratio of 0.58. The Na,K-ATPase is resistant to denaturing effects of SDS at this ratio. BSA was used to buffer the SDS in order to control the exposure to detergent.^130^ In brief, brain sample homogenates were preincubated with SDS for 10 min at room temperature in a solution containing 1.0 mg/ml BSA, then diluted with three volumes of a solution containing 0.3 mg/ml BSA and no additional detergent and kept on ice until assay. The ATPase reaction mixture contained 140 mM NaCl, 20 mM KCl, 4 mM MgCl_2,_ 3 mM tris ATP (Sigma), 30 mM histidine, pH 7.2. Samples containing 13.5 μg protein were added to 400 μl aliquots of reaction mixture and incubated at 37 °C for 15 min, followed by quenching with acid-molybdate and color development with Fiske-Subbarow reducing solution. Developed color was read at 700 nm with a spectrophotometer. Specific activity was calculated in μmoles of inorganic phosphate released by 1 mg of total protein per hour. Because mouse Atp1a1 Na,K-ATPase has a low affinity for ouabain, Na,K-ATPase activity due to Atp1a3 plus Atp1a2 containing isozymes was defined as total activity inhibited by 10 μM ouabain. Activity due to Atp1a1 was defined as the activity remaining in 10 μM ouabain that was inhibited by 3 mM ouabain. The amount of Atp1a2 Na,K-ATPase was negligible in hippocampal samples.

#### BaseScope (in situ hybridization) to assess biodistribution and expression of human *ATP1A3*

NBF-fixed and paraffin embedded brains were sectioned, sections were deparaffinized and rehydrated for RNA in situ hybridization. In situ hybridization of BaseScope LS Probe-BA-Hs-ATP1A3-No-XMm-2zz-st-C1 (ACD, #1320908-C1) was performed with the BaseScope LS Reagent Kit (ACD, # 323600) using the manufacturer’s protocol on the automated Leica-Bond staining system (Leica Biosystems). Sections were counterstained with hematoxylin and histological slides were digitalized at 40x resolution using a digital slide scanner (Hamamatsu NanoZoomer).

#### Reverse transcription and quantitative PCR (TaqMan) analysis (Gene therapy studies)

The RNA was isolated from mouse tissue using RNeasy Mini Kit, DNase-treated following the manufacturer’s protocol (Qiagen) and was used for cDNA synthesis using High-Capacity cDNA Reverse Transcription Kit and oligo dT primer (ThermoFisher) as per the manufacturer’s protocol. Quantitative RT-PCR (TaqMan) reactions were performed using the TaqMan Fast Advance MasterMix (ThermoFisher) and the QuantStudio Flex 7 qPCR detection system (ThermoFisher). For the validation of TaqMan probes (species specificity or species cross-reaction), TaqMan probes were tested on mouse brain cDNA derived from wild type mice (brain tissue from JR #664, C57BL/6J, The Jackson Laboratory) and human cDNA derived from a mixture of human tissues including the CNS (Takara #636693). TaqMan probes were considered human specific when detection was only observed in human tissue derived cDNA but failed to result in amplification when using mouse brain cDNA. In contrast, TaqMan probes designed to detect human and mouse *Atp1a3* had to work on both human and mouse derived cDNA samples. For quantitative expression analysis, TaqMan probes (see table below) were multiplexed with probes for the gene of interest and input control (*TATA*-*binding protein, Tbp*). The transcript expression levels of the gene of interest (FAM) were normalized to those of *Tbp* (HEX) using the 2-ΔΔCT method^160^ and expressed as the fold change ± standard error of the mean (SEM) relative to vehicle (PBS) injected wild type (B6C3 WT) mice per brain region of interest. Quantifications were performed using mice of either sex.

TaqMan probes:

**Table:** 

Gene	Species cross reaction	Primer	Fluorophore	Vendor	Sequence 5′–3′
*Atp1a3/ATP1A3*	Mouse and Human	Forward	FAM	IDT	TACGGGCAGATTGGGATGAT
		Probe			CTCGGTGGTTTCTTCTCCTACTTTGT
		Reverse			CCGGGCAAGAAGCCATTT
*Tbp*	Mouse	N/A	HEX	IDT	Product #Mm.PT.39a.22214839

Note: underlined nucleotides show polymorphisms between human and mouse Atp1a3.

### QUANTIFICATION AND STATISTICAL ANALYSIS

The number of independent biological replicates and technical replicates for each experiment are described in the figure legends or the Method details section. For behavioral (e.g., P0 ICV injections, body weight recordings, HIP, rotarod, open field, survival monitoring) and molecular (e.g., TaqMan) analysis of animal experiments, operators were blinded to genotype and treatment (vehicle or test article) during testing, data collection and data analysis.

## Supplementary Material

Table S1

Table S3

Data S1

Data S2

Table S2

Methods S2

Methods S1

Video 1

Video 2

1

SUPPLEMENTAL INFORMATION

Supplemental information can be found online at https://doi.org/10.1016/j.cell.2025.06.038.

## Figures and Tables

**Table:** KEY RESOURCES TABLE

REAGENT or RESOURCE	SOURCE	IDENTIFIER
Antibodies		
ATP1A3 antibody	Santa Cruz Biotechnology	Cat#374050; RRID:AB_10918613
GAPDH-HRP antibody	Cell Signaling Technology	Cat#3683; RRID:AB_1642205
Bacterial and virus strains		
One Shot Mach1 T1 Phage-Resistant Chemically Competent *E. coli*	Thermo Fisher Scientific	Cat#C862003
Chemicals, peptides, and recombinant proteins		
BsaI-HFv2	New England BioLabs	Cat#R3733S
T4 DNA Ligase	New England BioLabs	Cat#M0202S
NEBuilder HiFi DNA assembly master mix	New England BioLabs	Cat#E2621S
Lambda Exonuclease	New England BioLabs	Cat#M0262L
*Escherichia coli* Exonuclease I	New England BioLabs	Cat#M0293L
USER enzyme	New England BioLabs	Cat#M5505L
T4 polynucleotide kinase	New England BioLabs	Cat#M0201L
T4 DNA ligase	New England BioLabs	Cat#M0202L
Cas9 Nuclease, *S. pyogenes*	New England BioLabs	Cat#M0386M
Proteinase K, Molecular Biology Grade	New England BioLabs	Cat#P8107S
Plasmid-Safe ATP-Dependent DNase	Lucigen	Cat#E3101K
Agencourt AMPureXP	Beckman Coulter	Cat#A63881
Dimethyl sulfoxide	Sigma-Aldrich	Cat#D8418–50ML
Carbenicillin	Gold Biotechnology	Cat#C-103
Lipofectamine 2000	Thermo Fisher Scientific	Cat#11668019
TrypLE	Thermo Fisher Scientific	Cat#12605010
Proteinase K, recombinant, PCR grade	Thermo Fisher Scientific	Cat#11668019
SDS (10% wt/vol)	Thermo Fisher Scientific	Cat#15553027
AMPure XP	Beckman Coulter	Cat#B23318
CleanCap Reagent AG	TriLink BioTechnologies	Cat#N-7113
N1 -Methylpseudouridine-50 -Triphosphate	TriLink BioTechnologies	Cat#N-1081
LiCl Precipitation Solution (7.5 M)	Thermo Fisher Scientific	Cat#AM9480
DMEM, high glucose, GlutaMAX supplement	Thermo Fisher Scientific	Cat#10566016
Fetal bovine serum	Thermo Fisher Scientific	Cat#16000044
Penicillin-Streptomycin	Thermo Fisher Scientific	Cat#15070063
Opti-MEM reduced serum medium	Thermo Fisher Scientific	Cat#31985070
BSA	NEB	Cat#B9000S
Vybrant DyeCycle Ruby	Thermo Fisher	Cat#V10309
Critical commercial assays		
Phusion U Multiplex PCR Master Mix	Thermo Fisher Scientific	Cat#F562L
Q5 High-Fidelity 2 × Master Mix	New England BioLabs	Cat#M0492L
Phusion Green Hot Start II High-Fidelity DNA Polymerase	Thermo Fisher Scientific	Cat#F537L
QIAquick PCR Purification Kit	QIAGEN	Cat#28104
QIAquick Gel Extraction Kit	QIAGEN	Cat#28704
QIAGEN Plasmid Plus Midi Kit	QIAGEN	Cat#12943
QIAprep Spin Miniprep Kit	QIAGEN	Cat#27106
Puregene Cell Kit (6.7 × 10^9^)	QIAGEN	Cat#158046
Qiagen Plasmid Plus 96 Miniprep Kit	QIAGEN	Cat#16181
Illustra TempliPhi 100 amplification kit	Cytiva	Cat#25640010
96 AFA-TUBE TPX Plate	Covaris	Cat#520291
NEBNext End Repair Module	New England BioLabs	Cat#E6050L
NEBNext dA-Tailing Module	New England BioLabs	Cat#E6053L
NEBNext Quick Ligation Module	New England BioLabs	Cat#E6056L
NEBNext Multiplex Oligos for Illumina (Dual Index Primer Set 1)	New England BioLabs	Cat#E7600S
NEB T7 HiScribe Kit	New England BioLabs	Cat#E2040S
AAVpro Titration Kit version 2	Clontech/Takara	Cat#6233
MiSeq Reagent Kit v2 (300-cycles)	Illumina	Cat#MS-102–2002
MiSeq Reagent Micro Kit v2 (300-cycles)	Illumina	Cat#MS-103–1002
NextSeq 1000/2000 P1 Reagents (300 cycles)	Illumina	Cat#20050264
Agilent Tapestation D1000 ScreenTape	Agilent	Cat#5067–5582
Agilent Tapestation D1000 Reagents	Agilent	Cat#5067–5583
KAPA HiFi HotStart Uracil+Ready Mix Kit	Roche	Cat#KK2802
rhAmpSeq CRISPR Library Kit 100rxn	Integrated DNA Technologies	Cat#10007318
Deposited data		
Amplicon sequencing data	This paper	BioProject: PRJNA1211588
Experimental models: Cell lines		
Human (female): HEK293T	ATCC	Cat#CRL-3216
Mouse (male): N2a	ATCC	Cat#CCL-131
Experimental models: Organisms/strains		
B6C3F1/J mice	The Jackson Laboratory	JR #100010
B6C3.*Atp1a3*^D801N/+^ mice	The Jackson Laboratory	MMRRC #071287
B6C3.*Atp1a3*^E815K/+^ mice	The Jackson Laboratory	MMRRC #071376
Oligonucleotides		
See Tables S2 and S3	N/A	N/A
Recombinant DNA		
pCMV-PE2	Addgene	#132775
pCMV-PEmax	Addgene	#174820
pT7-PEmax	Addgene	#178113
pEF1a-MLH1dn	Addgene	#174824
pU6-tevopreq1-GG-acceptor	Addgene	#174038
pU6-pegRNA-GG-acceptor	Addgene	#132777
pCMV-PE6b	Addgene	#207852
pCMV-PE6c	Addgene	#207853
pCMV-PE6d	Addgene	#207854
Software and algorithms		
CRISPResso2	Clement et al.^153^	https://github.com/pinellolab/CRISPResso2
Prism	GraphPad	https://www.graphpad.com/
Geneious Prime	Dotmatics	https://www.geneious.com/prime/
Python 3	Python	https://www.python.org/downloads/
Bowtie2	Langmead and Salzberg^154^	https://github.com/BenLangmead/bowtie2
SAMtools	Danecek et al.^155^	https://github.com/samtools/samtools
CHANGEseq analysis software	Lazzarotto et al.^156^	https://github.com/tsailabSJ/changeseq

## Highlights

- Prime editing and base editing efficiently correct *ATP1A3* mutations in AHC patient cells
- AAV9-delivered prime editing restores *Atp1a3* sequence and ATPase function in mice
- *In vivo* prime editing also strongly rescues behavioral phenotypes and extends lifespan
- Prime editing can serve as a one-time treatment to rescue a neurological disorder
