# Supplementary material for: In vivo prime editing rescues alternating hemiplegia of childhood in mice
Source: Cell. Author manuscript; Available in PMC 2025 Dec 14. (PMC12702498; doi:10.1016/j.cell.2025.06.038)
Supplement: Data S1 [file NIHMS2127386-supplement-Data_S1.pdf]

Analysis of allele-specific outcomes, related to Figures 1J-M.

In each scheme below, we analyzed all possible combinations of silent edits upon treatment with or without MLH1dn to determine partial incorporation or correction of silent edits (blue), pathogenic SNP (red), or wild-type SNP (green). All experiments are performed in patient-derived iPSCs in three independent biological replicates, with data re-analyzed from Figures 1J-M.

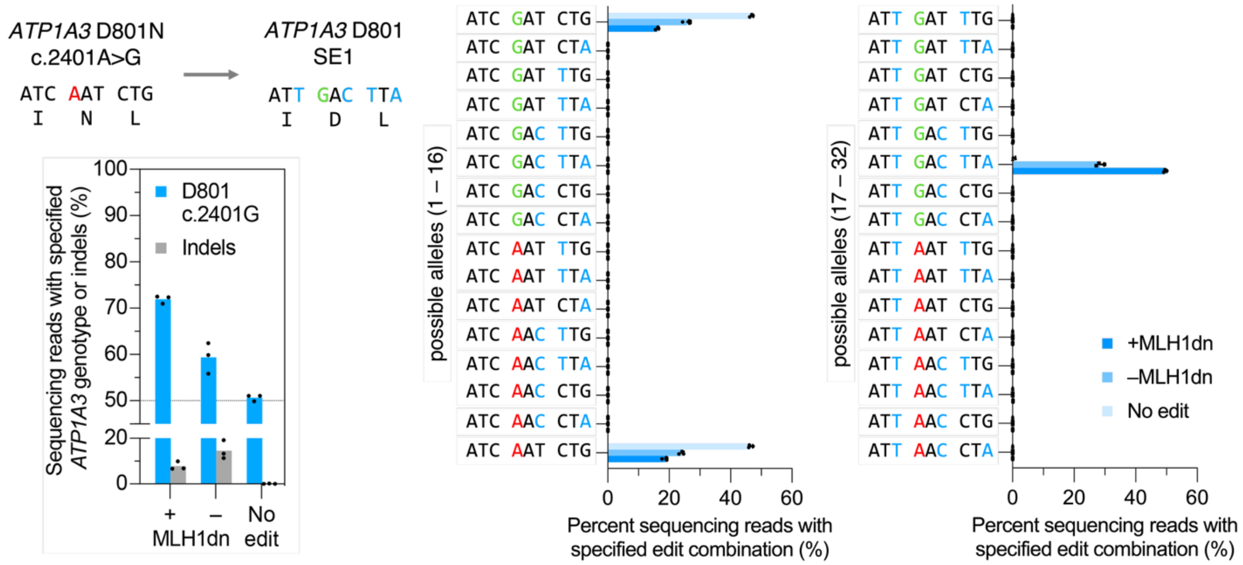

Partial edit allele-specific analysis of correction of D801N, related to Figures 1E and 1J.

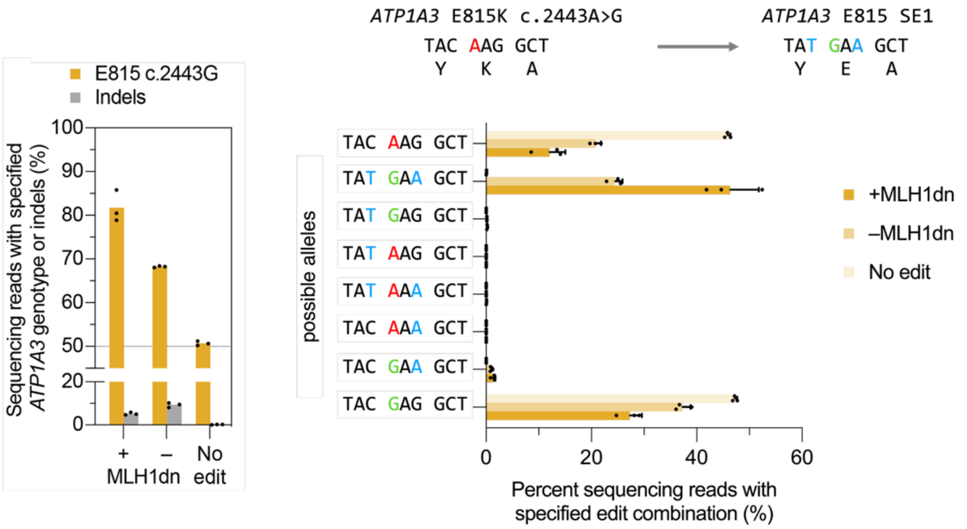

Partial edit allele-specific analysis of correction of E815K, related to Figures 1F and 1K.

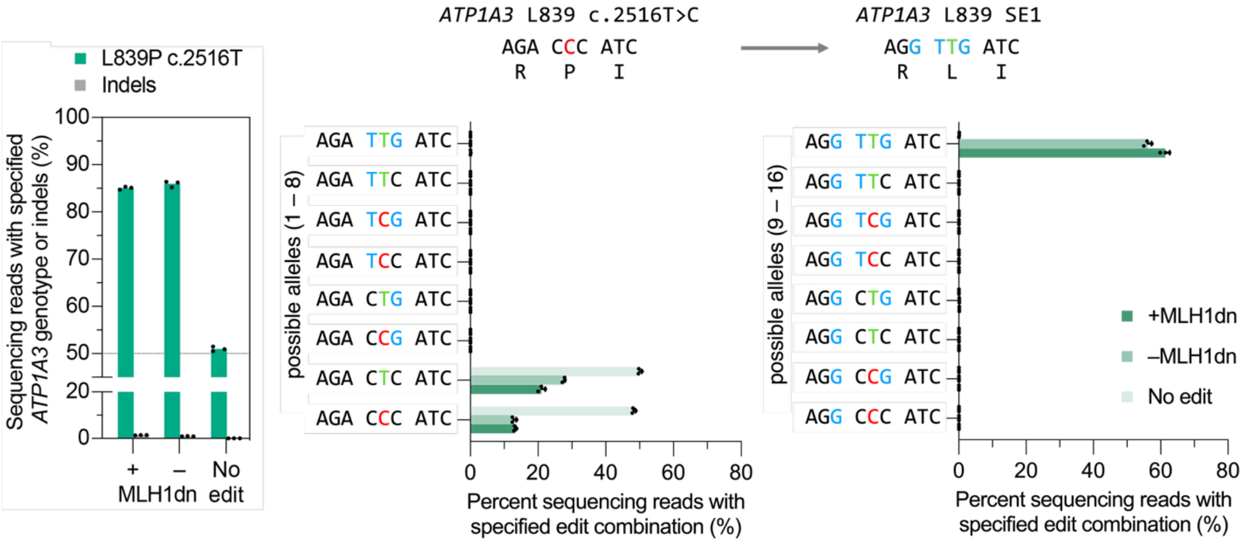

Partial edit allele-specific analysis of correction of L839P, related to Figures 1G and 1L.

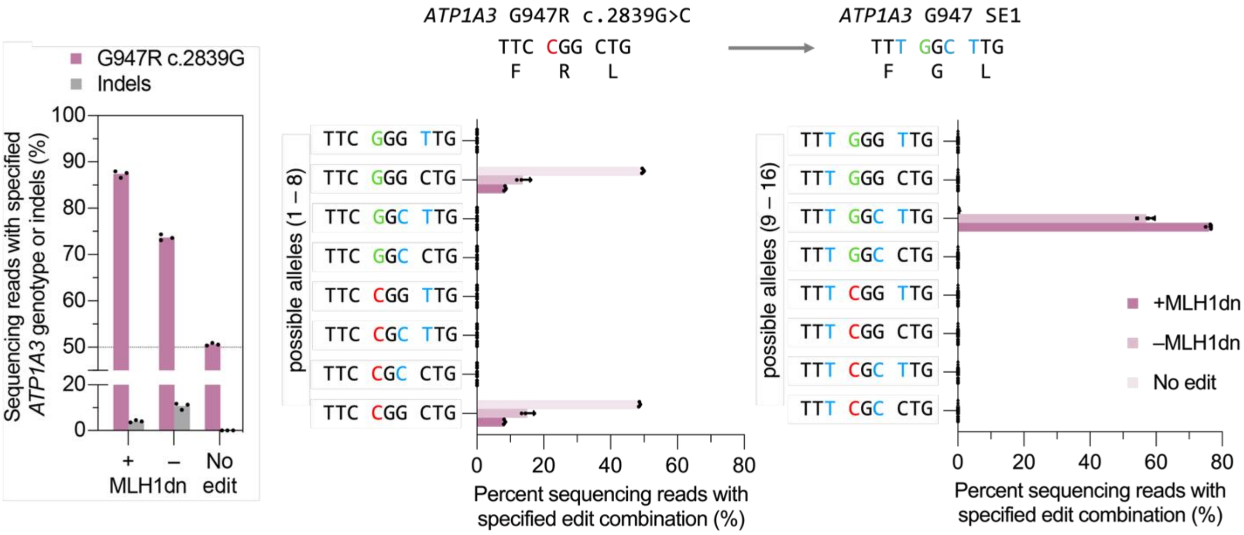

Partial edit allele-specific analysis of correction of G947R c.2893C, related to Figures 1H and 1M.
