# Supplementary material for: In vivo prime editing rescues alternating hemiplegia of childhood in mice
Source: Cell. Author manuscript; Available in PMC 2025 Dec 14. (PMC12702498; doi:10.1016/j.cell.2025.06.038)

Uncropped Atp1a3 western blot from vehicle and PE-treated WT and D801N mice, related to Supplementary Figures 6K and 6M.

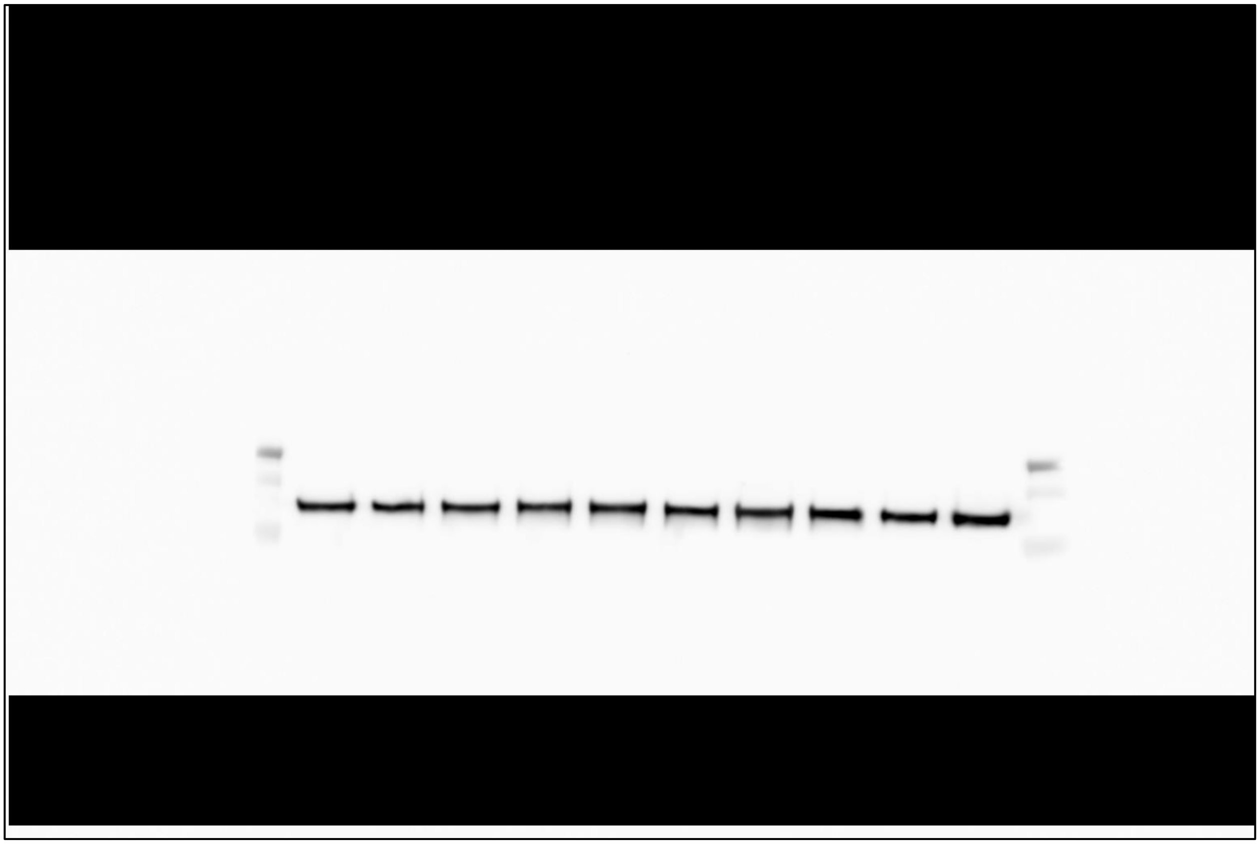

Uncropped GAPDH western blot from vehicle and PE-treated WT and D801N mice, related to Figure Supplementary Figures 6K and 6M.

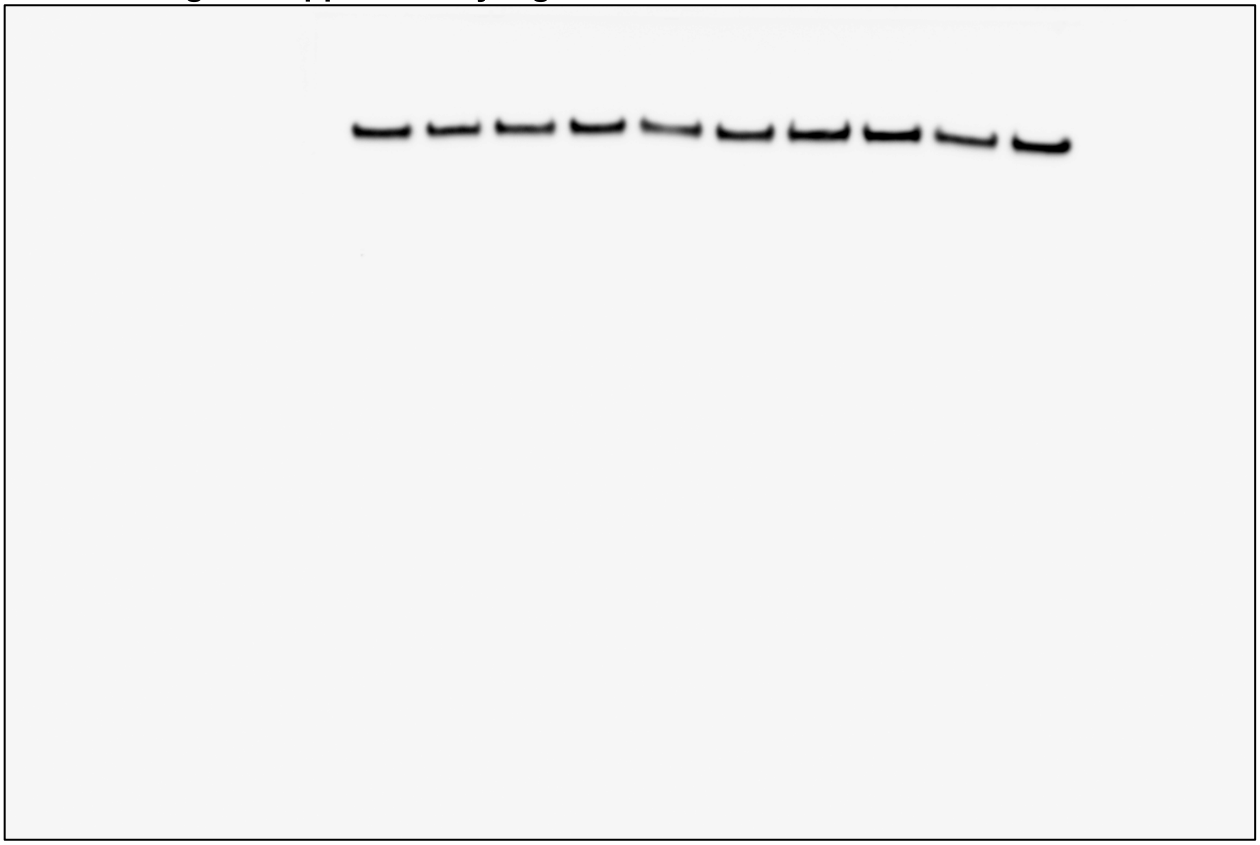

Uncropped Atp1a3 western blot from vehicle and PE-treated WT and E815K mice, related to Supplementary Figures 6L and 6N.

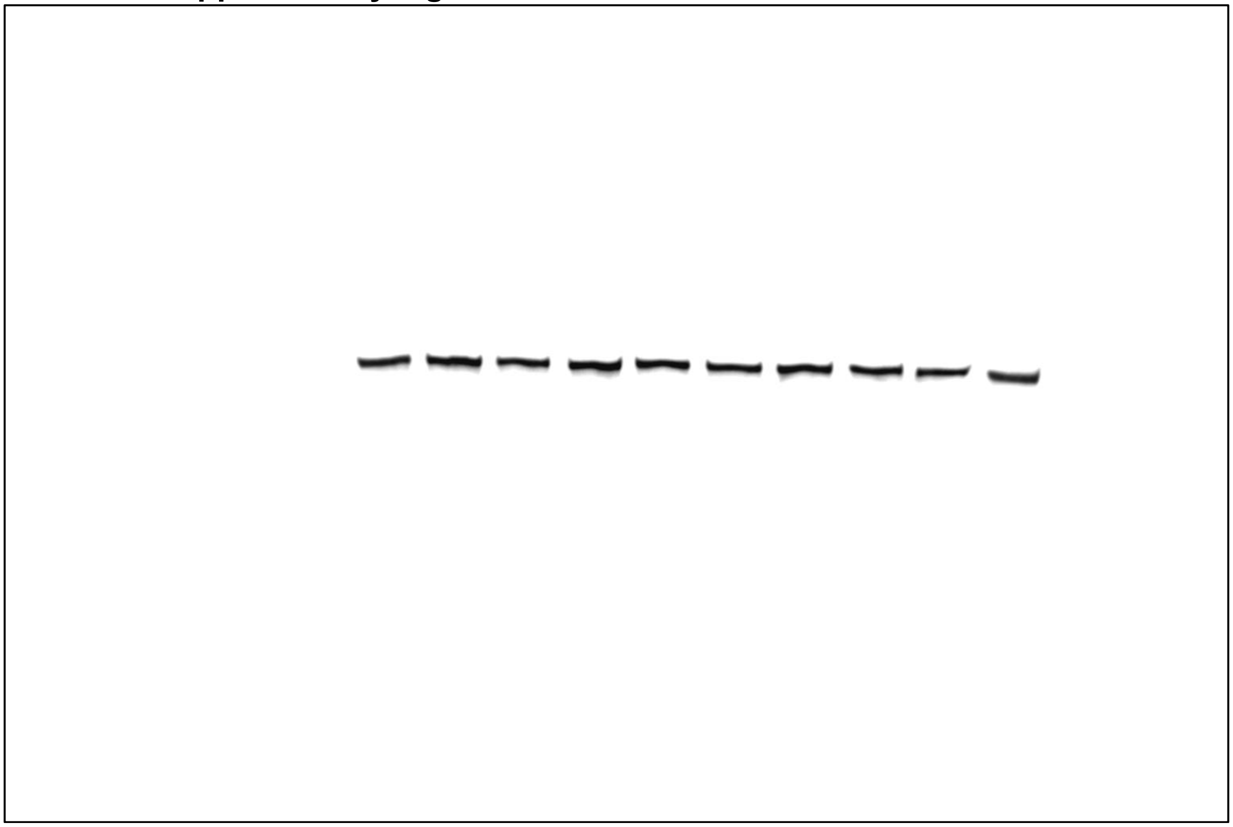

Supplementary Note S6. Uncropped GAPDH western blot from vehicle and PE-treated WT and E815K mice, related to Supplementary Figures 6L and 6N.

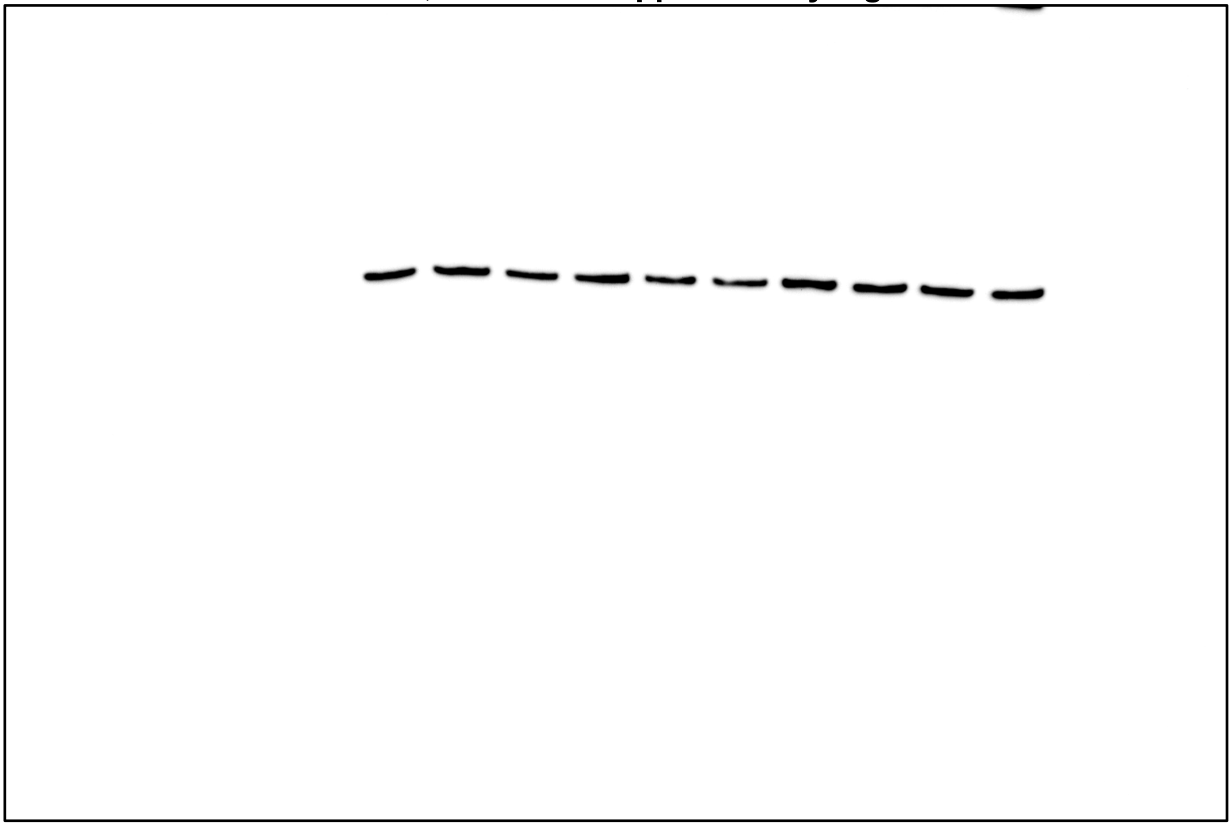

Supplement: Data S2 [file NIHMS2127386-supplement-Data_S2.pdf]
