# Supplementary material for: In vivo prime editing rescues alternating hemiplegia of childhood in mice
Source: Cell. Author manuscript; Available in PMC 2025 Dec 14. (PMC12702498; doi:10.1016/j.cell.2025.06.038)
Supplement: Methods S2 [file NIHMS2127386-supplement-Methods_S2.pdf]

Custom Python script for analysis of BE allelic outcomes, related to Figure 1 and Figure S1.

For analysis of **on-target adenine base editing** outcomes with A-to-G alleles:

```
import pandas as pd
import os
import glob
import re
import itertools

### change these parameters

folders = ['CRISPResso_batch_on_batch']
window_start = 1
window_end = 20

infer_ref_seq = True
ref_seq = 'TCTGAACACAAGAAGTGGATCCAT' #leave empty to infer this based on the -g sequence
used for CRISPResso

###

def generate_possible_alleles(ref_seq, window_start, window_end):
    A_positions = get_As_in_window(ref_seq, window_start, window_end)
    strings = [[ref_seq[:A_positions[0] - 1]], ['A', 'G']]
    for position in range(1,len(A_positions)):
        strings.append([ref_seq[A_positions[position - 1]:A_positions[position] - 1]])
        strings.append(['A', 'G'])
    strings.append([ref_seq[A_positions[-1]:]])
    possible_alleles = []
    for element in itertools.product(*strings):
        possible_alleles.append(''.join(str(i) for i in element))
    return possible_alleles

def get_As_in_window(seq, window_start, window_end):
    return [m.start() + 1 for m in re.finditer('A', seq) if m.start() + 1 >= window_start and m.start() + 1 <=
window_end]

def get_allele_frequencies(possible_alleles):
    data = pd.read_csv(glob.glob('*Alleles_frequency_table_around_sgRNA_*.txt')[0],delimiter='\t')
    #data = data.loc[data['%Reads'] > 0.1]
    allele_frequencies = []
    for allele in possible_alleles:
        if data.loc[data['Aligned_Sequence'].str.contains(allele)]['%Reads'].empty:
            allele_frequencies.append(0)
        else:

allele_frequencies.append(float(data.loc[data['Aligned_Sequence'].str.contains(allele)]['%Reads'].sum()))
    return allele_frequencies

def compare_allele_to_reference(ref_seq, allele):
    return [i + 1 for i in range(20) if ref_seq[i] != allele[i]]

original_dir = os.getcwd()
```

```
for folder in folders:
    os.chdir(folder)
    data_dict = {}
    #calculate alleles
    if infer_ref_seq:
        ref_seq = glob.glob("*sgRNA_*.pdf")[0].split("sgRNA_")[-1].split(".")[0]
    possible_alleles = generate_possible_alleles(ref_seq, window_start, window_end)
    data_dict['Alleles'] = possible_alleles
    #compare alleles to reference
    data_dict['Edited_bases'] = [compare_allele_to_reference(ref_seq, allele) for allele in possible_alleles]
    #get sample data for single folder
    data_dict[folder] = get_allele_frequencies(possible_alleles)

    #dataframe from dict
    output = pd.DataFrame.from_dict(data_dict)
    #sort column names
    cols = ['Alleles', 'Edited_bases', folder] #sorted(sample_names)
    output = output[cols]
    os.chdir(original_dir)
    output.to_csv('Allele_frequency_summary_' + folder + '.csv')
```

For analysis of **on-target cytosine base editing** outcomes with C-to-T alleles:

```
import pandas as pd
import os
import glob
import re
import itertools

### change these parameters

folders = ['CRISPResso_batch_on_batch']
window_start = 1
window_end = 20

infer_ref_seq = True
ref_seq = 'TCTGAACACAAGAAGTGGATCCAT' #leave empty to infer this based on the -g sequence
used for CRISPResso

###

def generate_possible_alleles(ref_seq, window_start, window_end):
    C_positions = get_Cs_in_window(ref_seq, window_start, window_end)
    strings = [[ref_seq[:C_positions[0] - 1]], ['C', 'T']]
    for position in range(1,len(C_positions)):
        strings.append([ref_seq[C_positions[position - 1]:C_positions[position] - 1]])
        strings.append(['C', 'T'])
    strings.append([ref_seq[C_positions[-1]:]])
    possible_alleles = []
    for element in itertools.product(*strings):
        possible_alleles.append(''.join(str(i) for i in element))
```

```
    return possible_alleles

def get_Cs_in_window(seq, window_start, window_end):
    return [m.start() + 1 for m in re.finditer('C', seq) if m.start() + 1 >= window_start and m.start() + 1 <=
window_end]

def get_allele_frequencies(possible_alleles):
    data = pd.read_csv(glob.glob('*Alleles_frequency_table_around_sgRNA_*.txt')[0],delimiter='\t')
    #data = data.loc[data['%Reads'] > 0.1]
    allele_frequencies = []
    for allele in possible_alleles:
        if data.loc[data['Aligned_Sequence'].str.contains(allele)]['%Reads'].empty:
            allele_frequencies.append(0)
        else:

allele_frequencies.append(float(data.loc[data['Aligned_Sequence'].str.contains(allele)]['%Reads'].sum()))
    return allele_frequencies

def compare_allele_to_reference(ref_seq, allele):
    return [i + 1 for i in range(20) if ref_seq[i] != allele[i]]

original_dir = os.getcwd()
original_dir = os.getcwd()
for folder in folders:
    os.chdir(folder)
    data_dict = {}
    #calculate alleles
    if infer_ref_seq:
        ref_seq = glob.glob("*sgRNA_*.pdf")[0].split("sgRNA_")[-1].split(".")[0]
    possible_alleles = generate_possible_alleles(ref_seq, window_start, window_end)
    data_dict['Alleles'] = possible_alleles
    #compare alleles to reference
    data_dict['Edited_bases'] = [compare_allele_to_reference(ref_seq, allele) for allele in possible_alleles]
    #get sample data for single folder
    data_dict[folder] = get_allele_frequencies(possible_alleles)

    #dataframe from dict
    output = pd.DataFrame.from_dict(data_dict)
    #sort column names
    cols = ['Alleles', 'Edited_bases', folder] #sorted(sample_names)
    output = output[cols]
    output.to_csv('Allele_frequency_summary_' + folder + '.csv')
    os.chdir(original_dir)
```

For analysis of **off-target adenine base editing** outcomes with A-to-G alleles:

```
#!/broad/liulabdata/CRISPRessoUGER/CRISPResso_env/bin/python

import pandas as pd
import os
import glob
import re
import itertools

### Change these parameters

# Assign 'folders' to a list of all folders in the current directory starting with "CRISPResso_on_"
# folders = [f for f in os.listdir('.') if os.path.isdir(f) and f.startswith("CRISPResso_on_")]
# Assign 'folders' by navigating into 'LocalAlignment', then into 'CRISPResso_batch_on_*_BE' folders,
and then collecting 'CRISPResso_on_*' folders
base_dir = os.path.join('.', 'LocalAlignment')
batch_dirs = [d for d in os.listdir(base_dir) if os.path.isdir(os.path.join(base_dir, d)) and
d.startswith('CRISPResso_batch_on_') and d.endswith('_BE')]
folders = []
for batch_dir in batch_dirs:
    batch_dir_path = os.path.join(base_dir, batch_dir)
    # Find all 'CRISPResso_on_*' directories inside each batch_dir
    crispr_dirs = [os.path.join(batch_dir_path, d) for d in os.listdir(batch_dir_path)
                   if os.path.isdir(os.path.join(batch_dir_path, d)) and d.startswith('CRISPResso_on_')]
    folders.extend(crispr_dirs)


window_start = 4  # Start position of the window
window_end = 10   # End position of the window

infer_ref_seq = True  # Whether to infer the reference sequence
ref_seq = ''  # Leave empty to infer based on the -g sequence used for CRISPResso

def reverse_complement(seq):
    """
    Computes the reverse complement of a DNA sequence.

    Parameters:
    - seq: DNA sequence string.

    Returns:
    - Reverse complement of the input sequence.
    """
    complement = str.maketrans('ATCGN', 'TAGCN')
    return seq.translate(complement)[::-1]

def generate_possible_alleles(ref_seq, window_start, window_end):
    """
    Generates all possible alleles by substituting 'A's in the reference sequence
    within the specified window with 'G's.

    Parameters:
```

```
    - ref_seq: Reference DNA sequence.
    - window_start: Start position of the substitution window.
    - window_end: End position of the window.

    Returns:
    - possible_alleles: List of all possible allele sequences.
    """
    # Get positions of 'A's within the specified window
    A_positions = get_As_in_window(ref_seq, window_start, window_end)
    if not A_positions:
        return [ref_seq]
    # Initialize the list of sequence fragments
    strings = []
    prev_pos = 0
    for pos in A_positions:
        # Append sequence before 'A'
        strings.append([ref_seq[prev_pos:pos-1]])
        # Append possible substitutions at 'A' positions
        strings.append(['A', 'G'])
        prev_pos = pos
    # Append the remaining sequence after the last 'A'
    strings.append([ref_seq[prev_pos:]])
    # Generate all combinations of sequence fragments
    possible_alleles = [''.join(seq) for seq in itertools.product(*strings)]
    return possible_alleles

def get_As_in_window(seq, window_start, window_end):
    """
    Finds positions of 'A's in the sequence within the specified window.

    Parameters:
    - seq: DNA sequence.
    - window_start: Start position of the window.
    - window_end: End position of the window.

    Returns:
    - List of positions (1-based indexing) of 'A's in the window.
    """
    return [m.start() + 1 for m in re.finditer('A', seq) if window_start <= m.start() + 1 <= window_end]

def compare_allele_to_reference(ref_seq, allele):
    """
    Compares an allele sequence to the reference sequence and returns positions of differences.

    Parameters:
    - ref_seq: Reference DNA sequence.
    - allele: Allele DNA sequence.

    Returns:
    - List of positions (1-based indexing) where the allele differs from the reference.
    """
    return [i + 1 for i in range(len(ref_seq)) if ref_seq[i] != allele[i]]

def get_allele_frequencies(possible_alleles, data, orientation='direct'):
    """
    Retrieves the frequencies of the possible alleles by searching for any occurrence
```

```
    of each possible allele within the 'Aligned_Sequence' column, and summing the '%Reads'.

    Parameters:
    - possible_alleles: List of possible allele sequences (substrings).
    - data: DataFrame containing 'Aligned_Sequence' and '%Reads'.
    - orientation: 'direct' or 'reverse' indicating the orientation to use.

    Returns:
    - allele_frequencies: List of frequencies corresponding to each allele.
    """
    # Ensure 'Aligned_Sequence' is a string and uppercase
    data['Aligned_Sequence'] = data['Aligned_Sequence'].astype(str).str.upper()
    # Remove non-ATCG characters from 'Aligned_Sequence'
    data['Aligned_Sequence'] = data['Aligned_Sequence'].str.replace('[^ATCG]', '', regex=True)

    # Process possible_alleles: uppercase and remove non-ATCG characters
    processed_alleles = [re.sub('[^ATCG]', '', allele.upper()) for allele in possible_alleles]
    if orientation == 'reverse':
        # Reverse complement the alleles if orientation is reverse
        processed_alleles = [reverse_complement(allele) for allele in processed_alleles]

    allele_frequencies = []
    for allele in processed_alleles:
        # Escape special characters in allele sequences
        escaped_allele = re.escape(allele)
        # Create a boolean mask where 'Aligned_Sequence' contains the allele substring
        mask = data['Aligned_Sequence'].str.contains(escaped_allele, na=False)
        # Sum the '%Reads' for all sequences that contain the allele substring
        frequency = data.loc[mask, '%Reads'].sum()
        allele_frequencies.append(frequency)
    return allele_frequencies

def determine_orientation(ref_seq, data):
    """
    Determines whether the sequences in 'Aligned_Sequence' are in the same orientation
    as ref_seq or its reverse complement, based on which orientation results in the
    higher sum of '%Reads'.

    Parameters:
    - ref_seq: Reference DNA sequence.
    - data: DataFrame containing 'Aligned_Sequence' and '%Reads'.

    Returns:
    - orientation: 'direct' or 'reverse' indicating the correct orientation.
    """
    # Ensure 'Aligned_Sequence' is a string and uppercase
    data['Aligned_Sequence'] = data['Aligned_Sequence'].astype(str).str.upper()
    # Remove non-ATCG characters from 'Aligned_Sequence'
    data['Aligned_Sequence'] = data['Aligned_Sequence'].str.replace('[^ATCG]', '', regex=True)

    # Process ref_seq: uppercase and remove non-ATCG characters
    processed_ref_seq = re.sub('[^ATCG]', '', ref_seq.upper())
    reverse_ref_seq = reverse_complement(processed_ref_seq)

    # Calculate sum of '%Reads' for ref_seq
    mask_direct = data['Aligned_Sequence'].str.contains(re.escape(processed_ref_seq), na=False)
```

```
    sum_direct = data.loc[mask_direct, '%Reads'].sum()

    # Calculate sum of '%Reads' for reverse complement of ref_seq
    mask_reverse = data['Aligned_Sequence'].str.contains(re.escape(reverse_ref_seq), na=False)
    sum_reverse = data.loc[mask_reverse, '%Reads'].sum()

    # Determine orientation
    if sum_direct >= sum_reverse:
        orientation = 'direct'
    else:
        orientation = 'reverse'

    print(f"Orientation determined: {orientation} (Direct sum: {sum_direct}, Reverse sum: {sum_reverse})")
    return orientation

if __name__ == '__main__':
    # Save the current working directory
    original_dir = os.getcwd()
    print("Currently in", os.getcwd())

    # Initialize a dictionary to store the BE summary
    be_summary_dict = {}

    # Loop through each folder in the 'folders' list
    for folder in folders:
        print('Tabulating for ' + folder + '...')
        folder_path = os.path.join(original_dir, folder)
        data_dict = {}  # Initialize a dictionary to store data

        # Infer the reference sequence if 'infer_ref_seq' is True
        if infer_ref_seq:
            sgRNA_pdf_files = glob.glob(os.path.join(folder_path, "*sgRNA_*.pdf"))
            if not sgRNA_pdf_files:
                print(f"No sgRNA PDF file found in {folder_path}")
                continue
            ref_seq = sgRNA_pdf_files[0].split("sgRNA_")[-1].split(".")[0]

        # Generate possible alleles
        possible_alleles = generate_possible_alleles(ref_seq, window_start, window_end)
        data_dict['Alleles'] = possible_alleles

        # Compare alleles to reference
        data_dict['Edited_bases'] = [compare_allele_to_reference(ref_seq, allele) for allele in
possible_alleles]

        # Read the allele frequency table file
        allele_freq_files = glob.glob(os.path.join(folder_path,
'*Alleles_frequency_table_around_sgRNA_*.txt'))
        if not allele_freq_files:
            print(f"No allele frequency table found in {folder_path}")
            continue
        data = pd.read_csv(allele_freq_files[0], delimiter='\t')

        # Determine the correct orientation
        orientation = determine_orientation(ref_seq, data)
```

```
        # Get allele frequencies using the correct orientation
        data_dict[folder] = get_allele_frequencies(possible_alleles, data, orientation)

        # Create DataFrame from data_dict
        output = pd.DataFrame.from_dict(data_dict)

        # Calculate sum_unedited and sum_edited directly from data_dict
        # Identify unedited alleles where Edited_bases is an empty list
        unedited_mask = output['Edited_bases'].apply(lambda x: len(x) == 0)
        sum_unedited = output.loc[unedited_mask, folder].sum()

        # Edited alleles are those where Edited_bases is not empty
        edited_mask = output['Edited_bases'].apply(lambda x: len(x) != 0)
        sum_edited = output.loc[edited_mask, folder].sum()

        # Create summary DataFrame
        summary_df = pd.DataFrame({
            'Alleles': ['Unedited', 'Base edited'],
            'Edited_bases': ['none', 'any'],
            folder: [sum_unedited, sum_edited]
        })

        # Append summary_df to output
        output = pd.concat([output, summary_df], ignore_index=True)

        # Define the order of columns in the output DataFrame
        cols = ['Alleles', 'Edited_bases', folder]
        output = output[cols]

        # Save the DataFrame to a CSV file
        output_file = os.path.join(folder_path, 'Allele_frequency_summary_ABE.csv')
        output.to_csv(output_file, index=False)

        # Add the 'Unedited' and 'Base edited' values to the BE summary dictionary
        be_summary_dict[folder] = (sum_unedited, sum_edited)

    # After processing all folders, create the BE_Summary.csv file
    # Create a DataFrame from the be_summary_dict
    be_summary_df = pd.DataFrame.from_dict(be_summary_dict, orient='index', columns=['Unedited',
'Base edited'])

    # Save the summary DataFrame to a CSV file
    be_summary_df.index.name = 'Folder'
    be_summary_df.to_csv('BE_Summary.csv')

    print("Processing completed. BE_Summary.csv file has been created.")
```

Custom BASH script for demultiplexing rhAmpSeq libraries into separated FASTQ files for each amplicon, related to Figure 2.

The following script was executed in a folder containing Bowtie2-indexed reference files built for each rhAmpSeq library containing the expected amplicon sequences extracted from the hg19 genome assembly.

```
#!/bin/bash
#$ -N full_pipeline
#$ -l h_vmem=8G
#$ -cwd
#$ -q broad
#$ -l h_rt=12:00:00
#$ -pe smp 4
#$ -R y
#$ -binding linear:4
#$ -j y
#$ -o qsub_out/redi.o$JOB_ID

# --- Step 1: Bowtie2 Alignment ---
if [ $# -ne 1 ]; then
  echo "Usage: $0 <input.fastq.gz>"
  exit 1
fi

# Load necessary modules
source /broad/software/scripts/useuse
reuse UGER
reuse Bowtie2
reuse Samtools

export R1=$1
export PREFIX=$(basename $R1)
export PREFIX=${PREFIX/\.fastq\.gz/}

echo "Working directory: $PWD"
echo "Processing file: $R1"

# Run Bowtie2 to align reads and generate SAM file
echo "Running Bowtie2..."
bowtie2 -x ref -U $R1 -S $PREFIX".sam" --threads 4 --very-fast-local
if [ $? -ne 0 ]; then
  echo "Error during Bowtie2 alignment."
  exit 1
fi
echo "Bowtie2 alignment completed. SAM file generated: $PREFIX.sam"

# --- Step 2: Samtools Sorting and Indexing ---
echo "Converting SAM to BAM and sorting..."
samtools view -@ 4 -S -b ${PREFIX}.sam > ${PREFIX}.bam
samtools sort -@ 4 ${PREFIX}.bam -o ${PREFIX}.sorted.bam
if [ $? -ne 0 ]; then
  echo "Error during SAM to BAM conversion or sorting."
  exit 1
```

```
fi

echo "Indexing BAM file..."
samtools index ${PREFIX}.sorted.bam

echo "Generating idxstats..."
samtools idxstats ${PREFIX}.sorted.bam > ${PREFIX}.txt
if [ $? -ne 0 ]; then
    echo "Error generating idxstats."
    exit 1
fi

# Clean up intermediate files
rm ${PREFIX}.sam ${PREFIX}.bam
echo "SAM to sorted BAM conversion completed. Output: ${PREFIX}.sorted.bam"

# --- Step 3: Split BAM by Reference and Convert to FASTQ ---
echo "Splitting BAM by reference and converting to FASTQ..."

output_folder="./${PREFIX}-split-fastqs"
mkdir -p ${output_folder}

# Create reference file
reference_file="${output_folder}/${PREFIX}.txt"
samtools idxstats ${PREFIX}.sorted.bam | cut -f1 | grep -v '*' > ${reference_file}

if [ ! -s ${reference_file} ]; then
    echo "Error: No reference sequences found in the BAM header."
    rm -f ${reference_file}
    exit 1
fi

# Process each reference sequence
reference_sequences=$(cat ${reference_file})
for ref in $reference_sequences; do
    mini_bam="${output_folder}/${ref}.bam"
    fastq_file="${output_folder}/${PREFIX}_${ref}.fastq.gz"

    # Extract reads and convert to FASTQ
    samtools view -h -b ${PREFIX}.sorted.bam ${ref} > ${mini_bam}
    samtools fastq ${mini_bam} | gzip > "${fastq_file}"

    # Remove mini BAM file
    rm -f ${mini_bam}
    echo "Processed reference ${ref}. FASTQ saved to ${fastq_file}"
done

# Clean up reference file
rm -f ${reference_file}
echo "BAM splitting and FASTQ conversion completed."
```
