# Supplementary material for: In vivo prime editing rescues alternating hemiplegia of childhood in mice
Source: Cell. Author manuscript; Available in PMC 2025 Dec 14. (PMC12702498; doi:10.1016/j.cell.2025.06.038)
Supplement: Methods S1 [file NIHMS2127386-supplement-Methods_S1.pdf]

Complete DNA and protein sequences of base editors, prime editors, and AAV constructs used in this study, related to Figures 1-6.

D801N-PE-AAV9; N-Term
Plasmid ID: LpAS7267

DNA sequence
CTGCGCGCTCGCTCGCTCACTGAGGCCGCCCGGGCAAAGCCCGGGCGTCGGGCGACCTTTGGTC
GCCCGGCCTCAGTGAGCGAGCGAGCGCGCAGAGAGGGAGTGGCCAACTCCATCACTAGGGGTTCC
TGCGGCCTCTAGAATTCGCTAGCTAGGTCTTGAAAGGAGTGGGAATTGGCTCCGGTGCCCGTCAGT
GGGCAGAGCGCACATCGCCCACAGTCCCCGAGAAGTTGGGGGGAGGGGTCGGCAATTGATCCGGT
GCCTAGAGAAGGTGGCGCGGGGTAAACTGGGAAAGTGATGTCGTGTACTGGCTCCGCCTTTTTCCC
GAGGGTGGGGGAGAACCGTATATAAGTGCAGTAGTCGCCGTGAACGTTCTTTTTCGCAACGGGTTT
GCCGCCAGAACACAGGACCGGTGCCACCATGAAACGGACAGCCGACGGAAGCGAGTTCGAGTCAC
CAAAGAAGAAGCGGAAAGTCGACAAGAAGTACAGCATCGGCCTGGACATCGGCACCAACTCTGTGG
GCTGGGCCGTGATCACCGACGAGTACAAGGTGCCCAGCAAGAAATTCAAGGTGCTGGGCAACACC
GACCGGCACAGCATCAAGAAGAACCTGATCGGAGCCCTGCTGTTCGACAGCGGCGAAACAGCCGA
GGCCACCCGGCTGAAGAGAACCGCCAGAAGAAGATACACCAGACGGAAGAACCGGATCTGCTATCT
GCAAGAGATCTTCAGCAACGAGATGGCCAAGGTGGACGACAGCTTCTTCCACAGACTGGAAGAGTC
CTTCCTGGTGGAAGAGGATAAGAAGCACGAGCGGCACCCCATCTTCGGCAACATCGTGGACGAGGT
GGCCTACCACGAGAAGTACCCCACCATCTACCACCTGAGAAAGAAACTGGTGGACAGCACCGACAA
GGCCGACCTGCGGCTGATCTATCTGGCCCTGGCCCACATGATCAAGTTCCGGGGCCACTTCCTGAT
CGAGGGCGACCTGAACCCCGACAACAGCGACGTGGACAAGCTGTTCATCCAGCTGGTGCAGACCTA
CAACCAGCTGTTCGAGGAAAACCCCATCAACGCCAGCGGCGTGGACGCCAAGGCCATCCTGTCTGC
CAGACTGAGCAAGAGCAGAAAGCTGGAAAATCTGATCGCCCAGCTGCCCGGCGAGAAGAAGAATGG
CCTGTTCGGAAACCTGATTGCCCTGAGCCTGGGCCTGACCCCCAACTTCAAGAGCAACTTCGACCT
GGCCGAGGATGCCAAACTGCAGCTGAGCAAGGACACCTACGACGACGACCTGGACAACCTGCTGG
CCCAGATCGGCGACCAGTACGCCGACCTGTTTCTGGCCGCCAAGAACCTGTCCGACGCCATCCTGC
TGAGCGACATCCTGAGAGTGAACACCGAGATCACCAAGGCCCCCCTGAGCGCCTCTATGATCAAGA
GATACGACGAGCACCACCAGGACCTGACCCTGCTGAAAGCTCTCGTGCGGCAGCAGCTGCCTGAG
AAGTACAAAGAGATTTTCTTCGACCAGAGCAAGAACGGCTACGCCGGCTACATTGACGGCGGAGCC
AGCCAGGAAGAGTTCTACAAGTTCATCAAGCCCATCCTGGAAAAGATGGACGGCACCGAGGAACTG
CTCGTGAAGCTGAAGAGAGAGGACCTGCTGCGGAAGCAGCGGACCTTCGACAACGGCAGCATCCC
CCACCAGATCCACCTGGGAGAGCTGCACGCCATTCTGCGGCGGCAGGAAGATTTTTACCCATTCCT
GAAGGACAACCGGGAAAAGATCGAGAAGATCCTGACCTTCCGCATCCCCTACTACGTGGGCCCTCT
GGCCAGGGGAAACAGCAGATTCGCCTGGATGACCAGAAAGAGCGAGGAAACCATCACCCCCTGGA
ACTTCGAGGAAGTGGTGGACAAGGGCGCTTCCGCCCAGAGCTTCATCGAGCGGATGACCAACTTCG
ATAAGAACCTGCCCAACGAGAAGGTGCTGCCCAAGCACAGCCTGCTGTACGAGTACTTCACCGTGT
ATAACGAGCTGACCAAAGTGAAATACGTGACCGAGGGAATGAGAAAGCCCGCCTTCCTGAGCGGCG
AGCAGAAAAAGGCCATCGTGGACCTGCTGTTCAAGACCAACCGGAAAGTGACCGTGAAGCAGCTGA
AAGAGGACTACTTCAAGAAAATCGAGTGCTTCGACTCCGTGGAAATCTCCGGCGTGGAAGATCGGTT
CAACGCCTCCCTGGGCACATACCACGATCTGCTGAAAATTATCAAGGACAAGGACTTCCTGGACAAT
GAGGAAAACGAGGACATTCTGGAAGATATCGTGCTGACCCTGACACTGTTTGAGGACAGAGAGATG
ATCGAGGAACGGCTGAAAACCTATGCCCACCTGTTCGACGACAAAGTGATGAAGCAGCTGAAGCGG
CGGAGATACACCGGCTGGGGCAGGCTGAGCCGGAAGCTGATCAACGGCATCCGGGACAAGCAGTC
CGGCAAGACAATCCTGGATTTCCTGAAGTCCGACGGCTTCGCCAACAGAAACTTCATGCAGCTGATC
CACGACGACAGCCTGACCTTTAAAGAGGACATCCAGAAAGCCCAGGTGTCCGGCCAGGGCGATAGC
CTGCACGAGCACATTGCCAATCTGGCCGGCAGCCCCGCCATTAAGAAGGGCATCCTGCAGACAGTG
AAGGTGGTGGACGAGCTCGTGAAAGTGATGGGCCGGCACAAGCCCGAGAACATCGTGATCGAAAT
GGCCAGAGAGAACCAGACCACCCAGAAGGGACAGAAGAACAGCCGCGAGAGAATGAAGCGGATCG
AAGAGGGCATCAAAGAGCTGGGCAGCCAGATCCTGAAAGAACACCCCGTGGAAAACACCCAGCTGC
AGAACGAGAAGCTGTACCTGTACTACCTGCAGAATGGGCGGGATATGTACGTGGACCAGGAACTGG
ACATCAACCGGCTGTCCGACTACGATGTGGACGCTATCGTGCCTCAGAGCTTTCTGAAGGACGACT
CCATCGACAACAAGGTGCTGACCAGAAGCGACAAGAACCGGGGCAAGAGCGACAACGTGCCCTCC
GAAGAGGTCGTGAAGAAGATGAAGAACTACTGGCGGCAGCTGCTGAACGCCAAGCTGATTACCCAG

AGAAAGTTCGACAATCTGACCAAGGCCGAGAGAGGCGGCCTGAGCGAACTGGATAAGGCCGGCTT
CATCAAGAGACAGCTGGTGGAAACCCGGCAGATCACAAAGCACGTGGCACAGATCCTGGACTCCCG
GATGAACACTAAGTACGACGAGAATGACAAGCTGATCCGGGAAGTGAAAGTGATCACCCTGAAGTC
CAAGCTGGTGTCCGATTTCCGGAAGGATTTCCAGTTTTACAAAGTGCGCGAGATCAACAACTACCAC
CACGCCCACGACGCCTACCTGAACGCCGTCGTGGGAACCGCCCTGATCAAAAAGTACCCTAAGCTG
GAAAGCGAGTTCGTGTACGGCGACTACAAGGTGTACGACGTGCGGAAGATGATCGCCAAGTGCCTG
TCCTACGAGACAGAGATCCTGACAGTGGAGTATGGCCTGCTGCCAATCGGCAAGATCGTGGAGAAG
AGGATCGAGTGTACCGTGTACTCTGTGGATAACAATGGCAACATCTATACACAGCCCGTGGCACAGT
GGCACGATAGGGGAGAGCAGGAGGTGTTCGAGTATTGCCTGGAGGACGGCAGCCTGATCAGGGCA
ACCAAGGACCACAAGTTCATGACAGTGGATGGCCAGATGCTGCCCATCGACGAGATTTTCGAGCGG
GAGCTGGACCTGATGAGAGTGGATAACCTGCCTAATTCTGGCGGCTCAAAAAGAACCGCCGACGGC
AGCGAATTCGAGAGTCCCAAGAAGAAGAGGAAAGTCTAAGATCTGATAATCAACCTCTGGATTACAA
AATTTGTGAAAGATTGACTGGTATTCTTAACTATGTTGCTCCTTTTACGCTATGTGGATACGCTGCTTT
AATGCCTTTGTATCATGCTATTGCTTCCCGTATGGCTTTCATTTTCTCCTCCTTGTATAAATCCTGGTT
AGTTCTTGCCACGGCGGAACTCATCGCCGCCTGCCTTGCCCGCTGCTGGACAGGGGCTCGGCTGTT
GGGCACTGACAATTCCGTGGTGCGACTGTGCCTTCTAGTTGCCAGCCATCTGTTGTTTGCCCCTCCC
CCGTGCCTTCCTTGACCCTGGAAGGTGCCACTCCCACTGTCCTTTCCTAATAAAATGAGGAAATTGC
ATCGCATTGTCTGAGTAGGTGTCATTCTATTCTGGGGGGTGGGGTGGGGCAGGACAGCAAGGGGG
AGGATTGGGAAGACAATAGCAGGCATGCTGGGGATGCGGTGGGCTCTATGGCTCGAGAAAAAAAGC
ACCGACTCGGTGCCACTTTTTCAAGTTGATAACGGACTAGCCTTATTTGAACTTGCTATGCTGTTTCC
AGCATAGCTCTCAAACCCACACGCTTGCGCCGGTGTTTCGTCCTTTCCACAAGATATATAAAGCCAA
GAAATCGAAATACTTTCAAGTTACGGTAAGCATATGATAGTCCATTTTAAAACATAATTTTAAAACTGC
AAACTACCCAAGAAATTATTACTTTCTACGTCACGTATTTTGTACTAATATCTTTGTGTTTACAGTCAAA
TTAATTCTAATTATCTCTCTAACAGCCTTGTATCGTATATGCAAATATGAAGGAATCATGGGAAATAGG
CCCTCTTCCTGCCCGACCTTGCGGCCGCAGGAACCCCTAGTGATGGAGTTGGCCACTCCCTCTCTG
CGCGCTCGCTCGCTCACTGAGGCCGGGCGACCAAAGGTCGCCCGACGCCCGGGCTTTGCCCGGG
CGGCCTCAGTGAGCGAGCGAGCGCGCAG

Start	End	Feature Description
1	130	5’ inverted terminal repeat (ITR), pSub201(+)
153	407	EFS promoter for transcription initiation
421	477	SV40 nuclear localization sequence
478	3546	Sp Cas9 N-terminal fragment, PEmax architecture
3547	3852	NpuN, N-terminal fragment of trans splicing intein
3853	3864	Flexible linker
3865	3921	SV40 nuclear localization sequence
3927	4087	WHV Posttranscriptional regulatory element (WPRE), gamma
4176	4386	BGH polyA signal
4416	4410	RNA polymerase III terminator
4502	4417	Cas9 sgRNA scaffold sequence
4503	4517	m.*Atp1a3* D801N locus dead sgRNA spacer (reverse complement)
4518	4781	U6 promoter (reverse complement)
4790	4919	3’ inverted terminal repeat (ITR), pSub201(+)

Amino acid sequence

MKRTADGSEFESPKKKRKVDKKYSIGLDIGTNSVGWAVITDEYKVPSKKFKVLGNTDRHSIKKNLIGALLF
DSGETAEATRLKRTARRRYTRRKNRICYLQEIFSNEMAKVDDSFFHRLEESFLVEEDKKHERHPIFGNIVD
EVAYHEKYPTIYHLRKKLVDSTDKADLRLIYLALAHMIKFRGHFLIEGDLNPDNSDVDKLFIQLVQTYNQLFE
ENPINASGVDAKAILSARLSKSRKLENLIAQLPGEKKNGLFGNLIALSLGLTPNFKSNFDLAEDAKLQLSKD

TYDDDLDNLLAQIGDQYADLFLAAKNLSDAILLSDILRVNTEITKAPLSASMIKRYDEHHQDLTLLKALVRQQ
LPEKYKEIFFDQSKNGYAGYIDGGASQEEFYKFIKPILEKMDGTEELLVKLKREDLLRKQRTFDNGSIPHQI
HLGELHAILRRQEDFYPFLKDNREKIEKILTFRIPYYVGPLARGNSRFAWMTRKSEETITPWNFEEVVDKG
ASAQSFIERMTNFDKNLPNEKVLPKHSLLYEYFTVYNELTKVKYVTEGMRKPAFLSGEQKKAIVDLLFKTN
RKVTVKQLKEDYFKKIECFDSVEISGVEDRFNASLGTYHDLLKIIKDKDFLDNEENEDILEDIVLTLTLFEDR
EMIEERLKTYAHLFDDKVMKQLKRRRYTGWGRLSRKLINGIRDKQSGKTILDFLKSDGFANRNFMQLIHD
DSLTFKEDIQKAQVSGQGDSLHEHIANLAGSPAIKKGILQTVKVVDELVKVMGRHKPENIVIEMARENQTT
QKGQKNSRERMKRIEEGIKELGSQILKEHPVENTQLQNEKLYLYYLQNGRDMYVDQELDINRLSDYDVDA
IVPQSFLKDDSIDNKVLTRSDKNRGKSDNVPSEEVVKKMKNYWRQLLNAKLITQRKFDNLTKAERGGLSE
LDKAGFIKRQLVETRQITKHVAQILDSRMNTKYDENDKLIREVKVITLKSKLVSDFRKDFQFYKVREINNYH
HAHDAYLNAVVGTALIKKYPKLESEFVYGDYKVYDVRKMIAKCLSYETEILTVEYGLLPIGKIVEKRIECTVY
SVDNNGNIYTQPVAQWHDRGEQEVFEYCLEDGSLIRATKDHKFMTVDGQMLPIDEIFERELDLMRVDNL
PNSGGSKRTADGSEFESPKKKRKV

Start	End	Feature Description
1	19	SV40 nuclear localization sequence
20	1042	Sp Cas9 N-terminal fragment, PEmax architecture
1043	1144	NpuN, N-terminal fragment of trans splicing intein
1145	1148	Flexible linker
1149	1166	SV40 nuclear localization sequence

D801N-PE-AAV9; C-term
Plasmid ID: LpAS7269

DNA sequence
CTGCGCGCTCGCTCGCTCACTGAGGCCGCCCGGGCAAAGCCCGGGCGTCGGGCGACCTTTGGTC
GCCCGGCCTCAGTGAGCGAGCGAGCGCGCAGAGAGGGAGTGGCCAACTCCATCACTAGGGGTTCC
TGCGGCCTCTAGAATTCGCTAGCTAGGTCTTGAAAGGAGTGGGAATTGGCTCCGGTGCCCGTCAGT
GGGCAGAGCGCACATCGCCCACAGTCCCCGAGAAGTTGGGGGGAGGGGTCGGCAATTGATCCGGT
GCCTAGAGAAGGTGGCGCGGGGTAAACTGGGAAAGTGATGTCGTGTACTGGCTCCGCCTTTTTCCC
GAGGGTGGGGGAGAACCGTATATAAGTGCAGTAGTCGCCGTGAACGTTCTTTTTCGCAACGGGTTT
GCCGCCAGAACACAGGACCGGTGCCACCATGAAACGGACAGCCGACGGAAGCGAGTTCGAGTCAC
CAAAGAAGAAGCGGAAAGTCATCAAGATTGCTACACGGAAATACCTGGGAAAGCAGAACGTGTACG
ACATCGGCGTGGAGCGGGATCACAACTTCGCCCTGAAGAATGGCTTTATCGCCAGCAATTGTTTCAA
CGAAATCGGCAAGGCTACCGCCAAGTACTTCTTCTACAGCAACATCATGAACTTTTTCAAGACCGAG
ATTACCCTGGCCAACGGCGAGATCCGGAAGCGGCCTCTGATCGAGACAAACGGCGAAACCGGGGA
GATCGTGTGGGATAAGGGCCGGGATTTTGCCACCGTGCGGAAAGTGCTGAGCATGCCCCAAGTGAA
TATCGTGAAAAAGACCGAGGTGCAGACAGGCGGCTTCAGCAAAGAGTCTATCCTGCCCAAGAGGAA
CAGCGATAAGCTGATCGCCAGAAAGAAGGACTGGGACCCTAAGAAGTACGGCGGCTTCGACAGCCC
CACCGTGGCCTATTCTGTGCTGGTGGTGGCCAAAGTGGAAAAGGGCAAGTCCAAGAAACTGAAGAG
TGTGAAAGAGCTGCTGGGGATCACCATCATGGAAAGAAGCAGCTTCGAGAAGAATCCCATCGACTTT
CTGGAAGCCAAGGGCTACAAAGAAGTGAAAAAGGACCTGATCATCAAGCTGCCTAAGTACTCCCTGT
TCGAGCTGGAAAACGGCCGGAAGAGAATGCTGGCCTCTGCCGGCGAACTGCAGAAGGGAAACGAA
CTGGCCCTGCCCTCCAAATATGTGAACTTCCTGTACCTGGCCAGCCACTATGAGAAGCTGAAGGGCT
CCCCCGAGGATAATGAGCAGAAACAGCTGTTTGTGGAACAGCACAAGCACTACCTGGACGAGATCA
TCGAGCAGATCAGCGAGTTCTCCAAGAGAGTGATCCTGGCCGACGCTAATCTGGACAAAGTGCTGT
CCGCCTACAACAAGCACCGGGATAAGCCCATCAGAGAGCAGGCCGAGAATATCATCCACCTGTTTA
CCCTGACCAATCTGGGAGCCCCTGCCGCCTTCAAGTACTTTGACACCACCATCGACCGGAAGAGGT
ACACCAGCACCAAAGAGGTGCTGGACGCCACCCTGATCCACCAGAGCATCACCGGCCTGTACGAGA
CACGGATCGACCTGTCTCAGCTGGGAGGTGACTCCGGCGGAAGCTCTGGTGGCAGCAAGCGGACC
GCCGACGGCTCTGAATTCGAGAGCCCTAAGAAGAAAAGAAAGGTGAGCGGAGGCTCTAGCGGCGG
AAGCATCAGCAGCTCTAAGCACACCCTGAGCCAGATGAACAAGGTGAGCAACATCGTGAAGGAACC
CGAGCTGCCCGACATCTACAAGGAATTTAAGGACATCACCGCCGACACCAATACCGAGAAGCTGCC
TAAGCCTATTAAGGGCCTGGAATTTGAAGTGGAACTGACACAGGAGAACTACAGACTGCCTATCCGG
AACTATCCTCTGACTCCAGTCAAGATGCAGGCCATGAACGACGAGATCAATCAAGGCCTGAAAGGC
GGCATCATCAGAGAGAGCAAGGCCATCAACGCCTGCCCTGTTATATTCGTGCCCAGAAAGGAAGGC
ACACTGCGCATGGTGGTCGACTACAGGCCCCTGAACAAGTACGTGAAGCCCAACGTCTACCCCCTG
CCACTGATTGAACAACTGCTGGCCAAGATCCAGGGCAGCACCATTTTCACCAAGCTGGACCTGAAAA
GCGCCTACCACCAGATCAGAGTGCGAAAGGGAGATGAGCACAAGCTGGCCTTCCGGTGCCCCAGA
GGAGTCTTCGAGTACCTGGTGATGCCTTACGGCATCAAGACAGCCCCTGCCCACTTTCAGTACTTCA
TCAACACAATCCTGGGCGAGGCCAAGGAAAGCCACGTGGTGTGCTACATGGACGACATCCTGATCC
ATTCCAAGTCCGAGTCCGAACACGTGAAACATGTGAAGGACGTGCTGCAAAAGCTGAAGAACGCTAA
TCTGATCATCAACCAGGCCAAATGCGAGTTTCACCAGAGCCAAGTGAAGTTCCTGGGCTACCACATC
AGCGAGAAGGGCTTAACCCCATGTCAGGAGAACATCGACAAGGTGCTGCAGTGGAAACAGCCTAAA
AACCAGAAGGAACTGAGACAGTTCCTGGGCCAGGTGAACTACCTGAGAAAATTCATCCCCAAAACCA
GCCAGTTGACCCACCCTCTGAACAAACTCCTGAAAAAGGATGTCAGATGGAAATGGACCCCTACCCA
GACCCAGGCTATCGAGAATATCAAGCAATGTCTGGTGTCTCCTCCTGTGCTGAGGCACTTCGACTTC
AGCAAGAAGATCCTGCTTGAGACAGACGTTTCTGATGTGGCCGTGGGAGCCGTGCTGAGCCAGAAG
CATGATGATGATAAGTACTACCCTGTGGGCTATTACAGCGCTAAAATGAGCAAAGCCCAGCTGAATT
ATTCTGTGTCCGACAAGGAGATGCTGGCTATCATCAAAAGCCTGGAGCACTGGCGGCACTACCTGG
AATCTACAATCGAGCCCTTCAAGATCCTGACCGACCACAGAAACCTGATTGGAAGAATCACAAACGA
GAGCGAACCAGAGAACAAGCGGCTGGCCAGATGGCAGCTGTTCCTGCAGGACTTCAACTTCGAGAT
CAACTACAGACCTGGCTCTGCAAATCACATCGCCGATGCCCTGTCTAGAATCGTGGACGAGACTGA
GCCTATCCCTAAGGACAACGAAGATAACAGCATCAACTTCGTGAACCAGATCAGCATCTCTGGCGGC
TCAAAAAGAACCGCCGACGGCAGCGAATTCGAGTCTCCCAAGAAGAAGAGGAAAGTCTAAGATCTG
ATAATCAACCTCTGGATTACAAAATTTGTGAAAGATTGACTGGTATTCTTAACTATGTTGCTCCTTTTA
CGCTATGTGGATACGCTGCTTTAATGCCTTTGTATCATGCTATTGCTTCCCGTATGGCTTTCATTTTCT

CCTCCTTGTATAAATCCTGGTTAGTTCTTGCCACGGCGGAACTCATCGCCGCCTGCCTTGCCCGCTG
CTGGACAGGGGCTCGGCTGTTGGGCACTGACAATTCCGTGGTGCGACTGTGCCTTCTAGTTGCCAG
CCATCTGTTGTTTGCCCCTCCCCCGTGCCTTCCTTGACCCTGGAAGGTGCCACTCCCACTGTCCTTT
CCTAATAAAATGAGGAAATTGCATCGCATTGTCTGAGTAGGTGTCATTCTATTCTGGGGGGTGGGGT
GGGGCAGGACAGCAAGGGGGAGGATTGGGAAGACAATAGCAGGCATGCTGGGGATGCGGTGGGC
TCTATGGCTCGAGAAAAAAAGCACCGACTCGGTGCCACTTTTTCAAGTTGATAACGGACTAGCCTTAT
TTGAACTTGCTATGCTGTTTCCAGCATAGCTCTCAAACAATCGATGCAGAGGATGGTGCAAACAAGG
CTTTTCTCCAAGGGATATTTATAGTCTCAAAACACACAATTACTTTACAGTTAGGGTGAGTTTCCTTTT
GTGCTGTTTTTTAAAATAATAATTTAGTATTTGTATCTCTTATAGAAATCCAAGCCTATCATGTAAAATG
TAGCTAGTATTAAAAAGAACAGATTATCTGTCTTTTATCGCACATTAAGCCTCTATAGTTACTAGGAAA
TATTATATGCAAATTAACCGGGGCAGGGGAGTAGCCGAGCTTCTCCCACAAGTCTGTGCGAGGGGG
CCGGCGCGGGCCTAGAGATGGCGGCGTCGGATCGCTAGCTCAAAAAAATTCTAGTTGGTTTAACGC
GTAACTAGATAGAACCGCG**ATGTCGGTA***CCCAAATCGATGCAGAGGATGGT*GCACCGACTCGGTGC
CACTTTTTCAAGTTGATAACGGACTAGCCTTATTTGAACTTGCTATGCTGTTTCCAGCATAGCTCTCAA
ACAGGTACCGACATGGTAAGCCGGTGTTTCGTCCTTTCCACAAGATATATAAAGCCAAGAAATCGAA
ATACTTTCAAGTTACGGTAAGCATATGATAGTCCATTTTAAAACATAATTTTAAAACTGCAAACTACCC
AAGAAATTATTACTTTCTACGTCACGTATTTTGTACTAATATCTTTGTGTTTACAGTCAAATTAATTCTA
ATTATCTCTCTAACAGCCTTGTATCGTATATGCAAATATGAAGGAATCATGGGAAATAGGCCCTCTTC
CTGCCCGACCTTGCGGCCGCAGGAACCCCTAGTGATGGAGTTGGCCACTCCCTCTCTGCGCGCTC
GCTCGCTCACTGAGGCCGGGCGACCAAAGGTCGCCCGACGCCCGGGCTTTGCCCGGGCGGCCTC
AGTGAGCGAGCGAGCGCGCAG

Start	End	Feature Description
1	130	5' inverted terminal repeat (ITR), pSub201(+)
153	407	EFS promoter for transcription initiation
421	477	SV40 nuclear localization sequence (NLS)
478	582	NpuC, C-terminal fragment of trans splicing intein
583	1614	Sp Cas9 N-terminal fragment, PEmax architecture
1615	1716	(SGGS)2 – SV40 NLS – SGGS2 optimized flexible linker
1717	3231	PE6c reverse transcriptase domain (evolved from Tf1)
3232	3300	Linker and SV40 nuclear localization sequence
3306	3466	WHV Posttranscriptional regulatory element (WPRE), gamma
3555	3765	BGH polyA signal sequence
3789	3795	RNA polymerase III terminator (reverse complement)
3796	3881	Sp Cas9 sgRNA scaffold (reverse complement)
3882	3902	m.*Atp1a3* D801N ngRNA with leading "g" (reverse complement)
3903	4217	U6 promoter from M. musculus (reverse complement)
4226	4232	RNA polymerase III terminator (reverse complement)
4233	4269	trimmed evo-preQ1 motif (reverse complement)
4270	4278	**Primer binding site (PBS) (reverse complement)**
4279	4301	*Reverse transcription template (RTT) (reverse complement)*

4302	4387	Sp Cas9 sgRNA scaffold (reverse complement)
4388	4407	m.*Atp1a3* D801N pegRNA spacer (reverse complement)
4408	4671	U6 promoter from H. sapiens (reverse complement)
4680	4809	3' inverted terminal repeat (ITR), pSub201(+)

Amino acid sequence
MKRTADGSEFESPKKKRKVIKIATRKYLGKQNVYDIGVERDHNFALKNGFIASNCFNEIGKATAKYFFYSNI
MNFFKTEITLANGEIRKRPLIETNGETGEIVWDKGRDFATVRKVLSMPQVNIVKKTEVQTGGFSKESILPKR
NSDKLIARKKDWDPKKYGGFDSPTVAYSVLVVAKVEKGKSKKLKSVKELLGITIMERSSFEKNPIDFLEAK
GYKEVKKDLIIKLPKYSLFELENGRKRMLASAGELQKGNELALPSKYVNFLYLASHYEKLKGSPEDNEQKQ
LFVEQHKHYLDEIIEQISEFSKRVILADANLDKVLSAYNKHRDKPIREQAENIIHLFTLTNLGAPAAFKYFDTT
IDRKRYTSTKEVLDATLIHQSITGLYETRIDLSQLGGDSGGSSGGSKRTADGSEFESPKKKRKVSGGSSG
GSISSSKHTLSQMNKVSNIVKEPELPDIYKEFKDITADTNTEKLPKPIKGLEFEVELTQENYRLPIRNYPLTP
VKMQAMNDEINQGLKGGIIRESKAINACPVIFVPRKEGTLRMVVDYRPLNKYVKPNVYPLPLIEQLLAKIQG
STIFTKLDLKSAYHQIRVRKGDEHKLAFRCPRGVFEYLVMPYGIKTAPAHFQYFINTILGEAKESHVVCYMD
DILIHSKSESEHVKHVKDVLQKLKNANLIINQAKCEFHQSQVKFLGYHISEKGLTPCQENIDKVLQWKQPK
NQKELRQFLGQVNYLRKFIPKTSQLTHPLNKLLKKDVRWKWTPTQTQAIENIKQCLVSPPVLRHFDFSKKI
LLETDVSDVAVGAVLSQKHDDDKYYPVGYYSAKMSKAQLNYSVSDKEMLAIIKSLEHWRHYLESTIEPFKI
LTDHRNLIGRITNESEPENKRLARWQLFLQDFNFEINYRPGSANHIADALSRIVDETEPIPKDNEDNSINFV
NQISISGGSKRTADGSEFESPKKKRKV

Start	End	Feature Description
1	19	SV40 nuclear localization sequence
20	54	NpuC, C-terminal fragment of trans splicing intein
55	398	Sp Cas9 C-terminal fragment, PEmax architecture
399	432	(SGGS)2 – SV40 NLS – (SGGS)2 optimized flexible linker
433	937	PE6c reverse transcriptase domain, evolved from Tf1
938	959	Flexible linker and SV40 NLS

pFB-PhSyn-hsaATP1A3nonopt-rBGpA
[transfer plasmid for gene therapy study, referred to as "AAV9-*ATP1A3*"]

DNA sequence

CCTGCAGGCAGCTGCGCGCTCGCTCGCTCACTGAGGCCGCCCGGGCAAAGCCCGGGCGTCGGGC
GACCTTTGGTCGCCCGGCCTCAGTGAGCGAGCGAGCGCGCAGAGAGGGAGTGGCCAACTCCATCA
CTAGGGGTTCCTGCGGCCGCACGCGTATACGCCGGCGATTGGTTACTAGTTTAATTAAACTAGACAGA
CTGCAGAGGGCCCTGCGTATGAGTGCAAGTGGGTTTTAGGACCAGGATGAGGCGGGGTGGGGGTG
CCTACCTGACGACCGACCCCGACCCACTGGACAAGCACCCAACCCCCATTCCCCAAATTGCGCATCC
CCTATCAGAGAGGGGGAGGGGAAACAGGATGCGGCGAGGCGCGTCGCGACTGCCAGCTTCAGCAC
CGCGGACAGTGCCTTCGCCCCCGCCTGGCGGCGCGCGCCACCGCCGCCTCAGCACTGAAGGCGC
GCTGACGTCACTCGCCGGTCCCCCGCAAACTCCCCTTCCCGGCCACCTTGGTCGCGTCCGCGCCG
CCGCCGGCCCAGCCGGACCGCACCACGCGAGGCGCGAGATAGGGGGGCACGGGCGCGACCATCT
GCGCTGCGGCGCCGGCGACTCAGCGCTGCCTCAGTCTGCGGTGGGCAGCGGAGGAGTCGTGTCG
TGCCTGAGAGCGCAGTCGAGAGACGCCGGACGCGCGCGCTGTGATCGTCACTTGGTAAGTCACTGA
CTGTCTATGCCTCGTCGACGGCCACCATGGGGGACAAGAAAGATGACAAGGACTCACCCAAGAAGA
ACAAGGGCAAGGAGCGCCGGGACCTGGATGACCTCAAGAAGGAGGTGGCTATGACAGAGCACAAG
ATGTCAGTGGAAGAGGTCTGCCGGAAATACAACACAGACTGTGTGCAGGGTTTGACCCACAGCAAAG
CCCAGGAGATCCTGGCCCGGGATGGGCCTAACGCACTCACGCCACCGCCTACCACCCCAGAGTGG
GTCAAGTTTTGCCGGCAGCTCTTCGGGGGCTTCTCCATCCTGCTGTGGATCGGGGCTATCCTCTGCT
TCCTGGCCTACGGTATCCAGGCGGGCACCGAGGACGACCCCTCTGGTGACAACCTGTACCTGGGCA
TCGTGCTGGCGGCCGTGGTGATCATCACTGGCTGCTTCTCCTACTACCAGGAGGCCAAGAGCTCCA
AGATCATGGAGTCCTTCAAGAACATGGTGCCCCAGCAAGCCCTGGTGATCCGGGAAGGTGAGAAGAT
GCAGGTGAACGCTGAGGAGGTGGTGGTCGGGGACCTGGTGGAGATCAAGGGTGGAGACCGAGTG
CCAGCTGACCTGCGGATCATCTCAGCCCACGGCTGCAAGGTGGACAACTCCTCCCTGACTGGCGAA
TCCGAGCCCCAGACTCGCTCTCCCGACTGCACTCACGACAACCCCTTGGAGACTCGGAACATCACC
TTCTTTTCCACCAACTGTGTGGAAGGCACGGCTCGGGGCGTGGTGGTGGCCACGGGCGACCGCAC
TGTCATGGGCCGTATCGCCACCCTGGCATCAGGGCTGGAGGTGGGCAAGACGCCCATCGCCATCGA
GATTGAGCACTTCATCCAGCTCATCACCGGCGTGGCTGTCTTCCTGGGTGTCTCCTTCTTCATCCTCT
CCCTCATTCTCGGATACACCTGGCTTGAGGCTGTCATCTTCCTCATCGGCATCATCGTGGCCAATGTC
CCAGAGGGTCTGCTGGCCACTGTCACTGTGTGTCTGACGCTGACCGCCAAGCGCATGGCCCGGAA
GAACTGCCTGGTGAAGAACCTGGAGGCTGTAGAAACCCTGGGCTCCACGTCCACCATCTGCTCAGA
TAAGACAGGGACCCTCACTCAGAACCGCATGACAGTCGCCCACATGTGGTTTGACAACCAGATCCAC
GAGGCTGACACCACTGAGGACCAGTCAGGGACCTCATTTGACAAGAGTTCGCACACCTGGGTGGCC
CTGTCTCACATCGCTGGGCTCTGCAATCGCGCTGTCTTCAAGGGTGGTCAGGACAACATCCCTGTGC
TCAAGAGGGATGTGGCTGGGGATGCGTCTGAGTCTGCCCTGCTCAAGTGCATCGAGCTGTCCTCTG
GCTCCGTGAAGCTGATGCGTGAACGCAACAAGAAAGTGGCTGAGATTCCCTTCAATTCCACCAACAA
ATACCAGCTCTCCATCCATGAGACCGAGGACCCCAACGACAACCGATACCTGCTGGTGATGAAGGGT
GCCCCCGAGCGCATCCTGGACCGCTGCTCCACCATCCTGCTACAGGGCAAGGAGCAGCCTCTGGAC
GAGGAAATGAAGGAGGCCTTCCAGAATGCCTACCTTGAGCTCGGTGGCCTGGGCGAGCGCGTGCTT
GGTTTCTGCCATTATTACCTGCCCGAGGAGCAGTTCCCCAAGGGCTTTGCCTTCGACTGTGATGACG
TGAACTTCACCACGGACAACCTCTGCTTTGTGGGCCTCATGTCCATGATCGACCCACCCCGGGCAGC
CGTCCCTGACGCGGTGGGCAAGTGTCGCAGCGCAGGCATCAAGGTCATCATGGTCACCGGCGATCA
CCCCATCACGGCCAAGGCCATTGCCAAGGGTGTGGGCATCATCTCTGAGGGCAACGAGACTGTGGA
GGACATCGCCGCCCGGCTCAACATTCCCGTCAGCCAGGTTAACCCCCGGGATGCCAAGGCCTGCGT
GATCCACGGCACCGACCTCAAGGACTTCACCTCCGAGCAAATCGACGAGATCCTGCAGAATCACACC
GAGATCGTCTTCGCCCGCACATCCCCCCAGCAGAAGCTCATCATTGTGGAGGGCTGTCAGAGACAG
GGTGCAATTGTGGCTGTGACCGGGGATGGTGTGAACGACTCCCCCGCTCTGAAGAAGGCCGACATT
GGGGTGGCCATGGGCATCGCTGGCTCTGACGTCTCCAAGCAGGCAGCTGACATGATCCTGCTGGAC
GACAACTTTGCCTCCATCGTCACAGGGGTGGAGGAGGGCCGCCTGATCTTCGACAACCTAAAGAAG
TCCATTGCCTACACCCTGACCAGCAATATCCCGGAGATCACGCCCTTCCTGCTGTTCATCATGGCCAA
CATCCCGCTGCCCCTGGGCACCATCACCATCCTCTGCATCGATCTGGGCACTGACATGGTCCCTGCC
ATCTCACTGGCGTACGAGGCTGCCGAAAGCGACATCATGAAGAGACAGCCCAGGAACCCGCGGACG
GACAAATTGGTCAATGAGAGACTCATCAGCATGGCCTACGGGCAGATTGGAATGATCCAGGCTCTCG
GTGGCTTCTTCTCTTACTTTGTGATCCTGGCAGAAAATGGCTTCTTGCCCGGCAACCTGGTGGGCAT
CCGGCTGAACTGGGATGACCGCACCGTCAATGACCTGGAAGACAGTTACGGGCAGCAGTGGACATA

CGAGCAGAGGAAGGTGGTGGAGTTCACCTGCCACACGGCCTTCTTTGTGAGCATCGTTGTCGTCCA
GTGGGCCGATCTGATCATCTGCAAGACCCGGAGGAACTCGGTCTTCCAGCAGGGCATGAAGAACAA
GATCCTGATCTTCGGGCTGTTTGAGGAGACGGCCCTGGCTGCCTTCCTGTCCTACTGCCCCGGCAT
GGACGTGGCCCTGCGCATGTACCCTCTCAAGCCCAGCTGGTGGTTCTGTGCCTTCCCCTACAGTTTC
CTCATCTTCGTCTACGACGAAATCCGCAAACTCATCCTGCGCAGGAACCCAGGGGGTTGGGTGGAG
AAGGAAACCTACTACTGATAAGCGATCGCTAGCAATGGCATCCTCGAGGGATCTTTTTCCCTCTGCCA
AAAATTATGGGGACATCATGAAGCCCCTTGAGCATCTGACTTCTGGCTAATAAAGGAAATTTATTTTCAT
TGCAATAGTGTGTTGGAATTTTTTGTGTCTCTCACTCGGGCGCGCCTTTCCGGACACGTGCGGACCG
AGCGGCCGCAGGAACCCCTAGTGATGGAGTTGGCCACTCCCTCTCTGCGCGCTCGCTCGCTCACTG
AGGCCGGGCGACCAAAGGTCGCCCGACGCCCGGGCTTTGCCCGGGCGGCCTCAGTGAGCGAGCG
AGCGCGCAGCTGCCTGCAGG

Start	End	Feature Description
1	141	AAV2/9 ITR
142	197	linker
198	668	hSyn promoter
669	737	5’ UTR
738	3785	Human *ATP1A3* gene cDNA sequence
3786	3816	3’ UTR
3817	3943	rBG polyadenylation signal
3944	3981	linker
3982	4122	AAV2/9 ITR

Amino acid sequence

ATMGDKKDDKDSPKKNKGKERRDLDDLKKEVAMTEHKMSVEEVCRKYNTDCVQGLTHSKAQEILARDG
PNALTPPPTTPEWVKFCRQLFGGFSILLWIGAILCFLAYGIQAGTEDDPSGDNLYLGIVLAAVVIITGCFSYY
QEAKSSKIMESFKNMVPQQALVIREGEKMQVNAEEVVVGDLVEIKGGDRVPADLRIISAHGCKVDNSSLT
GESEPQTRSPDCTHDNPLETRNITFFSTNCVEGTARGVVVATGDRTVMGRIATLASGLEVGKTPIAIEIEHF
IQLITGVAVFLGVSFFILSLILGYTWLEAVIFLIGIIVANVPEGLLATVTVCLTLTAKRMARKNCLVKNLEAVETL
GSTSTICSDKTGTLTQNRMTVAHMWFDNQIHEADTTEDQSGTSFDKSSHTWVALSHIAGLCNRAVFKGG
QDNIPVLKRDVAGDASESALLKCIELSSGSVKLMRERNKKVAEIPFNSTNKYQLSIHETEDPNDNRYLLVM
KGAPERILDRCSTILLQGKEQPLDEEMKEAFQNAYLELGGLGERVLGFCHYYLPEEQFPKGFAFDCDDVN
FTTDNLCFVGLMSMIDPPRAAVPDAVGKCRSAGIKVIMVTGDHPITAKAIAKGVGIISEGNETVEDIAARLNI
PVSQVNPRDAKACVIHGTDLKDFTSEQIDEILQNHTEIVFARTSPQQKLIIVEGCQRQGAIVAVTGDGVNDS
PALKKADIGVAMGIAGSDVSKQAADMILLDDNFASIVTGVEEGRLIFDNLKKSIAYTLTSNIPEITPFLLFIMAN
IPLPLGTITILCIDLGTDMVPAISLAYEAAESDIMKRQPRNPRTDKLVNERLISMAYGQIGMIQALGGFFSYFV
ILAENGFLPGNLVGIRLNWDDRTVNDLEDSYGQQWTYEQRKVVEFTCHTAFFVSIVVVQWADLIICKTRRN
SVFQQGMKNKILIFGLFEETALAAFLSYCPGMDVALRMYPLKPSWWFCAFPYSFLIFVYDEIRKLILRRNPG
GWVEKETYY

Start	End	Feature Description
1	1015	Human ATP1A3 protein sequence

ABE7.10 – Cas9 (D10A) – SpRY variant
Plasmid ID: pHS0369

DNA sequence

GACATTGATTATTGACTAGTTATTAATAGTAATCAATTACGGGGTCATTAGTTCATAGCCCATATATGGAG
TTCCGCGTTACATAACTTACGGTAAATGGCCCGCCTGGCTGACCGCCCAACGACCCCCGCCCATTGA
CGTCAATAATGACGTATGTTCCCATAGTAACGCCAATAGGGACTTTCCATTGACGTCAATGGGTGGAGT
ATTTACGGTAAACTGCCCACTTGGCAGTACATCAAGTGTATCATATGCCAAGTACGCCCCCTATTGACG
TCAATGACGGTAAATGGCCCGCCTGGCATTATGCCCAGTACATGACCTTATGGGACTTTCCTACTTGG
CAGTACATCTACGTATTAGTCATCGCTATTACCATGGTGATGCGGTTTTGGCAGTACATCAATGGGCGT
GGATAGCGGTTTGACTCACGGGGATTTCCAAGTCTCCACCCCATTGACGTCAATGGGAGTTTGTTTTG
GCACCAAAATCAACGGGACTTTCCAAAATGTCGTAACAACTCCGCCCCATTGACGCAAATGGGCGGT
AGGCGTGTACGGTGGGAGGTCTATATAAGCAGAGCTGGTTTAGTGAACCGTCAGATCTCGAGCTCGG
TACCTAATACGACACACTATAAGGAAATAAGAGAGAAAAGAAGAGTAAGAAGAAATATAAGAGCCACCA
TGAAACGGACAGCCGACGGAAGCGAGTTCGAGTCACCAAAGAAGAAGCGGAAAGTCTCTGAAGTCG
AGTTTAGCCACGAGTATTGGATGAGGCACGCACTGACCCTGGCAAAGCGAGCATGGGATGAAAGAGA
AGTCCCCGTGGGCGCCGTGCTGGTGCACAACAATAGAGTGATCGGAGAGGGATGGAACAGGCCAAT
CGGCCGCCACGACCCTACCGCACACGCAGAGATCATGGCACTGAGGCAGGGAGGCCTGGTCATGC
AGAATTACCGCCTGATCGATGCCACCCTGTATGTGACACTGGAGCCATGCGTGATGTGCGCAGGAGC
AATGATCCACAGCAGGATCGGAAGAGTGGTGTTCGGAGCACGGGACGCCAAGACCGGCGCAGCAG
GCTCCCTGATGGATGTGCTGCACCACCCCGGCATGAACCACCGGGTGGAGATCACAGAGGGAATCC
TGGCAGACGAGTGCGCCGCCCTGCTGAGCGATTTCTTTAGAATGCGGAGACAGGAGATCAAGGCCC
AGAAGAAGGCACAGAGCTCCACCGACTCTGGAGGATCTAGCGGAGGATCCTCTGGAAGCGAGACAC
CAGGCACAAGCGAGTCCGCCACACCAGAGAGCTCCGGCGGCTCCTCCGGAGGATCCTCTGAGGTG
GAGTTTTCCCACGAGTACTGGATGAGACATGCCCTGACCCTGGCCAAGAGGGCACGCGATGAGAGG
GAGGTGCCTGTGGGAGCCGTGCTGGTGCTGAACAATAGAGTGATCGGCGAGGGCTGGAACAGAGC
CATCGGCCTGCACGACCCAACAGCCCATGCCGAAATTATGGCCCTGAGACAGGGCGGCCTGGTCAT
GCAGAACTACAGACTGATTGACGCCACCCTGTACGTGACATTCGAGCCTTGCGTGATGTGCGCCGGC
GCCATGATCCACTCTAGGATCGGCCGCGTGGTGTTTGGCGTGAGGAACGCAAAAACCGGCGCCGCA
GGCTCCCTGATGGACGTGCTGCACTACCCCGGCATGAATCACCGCGTCGAAATTACCGAGGGAATCC
TGGCAGATGAATGTGCCGCCCTGCTGTGCTATTTCTTTCGGATGCCTAGACAGGTGTTCAATGCTCAG
AAGAAGGCCCAGAGCTCCACCGACTCCGGAGGATCTAGCGGAGGCTCCTCTGGCTCTGAGACACCT
GGCACAAGCGAGAGCGCAACACCTGAAAGCAGCGGGGGCAGCAGCGGGGGGTCAGACAAGAAGT
ACAGCATCGGCCTGGCCATCGGCACCAACTCTGTGGGCTGGGCCGTGATCACCGACGAGTACAAGG
TGCCCAGCAAGAAATTCAAGGTGCTGGGCAACACCGACCGGCACAGCATCAAGAAGAACCTGATCG
GAGCCCTGCTGTTCGACAGCGGCGAAACAGCCGAGAGAACCCGGCTGAAGAGAACCGCCAGAAGA
AGATACACCAGACGGAAGAACCGGATCTGCTATCTGCAAGAGATCTTCAGCAACGAGATGGCCAAGG
TGGACGACAGCTTCTTCCACAGACTGGAAGAGTCCTTCCTGGTGGAAGAGGATAAGAAGCACGAGC
GGCACCCCATCTTCGGCAACATCGTGGACGAGGTGGCCTACCACGAGAAGTACCCCACCATCTACCA
CCTGAGAAAGAAACTGGTGGACAGCACCGACAAGGCCGACCTGCGGCTGATCTATCTGGCCCTGGC
CCACATGATCAAGTTCCGGGGCCACTTCCTGATCGAGGGCGACCTGAACCCCGACAACAGCGACGT
GGACAAGCTGTTCATCCAGCTGGTGCAGACCTACAACCAGCTGTTCGAGGAAAACCCCATCAACGCC
AGCGGCGTGGACGCCAAGGCCATCCTGTCTGCCAGACTGAGCAAGAGCAGACGGCTGGAAAATCTG
ATCGCCCAGCTGCCCGGCGAGAAGAAGAATGGCCTGTTCGGAAACCTGATTGCCCTGAGCCTGGGC
CTGACCCCCAACTTCAAGAGCAACTTCGACCTGGCCGAGGATGCCAAACTGCAGCTGAGCAAGGAC
ACCTACGACGACGACCTGGACAACCTGCTGGCCCAGATCGGCGACCAGTACGCCGACCTGTTTCTG
GCCGCCAAGAACCTGTCCGACGCCATCCTGCTGAGCGACATCCTGAGAGTGAACACCGAGATCACC
AAGGCCCCCCTGAGCGCCTCTATGATCAAGAGATACGACGAGCACCACCAGGACCTGACCCTGCTG
AAAGCTCTCGTGCGGCAGCAGCTGCCTGAGAAGTACAAAGAGATTTTCTTCGACCAGAGCAAGAACG
GCTACGCCGGCTACATTGACGGCGGAGCCAGCCAGGAAGAGTTCTACAAGTTCATCAAGCCCATCCT
GGAAAAGATGGACGGCACCGAGGAACTGCTCGTGAAGCTGAACAGAGAGGACCTGCTGCGGAAGC
AGCGGACCTTCGACAACGGCAGCATCCCCCACCAGATCCACCTGGGAGAGCTGCACGCCATTCTGC
GGCGGCAGGAAGATTTTTACCCATTCCTGAAGGACAACCGGGAAAAGATCGAGAAGATCCTGACCTT
CCGCATCCCCTACTACGTGGGCCCTCTGGCCAGGGGAAACAGCAGATTCGCCTGGATGACCAGAAA
GAGCGAGGAAACCATCACCCCCTGGAACTTCGAGGAAGTGGTGGACAAGGGCGCTTCCGCCCAGA
GCTTCATCGAGCGGATGACCAACTTCGATAAGAACCTGCCCAACGAGAAGGTGCTGCCCAAGCACA

GCCTGCTGTACGAGTACTTCACCGTGTATAACGAGCTGACCAAAGTGAAATACGTGACCGAGGGAAT
GAGAAAGCCCGCCTTCCTGAGCGGCGAGCAGAAAAAGGCCATCGTGGACCTGCTGTTCAAGACCAA
CCGGAAAGTGACCGTGAAGCAGCTGAAAGAGGACTACTTCAAGAAAATCGAGTGCTTCGACTCCGTG
GAAATCTCCGGCGTGGAAGATCGGTTCAACGCCTCCCTGGGCACATACCACGATCTGCTGAAAATTAT
CAAGGACAAGGACTTCCTGGACAATGAGGAAAACGAGGACATTCTGGAAGATATCGTGCTGACCCTG
ACACTGTTTGAGGACAGAGAGATGATCGAGGAACGGCTGAAAACCTATGCCCACCTGTTCGACGACA
AAGTGATGAAGCAGCTGAAGCGGCGGAGATACACCGGCTGGGGCAGGCTGAGCCGGAAGCTGATC
AACGGCATCCGGGACAAGCAGTCCGGCAAGACAATCCTGGATTTCCTGAAGTCCGACGGCTTCGCC
AACAGAAACTTCATGCAGCTGATCCACGACGACAGCCTGACCTTTAAAGAGGACATCCAGAAAGCCC
AGGTGTCCGGCCAGGGCGATAGCCTGCACGAGCACATTGCCAATCTGGCCGGCAGCCCCGCCATTA
AGAAGGGCATCCTGCAGACAGTGAAGGTGGTGGACGAGCTCGTGAAAGTGATGGGCCGGCACAAG
CCCGAGAACATCGTGATCGAAATGGCCAGAGAGAACCAGACCACCCAGAAGGGACAGAAGAACAGC
CGCGAGAGAATGAAGCGGATCGAAGAGGGCATCAAAGAGCTGGGCAGCCAGATCCTGAAAGAACAC
CCCGTGGAAAACACCCAGCTGCAGAACGAGAAGCTGTACCTGTACTACCTGCAGAATGGGCGGGATA
TGTACGTGGACCAGGAACTGGACATCAACCGGCTGTCCGACTACGATGTGGACCATATCGTGCCTCA
GAGCTTTCTGAAGGACGACTCCATCGACAACAAGGTGCTGACCAGAAGCGACAAGAACCGGGGCAA
GAGCGACAACGTGCCCTCCGAAGAGGTCGTGAAGAAGATGAAGAACTACTGGCGGCAGCTGCTGAA
CGCCAAGCTGATTACCCAGAGAAAGTTCGACAATCTGACCAAGGCCGAGAGAGGCGGCCTGAGCGA
ACTGGATAAGGCCGGCTTCATCAAGAGACAGCTGGTGGAAACCCGGCAGATCACAAAGCACGTGGC
ACAGATCCTGGACTCCCGGATGAACACTAAGTACGACGAGAATGACAAGCTGATCCGGGAAGTGAAA
GTGATCACCCTGAAGTCCAAGCTGGTGTCCGATTTCCGGAAGGATTTCCAGTTTTACAAAGTGCGCG
AGATCAACAACTACCACCACGCCCACGACGCCTACCTGAACGCCGTCGTGGGAACCGCCCTGATCA
AAAAGTACCCTAAGCTGGAAAGCGAGTTCGTGTACGGCGACTACAAGGTGTACGACGTGCGGAAGAT
GATCGCCAAGAGCGAGCAGGAAATCGGCAAGGCTACCGCCAAGTACTTCTTCTACAGCAACATCATG
AACTTTTTCAAGACCGAGATTACCCTGGCCAACGGCGAGATCCGGAAGCGGCCTCTGATCGAGACAA
ACGGCGAAACCGGGGAGATCGTGTGGGATAAGGGCCGGGATTTTGCCACCGTGCGGAAAGTGCTG
AGCATGCCCCAAGTGAATATCGTGAAAAAGACCGAGGTGCAGACAGGCGGCTTCAGCAAAGAGTCTA
TCAGACCCAAGAGGAACAGCGATAAGCTGATCGCCAGAAAGAAGGACTGGGACCCTAAGAAGTACG
GCGGCTTCCTGTGGCCCACCGTGGCCTATTCTGTGCTGGTGGTGGCCAAAGTGGAAAAGGGCAAGT
CCAAGAAACTGAAGAGTGTGAAAGAGCTGCTGGGGATCACCATCATGGAAAGAAGCAGCTTCGAGAA
GAATCCCATCGACTTTCTGGAAGCCAAGGGCTACAAAGAAGTGAAAAAGGACCTGATCATCAAGCTG
CCTAAGTACTCCCTGTTCGAGCTGGAAAACGGCCGGAAGAGAATGCTGGCCTCTGCCAAGCAGCTG
CAGAAGGGAAACGAACTGGCCCTGCCCTCCAAATATGTGAACTTCCTGTACCTGGCCAGCCACTATG
AGAAGCTGAAGGGCTCCCCCGAGGATAATGAGCAGAAACAGCTGTTTGTGGAACAGCACAAGCACTA
CCTGGACGAGATCATCGAGCAGATCAGCGAGTTCTCCAAGAGAGTGATCCTGGCCGACGCTAATCTG
GACAAAGTGCTGTCCGCCTACAACAAGCACCGGGATAAGCCCATCAGAGAGCAGGCCGAGAATATCA
TCCACCTGTTTACCCTGACCAGACTGGGAGCCCCTAGAGCCTTCAAGTACTTTGACACCACCATCGA
CCCCAAGCAGTACAGAAGCACCAAAGAGGTGCTGGACGCCACCCTGATCCACCAGAGCATCACCGG
CCTGTACGAGACACGGATCGACCTGTCTCAGCTGGGAGGTGACTCTGGCGGCTCAAAAAGAACCGC
CGACGGCAGCGAATTCGAGCCCAAGAAGAAGAGGAAAGTCTTAATTAAGCTGCCTTCTGCGGGGCTT
GCCTTCTGGCCATGCCCTTCTTCTCTCCCTTGCACCTGTACCTCTTGGTCTTTGAATAAAGCCTGAGT
AGGAAGCGACTGTGCCTTCTAGTTGCCAGCCATCTGTTGTTTGCCCCTCCCCCGTGCCTTCCTTGAC
CCTGGAAGGTGCCACTCCCACTGTCCTTTCCTAATAAAATGAGAAATTGCATCGCATTGTCTGAGTA
GGTGTCATTCTATTCTGGGGGGTGGGGTGGGGCAGGACAGCAAGGGGGAGGATTGGGAAGACAATA
GCAGGCATGCTGGGGATGCGGTGGGCTCTATGG

Start	End	Feature Description
1	380	CMV enhancer
381	619	CMV promoter
620	636	T7 promoter with mismatch
637	683	5' UTR

684	740	BP NLS
741	1238	TadA wild type
1239	1334	(SGGS)2 – XTEN – (SGGS)2 linker
1335	1832	Engineered TadA* variant 7.10
1833	1928	(SGGS)2 – XTEN – (SGGS)2 linker
1929	6029	Cas9(D10A) SpRY PAM variant
6030	6095	Linker + SV40 BP NLS
6096	6196	3' UTR
6197	6424	bGH poly(A) signal

Amino acid sequence

MKRTADGSEFESPKKKRKVSEVEFSHEYWMRHALTLAKRAWDEREVPVGAVLVHNNRVIGEGWNRPIG
RHDPTAHAEIMALRQGGLVMQNYRLIDATLYVTLEPCVMCAGAMIHSRIGRVVFGARDAKTGAAGSLMDV
LHHPGMNHRVEITEGILADECAALLSDFFRMRRQEIKAQKKAQSSTDSGGSSGGSSGSETPGTSESATP
ESSGGSSGGSSEVEFSHEYWMRHALTLAKRARDEREVPVGAVLVLNNRVIGEGWNRAIGLHDPTAHAEI
MALRQGGLVMQNYRLIDATLYVTFEPCVMCAGAMIHSRIGRVVFGVRNAKTGAAGSLMDVLHYPGMNHR
VEITEGILADECAALLCYFFRMPRQVFNAQKKAQSSTDSGGSSGGSSGSETPGTSESATPESSGGSSGG
SDKKYSIGLAIGTNSVGWAVITDEYKVPSKKFKVLGNTDRHSIKKNLIGALLFDSGETAERTRLKRTARRRY
TRRKNRICYLQEIFSNEMAKVDDSFFHRLEESFLVEEDKKHERHPIFGNIVDEVAYHEKYPTIYHLRKKLVD
STDKADLRLIYLALAHMIKFRGHFLIEGDLNPDNSDVDKLFIQLVQTYNQLFEENPINASGVDAKAILSARLS
KSRRLENLIAQLPGEKKNGLFGNLIALSLGLTPNFKSNFDLAEDAKLQLSKDTYDDDLDNLLAQIGDQYADL
FLAAKNLSDAILLSDILRVNTEITKAPLSASMIKRYDEHHQDLTLLKALVRQQLPEKYKEIFFDQSKNGYAGYI
DGGASQEEFYKFIKPILEKMDGTEELLVKLNREDLLRKQRTFDNGSIPHQIHLGELHAILRRQEDFYPFLKD
NREKIEKILTFRIPYYVGPLARGNSRFAWMTRKSEETITPWNFEEVVDKGASAQSFIERMTNFDKNLPNEK
VLPKHSLLYEYFTVYNELTKVKYVTEGMRKPAFLSGEQKKAIVDLLFKTNRKVTVKQLKEDYFKKIECFDSV
EISGVEDRFNASLGTYHDLLKIIKDKDFLDNEENEDILEDIVLTLTLFEDREMIEERLKTYAHLFDDKVMKQLK
RRRYTGWGRLSRKLINGIRDKQSGKTILDFLKSDGFANRNFMQLIHDDSLTFKEDIQKAQVSGQGDSLHE
HIANLAGSPAIKKGILQTVKVVDELVKVMGRHKPENIVIEMARENQTTQKGQKNSRERMKRIEEGIKELGS
QILKEHPVENTQLQNEKLYLYYLQNGRDMYVDQELDINRLSDYDVDHIVPQSFLKDDSIDNKVLTRSDKNR
GKSDNVPSEEVVKKMKNYWRQLLNAKLITQRKFDNLTKAERGGLSELDKAGFIKRQLVETRQITKHVAQIL
DSRMNTKYDENDKLIREVKVITLKSKLVSDFRKDFQFYKVREINNYHHAHDAYLNAVVGTALIKKYPKLESE
FVYGDYKVYDVRKMIAKSEQEIGKATAKYFFYSNIMNFFKTEITLANGEIRKRPLIETNGETGEIVWDKGRD
FATVRKVLSMPQVNIVKKTEVQTGGFSKESIRPKRNSDKLIARKKDWDPKKYGGFLWPTVAYSVLVVAKVE
KGKSKKLKSVKELLGITIMERSSFEKNPIDFLEAKGYKEVKKDLIIKLPKYSLFELENGRKRMLASAKQLQK
GNELALPSKYVNFLYLASHYEKLKGSPEDNEQKQLFVEQHKHYLDEIIEQISEFSKRVILADANLDKVLSAY
NKHRDKPIREQAENIIHLFTLTRLGAPRAFKYFDTTIDPKQYRSTKEVLDATLIHQSITGLYETRIDLSQLGGD
SGGSKRTADGSEFEPKKKRKV

Start	End	Feature Description
1	19	BP NLS
20	185	TadA wild type
186	217	(SGGS)2 – XTEN – (SGGS)2 linker
218	383	Engineered TadA* variant 7.10
384	415	(SGGS)2 – XTEN – (SGGS)2 linker

416	1782	Cas9(D10A) SpRY PAM variant
1783	1803	Linker + SV40 BP NLS

ABE8e – Cas9 (D10A) – SpRY variant
Plasmid ID: pHS0363

<u>DNA sequence</u>
GACATTGATTATTGACTAGTTATTAATAGTAATCAATTACGGGGTCATTAGTTCATAGCCCATATATGGAG
TTCCGCGTTACATAACTTACGGTAAATGGCCCGCCTGGCTGACCGCCCAACGACCCCCGCCCATTGA
CGTCAATAATGACGTATGTTCCCATAGTAACGCCAATAGGGACTTTCCATTGACGTCAATGGGTGGAGT
ATTTACGGTAAACTGCCCACTTGGCAGTACATCAAGTGTATCATATGCCAAGTACGCCCCCTATTGACG
TCAATGACGGTAAATGGCCCGCCTGGCATTATGCCCAGTACATGACCTTATGGGACTTTCCTACTTGG
CAGTACATCTACGTATTAGTCATCGCTATTACCATGGTGATGCGGTTTTGGCAGTACATCAATGGGCGT
GGATAGCGGTTTGACTCACGGGGATTTCCAAGTCTCCACCCCATTGACGTCAATGGGAGTTTGTTTTG
GCACCAAAATCAACGGGACTTTCCAAAATGTCGTAACAACTCCGCCCCATTGACGCAAATGGGCGGT
AGGCGTGTACGGTGGGAGGTCTATATAAGCAGAGCTGGTTTAGTGAACCGTCAGATCTCGAGCTCGG
TACCTAATACGACACACTATAAGGAAATAAGAGAGAAAAGAAGAGTAAGAAGAAATATAAGAGCCACCA
TGAAACGGACAGCCGACGGAAGCGAGTTCGAGTCACCAAAGAAGAAGCGGAAAGTCTCTGAGGTG
GAGTTTTCCCACGAGTACTGGATGAGACATGCCCTGACCCTGGCCAAGAGGGCACGGGATGAGAGG
GAGGTGCCTGTGGGAGCCGTGCTGGTGCTGAACAATAGAGTGATCGGCGAGGGCTGGAACAGAGC
CATCGGCCTGCACGACCCAACAGCCCATGCCGAAATTATGGCCCTGAGACAGGGCGGCCTGGTCAT
GCAGAACTACAGACTGATTGACGCCACCCTGTACGTGACATTCGAGCCTTGCGTGATGTGCGCCGGC
GCCATGATCCACTCTAGGATCGGCCGCGTGGTGTTTGGCGTGAGGAACTCAAAAAGAGGCGCCGCA
GGCTCCCTGATGAACGTGCTGAACTACCCCGGCATGAATCACCGCGTCGAAATTACCGAGGGAATCC
TGGCAGATGAATGTGCCGCCCTGCTGTGCGATTTCTATCGGATGCCTAGACAGGTGTTCAATGCTCAG
AAGAAGGCCCAGAGCTCCATCAACTCCGGAGGATCTAGCGGAGGCTCCTCTGGCTCTGAGACACCT
GGCACAAGCGAGAGCGCAACACCTGAAAGCAGCGGGGGCAGCAGCGGGGGGTCAGACAAGAAGT
ACAGCATCGGCCTGGCCATCGGCACCAACTCTGTGGGCTGGGCCGTGATCACCGACGAGTACAAGG
TGCCCAGCAAGAAATTCAAGGTGCTGGGCAACACCGACCGGCACAGCATCAAGAAGAACCTGATCG
GAGCCCTGCTGTTCGACAGCGGCGAAACAGCCGAGAGAACCCGGCTGAAGAGAACCGCCAGAAGA
AGATACACCAGACGGAAGAACCGGATCTGCTATCTGCAAGAGATCTTCAGCAACGAGATGGCCAAGG
TGGACGACAGCTTCTTCCACAGACTGGAAGAGTCCTTCCTGGTGGAAGAGGATAAGAAGCACGAGC
GGCACCCCATCTTCGGCAACATCGTGGACGAGGTGGCCTACCACGAGAAGTACCCCACCATCTACCA
CCTGAGAAAGAAACTGGTGGACAGCACCGACAAGGCCGACCTGCGGCTGATCTATCTGGCCCTGGC
CCACATGATCAAGTTCCGGGGCCACTTCCTGATCGAGGGCGACCTGAACCCCGACAACAGCGACGT
GGACAAGCTGTTCATCCAGCTGGTGCAGACCTACAACCAGCTGTTCGAGGAAAACCCCATCAACGCC
AGCGGCGTGGACGCCAAGGCCATCCTGTCTGCCAGACTGAGCAAGAGCAGACGGCTGGAAAATCTG
ATCGCCCAGCTGCCCGGCGAGAAGAAGAATGGCCTGTTCGGAAACCTGATTGCCCTGAGCCTGGGC
CTGACCCCCAACTTCAAGAGCAACTTCGACCTGGCCGAGGATGCCAAACTGCAGCTGAGCAAGGAC
ACCTACGACGACGACCTGGACAACCTGCTGGCCCAGATCGGCGACCAGTACGCCGACCTGTTTCTG
GCCGCCAAGAACCTGTCCGACGCCATCCTGCTGAGCGACATCCTGAGAGTGAACACCGAGATCACC
AAGGCCCCCCTGAGCGCCTCTATGATCAAGAGATACGACGAGCACCACCAGGACCTGACCCTGCTG
AAAGCTCTCGTGCGGCAGCAGCTGCCTGAGAAGTACAAAGAGATTTTCTTCGACCAGAGCAAGAACG
GCTACGCCGGCTACATTGACGGCGGAGCCAGCCAGGAAGAGTTCTACAAGTTCATCAAGCCCATCCT
GGAAAAGATGGACGGCACCGAGGAACTGCTCGTGAAGCTGAACAGAGAGGACCTGCTGCGGAAGC
AGCGGACCTTCGACAACGGCAGCATCCCCCACCAGATCCACCTGGGAGAGCTGCACGCCATTCTGC
GGCGGCAGGAAGATTTTTACCCATTCCTGAAGGACAACCGGGAAAAGATCGAGAAGATCCTGACCTT
CCGCATCCCCTACTACGTGGGCCCTCTGGCCAGGGGAAACAGCAGATTCGCCTGGATGACCAGAAA
GAGCGAGGAAACCATCACCCCCTGGAACTTCGAGGAAGTGGTGGACAAGGGCGCTTCCGCCCAGA
GCTTCATCGAGCGGATGACCAACTTCGATAAGAACCTGCCCAACGAGAAGGTGCTGCCCAAGCACA
GCCTGCTGTACGAGTACTTCACCGTGTATAACGAGCTGACCAAAGTGAAATACGTGACCGAGGGAAT
GAGAAAGCCCGCCTTCCTGAGCGGCGAGCAGAAAAAGGCCATCGTGGACCTGCTGTTCAAGACCAA
CCGGAAAGTGACCGTGAAGCAGCTGAAAGAGGACTACTTCAAGAAAATCGAGTGCTTCGACTCCGTG
GAAATCTCCGGCGTGGAAGATCGGTTCAACGCCTCCCTGGGCACATACCACGATCTGCTGAAAATTAT
CAAGGACAAGGACTTCCTGGACAATGAGGAAAACGAGGACATTCTGGAAGATATCGTGCTGACCCTG
ACACTGTTTGAGGACAGAGAGATGATCGAGGAACGGCTGAAAACCTATGCCCACCTGTTCGACGACA
AAGTGATGAAGCAGCTGAAGCGGCGGAGATACACCGGCTGGGGCAGGCTGAGCCGGAAGCTGATC
AACGGCATCCGGGACAAGCAGTCCGGCAAGACAATCCTGGATTTCCTGAAGTCCGACGGCTTCGCC
AACAGAAACTTCATGCAGCTGATCCACGACGACAGCCTGACCTTTAAAGAGGACATCCAGAAAGCCC

AGGTGTCCGGCCAGGGCGATAGCCTGCACGAGCACATTGCCAATCTGGCCGGCAGCCCCGCCATTA
AGAAGGGCATCCTGCAGACAGTGAAGGTGGTGGACGAGCTCGTGAAAGTGATGGGCCGGCACAAG
CCCGAGAACATCGTGATCGAAATGGCCAGAGAGAACCAGACCACCCAGAAGGGACAGAAGAACAGC
CGCGAGAGAATGAAGCGGATCGAAGAGGGCATCAAAGAGCTGGGCAGCCAGATCCTGAAAGAACAC
CCCGTGGAAAACACCCAGCTGCAGAACGAGAAGCTGTACCTGTACTACCTGCAGAATGGGCGGGATA
TGTACGTGGACCAGGAACTGGACATCAACCGGCTGTCCGACTACGATGTGGACCATATCGTGCCTCA
GAGCTTTCTGAAGGACGACTCCATCGACAACAAGGTGCTGACCAGAAGCGACAAGAACCGGGGCAA
GAGCGACAACGTGCCCTCCGAAGAGGTCGTGAAGAAGATGAAGAACTACTGGCGGCAGCTGCTGAA
CGCCAAGCTGATTACCCAGAGAAAGTTCGACAATCTGACCAAGGCCGAGAGAGGCGGCCTGAGCGA
ACTGGATAAGGCCGGCTTCATCAAGAGACAGCTGGTGGAAACCCGGCAGATCACAAAGCACGTGGC
ACAGATCCTGGACTCCCGGATGAACACTAAGTACGACGAGAATGACAAGCTGATCCGGGAAGTGAAA
GTGATCACCCTGAAGTCCAAGCTGGTGTCCGATTTCCGGAAGGATTTCCAGTTTTACAAAGTGCGCG
AGATCAACAACTACCACCACGCCCACGACGCCTACCTGAACGCCGTCGTGGGAACCGCCCTGATCA
AAAAGTACCCTAAGCTGGAAAGCGAGTTCGTGTACGGCGACTACAAGGTGTACGACGTGCGGAAGAT
GATCGCCAAGAGCGAGCAGGAAATCGGCAAGGCTACCGCCAAGTACTTCTTCTACAGCAACATCATG
AACTTTTTCAAGACCGAGATTACCCTGGCCAACGGCGAGATCCGGAAGCGGCCTCTGATCGAGACAA
ACGGCGAAACCGGGGAGATCGTGTGGGATAAGGGCCGGGATTTTGCCACCGTGCGGAAAGTGCTG
AGCATGCCCCAAGTGAATATCGTGAAAAAGACCGAGGTGCAGACAGGCGGCTTCAGCAAAGAGTCTA
TCAGACCCAAGAGGAACAGCGATAAGCTGATCGCCAGAAAGAAGGACTGGGACCCTAAGAAGTACG
GCGGCTTCCTGTGGCCCACCGTGGCCTATTCTGTGCTGGTGGTGGCCAAAGTGGAAAAGGGCAAGT
CCAAGAAACTGAAGAGTGTGAAAGAGCTGCTGGGGATCACCATCATGGAAAGAAGCAGCTTCGAGAA
GAATCCCATCGACTTTCTGGAAGCCAAGGGCTACAAAGAAGTGAAAAAGGACCTGATCATCAAGCTG
CCTAAGTACTCCCTGTTCGAGCTGGAAAACGGCCGGAAGAGAATGCTGGCCTCTGCCAAGCAGCTG
CAGAAGGGAAACGAACTGGCCCTGCCCTCCAAATATGTGAACTTCCTGTACCTGGCCAGCCACTATG
AGAAGCTGAAGGGCTCCCCCGAGGATAATGAGCAGAAACAGCTGTTTGTGGAACAGCACAAGCACTA
CCTGGACGAGATCATCGAGCAGATCAGCGAGTTCTCCAAGAGAGTGATCCTGGCCGACGCTAATCTG
GACAAAGTGCTGTCCGCCTACAACAAGCACCGGGATAAGCCCATCAGAGAGCAGGCCGAGAATATCA
TCCACCTGTTTACCCTGACCAGACTGGGAGCCCCTAGAGCCTTCAAGTACTTTGACACCACCATCGA
CCCCAAGCAGTACAGAAGCACCAAAGAGGTGCTGGACGCCACCCTGATCCACCAGAGCATCACCGG
CCTGTACGAGACACGGATCGACCTGTCTCAGCTGGGAGGTGACTCTGGCGGCTCAAAAAGAACCGC
CGACGGCAGCGAATTCGAGCCCAAGAAGAAGAGGAAAGTCTAATTAATTAAGCTGCCTTCTGCGGGG
CTTGCCTTCTGGCCATGCCCTTCTTCTCTCCCTTGCACCTGTACCTCTTGGTCTTTGAATAAAGCCTG
AGTAGGAAGCGACTGTGCCTTCTAGTTGCCAGCCATCTGTTGTTTGCCCCTCCCCCGTGCCTTCCTT
GACCCTGGAAGGTGCCACTCCCACTGTCCTTTCCTAATAAAATGAGAAATTGCATCGCATTGTCTGA
GTAGGTGTCATTCTATTCTGGGGGGTGGGGTGGGGCAGGACAGCAAGGGGGAGGATTGGGAAGAC
AATAGCAGGCATGCTGGGGATGCGGTGGGCTCTATGG

Start	End	Feature Description
1	380	CMV enhancer
381	619	CMV promoter
620	636	T7 promoter with mismatch
637	683	5' UTR
684	740	BP NLS
741	1238	Engineered TadA* variant 8e
1239	1334	(SGGS)2 – XTEN – (SGGS)2 linker
1335	5435	Cas9(D10A) SpRY PAM variant
5436	5501	Linker + SV40 BP NLS

5502	5605	3’ UTR
5606	5830	bGH poly(A) signal

Amino acid sequence

MKRTADGSEFESPKKKRKVSEVEFSHEYWMRHALTLAKRARDEREVPVGAVLVLNNRVIGEGWNRAIGL
HDPTAHAEIMALRQGGLVMQNYRLIDATLYVTFEPCVMCAGAMIHSRIGRVVFGVRNSKRGAAGSLMNVL
NYPGMNHRVEITEGILADECAALLCDFYRMPRQVFNAQKKAQSSINSGGSSGGSSGSETPGTSESATPE
SSGGSSGGSDKKYSIGLAIGTNSVGWAVITDEYKVPSKKFKVLGNTDRHSIKKNLIGALLFDSGETAERTR
LKRTARRRYTRRKNRICYLQEIFSNEMAKVDDSFFHRLEESFLVEEDKKHERHPIFGNIVDEVAYHEKYPTI
YHLRKKLVDSTDKADLRLIYLALAHMIKFRGHFLIEGDLNPDNSDVDKLFIQLVQTYNQLFEENPINASGVD
AKAILSARLSKSRRLENLIAQLPGEKKNGLFGNLIALSLGLTPNFKSNFDLAEDAKLQLSKDTYDDDLDNLL
AQIGDQYADLFLAAKNLSDAILLSDILRVNTEITKAPLSASMIKRYDEHHQDLTLLKALVRQQLPEKYKEIFFD
QSKNGYAGYIDGGASQEEFYKFIKPILEKMDGTEELLVKLNREDLLRKQRTFDNGSIPHQIHLGELHAILRR
QEDFYPFLKDNREKIEKILTFRIPYYVGPLARGNSRFAWMTRKSEETITPWNFEEVVDKGASAQSFIERMT
NFDKNLPNEKVLPKHSLLYEYFTVYNELTKVKYVTEGMRKPAFLSGEQKKAIVDLLFKTNRKVTVKQLKED
YFKKIECFDSVEISGVEDRFNASLGTYHDLLKIIKDKDFLDNEENEDILEDIVLTLTLFEDREMIEERLKTYAH
LFDDKVMKQLKRRRYTGWGRLSRKLINGIRDKQSGKTILDFLKSDGFANRNFMQLIHDDSLTFKEDIQKAQ
VSGQGDSLHEHIANLAGSPAIKKGILQTVKVVDELVKVMGRHKPENIVIEMARENQTTQKGQKNSRERMK
RIEEGIKELGSQILKEHPVENTQLQNEKLYLYYLQNGRDMYVDQELDINRLSDYDVDHIVPQSFLKDDSIDN
KVLTRSDKNRGKSDNVPSEEVVKKMKNYWRQLLNAKLITQRKFDNLTKAERGGLSELDKAGFIKRQLVET
RQITKHVAQILDSRMNTKYDENDKLIREVKVITLKSKLVSDFRKDFQFYKVREINNYHHAHDAYLNAVVGTA
LIKKYPKLESEFVYGDYKVYDVRKMIAKSEQEIGKATAKYFFYSNIMNFFKTEITLANGEIRKRPLIETNGET
GEIVWDKGRDFATVRKVLSMPQVNIVKKTEVQTGGFSKESIRPKRNSDKLIARKKDWDPKKYGGFLWPTV
AYSVLVVAKVEKGKSKKLKSVKELLGITIMERSSFEKNPIDFLEAKGYKEVKKDLIIKLPKYSLFELENGRKR
MLASAKQLQKGNELALPSKYVNFLYLASHYEKLKGSPEDNEQKQLFVEQHKHYLDEIIEQISEFSKRVILAD
ANLDKVLSAYNKHRDKPIREQAENIIHLFTLTRLGAPRAFKYFDTTIDPKQYRSTKEVLDATLIHQSITGLYET
RIDLSQLGGDSGGSKRTADGSEFEPKKKRKV

Start	End	Feature Description
1	19	BP NLS
20	185	Engineered TadA* variant 8e
186	217	(SGGS)2 – XTEN – (SGGS)2 linker
218	1584	Cas9(D10A) SpRY PAM variant
1585	1605	Linker + SV40 BP NLS

ABE8e(V106W) – Cas9 (D10A) – SpRY variant
Plasmid ID: pHS0364

DNA sequence

GACATTGATTATTGACTAGTTATTAATAGTAATCAATTACGGGGTCATTAGTTCATAGCCCATATATGGAG
TTCCGCGTTACATAACTTACGGTAAATGGCCCGCCTGGCTGACCGCCCAACGACCCCCGCCCATTGA
CGTCAATAATGACGTATGTTCCCATAGTAACGCCAATAGGGACTTTCCATTGACGTCAATGGGTGGAGT
ATTTACGGTAAACTGCCCACTTGGCAGTACATCAAGTGTATCATATGCCAAGTACGCCCCCTATTGACG
TCAATGACGGTAAATGGCCCGCCTGGCATTATGCCCAGTACATGACCTTATGGGACTTTCCTACTTGG
CAGTACATCTACGTATTAGTCATCGCTATTACCATGGTGATGCGGTTTTGGCAGTACATCAATGGGCGT
GGATAGCGGTTTGACTCACGGGGATTTCCAAGTCTCCACCCCATTGACGTCAATGGGAGTTTGTTTTG
GCACCAAAATCAACGGGACTTTCCAAAATGTCGTAACAACTCCGCCCCATTGACGCAAATGGGCGGT
AGGCGTGTACGGTGGGAGGTCTATATAAGCAGAGCTGGTTTAGTGAACCGTCAGATCTCGAGCTCGG
TACCTAATACGACACACTATAAGGAAATAAGAGAGAAAAGAAGAGTAAGAAGAAATATAAGAGCCACCA
TGAAACGGACAGCCGACGGAAGCGAGTTCGAGTCACCAAAGAAGAAGCGGAAAGTCTCTGAGGTG
GAGTTTTCCCACGAGTACTGGATGAGACATGCCCTGACCCTGGCCAAGAGGGCACGGGATGAGAGG
GAGGTGCCTGTGGGAGCCGTGCTGGTGCTGAACAATAGAGTGATCGGCGAGGGCTGGAACAGAGC
CATCGGCCTGCACGACCCAACAGCCCATGCCGAAATTATGGCCCTGAGACAGGGCGGCCTGGTCAT
GCAGAACTACAGACTGATTGACGCCACCCTGTACGTGACATTCGAGCCTTGCGTGATGTGCGCCGGC
GCCATGATCCACTCTAGGATCGGCCGCGTGGTGTTTGGATGGAGAAATTCTAAAAGAGGCGCCGCAG
GCTCCCTGATGAACGTGCTGAACTACCCCGGCATGAATCACCGCGTCGAAATTACCGAGGGAATCCT
GGCAGATGAATGTGCCGCCCTGCTGTGCGATTTCTATCGGATGCCTAGACAGGTGTTCAATGCTCAG
AAGAAGGCCCAGAGCTCCATCAACTCCGGAGGATCTAGCGGAGGCTCCTCTGGCTCTGAGACACCT
GGCACAAGCGAGAGCGCAACACCTGAAAGCAGCGGGGGCAGCAGCGGGGGGTCAGACAAGAAGT
ACAGCATCGGCCTGGCCATCGGCACCAACTCTGTGGGCTGGGCCGTGATCACCGACGAGTACAAGG
TGCCCAGCAAGAAATTCAAGGTGCTGGGCAACACCGACCGGCACAGCATCAAGAAGAACCTGATCG
GAGCCCTGCTGTTCGACAGCGGCGAAACAGCCGAGAGAACCCGGCTGAAGAGAACCGCCAGAAGA
AGATACACCAGACGGAAGAACCGGATCTGCTATCTGCAAGAGATCTTCAGCAACGAGATGGCCAAGG
TGGACGACAGCTTCTTCCACAGACTGGAAGAGTCCTTCCTGGTGGAAGAGGATAAGAAGCACGAGC
GGCACCCCATCTTCGGCAACATCGTGGACGAGGTGGCCTACCACGAGAAGTACCCCACCATCTACCA
CCTGAGAAAGAAACTGGTGGACAGCACCGACAAGGCCGACCTGCGGCTGATCTATCTGGCCCTGGC
CCACATGATCAAGTTCCGGGGCCACTTCCTGATCGAGGGCGACCTGAACCCCGACAACAGCGACGT
GGACAAGCTGTTCATCCAGCTGGTGCAGACCTACAACCAGCTGTTCGAGGAAAACCCCATCAACGCC
AGCGGCGTGGACGCCAAGGCCATCCTGTCTGCCAGACTGAGCAAGAGCAGACGGCTGGAAAATCTG
ATCGCCCAGCTGCCCGGCGAGAAGAAGAATGGCCTGTTCGGAAACCTGATTGCCCTGAGCCTGGGC
CTGACCCCCAACTTCAAGAGCAACTTCGACCTGGCCGAGGATGCCAAACTGCAGCTGAGCAAGGAC
ACCTACGACGACGACCTGGACAACCTGCTGGCCCAGATCGGCGACCAGTACGCCGACCTGTTTCTG
GCCGCCAAGAACCTGTCCGACGCCATCCTGCTGAGCGACATCCTGAGAGTGAACACCGAGATCACC
AAGGCCCCCCTGAGCGCCTCTATGATCAAGAGATACGACGAGCACCACCAGGACCTGACCCTGCTG
AAAGCTCTCGTGCGGCAGCAGCTGCCTGAGAAGTACAAAGAGATTTTCTTCGACCAGAGCAAGAACG
GCTACGCCGGCTACATTGACGGCGGAGCCAGCCAGGAAGAGTTCTACAAGTTCATCAAGCCCATCCT
GGAAAAGATGGACGGCACCGAGGAACTGCTCGTGAAGCTGAACAGAGAGGACCTGCTGCGGAAGC
AGCGGACCTTCGACAACGGCAGCATCCCCCACCAGATCCACCTGGGAGAGCTGCACGCCATTCTGC
GGCGGCAGGAAGATTTTTACCCATTCCTGAAGGACAACCGGGAAAAGATCGAGAAGATCCTGACCTT
CCGCATCCCCTACTACGTGGGCCCTCTGGCCAGGGGAAACAGCAGATTCGCCTGGATGACCAGAAA
GAGCGAGGAAACCATCACCCCCTGGAACTTCGAGGAAGTGGTGGACAAGGGCGCTTCCGCCCAGA
GCTTCATCGAGCGGATGACCAACTTCGATAAGAACCTGCCCAACGAGAAGGTGCTGCCCAAGCACA
GCCTGCTGTACGAGTACTTCACCGTGTATAACGAGCTGACCAAAGTGAAATACGTGACCGAGGGAAT
GAGAAAGCCCGCCTTCCTGAGCGGCGAGCAGAAAAAGGCCATCGTGGACCTGCTGTTCAAGACCAA
CCGGAAAGTGACCGTGAAGCAGCTGAAAGAGGACTACTTCAAGAAAATCGAGTGCTTCGACTCCGTG
GAAATCTCCGGCGTGGAAGATCGGTTCAACGCCTCCCTGGGCACATACCACGATCTGCTGAAAATTAT
CAAGGACAAGGACTTCCTGGACAATGAGGAAAACGAGGACATTCTGGAAGATATCGTGCTGACCCTG
ACACTGTTTGAGGACAGAGAGATGATCGAGGAACGGCTGAAAACCTATGCCCACCTGTTCGACGACA
AAGTGATGAAGCAGCTGAAGCGGCGGAGATACACCGGCTGGGGCAGGCTGAGCCGGAAGCTGATC
AACGGCATCCGGGACAAGCAGTCCGGCAAGACAATCCTGGATTTCCTGAAGTCCGACGGCTTCGCC
AACAGAAACTTCATGCAGCTGATCCACGACGACAGCCTGACCTTTAAAGAGGACATCCAGAAAGCCC

AGGTGTCCGGCCAGGGCGATAGCCTGCACGAGCACATTGCCAATCTGGCCGGCAGCCCCGCCATTA
AGAAGGGCATCCTGCAGACAGTGAAGGTGGTGGACGAGCTCGTGAAAGTGATGGGCCGGCACAAG
CCCGAGAACATCGTGATCGAAATGGCCAGAGAGAACCAGACCACCCAGAAGGGACAGAAGAACAGC
CGCGAGAGAATGAAGCGGATCGAAGAGGGCATCAAAGAGCTGGGCAGCCAGATCCTGAAAGAACAC
CCCGTGGAAAACACCCAGCTGCAGAACGAGAAGCTGTACCTGTACTACCTGCAGAATGGGCGGGATA
TGTACGTGGACCAGGAACTGGACATCAACCGGCTGTCCGACTACGATGTGGACCATATCGTGCCTCA
GAGCTTTCTGAAGGACGACTCCATCGACAACAAGGTGCTGACCAGAAGCGACAAGAACCGGGGCAA
GAGCGACAACGTGCCCTCCGAAGAGGTCGTGAAGAAGATGAAGAACTACTGGCGGCAGCTGCTGAA
CGCCAAGCTGATTACCCAGAGAAAGTTCGACAATCTGACCAAGGCCGAGAGAGGCGGCCTGAGCGA
ACTGGATAAGGCCGGCTTCATCAAGAGACAGCTGGTGGAAACCCGGCAGATCACAAAGCACGTGGC
ACAGATCCTGGACTCCCGGATGAACACTAAGTACGACGAGAATGACAAGCTGATCCGGGAAGTGAAA
GTGATCACCCTGAAGTCCAAGCTGGTGTCCGATTTCCGGAAGGATTTCCAGTTTTACAAAGTGCGCG
AGATCAACAACTACCACCACGCCCACGACGCCTACCTGAACGCCGTCGTGGGAACCGCCCTGATCA
AAAAGTACCCTAAGCTGGAAAGCGAGTTCGTGTACGGCGACTACAAGGTGTACGACGTGCGGAAGAT
GATCGCCAAGAGCGAGCAGGAAATCGGCAAGGCTACCGCCAAGTACTTCTTCTACAGCAACATCATG
AACTTTTTCAAGACCGAGATTACCCTGGCCAACGGCGAGATCCGGAAGCGGCCTCTGATCGAGACAA
ACGGCGAAACCGGGGAGATCGTGTGGGATAAGGGCCGGGATTTTGCCACCGTGCGGAAAGTGCTG
AGCATGCCCCAAGTGAATATCGTGAAAAAGACCGAGGTGCAGACAGGCGGCTTCAGCAAAGAGTCTA
TCAGACCCAAGAGGAACAGCGATAAGCTGATCGCCAGAAAGAAGGACTGGGACCCTAAGAAGTACG
GCGGCTTCCTGTGGCCCACCGTGGCCTATTCTGTGCTGGTGGTGGCCAAAGTGGAAAAGGGCAAGT
CCAAGAAACTGAAGAGTGTGAAAGAGCTGCTGGGGATCACCATCATGGAAAGAAGCAGCTTCGAGAA
GAATCCCATCGACTTTCTGGAAGCCAAGGGCTACAAAGAAGTGAAAAAGGACCTGATCATCAAGCTG
CCTAAGTACTCCCTGTTCGAGCTGGAAAACGGCCGGAAGAGAATGCTGGCCTCTGCCAAGCAGCTG
CAGAAGGGAAACGAACTGGCCCTGCCCTCCAAATATGTGAACTTCCTGTACCTGGCCAGCCACTATG
AGAAGCTGAAGGGCTCCCCCGAGGATAATGAGCAGAAACAGCTGTTTGTGGAACAGCACAAGCACTA
CCTGGACGAGATCATCGAGCAGATCAGCGAGTTCTCCAAGAGAGTGATCCTGGCCGACGCTAATCTG
GACAAAGTGCTGTCCGCCTACAACAAGCACCGGGATAAGCCCATCAGAGAGCAGGCCGAGAATATCA
TCCACCTGTTTACCCTGACCAGACTGGGAGCCCCTAGAGCCTTCAAGTACTTTGACACCACCATCGA
CCCCAAGCAGTACAGAAGCACCAAAGAGGTGCTGGACGCCACCCTGATCCACCAGAGCATCACCGG
CCTGTACGAGACACGGATCGACCTGTCTCAGCTGGGAGGTGACTCTGGCGGCTCAAAAAGAACCGC
CGACGGCAGCGAATTCGAGCCCAAGAAGAAGAGGAAAGTCTAATTAATTAAGCTGCCTTCTGCGGGG
CTTGCCTTCTGGCCATGCCCTTCTTCTCTCCCTTGCACCTGTACCTCTTGGTCTTTGAATAAAGCCTG
AGTAGGAAGCGACTGTGCCTTCTAGTTGCCAGCCATCTGTTGTTTGCCCCTCCCCCGTGCCTTCCTT
GACCCTGGAAGGTGCCACTCCCACTGTCCTTTCCTAATAAAATGAGAAATTGCATCGCATTGTCTGA
GTAGGTGTCATTCTATTCTGGGGGGTGGGGTGGGGCAGGACAGCAAGGGGGAGGATTGGGAAGAC
AATAGCAGGCATGCTGGGGATGCGGTGGGCTCTATGG

Start	End	Feature Description
1	380	CMV enhancer
381	619	CMV promoter
620	636	T7 promoter with mismatch
637	683	5' UTR
684	740	BP NLS
741	1238	Engineered TadA* variant 8e(V106W)
1239	1334	(SGGS)2 – XTEN – (SGGS)2 linker
1335	5435	Cas9(D10A) SpRY PAM variant
5436	5501	Linker + SV40 BP NLS

5502	5605	3' UTR
5606	5830	bGH poly(A) signal

Amino acid sequence
MKRTADGSEFESPKKKRKVSEVEFSHEYWMRHALTLAKRARDEREVPVGAVLVLNNRVIGEGWNRAIGL
HDPTAHAEIMALRQGGLVMQNYRLIDATLYVTFEPCVMCAGAMIHSRIGRVVFGWRNSKRGAAGSLMNV
LNYPGMNHRVEITEGILADECAALLCDFYRMPRQVFNAQKKAQSSINSGGSSGGSSGSETPGTSESATP
ESSGGSSGGSDKKYSIGLAIGTNSVGWAVITDEYKVPSKKFKVLGNTDRHSIKKNLIGALLFDSGETAERT
RLKRTARRRYTRRKNRICYLQEIFSNEMAKVDDSFFHRLEESFLVEEDKKHERHPIFGNIVDEVAYHEKYP
TIYHLRKKLVDSTDKADLRLIYLALAHMIKFRGHFLIEGDLNPDNSDVDKLFIQLVQTYNQLFEENPINASGV
DAKAILSARLSKSRRLENLIAQLPGEKKNGLFGNLIALSLGLTPNFKSNFDLAEDAKLQLSKDTYDDDLDNL
LAQIGDQYADLFLAAKNLSDAILLSDILRVNTEITKAPLSASMIKRYDEHHQDLTLLKALVRQQLPEKYKEIFF
DQSKNGYAGYIDGGASQEEFYKFIKPILEKMDGTEELLVKLNREDLLRKQRTFDNGSIPHQIHLGELHAILR
RQEDFYPFLKDNREKIEKILTFRIPYYVGPLARGNSRFAWMTRKSEETITPWNFEEVVDKGASAQSFIERM
TNFDKNLPNEKVLPKHSLLYEYFTVYNELTKVKYVTEGMRKPAFLSGEQKKAIVDLLFKTNRKVTVKQLKE
DYFKKIECFDSVEISGVEDRFNASLGTYHDLLKIIKDKDFLDNEENEDILEDIVLTLTLFEDREMIEERLKTYA
HLFDDKVMKQLKRRRYTGWGRLSRKLINGIRDKQSGKTILDFLKSDGFANRNFMQLIHDDSLTFKEDIQKA
QVSGQGDSLHEHIANLAGSPAIKKGILQTVKVVDELVKVMGRHKPENIVIEMARENQTTQKGQKNSRERM
KRIEEGIKELGSQILKEHPVENTQLQNEKLYLYYLQNGRDMYVDQELDINRLSDYDVDHIVPQSFLKDDSID
NKVLTRSDKNRGKSDNVPSEEVVKKMKNYWRQLLNAKLITQRKFDNLTKAERGGLSELDKAGFIKRQLVE
TRQITKHVAQILDSRMNTKYDENDKLIREVKVITLKSKLVSDFRKDFQFYKVREINNYHHAHDAYLNAVVGT
ALIKKYPKLESEFVYGDYKVYDVRKMIAKSEQEIGKATAKYFFYSNIMNFFKTEITLANGEIRKRPLIETNGE
TGEIVWDKGRDFATVRKVLSMPQVNIVKKTEVQTGGFSKESIRPKRNSDKLIARKKDWDPKKYGGFLWPT
VAYSVLVVAKVEKGKSKKLKSVKELLGITIMERSSFEKNPIDFLEAKGYKEVKKDLIIKLPKYSLFELENGRK
RMLASAKQLQKGNELALPSKYVNFLYLASHYEKLKGSPEDNEQKQLFVEQHKHYLDEIIEQISEFSKRVILA
DANLDKVLSAYNKHRDKPIREQAENIIHLFTLTRLGAPRAFKYFDTTIDPKQYRSTKEVLDATLIHQSITGLYE
TRIDLSQLGGDSGGSKRTADGSEFEPKKKRKV

Start	End	Feature Description
1	19	BP NLS
20	185	Engineered TadA* variant 8e
186	217	(SGGS)2 – XTEN – (SGGS)2 linker
218	1584	Cas9(D10A) SpRY PAM variant
1585	1605	Linker + SV40 BP NLS

ABE9 – Cas9 (D10A) – SpRY variant
Plasmid ID: pHS0409

DNA sequence
GACATTGATTATTGACTAGTTATTAATAGTAATCAATTACGGGGTCATTAGTTCATAGCCCATATATGGAG
TTCCGCGTTACATAACTTACGGTAAATGGCCCGCCTGGCTGACCGCCCAACGACCCCCGCCCATTGA
CGTCAATAATGACGTATGTTCCCATAGTAACGCCAATAGGGACTTTCCATTGACGTCAATGGGTGGAGT
ATTTACGGTAAACTGCCCACTTGGCAGTACATCAAGTGTATCATATGCCAAGTACGCCCCCTATTGACG
TCAATGACGGTAAATGGCCCGCCTGGCATTATGCCCAGTACATGACCTTATGGGACTTTCCTACTTGG
CAGTACATCTACGTATTAGTCATCGCTATTACCATGGTGATGCGGTTTTGGCAGTACATCAATGGGCGT
GGATAGCGGTTTGACTCACGGGGATTTCCAAGTCTCCACCCCATTGACGTCAATGGGAGTTTGTTTTG
GCACCAAAATCAACGGGACTTTCCAAAATGTCGTAACAACTCCGCCCCATTGACGCAAATGGGCGGT
AGGCGTGTACGGTGGGAGGTCTATATAAGCAGAGCTGGTTTAGTGAACCGTCAGATCTCGAGCTCGG
TACCTAATACGACACACTATAAGGAAATAAGAGAGAAAAGAAGAGTAAGAAGAAATATAAGAGCCACCA
TGAAACGGACAGCCGACGGAAGCGAGTTCGAGTCACCAAAGAAGAAGCGGAAAGTCTCTGAGGTG
GAGTTTTCCCACGAGTACTGGATGAGACATGCCCTGACCCTGGCCAAGAGGGCACGGGATGAGAGG
GAGGTGCCTGTGGGAGCCGTGCTGGTGCTGAACAATAGAGTGATCGGCGAGGGCTGGAACAGAGC
CATCGGCCTGCACGACCCAACAGCCCATGCCGAAATTATGGCCCTGAGACAGGGCGGCCTGGTCAT
GCAGAACTACAGACTGATTGACGCCACCCTGTACGTGACATTCGAGCCTTGCGTGATGTGCGCCGGC
GCCATGATCCACTCTAGGATCGGCCGCGTGGTGTTTGGCGTGAGGCAGTCAAAAAGAGGCGCCGCA
GGCTCCCTGATGAACGTGCTGAACTACCCCGGCATGAATCACCGCGTCGAAATTACCGAGGGAATCC
TGGCAGATGAATGTGCCGCCCTGACCTGCGATTTCTATCGGATGCCTAGACAGGTGTTCAATGCTCAG
AAGAAGGCCCAGAGCTCCATCAACTCCGGAGGATCTAGCGGAGGCTCCTCTGGCTCTGAGACACCT
GGCACAAGCGAGAGCGCAACACCTGAAAGCAGCGGGGGCAGCAGCGGGGGGTCAGACAAGAAGT
ACAGCATCGGCCTGGCCATCGGCACCAACTCTGTGGGCTGGGCCGTGATCACCGACGAGTACAAGG
TGCCCAGCAAGAAATTCAAGGTGCTGGGCAACACCGACCGGCACAGCATCAAGAAGAACCTGATCG
GAGCCCTGCTGTTCGACAGCGGCGAAACAGCCGAGAGAACCCGGCTGAAGAGAACCGCCAGAAGA
AGATACACCAGACGGAAGAACCGGATCTGCTATCTGCAAGAGATCTTCAGCAACGAGATGGCCAAGG
TGGACGACAGCTTCTTCCACAGACTGGAAGAGTCCTTCCTGGTGGAAGAGGATAAGAAGCACGAGC
GGCACCCCATCTTCGGCAACATCGTGGACGAGGTGGCCTACCACGAGAAGTACCCCACCATCTACCA
CCTGAGAAAGAAACTGGTGGACAGCACCGACAAGGCCGACCTGCGGCTGATCTATCTGGCCCTGGC
CCACATGATCAAGTTCCGGGGCCACTTCCTGATCGAGGGCGACCTGAACCCCGACAACAGCGACGT
GGACAAGCTGTTCATCCAGCTGGTGCAGACCTACAACCAGCTGTTCGAGGAAAACCCCATCAACGCC
AGCGGCGTGGACGCCAAGGCCATCCTGTCTGCCAGACTGAGCAAGAGCAGACGGCTGGAAAATCTG
ATCGCCCAGCTGCCCGGCGAGAAGAAGAATGGCCTGTTCGGAAACCTGATTGCCCTGAGCCTGGGC
CTGACCCCCAACTTCAAGAGCAACTTCGACCTGGCCGAGGATGCCAAACTGCAGCTGAGCAAGGAC
ACCTACGACGACGACCTGGACAACCTGCTGGCCCAGATCGGCGACCAGTACGCCGACCTGTTTCTG
GCCGCCAAGAACCTGTCCGACGCCATCCTGCTGAGCGACATCCTGAGAGTGAACACCGAGATCACC
AAGGCCCCCCTGAGCGCCTCTATGATCAAGAGATACGACGAGCACCACCAGGACCTGACCCTGCTG
AAAGCTCTCGTGCGGCAGCAGCTGCCTGAGAAGTACAAAGAGATTTTCTTCGACCAGAGCAAGAACG
GCTACGCCGGCTACATTGACGGCGGAGCCAGCCAGGAAGAGTTCTACAAGTTCATCAAGCCCATCCT
GGAAAAGATGGACGGCACCGAGGAACTGCTCGTGAAGCTGAACAGAGAGGACCTGCTGCGGAAGC
AGCGGACCTTCGACAACGGCAGCATCCCCCACCAGATCCACCTGGGAGAGCTGCACGCCATTCTGC
GGCGGCAGGAAGATTTTTACCCATTCCTGAAGGACAACCGGGAAAAGATCGAGAAGATCCTGACCTT
CCGCATCCCCTACTACGTGGGCCCTCTGGCCAGGGGAAACAGCAGATTCGCCTGGATGACCAGAAA
GAGCGAGGAAACCATCACCCCCTGGAACTTCGAGGAAGTGGTGGACAAGGGCGCTTCCGCCCAGA
GCTTCATCGAGCGGATGACCAACTTCGATAAGAACCTGCCCAACGAGAAGGTGCTGCCCAAGCACA
GCCTGCTGTACGAGTACTTCACCGTGTATAACGAGCTGACCAAAGTGAAATACGTGACCGAGGGAAT
GAGAAAGCCCGCCTTCCTGAGCGGCGAGCAGAAAAAGGCCATCGTGGACCTGCTGTTCAAGACCAA
CCGGAAAGTGACCGTGAAGCAGCTGAAAGAGGACTACTTCAAGAAAATCGAGTGCTTCGACTCCGTG
GAAATCTCCGGCGTGGAAGATCGGTTCAACGCCTCCCTGGGCACATACCACGATCTGCTGAAAATTAT
CAAGGACAAGGACTTCCTGGACAATGAGGAAAACGAGGACATTCTGGAAGATATCGTGCTGACCCTG
ACACTGTTTGAGGACAGAGAGATGATCGAGGAACGGCTGAAAACCTATGCCCACCTGTTCGACGACA
AAGTGATGAAGCAGCTGAAGCGGCGGAGATACACCGGCTGGGGCAGGCTGAGCCGGAAGCTGATC
AACGGCATCCGGGACAAGCAGTCCGGCAAGACAATCCTGGATTTCCTGAAGTCCGACGGCTTCGCC
AACAGAAACTTCATGCAGCTGATCCACGACGACAGCCTGACCTTTAAAGAGGACATCCAGAAAGCCC

AGGTGTCCGGCCAGGGCGATAGCCTGCACGAGCACATTGCCAATCTGGCCGGCAGCCCCGCCATTA
AGAAGGGCATCCTGCAGACAGTGAAGGTGGTGGACGAGCTCGTGAAAGTGATGGGCCGGCACAAG
CCCGAGAACATCGTGATCGAAATGGCCAGAGAGAACCAGACCACCCAGAAGGGACAGAAGAACAGC
CGCGAGAGAATGAAGCGGATCGAAGAGGGCATCAAAGAGCTGGGCAGCCAGATCCTGAAAGAACAC
CCCGTGGAAAACACCCAGCTGCAGAACGAGAAGCTGTACCTGTACTACCTGCAGAATGGGCGGGATA
TGTACGTGGACCAGGAACTGGACATCAACCGGCTGTCCGACTACGATGTGGACCATATCGTGCCTCA
GAGCTTTCTGAAGGACGACTCCATCGACAACAAGGTGCTGACCAGAAGCGACAAGAACCGGGGCAA
GAGCGACAACGTGCCCTCCGAAGAGGTCGTGAAGAAGATGAAGAACTACTGGCGGCAGCTGCTGAA
CGCCAAGCTGATTACCCAGAGAAAGTTCGACAATCTGACCAAGGCCGAGAGAGGCGGCCTGAGCGA
ACTGGATAAGGCCGGCTTCATCAAGAGACAGCTGGTGGAAACCCGGCAGATCACAAAGCACGTGGC
ACAGATCCTGGACTCCCGGATGAACACTAAGTACGACGAGAATGACAAGCTGATCCGGGAAGTGAAA
GTGATCACCCTGAAGTCCAAGCTGGTGTCCGATTTCCGGAAGGATTTCCAGTTTTACAAAGTGCGCG
AGATCAACAACTACCACCACGCCCACGACGCCTACCTGAACGCCGTCGTGGGAACCGCCCTGATCA
AAAAGTACCCTAAGCTGGAAAGCGAGTTCGTGTACGGCGACTACAAGGTGTACGACGTGCGGAAGAT
GATCGCCAAGAGCGAGCAGGAAATCGGCAAGGCTACCGCCAAGTACTTCTTCTACAGCAACATCATG
AACTTTTTCAAGACCGAGATTACCCTGGCCAACGGCGAGATCCGGAAGCGGCCTCTGATCGAGACAA
ACGGCGAAACCGGGGAGATCGTGTGGGATAAGGGCCGGGATTTTGCCACCGTGCGGAAAGTGCTG
AGCATGCCCCAAGTGAATATCGTGAAAAAGACCGAGGTGCAGACAGGCGGCTTCAGCAAAGAGTCTA
TCAGACCCAAGAGGAACAGCGATAAGCTGATCGCCAGAAAGAAGGACTGGGACCCTAAGAAGTACG
GCGGCTTCCTGTGGCCCACCGTGGCCTATTCTGTGCTGGTGGTGGCCAAAGTGGAAAAGGGCAAGT
CCAAGAAACTGAAGAGTGTGAAAGAGCTGCTGGGGATCACCATCATGGAAAGAAGCAGCTTCGAGAA
GAATCCCATCGACTTTCTGGAAGCCAAGGGCTACAAAGAAGTGAAAAAGGACCTGATCATCAAGCTG
CCTAAGTACTCCCTGTTCGAGCTGGAAAACGGCCGGAAGAGAATGCTGGCCTCTGCCAAGCAGCTG
CAGAAGGGAAACGAACTGGCCCTGCCCTCCAAATATGTGAACTTCCTGTACCTGGCCAGCCACTATG
AGAAGCTGAAGGGCTCCCCCGAGGATAATGAGCAGAAACAGCTGTTTGTGGAACAGCACAAGCACTA
CCTGGACGAGATCATCGAGCAGATCAGCGAGTTCTCCAAGAGAGTGATCCTGGCCGACGCTAATCTG
GACAAAGTGCTGTCCGCCTACAACAAGCACCGGGATAAGCCCATCAGAGAGCAGGCCGAGAATATCA
TCCACCTGTTTACCCTGACCAGACTGGGAGCCCCTAGAGCCTTCAAGTACTTTGACACCACCATCGA
CCCCAAGCAGTACAGAAGCACCAAAGAGGTGCTGGACGCCACCCTGATCCACCAGAGCATCACCGG
CCTGTACGAGACACGGATCGACCTGTCTCAGCTGGGAGGTGACTCTGGCGGCTCAAAAAGAACCGC
CGACGGCAGCGAATTCGAGCCCAAGAAGAAGAGGAAAGTCTAATTAATTAAGCTGCCTTCTGCGGGG
CTTGCCTTCTGGCCATGCCCTTCTTCTCTCCCTTGCACCTGTACCTCTTGGTCTTTGAATAAAGCCTG
AGTAGGAAGCGACTGTGCCTTCTAGTTGCCAGCCATCTGTTGTTTGCCCCTCCCCCGTGCCTTCCTT
GACCCTGGAAGGTGCCACTCCCACTGTCCTTTCCTAATAAAATGAGAAATTGCATCGCATTGTCTGA
GTAGGTGTCATTCTATTCTGGGGGGTGGGGTGGGGCAGGACAGCAAGGGGGAGGATTGGGAAGAC
AATAGCAGGCATGCTGGGGATGCGGTGGGCTCTATGG

Start	End	Feature Description
1	380	CMV enhancer
381	619	CMV promoter
620	636	T7 promoter with mismatch
637	683	5' UTR
684	740	BP NLS
741	1238	Engineered TadA* variant ABE9 (TadA-8e N108Q L145T)
1239	1334	(SGGS)2 – XTEN – (SGGS)2 linker
1335	5435	Cas9(D10A) SpRY PAM variant
5436	5501	Linker + SV40 BP NLS

5502	5605	3’ UTR
5606	5830	bGH poly(A) signal

Amino acid sequence

MKRTADGSEFESPKKKRKVSEVEFSHEYWMRHALTLAKRARDEREVPVGAVLVLNNRVIGEGWNRAIGL
HDPTAHAEIMALRQGGLVMQNYRLIDATLYVTFEPCVMCAGAMIHSRIGRVVFGVRQSKRGAAGSLMNVL
NYPGMNHRVEITEGILADECAALTCDFYRMPRQVFNAQKKAQSSINSGGSSGGSSGSETPGTSESATPE
SSGGSSGGSDKKYSIGLAIGTNSVGWAVITDEYKVPSKKFKVLGNTDRHSIKKNLIGALLFDSGETAERTR
LKRTARRRYTRRKNRICYLQEIFSNEMAKVDDSFFHRLEESFLVEEDKKHERHPIFGNIVDEVAYHEKYPTI
YHLRKKLVDSTDKADLRLIYLALAHMIKFRGHFLIEGDLNPDNSDVDKLFIQLVQTYNQLFEENPINASGVD
AKAILSARLSKSRRLENLIAQLPGEKKNGLFGNLIALSLGLTPNFKSNFDLAEDAKLQLSKDTYDDDLDNLL
AQIGDQYADLFLAAKNLSDAILLSDILRVNTEITKAPLSASMIKRYDEHHQDLTLLKALVRQQLPEKYKEIFFD
QSKNGYAGYIDGGASQEEFYKFIKPILEKMDGTEELLVKLNREDLLRKQRTFDNGSIPHQIHLGELHAILRR
QEDFYPFLKDNREKIEKILTFRIPYYVGPLARGNSRFAWMTRKSEETITPWNFEEVVDKGASAQSFIERMT
NFDKNLPNEKVLPKHSLLYEYFTVYNELTKVKYVTEGMRKPAFLSGEQKKAIVDLLFKTNRKVTVKQLKED
YFKKIECFDSVEISGVEDRFNASLGTYHDLLKIIKDKDFLDNEENEDILEDIVLTLTLFEDREMIEERLKTYAH
LFDDKVMKQLKRRRYTGWGRLSRKLINGIRDKQSGKTILDFLKSDGFANRNFMQLIHDDSLTFKEDIQKAQ
VSGQGDSLHEHIANLAGSPAIKKGILQTVKVVDELVKVMGRHKPENIVIEMARENQTTQKGQKNSRERMK
RIEEGIKELGSQILKEHPVENTQLQNEKLYLYYLQNGRDMYVDQELDINRLSDYDVDHIVPQSFLKDDSIDN
KVLTRSDKNRGKSDNVPSEEVVKKMKNYWRQLLNAKLITQRKFDNLTKAERGGLSELDKAGFIKRQLVET
RQITKHVAQILDSRMNTKYDENDKLIREVKVITLKSKLVSDFRKDFQFYKVREINNYHHAHDAYLNAVVGTA
LIKKYPKLESEFVYGDYKVYDVRKMIAKSEQEIGKATAKYFFYSNIMNFFKTEITLANGEIRKRPLIETNGET
GEIVWDKGRDFATVRKVLSMPQVNIVKKTEVQTGGFSKESIRPKRNSDKLIARKKDWDPKKYGGFLWPTV
AYSVLVVAKVEKGKSKKLKSVKELLGITIMERSSFEKNPIDFLEAKGYKEVKKDLIIKLPKYSLFELENGRKR
MLASAKQLQKGNELALPSKYVNFLYLASHYEKLKGSPEDNEQKQLFVEQHKHYLDEIIEQISEFSKRVILAD
ANLDKVLSAYNKHRDKPIREQAENIIHLFTLTRLGAPRAFKYFDTTIDPKQYRSTKEVLDATLIHQSITGLYET
RIDLSQLGGDSGGSKRTADGSEFEPKKKRKV

Start	End	Feature Description
1	19	BP NLS
20	185	Engineered TadA* variant 8e
186	217	(SGGS)2 – XTEN – (SGGS)2 linker
218	1584	Cas9(D10A) SpRY PAM variant
1585	1605	Linker + SV40 BP NLS

ABE7.10 – Cas9 (D10A) – SpNG variant
Plasmid ID: pHS0385

DNA sequence
GACATTGATTATTGACTAGTTATTAATAGTAATCAATTACGGGGTCATTAGTTCATAGCCCATATATGGAG
TTCCGCGTTACATAACTTACGGTAAATGGCCCGCCTGGCTGACCGCCCAACGACCCCCGCCCATTGA
CGTCAATAATGACGTATGTTCCCATAGTAACGCCAATAGGGACTTTCCATTGACGTCAATGGGTGGAGT
ATTTACGGTAAACTGCCCACTTGGCAGTACATCAAGTGTATCATATGCCAAGTACGCCCCCTATTGACG
TCAATGACGGTAAATGGCCCGCCTGGCATTATGCCCAGTACATGACCTTATGGGACTTTCCTACTTGG
CAGTACATCTACGTATTAGTCATCGCTATTACCATGGTGATGCGGTTTTGGCAGTACATCAATGGGCGT
GGATAGCGGTTTGACTCACGGGGATTTCCAAGTCTCCACCCCATTGACGTCAATGGGAGTTTGTTTTG
GCACCAAAATCAACGGGACTTTCCAAAATGTCGTAACAACTCCGCCCCATTGACGCAAATGGGCGGT
AGGCGTGTACGGTGGGAGGTCTATATAAGCAGAGCTGGTTTAGTGAACCGTCAGATCCGCTAGAGAT
CCGCGGCCGCTAATACGACTCACTATAGGGAGAGCCGCCACCATGAAACGGACAGCCGACGGAAGC
GAGTTCGAGTCACCAAAGAAGAAGCGGAAAGTCTCTGAAGTCGAGTTTAGCCACGAGTATTGGATGA
GGCACGCACTGACCCTGGCAAAGCGAGCATGGGATGAAAGAGAAGTCCCCGTGGGCGCCGTGCTG
GTGCACAACAATAGAGTGATCGGAGAGGGATGGAACAGGCCAATCGGCCGCCACGACCCTACCGCA
CACGCAGAGATCATGGCACTGAGGCAGGGAGGCCTGGTCATGCAGAATTACCGCCTGATCGATGCC
ACCCTGTATGTGACACTGGAGCCATGCGTGATGTGCGCAGGAGCAATGATCCACAGCAGGATCGGAA
GAGTGGTGTTCGGAGCACGGGACGCCAAGACCGGCGCAGCAGGCTCCCTGATGGATGTGCTGCAC
CACCCCGGCATGAACCACCGGGTGGAGATCACAGAGGGAATCCTGGCAGACGAGTGCGCCGCCCT
GCTGAGCGATTTCTTTAGAATGCGGAGACAGGAGATCAAGGCCCAGAAGAAGGCACAGAGCTCCAC
CGACTCTGGAGGATCTAGCGGAGGATCCTCTGGAAGCGAGACACCAGGCACAAGCGAGTCCGCCAC
ACCAGAGAGCTCCGGCGGCTCCTCCGGAGGATCCTCTGAGGTGGAGTTTTCCCACGAGTACTGGAT
GAGACATGCCCTGACCCTGGCCAAGAGGGCACGCGATGAGAGGGAGGTGCCTGTGGGAGCCGTGC
TGGTGCTGAACAATAGAGTGATCGGCGAGGGCTGGAACAGAGCCATCGGCCTGCACGACCCAACAG
CCCATGCCGAAATTATGGCCCTGAGACAGGGCGGCCTGGTCATGCAGAACTACAGACTGATTGACGC
CACCCTGTACGTGACATTCGAGCCTTGCGTGATGTGCGCCGGCGCCATGATCCACTCTAGGATCGGC
CGCGTGGTGTTTGGCGTGAGGAACGCAAAAACCGGCGCCGCAGGCTCCCTGATGGACGTGCTGCA
CTACCCCGGCATGAATCACCGCGTCGAAATTACCGAGGGAATCCTGGCAGATGAATGTGCCGCCCTG
CTGTGCTATTTCTTTCGGATGCCTAGACAGGTGTTCAATGCTCAGAAGAAGGCCCAGAGCTCCACCG
ACTCCGGAGGATCTAGCGGAGGCTCCTCTGGCTCTGAGACACCTGGCACAAGCGAGAGCGCAACAC
CTGAAAGCAGCGGGGGCAGCAGCGGGGGGTCAGACAAGAAGTACAGCATCGGCCTGGCCATCGGC
ACCAACTCTGTGGGCTGGGCCGTGATCACCGACGAGTACAAGGTGCCCAGCAAGAAATTCAAGGTG
CTGGGCAACACCGACCGGCACAGCATCAAGAAGAACCTGATCGGAGCCCTGCTGTTCGACAGCGGC
GAAACAGCCGAGGCCACCCGGCTGAAGAGAACCGCCAGAAGAAGATACACCAGACGGAAGAACCG
GATCTGCTATCTGCAAGAGATCTTCAGCAACGAGATGGCCAAGGTGGACGACAGCTTCTTCCACAGA
CTGGAAGAGTCCTTCCTGGTGGAAGAGGATAAGAAGCACGAGCGGCACCCCATCTTCGGCAACATC
GTGGACGAGGTGGCCTACCACGAGAAGTACCCCACCATCTACCACCTGAGAAAGAAACTGGTGGAC
AGCACCGACAAGGCCGACCTGCGGCTGATCTATCTGGCCCTGGCCCACATGATCAAGTTCCGGGGC
CACTTCCTGATCGAGGGCGACCTGAACCCCGACAACAGCGACGTGGACAAGCTGTTCATCCAGCTG
GTGCAGACCTACAACCAGCTGTTCGAGGAAAACCCCATCAACGCCAGCGGCGTGGACGCCAAGGCC
ATCCTGTCTGCCAGACTGAGCAAGAGCAGACGGCTGGAAAATCTGATCGCCCAGCTGCCCGGCGAG
AAGAAGAATGGCCTGTTCGGAAACCTGATTGCCCTGAGCCTGGGCCTGACCCCCAACTTCAAGAGC
AACTTCGACCTGGCCGAGGATGCCAAACTGCAGCTGAGCAAGGACACCTACGACGACGACCTGGAC
AACCTGCTGGCCCAGATCGGCGACCAGTACGCCGACCTGTTTCTGGCCGCCAAGAACCTGTCCGAC
GCCATCCTGCTGAGCGACATCCTGAGAGTGAACACCGAGATCACCAAGGCCCCCCTGAGCGCCTCT
ATGATCAAGAGATACGACGAGCACCACCAGGACCTGACCCTGCTGAAAGCTCTCGTGCGGCAGCAG
CTGCCTGAGAAGTACAAAGAGATTTTCTTCGACCAGAGCAAGAACGGCTACGCCGGCTACATTGACG
GCGGAGCCAGCCAGGAAGAGTTCTACAAGTTCATCAAGCCCATCCTGGAAAAGATGGACGGCACCG
AGGAACTGCTCGTGAAGCTGAACAGAGAGGACCTGCTGCGGAAGCAGCGGACCTTCGACAACGGC
AGCATCCCCCACCAGATCCACCTGGGAGAGCTGCACGCCATTCTGCGGCGGCAGGAAGATTTTTACC
CATTCCTGAAGGACAACCGGGAAAAGATCGAGAAGATCCTGACCTTCCGCATCCCCTACTACGTGGG
CCCTCTGGCCAGGGGAAACAGCAGATTCGCCTGGATGACCAGAAAGAGCGAGGAAACCATCACCCC
CTGGAACTTCGAGGAAGTGGTGGACAAGGGCGCTTCCGCCCAGAGCTTCATCGAGCGGATGACCAA
CTTCGATAAGAACCTGCCCAACGAGAAGGTGCTGCCCAAGCACAGCCTGCTGTACGAGTACTTCACC

GTGTATAACGAGCTGACCAAAGTGAAATACGTGACCGAGGGAATGAGAAAGCCCGCCTTCCTGAGCG
GCGAGCAGAAAAAGGCCATCGTGGACCTGCTGTTCAAGACCAACCGGAAAGTGACCGTGAAGCAGC
TGAAAGAGGACTACTTCAAGAAAATCGAGTGCTTCGACTCCGTGGAAATCTCCGGCGTGGAAGATCG
GTTCAACGCCTCCCTGGGCACATACCACGATCTGCTGAAAATTATCAAGGACAAGGACTTCCTGGACA
ATGAGGAAAACGAGGACATTCTGGAAGATATCGTGCTGACCCTGACACTGTTTGAGGACAGAGAGAT
GATCGAGGAACGGCTGAAAACCTATGCCCACCTGTTCGACGACAAAGTGATGAAGCAGCTGAAGCG
GCGGAGATACACCGGCTGGGGCAGGCTGAGCCGGAAGCTGATCAACGGCATCCGGGACAAGCAGT
CCGGCAAGACAATCCTGGATTTCCTGAAGTCCGACGGCTTCGCCAACAGAAACTTCATGCAGCTGAT
CCACGACGACAGCCTGACCTTTAAAGAGGACATCCAGAAAGCCCAGGTGTCCGGCCAGGGCGATAG
CCTGCACGAGCACATTGCCAATCTGGCCGGCAGCCCCGCCATTAAGAAGGGCATCCTGCAGACAGT
GAAGGTGGTGGACGAGCTCGTGAAAGTGATGGGCCGGCACAAGCCCGAGAACATCGTGATCGAAAT
GGCCAGAGAGAACCAGACCACCCAGAAGGGACAGAAGAACAGCCGCGAGAGAATGAAGCGGATCG
AAGAGGGCATCAAAGAGCTGGGCAGCCAGATCCTGAAAGAACACCCCGTGGAAAACACCCAGCTGC
AGAACGAGAAGCTGTACCTGTACTACCTGCAGAATGGGCGGGATATGTACGTGGACCAGGAACTGGA
CATCAACCGGCTGTCCGACTACGATGTGGACCATATCGTGCCTCAGAGCTTTCTGAAGGACGACTCC
ATCGACAACAAGGTGCTGACCAGAAGCGACAAGAACCGGGGCAAGAGCGACAACGTGCCCTCCGA
AGAGGTCGTGAAGAAGATGAAGAACTACTGGCGGCAGCTGCTGAACGCCAAGCTGATTACCCAGAG
AAAGTTCGACAATCTGACCAAGGCCGAGAGAGGCGGCCTGAGCGAACTGGATAAGGCCGGCTTCAT
CAAGAGACAGCTGGTGGAAACCCGGCAGATCACAAAGCACGTGGCACAGATCCTGGACTCCCGGAT
GAACACTAAGTACGACGAGAATGACAAGCTGATCCGGGAAGTGAAAGTGATCACCCTGAAGTCCAAG
CTGGTGTCCGATTTCCGGAAGGATTTCCAGTTTTACAAAGTGCGCGAGATCAACAACTACCACCACGC
CCACGACGCCTACCTGAACGCCGTCGTGGGAACCGCCCTGATCAAAAAGTACCCTAAGCTGGAAAG
CGAGTTCGTGTACGGCGACTACAAGGTGTACGACGTGCGGAAGATGATCGCCAAGAGCGAGCAGGA
AATCGGCAAGGCTACCGCCAAGTACTTCTTCTACAGCAACATCATGAACTTTTTCAAGACCGAGATTAC
CCTGGCCAACGGCGAGATCCGGAAGCGGCCTCTGATCGAGACAAACGGCGAAACCGGGGAGATCG
TGTGGGATAAGGGCCGGGATTTTGCCACCGTGCGGAAAGTGCTGAGCATGCCCCAAGTGAATATCGT
GAAAAAGACCGAGGTGCAGACAGGCGGCTTCAGCAAAGAGTCTATCCGGCCCAAGAGGAACAGCGA
TAAGCTGATCGCCAGAAAGAAGGACTGGGACCCTAAGAAGTACGGCGGCTTCGTCAGCCCCACCGT
GGCCTATTCTGTGCTGGTGGTGGCCAAAGTGGAAAAGGGCAAGTCCAAGAAACTGAAGAGTGTGAA
AGAGCTGCTGGGGATCACCATCATGGAAAGAAGCAGCTTCGAGAAGAATCCCATCGACTTTCTGGAA
GCCAAGGGCTACAAAGAAGTGAAAAAGGACCTGATCATCAAGCTGCCTAAGTACTCCCTGTTCGAGC
TGGAAAACGGCCGGAAGAGAATGCTGGCCTCTGCCCGCTTCCTGCAGAAGGGAAACGAACTGGCC
CTGCCCTCCAAATATGTGAACTTCCTGTACCTGGCCAGCCACTATGAGAAGCTGAAGGGCTCCCCCG
AGGATAATGAGCAGAAACAGCTGTTTGTGGAACAGCACAAGCACTACCTGGACGAGATCATCGAGCA
GATCAGCGAGTTCTCCAAGAGAGTGATCCTGGCCGACGCTAATCTGGACAAAGTGCTGTCCGCCTAC
AACAAGCACCGGGATAAGCCCATCAGAGAGCAGGCCGAGAATATCATCCACCTGTTTACCCTGACCA
ATCTGGGAGCCCCTCGCGCCTTCAAGTACTTTGACACCACCATCGACCGGAAGGTGTACCGCAGCA
CCAAAGAGGTGCTGGACGCCACCCTGATCCACCAGAGCATCACCGGCCTGTACGAGACACGGATCG
ACCTGTCTCAGCTGGGAGGTGACTCTGGCGGCTCAAAAAGAACCGCCGACGGCAGCGAATTCGAGC
CCAAGAAGAAGAGGAAAGTCTAACCGGTCATCATCACCATCACCATTGAGTTTAAACCCGCTGATCAG
CCTCGACTGTGCCTTCTAGTTGCCAGCCATCTGTTGTTTGCCCCTCCCCCGTGCCTTCCTTGACCCT
GGAAGGTGCCACTCCCACTGTCCTTTCCTAATAAAATGAGGAAATTGCATCGCATTGTCTGAGTAGGT
GTCATTCTATTCTGGGGGGTGGGGTGGGGCAGGACAGCAAGGGGGAGGATTGGGAAGACAATAGCA
GGCATGCTGGGGATGCGGTGGGCTCTATGG

Start	End	Feature Description
1	380	CMV enhancer
381	625	CMV promoter
626	642	T7 promoter
643	657	5' UTR
658	714	BP NLS

715	1212	TadA wild type
1213	1308	(SGGS)2 – XTEN – (SGGS)2 linker
1309	1806	Engineered TadA* variant 7.10
1807	1902	(SGGS)2 – XTEN – (SGGS)2 linker
1903	6003	Cas9(D10A) SpNG PAM variant
6004	6069	Linker + SV40 BP NLS
6070	6117	3' UTR
6118	6345	bGH poly(A) signal

Amino acid sequence

MKRTADGSEFESPKKKRKVSEVEFSHEYWMRHALTLAKRAWDEREVPVGAVLVHNNRVIGEGWNRPIG
RHDPTAHAEIMALRQGGLVMQNYRLIDATLYVTLEPCVMCAGAMIHSRIGRVVFGARDAKTGAAGSLMDV
LHHPGMNHRVEITEGILADECAALLSDFFRMRRQEIKAQKKAQSSTDSGGSSGGSSGSETPGTSESATP
ESSGGSSGGSSEVEFSHEYWMRHALTLAKRARDEREVPVGAVLVLNNRVIGEGWNRAIGLHDPTAHAEI
MALRQGGLVMQNYRLIDATLYVTFEPCVMCAGAMIHSRIGRVVFGVRNAKTGAAGSLMDVLHYPGMNHR
VEITEGILADECAALLCYFFRMPRQVFNAQKKAQSSTDSGGSSGGSSGSETPGTSESATPESSGGSSGG
SDKKYSIGLAIGTNSVGWAVITDEYKVPSKKFKVLGNTDRHSIKKNLIGALLFDSGETAEATRLKRTARRRY
TRRKNRICYLQEIFSNEMAKVDDSFFHRLEESFLVEEDKKHERHPIFGNIVDEVAYHEKYPTIYHLRKKLVD
STDKADLRLIYLALAHMIKFRGHFLIEGDLNPDNSDVDKLFIQLVQTYNQLFEENPINASGVDAKAILSARLS
KSRRLENLIAQLPGEKKNGLFGNLIALSLGLTPNFKSNFDLAEDAKLQLSKDTYDDDLDNLLAQIGDQYADL
FLAAKNLSDAILLSDILRVNTEITKAPLSASMIKRYDEHHQDLTLLKALVRQQLPEKYKEIFFDQSKNGYAGYI
DGGASQEEFYKFIKPILEKMDGTEELLVKLNREDLLRKQRTFDNGSIPHQIHLGELHAILRRQEDFYPFLKD
NREKIEKILTFRIPYYVGPLARGNSRFAWMTRKSEETITPWNFEEVVDKGASAQSFIERMTNFDKNLPNEK
VLPKHSLLYEYFTVYNELTKVKYVTEGMRKPAFLSGEQKKAIVDLLFKTNRKVTVKQLKEDYFKKIECFDSV
EISGVEDRFNASLGTYHDLLKIIKDKDFLDNEENEDILEDIVLTLTLFEDREMIEERLKTYAHLFDDKVMKQLK
RRRYTGWGRLSRKLINGIRDKQSGKTILDFLKSDGFANRNFMQLIHDDSLTFKEDIQKAQVSGQGDSLHE
HIANLAGSPAIKKGILQTVKVVDELVKVMGRHKPENIVIEMARENQTTQKGQKNSRERMKRIEEGIKELGS
QILKEHPVENTQLQNEKLYLYYLQNGRDMYVDQELDINRLSDYDVDHIVPQSFLKDDSIDNKVLTRSDKNR
GKSDNVPSEEVVKKMKNYWRQLLNAKLITQRKFDNLTKAERGGLSELDKAGFIKRQLVETRQITKHVAQIL
DSRMNTKYDENDKLIREVKVITLKSKLVSDFRKDFQFYKVREINNYHHAHDAYLNAVVGTALIKKYPKLESE
FVYGDYKVYDVRKMIAKSEQEIGKATAKYFFYSNIMNFFKTEITLANGEIRKRPLIETNGETGEIVWDKGRD
FATVRKVLSMPQVNIVKKTEVQTGGFSKESIRPKRNSDKLIARKKDWDPKKYGGFVSPTVAYSVLVVAKVE
KGKSKKLKSVKELLGITIMERSSFEKNPIDFLEAKGYKEVKKDLIIKLPKYSLFELENGRKRMLASARFLQKG
NELALPSKYVNFLYLASHYEKLKGSPEDNEQKQLFVEQHKHYLDEIIEQISEFSKRVILADANLDKVLSAYN
KHRDKPIREQAENIIHLFTLTNLGAPRAFKYFDTTIDRKVYRSTKEVLDATLIHQSITGLYETRIDLSQLGGDS
GGSKRTADGSEFEPKKKRKV

Start	End	Feature Description
1	19	BP NLS
20	185	TadA wild type
186	217	(SGGS)2 – XTEN – (SGGS)2 linker
218	383	Engineered TadA* variant 7.10
384	415	(SGGS)2 – XTEN – (SGGS)2 linker

416	1782	Cas9(D10A) SpNG PAM variant
1783	1803	Linker + SV40 BP NLS

ABE8e – Cas9 (D10A) – SpNG variant
Plasmid ID: pHS0379

DNA sequence
GACATTGATTATTGACTAGTTATTAATAGTAATCAATTACGGGGTCATTAGTTCATAGCCCATATATGGAG
TTCCGCGTTACATAACTTACGGTAAATGGCCCGCCTGGCTGACCGCCCAACGACCCCCGCCCATTGA
CGTCAATAATGACGTATGTTCCCATAGTAACGCCAATAGGGACTTTCCATTGACGTCAATGGGTGGAGT
ATTTACGGTAAACTGCCCACTTGGCAGTACATCAAGTGTATCATATGCCAAGTACGCCCCCTATTGACG
TCAATGACGGTAAATGGCCCGCCTGGCATTATGCCCAGTACATGACCTTATGGGACTTTCCTACTTGG
CAGTACATCTACGTATTAGTCATCGCTATTACCATGGTGATGCGGTTTTGGCAGTACATCAATGGGCGT
GGATAGCGGTTTGACTCACGGGGATTTCCAAGTCTCCACCCCATTGACGTCAATGGGAGTTTGTTTTG
GCACCAAAATCAACGGGACTTTCCAAAATGTCGTAACAACTCCGCCCCATTGACGCAAATGGGCGGT
AGGCGTGTACGGTGGGAGGTCTATATAAGCAGAGCTGGTTTAGTGAACCGTCAGATCTCGAGCTCGG
TACCTAATACGACACACTATAAGGAAATAAGAGAGAAAAGAAGAGTAAGAAGAAATATAAGAGCCACCA
TGAAACGGACAGCCGACGGAAGCGAGTTCGAGTCACCAAAGAAGAAGCGGAAAGTCTCTGAGGTG
GAGTTTTCCCACGAGTACTGGATGAGACATGCCCTGACCCTGGCCAAGAGGGCACGGGATGAGAGG
GAGGTGCCTGTGGGAGCCGTGCTGGTGCTGAACAATAGAGTGATCGGCGAGGGCTGGAACAGAGC
CATCGGCCTGCACGACCCAACAGCCCATGCCGAAATTATGGCCCTGAGACAGGGCGGCCTGGTCAT
GCAGAACTACAGACTGATTGACGCCACCCTGTACGTGACATTCGAGCCTTGCGTGATGTGCGCCGGC
GCCATGATCCACTCTAGGATCGGCCGCGTGGTGTTTGGCGTGAGGAACTCAAAAAGAGGCGCCGCA
GGCTCCCTGATGAACGTGCTGAACTACCCCGGCATGAATCACCGCGTCGAAATTACCGAGGGAATCC
TGGCAGATGAATGTGCCGCCCTGCTGTGCGATTTCTATCGGATGCCTAGACAGGTGTTCAATGCTCAG
AAGAAGGCCCAGAGCTCCATCAACTCCGGAGGATCTAGCGGAGGCTCCTCTGGCTCTGAGACACCT
GGCACAAGCGAGAGCGCAACACCTGAAAGCAGCGGGGGCAGCAGCGGGGGGTCAGACAAGAAGT
ACAGCATCGGCCTGGCCATCGGCACCAACTCTGTGGGCTGGGCCGTGATCACCGACGAGTACAAGG
TGCCCAGCAAGAAATTCAAGGTGCTGGGCAACACCGACCGGCACAGCATCAAGAAGAACCTGATCG
GAGCCCTGCTGTTCGACAGCGGCGAAACAGCCGAGGCCACCCGGCTGAAGAGAACCGCCAGAAGA
AGATACACCAGACGGAAGAACCGGATCTGCTATCTGCAAGAGATCTTCAGCAACGAGATGGCCAAGG
TGGACGACAGCTTCTTCCACAGACTGGAAGAGTCCTTCCTGGTGGAAGAGGATAAGAAGCACGAGC
GGCACCCCATCTTCGGCAACATCGTGGACGAGGTGGCCTACCACGAGAAGTACCCCACCATCTACCA
CCTGAGAAAGAAACTGGTGGACAGCACCGACAAGGCCGACCTGCGGCTGATCTATCTGGCCCTGGC
CCACATGATCAAGTTCCGGGGCCACTTCCTGATCGAGGGCGACCTGAACCCCGACAACAGCGACGT
GGACAAGCTGTTCATCCAGCTGGTGCAGACCTACAACCAGCTGTTCGAGGAAAACCCCATCAACGCC
AGCGGCGTGGACGCCAAGGCCATCCTGTCTGCCAGACTGAGCAAGAGCAGACGGCTGGAAAATCTG
ATCGCCCAGCTGCCCGGCGAGAAGAAGAATGGCCTGTTCGGAAACCTGATTGCCCTGAGCCTGGGC
CTGACCCCCAACTTCAAGAGCAACTTCGACCTGGCCGAGGATGCCAAACTGCAGCTGAGCAAGGAC
ACCTACGACGACGACCTGGACAACCTGCTGGCCCAGATCGGCGACCAGTACGCCGACCTGTTTCTG
GCCGCCAAGAACCTGTCCGACGCCATCCTGCTGAGCGACATCCTGAGAGTGAACACCGAGATCACC
AAGGCCCCCCTGAGCGCCTCTATGATCAAGAGATACGACGAGCACCACCAGGACCTGACCCTGCTG
AAAGCTCTCGTGCGGCAGCAGCTGCCTGAGAAGTACAAAGAGATTTTCTTCGACCAGAGCAAGAACG
GCTACGCCGGCTACATTGACGGCGGAGCCAGCCAGGAAGAGTTCTACAAGTTCATCAAGCCCATCCT
GGAAAAGATGGACGGCACCGAGGAACTGCTCGTGAAGCTGAACAGAGAGGACCTGCTGCGGAAGC
AGCGGACCTTCGACAACGGCAGCATCCCCCACCAGATCCACCTGGGAGAGCTGCACGCCATTCTGC
GGCGGCAGGAAGATTTTTACCCATTCCTGAAGGACAACCGGGAAAAGATCGAGAAGATCCTGACCTT
CCGCATCCCCTACTACGTGGGCCCTCTGGCCAGGGGAAACAGCAGATTCGCCTGGATGACCAGAAA
GAGCGAGGAAACCATCACCCCCTGGAACTTCGAGGAAGTGGTGGACAAGGGCGCTTCCGCCCAGA
GCTTCATCGAGCGGATGACCAACTTCGATAAGAACCTGCCCAACGAGAAGGTGCTGCCCAAGCACA
GCCTGCTGTACGAGTACTTCACCGTGTATAACGAGCTGACCAAAGTGAAATACGTGACCGAGGGAAT
GAGAAAGCCCGCCTTCCTGAGCGGCGAGCAGAAAAAGGCCATCGTGGACCTGCTGTTCAAGACCAA
CCGGAAAGTGACCGTGAAGCAGCTGAAAGAGGACTACTTCAAGAAAATCGAGTGCTTCGACTCCGTG
GAAATCTCCGGCGTGGAAGATCGGTTCAACGCCTCCCTGGGCACATACCACGATCTGCTGAAAATTAT
CAAGGACAAGGACTTCCTGGACAATGAGGAAAACGAGGACATTCTGGAAGATATCGTGCTGACCCTG
ACACTGTTTGAGGACAGAGAGATGATCGAGGAACGGCTGAAAACCTATGCCCACCTGTTCGACGACA
AAGTGATGAAGCAGCTGAAGCGGCGGAGATACACCGGCTGGGGCAGGCTGAGCCGGAAGCTGATC
AACGGCATCCGGGACAAGCAGTCCGGCAAGACAATCCTGGATTTCCTGAAGTCCGACGGCTTCGCC
AACAGAAACTTCATGCAGCTGATCCACGACGACAGCCTGACCTTTAAAGAGGACATCCAGAAAGCCC

AGGTGTCCGGCCAGGGCGATAGCCTGCACGAGCACATTGCCAATCTGGCCGGCAGCCCCGCCATTA
AGAAGGGCATCCTGCAGACAGTGAAGGTGGTGGACGAGCTCGTGAAAGTGATGGGCCGGCACAAG
CCCGAGAACATCGTGATCGAAATGGCCAGAGAGAACCAGACCACCCAGAAGGGACAGAAGAACAGC
CGCGAGAGAATGAAGCGGATCGAAGAGGGCATCAAAGAGCTGGGCAGCCAGATCCTGAAAGAACAC
CCCGTGGAAAACACCCAGCTGCAGAACGAGAAGCTGTACCTGTACTACCTGCAGAATGGGCGGGATA
TGTACGTGGACCAGGAACTGGACATCAACCGGCTGTCCGACTACGATGTGGACCATATCGTGCCTCA
GAGCTTTCTGAAGGACGACTCCATCGACAACAAGGTGCTGACCAGAAGCGACAAGAACCGGGGCAA
GAGCGACAACGTGCCCTCCGAAGAGGTCGTGAAGAAGATGAAGAACTACTGGCGGCAGCTGCTGAA
CGCCAAGCTGATTACCCAGAGAAAGTTCGACAATCTGACCAAGGCCGAGAGAGGCGGCCTGAGCGA
ACTGGATAAGGCCGGCTTCATCAAGAGACAGCTGGTGGAAACCCGGCAGATCACAAAGCACGTGGC
ACAGATCCTGGACTCCCGGATGAACACTAAGTACGACGAGAATGACAAGCTGATCCGGGAAGTGAAA
GTGATCACCCTGAAGTCCAAGCTGGTGTCCGATTTCCGGAAGGATTTCCAGTTTTACAAAGTGCGCG
AGATCAACAACTACCACCACGCCCACGACGCCTACCTGAACGCCGTCGTGGGAACCGCCCTGATCA
AAAAGTACCCTAAGCTGGAAAGCGAGTTCGTGTACGGCGACTACAAGGTGTACGACGTGCGGAAGAT
GATCGCCAAGAGCGAGCAGGAAATCGGCAAGGCTACCGCCAAGTACTTCTTCTACAGCAACATCATG
AACTTTTTCAAGACCGAGATTACCCTGGCCAACGGCGAGATCCGGAAGCGGCCTCTGATCGAGACAA
ACGGCGAAACCGGGGAGATCGTGTGGGATAAGGGCCGGGATTTTGCCACCGTGCGGAAAGTGCTG
AGCATGCCCCAAGTGAATATCGTGAAAAAGACCGAGGTGCAGACAGGCGGCTTCAGCAAAGAGTCTA
TCAGGCCCAAGAGGAACAGCGATAAGCTGATCGCCAGAAAGAAGGACTGGGACCCTAAGAAGTACG
GCGGCTTCGTCAGCCCCACCGTGGCCTATTCTGTGCTGGTGGTGGCCAAAGTGGAAAAGGGCAAGT
CCAAGAAACTGAAGAGTGTGAAAGAGCTGCTGGGGATCACCATCATGGAAAGAAGCAGCTTCGAGAA
GAATCCCATCGACTTTCTGGAAGCCAAGGGCTACAAAGAAGTGAAAAAGGACCTGATCATCAAGCTG
CCTAAGTACTCCCTGTTCGAGCTGGAAAACGGCCGGAAGAGAATGCTGGCCTCTGCCAGATTCCTGC
AGAAGGGAAACGAACTGGCCCTGCCCTCCAAATATGTGAACTTCCTGTACCTGGCCAGCCACTATGA
GAAGCTGAAGGGCTCCCCCGAGGATAATGAGCAGAAACAGCTGTTTGTGGAACAGCACAAGCACTA
CCTGGACGAGATCATCGAGCAGATCAGCGAGTTCTCCAAGAGAGTGATCCTGGCCGACGCTAATCTG
GACAAAGTGCTGTCCGCCTACAACAAGCACCGGGATAAGCCCATCAGAGAGCAGGCCGAGAATATCA
TCCACCTGTTTACCCTGACCAATCTGGGAGCCCCTAGGGCCTTCAAGTACTTTGACACCACCATCGAC
CGGAAGGTGTACAGGAGCACCAAAGAGGTGCTGGACGCCACCCTGATCCACCAGAGCATCACCGGC
CTGTACGAGACACGGATCGACCTGTCTCAGCTGGGAGGTGACTCTGGCGGCTCAAAAAGAACCGCC
GACGGCAGCGAATTCGAGCCCAAGAAGAAGAGGAAAGTCTAATTAATTAAGCTGCCTTCTGCGGGGC
TTGCCTTCTGGCCATGCCCTTCTTCTCTCCCTTGCACCTGTACCTCTTGGTCTTTGAATAAAGCCTGA
GTAGGAAGCGACTGTGCCTTCTAGTTGCCAGCCATCTGTTGTTTGCCCCTCCCCCGTGCCTTCCTTG
ACCCTGGAAGGTGCCACTCCCACTGTCCTTTCCTAATAAAATGAGAAATTGCATCGCATTGTCTGAG
TAGGTGTCATTCTATTCTGGGGGGTGGGGTGGGGCAGGACAGCAAGGGGGAGGATTGGGAAGACAA
TAGCAGGCATGCTGGGGATGCGGTGGGCTCTATGG

Start	End	Feature Description
1	380	CMV enhancer
381	619	CMV promoter
620	636	T7 promoter with mismatch
637	683	5' UTR
684	740	BP NLS
741	1238	Engineered TadA* variant 8e
1239	1334	(SGGS)2 – XTEN – (SGGS)2 linker
1335	5435	Cas9(D10A) SpNG PAM variant
5436	5501	Linker + SV40 BP NLS

5502	5605	3' UTR
5606	5830	bGH poly(A) signal

Amino acid sequence

MKRTADGSEFESPKKKRKVSEVEFSHEYWMRHALTLAKRARDEREVPVGAVLVLNNRVIGEGWNRAIGL
HDPTAHAEIMALRQGGLVMQNYRLIDATLYVTFEPCVMCAGAMIHSRIGRVVFGVRNSKRGAAGSLMNVL
NYPGMNHRVEITEGILADECAALLCDFYRMPRQVFNAQKKAQSSINSGGSSGGSSGSETPGTSESATPE
SSGGSSGGSDKKYSIGLAIGTNSVGWAVITDEYKVPSKKFKVLGNTDRHSIKKNLIGALLFDSGETAEATRL
KRTARRRYTRRKNRICYLQEIFSNEMAKVDDSFFHRLEESFLVEEDKKHERHPIFGNIVDEVAYHEKYPTIY
HLRKKLVDSTDKADLRLIYLALAHMIKFRGHFLIEGDLNPDNSDVDKLFIQLVQTYNQLFEENPINASGVDA
KAILSARLSKSRRLENLIAQLPGEKKNGLFGNLIALSLGLTPNFKSNFDLAEDAKLQLSKDTYDDDLDNLLA
QIGDQYADLFLAAKNLSDAILLSDILRVNTEITKAPLSASMIKRYDEHHQDLTLLKALVRQQLPEKYKEIFFD
QSKNGYAGYIDGGASQEEFYKFIKPILEKMDGTEELLVKLNREDLLRKQRTFDNGSIPHQIHLGELHAILRR
QEDFYPFLKDNREKIEKILTFRIPYYVGPLARGNSRFAWMTRKSEETITPWNFEEVVDKGASAQSFIERMT
NFDKNLPNEKVLPKHSLLYEYFTVYNELTKVKYVTEGMRKPAFLSGEQKKAIVDLLFKTNRKVTVKQLKED
YFKKIECFDSVEISGVEDRFNASLGTYHDLLKIIKDKDFLDNEENEDILEDIVLTLTLFEDREMIEERLKTYAH
LFDDKVMKQLKRRRYTGWGRLSRKLINGIRDKQSGKTILDFLKSDGFANRNFMQLIHDDSLTFKEDIQKAQ
VSGQGDSLHEHIANLAGSPAIKKGILQTVKVVDELVKVMGRHKPENIVIEMARENQTTQKGQKNSRERMK
RIEEGIKELGSQILKEHPVENTQLQNEKLYLYYLQNGRDMYVDQELDINRLSDYDVDHIVPQSFLKDDSIDN
KVLTRSDKNRGKSDNVPSEEVVKKMKNYWRQLLNAKLITQRKFDNLTKAERGGLSELDKAGFIKRQLVET
RQITKHVAQILDSRMNTKYDENDKLIREVKVITLKSKLVSDFRKDFQFYKVREINNYHHAHDAYLNAVVGTA
LIKKYPKLESEFVYGDYKVYDVRKMIAKSEQEIGKATAKYFFYSNIMNFFKTEITLANGEIRKRPLIETNGET
GEIVWDKGRDFATVRKVLSMPQVNIVKKTEVQTGGFSKESIRPKRNSDKLIARKKDWDPKKYGGFVSPTV
AYSVLVVAKVEKGKSKKLKSVKELLGITIMERSSFEKNPIDFLEAKGYKEVKKDLIIKLPKYSLFELENGRKR
MLASARFLQKGNELALPSKYVNFLYLASHYEKLKGSPEDNEQKQLFVEQHKHYLDEIIEQISEFSKRVILAD
ANLDKVLSAYNKHRDKPIREQAENIIHLFTLTNLGAPRAFKYFDTTIDRKVYRSTKEVLDATLIHQSITGLYET
RIDLSQLGGDSGGSKRTADGSEFEPKKKRKV

Start	End	Feature Description
1	19	BP NLS
20	185	Engineered TadA* variant 8e
186	217	(SGGS)2 – XTEN – (SGGS)2 linker
218	1584	Cas9(D10A) SpNG PAM variant
1585	1605	Linker + SV40 BP NLS

ABE8e(V106W) – Cas9 (D10A) – SpNG variant
Plasmid ID: pHS0408

DNA sequence

GACATTGATTATTGACTAGTTATTAATAGTAATCAATTACGGGGTCATTAGTTCATAGCCCATATATGGAG
TTCCGCGTTACATAACTTACGGTAAATGGCCCGCCTGGCTGACCGCCCAACGACCCCCGCCCATTGA
CGTCAATAATGACGTATGTTCCCATAGTAACGCCAATAGGGACTTTCCATTGACGTCAATGGGTGGAGT
ATTTACGGTAAACTGCCCACTTGGCAGTACATCAAGTGTATCATATGCCAAGTACGCCCCCTATTGACG
TCAATGACGGTAAATGGCCCGCCTGGCATTATGCCCAGTACATGACCTTATGGGACTTTCCTACTTGG
CAGTACATCTACGTATTAGTCATCGCTATTACCATGGTGATGCGGTTTTGGCAGTACATCAATGGGCGT
GGATAGCGGTTTGACTCACGGGGATTTCCAAGTCTCCACCCCATTGACGTCAATGGGAGTTTGTTTTG
GCACCAAAATCAACGGGACTTTCCAAAATGTCGTAACAACTCCGCCCCATTGACGCAAATGGGCGGT
AGGCGTGTACGGTGGGAGGTCTATATAAGCAGAGCTGGTTTAGTGAACCGTCAGATCTCGAGCTCGG
TACCTAATACGACACACTATAAGGAAATAAGAGAGAAAAGAAGAGTAAGAAGAAATATAAGAGCCACCA
TGAAACGGACAGCCGACGGAAGCGAGTTCGAGTCACCAAAGAAGAAGCGGAAAGTCTCTGAGGTG
GAGTTTTCCCACGAGTACTGGATGAGACATGCCCTGACCCTGGCCAAGAGGGCACGGGATGAGAGG
GAGGTGCCTGTGGGAGCCGTGCTGGTGCTGAACAATAGAGTGATCGGCGAGGGCTGGAACAGAGC
CATCGGCCTGCACGACCCAACAGCCCATGCCGAAATTATGGCCCTGAGACAGGGCGGCCTGGTCAT
GCAGAACTACAGACTGATTGACGCCACCCTGTACGTGACATTCGAGCCTTGCGTGATGTGCGCCGGC
GCCATGATCCACTCTAGGATCGGCCGCGTGGTGTTTGGATGGAGAAATTCTAAAAGAGGCGCCGCAG
GCTCCCTGATGAACGTGCTGAACTACCCCGGCATGAATCACCGCGTCGAAATTACCGAGGGAATCCT
GGCAGATGAATGTGCCGCCCTGCTGTGCGATTTCTATCGGATGCCTAGACAGGTGTTCAATGCTCAG
AAGAAGGCCCAGAGCTCCATCAACTCCGGAGGATCTAGCGGAGGCTCCTCTGGCTCTGAGACACCT
GGCACAAGCGAGAGCGCAACACCTGAAAGCAGCGGGGGCAGCAGCGGGGGGTCAGACAAGAAGT
ACAGCATCGGCCTGGCCATCGGCACCAACTCTGTGGGCTGGGCCGTGATCACCGACGAGTACAAGG
TGCCCAGCAAGAAATTCAAGGTGCTGGGCAACACCGACCGGCACAGCATCAAGAAGAACCTGATCG
GAGCCCTGCTGTTCGACAGCGGCGAAACAGCCGAGGCCACCCGGCTGAAGAGAACCGCCAGAAGA
AGATACACCAGACGGAAGAACCGGATCTGCTATCTGCAAGAGATCTTCAGCAACGAGATGGCCAAGG
TGGACGACAGCTTCTTCCACAGACTGGAAGAGTCCTTCCTGGTGGAAGAGGATAAGAAGCACGAGC
GGCACCCCATCTTCGGCAACATCGTGGACGAGGTGGCCTACCACGAGAAGTACCCCACCATCTACCA
CCTGAGAAAGAAACTGGTGGACAGCACCGACAAGGCCGACCTGCGGCTGATCTATCTGGCCCTGGC
CCACATGATCAAGTTCCGGGGCCACTTCCTGATCGAGGGCGACCTGAACCCCGACAACAGCGACGT
GGACAAGCTGTTCATCCAGCTGGTGCAGACCTACAACCAGCTGTTCGAGGAAAACCCCATCAACGCC
AGCGGCGTGGACGCCAAGGCCATCCTGTCTGCCAGACTGAGCAAGAGCAGACGGCTGGAAAATCTG
ATCGCCCAGCTGCCCGGCGAGAAGAAGAATGGCCTGTTCGGAAACCTGATTGCCCTGAGCCTGGGC
CTGACCCCCAACTTCAAGAGCAACTTCGACCTGGCCGAGGATGCCAAACTGCAGCTGAGCAAGGAC
ACCTACGACGACGACCTGGACAACCTGCTGGCCCAGATCGGCGACCAGTACGCCGACCTGTTTCTG
GCCGCCAAGAACCTGTCCGACGCCATCCTGCTGAGCGACATCCTGAGAGTGAACACCGAGATCACC
AAGGCCCCCCTGAGCGCCTCTATGATCAAGAGATACGACGAGCACCACCAGGACCTGACCCTGCTG
AAAGCTCTCGTGCGGCAGCAGCTGCCTGAGAAGTACAAAGAGATTTTCTTCGACCAGAGCAAGAACG
GCTACGCCGGCTACATTGACGGCGGAGCCAGCCAGGAAGAGTTCTACAAGTTCATCAAGCCCATCCT
GGAAAAGATGGACGGCACCGAGGAACTGCTCGTGAAGCTGAACAGAGAGGACCTGCTGCGGAAGC
AGCGGACCTTCGACAACGGCAGCATCCCCCACCAGATCCACCTGGGAGAGCTGCACGCCATTCTGC
GGCGGCAGGAAGATTTTTACCCATTCCTGAAGGACAACCGGGAAAAGATCGAGAAGATCCTGACCTT
CCGCATCCCCTACTACGTGGGCCCTCTGGCCAGGGGAAACAGCAGATTCGCCTGGATGACCAGAAA
GAGCGAGGAAACCATCACCCCCTGGAACTTCGAGGAAGTGGTGGACAAGGGCGCTTCCGCCCAGA
GCTTCATCGAGCGGATGACCAACTTCGATAAGAACCTGCCCAACGAGAAGGTGCTGCCCAAGCACA
GCCTGCTGTACGAGTACTTCACCGTGTATAACGAGCTGACCAAAGTGAAATACGTGACCGAGGGAAT
GAGAAAGCCCGCCTTCCTGAGCGGCGAGCAGAAAAAGGCCATCGTGGACCTGCTGTTCAAGACCAA
CCGGAAAGTGACCGTGAAGCAGCTGAAAGAGGACTACTTCAAGAAAATCGAGTGCTTCGACTCCGTG
GAAATCTCCGGCGTGGAAGATCGGTTCAACGCCTCCCTGGGCACATACCACGATCTGCTGAAAATTAT
CAAGGACAAGGACTTCCTGGACAATGAGGAAAACGAGGACATTCTGGAAGATATCGTGCTGACCCTG
ACACTGTTTGAGGACAGAGAGATGATCGAGGAACGGCTGAAAACCTATGCCCACCTGTTCGACGACA
AAGTGATGAAGCAGCTGAAGCGGCGGAGATACACCGGCTGGGGCAGGCTGAGCCGGAAGCTGATC
AACGGCATCCGGGACAAGCAGTCCGGCAAGACAATCCTGGATTTCCTGAAGTCCGACGGCTTCGCC
AACAGAAACTTCATGCAGCTGATCCACGACGACAGCCTGACCTTTAAAGAGGACATCCAGAAAGCCC

AGGTGTCCGGCCAGGGCGATAGCCTGCACGAGCACATTGCCAATCTGGCCGGCAGCCCCGCCATTA
AGAAGGGCATCCTGCAGACAGTGAAGGTGGTGGACGAGCTCGTGAAAGTGATGGGCCGGCACAAG
CCCGAGAACATCGTGATCGAAATGGCCAGAGAGAACCAGACCACCCAGAAGGGACAGAAGAACAGC
CGCGAGAGAATGAAGCGGATCGAAGAGGGCATCAAAGAGCTGGGCAGCCAGATCCTGAAAGAACAC
CCCGTGGAAAACACCCAGCTGCAGAACGAGAAGCTGTACCTGTACTACCTGCAGAATGGGCGGGATA
TGTACGTGGACCAGGAACTGGACATCAACCGGCTGTCCGACTACGATGTGGACCATATCGTGCCTCA
GAGCTTTCTGAAGGACGACTCCATCGACAACAAGGTGCTGACCAGAAGCGACAAGAACCGGGGCAA
GAGCGACAACGTGCCCTCCGAAGAGGTCGTGAAGAAGATGAAGAACTACTGGCGGCAGCTGCTGAA
CGCCAAGCTGATTACCCAGAGAAAGTTCGACAATCTGACCAAGGCCGAGAGAGGCGGCCTGAGCGA
ACTGGATAAGGCCGGCTTCATCAAGAGACAGCTGGTGGAAACCCGGCAGATCACAAAGCACGTGGC
ACAGATCCTGGACTCCCGGATGAACACTAAGTACGACGAGAATGACAAGCTGATCCGGGAAGTGAAA
GTGATCACCCTGAAGTCCAAGCTGGTGTCCGATTTCCGGAAGGATTTCCAGTTTTACAAAGTGCGCG
AGATCAACAACTACCACCACGCCCACGACGCCTACCTGAACGCCGTCGTGGGAACCGCCCTGATCA
AAAAGTACCCTAAGCTGGAAAGCGAGTTCGTGTACGGCGACTACAAGGTGTACGACGTGCGGAAGAT
GATCGCCAAGAGCGAGCAGGAAATCGGCAAGGCTACCGCCAAGTACTTCTTCTACAGCAACATCATG
AACTTTTTCAAGACCGAGATTACCCTGGCCAACGGCGAGATCCGGAAGCGGCCTCTGATCGAGACAA
ACGGCGAAACCGGGGAGATCGTGTGGGATAAGGGCCGGGATTTTGCCACCGTGCGGAAAGTGCTG
AGCATGCCCCAAGTGAATATCGTGAAAAAGACCGAGGTGCAGACAGGCGGCTTCAGCAAAGAGTCTA
TCAGGCCCAAGAGGAACAGCGATAAGCTGATCGCCAGAAAGAAGGACTGGGACCCTAAGAAGTACG
GCGGCTTCGTCAGCCCCACCGTGGCCTATTCTGTGCTGGTGGTGGCCAAAGTGGAAAAGGGCAAGT
CCAAGAAACTGAAGAGTGTGAAAGAGCTGCTGGGGATCACCATCATGGAAAGAAGCAGCTTCGAGAA
GAATCCCATCGACTTTCTGGAAGCCAAGGGCTACAAAGAAGTGAAAAAGGACCTGATCATCAAGCTG
CCTAAGTACTCCCTGTTCGAGCTGGAAAACGGCCGGAAGAGAATGCTGGCCTCTGCCAGATTCCTGC
AGAAGGGAAACGAACTGGCCCTGCCCTCCAAATATGTGAACTTCCTGTACCTGGCCAGCCACTATGA
GAAGCTGAAGGGCTCCCCCGAGGATAATGAGCAGAAACAGCTGTTTGTGGAACAGCACAAGCACTA
CCTGGACGAGATCATCGAGCAGATCAGCGAGTTCTCCAAGAGAGTGATCCTGGCCGACGCTAATCTG
GACAAAGTGCTGTCCGCCTACAACAAGCACCGGGATAAGCCCATCAGAGAGCAGGCCGAGAATATCA
TCCACCTGTTTACCCTGACCAATCTGGGAGCCCCTAGGGCCTTCAAGTACTTTGACACCACCATCGAC
CGGAAGGTGTACAGGAGCACCAAAGAGGTGCTGGACGCCACCCTGATCCACCAGAGCATCACCGGC
CTGTACGAGACACGGATCGACCTGTCTCAGCTGGGAGGTGACTCTGGCGGCTCAAAAAGAACCGCC
GACGGCAGCGAATTCGAGCCCAAGAAGAAGAGGAAAGTCTAATTAATTAAGCTGCCTTCTGCGGGGC
TTGCCTTCTGGCCATGCCCTTCTTCTCTCCCTTGCACCTGTACCTCTTGGTCTTTGAATAAAGCCTGA
GTAGGAAGCGACTGTGCCTTCTAGTTGCCAGCCATCTGTTGTTTGCCCCTCCCCCGTGCCTTCCTTG
ACCCTGGAAGGTGCCACTCCCACTGTCCTTTCCTAATAAAATGAGAAATTGCATCGCATTGTCTGAG
TAGGTGTCATTCTATTCTGGGGGGTGGGGTGGGGCAGGACAGCAAGGGGGAGGATTGGGAAGACAA
TAGCAGGCATGCTGGGGATGCGGTGGGCTCTATGG

Start	End	Feature Description
1	380	CMV enhancer
381	619	CMV promoter
620	636	T7 promoter with mismatch
637	683	5' UTR
684	740	BP NLS
741	1238	Engineered TadA* variant 8e(V106W)
1239	1334	(SGGS)2 – XTEN – (SGGS)2 linker
1335	5435	Cas9(D10A) SpNG PAM variant
5436	5501	Linker + SV40 BP NLS

5502	5605	3’ UTR
5606	5830	bGH poly(A) signal

Amino acid sequence

MKRTADGSEFESPKKKRKVSEVEFSHEYWMRHALTLAKRARDEREVPVGAVLVLNNRVIGEGWNRAIGL
HDPTAHAEIMALRQGGLVMQNYRLIDATLYVTFEPCVMCAGAMIHSRIGRVVFGWRNSKRGAAGSLMNV
LNYPGMNHRVEITEGILADECAALLCDFYRMPRQVFNAQKKAQSSINSGGSSGGSSGSETPGTSESATP
ESSGGSSGGSDKKYSIGLAIGTNSVGWAVITDEYKVPSKKFKVLGNTDRHSIKKNLIGALLFDSGETAEAT
RLKRTARRRYTRRKNRICYLQEIFSNEMAKVDDSFFHRLEESFLVEEDKKHERHPIFGNIVDEVAYHEKYP
TIYHLRKKLVDSTDKADLRLIYLALAHMIKFRGHFLIEGDLNPDNSDVDKLFIQLVQTYNQLFEENPINASGV
DAKAILSARLSKSRRLENLIAQLPGEKKNGLFGNLIALSLGLTPNFKSNFDLAEDAKLQLSKDTYDDDLDNL
LAQIGDQYADLFLAAKNLSDAILLSDILRVNTEITKAPLSASMIKRYDEHHQDLTLLKALVRQQLPEKYKEIFF
DQSKNGYAGYIDGGASQEEFYKFIKPILEKMDGTEELLVKLNREDLLRKQRTFDNGSIPHQIHLGELHAILR
RQEDFYPFLKDNREKIEKILTFRIPYYVGPLARGNSRFAWMTRKSEETITPWNFEEVVDKGASAQSFIERM
TNFDKNLPNEKVLPKHSLLYEYFTVYNELTKVKYVTEGMRKPAFLSGEQKKAIVDLLFKTNRKVTVKQLKE
DYFKKIECFDSVEISGVEDRFNASLGTYHDLLKIIKDKDFLDNEENEDILEDIVLTLTLFEDREMIEERLKTYA
HLFDDKVMKQLKRRRYTGWGRLSRKLINGIRDKQSGKTILDFLKSDGFANRNFMQLIHDDSLTFKEDIQKA
QVSGQGDSLHEHIANLAGSPAIKKGILQTVKVVDELVKVMGRHKPENIVIEMARENQTTQKGQKNSRERM
KRIEEGIKELGSQILKEHPVENTQLQNEKLYLYYLQNGRDMYVDQELDINRLSDYDVDHIVPQSFLKDDSID
NKVLTRSDKNRGKSDNVPSEEVVKKMKNYWRQLLNAKLITQRKFDNLTKAERGGLSELDKAGFIKRQLVE
TRQITKHVAQILDSRMNTKYDENDKLIREVKVITLKSKLVSDFRKDFQFYKVREINNYHHAHDAYLNAVVGT
ALIKKYPKLESEFVYGDYKVYDVRKMIAKSEQEIGKATAKYFFYSNIMNFFKTEITLANGEIRKRPLIETNGE
TGEIVWDKGRDFATVRKVLSMPQVNIVKKTEVQTGGFSKESIRPKRNSDKLIARKKDWDPKKYGGFVSPT
VAYSVLVVAKVEKGKSKKLKSVKELLGITIMERSSFEKNPIDFLEAKGYKEVKKDLIIKLPKYSLFELENGRK
RMLASARFLQKGNELALPSKYVNFLYLASHYEKLKGSPEDNEQKQLFVEQHKHYLDEIIEQISEFSKRVILA
DANLDKVLSAYNKHRDKPIREQAENIIHLFTLTNLGAPRAFKYFDTTIDRKVYRSTKEVLDATLIHQSITGLYE
TRIDLSQLGGDSGGSKRTADGSEFEPKKKRKV

Start	End	Feature Description
1	19	BP NLS
20	185	Engineered TadA* variant 8e
186	217	(SGGS)2 – XTEN – (SGGS)2 linker
218	1584	Cas9(D10A) SpNG PAM variant
1585	1605	Linker + SV40 BP NLS

ABE9 – Cas9 (D10A) – SpNG variant
Plasmid ID: pHS0414

DNA sequence

GACATTGATTATTGACTAGTTATTAATAGTAATCAATTACGGGGTCATTAGTTCATAGCCCATATATGGAG
TTCCGCGTTACATAACTTACGGTAAATGGCCCGCCTGGCTGACCGCCCAACGACCCCCGCCCATTGA
CGTCAATAATGACGTATGTTCCCATAGTAACGCCAATAGGGACTTTCCATTGACGTCAATGGGTGGAGT
ATTTACGGTAAACTGCCCACTTGGCAGTACATCAAGTGTATCATATGCCAAGTACGCCCCCTATTGACG
TCAATGACGGTAAATGGCCCGCCTGGCATTATGCCCAGTACATGACCTTATGGGACTTTCCTACTTGG
CAGTACATCTACGTATTAGTCATCGCTATTACCATGGTGATGCGGTTTTGGCAGTACATCAATGGGCGT
GGATAGCGGTTTGACTCACGGGGATTTCCAAGTCTCCACCCCATTGACGTCAATGGGAGTTTGTTTTG
GCACCAAAATCAACGGGACTTTCCAAAATGTCGTAACAACTCCGCCCCATTGACGCAAATGGGCGGT
AGGCGTGTACGGTGGGAGGTCTATATAAGCAGAGCTGGTTTAGTGAACCGTCAGATCTCGAGCTCGG
TACCTAATACGACACACTATAAGGAAATAAGAGAGAAAAGAAGAGTAAGAAGAAATATAAGAGCCACCA
TGAAACGGACAGCCGACGGAAGCGAGTTCGAGTCACCAAAGAAGAAGCGGAAAGTCTCTGAGGTG
GAGTTTTCCCACGAGTACTGGATGAGACATGCCCTGACCCTGGCCAAGAGGGCACGGGATGAGAGG
GAGGTGCCTGTGGGAGCCGTGCTGGTGCTGAACAATAGAGTGATCGGCGAGGGCTGGAACAGAGC
CATCGGCCTGCACGACCCAACAGCCCATGCCGAAATTATGGCCCTGAGACAGGGCGGCCTGGTCAT
GCAGAACTACAGACTGATTGACGCCACCCTGTACGTGACATTCGAGCCTTGCGTGATGTGCGCCGGC
GCCATGATCCACTCTAGGATCGGCCGCGTGGTGTTTGGCGTGAGGCAGTCAAAAAGAGGCGCCGCA
GGCTCCCTGATGAACGTGCTGAACTACCCCGGCATGAATCACCGCGTCGAAATTACCGAGGGAATCC
TGGCAGATGAATGTGCCGCCCTGACCTGCGATTTCTATCGGATGCCTAGACAGGTGTTCAATGCTCAG
AAGAAGGCCCAGAGCTCCATCAACTCCGGAGGATCTAGCGGAGGCTCCTCTGGCTCTGAGACACCT
GGCACAAGCGAGAGCGCAACACCTGAAAGCAGCGGGGGCAGCAGCGGGGGGTCAGACAAGAAGT
ACAGCATCGGCCTGGCCATCGGCACCAACTCTGTGGGCTGGGCCGTGATCACCGACGAGTACAAGG
TGCCCAGCAAGAAATTCAAGGTGCTGGGCAACACCGACCGGCACAGCATCAAGAAGAACCTGATCG
GAGCCCTGCTGTTCGACAGCGGCGAAACAGCCGAGGCCACCCGGCTGAAGAGAACCGCCAGAAGA
AGATACACCAGACGGAAGAACCGGATCTGCTATCTGCAAGAGATCTTCAGCAACGAGATGGCCAAGG
TGGACGACAGCTTCTTCCACAGACTGGAAGAGTCCTTCCTGGTGGAAGAGGATAAGAAGCACGAGC
GGCACCCCATCTTCGGCAACATCGTGGACGAGGTGGCCTACCACGAGAAGTACCCCACCATCTACCA
CCTGAGAAAGAAACTGGTGGACAGCACCGACAAGGCCGACCTGCGGCTGATCTATCTGGCCCTGGC
CCACATGATCAAGTTCCGGGGCCACTTCCTGATCGAGGGCGACCTGAACCCCGACAACAGCGACGT
GGACAAGCTGTTCATCCAGCTGGTGCAGACCTACAACCAGCTGTTCGAGGAAAACCCCATCAACGCC
AGCGGCGTGGACGCCAAGGCCATCCTGTCTGCCAGACTGAGCAAGAGCAGACGGCTGGAAAATCTG
ATCGCCCAGCTGCCCGGCGAGAAGAAGAATGGCCTGTTCGGAAACCTGATTGCCCTGAGCCTGGGC
CTGACCCCCAACTTCAAGAGCAACTTCGACCTGGCCGAGGATGCCAAACTGCAGCTGAGCAAGGAC
ACCTACGACGACGACCTGGACAACCTGCTGGCCCAGATCGGCGACCAGTACGCCGACCTGTTTCTG
GCCGCCAAGAACCTGTCCGACGCCATCCTGCTGAGCGACATCCTGAGAGTGAACACCGAGATCACC
AAGGCCCCCCTGAGCGCCTCTATGATCAAGAGATACGACGAGCACCACCAGGACCTGACCCTGCTG
AAAGCTCTCGTGCGGCAGCAGCTGCCTGAGAAGTACAAAGAGATTTTCTTCGACCAGAGCAAGAACG
GCTACGCCGGCTACATTGACGGCGGAGCCAGCCAGGAAGAGTTCTACAAGTTCATCAAGCCCATCCT
GGAAAAGATGGACGGCACCGAGGAACTGCTCGTGAAGCTGAACAGAGAGGACCTGCTGCGGAAGC
AGCGGACCTTCGACAACGGCAGCATCCCCCACCAGATCCACCTGGGAGAGCTGCACGCCATTCTGC
GGCGGCAGGAAGATTTTTACCCATTCCTGAAGGACAACCGGGAAAAGATCGAGAAGATCCTGACCTT
CCGCATCCCCTACTACGTGGGCCCTCTGGCCAGGGGAAACAGCAGATTCGCCTGGATGACCAGAAA
GAGCGAGGAAACCATCACCCCCTGGAACTTCGAGGAAGTGGTGGACAAGGGCGCTTCCGCCCAGA
GCTTCATCGAGCGGATGACCAACTTCGATAAGAACCTGCCCAACGAGAAGGTGCTGCCCAAGCACA
GCCTGCTGTACGAGTACTTCACCGTGTATAACGAGCTGACCAAAGTGAAATACGTGACCGAGGGAAT
GAGAAAGCCCGCCTTCCTGAGCGGCGAGCAGAAAAAGGCCATCGTGGACCTGCTGTTCAAGACCAA
CCGGAAAGTGACCGTGAAGCAGCTGAAAGAGGACTACTTCAAGAAAATCGAGTGCTTCGACTCCGTG
GAAATCTCCGGCGTGGAAGATCGGTTCAACGCCTCCCTGGGCACATACCACGATCTGCTGAAAATTAT
CAAGGACAAGGACTTCCTGGACAATGAGGAAAACGAGGACATTCTGGAAGATATCGTGCTGACCCTG
ACACTGTTTGAGGACAGAGAGATGATCGAGGAACGGCTGAAAACCTATGCCCACCTGTTCGACGACA
AAGTGATGAAGCAGCTGAAGCGGCGGAGATACACCGGCTGGGGCAGGCTGAGCCGGAAGCTGATC
AACGGCATCCGGGACAAGCAGTCCGGCAAGACAATCCTGGATTTCCTGAAGTCCGACGGCTTCGCC
AACAGAAACTTCATGCAGCTGATCCACGACGACAGCCTGACCTTTAAAGAGGACATCCAGAAAGCCC

AGGTGTCCGGCCAGGGCGATAGCCTGCACGAGCACATTGCCAATCTGGCCGGCAGCCCCGCCATTA
AGAAGGGCATCCTGCAGACAGTGAAGGTGGTGGACGAGCTCGTGAAAGTGATGGGCCGGCACAAG
CCCGAGAACATCGTGATCGAAATGGCCAGAGAGAACCAGACCACCCAGAAGGGACAGAAGAACAGC
CGCGAGAGAATGAAGCGGATCGAAGAGGGCATCAAAGAGCTGGGCAGCCAGATCCTGAAAGAACAC
CCCGTGGAAAACACCCAGCTGCAGAACGAGAAGCTGTACCTGTACTACCTGCAGAATGGGCGGGATA
TGTACGTGGACCAGGAACTGGACATCAACCGGCTGTCCGACTACGATGTGGACCATATCGTGCCTCA
GAGCTTTCTGAAGGACGACTCCATCGACAACAAGGTGCTGACCAGAAGCGACAAGAACCGGGGCAA
GAGCGACAACGTGCCCTCCGAAGAGGTCGTGAAGAAGATGAAGAACTACTGGCGGCAGCTGCTGAA
CGCCAAGCTGATTACCCAGAGAAAGTTCGACAATCTGACCAAGGCCGAGAGAGGCGGCCTGAGCGA
ACTGGATAAGGCCGGCTTCATCAAGAGACAGCTGGTGGAAACCCGGCAGATCACAAAGCACGTGGC
ACAGATCCTGGACTCCCGGATGAACACTAAGTACGACGAGAATGACAAGCTGATCCGGGAAGTGAAA
GTGATCACCCTGAAGTCCAAGCTGGTGTCCGATTTCCGGAAGGATTTCCAGTTTTACAAAGTGCGCG
AGATCAACAACTACCACCACGCCCACGACGCCTACCTGAACGCCGTCGTGGGAACCGCCCTGATCA
AAAAGTACCCTAAGCTGGAAAGCGAGTTCGTGTACGGCGACTACAAGGTGTACGACGTGCGGAAGAT
GATCGCCAAGAGCGAGCAGGAAATCGGCAAGGCTACCGCCAAGTACTTCTTCTACAGCAACATCATG
AACTTTTTCAAGACCGAGATTACCCTGGCCAACGGCGAGATCCGGAAGCGGCCTCTGATCGAGACAA
ACGGCGAAACCGGGGAGATCGTGTGGGATAAGGGCCGGGATTTTGCCACCGTGCGGAAAGTGCTG
AGCATGCCCCAAGTGAATATCGTGAAAAAGACCGAGGTGCAGACAGGCGGCTTCAGCAAAGAGTCTA
TCAGGCCCAAGAGGAACAGCGATAAGCTGATCGCCAGAAAGAAGGACTGGGACCCTAAGAAGTACG
GCGGCTTCGTCAGCCCCACCGTGGCCTATTCTGTGCTGGTGGTGGCCAAAGTGGAAAAGGGCAAGT
CCAAGAAACTGAAGAGTGTGAAAGAGCTGCTGGGGATCACCATCATGGAAAGAAGCAGCTTCGAGAA
GAATCCCATCGACTTTCTGGAAGCCAAGGGCTACAAAGAAGTGAAAAAGGACCTGATCATCAAGCTG
CCTAAGTACTCCCTGTTCGAGCTGGAAAACGGCCGGAAGAGAATGCTGGCCTCTGCCAGATTCCTGC
AGAAGGGAAACGAACTGGCCCTGCCCTCCAAATATGTGAACTTCCTGTACCTGGCCAGCCACTATGA
GAAGCTGAAGGGCTCCCCCGAGGATAATGAGCAGAAACAGCTGTTTGTGGAACAGCACAAGCACTA
CCTGGACGAGATCATCGAGCAGATCAGCGAGTTCTCCAAGAGAGTGATCCTGGCCGACGCTAATCTG
GACAAAGTGCTGTCCGCCTACAACAAGCACCGGGATAAGCCCATCAGAGAGCAGGCCGAGAATATCA
TCCACCTGTTTACCCTGACCAATCTGGGAGCCCCTAGGGCCTTCAAGTACTTTGACACCACCATCGAC
CGGAAGGTGTACAGGAGCACCAAAGAGGTGCTGGACGCCACCCTGATCCACCAGAGCATCACCGGC
CTGTACGAGACACGGATCGACCTGTCTCAGCTGGGAGGTGACTCTGGCGGCTCAAAAAGAACCGCC
GACGGCAGCGAATTCGAGCCCAAGAAGAAGAGGAAAGTCTAATTAATTAAGCTGCCTTCTGCGGGGC
TTGCCTTCTGGCCATGCCCTTCTTCTCTCCCTTGCACCTGTACCTCTTGGTCTTTGAATAAAGCCTGA
GTAGGAAGCGACTGTGCCTTCTAGTTGCCAGCCATCTGTTGTTTGCCCCTCCCCCGTGCCTTCCTTG
ACCCTGGAAGGTGCCACTCCCACTGTCCTTTCCTAATAAAATGAGAAATTGCATCGCATTGTCTGAG
TAGGTGTCATTCTATTCTGGGGGGTGGGGTGGGGCAGGACAGCAAGGGGGAGGATTGGGAAGACAA
TAGCAGGCATGCTGGGGATGCGGTGGGCTCTATGG

Start	End	Feature Description
1	380	CMV enhancer
381	619	CMV promoter
620	636	T7 promoter with mismatch
637	683	5' UTR
684	740	BP NLS
741	1238	Engineered TadA* variant ABE9 (TadA-8e N108Q L145T)
1239	1334	(SGGS)2 – XTEN – (SGGS)2 linker
1335	5435	Cas9(D10A) SpNG PAM variant
5436	5501	Linker + SV40 BP NLS

5502	5605	3' UTR
5606	5830	bGH poly(A) signal

<u>Amino acid sequence</u>
MKRTADGSEFESPKKKRKVSEVEFSHEYWMRHALTLAKRARDEREVPVGAVLVLNNRVIGEGWNRAIGL
HDPTAHAEIMALRQGGLVMQNYRLIDATLYVTFEPCVMCAGAMIHSRIGRVVFGVRQSKRGAAGSLMNVL
NYPGMNHRVEITEGILADECAALTCDFYRMPRQVFNAQKKAQSSINSGGSSGGSSGSETPGTSESATPE
SSGGSSGGSDKKYSIGLAIGTNSVGWAVITDEYKVPSKKFKVLGNTDRHSIKKNLIGALLFDSGETAEATRL
KRTARRRYTRRKNRICYLQEIFSNEMAKVDDSFFHRLEESFLVEEDKKHERHPIFGNIVDEVAYHEKYPTIY
HLRKKLVDSTDKADLRLIYLALAHMIKFRGHFLIEGDLNPDNSDVDKLFIQLVQTYNQLFEENPINASGVDA
KAILSARLSKSRRLENLIAQLPGEKKNGLFGNLIALSLGLTPNFKSNFDLAEDAKLQLSKDTYDDDLDNLLA
QIGDQYADLFLAAKNLSDAILLSDILRVNTEITKAPLSASMIKRYDEHHQDLTLLKALVRQQLPEKYKEIFFD
QSKNGYAGYIDGGASQEEFYKFIKPILEKMDGTEELLVKLNREDLLRKQRTFDNGSIPHQIHLGELHAILRR
QEDFYPFLKDNREKIEKILTFRIPYYVGPLARGNSRFAWMTRKSEETITPWNFEEVVDKGASAQSFIERMT
NFDKNLPNEKVLPKHSLLYEYFTVYNELTKVKYVTEGMRKPAFLSGEQKKAIVDLLFKTNRKVTVKQLKED
YFKKIECFDSVEISGVEDRFNASLGTYHDLLKIIKDKDFLDNEENEDILEDIVLTLTLFEDREMIEERLKTYAH
LFDDKVMKQLKRRRYTGWGRLSRKLINGIRDKQSGKTILDFLKSDGFANRNFMQLIHDDSLTFKEDIQKAQ
VSGQGDSLHEHIANLAGSPAIKKGILQTVKVVDELVKVMGRHKPENIVIEMARENQTTQKGQKNSRERMK
RIEEGIKELGSQILKEHPVENTQLQNEKLYLYYLQNGRDMYVDQELDINRLSDYDVDHIVPQSFLKDDSIDN
KVLTRSDKNRGKSDNVPSEEVVKKMKNYWRQLLNAKLITQRKFDNLTKAERGGLSELDKAGFIKRQLVET
RQITKHVAQILDSRMNTKYDENDKLIREVKVITLKSKLVSDFRKDFQFYKVREINNYHHAHDAYLNAVVGTA
LIKKYPKLESEFVYGDYKVYDVRKMIAKSEQEIGKATAKYFFYSNIMNFFKTEITLANGEIRKRPLIETNGET
GEIVWDKGRDFATVRKVLSMPQVNIVKKTEVQTGGFSKESIRPKRNSDKLIARKKDWDPKKYGGFVSPTV
AYSVLVVAKVEKGKSKKLKSVKELLGITIMERSSFEKNPIDFLEAKGYKEVKKDLIIKLPKYSLFELENGRKR
MLASARFLQKGNELALPSKYVNFLYLASHYEKLKGSPEDNEQKQLFVEQHKHYLDEIIEQISEFSKRVILAD
ANLDKVLSAYNKHRDKPIREQAENIIHLFTLTNLGAPRAFKYFDTTIDRKVYRSTKEVLDATLIHQSITGLYET
RIDLSQLGGDSGGSKRTADGSEFEPKKKRKV

Start	End	Feature Description
1	19	BP NLS
20	185	Engineered TadA* variant 8e
186	217	(SGGS)2 – XTEN – (SGGS)2 linker
218	1584	Cas9(D10A) SpNG PAM variant
1585	1605	Linker + SV40 BP NLS

ABE7.10 – Cas9 (D10A) – VRQR variant
Plasmid ID: pHS0382

DNA sequence

GACATTGATTATTGACTAGTTATTAATAGTAATCAATTACGGGGTCATTAGTTCATAGCCCATATATGGAG
TTCCGCGTTACATAACTTACGGTAAATGGCCCGCCTGGCTGACCGCCCAACGACCCCCGCCCATTGA
CGTCAATAATGACGTATGTTCCCATAGTAACGCCAATAGGGACTTTCCATTGACGTCAATGGGTGGAGT
ATTTACGGTAAACTGCCCACTTGGCAGTACATCAAGTGTATCATATGCCAAGTACGCCCCCTATTGACG
TCAATGACGGTAAATGGCCCGCCTGGCATTATGCCCAGTACATGACCTTATGGGACTTTCCTACTTGG
CAGTACATCTACGTATTAGTCATCGCTATTACCATGGTGATGCGGTTTTGGCAGTACATCAATGGGCGT
GGATAGCGGTTTGACTCACGGGGATTTCCAAGTCTCCACCCCATTGACGTCAATGGGAGTTTGTTTTG
GCACCAAAATCAACGGGACTTTCCAAAATGTCGTAACAACTCCGCCCCATTGACGCAAATGGGCGGT
AGGCGTGTACGGTGGGAGGTCTATATAAGCAGAGCTGGTTTAGTGAACCGTCAGATCCGCTAGAGAT
CCGCGGCCGCTAATACGACTCACTATAGGGAGAGCCGCCACCATGAAACGGACAGCCGACGGAAGC
GAGTTCGAGTCACCAAAGAAGAAGCGGAAAGTCTCTGAAGTCGAGTTTAGCCACGAGTATTGGATGA
GGCACGCACTGACCCTGGCAAAGCGAGCATGGGATGAAAGAGAAGTCCCCGTGGGCGCCGTGCTG
GTGCACAACAATAGAGTGATCGGAGAGGGATGGAACAGGCCAATCGGCCGCCACGACCCTACCGCA
CACGCAGAGATCATGGCACTGAGGCAGGGAGGCCTGGTCATGCAGAATTACCGCCTGATCGATGCC
ACCCTGTATGTGACACTGGAGCCATGCGTGATGTGCGCAGGAGCAATGATCCACAGCAGGATCGGAA
GAGTGGTGTTCGGAGCACGGGACGCCAAGACCGGCGCAGCAGGCTCCCTGATGGATGTGCTGCAC
CACCCCGGCATGAACCACCGGGTGGAGATCACAGAGGGAATCCTGGCAGACGAGTGCGCCGCCCT
GCTGAGCGATTTCTTTAGAATGCGGAGACAGGAGATCAAGGCCCAGAAGAAGGCACAGAGCTCCAC
CGACTCTGGAGGATCTAGCGGAGGATCCTCTGGAAGCGAGACACCAGGCACAAGCGAGTCCGCCAC
ACCAGAGAGCTCCGGCGGCTCCTCCGGAGGATCCTCTGAGGTGGAGTTTTCCCACGAGTACTGGAT
GAGACATGCCCTGACCCTGGCCAAGAGGGCACGCGATGAGAGGGAGGTGCCTGTGGGAGCCGTGC
TGGTGCTGAACAATAGAGTGATCGGCGAGGGCTGGAACAGAGCCATCGGCCTGCACGACCCAACAG
CCCATGCCGAAATTATGGCCCTGAGACAGGGCGGCCTGGTCATGCAGAACTACAGACTGATTGACGC
CACCCTGTACGTGACATTCGAGCCTTGCGTGATGTGCGCCGGCGCCATGATCCACTCTAGGATCGGC
CGCGTGGTGTTTGGCGTGAGGAACGCAAAAACCGGCGCCGCAGGCTCCCTGATGGACGTGCTGCA
CTACCCCGGCATGAATCACCGCGTCGAAATTACCGAGGGAATCCTGGCAGATGAATGTGCCGCCCTG
CTGTGCTATTTCTTTCGGATGCCTAGACAGGTGTTCAATGCTCAGAAGAAGGCCCAGAGCTCCACCG
ACTCCGGAGGATCTAGCGGAGGCTCCTCTGGCTCTGAGACACCTGGCACAAGCGAGAGCGCAACAC
CTGAAAGCAGCGGGGGCAGCAGCGGGGGGTCAGACAAGAAGTACAGCATCGGCCTGGCCATCGGC
ACCAACTCTGTGGGCTGGGCCGTGATCACCGACGAGTACAAGGTGCCCAGCAAGAAATTCAAGGTG
CTGGGCAACACCGACCGGCACAGCATCAAGAAGAACCTGATCGGAGCCCTGCTGTTCGACAGCGGC
GAAACAGCCGAGGCCACCCGGCTGAAGAGAACCGCCAGAAGAAGATACACCAGACGGAAGAACCG
GATCTGCTATCTGCAAGAGATCTTCAGCAACGAGATGGCCAAGGTGGACGACAGCTTCTTCCACAGA
CTGGAAGAGTCCTTCCTGGTGGAAGAGGATAAGAAGCACGAGCGGCACCCCATCTTCGGCAACATC
GTGGACGAGGTGGCCTACCACGAGAAGTACCCCACCATCTACCACCTGAGAAAGAAACTGGTGGAC
AGCACCGACAAGGCCGACCTGCGGCTGATCTATCTGGCCCTGGCCCACATGATCAAGTTCCGGGGC
CACTTCCTGATCGAGGGCGACCTGAACCCCGACAACAGCGACGTGGACAAGCTGTTCATCCAGCTG
GTGCAGACCTACAACCAGCTGTTCGAGGAAAACCCCATCAACGCCAGCGGCGTGGACGCCAAGGCC
ATCCTGTCTGCCAGACTGAGCAAGAGCAGACGGCTGGAAAATCTGATCGCCCAGCTGCCCGGCGAG
AAGAAGAATGGCCTGTTCGGAAACCTGATTGCCCTGAGCCTGGGCCTGACCCCCAACTTCAAGAGC
AACTTCGACCTGGCCGAGGATGCCAAACTGCAGCTGAGCAAGGACACCTACGACGACGACCTGGAC
AACCTGCTGGCCCAGATCGGCGACCAGTACGCCGACCTGTTTCTGGCCGCCAAGAACCTGTCCGAC
GCCATCCTGCTGAGCGACATCCTGAGAGTGAACACCGAGATCACCAAGGCCCCCCTGAGCGCCTCT
ATGATCAAGAGATACGACGAGCACCACCAGGACCTGACCCTGCTGAAAGCTCTCGTGCGGCAGCAG
CTGCCTGAGAAGTACAAAGAGATTTTCTTCGACCAGAGCAAGAACGGCTACGCCGGCTACATTGACG
GCGGAGCCAGCCAGGAAGAGTTCTACAAGTTCATCAAGCCCATCCTGGAAAAGATGGACGGCACCG
AGGAACTGCTCGTGAAGCTGAACAGAGAGGACCTGCTGCGGAAGCAGCGGACCTTCGACAACGGC
AGCATCCCCCACCAGATCCACCTGGGAGAGCTGCACGCCATTCTGCGGCGGCAGGAAGATTTTTACC
CATTCCTGAAGGACAACCGGGAAAAGATCGAGAAGATCCTGACCTTCCGCATCCCCTACTACGTGGG
CCCTCTGGCCAGGGGAAACAGCAGATTCGCCTGGATGACCAGAAAGAGCGAGGAAACCATCACCCC
CTGGAACTTCGAGGAAGTGGTGGACAAGGGCGCTTCCGCCCAGAGCTTCATCGAGCGGATGACCAA
CTTCGATAAGAACCTGCCCAACGAGAAGGTGCTGCCCAAGCACAGCCTGCTGTACGAGTACTTCACC

GTGTATAACGAGCTGACCAAAGTGAAATACGTGACCGAGGGAATGAGAAAGCCCGCCTTCCTGAGCG
GCGAGCAGAAAAAGGCCATCGTGGACCTGCTGTTCAAGACCAACCGGAAAGTGACCGTGAAGCAGC
TGAAAGAGGACTACTTCAAGAAAATCGAGTGCTTCGACTCCGTGGAAATCTCCGGCGTGGAAGATCG
GTTCAACGCCTCCCTGGGCACATACCACGATCTGCTGAAAATTATCAAGGACAAGGACTTCCTGGACA
ATGAGGAAAACGAGGACATTCTGGAAGATATCGTGCTGACCCTGACACTGTTTGAGGACAGAGAGAT
GATCGAGGAACGGCTGAAAACCTATGCCCACCTGTTCGACGACAAAGTGATGAAGCAGCTGAAGCG
GCGGAGATACACCGGCTGGGGCAGGCTGAGCCGGAAGCTGATCAACGGCATCCGGGACAAGCAGT
CCGGCAAGACAATCCTGGATTTCCTGAAGTCCGACGGCTTCGCCAACAGAAACTTCATGCAGCTGAT
CCACGACGACAGCCTGACCTTTAAAGAGGACATCCAGAAAGCCCAGGTGTCCGGCCAGGGCGATAG
CCTGCACGAGCACATTGCCAATCTGGCCGGCAGCCCCGCCATTAAGAAGGGCATCCTGCAGACAGT
GAAGGTGGTGGACGAGCTCGTGAAAGTGATGGGCCGGCACAAGCCCGAGAACATCGTGATCGAAAT
GGCCAGAGAGAACCAGACCACCCAGAAGGGACAGAAGAACAGCCGCGAGAGAATGAAGCGGATCG
AAGAGGGCATCAAAGAGCTGGGCAGCCAGATCCTGAAAGAACACCCCGTGGAAAACACCCAGCTGC
AGAACGAGAAGCTGTACCTGTACTACCTGCAGAATGGGCGGGATATGTACGTGGACCAGGAACTGGA
CATCAACCGGCTGTCCGACTACGATGTGGACCATATCGTGCCTCAGAGCTTTCTGAAGGACGACTCC
ATCGACAACAAGGTGCTGACCAGAAGCGACAAGAACCGGGGCAAGAGCGACAACGTGCCCTCCGA
AGAGGTCGTGAAGAAGATGAAGAACTACTGGCGGCAGCTGCTGAACGCCAAGCTGATTACCCAGAG
AAAGTTCGACAATCTGACCAAGGCCGAGAGAGGCGGCCTGAGCGAACTGGATAAGGCCGGCTTCAT
CAAGAGACAGCTGGTGGAAACCCGGCAGATCACAAAGCACGTGGCACAGATCCTGGACTCCCGGAT
GAACACTAAGTACGACGAGAATGACAAGCTGATCCGGGAAGTGAAAGTGATCACCCTGAAGTCCAAG
CTGGTGTCCGATTTCCGGAAGGATTTCCAGTTTTACAAAGTGCGCGAGATCAACAACTACCACCACGC
CCACGACGCCTACCTGAACGCCGTCGTGGGAACCGCCCTGATCAAAAAGTACCCTAAGCTGGAAAG
CGAGTTCGTGTACGGCGACTACAAGGTGTACGACGTGCGGAAGATGATCGCCAAGAGCGAGCAGGA
AATCGGCAAGGCTACCGCCAAGTACTTCTTCTACAGCAACATCATGAACTTTTTCAAGACCGAGATTAC
CCTGGCCAACGGCGAGATCCGGAAGCGGCCTCTGATCGAGACAAACGGCGAAACCGGGGAGATCG
TGTGGGATAAGGGCCGGGATTTTGCCACCGTGCGGAAAGTGCTGAGCATGCCCCAAGTGAATATCGT
GAAAAAGACCGAGGTGCAGACAGGCGGCTTCAGCAAAGAGTCTATCCTGCCCAAGAGGAACAGCGA
TAAGCTGATCGCCAGAAAGAAGGACTGGGACCCTAAGAAGTACGGCGGCTTCGTGAGCCCCACCGT
GGCCTATTCTGTGCTGGTGGTGGCCAAAGTGGAAAAGGGCAAGTCCAAGAAACTGAAGAGTGTGAA
AGAGCTGCTGGGGATCACCATCATGGAAAGAAGCAGCTTCGAGAAGAATCCCATCGACTTTCTGGAA
GCCAAGGGCTACAAAGAAGTGAAAAAGGACCTGATCATCAAGCTGCCTAAGTACTCCCTGTTCGAGC
TGGAAAACGGCCGGAAGAGAATGCTGGCCTCAGCCAGAGAACTGCAGAAGGGAAACGAACTGGCC
CTGCCCTCCAAATATGTGAACTTCCTGTACCTGGCCAGCCACTATGAGAAGCTGAAGGGCTCCCCCG
AGGATAATGAGCAGAAACAGCTGTTTGTGGAACAGCACAAGCACTACCTGGACGAGATCATCGAGCA
GATCAGCGAGTTCTCCAAGAGAGTGATCCTGGCCGACGCTAATCTGGACAAAGTGCTGTCCGCCTAC
AACAAGCACCGGGATAAGCCCATCAGAGAGCAGGCCGAGAATATCATCCACCTGTTTACCCTGACCA
ATCTGGGAGCCCCTGCCGCCTTCAAGTACTTTGACACCACCATCGACCGGAAGCAGTACAGAAGCAC
CAAAGAGGTGCTGGACGCCACCCTGATCCACCAGAGCATCACCGGCCTGTACGAGACACGGATCGA
CCTGTCTCAGCTGGGAGGTGACTCTGGCGGCTCAAAAAGAACCGCCGACGGCAGCGAATTCGAGC
CCAAGAAGAAGAGGAAAGTCTAACCGGTCATCATCACCATCACCATTGAGTTTAAACCCGCTGATCAG
CCTCGACTGTGCCTTCTAGTTGCCAGCCATCTGTTGTTTGCCCCTCCCCCGTGCCTTCCTTGACCCT
GGAAGGTGCCACTCCCACTGTCCTTTCCTAATAAAATGAGGAAATTGCATCGCATTGTCTGAGTAGGT
GTCATTCTATTCTGGGGGGTGGGGTGGGGCAGGACAGCAAGGGGGAGGATTGGGAAGACAATAGCA
GGCATGCTGGGGATGCGGTGGGCTCTATGG

Start	End	Feature Description
1	380	CMV enhancer
381	625	CMV promoter
626	642	T7 promoter
643	657	5' UTR
658	714	BP NLS

715	1212	TadA wild type
1213	1308	(SGGS)2 – XTEN – (SGGS)2 linker
1309	1806	Engineered TadA* variant 7.10
1807	1902	(SGGS)2 – XTEN – (SGGS)2 linker
1903	6003	Cas9(D10A) Sp-VRQR PAM variant
6004	6069	Linker + SV40 BP NLS
6069	6117	3' UTR
6118	6345	bGH poly(A) signal

Amino acid sequence
MKRTADGSEFESPKKKRKVSEVEFSHEYWMRHALTLAKRAWDEREVPVGAVLVHNNRVIGEGWNRPIG
RHDPTAHAEIMALRQGGLVMQNYRLIDATLYVTLEPCVMCAGAMIHSRIGRVVFGARDAKTGAAGSLMDV
LHHPGMNHRVEITEGILADECAALLSDFFRMRRQEIKAQKKAQSSTDSGGSSGGSSGSETPGTSESATP
ESSGGSSGGSSEVEFSHEYWMRHALTLAKRARDEREVPVGAVLVLNNRVIGEGWNRAIGLHDPTAHAEI
MALRQGGLVMQNYRLIDATLYVTFEPCVMCAGAMIHSRIGRVVFGVRNAKTGAAGSLMDVLHYPGMNHR
VEITEGILADECAALLCYFFRMPRQVFNAQKKAQSSTDSGGSSGGSSGSETPGTSESATPESSGGSSGG
SDKKYSIGLAIGTNSVGWAVITDEYKVPSKKFKVLGNTDRHSIKKNLIGALLFDSGETAEATRLKRTARRRY
TRRKNRICYLQEIFSNEMAKVDDSFFHRLEESFLVEEDKKHERHPIFGNIVDEVAYHEKYPTIYHLRKKLVD
STDKADLRLIYLALAHMIKFRGHFLIEGDLNPDNSDVDKLFIQLVQTYNQLFEENPINASGVDAKAILSARLS
KSRRLENLIAQLPGEKKNGLFGNLIALSLGLTPNFKSNFDLAEDAKLQLSKDTYDDDLDNLLAQIGDQYADL
FLAAKNLSDAILLSDILRVNTEITKAPLSASMIKRYDEHHQDLTLLKALVRQQLPEKYKEIFFDQSKNGYAGYI
DGGASQEEFYKFIKPILEKMDGTEELLVKLNREDLLRKQRTFDNGSIPHQIHLGELHAILRRQEDFYPFLKD
NREKIEKILTFRIPYYVGPLARGNSRFAWMTRKSEETITPWNFEEVVDKGASAQSFIERMTNFDKNLPNEK
VLPKHSLLYEYFTVYNELTKVKYVTEGMRKPAFLSGEQKKAIVDLLFKTNRKVTVKQLKEDYFKKIECFDSV
EISGVEDRFNASLGTYHDLLKIIKDKDFLDNEENEDILEDIVLTLTLFEDREMIEERLKTYAHLFDDKVMKQLK
RRRYTGWGRLSRKLINGIRDKQSGKTILDFLKSDGFANRNFMQLIHDDSLTFKEDIQKAQVSGQGDSLHE
HIANLAGSPAIKKGILQTVKVVDELVKVMGRHKPENIVIEMARENQTTQKGQKNSRERMKRIEEGIKELGS
QILKEHPVENTQLQNEKLYLYYLQNGRDMYVDQELDINRLSDYDVDHIVPQSFLKDDSIDNKVLTRSDKNR
GKSDNVPSEEVVKKMKNYWRQLLNAKLITQRKFDNLTKAERGGLSELDKAGFIKRQLVETRQITKHVAQIL
DSRMNTKYDENDKLIREVKVITLKSKLVSDFRKDFQFYKVREINNYHHAHDAYLNAVVGTALIKKYPKLESE
FVYGDYKVYDVRKMIAKSEQEIGKATAKYFFYSNIMNFFKTEITLANGEIRKRPLIETNGETGEIVWDKGRD
FATVRKVLSMPQVNIVKKTEVQTGGFSKESILPKRNSDKLIARKKDWDPKKYGGFVSPTVAYSVLVVAKVE
KGKSKKLKSVKELLGITIMERSSFEKNPIDFLEAKGYKEVKKDLIIKLPKYSLFELENGRKRMLASARELQK
GNELALPSKYVNFLYLASHYEKLKGSPEDNEQKQLFVEQHKHYLDEIIEQISEFSKRVILADANLDKVLSAY
NKHRDKPIREQAENIIHLFTLTNLGAPAAFKYFDTTIDRKQYRSTKEVLDATLIHQSITGLYETRIDLSQLGGD
SGGSKRTADGSEFEPKKKRKV

Start	End	Feature Description
1	19	BP NLS
20	185	TadA wild type
186	217	(SGGS)2 – XTEN – (SGGS)2 linker
218	383	Engineered TadA* variant 7.10
384	415	(SGGS)2 – XTEN – (SGGS)2 linker

416	1782	Cas9(D10A) Sp-VRQR PAM variant
1783	1803	Linker + SV40 BP NLS

ABE8e – Cas9 (D10A) – VRQR variant
Plasmid ID: pHS0380

DNA sequence
GACATTGATTATTGACTAGTTATTAATAGTAATCAATTACGGGGTCATTAGTTCATAGCCCATATATGGAG
TTCCGCGTTACATAACTTACGGTAAATGGCCCGCCTGGCTGACCGCCCAACGACCCCCGCCCATTGA
CGTCAATAATGACGTATGTTCCCATAGTAACGCCAATAGGGACTTTCCATTGACGTCAATGGGTGGAGT
ATTTACGGTAAACTGCCCACTTGGCAGTACATCAAGTGTATCATATGCCAAGTACGCCCCCTATTGACG
TCAATGACGGTAAATGGCCCGCCTGGCATTATGCCCAGTACATGACCTTATGGGACTTTCCTACTTGG
CAGTACATCTACGTATTAGTCATCGCTATTACCATGGTGATGCGGTTTTGGCAGTACATCAATGGGCGT
GGATAGCGGTTTGACTCACGGGGATTTCCAAGTCTCCACCCCATTGACGTCAATGGGAGTTTGTTTTG
GCACCAAAATCAACGGGACTTTCCAAAATGTCGTAACAACTCCGCCCCATTGACGCAAATGGGCGGT
AGGCGTGTACGGTGGGAGGTCTATATAAGCAGAGCTGGTTTAGTGAACCGTCAGATCTCGAGCTCGG
TACCTAATACGACACACTATAAGGAAATAAGAGAGAAAAGAAGAGTAAGAAGAAATATAAGAGCCACCA
TGAAACGGACAGCCGACGGAAGCGAGTTCGAGTCACCAAAGAAGAAGCGGAAAGTCTCTGAGGTG
GAGTTTTCCCACGAGTACTGGATGAGACATGCCCTGACCCTGGCCAAGAGGGCACGGGATGAGAGG
GAGGTGCCTGTGGGAGCCGTGCTGGTGCTGAACAATAGAGTGATCGGCGAGGGCTGGAACAGAGC
CATCGGCCTGCACGACCCAACAGCCCATGCCGAAATTATGGCCCTGAGACAGGGCGGCCTGGTCAT
GCAGAACTACAGACTGATTGACGCCACCCTGTACGTGACATTCGAGCCTTGCGTGATGTGCGCCGGC
GCCATGATCCACTCTAGGATCGGCCGCGTGGTGTTTGGCGTGAGGAACTCAAAAAGAGGCGCCGCA
GGCTCCCTGATGAACGTGCTGAACTACCCCGGCATGAATCACCGCGTCGAAATTACCGAGGGAATCC
TGGCAGATGAATGTGCCGCCCTGCTGTGCGATTTCTATCGGATGCCTAGACAGGTGTTCAATGCTCAG
AAGAAGGCCCAGAGCTCCATCAACTCCGGAGGATCTAGCGGAGGCTCCTCTGGCTCTGAGACACCT
GGCACAAGCGAGAGCGCAACACCTGAAAGCAGCGGGGGCAGCAGCGGGGGGTCAGACAAGAAGT
ACAGCATCGGCCTGGCCATCGGCACCAACTCTGTGGGCTGGGCCGTGATCACCGACGAGTACAAGG
TGCCCAGCAAGAAATTCAAGGTGCTGGGCAACACCGACCGGCACAGCATCAAGAAGAACCTGATCG
GAGCCCTGCTGTTCGACAGCGGCGAAACAGCCGAGGCCACCCGGCTGAAGAGAACCGCCAGAAGA
AGATACACCAGACGGAAGAACCGGATCTGCTATCTGCAAGAGATCTTCAGCAACGAGATGGCCAAGG
TGGACGACAGCTTCTTCCACAGACTGGAAGAGTCCTTCCTGGTGGAAGAGGATAAGAAGCACGAGC
GGCACCCCATCTTCGGCAACATCGTGGACGAGGTGGCCTACCACGAGAAGTACCCCACCATCTACCA
CCTGAGAAAGAAACTGGTGGACAGCACCGACAAGGCCGACCTGCGGCTGATCTATCTGGCCCTGGC
CCACATGATCAAGTTCCGGGGCCACTTCCTGATCGAGGGCGACCTGAACCCCGACAACAGCGACGT
GGACAAGCTGTTCATCCAGCTGGTGCAGACCTACAACCAGCTGTTCGAGGAAAACCCCATCAACGCC
AGCGGCGTGGACGCCAAGGCCATCCTGTCTGCCAGACTGAGCAAGAGCAGACGGCTGGAAAATCTG
ATCGCCCAGCTGCCCGGCGAGAAGAAGAATGGCCTGTTCGGAAACCTGATTGCCCTGAGCCTGGGC
CTGACCCCCAACTTCAAGAGCAACTTCGACCTGGCCGAGGATGCCAAACTGCAGCTGAGCAAGGAC
ACCTACGACGACGACCTGGACAACCTGCTGGCCCAGATCGGCGACCAGTACGCCGACCTGTTTCTG
GCCGCCAAGAACCTGTCCGACGCCATCCTGCTGAGCGACATCCTGAGAGTGAACACCGAGATCACC
AAGGCCCCCCTGAGCGCCTCTATGATCAAGAGATACGACGAGCACCACCAGGACCTGACCCTGCTG
AAAGCTCTCGTGCGGCAGCAGCTGCCTGAGAAGTACAAAGAGATTTTCTTCGACCAGAGCAAGAACG
GCTACGCCGGCTACATTGACGGCGGAGCCAGCCAGGAAGAGTTCTACAAGTTCATCAAGCCCATCCT
GGAAAAGATGGACGGCACCGAGGAACTGCTCGTGAAGCTGAACAGAGAGGACCTGCTGCGGAAGC
AGCGGACCTTCGACAACGGCAGCATCCCCCACCAGATCCACCTGGGAGAGCTGCACGCCATTCTGC
GGCGGCAGGAAGATTTTTACCCATTCCTGAAGGACAACCGGGAAAAGATCGAGAAGATCCTGACCTT
CCGCATCCCCTACTACGTGGGCCCTCTGGCCAGGGGAAACAGCAGATTCGCCTGGATGACCAGAAA
GAGCGAGGAAACCATCACCCCCTGGAACTTCGAGGAAGTGGTGGACAAGGGCGCTTCCGCCCAGA
GCTTCATCGAGCGGATGACCAACTTCGATAAGAACCTGCCCAACGAGAAGGTGCTGCCCAAGCACA
GCCTGCTGTACGAGTACTTCACCGTGTATAACGAGCTGACCAAAGTGAAATACGTGACCGAGGGAAT
GAGAAAGCCCGCCTTCCTGAGCGGCGAGCAGAAAAAGGCCATCGTGGACCTGCTGTTCAAGACCAA
CCGGAAAGTGACCGTGAAGCAGCTGAAAGAGGACTACTTCAAGAAAATCGAGTGCTTCGACTCCGTG
GAAATCTCCGGCGTGGAAGATCGGTTCAACGCCTCCCTGGGCACATACCACGATCTGCTGAAAATTAT
CAAGGACAAGGACTTCCTGGACAATGAGGAAAACGAGGACATTCTGGAAGATATCGTGCTGACCCTG
ACACTGTTTGAGGACAGAGAGATGATCGAGGAACGGCTGAAAACCTATGCCCACCTGTTCGACGACA
AAGTGATGAAGCAGCTGAAGCGGCGGAGATACACCGGCTGGGGCAGGCTGAGCCGGAAGCTGATC
AACGGCATCCGGGACAAGCAGTCCGGCAAGACAATCCTGGATTTCCTGAAGTCCGACGGCTTCGCC
AACAGAAACTTCATGCAGCTGATCCACGACGACAGCCTGACCTTTAAAGAGGACATCCAGAAAGCCC

AGGTGTCCGGCCAGGGCGATAGCCTGCACGAGCACATTGCCAATCTGGCCGGCAGCCCCGCCATTA
AGAAGGGCATCCTGCAGACAGTGAAGGTGGTGGACGAGCTCGTGAAAGTGATGGGCCGGCACAAG
CCCGAGAACATCGTGATCGAAATGGCCAGAGAGAACCAGACCACCCAGAAGGGACAGAAGAACAGC
CGCGAGAGAATGAAGCGGATCGAAGAGGGCATCAAAGAGCTGGGCAGCCAGATCCTGAAAGAACAC
CCCGTGGAAAACACCCAGCTGCAGAACGAGAAGCTGTACCTGTACTACCTGCAGAATGGGCGGGATA
TGTACGTGGACCAGGAACTGGACATCAACCGGCTGTCCGACTACGATGTGGACCATATCGTGCCTCA
GAGCTTTCTGAAGGACGACTCCATCGACAACAAGGTGCTGACCAGAAGCGACAAGAACCGGGGCAA
GAGCGACAACGTGCCCTCCGAAGAGGTCGTGAAGAAGATGAAGAACTACTGGCGGCAGCTGCTGAA
CGCCAAGCTGATTACCCAGAGAAAGTTCGACAATCTGACCAAGGCCGAGAGAGGCGGCCTGAGCGA
ACTGGATAAGGCCGGCTTCATCAAGAGACAGCTGGTGGAAACCCGGCAGATCACAAAGCACGTGGC
ACAGATCCTGGACTCCCGGATGAACACTAAGTACGACGAGAATGACAAGCTGATCCGGGAAGTGAAA
GTGATCACCCTGAAGTCCAAGCTGGTGTCCGATTTCCGGAAGGATTTCCAGTTTTACAAAGTGCGCG
AGATCAACAACTACCACCACGCCCACGACGCCTACCTGAACGCCGTCGTGGGAACCGCCCTGATCA
AAAAGTACCCTAAGCTGGAAAGCGAGTTCGTGTACGGCGACTACAAGGTGTACGACGTGCGGAAGAT
GATCGCCAAGAGCGAGCAGGAAATCGGCAAGGCTACCGCCAAGTACTTCTTCTACAGCAACATCATG
AACTTTTTCAAGACCGAGATTACCCTGGCCAACGGCGAGATCCGGAAGCGGCCTCTGATCGAGACAA
ACGGCGAAACCGGGGAGATCGTGTGGGATAAGGGCCGGGATTTTGCCACCGTGCGGAAAGTGCTG
AGCATGCCCCAAGTGAATATCGTGAAAAAGACCGAGGTGCAGACAGGCGGCTTCAGCAAAGAGTCTA
TCCTGCCCAAGAGGAACAGCGATAAGCTGATCGCCAGAAAGAAGGACTGGGACCCTAAGAAGTACG
GCGGCTTCGTGAGCCCCACCGTGGCCTATTCTGTGCTGGTGGTGGCCAAAGTGGAAAAGGGCAAGT
CCAAGAAACTGAAGAGTGTGAAAGAGCTGCTGGGGATCACCATCATGGAAAGAAGCAGCTTCGAGAA
GAATCCCATCGACTTTCTGGAAGCCAAGGGCTACAAAGAAGTGAAAAAGGACCTGATCATCAAGCTG
CCTAAGTACTCCCTGTTCGAGCTGGAAAACGGCCGGAAGAGAATGCTGGCCTCAGCCAGAGAACTG
CAGAAGGGAAACGAACTGGCCCTGCCCTCCAAATATGTGAACTTCCTGTACCTGGCCAGCCACTATG
AGAAGCTGAAGGGCTCCCCCGAGGATAATGAGCAGAAACAGCTGTTTGTGGAACAGCACAAGCACTA
CCTGGACGAGATCATCGAGCAGATCAGCGAGTTCTCCAAGAGAGTGATCCTGGCCGACGCTAATCTG
GACAAAGTGCTGTCCGCCTACAACAAGCACCGGGATAAGCCCATCAGAGAGCAGGCCGAGAATATCA
TCCACCTGTTTACCCTGACCAATCTGGGAGCCCCTGCCGCCTTCAAGTACTTTGACACCACCATCGA
CCGGAAGCAGTACAGAAGCACCAAAGAGGTGCTGGACGCCACCCTGATCCACCAGAGCATCACCGG
CCTGTACGAGACACGGATCGACCTGTCTCAGCTGGGAGGTGACTCTGGCGGCTCAAAAAGAACCGC
CGACGGCAGCGAATTCGAGCCCAAGAAGAAGAGGAAAGTCTAATTAATTAAGCTGCCTTCTGCGGGG
CTTGCCTTCTGGCCATGCCCTTCTTCTCTCCCTTGCACCTGTACCTCTTGGTCTTTGAATAAAGCCTG
AGTAGGAAGCGACTGTGCCTTCTAGTTGCCAGCCATCTGTTGTTTGCCCCTCCCCCGTGCCTTCCTT
GACCCTGGAAGGTGCCACTCCCACTGTCCTTTCCTAATAAAATGAGGAAATTGCATCGCATTGTCTGA
GTAGGTGTCATTCTATTCTGGGGGGTGGGGTGGGGCAGGACAGCAAGGGGGAGGATTGGGAAGAC
AATAGCAGGCATGCTGGGGATGCGGTGGGCTCTATGG

Start	End	Feature Description
1	380	CMV enhancer
381	619	CMV promoter
620	636	T7 promoter with mismatch
637	683	5' UTR
684	740	BP NLS
741	1238	Engineered TadA* variant 8e
1239	1334	(SGGS)2 – XTEN – (SGGS)2 linker
1335	5435	Cas9(D10A) Sp-VRQR PAM variant
5436	5501	Linker + SV40 BP NLS

5502	5605	3’ UTR
5606	5830	bGH poly(A) signal

Amino acid sequence

MKRTADGSEFESPKKKRKVSEVEFSHEYWMRHALTLAKRARDEREVPVGAVLVLNNRVIGEGWNRAIGL
HDPTAHAEIMALRQGGLVMQNYRLIDATLYVTFEPCVMCAGAMIHSRIGRVVFGVRNSKRGAAGSLMNVL
NYPGMNHRVEITEGILADECAALLCDFYRMPRQVFNAQKKAQSSINSGGSSGGSSGSETPGTSESATPE
SSGGSSGGSDKKYSIGLAIGTNSVGWAVITDEYKVPSKKFKVLGNTDRHSIKKNLIGALLFDSGETAEATRL
KRTARRRYTRRKNRICYLQEIFSNEMAKVDDSFFHRLEESFLVEEDKKHERHPIFGNIVDEVAYHEKYPTIY
HLRKKLVDSTDKADLRLIYLALAHMIKFRGHFLIEGDLNPDNSDVDKLFIQLVQTYNQLFEENPINASGVDA
KAILSARLSKSRRLENLIAQLPGEKKNGLFGNLIALSLGLTPNFKSNFDLAEDAKLQLSKDTYDDDLDNLLA
QIGDQYADLFLAAKNLSDAILLSDILRVNTEITKAPLSASMIKRYDEHHQDLTLLKALVRQQLPEKYKEIFFD
QSKNGYAGYIDGGASQEEFYKFIKPILEKMDGTEELLVKLNREDLLRKQRTFDNGSIPHQIHLGELHAILRR
QEDFYPFLKDNREKIEKILTFRIPYYVGPLARGNSRFAWMTRKSEETITPWNFEEVVDKGASAQSFIERMT
NFDKNLPNEKVLPKHSLLYEYFTVYNELTKVKYVTEGMRKPAFLSGEQKKAIVDLLFKTNRKVTVKQLKED
YFKKIECFDSVEISGVEDRFNASLGTYHDLLKIIKDKDFLDNEENEDILEDIVLTLTLFEDREMIEERLKTYAH
LFDDKVMKQLKRRRYTGWGRLSRKLINGIRDKQSGKTILDFLKSDGFANRNFMQLIHDDSLTFKEDIQKAQ
VSGQGDSLHEHIANLAGSPAIKKGILQTVKVVDELVKVMGRHKPENIVIEMARENQTTQKGQKNSRERMK
RIEEGIKELGSQILKEHPVENTQLQNEKLYLYYLQNGRDMYVDQELDINRLSDYDVDHIVPQSFLKDDSIDN
KVLTRSDKNRGKSDNVPSEEVVKKMKNYWRQLLNAKLITQRKFDNLTKAERGGLSELDKAGFIKRQLVET
RQITKHVAQILDSRMNTKYDENDKLIREVKVITLKSKLVSDFRKDFQFYKVREINNYHHAHDAYLNAVVGTA
LIKKYPKLESEFVYGDYKVYDVRKMIAKSEQEIGKATAKYFFYSNIMNFFKTEITLANGEIRKRPLIETNGET
GEIVWDKGRDFATVRKVLSMPQVNIVKKTEVQTGGFSKESILPKRNSDKLIARKKDWDPKKYGGFVSPTV
AYSVLVVAKVEKGKSKKLKSVKELLGITIMERSSFEKNPIDFLEAKGYKEVKKDLIIKLPKYSLFELENGRKR
MLASARELQKGNELALPSKYVNFLYLASHYEKLKGSPEDNEQKQLFVEQHKHYLDEIIEQISEFSKRVILAD
ANLDKVLSAYNKHRDKPIREQAENIIHLFTLTNLGAPAAFKYFDTTIDRKQYRSTKEVLDATLIHQSITGLYET
RIDLSQLGGDSGGSKRTADGSEFEPKKKRKV

Start	End	Feature Description
1	19	BP NLS
20	185	Engineered TadA* variant 8e
186	217	(SGGS)2 – XTEN – (SGGS)2 linker
218	1584	Cas9(D10A) Sp-VRQR PAM variant
1585	1605	Linker + SV40 BP NLS

ABE8e(V106W) – Cas9 (D10A) – VRQR variant
Plasmid ID: pHS0381

DNA sequence
GACATTGATTATTGACTAGTTATTAATAGTAATCAATTACGGGGTCATTAGTTCATAGCCCATATATGGAG
TTCCGCGTTACATAACTTACGGTAAATGGCCCGCCTGGCTGACCGCCCAACGACCCCCGCCCATTGA
CGTCAATAATGACGTATGTTCCCATAGTAACGCCAATAGGGACTTTCCATTGACGTCAATGGGTGGAGT
ATTTACGGTAAACTGCCCACTTGGCAGTACATCAAGTGTATCATATGCCAAGTACGCCCCCTATTGACG
TCAATGACGGTAAATGGCCCGCCTGGCATTATGCCCAGTACATGACCTTATGGGACTTTCCTACTTGG
CAGTACATCTACGTATTAGTCATCGCTATTACCATGGTGATGCGGTTTTGGCAGTACATCAATGGGCGT
GGATAGCGGTTTGACTCACGGGGATTTCCAAGTCTCCACCCCATTGACGTCAATGGGAGTTTGTTTTG
GCACCAAAATCAACGGGACTTTCCAAAATGTCGTAACAACTCCGCCCCATTGACGCAAATGGGCGGT
AGGCGTGTACGGTGGGAGGTCTATATAAGCAGAGCTGGTTTAGTGAACCGTCAGATCTCGAGCTCGG
TACCTAATACGACACACTATAAGGAAATAAGAGAGAAAAGAAGAGTAAGAAGAAATATAAGAGCCACCA
TGAAACGGACAGCCGACGGAAGCGAGTTCGAGTCACCAAAGAAGAAGCGGAAAGTCTCTGAGGTG
GAGTTTTCCCACGAGTACTGGATGAGACATGCCCTGACCCTGGCCAAGAGGGCACGGGATGAGAGG
GAGGTGCCTGTGGGAGCCGTGCTGGTGCTGAACAATAGAGTGATCGGCGAGGGCTGGAACAGAGC
CATCGGCCTGCACGACCCAACAGCCCATGCCGAAATTATGGCCCTGAGACAGGGCGGCCTGGTCAT
GCAGAACTACAGACTGATTGACGCCACCCTGTACGTGACATTCGAGCCTTGCGTGATGTGCGCCGGC
GCCATGATCCACTCTAGGATCGGCCGCGTGGTGTTTGGATGGAGAAATTCTAAAAGAGGCGCCGCAG
GCTCCCTGATGAACGTGCTGAACTACCCCGGCATGAATCACCGCGTCGAAATTACCGAGGGAATCCT
GGCAGATGAATGTGCCGCCCTGCTGTGCGATTTCTATCGGATGCCTAGACAGGTGTTCAATGCTCAG
AAGAAGGCCCAGAGCTCCATCAACTCCGGAGGATCTAGCGGAGGCTCCTCTGGCTCTGAGACACCT
GGCACAAGCGAGAGCGCAACACCTGAAAGCAGCGGGGGCAGCAGCGGGGGGTCAGACAAGAAGT
ACAGCATCGGCCTGGCCATCGGCACCAACTCTGTGGGCTGGGCCGTGATCACCGACGAGTACAAGG
TGCCCAGCAAGAAATTCAAGGTGCTGGGCAACACCGACCGGCACAGCATCAAGAAGAACCTGATCG
GAGCCCTGCTGTTCGACAGCGGCGAAACAGCCGAGGCCACCCGGCTGAAGAGAACCGCCAGAAGA
AGATACACCAGACGGAAGAACCGGATCTGCTATCTGCAAGAGATCTTCAGCAACGAGATGGCCAAGG
TGGACGACAGCTTCTTCCACAGACTGGAAGAGTCCTTCCTGGTGGAAGAGGATAAGAAGCACGAGC
GGCACCCCATCTTCGGCAACATCGTGGACGAGGTGGCCTACCACGAGAAGTACCCCACCATCTACCA
CCTGAGAAAGAAACTGGTGGACAGCACCGACAAGGCCGACCTGCGGCTGATCTATCTGGCCCTGGC
CCACATGATCAAGTTCCGGGGCCACTTCCTGATCGAGGGCGACCTGAACCCCGACAACAGCGACGT
GGACAAGCTGTTCATCCAGCTGGTGCAGACCTACAACCAGCTGTTCGAGGAAAACCCCATCAACGCC
AGCGGCGTGGACGCCAAGGCCATCCTGTCTGCCAGACTGAGCAAGAGCAGACGGCTGGAAAATCTG
ATCGCCCAGCTGCCCGGCGAGAAGAAGAATGGCCTGTTCGGAAACCTGATTGCCCTGAGCCTGGGC
CTGACCCCCAACTTCAAGAGCAACTTCGACCTGGCCGAGGATGCCAAACTGCAGCTGAGCAAGGAC
ACCTACGACGACGACCTGGACAACCTGCTGGCCCAGATCGGCGACCAGTACGCCGACCTGTTTCTG
GCCGCCAAGAACCTGTCCGACGCCATCCTGCTGAGCGACATCCTGAGAGTGAACACCGAGATCACC
AAGGCCCCCCTGAGCGCCTCTATGATCAAGAGATACGACGAGCACCACCAGGACCTGACCCTGCTG
AAAGCTCTCGTGCGGCAGCAGCTGCCTGAGAAGTACAAAGAGATTTTCTTCGACCAGAGCAAGAACG
GCTACGCCGGCTACATTGACGGCGGAGCCAGCCAGGAAGAGTTCTACAAGTTCATCAAGCCCATCCT
GGAAAAGATGGACGGCACCGAGGAACTGCTCGTGAAGCTGAACAGAGAGGACCTGCTGCGGAAGC
AGCGGACCTTCGACAACGGCAGCATCCCCCACCAGATCCACCTGGGAGAGCTGCACGCCATTCTGC
GGCGGCAGGAAGATTTTTACCCATTCCTGAAGGACAACCGGGAAAAGATCGAGAAGATCCTGACCTT
CCGCATCCCCTACTACGTGGGCCCTCTGGCCAGGGGAAACAGCAGATTCGCCTGGATGACCAGAAA
GAGCGAGGAAACCATCACCCCCTGGAACTTCGAGGAAGTGGTGGACAAGGGCGCTTCCGCCCAGA
GCTTCATCGAGCGGATGACCAACTTCGATAAGAACCTGCCCAACGAGAAGGTGCTGCCCAAGCACA
GCCTGCTGTACGAGTACTTCACCGTGTATAACGAGCTGACCAAAGTGAAATACGTGACCGAGGGAAT
GAGAAAGCCCGCCTTCCTGAGCGGCGAGCAGAAAAAGGCCATCGTGGACCTGCTGTTCAAGACCAA
CCGGAAAGTGACCGTGAAGCAGCTGAAAGAGGACTACTTCAAGAAAATCGAGTGCTTCGACTCCGTG
GAAATCTCCGGCGTGGAAGATCGGTTCAACGCCTCCCTGGGCACATACCACGATCTGCTGAAAATTAT
CAAGGACAAGGACTTCCTGGACAATGAGGAAAACGAGGACATTCTGGAAGATATCGTGCTGACCCTG
ACACTGTTTGAGGACAGAGAGATGATCGAGGAACGGCTGAAAACCTATGCCCACCTGTTCGACGACA
AAGTGATGAAGCAGCTGAAGCGGCGGAGATACACCGGCTGGGGCAGGCTGAGCCGGAAGCTGATC
AACGGCATCCGGGACAAGCAGTCCGGCAAGACAATCCTGGATTTCCTGAAGTCCGACGGCTTCGCC
AACAGAAACTTCATGCAGCTGATCCACGACGACAGCCTGACCTTTAAAGAGGACATCCAGAAAGCCC

AGGTGTCCGGCCAGGGCGATAGCCTGCACGAGCACATTGCCAATCTGGCCGGCAGCCCCGCCATTA
AGAAGGGCATCCTGCAGACAGTGAAGGTGGTGGACGAGCTCGTGAAAGTGATGGGCCGGCACAAG
CCCGAGAACATCGTGATCGAAATGGCCAGAGAGAACCAGACCACCCAGAAGGGACAGAAGAACAGC
CGCGAGAGAATGAAGCGGATCGAAGAGGGCATCAAAGAGCTGGGCAGCCAGATCCTGAAAGAACAC
CCCGTGGAAAACACCCAGCTGCAGAACGAGAAGCTGTACCTGTACTACCTGCAGAATGGGCGGGATA
TGTACGTGGACCAGGAACTGGACATCAACCGGCTGTCCGACTACGATGTGGACCATATCGTGCCTCA
GAGCTTTCTGAAGGACGACTCCATCGACAACAAGGTGCTGACCAGAAGCGACAAGAACCGGGGCAA
GAGCGACAACGTGCCCTCCGAAGAGGTCGTGAAGAAGATGAAGAACTACTGGCGGCAGCTGCTGAA
CGCCAAGCTGATTACCCAGAGAAAGTTCGACAATCTGACCAAGGCCGAGAGAGGCGGCCTGAGCGA
ACTGGATAAGGCCGGCTTCATCAAGAGACAGCTGGTGGAAACCCGGCAGATCACAAAGCACGTGGC
ACAGATCCTGGACTCCCGGATGAACACTAAGTACGACGAGAATGACAAGCTGATCCGGGAAGTGAAA
GTGATCACCCTGAAGTCCAAGCTGGTGTCCGATTTCCGGAAGGATTTCCAGTTTTACAAAGTGCGCG
AGATCAACAACTACCACCACGCCCACGACGCCTACCTGAACGCCGTCGTGGGAACCGCCCTGATCA
AAAAGTACCCTAAGCTGGAAAGCGAGTTCGTGTACGGCGACTACAAGGTGTACGACGTGCGGAAGAT
GATCGCCAAGAGCGAGCAGGAAATCGGCAAGGCTACCGCCAAGTACTTCTTCTACAGCAACATCATG
AACTTTTTCAAGACCGAGATTACCCTGGCCAACGGCGAGATCCGGAAGCGGCCTCTGATCGAGACAA
ACGGCGAAACCGGGGAGATCGTGTGGGATAAGGGCCGGGATTTTGCCACCGTGCGGAAAGTGCTG
AGCATGCCCCAAGTGAATATCGTGAAAAAGACCGAGGTGCAGACAGGCGGCTTCAGCAAAGAGTCTA
TCCTGCCCAAGAGGAACAGCGATAAGCTGATCGCCAGAAAGAAGGACTGGGACCCTAAGAAGTACG
GCGGCTTCGTGAGCCCCACCGTGGCCTATTCTGTGCTGGTGGTGGCCAAAGTGGAAAAGGGCAAGT
CCAAGAAACTGAAGAGTGTGAAAGAGCTGCTGGGGATCACCATCATGGAAAGAAGCAGCTTCGAGAA
GAATCCCATCGACTTTCTGGAAGCCAAGGGCTACAAAGAAGTGAAAAAGGACCTGATCATCAAGCTG
CCTAAGTACTCCCTGTTCGAGCTGGAAAACGGCCGGAAGAGAATGCTGGCCTCAGCCAGAGAACTG
CAGAAGGGAAACGAACTGGCCCTGCCCTCCAAATATGTGAACTTCCTGTACCTGGCCAGCCACTATG
AGAAGCTGAAGGGCTCCCCCGAGGATAATGAGCAGAAACAGCTGTTTGTGGAACAGCACAAGCACTA
CCTGGACGAGATCATCGAGCAGATCAGCGAGTTCTCCAAGAGAGTGATCCTGGCCGACGCTAATCTG
GACAAAGTGCTGTCCGCCTACAACAAGCACCGGGATAAGCCCATCAGAGAGCAGGCCGAGAATATCA
TCCACCTGTTTACCCTGACCAATCTGGGAGCCCCTGCCGCCTTCAAGTACTTTGACACCACCATCGA
CCGGAAGCAGTACAGAAGCACCAAAGAGGTGCTGGACGCCACCCTGATCCACCAGAGCATCACCGG
CCTGTACGAGACACGGATCGACCTGTCTCAGCTGGGAGGTGACTCTGGCGGCTCAAAAAGAACCGC
CGACGGCAGCGAATTCGAGCCCAAGAAGAAGAGGAAAGTCTAATTAATTAAGCTGCCTTCTGCGGGG
CTTGCCTTCTGGCCATGCCCTTCTTCTCTCCCTTGCACCTGTACCTCTTGGTCTTTGAATAAAGCCTG
AGTAGGAAGCGACTGTGCCTTCTAGTTGCCAGCCATCTGTTGTTTGCCCCTCCCCCGTGCCTTCCTT
GACCCTGGAAGGTGCCACTCCCACTGTCCTTTCCTAATAAAATGAGGAAATTGCATCGCATTGTCTGA
GTAGGTGTCATTCTATTCTGGGGGGTGGGGTGGGGCAGGACAGCAAGGGGGAGGATTGGGAAGAC
AATAGCAGGCATGCTGGGGATGCGGTGGGCTCTATGG

Start	End	Feature Description
1	380	CMV enhancer
381	619	CMV promoter
620	636	T7 promoter with mismatch
637	683	5' UTR
684	740	BP NLS
741	1238	Engineered TadA* variant 8e(V106W)
1239	1334	(SGGS)2 – XTEN – (SGGS)2 linker
1335	5435	Cas9(D10A) Sp-VRQR PAM variant
5436	5501	Linker + SV40 BP NLS

5502	5605	3' UTR
5606	5830	bGH poly(A) signal

Amino acid sequence

MKRTADGSEFESPKKKRKVSEVEFSHEYWMRHALTLAKRARDEREVPVGAVLVLNNRVIGEGWNRAIGL
HDPTAHAEIMALRQGGLVMQNYRLIDATLYVTFEPCVMCAGAMIHSRIGRVVFGWRNSKRGAAGSLMNV
LNYPGMNHRVEITEGILADECAALLCDFYRMPRQVFNAQKKAQSSINSGGSSGGSSGSETPGTSESATP
ESSGGSSGGSDKKYSIGLAIGTNSVGWAVITDEYKVPSKKFKVLGNTDRHSIKKNLIGALLFDSGETAEAT
RLKRTARRRYTRRKNRICYLQEIFSNEMAKVDDSFFHRLEESFLVEEDKKHERHPIFGNIVDEVAYHEKYP
TIYHLRKKLVDSTDKADLRLIYLALAHMIKFRGHFLIEGDLNPDNSDVDKLFIQLVQTYNQLFEENPINASGV
DAKAILSARLSKSRRLENLIAQLPGEKKNGLFGNLIALSLGLTPNFKSNFDLAEDAKLQLSKDTYDDDLDNL
LAQIGDQYADLFLAAKNLSDAILLSDILRVNTEITKAPLSASMIKRYDEHHQDLTLLKALVRQQLPEKYKEIFF
DQSKNGYAGYIDGGASQEEFYKFIKPILEKMDGTEELLVKLNREDLLRKQRTFDNGSIPHQIHLGELHAILR
RQEDFYPFLKDNREKIEKILTFRIPYYVGPLARGNSRFAWMTRKSEETITPWNFEEVVDKGASAQSFIERM
TNFDKNLPNEKVLPKHSLLYEYFTVYNELTKVKYVTEGMRKPAFLSGEQKKAIVDLLFKTNRKVTVKQLKE
DYFKKIECFDSVEISGVEDRFNASLGTYHDLLKIIKDKDFLDNEENEDILEDIVLTLTLFEDREMIEERLKTYA
HLFDDKVMKQLKRRRYTGWGRLSRKLINGIRDKQSGKTILDFLKSDGFANRNFMQLIHDDSLTFKEDIQKA
QVSGQGDSLHEHIANLAGSPAIKKGILQTVKVVDELVKVMGRHKPENIVIEMARENQTTQKGQKNSRERM
KRIEEGIKELGSQILKEHPVENTQLQNEKLYLYYLQNGRDMYVDQELDINRLSDYDVDHIVPQSFLKDDSID
NKVLTRSDKNRGKSDNVPSEEVVKKMKNYWRQLLNAKLITQRKFDNLTKAERGGLSELDKAGFIKRQLVE
TRQITKHVAQILDSRMNTKYDENDKLIREVKVITLKSKLVSDFRKDFQFYKVREINNYHHAHDAYLNAVVGT
ALIKKYPKLESEFVYGDYKVYDVRKMIAKSEQEIGKATAKYFFYSNIMNFFKTEITLANGEIRKRPLIETNGE
TGEIVWDKGRDFATVRKVLSMPQVNIVKKTEVQTGGFSKESILPKRNSDKLIARKKDWDPKKYGGFVSPT
VAYSVLVVAKVEKGKSKKLKSVKELLGITIMERSSFEKNPIDFLEAKGYKEVKKDLIIKLPKYSLFELENGRK
RMLASARELQKGNELALPSKYVNFLYLASHYEKLKGSPEDNEQKQLFVEQHKHYLDEIIEQISEFSKRVILA
DANLDKVLSAYNKHRDKPIREQAENIIHLFTLTNLGAPAAFKYFDTTIDRKQYRSTKEVLDATLIHQSITGLYE
TRIDLSQLGGDSGGSKRTADGSEFEPKKKRKV

Start	End	Feature Description
1	19	BP NLS
20	185	Engineered TadA* variant 8e
186	217	(SGGS)2 – XTEN – (SGGS)2 linker
218	1584	Cas9(D10A) Sp-VRQR PAM variant
1585	1605	Linker + SV40 BP NLS

ABE9 – Cas9 (D10A) – VRQR variant
Plasmid ID: pHS0410

DNA sequence

GACATTGATTATTGACTAGTTATTAATAGTAATCAATTACGGGGTCATTAGTTCATAGCCCATATATGGAG
TTCCGCGTTACATAACTTACGGTAAATGGCCCGCCTGGCTGACCGCCCAACGACCCCCGCCCATTGA
CGTCAATAATGACGTATGTTCCCATAGTAACGCCAATAGGGACTTTCCATTGACGTCAATGGGTGGAGT
ATTTACGGTAAACTGCCCACTTGGCAGTACATCAAGTGTATCATATGCCAAGTACGCCCCCTATTGACG
TCAATGACGGTAAATGGCCCGCCTGGCATTATGCCCAGTACATGACCTTATGGGACTTTCCTACTTGG
CAGTACATCTACGTATTAGTCATCGCTATTACCATGGTGATGCGGTTTTGGCAGTACATCAATGGGCGT
GGATAGCGGTTTGACTCACGGGGATTTCCAAGTCTCCACCCCATTGACGTCAATGGGAGTTTGTTTTG
GCACCAAAATCAACGGGACTTTCCAAAATGTCGTAACAACTCCGCCCCATTGACGCAAATGGGCGGT
AGGCGTGTACGGTGGGAGGTCTATATAAGCAGAGCTGGTTTAGTGAACCGTCAGATCTCGAGCTCGG
TACCTAATACGACACACTATAAGGAAATAAGAGAGAAAAGAAGAGTAAGAAGAAATATAAGAGCCACCA
TGAAACGGACAGCCGACGGAAGCGAGTTCGAGTCACCAAAGAAGAAGCGGAAAGTCTCTGAGGTG
GAGTTTTCCCACGAGTACTGGATGAGACATGCCCTGACCCTGGCCAAGAGGGCACGGGATGAGAGG
GAGGTGCCTGTGGGAGCCGTGCTGGTGCTGAACAATAGAGTGATCGGCGAGGGCTGGAACAGAGC
CATCGGCCTGCACGACCCAACAGCCCATGCCGAAATTATGGCCCTGAGACAGGGCGGCCTGGTCAT
GCAGAACTACAGACTGATTGACGCCACCCTGTACGTGACATTCGAGCCTTGCGTGATGTGCGCCGGC
GCCATGATCCACTCTAGGATCGGCCGCGTGGTGTTTGGCGTGAGGCAGTCAAAAAGAGGCGCCGCA
GGCTCCCTGATGAACGTGCTGAACTACCCCGGCATGAATCACCGCGTCGAAATTACCGAGGGAATCC
TGGCAGATGAATGTGCCGCCCTGACCTGCGATTTCTATCGGATGCCTAGACAGGTGTTCAATGCTCAG
AAGAAGGCCCAGAGCTCCATCAACTCCGGAGGATCTAGCGGAGGCTCCTCTGGCTCTGAGACACCT
GGCACAAGCGAGAGCGCAACACCTGAAAGCAGCGGGGGCAGCAGCGGGGGGTCAGACAAGAAGT
ACAGCATCGGCCTGGCCATCGGCACCAACTCTGTGGGCTGGGCCGTGATCACCGACGAGTACAAGG
TGCCCAGCAAGAAATTCAAGGTGCTGGGCAACACCGACCGGCACAGCATCAAGAAGAACCTGATCG
GAGCCCTGCTGTTCGACAGCGGCGAAACAGCCGAGGCCACCCGGCTGAAGAGAACCGCCAGAAGA
AGATACACCAGACGGAAGAACCGGATCTGCTATCTGCAAGAGATCTTCAGCAACGAGATGGCCAAGG
TGGACGACAGCTTCTTCCACAGACTGGAAGAGTCCTTCCTGGTGGAAGAGGATAAGAAGCACGAGC
GGCACCCCATCTTCGGCAACATCGTGGACGAGGTGGCCTACCACGAGAAGTACCCCACCATCTACCA
CCTGAGAAAGAAACTGGTGGACAGCACCGACAAGGCCGACCTGCGGCTGATCTATCTGGCCCTGGC
CCACATGATCAAGTTCCGGGGCCACTTCCTGATCGAGGGCGACCTGAACCCCGACAACAGCGACGT
GGACAAGCTGTTCATCCAGCTGGTGCAGACCTACAACCAGCTGTTCGAGGAAAACCCCATCAACGCC
AGCGGCGTGGACGCCAAGGCCATCCTGTCTGCCAGACTGAGCAAGAGCAGACGGCTGGAAAATCTG
ATCGCCCAGCTGCCCGGCGAGAAGAAGAATGGCCTGTTCGGAAACCTGATTGCCCTGAGCCTGGGC
CTGACCCCCAACTTCAAGAGCAACTTCGACCTGGCCGAGGATGCCAAACTGCAGCTGAGCAAGGAC
ACCTACGACGACGACCTGGACAACCTGCTGGCCCAGATCGGCGACCAGTACGCCGACCTGTTTCTG
GCCGCCAAGAACCTGTCCGACGCCATCCTGCTGAGCGACATCCTGAGAGTGAACACCGAGATCACC
AAGGCCCCCCTGAGCGCCTCTATGATCAAGAGATACGACGAGCACCACCAGGACCTGACCCTGCTG
AAAGCTCTCGTGCGGCAGCAGCTGCCTGAGAAGTACAAAGAGATTTTCTTCGACCAGAGCAAGAACG
GCTACGCCGGCTACATTGACGGCGGAGCCAGCCAGGAAGAGTTCTACAAGTTCATCAAGCCCATCCT
GGAAAAGATGGACGGCACCGAGGAACTGCTCGTGAAGCTGAACAGAGAGGACCTGCTGCGGAAGC
AGCGGACCTTCGACAACGGCAGCATCCCCCACCAGATCCACCTGGGAGAGCTGCACGCCATTCTGC
GGCGGCAGGAAGATTTTTACCCATTCCTGAAGGACAACCGGGAAAAGATCGAGAAGATCCTGACCTT
CCGCATCCCCTACTACGTGGGCCCTCTGGCCAGGGGAAACAGCAGATTCGCCTGGATGACCAGAAA
GAGCGAGGAAACCATCACCCCCTGGAACTTCGAGGAAGTGGTGGACAAGGGCGCTTCCGCCCAGA
GCTTCATCGAGCGGATGACCAACTTCGATAAGAACCTGCCCAACGAGAAGGTGCTGCCCAAGCACA
GCCTGCTGTACGAGTACTTCACCGTGTATAACGAGCTGACCAAAGTGAAATACGTGACCGAGGGAAT
GAGAAAGCCCGCCTTCCTGAGCGGCGAGCAGAAAAAGGCCATCGTGGACCTGCTGTTCAAGACCAA
CCGGAAAGTGACCGTGAAGCAGCTGAAAGAGGACTACTTCAAGAAAATCGAGTGCTTCGACTCCGTG
GAAATCTCCGGCGTGGAAGATCGGTTCAACGCCTCCCTGGGCACATACCACGATCTGCTGAAAATTAT
CAAGGACAAGGACTTCCTGGACAATGAGGAAAACGAGGACATTCTGGAAGATATCGTGCTGACCCTG
ACACTGTTTGAGGACAGAGAGATGATCGAGGAACGGCTGAAAACCTATGCCCACCTGTTCGACGACA
AAGTGATGAAGCAGCTGAAGCGGCGGAGATACACCGGCTGGGGCAGGCTGAGCCGGAAGCTGATC
AACGGCATCCGGGACAAGCAGTCCGGCAAGACAATCCTGGATTTCCTGAAGTCCGACGGCTTCGCC
AACAGAAACTTCATGCAGCTGATCCACGACGACAGCCTGACCTTTAAAGAGGACATCCAGAAAGCCC

AGGTGTCCGGCCAGGGCGATAGCCTGCACGAGCACATTGCCAATCTGGCCGGCAGCCCCGCCATTA
AGAAGGGCATCCTGCAGACAGTGAAGGTGGTGGACGAGCTCGTGAAAGTGATGGGCCGGCACAAG
CCCGAGAACATCGTGATCGAAATGGCCAGAGAGAACCAGACCACCCAGAAGGGACAGAAGAACAGC
CGCGAGAGAATGAAGCGGATCGAAGAGGGCATCAAAGAGCTGGGCAGCCAGATCCTGAAAGAACAC
CCCGTGGAAAACACCCAGCTGCAGAACGAGAAGCTGTACCTGTACTACCTGCAGAATGGGCGGGATA
TGTACGTGGACCAGGAACTGGACATCAACCGGCTGTCCGACTACGATGTGGACCATATCGTGCCTCA
GAGCTTTCTGAAGGACGACTCCATCGACAACAAGGTGCTGACCAGAAGCGACAAGAACCGGGGCAA
GAGCGACAACGTGCCCTCCGAAGAGGTCGTGAAGAAGATGAAGAACTACTGGCGGCAGCTGCTGAA
CGCCAAGCTGATTACCCAGAGAAAGTTCGACAATCTGACCAAGGCCGAGAGAGGCGGCCTGAGCGA
ACTGGATAAGGCCGGCTTCATCAAGAGACAGCTGGTGGAAACCCGGCAGATCACAAAGCACGTGGC
ACAGATCCTGGACTCCCGGATGAACACTAAGTACGACGAGAATGACAAGCTGATCCGGGAAGTGAAA
GTGATCACCCTGAAGTCCAAGCTGGTGTCCGATTTCCGGAAGGATTTCCAGTTTTACAAAGTGCGCG
AGATCAACAACTACCACCACGCCCACGACGCCTACCTGAACGCCGTCGTGGGAACCGCCCTGATCA
AAAAGTACCCTAAGCTGGAAAGCGAGTTCGTGTACGGCGACTACAAGGTGTACGACGTGCGGAAGAT
GATCGCCAAGAGCGAGCAGGAAATCGGCAAGGCTACCGCCAAGTACTTCTTCTACAGCAACATCATG
AACTTTTTCAAGACCGAGATTACCCTGGCCAACGGCGAGATCCGGAAGCGGCCTCTGATCGAGACAA
ACGGCGAAACCGGGGAGATCGTGTGGGATAAGGGCCGGGATTTTGCCACCGTGCGGAAAGTGCTG
AGCATGCCCCAAGTGAATATCGTGAAAAAGACCGAGGTGCAGACAGGCGGCTTCAGCAAAGAGTCTA
TCCTGCCCAAGAGGAACAGCGATAAGCTGATCGCCAGAAAGAAGGACTGGGACCCTAAGAAGTACG
GCGGCTTCGTGAGCCCCACCGTGGCCTATTCTGTGCTGGTGGTGGCCAAAGTGGAAAAGGGCAAGT
CCAAGAAACTGAAGAGTGTGAAAGAGCTGCTGGGGATCACCATCATGGAAAGAAGCAGCTTCGAGAA
GAATCCCATCGACTTTCTGGAAGCCAAGGGCTACAAAGAAGTGAAAAAGGACCTGATCATCAAGCTG
CCTAAGTACTCCCTGTTCGAGCTGGAAAACGGCCGGAAGAGAATGCTGGCCTCAGCCAGAGAACTG
CAGAAGGGAAACGAACTGGCCCTGCCCTCCAAATATGTGAACTTCCTGTACCTGGCCAGCCACTATG
AGAAGCTGAAGGGCTCCCCCGAGGATAATGAGCAGAAACAGCTGTTTGTGGAACAGCACAAGCACTA
CCTGGACGAGATCATCGAGCAGATCAGCGAGTTCTCCAAGAGAGTGATCCTGGCCGACGCTAATCTG
GACAAAGTGCTGTCCGCCTACAACAAGCACCGGGATAAGCCCATCAGAGAGCAGGCCGAGAATATCA
TCCACCTGTTTACCCTGACCAATCTGGGAGCCCCTGCCGCCTTCAAGTACTTTGACACCACCATCGA
CCGGAAGCAGTACAGAAGCACCAAAGAGGTGCTGGACGCCACCCTGATCCACCAGAGCATCACCGG
CCTGTACGAGACACGGATCGACCTGTCTCAGCTGGGAGGTGACTCTGGCGGCTCAAAAAGAACCGC
CGACGGCAGCGAATTCGAGCCCAAGAAGAAGAGGAAAGTCTAATTAATTAAGCTGCCTTCTGCGGGG
CTTGCCTTCTGGCCATGCCCTTCTTCTCTCCCTTGCACCTGTACCTCTTGGTCTTTGAATAAAGCCTG
AGTAGGAAGCGACTGTGCCTTCTAGTTGCCAGCCATCTGTTGTTTGCCCCTCCCCCGTGCCTTCCTT
GACCCTGGAAGGTGCCACTCCCACTGTCCTTTCCTAATAAAATGAGAAATTGCATCGCATTGTCTGA
GTAGGTGTCATTCTATTCTGGGGGGTGGGGTGGGGCAGGACAGCAAGGGGGAGGATTGGGAAGAC
AATAGCAGGCATGCTGGGGATGCGGTGGGCTCTATGG

Start	End	Feature Description
1	380	CMV enhancer
381	619	CMV promoter
620	636	T7 promoter with mismatch
637	683	5' UTR
684	740	BP NLS
741	1238	Engineered TadA* variant ABE9 (TadA-8e N108Q L145T)
1239	1334	(SGGS)2 – XTEN – (SGGS)2 linker
1335	5435	Cas9(D10A) Sp-VRQR PAM variant
5436	5501	Linker + SV40 BP NLS

5502	5605	3’ UTR
5606	5830	bGH poly(A) signal

Amino acid sequence
MKRTADGSEFESPKKKRKVSEVEFSHEYWMRHALTLAKRARDEREVPVGAVLVLNNRVIGEGWNRAIGL
HDPTAHAEIMALRQGGLVMQNYRLIDATLYVTFEPCVMCAGAMIHSRIGRVVFGVRQSKRGAAGSLMNVL
NYPGMNHRVEITEGILADECAALTCDFYRMPRQVFNAQKKAQSSINSGGSSGGSSGSETPGTSESATPE
SSGGSSGGSDKKYSIGLAIGTNSVGWAVITDEYKVPSKKFKVLGNTDRHSIKKNLIGALLFDSGETAEATRL
KRTARRRYTRRKNRICYLQEIFSNEMAKVDDSFFHRLEESFLVEEDKKHERHPIFGNIVDEVAYHEKYPTIY
HLRKKLVDSTDKADLRLIYLALAHMIKFRGHFLIEGDLNPDNSDVDKLFIQLVQTYNQLFEENPINASGVDA
KAILSARLSKSRRLENLIAQLPGEKKNGLFGNLIALSLGLTPNFKSNFDLAEDAKLQLSKDTYDDDLDNLLA
QIGDQYADLFLAAKNLSDAILLSDILRVNTEITKAPLSASMIKRYDEHHQDLTLLKALVRQQLPEKYKEIFFD
QSKNGYAGYIDGGASQEEFYKFIKPILEKMDGTEELLVKLNREDLLRKQRTFDNGSIPHQIHLGELHAILRR
QEDFYPFLKDNREKIEKILTFRIPYYVGPLARGNSRFAWMTRKSEETITPWNFEEVVDKGASAQSFIERMT
NFDKNLPNEKVLPKHSLLYEYFTVYNELTKVKYVTEGMRKPAFLSGEQKKAIVDLLFKTNRKVTVKQLKED
YFKKIECFDSVEISGVEDRFNASLGTYHDLLKIIKDKDFLDNEENEDILEDIVLTLTLFEDREMIEERLKTYAH
LFDDKVMKQLKRRRYTGWGRLSRKLINGIRDKQSGKTILDFLKSDGFANRNFMQLIHDDSLTFKEDIQKAQ
VSGQGDSLHEHIANLAGSPAIKKGILQTVKVVDELVKVMGRHKPENIVIEMARENQTTQKGQKNSRERMK
RIEEGIKELGSQILKEHPVENTQLQNEKLYLYYLQNGRDMYVDQELDINRLSDYDVDHIVPQSFLKDDSIDN
KVLTRSDKNRGKSDNVPSEEVVKKMKNYWRQLLNAKLITQRKFDNLTKAERGGLSELDKAGFIKRQLVET
RQITKHVAQILDSRMNTKYDENDKLIREVKVITLKSKLVSDFRKDFQFYKVREINNYHHAHDAYLNAVVGTA
LIKKYPKLESEFVYGDYKVYDVRKMIAKSEQEIGKATAKYFFYSNIMNFFKTEITLANGEIRKRPLIETNGET
GEIVWDKGRDFATVRKVLSMPQVNIVKKTEVQTGGFSKESILPKRNSDKLIARKKDWDPKKYGGFVSPTV
AYSVLVVAKVEKGKSKKLKSVKELLGITIMERSSFEKNPIDFLEAKGYKEVKKDLIIKLPKYSLFELENGRKR
MLASARELQKGNELALPSKYVNFLYLASHYEKLKGSPEDNEQKQLFVEQHKHYLDEIIEQISEFSKRVILAD
ANLDKVLSAYNKHRDKPIREQAENIIHLFTLTNLGAPAAFKYFDTTIDRKQYRSTKEVLDATLIHQSITGLYET
RIDLSQLGGDSGGSKRTADGSEFEPKKKRKV

Start	End	Feature Description
1	19	BP NLS
20	185	Engineered TadA* variant 8e
186	217	(SGGS)2 – XTEN – (SGGS)2 linker
218	1584	Cas9(D10A) Sp-VRQR PAM variant
1585	1605	Linker + SV40 BP NLS

ABE7.10 – Cas9 (D10T) – NRCH variant
Plasmid ID: pHS0378

DNA sequence
GACATTGATTATTGACTAGTTATTAATAGTAATCAATTACGGGGTCATTAGTTCATAGCCCATATATGGAG
TTCCGCGTTACATAACTTACGGTAAATGGCCCGCCTGGCTGACCGCCCAACGACCCCCGCCCATTGA
CGTCAATAATGACGTATGTTCCCATAGTAACGCCAATAGGGACTTTCCATTGACGTCAATGGGTGGAGT
ATTTACGGTAAACTGCCCACTTGGCAGTACATCAAGTGTATCATATGCCAAGTACGCCCCCTATTGACG
TCAATGACGGTAAATGGCCCGCCTGGCATTATGCCCAGTACATGACCTTATGGGACTTTCCTACTTGG
CAGTACATCTACGTATTAGTCATCGCTATTACCATGGTGATGCGGTTTTGGCAGTACATCAATGGGCGT
GGATAGCGGTTTGACTCACGGGGATTTCCAAGTCTCCACCCCATTGACGTCAATGGGAGTTTGTTTTG
GCACCAAAATCAACGGGACTTTCCAAAATGTCGTAACAACTCCGCCCCATTGACGCAAATGGGCGGT
AGGCGTGTACGGTGGGAGGTCTATATAAGCAGAGCTGGTTTAGTGAACCGTCAGATCTCGAGCTCGG
TACCTAATACGACACACTATAAGGAAATAAGAGAGAAAAGAAGAGTAAGAAGAAATATAAGAGCCACCA
TGAAACGGACAGCCGACGGAAGCGAGTTCGAGTCACCAAAGAAGAAGCGGAAAGTCTCTGAAGTCG
AGTTTAGCCACGAGTATTGGATGAGGCACGCACTGACCCTGGCAAAGCGAGCATGGGATGAAAGAGA
AGTCCCCGTGGGCGCCGTGCTGGTGCACAACAATAGAGTGATCGGAGAGGGATGGAACAGGCCAAT
CGGCCGCCACGACCCTACCGCACACGCAGAGATCATGGCACTGAGGCAGGGAGGCCTGGTCATGC
AGAATTACCGCCTGATCGATGCCACCCTGTATGTGACACTGGAGCCATGCGTGATGTGCGCAGGAGC
AATGATCCACAGCAGGATCGGAAGAGTGGTGTTCGGAGCACGGGACGCCAAGACCGGCGCAGCAG
GCTCCCTGATGGATGTGCTGCACCACCCCGGCATGAACCACCGGGTGGAGATCACAGAGGGAATCC
TGGCAGACGAGTGCGCCGCCCTGCTGAGCGATTTCTTTAGAATGCGGAGACAGGAGATCAAGGCCC
AGAAGAAGGCACAGAGCTCCACCGACTCTGGAGGATCTAGCGGAGGATCCTCTGGAAGCGAGACAC
CAGGCACAAGCGAGTCCGCCACACCAGAGAGCTCCGGCGGCTCCTCCGGAGGATCCTCTGAGGTG
GAGTTTTCCCACGAGTACTGGATGAGACATGCCCTGACCCTGGCCAAGAGGGCACGCGATGAGAGG
GAGGTGCCTGTGGGAGCCGTGCTGGTGCTGAACAATAGAGTGATCGGCGAGGGCTGGAACAGAGC
CATCGGCCTGCACGACCCAACAGCCCATGCCGAAATTATGGCCCTGAGACAGGGCGGCCTGGTCAT
GCAGAACTACAGACTGATTGACGCCACCCTGTACGTGACATTCGAGCCTTGCGTGATGTGCGCCGGC
GCCATGATCCACTCTAGGATCGGCCGCGTGGTGTTTGGCGTGAGGAACGCAAAAACCGGCGCCGCA
GGCTCCCTGATGGACGTGCTGCACTACCCCGGCATGAATCACCGCGTCGAAATTACCGAGGGAATCC
TGGCAGATGAATGTGCCGCCCTGCTGTGCTATTTCTTTCGGATGCCTAGACAGGTGTTCAATGCTCAG
AAGAAGGCCCAGAGCTCCACCGACTCCGGAGGATCTAGCGGAGGCTCCTCTGGCTCTGAGACACCT
GGCACAAGCGAGAGCGCAACACCTGAAAGCAGCGGGGGCAGCAGCGGGGGGTCAGACAAGAAGT
ACAGCATCGGCCTGACCATCGGCACCAACTCTGTGGGCTGGGCCGTGATCACCGACGAGTACAAGG
TGCCCAGCAAGAAATTCAAGGTGCTGGGCAACACCGACCGGCACAGCATCAAGAAGAACCTGATCG
GAGCCCTGCTGTTCGACAGCGGCGAAACAGCCGAGGCCACCCGGCTGAAGAGAACCGCCAGAAGA
AGATACACCAGACGGAAGAACCGGATCTGCTATCTGCAAGAGATCTTCAGCAACGAGATGGCCAAGG
TGGACGACAGCTTCTTCCACAGACTGGAAGAGTCCTTCCTGGTGGAAGAGGATAAGAAGCACGAGC
GGCACCCCATCTTCGGCAACATCGTGGACGAGGTGGCCTACCACGAGAAGTACCCCACCATCTACCA
CCTGAGAAAGAAACTGGTGGACAGCACCGACAAGGCCGACCTGCGGCTGATCTATCTGGCCCTGGC
CCACATGATCAAGTTCCGGGGCCACTTCCTGATCGAGGGCGACCTGAACCCCGACAACAGCGACGT
GGACAAGCTGTTCATCCAGCTGGTGCAGACCTACAACCAGCTGTTCGAGGAAAACCCCATCAACGCC
AGCGGCGTGGACGCCAAGGCCATCCTGTCTGCCAGACTGAGCAAGAGCAGACGGCTGGAAAATCTG
ATCGCCCAGCTGCCCGGCGAGAAGAAGAATGGCCTGTTCGGAAACCTGATTGCCCTGAGCCTGGGC
CTGACCCCCAACTTCAAGAGCAACTTCGACCTGGCCGAGGATGCCAAACTGCAGCTGAGCAAGGAC
ACCTACGACGACGACCTGGACAACCTGCTGGCCCAGATCGGCGACCAGTACGCCGACCTGTTTCTG
GCCGCCAAGAACCTGTCCGACGCCATCCTGCTGAGCGACATCCTGAGAGTGAACACCGAGATCACC
AAGGCCCCCCTGAGCGCCTCTATGGTGAAGAGATACGACGAGCACCACCAGGACCTGACCCTGCTG
AAAGCTCTCGTGCGGCAGCAGCTGCCTGAGAAGTACAAAGAGATTTTCTTCGACCAGAGCAAGAACG
GCTACGCCGGCTACATTGACGGCGGAGCCAGCCAGGAAGAGTTCTACAAGTTCATCAAGCCCATCCT
GGAAAAGATGGACGGCACCGAGGAACTGCTCGTGAAGCTGAACAGAGAGGACCTGCTGCGGAAGC
AGCGGACCTTCGACAACGGCATTATCCCCCACCAGATCCACCTGGGAGAGCTGCACGCCATTCTGC
GGCGGCAGGGCGATTTTTACCCATTCCTGAAGGACAACCGGGAAAAGATCGAGAAGATCCTGACCTT
CCGCATCCCCTACTACGTGGGCCCTCTGGCCAGGGGAAACAGCAGATTCGCCTGGATGACCAGAAA
GAGCGAGGAAACCATCACCCCCTGGAACTTCGAGGAAGTGGTGGACAAGGGCGCTTCCGCCCAGA
GCTTCATCGAGCGGATGACCAACTTCGATAAGAACCTGCCCAACGAGAAGGTGCTGCCCAAGCACA

GCCTGCTGTACGAGTACTTCACCGTGTATAACGAGCTGACCAAAGTGAAATACGTGACCGAGGGAAT
GAGAAAGCCCGCCTTCCTGAGCGGCGAGCAGAAAAAGGCCATCGTGGACCTGCTGTTCAAGACCAA
CCGGAAAGTGACCGTGAAGCAGCTGAAAGAGGACTACTTCAAGAAAATCGAGTGCTTCGACTCCGTG
GAAATCTCCGGCGTGGAAGATCGGTTCAACGCCTCCCTGGGCACATACCACGATCTGCTGAAAATTAT
CAAGGACAAGGACTTCCTGGACAATGAGGAAAACGAGGACATTCTGGAAGATATCGTGCTGACCCTG
ACACTGTTTGAGGACAGAGAGATGATCGAGGAACGGCTGAAAACCTATGCCCACCTGTTCGACGACA
AAGTGATGAAGCAGCTGAAGCGGCTGAGATACACCGGCTGGGGCAGGCTGAGCCGGAAGCTGATCA
ACGGCATCCGGGACAAGCAGTCCGGCAAGACAATCCTGGATTTCCTGAAGTCCGACGGCTTCGCCA
ACAGAAACTTCATGCAGCTGATCCACGACGACAGCCTGACCTTTAAAGAGGACATCCAGAAAGCCCA
GGTGTCCGGCCAGGGCGATAGCCTGCACGAGCACATTGCCAATCTGGCCGGCAGCCCCGCCATTAA
GAAGGGCATCCTGCAGACAGTGAAGGTGGTGGACGAGCTCGTGAAAGTGATGGGCGGCCACAAGC
CCGAGAACATCGTGATCGAAATGGCCAGAGAGAACCAGACCACCCAGAAGGGACAGAAGAACAGCC
GCGAGAGAATGAAGCGGATCGAAGAGGGCATCAAAGAGCTGGGCAGCCAGATCCTGAAAGAACACC
CCGTGGAAAACACCCAGCTGCAGAACGAGAAGCTGTACCTGTACTACCTGCAGAATGGGCGGGATAT
GTACGTGGACCAGGAACTGGACATCAACCGGCTGTCCGACTACGATGTGGACCATATCGTGCCTCAG
AGCTTTCTGAAGGACGACTCCATCGACAACAAGGTGCTGACCAGAAGCGACAAGAACCGGGGCAAG
AGCGACAACGTGCCCTCCGAAGAGGTCGTGAAGAAGATGAAGAACTACTGGCGGCAGCTGCTGAAC
GCCAAGCTGATTACCCAGAGAAAGTTCGACAATCTGACCAAGGCCGAGAGAGGCGGCCTGAGCGAA
CTGGATAAGGCCGGCTTCATCAAGAGACAGCTGGTGGAAACCCGGCAGATCACAAAGCACGTGGCA
CAGATCCTGGACTCCCGGATGAACACTAAGTACGACGAGAATGACAAGCTGATCCGGGAAGTGAAAG
TGATCACCCTGAAGTCCAAGCTGGTGTCCGATTTCCGGAAGGATTTCCAGTTTTACAAAGTGCGCGA
GATCAACAACTACCACCACGCCCACGACGCCTACCTGAACGCCGTCGTGGGAACCGCCCTGATCAA
AAAGTACCCTAAGCTGGAAAGCGAGTTCGTGTACGGCGACTACAAGGTGTACGACGTGCGGAAGATG
ATCGCCAAGAGCGAGCAGGAAATCGGCAAGGCTACCGCCAAGTACTTCTTCTACAGCAACATCATGA
ACTTTTTCAAGACCGAGATTACCCTGGCCAACGGCGAGATCCGGAAGCGGCCTCTGATCGAGACAAA
CGGCGAAACCGGGGAGATCGTGTGGGATAAGGGCCGGGATTTTGCCACCGTGCGGAAAGTGCTGA
GCATGCCCCAAGTGAATATCGTGAAAAAGACCGAGGTGCAGACAGGCGGCTTCAGCAAAGAGTCTAT
CCTGCCCAAGGGTAACAGCGATAAGCTGATCGCCAGAAAGAAGGACTGGGACCCTAAGAAGTACGG
CGGCTTCAACAGCCCCACCGTGGCCTATTCTGTGCTGGTGGTGGCCAAAGTGGAAAAGGGCAAGTC
CAAGAAACTGAAGAGTGTGAAAGAGCTGCTGGGGATCACCATCATGGAAAGAAGCAGCTTCGAGAA
GAATCCCATCGACTTTCTGGAAGCCAAGGGCTACAAAGAAGTGAAAAAGGACCTGATCATCAAGCTG
CCTAAGTACTCCCTGTTCGAGCTGGAAAACGGCCGGAAGAGAATGCTGGCCTCTGCCGGCGTGCTG
CAGAAGGGAAACGAACTGGCCCTGCCCTCCAAATATGTGAACTTCCTGTACCTGGCCAGCCACTATG
AGAAGCTGAAGGGCTCCCCCGAGGATAATGAGCAGAAACAGCTGTTTGTGGAACAGCACAAGCACTA
CCTGGACGAGATCATCGAGCAGATCAGCGAGTTCTCCAAGAGAGTGATCCTGGCCGACGCTAATCTG
GACAAAGTGCTGTCCGCCTACAACAAGCACCGGGATAAGCCCATCAGAGAGCAGGCCGAGAATATCA
TCCACCTGTTTACCCTGACCAATCTGGGAGCCCCTGCCGCCTTCAAGTACTTTGACACCACCATCAAC
CGGAAGCAATACAACACGACCAAAGAGGTGCTGGACGCCACCCTGATCCGTCAGAGCATCACCGGC
CTGTACGAGACACGGATCGACCTGTCTCAGCTGGGAGGTGACTCTGGCGGCTCAAAAAGAACCGCC
GACGGCAGCGAATTCGAGCCCAAGAAGAAGAGGAAAGTCTAATTAATTAAGCTGCCTTCTGCGGGGC
TTGCCTTCTGGCCATGCCCTTCTTCTCTCCCTTGCACCTGTACCTCTTGGTCTTTGAATAAAGCCTGA
GTAGGAAGCGACTGTGCCTTCTAGTTGCCAGCCATCTGTTGTTTGCCCCTCCCCCGTGCCTTCCTTG
ACCCTGGAAGGTGCCACTCCCACTGTCCTTTCCTAATAAAATGAGAAATTGCATCGCATTGTCTGAG
TAGGTGTCATTCTATTCTGGGGGGTGGGGTGGGGCAGGACAGCAAGGGGGAGGATTGGGAAGACAA
TAGCAGGCATGCTGGGGATGCGGTGGGCTCTATGG

Start	End	Feature Description
1	380	CMV enhancer
381	619	CMV promoter
620	636	T7 promoter with mismatch
637	683	5' UTR

684	740	BP NLS
741	1238	TadA wild type
1239	1334	(SGGS)2 – XTEN – (SGGS)2 linker
1335	1832	Engineered TadA* variant 7.10
1833	1928	(SGGS)2 – XTEN – (SGGS)2 linker
1929	6029	Cas9(D10A) Sp-NRCH PAM variant
6030	6095	Linker + SV40 BP NLS
6096	6196	3' UTR
6197	6424	bGH poly(A) signal

Amino acid sequence

MKRTADGSEFESPKKKRKVSEVEFSHEYWMRHALTLAKRAWDEREVPVGAVLVHNNRVIGEGWNRPIG
RHDPTAHAEIMALRQGGLVMQNYRLIDATLYVTLEPCVMCAGAMIHSRIGRVVFGARDAKTGAAGSLMDV
LHHPGMNHRVEITEGILADECAALLSDFFRMRRQEIKAQKKAQSSTDSGGSSGGSSGSETPGTSESATP
ESSGGSSGGSSEVEFSHEYWMRHALTLAKRARDEREVPVGAVLVLNNRVIGEGWNRAIGLHDPTAHAEI
MALRQGGLVMQNYRLIDATLYVTFEPCVMCAGAMIHSRIGRVVFGVRNAKTGAAGSLMDVLHYPGMNHR
VEITEGILADECAALLCYFFRMPRQVFNAQKKAQSSTDSGGSSGGSSGSETPGTSESATPESSGGSSGG
SDKKYSIGLTIGTNSVGWAVITDEYKVPSKKFKVLGNTDRHSIKKNLIGALLFDSGETAEATRLKRTARRRYT
RRKNRICYLQEIFSNEMAKVDDSFFHRLEESFLVEEDKKHERHPIFGNIVDEVAYHEKYPTIYHLRKKLVDS
TDKADLRLIYLALAHMIKFRGHFLIEGDLNPDNSDVDKLFIQLVQTYNQLFEENPINASGVDAKAILSARLSK
SRRLENLIAQLPGEKKNGLFGNLIALSLGLTPNFKSNFDLAEDAKLQLSKDTYDDDLDNLLAQIGDQYADLF
LAAKNLSDAILLSDILRVNTEITKAPLSASMVKRYDEHHQDLTLLKALVRQQLPEKYKEIFFDQSKNGYAGYI
DGGASQEEFYKFIKPILEKMDGTEELLVKLNREDLLRKQRTFDNGIIPHQIHLGELHAILRRQGDFYPFLKD
NREKIEKILTFRIPYYVGPLARGNSRFAWMTRKSEETITPWNFEEVVDKGASAQSFIERMTNFDKNLPNEK
VLPKHSLLYEYFTVYNELTKVKYVTEGMRKPAFLSGEQKKAIVDLLFKTNRKVTVKQLKEDYFKKIECFDSV
EISGVEDRFNASLGTYHDLLKIIKDKDFLDNEENEDILEDIVLTLTLFEDREMIEERLKTYAHLFDDKVMKQLK
RLRYTGWGRLSRKLINGIRDKQSGKTILDFLKSDGFANRNFMQLIHDDSLTFKEDIQKAQVSGQGDSLHE
HIANLAGSPAIKKGILQTVKVVDELVKVMGGHKPENIVIEMARENQTTQKGQKNSRERMKRIEEGIKELGS
QILKEHPVENTQLQNEKLYLYYLQNGRDMYVDQELDINRLSDYDVDHIVPQSFLKDDSIDNKVLTRSDKNR
GKSDNVPSEEVVKKMKNYWRQLLNAKLITQRKFDNLTKAERGGLSELDKAGFIKRQLVETRQITKHVAQIL
DSRMNTKYDENDKLIREVKVITLKSKLVSDFRKDFQFYKVREINNYHHAHDAYLNAVVGTALIKKYPKLESE
FVYGDYKVYDVRKMIAKSEQEIGKATAKYFFYSNIMNFFKTEITLANGEIRKRPLIETNGETGEIVWDKGRD
FATVRKVLSMPQVNIVKKTEVQTGGFSKESILPKGNSDKLIARKKDWDPKKYGGFNSPTVAYSVLVVAKVE
KGKSKKLKSVKELLGITIMERSSFEKNPIDFLEAKGYKEVKKDLIIKLPKYSLFELENGRKRMLASAGVLQK
GNELALPSKYVNFLYLASHYEKLKGSPEDNEQKQLFVEQHKHYLDEIIEQISEFSKRVILADANLDKVLSAY
NKHRDKPIREQAENIIHLFTLTNLGAPAAFKYFDTTINRKQYNTTKEVLDATLIRQSITGLYETRIDLSQLGGD
SGGSKRTADGSEFEPKKKRKV

Start	End	Feature Description
1	19	BP NLS
20	185	TadA wild type
186	217	(SGGS)2 – XTEN – (SGGS)2 linker
218	383	Engineered TadA* variant 7.10

384	415	(SGGS)2 – XTEN – (SGGS)2 linker
416	1782	Cas9(D10T) Sp-NRCH PAM variant
1783	1803	Linker + SV40 BP NLS

ABE8e – Cas9 (D10T) – NRCH variant
Plasmid ID: pHS0384

DNA sequence
GACATTGATTATTGACTAGTTATTAATAGTAATCAATTACGGGGTCATTAGTTCATAGCCCATATATGGAG
TTCCGCGTTACATAACTTACGGTAAATGGCCCGCCTGGCTGACCGCCCAACGACCCCCGCCCATTGA
CGTCAATAATGACGTATGTTCCCATAGTAACGCCAATAGGGACTTTCCATTGACGTCAATGGGTGGAGT
ATTTACGGTAAACTGCCCACTTGGCAGTACATCAAGTGTATCATATGCCAAGTACGCCCCCTATTGACG
TCAATGACGGTAAATGGCCCGCCTGGCATTATGCCCAGTACATGACCTTATGGGACTTTCCTACTTGG
CAGTACATCTACGTATTAGTCATCGCTATTACCATGGTGATGCGGTTTTGGCAGTACATCAATGGGCGT
GGATAGCGGTTTGACTCACGGGGATTTCCAAGTCTCCACCCCATTGACGTCAATGGGAGTTTGTTTTG
GCACCAAAATCAACGGGACTTTCCAAAATGTCGTAACAACTCCGCCCCATTGACGCAAATGGGCGGT
AGGCGTGTACGGTGGGAGGTCTATATAAGCAGAGCTGGTTTAGTGAACCGTCAGATCTCGAGCTCGG
TACCTAATACGACACACTATAAGGAAATAAGAGAGAAAAGAAGAGTAAGAAGAAATATAAGAGCCACCA
TGAAACGGACAGCCGACGGAAGCGAGTTCGAGTCACCAAAGAAGAAGCGGAAAGTCTCTGAGGTG
GAGTTTTCCCACGAGTACTGGATGAGACATGCCCTGACCCTGGCCAAGAGGGCACGGGATGAGAGG
GAGGTGCCTGTGGGAGCCGTGCTGGTGCTGAACAATAGAGTGATCGGCGAGGGCTGGAACAGAGC
CATCGGCCTGCACGACCCAACAGCCCATGCCGAAATTATGGCCCTGAGACAGGGCGGCCTGGTCAT
GCAGAACTACAGACTGATTGACGCCACCCTGTACGTGACATTCGAGCCTTGCGTGATGTGCGCCGGC
GCCATGATCCACTCTAGGATCGGCCGCGTGGTGTTTGGCGTGAGGAACTCAAAAAGAGGCGCCGCA
GGCTCCCTGATGAACGTGCTGAACTACCCCGGCATGAATCACCGCGTCGAAATTACCGAGGGAATCC
TGGCAGATGAATGTGCCGCCCTGCTGTGCGATTTCTATCGGATGCCTAGACAGGTGTTCAATGCTCAG
AAGAAGGCCCAGAGCTCCATCAACTCCGGAGGATCTAGCGGAGGCTCCTCTGGCTCTGAGACACCT
GGCACAAGCGAGAGCGCAACACCTGAAAGCAGCGGGGGCAGCAGCGGGGGGTCAGACAAGAAGT
ACAGCATCGGCCTGACCATCGGCACCAACTCTGTGGGCTGGGCCGTGATCACCGACGAGTACAAGG
TGCCCAGCAAGAAATTCAAGGTGCTGGGCAACACCGACCGGCACAGCATCAAGAAGAACCTGATCG
GAGCCCTGCTGTTCGACAGCGGCGAAACAGCCGAGGCCACCCGGCTGAAGAGAACCGCCAGAAGA
AGATACACCAGACGGAAGAACCGGATCTGCTATCTGCAAGAGATCTTCAGCAACGAGATGGCCAAGG
TGGACGACAGCTTCTTCCACAGACTGGAAGAGTCCTTCCTGGTGGAAGAGGATAAGAAGCACGAGC
GGCACCCCATCTTCGGCAACATCGTGGACGAGGTGGCCTACCACGAGAAGTACCCCACCATCTACCA
CCTGAGAAAGAAACTGGTGGACAGCACCGACAAGGCCGACCTGCGGCTGATCTATCTGGCCCTGGC
CCACATGATCAAGTTCCGGGGCCACTTCCTGATCGAGGGCGACCTGAACCCCGACAACAGCGACGT
GGACAAGCTGTTCATCCAGCTGGTGCAGACCTACAACCAGCTGTTCGAGGAAAACCCCATCAACGCC
AGCGGCGTGGACGCCAAGGCCATCCTGTCTGCCAGACTGAGCAAGAGCAGACGGCTGGAAAATCTG
ATCGCCCAGCTGCCCGGCGAGAAGAAGAATGGCCTGTTCGGAAACCTGATTGCCCTGAGCCTGGGC
CTGACCCCCAACTTCAAGAGCAACTTCGACCTGGCCGAGGATGCCAAACTGCAGCTGAGCAAGGAC
ACCTACGACGACGACCTGGACAACCTGCTGGCCCAGATCGGCGACCAGTACGCCGACCTGTTTCTG
GCCGCCAAGAACCTGTCCGACGCCATCCTGCTGAGCGACATCCTGAGAGTGAACACCGAGATCACC
AAGGCCCCCCTGAGCGCCTCTATGGTGAAGAGATACGACGAGCACCACCAGGACCTGACCCTGCTG
AAAGCTCTCGTGCGGCAGCAGCTGCCTGAGAAGTACAAAGAGATTTTCTTCGACCAGAGCAAGAACG
GCTACGCCGGCTACATTGACGGCGGAGCCAGCCAGGAAGAGTTCTACAAGTTCATCAAGCCCATCCT
GGAAAAGATGGACGGCACCGAGGAACTGCTCGTGAAGCTGAACAGAGAGGACCTGCTGCGGAAGC
AGCGGACCTTCGACAACGGCATTATCCCCCACCAGATCCACCTGGGAGAGCTGCACGCCATTCTGC
GGCGGCAGGGCGATTTTTACCCATTCCTGAAGGACAACCGGGAAAAGATCGAGAAGATCCTGACCTT
CCGCATCCCCTACTACGTGGGCCCTCTGGCCAGGGGAAACAGCAGATTCGCCTGGATGACCAGAAA
GAGCGAGGAAACCATCACCCCCTGGAACTTCGAGGAAGTGGTGGACAAGGGCGCTTCCGCCCAGA
GCTTCATCGAGCGGATGACCAACTTCGATAAGAACCTGCCCAACGAGAAGGTGCTGCCCAAGCACA
GCCTGCTGTACGAGTACTTCACCGTGTATAACGAGCTGACCAAAGTGAAATACGTGACCGAGGGAAT
GAGAAAGCCCGCCTTCCTGAGCGGCGAGCAGAAAAAGGCCATCGTGGACCTGCTGTTCAAGACCAA

CCGGAAAGTGACCGTGAAGCAGCTGAAAGAGGACTACTTCAAGAAAATCGAGTGCTTCGACTCCGTG
GAAATCTCCGGCGTGGAAGATCGGTTCAACGCCTCCCTGGGCACATACCACGATCTGCTGAAAATTAT
CAAGGACAAGGACTTCCTGGACAATGAGGAAAACGAGGACATTCTGGAAGATATCGTGCTGACCCTG
ACACTGTTTGAGGACAGAGAGATGATCGAGGAACGGCTGAAAACCTATGCCCACCTGTTCGACGACA
AAGTGATGAAGCAGCTGAAGCGGCTGAGATACACCGGCTGGGGCAGGCTGAGCCGGAAGCTGATCA
ACGGCATCCGGGACAAGCAGTCCGGCAAGACAATCCTGGATTTCCTGAAGTCCGACGGCTTCGCCA
ACAGAAACTTCATGCAGCTGATCCACGACGACAGCCTGACCTTTAAAGAGGACATCCAGAAAGCCCA
GGTGTCCGGCCAGGGCGATAGCCTGCACGAGCACATTGCCAATCTGGCCGGCAGCCCCGCCATTAA
GAAGGGCATCCTGCAGACAGTGAAGGTGGTGGACGAGCTCGTGAAAGTGATGGGCGGCCACAAGC
CCGAGAACATCGTGATCGAAATGGCCAGAGAGAACCAGACCACCCAGAAGGGACAGAAGAACAGCC
GCGAGAGAATGAAGCGGATCGAAGAGGGCATCAAAGAGCTGGGCAGCCAGATCCTGAAAGAACACC
CCGTGGAAAACACCCAGCTGCAGAACGAGAAGCTGTACCTGTACTACCTGCAGAATGGGCGGGATAT
GTACGTGGACCAGGAACTGGACATCAACCGGCTGTCCGACTACGATGTGGACCATATCGTGCCTCAG
AGCTTTCTGAAGGACGACTCCATCGACAACAAGGTGCTGACCAGAAGCGACAAGAACCGGGGCAAG
AGCGACAACGTGCCCTCCGAAGAGGTCGTGAAGAAGATGAAGAACTACTGGCGGCAGCTGCTGAAC
GCCAAGCTGATTACCCAGAGAAAGTTCGACAATCTGACCAAGGCCGAGAGAGGCGGCCTGAGCGAA
CTGGATAAGGCCGGCTTCATCAAGAGACAGCTGGTGGAAACCCGGCAGATCACAAAGCACGTGGCA
CAGATCCTGGACTCCCGGATGAACACTAAGTACGACGAGAATGACAAGCTGATCCGGGAAGTGAAAG
TGATCACCCTGAAGTCCAAGCTGGTGTCCGATTTCCGGAAGGATTTCCAGTTTTACAAAGTGCGCGA
GATCAACAACTACCACCACGCCCACGACGCCTACCTGAACGCCGTCGTGGGAACCGCCCTGATCAA
AAAGTACCCTAAGCTGGAAAGCGAGTTCGTGTACGGCGACTACAAGGTGTACGACGTGCGGAAGATG
ATCGCCAAGAGCGAGCAGGAAATCGGCAAGGCTACCGCCAAGTACTTCTTCTACAGCAACATCATGA
ACTTTTTCAAGACCGAGATTACCCTGGCCAACGGCGAGATCCGGAAGCGGCCTCTGATCGAGACAAA
CGGCGAAACCGGGGAGATCGTGTGGGATAAGGGCCGGGATTTTGCCACCGTGCGGAAAGTGCTGA
GCATGCCCCAAGTGAATATCGTGAAAAAGACCGAGGTGCAGACAGGCGGCTTCAGCAAAGAGTCTAT
CCTGCCCAAGGGTAACAGCGATAAGCTGATCGCCAGAAAGAAGGACTGGGACCCTAAGAAGTACGG
CGGCTTCAACAGCCCCACCGTGGCCTATTCTGTGCTGGTGGTGGCCAAAGTGGAAAAGGGCAAGTC
CAAGAAACTGAAGAGTGTGAAAGAGCTGCTGGGGATCACCATCATGGAAAGAAGCAGCTTCGAGAA
GAATCCCATCGACTTTCTGGAAGCCAAGGGCTACAAAGAAGTGAAAAAGGACCTGATCATCAAGCTG
CCTAAGTACTCCCTGTTCGAGCTGGAAAACGGCCGGAAGAGAATGCTGGCCTCTGCCGGCGTGCTG
CAGAAGGGAAACGAACTGGCCCTGCCCTCCAAATATGTGAACTTCCTGTACCTGGCCAGCCACTATG
AGAAGCTGAAGGGCTCCCCCGAGGATAATGAGCAGAAACAGCTGTTTGTGGAACAGCACAAGCACTA
CCTGGACGAGATCATCGAGCAGATCAGCGAGTTCTCCAAGAGAGTGATCCTGGCCGACGCTAATCTG
GACAAAGTGCTGTCCGCCTACAACAAGCACCGGGATAAGCCCATCAGAGAGCAGGCCGAGAATATCA
TCCACCTGTTTACCCTGACCAATCTGGGAGCCCCTGCCGCCTTCAAGTACTTTGACACCACCATCAAC
CGGAAGCAATACAACACGACCAAAGAGGTGCTGGACGCCACCCTGATCCGTCAGAGCATCACCGGC
CTGTACGAGACACGGATCGACCTGTCTCAGCTGGGAGGTGACTCTGGCGGCTCAAAAAGAACCGCC
GACGGCAGCGAATTCGAGCCCAAGAAGAAGAGGAAAGTCTAATTAATTAAGCTGCCTTCTGCGGGGC
TTGCCTTCTGGCCATGCCCTTCTTCTCTCCCTTGCACCTGTACCTCTTGGTCTTTGAATAAAGCCTGA
GTAGGAAGCGACTGTGCCTTCTAGTTGCCAGCCATCTGTTGTTTGCCCCTCCCCCGTGCCTTCCTTG
ACCCTGGAAGGTGCCACTCCCACTGTCCTTTCCTAATAAAATGAGAAAATTGCATCGCATTGTCTGAG
TAGGTGTCATTCTATTCTGGGGGGTGGGGTGGGGCAGGACAGCAAGGGGGAGGATTGGGAAGACAA
TAGCAGGCATGCTGGGGATGCGGTGGGCTCTATGG

Start	End	Feature Description
1	380	CMV enhancer
381	619	CMV promoter
620	636	T7 promoter with mismatch
637	683	5' UTR
684	740	BP NLS

741	1238	Engineered TadA* variant 8e
1239	1334	(SGGS)2 – XTEN – (SGGS)2 linker
1335	5435	Cas9(D10T) Sp-NRCH PAM variant
5436	5501	Linker + SV40 BP NLS
5502	5605	3' UTR
5606	5830	bGH poly(A) signal

Amino acid sequence

MKRTADGSEFESPKKKRKVSEVEFSHEYWMRHALTLAKRARDEREVPVGAVLVLNNRVIGEGWNRAIGL
HDPTAHAEIMALRQGGLVMQNYRLIDATLYVTFEPCVMCAGAMIHSRIGRVVFGVRNSKRGAAGSLMNVL
NYPGMNHRVEITEGILADECAALLCDFYRMPRQVFNAQKKAQSSINSGGSSGGSSGSETPGTSESATPE
SSGGSSGGSDKKYSIGLTIGTNSVGWAVITDEYKVPSKKFKVLGNTDRHSIKKNLIGALLFDSGETAEATRL
KRTARRRYTRRKNRICYLQEIFSNEMAKVDDSFFHRLEESFLVEEDKKHERHPIFGNIVDEVAYHEKYPTIY
HLRKKLVDSTDKADLRLIYLALAHMIKFRGHFLIEGDLNPDNSDVDKLFIQLVQTYNQLFEENPINASGVDA
KAILSARLSKSRRLENLIAQLPGEKKNGLFGNLIALSLGLTPNFKSNFDLAEDAKLQLSKDTYDDDLDNLLA
QIGDQYADLFLAAKNLSDAILLSDILRVNTEITKAPLSASMVKRYDEHHQDLTLLKALVRQQLPEKYKEIFFD
QSKNGYAGYIDGGASQEEFYKFIKPILEKMDGTEELLVKLNREDLLRKQRTFDNGIIPHQIHLGELHAILRR
QGDFYPFLKDNREKIEKILTFRIPYYVGPLARGNSRFAWMTRKSEETITPWNFEEVVDKGASAQSFIERMT
NFDKNLPNEKVLPKHSLLYEYFTVYNELTKVKYVTEGMRKPAFLSGEQKKAIVDLLFKTNRKVTVKQLKED
YFKKIECFDSVEISGVEDRFNASLGTYHDLLKIIKDKDFLDNEENEDILEDIVLTLTLFEDREMIEERLKTYAH
LFDDKVMKQLKRLRYTGWGRLSRKLINGIRDKQSGKTILDFLKSDGFANRNFMQLIHDDSLTFKEDIQKAQ
VSGQGDSLHEHIANLAGSPAIKKGILQTVKVVDELVKVMGGHKPENIVIEMARENQTTQKGQKNSRERMK
RIEEGIKELGSQILKEHPVENTQLQNEKLYLYYLQNGRDMYVDQELDINRLSDYDVDHIVPQSFLKDDSIDN
KVLTRSDKNRGKSDNVPSEEVVKKMKNYWRQLLNAKLITQRKFDNLTKAERGGLSELDKAGFIKRQLVET
RQITKHVAQILDSRMNTKYDENDKLIREVKVITLKSKLVSDFRKDFQFYKVREINNYHHAHDAYLNAVVGTA
LIKKYPKLESEFVYGDYKVYDVRKMIAKSEQEIGKATAKYFFYSNIMNFFKTEITLANGEIRKRPLIETNGET
GEIVWDKGRDFATVRKVLSMPQVNIVKKTEVQTGGFSKESILPKGNSDKLIARKKDWDPKKYGGFNSPTV
AYSVLVVAKVEKGKSKKLKSVKELLGITIMERSSFEKNPIDFLEAKGYKEVKKDLIIKLPKYSLFELENGRKR
MLASAGVLQKGNELALPSKYVNFLYLASHYEKLKGSPEDNEQKQLFVEQHKHYLDEIIEQISEFSKRVILAD
ANLDKVLSAYNKHRDKPIREQAENIIHLFTLTNLGAPAAFKYFDTTINRKQYNTTKEVLDATLIRQSITGLYET
RIDLSQLGGDSGGSKRTADGSEFEPKKKRKV

Start	End	Feature Description
1	19	BP NLS
20	185	Engineered TadA* variant 8e
186	217	(SGGS)2 – XTEN – (SGGS)2 linker
218	1584	Cas9(D10T) Sp-NRCH PAM variant
1585	1605	Linker + SV40 BP NLS

ABE8e(V106W) – Cas9 (D10T) – NRCH variant
Plasmid ID: pHS0377

DNA sequence
GACATTGATTATTGACTAGTTATTAATAGTAATCAATTACGGGGTCATTAGTTCATAGCCCATATATGGAG
TTCCGCGTTACATAACTTACGGTAAATGGCCCGCCTGGCTGACCGCCCAACGACCCCCGCCCATTGA
CGTCAATAATGACGTATGTTCCCATAGTAACGCCAATAGGGACTTTCCATTGACGTCAATGGGTGGAGT
ATTTACGGTAAACTGCCCACTTGGCAGTACATCAAGTGTATCATATGCCAAGTACGCCCCCTATTGACG
TCAATGACGGTAAATGGCCCGCCTGGCATTATGCCCAGTACATGACCTTATGGGACTTTCCTACTTGG
CAGTACATCTACGTATTAGTCATCGCTATTACCATGGTGATGCGGTTTTGGCAGTACATCAATGGGCGT
GGATAGCGGTTTGACTCACGGGGATTTCCAAGTCTCCACCCCATTGACGTCAATGGGAGTTTGTTTTG
GCACCAAAATCAACGGGACTTTCCAAAATGTCGTAACAACTCCGCCCCATTGACGCAAATGGGCGGT
AGGCGTGTACGGTGGGAGGTCTATATAAGCAGAGCTGGTTTAGTGAACCGTCAGATCTCGAGCTCGG
TACCTAATACGACACACTATAAGGAAATAAGAGAGAAAAGAAGAGTAAGAAGAAATATAAGAGCCACCA
TGAAACGGACAGCCGACGGAAGCGAGTTCGAGTCACCAAAGAAGAAGCGGAAAGTCTCTGAGGTG
GAGTTTTCCCACGAGTACTGGATGAGACATGCCCTGACCCTGGCCAAGAGGGCACGGGATGAGAGG
GAGGTGCCTGTGGGAGCCGTGCTGGTGCTGAACAATAGAGTGATCGGCGAGGGCTGGAACAGAGC
CATCGGCCTGCACGACCCAACAGCCCATGCCGAAATTATGGCCCTGAGACAGGGCGGCCTGGTCAT
GCAGAACTACAGACTGATTGACGCCACCCTGTACGTGACATTCGAGCCTTGCGTGATGTGCGCCGGC
GCCATGATCCACTCTAGGATCGGCCGCGTGGTGTTTGGATGGAGAAATTCTAAAAGAGGCGCCGCAG
GCTCCCTGATGAACGTGCTGAACTACCCCGGCATGAATCACCGCGTCGAAATTACCGAGGGAATCCT
GGCAGATGAATGTGCCGCCCTGCTGTGCGATTTCTATCGGATGCCTAGACAGGTGTTCAATGCTCAG
AAGAAGGCCCAGAGCTCCATCAACTCCGGAGGATCTAGCGGAGGCTCCTCTGGCTCTGAGACACCT
GGCACAAGCGAGAGCGCAACACCTGAAAGCAGCGGGGGCAGCAGCGGGGGGTCAGACAAGAAGT
ACAGCATCGGCCTGACCATCGGCACCAACTCTGTGGGCTGGGCCGTGATCACCGACGAGTACAAGG
TGCCCAGCAAGAAATTCAAGGTGCTGGGCAACACCGACCGGCACAGCATCAAGAAGAACCTGATCG
GAGCCCTGCTGTTCGACAGCGGCGAAACAGCCGAGGCCACCCGGCTGAAGAGAACCGCCAGAAGA
AGATACACCAGACGGAAGAACCGGATCTGCTATCTGCAAGAGATCTTCAGCAACGAGATGGCCAAGG
TGGACGACAGCTTCTTCCACAGACTGGAAGAGTCCTTCCTGGTGGAAGAGGATAAGAAGCACGAGC
GGCACCCCATCTTCGGCAACATCGTGGACGAGGTGGCCTACCACGAGAAGTACCCCACCATCTACCA
CCTGAGAAAGAAACTGGTGGACAGCACCGACAAGGCCGACCTGCGGCTGATCTATCTGGCCCTGGC
CCACATGATCAAGTTCCGGGGCCACTTCCTGATCGAGGGCGACCTGAACCCCGACAACAGCGACGT
GGACAAGCTGTTCATCCAGCTGGTGCAGACCTACAACCAGCTGTTCGAGGAAAACCCCATCAACGCC
AGCGGCGTGGACGCCAAGGCCATCCTGTCTGCCAGACTGAGCAAGAGCAGACGGCTGGAAAATCTG
ATCGCCCAGCTGCCCGGCGAGAAGAAGAATGGCCTGTTCGGAAACCTGATTGCCCTGAGCCTGGGC
CTGACCCCCAACTTCAAGAGCAACTTCGACCTGGCCGAGGATGCCAAACTGCAGCTGAGCAAGGAC
ACCTACGACGACGACCTGGACAACCTGCTGGCCCAGATCGGCGACCAGTACGCCGACCTGTTTCTG
GCCGCCAAGAACCTGTCCGACGCCATCCTGCTGAGCGACATCCTGAGAGTGAACACCGAGATCACC
AAGGCCCCCCTGAGCGCCTCTATGGTGAAGAGATACGACGAGCACCACCAGGACCTGACCCTGCTG
AAAGCTCTCGTGCGGCAGCAGCTGCCTGAGAAGTACAAAGAGATTTTCTTCGACCAGAGCAAGAACG
GCTACGCCGGCTACATTGACGGCGGAGCCAGCCAGGAAGAGTTCTACAAGTTCATCAAGCCCATCCT
GGAAAAGATGGACGGCACCGAGGAACTGCTCGTGAAGCTGAACAGAGAGGACCTGCTGCGGAAGC
AGCGGACCTTCGACAACGGCATTATCCCCCACCAGATCCACCTGGGAGAGCTGCACGCCATTCTGC
GGCGGCAGGGCGATTTTTACCCATTCCTGAAGGACAACCGGGAAAAGATCGAGAAGATCCTGACCTT
CCGCATCCCCTACTACGTGGGCCCTCTGGCCAGGGGAAACAGCAGATTCGCCTGGATGACCAGAAA
GAGCGAGGAAACCATCACCCCCTGGAACTTCGAGGAAGTGGTGGACAAGGGCGCTTCCGCCCAGA
GCTTCATCGAGCGGATGACCAACTTCGATAAGAACCTGCCCAACGAGAAGGTGCTGCCCAAGCACA
GCCTGCTGTACGAGTACTTCACCGTGTATAACGAGCTGACCAAAGTGAAATACGTGACCGAGGGAAT
GAGAAAGCCCGCCTTCCTGAGCGGCGAGCAGAAAAAGGCCATCGTGGACCTGCTGTTCAAGACCAA
CCGGAAAGTGACCGTGAAGCAGCTGAAAGAGGACTACTTCAAGAAAATCGAGTGCTTCGACTCCGTG
GAAATCTCCGGCGTGGAAGATCGGTTCAACGCCTCCCTGGGCACATACCACGATCTGCTGAAAATTAT
CAAGGACAAGGACTTCCTGGACAATGAGGAAAACGAGGACATTCTGGAAGATATCGTGCTGACCCTG
ACACTGTTTGAGGACAGAGAGATGATCGAGGAACGGCTGAAAACCTATGCCCACCTGTTCGACGACA
AAGTGATGAAGCAGCTGAAGCGGCTGAGATACACCGGCTGGGGCAGGCTGAGCCGGAAGCTGATCA
ACGGCATCCGGGACAAGCAGTCCGGCAAGACAATCCTGGATTTCCTGAAGTCCGACGGCTTCGCCA
ACAGAAACTTCATGCAGCTGATCCACGACGACAGCCTGACCTTTAAAGAGGACATCCAGAAAGCCCA

GGTGTCCGGCCAGGGCGATAGCCTGCACGAGCACATTGCCAATCTGGCCGGCAGCCCCGCCATTAA
GAAGGGCATCCTGCAGACAGTGAAGGTGGTGGACGAGCTCGTGAAAGTGATGGGCGGCCACAAGC
CCGAGAACATCGTGATCGAAATGGCCAGAGAGAACCAGACCACCCAGAAGGGACAGAAGAACAGCC
GCGAGAGAATGAAGCGGATCGAAGAGGGCATCAAAGAGCTGGGCAGCCAGATCCTGAAAGAACACC
CCGTGGAAAACACCCAGCTGCAGAACGAGAAGCTGTACCTGTACTACCTGCAGAATGGGCGGGATAT
GTACGTGGACCAGGAACTGGACATCAACCGGCTGTCCGACTACGATGTGGACCATATCGTGCCTCAG
AGCTTTCTGAAGGACGACTCCATCGACAACAAGGTGCTGACCAGAAGCGACAAGAACCGGGGCAAG
AGCGACAACGTGCCCTCCGAAGAGGTCGTGAAGAAGATGAAGAACTACTGGCGGCAGCTGCTGAAC
GCCAAGCTGATTACCCAGAGAAAGTTCGACAATCTGACCAAGGCCGAGAGAGGCGGCCTGAGCGAA
CTGGATAAGGCCGGCTTCATCAAGAGACAGCTGGTGGAAACCCGGCAGATCACAAAGCACGTGGCA
CAGATCCTGGACTCCCGGATGAACACTAAGTACGACGAGAATGACAAGCTGATCCGGGAAGTGAAAG
TGATCACCCTGAAGTCCAAGCTGGTGTCCGATTTCCGGAAGGATTTCCAGTTTTACAAAGTGCGCGA
GATCAACAACTACCACCACGCCCACGACGCCTACCTGAACGCCGTCGTGGGAACCGCCCTGATCAA
AAAGTACCCTAAGCTGGAAAGCGAGTTCGTGTACGGCGACTACAAGGTGTACGACGTGCGGAAGATG
ATCGCCAAGAGCGAGCAGGAAATCGGCAAGGCTACCGCCAAGTACTTCTTCTACAGCAACATCATGA
ACTTTTTCAAGACCGAGATTACCCTGGCCAACGGCGAGATCCGGAAGCGGCCTCTGATCGAGACAAA
CGGCGAAACCGGGGAGATCGTGTGGGATAAGGGCCGGGATTTTGCCACCGTGCGGAAAGTGCTGA
GCATGCCCCAAGTGAATATCGTGAAAAAGACCGAGGTGCAGACAGGCGGCTTCAGCAAAGAGTCTAT
CCTGCCCAAGGGTAACAGCGATAAGCTGATCGCCAGAAAGAAGGACTGGGACCCTAAGAAGTACGG
CGGCTTCAACAGCCCCACCGTGGCCTATTCTGTGCTGGTGGTGGCCAAAGTGGAAAAGGGCAAGTC
CAAGAAACTGAAGAGTGTGAAAGAGCTGCTGGGGATCACCATCATGGAAAGAAGCAGCTTCGAGAA
GAATCCCATCGACTTTCTGGAAGCCAAGGGCTACAAAGAAGTGAAAAAGGACCTGATCATCAAGCTG
CCTAAGTACTCCCTGTTCGAGCTGGAAAACGGCCGGAAGAGAATGCTGGCCTCTGCCGGCGTGCTG
CAGAAGGGAAACGAACTGGCCCTGCCCTCCAAATATGTGAACTTCCTGTACCTGGCCAGCCACTATG
AGAAGCTGAAGGGCTCCCCCGAGGATAATGAGCAGAAACAGCTGTTTGTGGAACAGCACAAGCACTA
CCTGGACGAGATCATCGAGCAGATCAGCGAGTTCTCCAAGAGAGTGATCCTGGCCGACGCTAATCTG
GACAAAGTGCTGTCCGCCTACAACAAGCACCGGGATAAGCCCATCAGAGAGCAGGCCGAGAATATCA
TCCACCTGTTTACCCTGACCAATCTGGGAGCCCCTGCCGCCTTCAAGTACTTTGACACCACCATCAAC
CGGAAGCAATACAACACGACCAAAGAGGTGCTGGACGCCACCCTGATCCGTCAGAGCATCACCGGC
CTGTACGAGACACGGATCGACCTGTCTCAGCTGGGAGGTGACTCTGGCGGCTCAAAAAGAACCGCC
GACGGCAGCGAATTCGAGCCCAAGAAGAAGAGGAAAGTCTAATTAATTAAGCTGCCTTCTGCGGGGC
TTGCCTTCTGGCCATGCCCTTCTTCTCTCCCTTGCACCTGTACCTCTTGGTCTTTGAATAAAGCCTGA
GTAGGAAGCGACTGTGCCTTCTAGTTGCCAGCCATCTGTTGTTTGCCCCTCCCCCGTGCCTTCCTTG
ACCCTGGAAGGTGCCACTCCCACTGTCCTTTCCTAATAAAATGAGAAAATTGCATCGCATTGTCTGAG
TAGGTGTCATTCTATTCTGGGGGGTGGGGTGGGGCAGGACAGCAAGGGGGAGGATTGGGAAGACAA
TAGCAGGCATGCTGGGGATGCGGTGGGCTCTATGG

Start	End	Feature Description
1	380	CMV enhancer
381	619	CMV promoter
620	636	T7 promoter with mismatch
637	683	5' UTR
684	740	BP NLS
741	1238	Engineered TadA* variant 8e(V106W)
1239	1334	(SGGS)2 – XTEN – (SGGS)2 linker
1335	5435	Cas9(D10T) Sp-NRCH PAM variant
5436	5501	Linker + SV40 BP NLS

5502	5605	3’ UTR
5606	5830	bGH poly(A) signal

Amino acid sequence

MKRTADGSEFESPKKKRKVSEVEFSHEYWMRHALTLAKRARDEREVPVGAVLVLNNRVIGEGWNRAIGL
HDPTAHAEIMALRQGGLVMQNYRLIDATLYVTFEPCVMCAGAMIHSRIGRVVFGWRNSKRGAAGSLMNV
LNYPGMNHRVEITEGILADECAALLCDFYRMPRQVFNAQKKAQSSINSGGSSGGSSGSETPGTSESATP
ESSGGSSGGSDKKYSIGLTIGTNSVGWAVITDEYKVPSKKFKVLGNTDRHSIKKNLIGALLFDSGETAEATR
LKRTARRRYTRRKNRICYLQEIFSNEMAKVDDSFFHRLEESFLVEEDKKHERHPIFGNIVDEVAYHEKYPTI
YHLRKKLVDSTDKADLRLIYLALAHMIKFRGHFLIEGDLNPDNSDVDKLFIQLVQTYNQLFEENPINASGVD
AKAILSARLSKSRRLENLIAQLPGEKKNGLFGNLIALSLGLTPNFKSNFDLAEDAKLQLSKDTYDDDLDNLL
AQIGDQYADLFLAAKNLSDAILLSDILRVNTEITKAPLSASMVKRYDEHHQDLTLLKALVRQQLPEKYKEIFF
DQSKNGYAGYIDGGASQEEFYKFIKPILEKMDGTEELLVKLNREDLLRKQRTFDNGIIPHQIHLGELHAILRR
QGDFYPFLKDNREKIEKILTFRIPYYVGPLARGNSRFAWMTRKSEETITPWNFEEVVDKGASAQSFIERMT
NFDKNLPNEKVLPKHSLLYEYFTVYNELTKVKYVTEGMRKPAFLSGEQKKAIVDLLFKTNRKVTVKQLKED
YFKKIECFDSVEISGVEDRFNASLGTYHDLLKIIKDKDFLDNEENEDILEDIVLTLTLFEDREMIEERLKTYAH
LFDDKVMKQLKRLRYTGWGRLSRKLINGIRDKQSGKTILDFLKSDGFANRNFMQLIHDDSLTFKEDIQKAQ
VSGQGDSLHEHIANLAGSPAIKKGILQTVKVVDELVKVMGGHKPENIVIEMARENQTTQKGQKNSRERMK
RIEEGIKELGSQILKEHPVENTQLQNEKLYLYYLQNGRDMYVDQELDINRLSDYDVDHIVPQSFLKDDSIDN
KVLTRSDKNRGKSDNVPSEEVVKKMKNYWRQLLNAKLITQRKFDNLTKAERGGLSELDKAGFIKRQLVET
RQITKHVAQILDSRMNTKYDENDKLIREVKVITLKSKLVSDFRKDFQFYKVREINNYHHAHDAYLNAVVGTA
LIKKYPKLESEFVYGDYKVYDVRKMIAKSEQEIGKATAKYFFYSNIMNFFKTEITLANGEIRKRPLIETNGET
GEIVWDKGRDFATVRKVLSMPQVNIVKKTEVQTGGFSKESILPKGNSDKLIARKKDWDPKKYGGFNSPTV
AYSVLVVAKVEKGKSKKLKSVKELLGITIMERSSFEKNPIDFLEAKGYKEVKKDLIIKLPKYSLFELENGRKR
MLASAGVLQKGNELALPSKYVNFLYLASHYEKLKGSPEDNEQKQLFVEQHKHYLDEIIEQISEFSKRVILAD
ANLDKVLSAYNKHRDKPIREQAENIIHLFTLTNLGAPAAFKYFDTTINRKQYNTTKEVLDATLIRQSITGLYET
RIDLSQLGGDSGGSKRTADGSEFEPKKKRKV

Start	End	Feature Description
1	19	BP NLS
20	185	Engineered TadA* variant 8e
186	217	(SGGS)2 – XTEN – (SGGS)2 linker
218	1584	Cas9(D10T) Sp-NRCH PAM variant
1585	1605	Linker + SV40 BP NLS

ABE9 – Cas9 (D10T) – NRCH variant
Plasmid ID: pHS0411

DNA sequence

GACATTGATTATTGACTAGTTATTAATAGTAATCAATTACGGGGTCATTAGTTCATAGCCCATATATGGAG
TTCCGCGTTACATAACTTACGGTAAATGGCCCGCCTGGCTGACCGCCCAACGACCCCCGCCCATTGA
CGTCAATAATGACGTATGTTCCCATAGTAACGCCAATAGGGACTTTCCATTGACGTCAATGGGTGGAGT
ATTTACGGTAAACTGCCCACTTGGCAGTACATCAAGTGTATCATATGCCAAGTACGCCCCCTATTGACG
TCAATGACGGTAAATGGCCCGCCTGGCATTATGCCCAGTACATGACCTTATGGGACTTTCCTACTTGG
CAGTACATCTACGTATTAGTCATCGCTATTACCATGGTGATGCGGTTTTGGCAGTACATCAATGGGCGT
GGATAGCGGTTTGACTCACGGGGATTTCCAAGTCTCCACCCCATTGACGTCAATGGGAGTTTGTTTTG
GCACCAAAATCAACGGGACTTTCCAAAATGTCGTAACAACTCCGCCCCATTGACGCAAATGGGCGGT
AGGCGTGTACGGTGGGAGGTCTATATAAGCAGAGCTGGTTTAGTGAACCGTCAGATCTCGAGCTCGG
TACCTAATACGACACACTATAAGGAAATAAGAGAGAAAAGAAGAGTAAGAAGAAATATAAGAGCCACCA
TGAAACGGACAGCCGACGGAAGCGAGTTCGAGTCACCAAAGAAGAAGCGGAAAGTCTCTGAGGTG
GAGTTTTCCCACGAGTACTGGATGAGACATGCCCTGACCCTGGCCAAGAGGGCACGGGATGAGAGG
GAGGTGCCTGTGGGAGCCGTGCTGGTGCTGAACAATAGAGTGATCGGCGAGGGCTGGAACAGAGC
CATCGGCCTGCACGACCCAACAGCCCATGCCGAAATTATGGCCCTGAGACAGGGCGGCCTGGTCAT
GCAGAACTACAGACTGATTGACGCCACCCTGTACGTGACATTCGAGCCTTGCGTGATGTGCGCCGGC
GCCATGATCCACTCTAGGATCGGCCGCGTGGTGTTTGGCGTGAGGCAGTCAAAAAGAGGCGCCGCA
GGCTCCCTGATGAACGTGCTGAACTACCCCGGCATGAATCACCGCGTCGAAATTACCGAGGGAATCC
TGGCAGATGAATGTGCCGCCCTGACCTGCGATTTCTATCGGATGCCTAGACAGGTGTTCAATGCTCAG
AAGAAGGCCCAGAGCTCCATCAACTCCGGAGGATCTAGCGGAGGCTCCTCTGGCTCTGAGACACCT
GGCACAAGCGAGAGCGCAACACCTGAAAGCAGCGGGGGCAGCAGCGGGGGGTCAGACAAGAAGT
ACAGCATCGGCCTGACCATCGGCACCAACTCTGTGGGCTGGGCCGTGATCACCGACGAGTACAAGG
TGCCCAGCAAGAAATTCAAGGTGCTGGGCAACACCGACCGGCACAGCATCAAGAAGAACCTGATCG
GAGCCCTGCTGTTCGACAGCGGCGAAACAGCCGAGGCCACCCGGCTGAAGAGAACCGCCAGAAGA
AGATACACCAGACGGAAGAACCGGATCTGCTATCTGCAAGAGATCTTCAGCAACGAGATGGCCAAGG
TGGACGACAGCTTCTTCCACAGACTGGAAGAGTCCTTCCTGGTGGAAGAGGATAAGAAGCACGAGC
GGCACCCCATCTTCGGCAACATCGTGGACGAGGTGGCCTACCACGAGAAGTACCCCACCATCTACCA
CCTGAGAAAGAAACTGGTGGACAGCACCGACAAGGCCGACCTGCGGCTGATCTATCTGGCCCTGGC
CCACATGATCAAGTTCCGGGGCCACTTCCTGATCGAGGGCGACCTGAACCCCGACAACAGCGACGT
GGACAAGCTGTTCATCCAGCTGGTGCAGACCTACAACCAGCTGTTCGAGGAAAACCCCATCAACGCC
AGCGGCGTGGACGCCAAGGCCATCCTGTCTGCCAGACTGAGCAAGAGCAGACGGCTGGAAAATCTG
ATCGCCCAGCTGCCCGGCGAGAAGAAGAATGGCCTGTTCGGAAACCTGATTGCCCTGAGCCTGGGC
CTGACCCCCAACTTCAAGAGCAACTTCGACCTGGCCGAGGATGCCAAACTGCAGCTGAGCAAGGAC
ACCTACGACGACGACCTGGACAACCTGCTGGCCCAGATCGGCGACCAGTACGCCGACCTGTTTCTG
GCCGCCAAGAACCTGTCCGACGCCATCCTGCTGAGCGACATCCTGAGAGTGAACACCGAGATCACC
AAGGCCCCCCTGAGCGCCTCTATGGTGAAGAGATACGACGAGCACCACCAGGACCTGACCCTGCTG
AAAGCTCTCGTGCGGCAGCAGCTGCCTGAGAAGTACAAAGAGATTTTCTTCGACCAGAGCAAGAACG
GCTACGCCGGCTACATTGACGGCGGAGCCAGCCAGGAAGAGTTCTACAAGTTCATCAAGCCCATCCT
GGAAAAGATGGACGGCACCGAGGAACTGCTCGTGAAGCTGAACAGAGAGGACCTGCTGCGGAAGC
AGCGGACCTTCGACAACGGCATTATCCCCCACCAGATCCACCTGGGAGAGCTGCACGCCATTCTGC
GGCGGCAGGGCGATTTTTACCCATTCCTGAAGGACAACCGGGAAAAGATCGAGAAGATCCTGACCTT
CCGCATCCCCTACTACGTGGGCCCTCTGGCCAGGGGAAACAGCAGATTCGCCTGGATGACCAGAAA
GAGCGAGGAAACCATCACCCCCTGGAACTTCGAGGAAGTGGTGGACAAGGGCGCTTCCGCCCAGA
GCTTCATCGAGCGGATGACCAACTTCGATAAGAACCTGCCCAACGAGAAGGTGCTGCCCAAGCACA
GCCTGCTGTACGAGTACTTCACCGTGTATAACGAGCTGACCAAAGTGAAATACGTGACCGAGGGAAT
GAGAAAGCCCGCCTTCCTGAGCGGCGAGCAGAAAAAGGCCATCGTGGACCTGCTGTTCAAGACCAA
CCGGAAAGTGACCGTGAAGCAGCTGAAAGAGGACTACTTCAAGAAAATCGAGTGCTTCGACTCCGTG
GAAATCTCCGGCGTGGAAGATCGGTTCAACGCCTCCCTGGGCACATACCACGATCTGCTGAAAATTAT
CAAGGACAAGGACTTCCTGGACAATGAGGAAAACGAGGACATTCTGGAAGATATCGTGCTGACCCTG
ACACTGTTTGAGGACAGAGAGATGATCGAGGAACGGCTGAAAACCTATGCCCACCTGTTCGACGACA
AAGTGATGAAGCAGCTGAAGCGGCTGAGATACACCGGCTGGGGCAGGCTGAGCCGGAAGCTGATCA
ACGGCATCCGGGACAAGCAGTCCGGCAAGACAATCCTGGATTTCCTGAAGTCCGACGGCTTCGCCA
ACAGAAACTTCATGCAGCTGATCCACGACGACAGCCTGACCTTTAAAGAGGACATCCAGAAAGCCCA

GGTGTCCGGCCAGGGCGATAGCCTGCACGAGCACATTGCCAATCTGGCCGGCAGCCCCGCCATTAA
GAAGGGCATCCTGCAGACAGTGAAGGTGGTGGACGAGCTCGTGAAAGTGATGGGCGGCCACAAGC
CCGAGAACATCGTGATCGAAATGGCCAGAGAGAACCAGACCACCCAGAAGGGACAGAAGAACAGCC
GCGAGAGAATGAAGCGGATCGAAGAGGGCATCAAAGAGCTGGGCAGCCAGATCCTGAAAGAACACC
CCGTGGAAAACACCCAGCTGCAGAACGAGAAGCTGTACCTGTACTACCTGCAGAATGGGCGGGATAT
GTACGTGGACCAGGAACTGGACATCAACCGGCTGTCCGACTACGATGTGGACCATATCGTGCCTCAG
AGCTTTCTGAAGGACGACTCCATCGACAACAAGGTGCTGACCAGAAGCGACAAGAACCGGGGCAAG
AGCGACAACGTGCCCTCCGAAGAGGTCGTGAAGAAGATGAAGAACTACTGGCGGCAGCTGCTGAAC
GCCAAGCTGATTACCCAGAGAAAGTTCGACAATCTGACCAAGGCCGAGAGAGGCGGCCTGAGCGAA
CTGGATAAGGCCGGCTTCATCAAGAGACAGCTGGTGGAAACCCGGCAGATCACAAAGCACGTGGCA
CAGATCCTGGACTCCCGGATGAACACTAAGTACGACGAGAATGACAAGCTGATCCGGGAAGTGAAAG
TGATCACCCTGAAGTCCAAGCTGGTGTCCGATTTCCGGAAGGATTTCCAGTTTTACAAAGTGCGCGA
GATCAACAACTACCACCACGCCCACGACGCCTACCTGAACGCCGTCGTGGGAACCGCCCTGATCAA
AAAGTACCCTAAGCTGGAAAGCGAGTTCGTGTACGGCGACTACAAGGTGTACGACGTGCGGAAGATG
ATCGCCAAGAGCGAGCAGGAAATCGGCAAGGCTACCGCCAAGTACTTCTTCTACAGCAACATCATGA
ACTTTTTCAAGACCGAGATTACCCTGGCCAACGGCGAGATCCGGAAGCGGCCTCTGATCGAGACAAA
CGGCGAAACCGGGGAGATCGTGTGGGATAAGGGCCGGGATTTTGCCACCGTGCGGAAAGTGCTGA
GCATGCCCCAAGTGAATATCGTGAAAAAGACCGAGGTGCAGACAGGCGGCTTCAGCAAAGAGTCTAT
CCTGCCCAAGGGTAACAGCGATAAGCTGATCGCCAGAAAGAAGGACTGGGACCCTAAGAAGTACGG
CGGCTTCAACAGCCCCACCGTGGCCTATTCTGTGCTGGTGGTGGCCAAAGTGGAAAAGGGCAAGTC
CAAGAAACTGAAGAGTGTGAAAGAGCTGCTGGGGATCACCATCATGGAAAGAAGCAGCTTCGAGAA
GAATCCCATCGACTTTCTGGAAGCCAAGGGCTACAAAGAAGTGAAAAAGGACCTGATCATCAAGCTG
CCTAAGTACTCCCTGTTCGAGCTGGAAAACGGCCGGAAGAGAATGCTGGCCTCTGCCGGCGTGCTG
CAGAAGGGAAACGAACTGGCCCTGCCCTCCAAATATGTGAACTTCCTGTACCTGGCCAGCCACTATG
AGAAGCTGAAGGGCTCCCCCGAGGATAATGAGCAGAAACAGCTGTTTGTGGAACAGCACAAGCACTA
CCTGGACGAGATCATCGAGCAGATCAGCGAGTTCTCCAAGAGAGTGATCCTGGCCGACGCTAATCTG
GACAAAGTGCTGTCCGCCTACAACAAGCACCGGGATAAGCCCATCAGAGAGCAGGCCGAGAATATCA
TCCACCTGTTTACCCTGACCAATCTGGGAGCCCCTGCCGCCTTCAAGTACTTTGACACCACCATCAAC
CGGAAGCAATACAACACGACCAAAGAGGTGCTGGACGCCACCCTGATCCGTCAGAGCATCACCGGC
CTGTACGAGACACGGATCGACCTGTCTCAGCTGGGAGGTGACTCTGGCGGCTCAAAAAGAACCGCC
GACGGCAGCGAATTCGAGCCCAAGAAGAAGAGGAAAGTCTAATTAATTAAGCTGCCTTCTGCGGGGC
TTGCCTTCTGGCCATGCCCTTCTTCTCTCCCTTGCACCTGTACCTCTTGGTCTTTGAATAAAGCCTGA
GTAGGAAGCGACTGTGCCTTCTAGTTGCCAGCCATCTGTTGTTTGCCCCTCCCCCGTGCCTTCCTTG
ACCCTGGAAGGTGCCACTCCCACTGTCCTTTCCTAATAAAATGAGAAATTGCATCGCATTGTCTGAG
TAGGTGTCATTCTATTCTGGGGGGTGGGGTGGGGCAGGACAGCAAGGGGGAGGATTGGGAAGACAA
TAGCAGGCATGCTGGGGATGCGGTGGGCTCTATGG

Start	End	Feature Description
1	380	CMV enhancer
381	619	CMV promoter
620	636	T7 promoter with mismatch
637	683	5' UTR
684	740	BP NLS
741	1238	Engineered TadA* variant ABE9 (TadA-8e N108Q L145T)
1239	1334	(SGGS)2 – XTEN – (SGGS)2 linker
1335	5435	Cas9(D10T) Sp-NRCH PAM variant
5436	5501	Linker + SV40 BP NLS

5502	5605	3’ UTR
5606	5830	bGH poly(A) signal

Amino acid sequence

MKRTADGSEFESPKKKRKVSEVEFSHEYWMRHALTLAKRARDEREVPVGAVLVLNNRVIGEGWNRAIGL
HDPTAHAEIMALRQGGLVMQNYRLIDATLYVTFEPCVMCAGAMIHSRIGRVVFGVRQSKRGAAGSLMNVL
NYPGMNHRVEITEGILADECAALTCDFYRMPRQVFNAQKKAQSSINSGGSSGGSSGSETPGTSESATPE
SSGGSSGGSDKKYSIGLTIGTNSVGWAVITDEYKVPSKKFKVLGNTDRHSIKKNLIGALLFDSGETAEATRL
KRTARRRYTRRKNRICYLQEIFSNEMAKVDDSFFHRLEESFLVEEDKKHERHPIFGNIVDEVAYHEKYPTIY
HLRKKLVDSTDKADLRLIYLALAHMIKFRGHFLIEGDLNPDNSDVDKLFIQLVQTYNQLFEENPINASGVDA
KAILSARLSKSRRLENLIAQLPGEKKNGLFGNLIALSLGLTPNFKSNFDLAEDAKLQLSKDTYDDDLDNLLA
QIGDQYADLFLAAKNLSDAILLSDILRVNTEITKAPLSASMVKRYDEHHQDLTLLKALVRQQLPEKYKEIFFD
QSKNGYAGYIDGGASQEEFYKFIKPILEKMDGTEELLVKLNREDLLRKQRTFDNGIIPHQIHLGELHAILRR
QGDFYPFLKDNREKIEKILTFRIPYYVGPLARGNSRFAWMTRKSEETITPWNFEEVVDKGASAQSFIERMT
NFDKNLPNEKVLPKHSLLYEYFTVYNELTKVKYVTEGMRKPAFLSGEQKKAIVDLLFKTNRKVTVKQLKED
YFKKIECFDSVEISGVEDRFNASLGTYHDLLKIIKDKDFLDNEENEDILEDIVLTLTLFEDREMIEERLKTYAH
LFDDKVMKQLKRLRYTGWGRLSRKLINGIRDKQSGKTILDFLKSDGFANRNFMQLIHDDSLTFKEDIQKAQ
VSGQGDSLHEHIANLAGSPAIKKGILQTVKVVDELVKVMGGHKPENIVIEMARENQTTQKGQKNSRERMK
RIEEGIKELGSQILKEHPVENTQLQNEKLYLYYLQNGRDMYVDQELDINRLSDYDVDHIVPQSFLKDDSIDN
KVLTRSDKNRGKSDNVPSEEVVKKMKNYWRQLLNAKLITQRKFDNLTKAERGGLSELDKAGFIKRQLVET
RQITKHVAQILDSRMNTKYDENDKLIREVKVITLKSKLVSDFRKDFQFYKVREINNYHHAHDAYLNAVVGTA
LIKKYPKLESEFVYGDYKVYDVRKMIAKSEQEIGKATAKYFFYSNIMNFFKTEITLANGEIRKRPLIETNGET
GEIVWDKGRDFATVRKVLSMPQVNIVKKTEVQTGGFSKESILPKGNSDKLIARKKDWDPKKYGGFNSPTV
AYSVLVVAKVEKGKSKKLKSVKELLGITIMERSSFEKNPIDFLEAKGYKEVKKDLIIKLPKYSLFELENGRKR
MLASAGVLQKGNELALPSKYVNFLYLASHYEKLKGSPEDNEQKQLFVEQHKHYLDEIIEQISEFSKRVILAD
ANLDKVLSAYNKHRDKPIREQAENIIHLFTLTNLGAPAAFKYFDTTINRKQYNTTKEVLDATLIRQSITGLYET
RIDLSQLGGDSGGSKRTADGSEFEPKKKRKV

Start	End	Feature Description
1	19	BP NLS
20	185	Engineered TadA* variant 8e
186	217	(SGGS)2 – XTEN – (SGGS)2 linker
218	1584	Cas9(D10T) Sp-NRCH PAM variant
1585	1605	Linker + SV40 BP NLS

ABE7.10 – Cas9 (D10T) – NRTH variant
Plasmid ID: pHS0376

DNA sequence

GACATTGATTATTGACTAGTTATTAATAGTAATCAATTACGGGGTCATTAGTTCATAGCCCATATATGGAG
TTCCGCGTTACATAACTTACGGTAAATGGCCCGCCTGGCTGACCGCCCAACGACCCCCGCCCATTGA
CGTCAATAATGACGTATGTTCCCATAGTAACGCCAATAGGGACTTTCCATTGACGTCAATGGGTGGAGT
ATTTACGGTAAACTGCCCACTTGGCAGTACATCAAGTGTATCATATGCCAAGTACGCCCCCTATTGACG
TCAATGACGGTAAATGGCCCGCCTGGCATTATGCCCAGTACATGACCTTATGGGACTTTCCTACTTGG
CAGTACATCTACGTATTAGTCATCGCTATTACCATGGTGATGCGGTTTTGGCAGTACATCAATGGGCGT
GGATAGCGGTTTGACTCACGGGGATTTCCAAGTCTCCACCCCATTGACGTCAATGGGAGTTTGTTTTG
GCACCAAAATCAACGGGACTTTCCAAAATGTCGTAACAACTCCGCCCCATTGACGCAAATGGGCGGT
AGGCGTGTACGGTGGGAGGTCTATATAAGCAGAGCTGGTTTAGTGAACCGTCAGATCTCGAGCTCGG
TACCTAATACGACACACTATAAGGAAATAAGAGAGAAAAGAAGAGTAAGAAGAAATATAAGAGCCACCA
TGAAACGGACAGCCGACGGAAGCGAGTTCGAGTCACCAAAGAAGAAGCGGAAAGTCTCTGAAGTCG
AGTTTAGCCACGAGTATTGGATGAGGCACGCACTGACCCTGGCAAAGCGAGCATGGGATGAAAGAGA
AGTCCCCGTGGGCGCCGTGCTGGTGCACAACAATAGAGTGATCGGAGAGGGATGGAACAGGCCAAT
CGGCCGCCACGACCCTACCGCACACGCAGAGATCATGGCACTGAGGCAGGGAGGCCTGGTCATGC
AGAATTACCGCCTGATCGATGCCACCCTGTATGTGACACTGGAGCCATGCGTGATGTGCGCAGGAGC
AATGATCCACAGCAGGATCGGAAGAGTGGTGTTCGGAGCACGGGACGCCAAGACCGGCGCAGCAG
GCTCCCTGATGGATGTGCTGCACCACCCCGGCATGAACCACCGGGTGGAGATCACAGAGGGAATCC
TGGCAGACGAGTGCGCCGCCCTGCTGAGCGATTTCTTTAGAATGCGGAGACAGGAGATCAAGGCCC
AGAAGAAGGCACAGAGCTCCACCGACTCTGGAGGATCTAGCGGAGGATCCTCTGGAAGCGAGACAC
CAGGCACAAGCGAGTCCGCCACACCAGAGAGCTCCGGCGGCTCCTCCGGAGGATCCTCTGAGGTG
GAGTTTTCCCACGAGTACTGGATGAGACATGCCCTGACCCTGGCCAAGAGGGCACGCGATGAGAGG
GAGGTGCCTGTGGGAGCCGTGCTGGTGCTGAACAATAGAGTGATCGGCGAGGGCTGGAACAGAGC
CATCGGCCTGCACGACCCAACAGCCCATGCCGAAATTATGGCCCTGAGACAGGGCGGCCTGGTCAT
GCAGAACTACAGACTGATTGACGCCACCCTGTACGTGACATTCGAGCCTTGCGTGATGTGCGCCGGC
GCCATGATCCACTCTAGGATCGGCCGCGTGGTGTTTGGCGTGAGGAACGCAAAAACCGGCGCCGCA
GGCTCCCTGATGGACGTGCTGCACTACCCCGGCATGAATCACCGCGTCGAAATTACCGAGGGAATCC
TGGCAGATGAATGTGCCGCCCTGCTGTGCTATTTCTTTCGGATGCCTAGACAGGTGTTCAATGCTCAG
AAGAAGGCCCAGAGCTCCACCGACTCCGGAGGATCTAGCGGAGGCTCCTCTGGCTCTGAGACACCT
GGCACAAGCGAGAGCGCAACACCTGAAAGCAGCGGGGGCAGCAGCGGGGGGTCAGACAAGAAGT
ACAGCATCGGCCTGACCATCGGCACCAACTCTGTGGGCTGGGCCGTGATCACCGACGAGTACAAGG
TGCCCAGCAAGAAATTCAAGGTGCTGGGCAACACCGACCGGCACAGCATCAAGAAGAACCTGATCG
GAGCCCTGCTGTTCGACAGCGGCGAAACAGCCGAGGCCACCCGGCTGAAGAGAACCGCCAGAAGA
AGATACACCAGACGGAAGAACCGGATCTGCTATCTGCAAGAGATCTTCAGCAACGAGATGGCCAAGG
TGGACGACAGCTTCTTCCACAGACTGGAAGAGTCCTTCCTGGTGGAAGAGGATAAGAAGCACGAGC
GGCACCCCATCTTCGGCAACATCGTGGACGAGGTGGCCTACCACGAGAAGTACCCCACCATCTACCA
CCTGAGAAAGAAACTGGTGGACAGCACCGACAAGGCCGACCTGCGGCTGATCTATCTGGCCCTGGC
CCACATGATCAAGTTCCGGGGCCACTTCCTGATCGAGGGCGACCTGAACCCCGACAACAGCGACGT
GGACAAGCTGTTCATCCAGCTGGTGCAGACCTACAACCAGCTGTTCGAGGAAAACCCCATCAACGCC
AGCGGCGTGGACGCCAAGGCCATCCTGTCTGCCAGACTGAGCAAGAGCAGACGGCTGGAAAATCTG
ATCGCCCAGCTGCCCGGCGAGAAGAAGAATGGCCTGTTCGGAAACCTGATTGCCCTGAGCCTGGGC
CTGACCCCCAACTTCAAGAGCAACTTCGACCTGGCCGAGGATGCCAAACTGCAGCTGAGCAAGGAC
ACCTACGACGACGACCTGGACAACCTGCTGGCCCAGATCGGCGACCAGTACGCCGACCTGTTTCTG
GCCGCCAAGAACCTGTCCGACGCCATCCTGCTGAGCGACATCCTGAGAGTGAACACCGAGATCACC
AAGGCCCCCCTGAGCGCCTCTATGGTGAAGAGATACGACGAGCACCACCAGGACCTGACCCTGCTG
AAAGCTCTCGTGCGGCAGCAGCTGCCTGAGAAGTACAAAGAGATTTTCTTCGACCAGAGCAAGAACG
GCTACGCCGGCTACATTGACGGCGGAGCCAGCCAGGAAGAGTTCTACAAGTTCATCAAGCCCATCCT
GGAAAAGATGGACGGCACCGAGGAACTGCTCGTGAAGCTGAACAGAGAGGACCTGCTGCGGAAGC
AGCGGACCTTCGACAACGGCATTATCCCCCACCAGATCCACCTGGGAGAGCTGCACGCCATTCTGC
GGCGGCAGGGCGATTTTTACCCATTCCTGAAGGACAACCGGGAAAAGATCGAGAAGATCCTGACCTT
CCGCATCCCCTACTACGTGGGCCCTCTGGCCAGGGGAAACAGCAGATTCGCCTGGATGACCAGAAA
GAGCGAGGAAACCATCACCCCCTGGAACTTCGAGGAAGTGGTGGACAAGGGCGCTTCCGCCCAGA
GCTTCATCGAGCGGATGACCAACTTCGATAAGAACCTGCCCAACGAGAAGGTGCTGCCCAAGCACA

GCCTGCTGTACGAGTACTTCACCGTGTATAACGAGCTGACCAAAGTGAAATACGTGACCGAGGGAAT
GAGAAAGCCCGCCTTCCTGAGCGGCGAGCAGAAAAAGGCCATCGTGGACCTGCTGTTCAAGACCAA
CCGGAAAGTGACCGTGAAGCAGCTGAAAGAGGACTACTTCAAGAAAATCGAGTGCTTCGACTCCGTG
GAAATCTCCGGCGTGGAAGATCGGTTCAACGCCTCCCTGGGCACATACCACGATCTGCTGAAAATTAT
CAAGGACAAGGACTTCCTGGACAATGAGGAAAACGAGGACATTCTGGAAGATATCGTGCTGACCCTG
ACACTGTTTGAGGACAGAGAGATGATCGAGGAACGGCTGAAAACCTATGCCCACCTGTTCGACGACA
AAGTGATGAAGCAGCTGAAGCGGCTGAGATACACCGGCTGGGGCAGGCTGAGCCGGAAGCTGATCA
ACGGCATCCGGGACAAGCAGTCCGGCAAGACAATCCTGGATTTCCTGAAGTCCGACGGCTTCGCCA
ACAGAAACTTCATGCAGCTGATCCACGACGACAGCCTGACCTTTAAAGAGGACATCCAGAAAGCCCA
GGTGTCCGGCCAGGGCGATAGCCTGCACGAGCACATTGCCAATCTGGCCGGCAGCCCCGCCATTAA
GAAGGGCATCCTGCAGACAGTGAAGGTGGTGGACGAGCTCGTGAAAGTGATGGGCGGCCACAAGC
CCGAGAACATCGTGATCGAAATGGCCAGAGAGAACCAGACCACCCAGAAGGGACAGAAGAACAGCC
GCGAGAGAATGAAGCGGATCGAAGAGGGCATCAAAGAGCTGGGCAGCCAGATCCTGAAAGAACACC
CCGTGGAAAACACCCAGCTGCAGAACGAGAAGCTGTACCTGTACTACCTGCAGAATGGGCGGGATAT
GTACGTGGACCAGGAACTGGACATCAACCGGCTGTCCGACTACGATGTGGACCATATCGTGCCTCAG
AGCTTTCTGAAGGACGACTCCATCGACAACAAGGTGCTGACCAGAAGCGACAAGAACCGGGGCAAG
AGCGACAACGTGCCCTCCGAAGAGGTCGTGAAGAAGATGAAGAACTACTGGCGGCAGCTGCTGAAC
GCCAAGCTGATTACCCAGAGAAAGTTCGACAATCTGACCAAGGCCGAAAGAGGCGGCCTGAGCGAA
CTGGATAAGGCCGGCTTCATCAAGAGACAGCTGGTGGAAACCCGGCAGATCACAAAGCACGTGGCA
CAGATCCTGGACTCCCGGATGAACACTAAGTACGACGAGAATGACAAGCTGATCCGGGAAGTGAAAG
TGATCACCCTGAAGTCCAAGCTGGTGTCCGATTTCCGGAAGGATTTCCAGTTTTACAAAGTGCGCGA
GATCAACAACTACCACCACGCCCACGACGCCTACCTGAACGCCGTCGTGGGAACCGCCCTGATCAA
AAAGTACCCTAAGCTGGAAAGCGAGTTCGTGTACGGCGACTACAAGGTGTACGACGTGCGGAAGATG
ATCGCCAAGAGCGAGCAGGAAATCGGCAAGGCTACCGCCAAGTACTTCTTCTACAGCAACATCATGA
ACTTTTTCAAGACCGAGATTACCCTGGCCAACGGCGAGATCCGGAAGCGGCCTCTGATCGAGACAAA
CGGCGAAACCGGGGAGATCGTGTGGGATAAGGGCCGGGATTTTGCCACCGTGCGGAAAGTGCTGA
GCATGCCCCAAGTGAATATCGTGAAAAAGACCGAGGTGCAGACAGGCGGCTTCAGCAAAGAGTCTAT
CCTGCCCAAGGGCAACAGCGATAAGCTGATCGCCAGAAAGAAGGACTGGGACCCTAAGAAGTACGG
CGGCTTCAACAGCCCCACCGTGGCCTATTCTGTGCTGGTGGTGGCCAAAGTGGAAAAGGGCAAGTC
CAAGAAACTGAAGAGTGTGAAAGAGCTGCTGGGGATCACCATCATGGAAAGAAGCAGCTTCGAGAA
GAATCCCATCGGCTTTCTGGAAGCCAAGGGCTACAAAGAAGTGAAAAAGGACCTGATCATCAAGCTG
CCTAAGTACTCCCTGTTCGAGCTGGAAAACGGCCGGAAGAGAATGCTGGCCTCTGCCAGCGTGCTG
CATAAGGGAAACGAACTGGCCCTGCCCTCCAAATATGTGAACTTCCTGTACCTGGCCAGCCACTATGA
GAAGCTGAAGGGCTCCAGCGAGGATAATAAACAGAAACAGCTGTTTGTGGAACAGCACAAGCACTAC
CTGGACGAGATCATCGAGCAGATCAGCGAGTTCTCCAAGAGAGTGATCCTGGCCGACGCTAATCTGG
ACAAAGTGCTGTCCGCCTACAACAAGCACCGGGATAAGCCCATCAGAGAGCAGGCCGAGAATATCAT
CCACCTGTTTACCCTGACCAATCTGGGAGCCAGCGCCGCCTTCAAGTACTTTGACACCACCATCGGC
CGGAAGCTGTACACCAGCACCAAAGAGGTGCTGGACGCCACCCTGATCCACCAGAGCATCACCGGC
CTGTACGAGACACGGATCGACCTGTCTCAGCTGGGAGGTGACTCTGGCGGCTCAAAAAGAACCGCC
GACGGCAGCGAATTCGAGCCCAAGAAGAAGAGGAAAGTCTAATTAATTAAGCTGCCTTCTGCGGGGC
TTGCCTTCTGGCCATGCCCTTCTTCTCTCCCTTGCACCTGTACCTCTTGGTCTTTGAATAAAGCCTGA
GTAGGAAGCGACTGTGCCTTCTAGTTGCCAGCCATCTGTTGTTTGCCCCTCCCCCGTGCCTTCCTTG
ACCCTGGAAGGTGCCACTCCCACTGTCCTTTCCTAATAAAATGAGAAATTGCATCGCATTGTCTGAG
TAGGTGTCATTCTATTCTGGGGGGTGGGGTGGGGCAGGACAGCAAGGGGGAGGATTGGGAAGACAA
TAGCAGGCATGCTGGGGATGCGGTGGGCTCTATGG

Start	End	Feature Description
1	380	CMV enhancer
381	619	CMV promoter
620	636	T7 promoter with mismatch
637	683	5' UTR

684	740	BP NLS
741	1238	TadA wild type
1239	1334	(SGGS)2 – XTEN – (SGGS)2 linker
1335	1832	Engineered TadA* variant 7.10
1833	1928	(SGGS)2 – XTEN – (SGGS)2 linker
1929	6029	Cas9(D10T) Sp-NRTH PAM variant
6030	6095	Linker + SV40 BP NLS
6096	6196	3' UTR
6197	6424	bGH poly(A) signal

Amino acid sequence

MKRTADGSEFESPKKKRKVSEVEFSHEYWMRHALTLAKRAWDEREVPVGAVLVHNNRVIGEGWNRPIG
RHDPTAHAEIMALRQGGLVMQNYRLIDATLYVTLEPCVMCAGAMIHSRIGRVVFGARDAKTGAAGSLMDV
LHHPGMNHRVEITEGILADECAALLSDFFRMRRQEIKAQKKAQSSTDSGGSSGGSSGSETPGTSESATP
ESSGGSSGGSSEVEFSHEYWMRHALTLAKRARDEREVPVGAVLVLNNRVIGEGWNRAIGLHDPTAHAEI
MALRQGGLVMQNYRLIDATLYVTFEPCVMCAGAMIHSRIGRVVFGVRNAKTGAAGSLMDVLHYPGMNHR
VEITEGILADECAALLCYFFRMPRQVFNAQKKAQSSTDSGGSSGGSSGSETPGTSESATPESSGGSSGG
SDKKYSIGLTIGTNSVGWAVITDEYKVPSKKFKVLGNTDRHSIKKNLIGALLFDSGETAEATRLKRTARRRYT
RRKNRICYLQEIFSNEMAKVDDSFFHRLEESFLVEEDKKHERHPIFGNIVDEVAYHEKYPTIYHLRKKLVDS
TDKADLRLIYLALAHMIKFRGHFLIEGDLNPDNSDVDKLFIQLVQTYNQLFEENPINASGVDAKAILSARLSK
SRRLENLIAQLPGEKKNGLFGNLIALSLGLTPNFKSNFDLAEDAKLQLSKDTYDDDLDNLLAQIGDQYADLF
LAAKNLSDAILLSDILRVNTEITKAPLSASMVKRYDEHHQDLTLLKALVRQQLPEKYKEIFFDQSKNGYAGYI
DGGASQEEFYKFIKPILEKMDGTEELLVKLNREDLLRKQRTFDNGIIPHQIHLGELHAILRRQGDFYPFLKD
NREKIEKILTFRIPYYVGPLARGNSRFAWMTRKSEETITPWNFEEVVDKGASAQSFIERMTNFDKNLPNEK
VLPKHSLLYEYFTVYNELTKVKYVTEGMRKPAFLSGEQKKAIVDLLFKTNRKVTVKQLKEDYFKKIECFDSV
EISGVEDRFNASLGTYHDLLKIIKDKDFLDNEENEDILEDIVLTLTLFEDREMIEERLKTYAHLFDDKVMKQLK
RLRYTGWGRLSRKLINGIRDKQSGKTILDFLKSDGFANRNFMQLIHDDSLTFKEDIQKAQVSGQGDSLHE
HIANLAGSPAIKKGILQTVKVVDELVKVMGGHKPENIVIEMARENQTTQKGQKNSRERMKRIEEGIKELGS
QILKEHPVENTQLQNEKLYLYYLQNGRDMYVDQELDINRLSDYDVDHIVPQSFLKDDSIDNKVLTRSDKNR
GKSDNVPSEEVVKKMKNYWRQLLNAKLITQRKFDNLTKAERGGLSELDKAGFIKRQLVETRQITKHVAQIL
DSRMNTKYDENDKLIREVKVITLKSKLVSDFRKDFQFYKVREINNYHHAHDAYLNAVVGTALIKKYPKLESE
FVYGDYKVYDVRKMIAKSEQEIGKATAKYFFYSNIMNFFKTEITLANGEIRKRPLIETNGETGEIVWDKGRD
FATVRKVLSMPQVNIVKKTEVQTGGFSKESILPKGNSDKLIARKKDWDPKKYGGFNSPTVAYSVLVVAKVE
KGKSKKLKSVKELLGITIMERSSFEKNPIGFLEAKGYKEVKKDLIIKLPKYSLFELENGRKRMLASASVLHKG
NELALPSKYVNFLYLASHYEKLKGSSEDNKQKQLFVEQHKHYLDEIIEQISEFSKRVILADANLDKVLSAYN
KHRDKPIREQAENIIHLFTLTNLGASAAFKYFDTTIGRKLYTSTKEVLDATLIHQSITGLYETRIDLSQLGGDS
GGSKRTADGSEFEPKKKRKV

Start	End	Feature Description
1	19	BP NLS
20	185	TadA wild type
186	217	(SGGS)2 – XTEN – (SGGS)2 linker
218	383	Engineered TadA* variant 7.10

384	415	(SGGS)2 – XTEN – (SGGS)2 linker
416	1782	Cas9(D10T) Sp-NRTH PAM variant
1783	1803	Linker + SV40 BP NLS

ABE8e – Cas9 (D10T) – NRTH variant
Plasmid ID: pHS0370

DNA sequence

GACATTGATTATTGACTAGTTATTAATAGTAATCAATTACGGGGTCATTAGTTCATAGCCCATATATGGAG
TTCCGCGTTACATAACTTACGGTAAATGGCCCGCCTGGCTGACCGCCCAACGACCCCCGCCCATTGA
CGTCAATAATGACGTATGTTCCCATAGTAACGCCAATAGGGACTTTCCATTGACGTCAATGGGTGGAGT
ATTTACGGTAAACTGCCCACTTGGCAGTACATCAAGTGTATCATATGCCAAGTACGCCCCCTATTGACG
TCAATGACGGTAAATGGCCCGCCTGGCATTATGCCCAGTACATGACCTTATGGGACTTTCCTACTTGG
CAGTACATCTACGTATTAGTCATCGCTATTACCATGGTGATGCGGTTTTGGCAGTACATCAATGGGCGT
GGATAGCGGTTTGACTCACGGGGATTTCCAAGTCTCCACCCCATTGACGTCAATGGGAGTTTGTTTTG
GCACCAAAATCAACGGGACTTTCCAAAATGTCGTAACAACTCCGCCCCATTGACGCAAATGGGCGGT
AGGCGTGTACGGTGGGAGGTCTATATAAGCAGAGCTGGTTTAGTGAACCGTCAGATCTCGAGCTCGG
TACCTAATACGACACACTATAAGGAAATAAGAGAGAAAAGAAGAGTAAGAAGAAATATAAGAGCCACCA
TGAAACGGACAGCCGACGGAAGCGAGTTCGAGTCACCAAAGAAGAAGCGGAAAGTCTCTGAGGTG
GAGTTTTCCCACGAGTACTGGATGAGACATGCCCTGACCCTGGCCAAGAGGGCACGGGATGAGAGG
GAGGTGCCTGTGGGAGCCGTGCTGGTGCTGAACAATAGAGTGATCGGCGAGGGCTGGAACAGAGC
CATCGGCCTGCACGACCCAACAGCCCATGCCGAAATTATGGCCCTGAGACAGGGCGGCCTGGTCAT
GCAGAACTACAGACTGATTGACGCCACCCTGTACGTGACATTCGAGCCTTGCGTGATGTGCGCCGGC
GCCATGATCCACTCTAGGATCGGCCGCGTGGTGTTTGGCGTGAGGAACTCAAAAAGAGGCGCCGCA
GGCTCCCTGATGAACGTGCTGAACTACCCCGGCATGAATCACCGCGTCGAAATTACCGAGGGAATCC
TGGCAGATGAATGTGCCGCCCTGCTGTGCGATTTCTATCGGATGCCTAGACAGGTGTTCAATGCTCAG
AAGAAGGCCCAGAGCTCCATCAACTCCGGAGGATCTAGCGGAGGCTCCTCTGGCTCTGAGACACCT
GGCACAAGCGAGAGCGCAACACCTGAAAGCAGCGGGGGCAGCAGCGGGGGGTCAGACAAGAAGT
ACAGCATCGGCCTGACCATCGGCACCAACTCTGTGGGCTGGGCCGTGATCACCGACGAGTACAAGG
TGCCCAGCAAGAAATTCAAGGTGCTGGGCAACACCGACCGGCACAGCATCAAGAAGAACCTGATCG
GAGCCCTGCTGTTCGACAGCGGCGAAACAGCCGAGGCCACCCGGCTGAAGAGAACCGCCAGAAGA
AGATACACCAGACGGAAGAACCGGATCTGCTATCTGCAAGAGATCTTCAGCAACGAGATGGCCAAGG
TGGACGACAGCTTCTTCCACAGACTGGAAGAGTCCTTCCTGGTGGAAGAGGATAAGAAGCACGAGC
GGCACCCCATCTTCGGCAACATCGTGGACGAGGTGGCCTACCACGAGAAGTACCCCACCATCTACCA
CCTGAGAAAGAAACTGGTGGACAGCACCGACAAGGCCGACCTGCGGCTGATCTATCTGGCCCTGGC
CCACATGATCAAGTTCCGGGGCCACTTCCTGATCGAGGGCGACCTGAACCCCGACAACAGCGACGT
GGACAAGCTGTTCATCCAGCTGGTGCAGACCTACAACCAGCTGTTCGAGGAAAACCCCATCAACGCC
AGCGGCGTGGACGCCAAGGCCATCCTGTCTGCCAGACTGAGCAAGAGCAGACGGCTGGAAAATCTG
ATCGCCCAGCTGCCCGGCGAGAAGAAGAATGGCCTGTTCGGAAACCTGATTGCCCTGAGCCTGGGC
CTGACCCCCAACTTCAAGAGCAACTTCGACCTGGCCGAGGATGCCAAACTGCAGCTGAGCAAGGAC
ACCTACGACGACGACCTGGACAACCTGCTGGCCCAGATCGGCGACCAGTACGCCGACCTGTTTCTG
GCCGCCAAGAACCTGTCCGACGCCATCCTGCTGAGCGACATCCTGAGAGTGAACACCGAGATCACC
AAGGCCCCCCTGAGCGCCTCTATGGTGAAGAGATACGACGAGCACCACCAGGACCTGACCCTGCTG
AAAGCTCTCGTGCGGCAGCAGCTGCCTGAGAAGTACAAAGAGATTTTCTTCGACCAGAGCAAGAACG
GCTACGCCGGCTACATTGACGGCGGAGCCAGCCAGGAAGAGTTCTACAAGTTCATCAAGCCCATCCT
GGAAAAGATGGACGGCACCGAGGAACTGCTCGTGAAGCTGAACAGAGAGGACCTGCTGCGGAAGC
AGCGGACCTTCGACAACGGCATTATCCCCCACCAGATCCACCTGGGAGAGCTGCACGCCATTCTGC
GGCGGCAGGGCGATTTTTACCCATTCCTGAAGGACAACCGGGAAAAGATCGAGAAGATCCTGACCTT
CCGCATCCCCTACTACGTGGGCCCTCTGGCCAGGGGAAACAGCAGATTCGCCTGGATGACCAGAAA
GAGCGAGGAAACCATCACCCCCTGGAACTTCGAGGAAGTGGTGGACAAGGGCGCTTCCGCCCAGA
GCTTCATCGAGCGGATGACCAACTTCGATAAGAACCTGCCCAACGAGAAGGTGCTGCCCAAGCACA
GCCTGCTGTACGAGTACTTCACCGTGTATAACGAGCTGACCAAAGTGAAATACGTGACCGAGGGAAT
GAGAAAGCCCGCCTTCCTGAGCGGCGAGCAGAAAAAGGCCATCGTGGACCTGCTGTTCAAGACCAA
CCGGAAAGTGACCGTGAAGCAGCTGAAAGAGGACTACTTCAAGAAAATCGAGTGCTTCGACTCCGTG
GAAATCTCCGGCGTGGAAGATCGGTTCAACGCCTCCCTGGGCACATACCACGATCTGCTGAAAATTAT
CAAGGACAAGGACTTCCTGGACAATGAGGAAAACGAGGACATTCTGGAAGATATCGTGCTGACCCTG
ACACTGTTTGAGGACAGAGAGATGATCGAGGAACGGCTGAAAACCTATGCCCACCTGTTCGACGACA
AAGTGATGAAGCAGCTGAAGCGGCTGAGATACACCGGCTGGGGCAGGCTGAGCCGGAAGCTGATCA
ACGGCATCCGGGACAAGCAGTCCGGCAAGACAATCCTGGATTTCCTGAAGTCCGACGGCTTCGCCA
ACAGAAACTTCATGCAGCTGATCCACGACGACAGCCTGACCTTTAAAGAGGACATCCAGAAAGCCCA

GGTGTCCGGCCAGGGCGATAGCCTGCACGAGCACATTGCCAATCTGGCCGGCAGCCCCGCCATTAA
GAAGGGCATCCTGCAGACAGTGAAGGTGGTGGACGAGCTCGTGAAAGTGATGGGCGGCCACAAGC
CCGAGAACATCGTGATCGAAATGGCCAGAGAGAACCAGACCACCCAGAAGGGACAGAAGAACAGCC
GCGAGAGAATGAAGCGGATCGAAGAGGGCATCAAAGAGCTGGGCAGCCAGATCCTGAAAGAACACC
CCGTGGAAAACACCCAGCTGCAGAACGAGAAGCTGTACCTGTACTACCTGCAGAATGGGCGGGATAT
GTACGTGGACCAGGAACTGGACATCAACCGGCTGTCCGACTACGATGTGGACCATATCGTGCCTCAG
AGCTTTCTGAAGGACGACTCCATCGACAACAAGGTGCTGACCAGAAGCGACAAGAACCGGGGCAAG
AGCGACAACGTGCCCTCCGAAGAGGTCGTGAAGAAGATGAAGAACTACTGGCGGCAGCTGCTGAAC
GCCAAGCTGATTACCCAGAGAAAGTTCGACAATCTGACCAAGGCCGAAAGAGGCGGCCTGAGCGAA
CTGGATAAGGCCGGCTTCATCAAGAGACAGCTGGTGGAAACCCGGCAGATCACAAAGCACGTGGCA
CAGATCCTGGACTCCCGGATGAACACTAAGTACGACGAGAATGACAAGCTGATCCGGGAAGTGAAAG
TGATCACCCTGAAGTCCAAGCTGGTGTCCGATTTCCGGAAGGATTTCCAGTTTTACAAAGTGCGCGA
GATCAACAACTACCACCACGCCCACGACGCCTACCTGAACGCCGTCGTGGGAACCGCCCTGATCAA
AAAGTACCCTAAGCTGGAAAGCGAGTTCGTGTACGGCGACTACAAGGTGTACGACGTGCGGAAGATG
ATCGCCAAGAGCGAGCAGGAAATCGGCAAGGCTACCGCCAAGTACTTCTTCTACAGCAACATCATGA
ACTTTTTCAAGACCGAGATTACCCTGGCCAACGGCGAGATCCGGAAGCGGCCTCTGATCGAGACAAA
CGGCGAAACCGGGGAGATCGTGTGGGATAAGGGCCGGGATTTTGCCACCGTGCGGAAAGTGCTGA
GCATGCCCCAAGTGAATATCGTGAAAAAGACCGAGGTGCAGACAGGCGGCTTCAGCAAAGAGTCTAT
CCTGCCCAAGGGCAACAGCGATAAGCTGATCGCCAGAAAGAAGGACTGGGACCCTAAGAAGTACGG
CGGCTTCAACAGCCCCACCGTGGCCTATTCTGTGCTGGTGGTGGCCAAAGTGGAAAAGGGCAAGTC
CAAGAAACTGAAGAGTGTGAAAGAGCTGCTGGGGATCACCATCATGGAAAGAAGCAGCTTCGAGAA
GAATCCCATCGGCTTTCTGGAAGCCAAGGGCTACAAAGAAGTGAAAAAGGACCTGATCATCAAGCTG
CCTAAGTACTCCCTGTTCGAGCTGGAAAACGGCCGGAAGAGAATGCTGGCCTCTGCCAGCGTGCTG
CATAAGGGAAACGAACTGGCCCTGCCCTCCAAATATGTGAACTTCCTGTACCTGGCCAGCCACTATGA
GAAGCTGAAGGGCTCCAGCGAGGATAATAAACAGAAACAGCTGTTTGTGGAACAGCACAAGCACTAC
CTGGACGAGATCATCGAGCAGATCAGCGAGTTCTCCAAGAGAGTGATCCTGGCCGACGCTAATCTGG
ACAAAGTGCTGTCCGCCTACAACAAGCACCGGGATAAGCCCATCAGAGAGCAGGCCGAGAATATCAT
CCACCTGTTTACCCTGACCAATCTGGGAGCCAGCGCCGCCTTCAAGTACTTTGACACCACCATCGGC
CGGAAGCTGTACACCAGCACCAAAGAGGTGCTGGACGCCACCCTGATCCACCAGAGCATCACCGGC
CTGTACGAGACACGGATCGACCTGTCTCAGCTGGGAGGTGACTCTGGCGGCTCAAAAAGAACCGCC
GACGGCAGCGAATTCGAGCCCAAGAAGAAGAGGAAAGTCTAATTAATTAAGCTGCCTTCTGCGGGGC
TTGCCTTCTGGCCATGCCCTTCTTCTCTCCCTTGCACCTGTACCTCTTGGTCTTTGAATAAAGCCTGA
GTAGGAAGCGACTGTGCCTTCTAGTTGCCAGCCATCTGTTGTTTGCCCCTCCCCCGTGCCTTCCTTG
ACCCTGGAAGGTGCCACTCCCACTGTCCTTTCCTAATAAAATGAGAAAATTGCATCGCATTGTCTGAG
TAGGTGTCATTCTATTCTGGGGGGTGGGGTGGGGCAGGACAGCAAGGGGGAGGATTGGGAAGACAA
TAGCAGGCATGCTGGGGATGCGGTGGGCTCTATGG

Start	End	Feature Description
1	380	CMV enhancer
381	619	CMV promoter
620	636	T7 promoter with mismatch
637	683	5' UTR
684	740	BP NLS
741	1238	Engineered TadA* variant 8e
1239	1334	(SGGS)2 – XTEN – (SGGS)2 linker
1335	5435	Cas9(D10T) Sp-NRTH PAM variant
5436	5501	Linker + SV40 BP NLS

5502	5605	3’ UTR
5606	5830	bGH poly(A) signal

Amino acid sequence

MKRTADGSEFESPKKKRKVSEVEFSHEYWMRHALTLAKRARDEREVPVGAVLVLNNRVIGEGWNRAIGL
HDPTAHAEIMALRQGGLVMQNYRLIDATLYVTFEPCVMCAGAMIHSRIGRVVFGVRNSKRGAAGSLMNVL
NYPGMNHRVEITEGILADECAALLCDFYRMPRQVFNAQKKAQSSINSGGSSGGSSGSETPGTSESATPE
SSGGSSGGSDKKYSIGLTIGTNSVGWAVITDEYKVPSKKFKVLGNTDRHSIKKNLIGALLFDSGETAEATRL
KRTARRRYTRRKNRICYLQEIFSNEMAKVDDSFFHRLEESFLVEEDKKHERHPIFGNIVDEVAYHEKYPTIY
HLRKKLVDSTDKADLRLIYLALAHMIKFRGHFLIEGDLNPDNSDVDKLFIQLVQTYNQLFEENPINASGVDA
KAILSARLSKSRRLENLIAQLPGEKKNGLFGNLIALSLGLTPNFKSNFDLAEDAKLQLSKDTYDDDLDNLLA
QIGDQYADLFLAAKNLSDAILLSDILRVNTEITKAPLSASMVKRYDEHHQDLTLLKALVRQQLPEKYKEIFFD
QSKNGYAGYIDGGASQEEFYKFIKPILEKMDGTEELLVKLNREDLLRKQRTFDNGIIPHQIHLGELHAILRR
QGDFYPFLKDNREKIEKILTFRIPYYVGPLARGNSRFAWMTRKSEETITPWNFEEVVDKGASAQSFIERMT
NFDKNLPNEKVLPKHSLLYEYFTVYNELTKVKYVTEGMRKPAFLSGEQKKAIVDLLFKTNRKVTVKQLKED
YFKKIECFDSVEISGVEDRFNASLGTYHDLLKIIKDKDFLDNEENEDILEDIVLTLTLFEDREMIEERLKTYAH
LFDDKVMKQLKRLRYTGWGRLSRKLINGIRDKQSGKTILDFLKSDGFANRNFMQLIHDDSLTFKEDIQKAQ
VSGQGDSLHEHIANLAGSPAIKKGILQTVKVVDELVKVMGGHKPENIVIEMARENQTTQKGQKNSRERMK
RIEEGIKELGSQILKEHPVENTQLQNEKLYLYYLQNGRDMYVDQELDINRLSDYDVDHIVPQSFLKDDSIDN
KVLTRSDKNRGKSDNVPSEEVVKKMKNYWRQLLNAKLITQRKFDNLTKAERGGLSELDKAGFIKRQLVET
RQITKHVAQILDSRMNTKYDENDKLIREVKVITLKSKLVSDFRKDFQFYKVREINNYHHAHDAYLNAVVGTA
LIKKYPKLESEFVYGDYKVYDVRKMIAKSEQEIGKATAKYFFYSNIMNFFKTEITLANGEIRKRPLIETNGET
GEIVWDKGRDFATVRKVLSMPQVNIVKKTEVQTGGFSKESILPKGNSDKLIARKKDWDPKKYGGFNSPTV
AYSVLVVAKVEKGKSKKLKSVKELLGITIMERSSFEKNPIGFLEAKGYKEVKKDLIIKLPKYSLFELENGRKR
MLASASVLHKGNELALPSKYVNFLYLASHYEKLKGSSEDNKQKQLFVEQHKHYLDEIIEQISEFSKRVILAD
ANLDKVLSAYNKHRDKPIREQAENIIHLFTLTNLGASAAFKYFDTTIGRKLYTSTKEVLDATLIHQSITGLYET
RIDLSQLGGDSGGSKRTADGSEFEPKKKRKV

Start	End	Feature Description
1	19	BP NLS
20	185	Engineered TadA* variant 8e
186	217	(SGGS)2 – XTEN – (SGGS)2 linker
218	1584	Cas9(D10T) Sp-NRTH PAM variant
1585	1605	Linker + SV40 BP NLS

ABE8e(V106W) – Cas9 (D10T) – NRTH variant
Plasmid ID: pHS0371

DNA sequence
GACATTGATTATTGACTAGTTATTAATAGTAATCAATTACGGGGTCATTAGTTCATAGCCCATATATGGAG
TTCCGCGTTACATAACTTACGGTAAATGGCCCGCCTGGCTGACCGCCCAACGACCCCCGCCCATTGA
CGTCAATAATGACGTATGTTCCCATAGTAACGCCAATAGGGACTTTCCATTGACGTCAATGGGTGGAGT
ATTTACGGTAAACTGCCCACTTGGCAGTACATCAAGTGTATCATATGCCAAGTACGCCCCCTATTGACG
TCAATGACGGTAAATGGCCCGCCTGGCATTATGCCCAGTACATGACCTTATGGGACTTTCCTACTTGG
CAGTACATCTACGTATTAGTCATCGCTATTACCATGGTGATGCGGTTTTGGCAGTACATCAATGGGCGT
GGATAGCGGTTTGACTCACGGGGATTTCCAAGTCTCCACCCCATTGACGTCAATGGGAGTTTGTTTTG
GCACCAAAATCAACGGGACTTTCCAAAATGTCGTAACAACTCCGCCCCATTGACGCAAATGGGCGGT
AGGCGTGTACGGTGGGAGGTCTATATAAGCAGAGCTGGTTTAGTGAACCGTCAGATCTCGAGCTCGG
TACCTAATACGACACACTATAAGGAAATAAGAGAGAAAAGAAGAGTAAGAAGAAATATAAGAGCCACCA
TGAAACGGACAGCCGACGGAAGCGAGTTCGAGTCACCAAAGAAGAAGCGGAAAGTCTCTGAGGTG
GAGTTTTCCCACGAGTACTGGATGAGACATGCCCTGACCCTGGCCAAGAGGGCACGGGATGAGAGG
GAGGTGCCTGTGGGAGCCGTGCTGGTGCTGAACAATAGAGTGATCGGCGAGGGCTGGAACAGAGC
CATCGGCCTGCACGACCCAACAGCCCATGCCGAAATTATGGCCCTGAGACAGGGCGGCCTGGTCAT
GCAGAACTACAGACTGATTGACGCCACCCTGTACGTGACATTCGAGCCTTGCGTGATGTGCGCCGGC
GCCATGATCCACTCTAGGATCGGCCGCGTGGTGTTTGGATGGAGAAATTCTAAAAGAGGCGCCGCAG
GCTCCCTGATGAACGTGCTGAACTACCCCGGCATGAATCACCGCGTCGAAATTACCGAGGGAATCCT
GGCAGATGAATGTGCCGCCCTGCTGTGCGATTTCTATCGGATGCCTAGACAGGTGTTCAATGCTCAG
AAGAAGGCCCAGAGCTCCATCAACTCCGGAGGATCTAGCGGAGGCTCCTCTGGCTCTGAGACACCT
GGCACAAGCGAGAGCGCAACACCTGAAAGCAGCGGGGGCAGCAGCGGGGGGTCAGACAAGAAGT
ACAGCATCGGCCTGACCATCGGCACCAACTCTGTGGGCTGGGCCGTGATCACCGACGAGTACAAGG
TGCCCAGCAAGAAATTCAAGGTGCTGGGCAACACCGACCGGCACAGCATCAAGAAGAACCTGATCG
GAGCCCTGCTGTTCGACAGCGGCGAAACAGCCGAGGCCACCCGGCTGAAGAGAACCGCCAGAAGA
AGATACACCAGACGGAAGAACCGGATCTGCTATCTGCAAGAGATCTTCAGCAACGAGATGGCCAAGG
TGGACGACAGCTTCTTCCACAGACTGGAAGAGTCCTTCCTGGTGGAAGAGGATAAGAAGCACGAGC
GGCACCCCATCTTCGGCAACATCGTGGACGAGGTGGCCTACCACGAGAAGTACCCCACCATCTACCA
CCTGAGAAAGAAACTGGTGGACAGCACCGACAAGGCCGACCTGCGGCTGATCTATCTGGCCCTGGC
CCACATGATCAAGTTCCGGGGCCACTTCCTGATCGAGGGCGACCTGAACCCCGACAACAGCGACGT
GGACAAGCTGTTCATCCAGCTGGTGCAGACCTACAACCAGCTGTTCGAGGAAAACCCCATCAACGCC
AGCGGCGTGGACGCCAAGGCCATCCTGTCTGCCAGACTGAGCAAGAGCAGACGGCTGGAAAATCTG
ATCGCCCAGCTGCCCGGCGAGAAGAAGAATGGCCTGTTCGGAAACCTGATTGCCCTGAGCCTGGGC
CTGACCCCCAACTTCAAGAGCAACTTCGACCTGGCCGAGGATGCCAAACTGCAGCTGAGCAAGGAC
ACCTACGACGACGACCTGGACAACCTGCTGGCCCAGATCGGCGACCAGTACGCCGACCTGTTTCTG
GCCGCCAAGAACCTGTCCGACGCCATCCTGCTGAGCGACATCCTGAGAGTGAACACCGAGATCACC
AAGGCCCCCCTGAGCGCCTCTATGGTGAAGAGATACGACGAGCACCACCAGGACCTGACCCTGCTG
AAAGCTCTCGTGCGGCAGCAGCTGCCTGAGAAGTACAAAGAGATTTTCTTCGACCAGAGCAAGAACG
GCTACGCCGGCTACATTGACGGCGGAGCCAGCCAGGAAGAGTTCTACAAGTTCATCAAGCCCATCCT
GGAAAAGATGGACGGCACCGAGGAACTGCTCGTGAAGCTGAACAGAGAGGACCTGCTGCGGAAGC
AGCGGACCTTCGACAACGGCATTATCCCCCACCAGATCCACCTGGGAGAGCTGCACGCCATTCTGC
GGCGGCAGGGCGATTTTTACCCATTCCTGAAGGACAACCGGGAAAAGATCGAGAAGATCCTGACCTT
CCGCATCCCCTACTACGTGGGCCCTCTGGCCAGGGGAAACAGCAGATTCGCCTGGATGACCAGAAA
GAGCGAGGAAACCATCACCCCCTGGAACTTCGAGGAAGTGGTGGACAAGGGCGCTTCCGCCCAGA
GCTTCATCGAGCGGATGACCAACTTCGATAAGAACCTGCCCAACGAGAAGGTGCTGCCCAAGCACA
GCCTGCTGTACGAGTACTTCACCGTGTATAACGAGCTGACCAAAGTGAAATACGTGACCGAGGGAAT
GAGAAAGCCCGCCTTCCTGAGCGGCGAGCAGAAAAAGGCCATCGTGGACCTGCTGTTCAAGACCAA
CCGGAAAGTGACCGTGAAGCAGCTGAAAGAGGACTACTTCAAGAAAATCGAGTGCTTCGACTCCGTG
GAAATCTCCGGCGTGGAAGATCGGTTCAACGCCTCCCTGGGCACATACCACGATCTGCTGAAAATTAT
CAAGGACAAGGACTTCCTGGACAATGAGGAAAACGAGGACATTCTGGAAGATATCGTGCTGACCCTG
ACACTGTTTGAGGACAGAGAGATGATCGAGGAACGGCTGAAAACCTATGCCCACCTGTTCGACGACA
AAGTGATGAAGCAGCTGAAGCGGCTGAGATACACCGGCTGGGGCAGGCTGAGCCGGAAGCTGATCA
ACGGCATCCGGGACAAGCAGTCCGGCAAGACAATCCTGGATTTCCTGAAGTCCGACGGCTTCGCCA
ACAGAAACTTCATGCAGCTGATCCACGACGACAGCCTGACCTTTAAAGAGGACATCCAGAAAGCCCA

GGTGTCCGGCCAGGGCGATAGCCTGCACGAGCACATTGCCAATCTGGCCGGCAGCCCCGCCATTAA
GAAGGGCATCCTGCAGACAGTGAAGGTGGTGGACGAGCTCGTGAAAGTGATGGGCGGCCACAAGC
CCGAGAACATCGTGATCGAAATGGCCAGAGAGAACCAGACCACCCAGAAGGGACAGAAGAACAGCC
GCGAGAGAATGAAGCGGATCGAAGAGGGCATCAAAGAGCTGGGCAGCCAGATCCTGAAAGAACACC
CCGTGGAAAACACCCAGCTGCAGAACGAGAAGCTGTACCTGTACTACCTGCAGAATGGGCGGGATAT
GTACGTGGACCAGGAACTGGACATCAACCGGCTGTCCGACTACGATGTGGACCATATCGTGCCTCAG
AGCTTTCTGAAGGACGACTCCATCGACAACAAGGTGCTGACCAGAAGCGACAAGAACCGGGGCAAG
AGCGACAACGTGCCCTCCGAAGAGGTCGTGAAGAAGATGAAGAACTACTGGCGGCAGCTGCTGAAC
GCCAAGCTGATTACCCAGAGAAAGTTCGACAATCTGACCAAGGCCGAAAGAGGCGGCCTGAGCGAA
CTGGATAAGGCCGGCTTCATCAAGAGACAGCTGGTGGAAACCCGGCAGATCACAAAGCACGTGGCA
CAGATCCTGGACTCCCGGATGAACACTAAGTACGACGAGAATGACAAGCTGATCCGGGAAGTGAAAG
TGATCACCCTGAAGTCCAAGCTGGTGTCCGATTTCCGGAAGGATTTCCAGTTTTACAAAGTGCGCGA
GATCAACAACTACCACCACGCCCACGACGCCTACCTGAACGCCGTCGTGGGAACCGCCCTGATCAA
AAAGTACCCTAAGCTGGAAAGCGAGTTCGTGTACGGCGACTACAAGGTGTACGACGTGCGGAAGATG
ATCGCCAAGAGCGAGCAGGAAATCGGCAAGGCTACCGCCAAGTACTTCTTCTACAGCAACATCATGA
ACTTTTTCAAGACCGAGATTACCCTGGCCAACGGCGAGATCCGGAAGCGGCCTCTGATCGAGACAAA
CGGCGAAACCGGGGAGATCGTGTGGGATAAGGGCCGGGATTTTGCCACCGTGCGGAAAGTGCTGA
GCATGCCCCAAGTGAATATCGTGAAAAAGACCGAGGTGCAGACAGGCGGCTTCAGCAAAGAGTCTAT
CCTGCCCAAGGGCAACAGCGATAAGCTGATCGCCAGAAAGAAGGACTGGGACCCTAAGAAGTACGG
CGGCTTCAACAGCCCCACCGTGGCCTATTCTGTGCTGGTGGTGGCCAAAGTGGAAAAGGGCAAGTC
CAAGAAACTGAAGAGTGTGAAAGAGCTGCTGGGGATCACCATCATGGAAAGAAGCAGCTTCGAGAA
GAATCCCATCGGCTTTCTGGAAGCCAAGGGCTACAAAGAAGTGAAAAAGGACCTGATCATCAAGCTG
CCTAAGTACTCCCTGTTCGAGCTGGAAAACGGCCGGAAGAGAATGCTGGCCTCTGCCAGCGTGCTG
CATAAGGGAAACGAACTGGCCCTGCCCTCCAAATATGTGAACTTCCTGTACCTGGCCAGCCACTATGA
GAAGCTGAAGGGCTCCAGCGAGGATAATAAACAGAAACAGCTGTTTGTGGAACAGCACAAGCACTAC
CTGGACGAGATCATCGAGCAGATCAGCGAGTTCTCCAAGAGAGTGATCCTGGCCGACGCTAATCTGG
ACAAAGTGCTGTCCGCCTACAACAAGCACCGGGATAAGCCCATCAGAGAGCAGGCCGAGAATATCAT
CCACCTGTTTACCCTGACCAATCTGGGAGCCAGCGCCGCCTTCAAGTACTTTGACACCACCATCGGC
CGGAAGCTGTACACCAGCACCAAAGAGGTGCTGGACGCCACCCTGATCCACCAGAGCATCACCGGC
CTGTACGAGACACGGATCGACCTGTCTCAGCTGGGAGGTGACTCTGGCGGCTCAAAAAGAACCGCC
GACGGCAGCGAATTCGAGCCCAAGAAGAAGAGGAAAGTCTAATTAATTAAGCTGCCTTCTGCGGGGC
TTGCCTTCTGGCCATGCCCTTCTTCTCTCCCTTGCACCTGTACCTCTTGGTCTTTGAATAAAGCCTGA
GTAGGAAGCGACTGTGCCTTCTAGTTGCCAGCCATCTGTTGTTTGCCCCTCCCCCGTGCCTTCCTTG
ACCCTGGAAGGTGCCACTCCCACTGTCCTTTCCTAATAAAATGAGAAATTGCATCGCATTGTCTGAG
TAGGTGTCATTCTATTCTGGGGGGTGGGGTGGGGCAGGACAGCAAGGGGGAGGATTGGGAAGACAA
TAGCAGGCATGCTGGGGATGCGGTGGGCTCTATGG

Start	End	Feature Description
1	380	CMV enhancer
381	619	CMV promoter
620	636	T7 promoter with mismatch
637	683	5' UTR
684	740	BP NLS
741	1238	Engineered TadA* variant 8e(V106W)
1239	1334	(SGGS)2 – XTEN – (SGGS)2 linker
1335	5435	Cas9(D10T) Sp-NRTH PAM variant
5436	5501	Linker + SV40 BP NLS

5502	5605	3' UTR
5606	5830	bGH poly(A) signal

Amino acid sequence
MKRTADGSEFESPKKKRKVSEVEFSHEYWMRHALTLAKRARDEREVPVGAVLVLNNRVIGEGWNRAIGL
HDPTAHAEIMALRQGGLVMQNYRLIDATLYVTFEPCVMCAGAMIHSRIGRVVFGWRNSKRGAAGSLMNV
LNYPGMNHRVEITEGILADECAALLCDFYRMPRQVFNAQKKAQSSINSGGSSGGSSGSETPGTSESATP
ESSGGSSGGSDKKYSIGLTIGTNSVGWAVITDEYKVPSKKFKVLGNTDRHSIKKNLIGALLFDSGETAEATR
LKRTARRRYTRRKNRICYLQEIFSNEMAKVDDSFFHRLEESFLVEEDKKHERHPIFGNIVDEVAYHEKYPTI
YHLRKKLVDSTDKADLRLIYLALAHMIKFRGHFLIEGDLNPDNSDVDKLFIQLVQTYNQLFEENPINASGVD
AKAILSARLSKSRRLENLIAQLPGEKKNGLFGNLIALSLGLTPNFKSNFDLAEDAKLQLSKDTYDDDLDNLL
AQIGDQYADLFLAAKNLSDAILLSDILRVNTEITKAPLSASMVKRYDEHHQDLTLLKALVRQQLPEKYKEIFF
DQSKNGYAGYIDGGASQEEFYKFIKPILEKMDGTEELLVKLNREDLLRKQRTFDNGIIPHQIHLGELHAILRR
QGDFYPFLKDNREKIEKILTFRIPYYVGPLARGNSRFAWMTRKSEETITPWNFEEVVDKGASAQSFIERMT
NFDKNLPNEKVLPKHSLLYEYFTVYNELTKVKYVTEGMRKPAFLSGEQKKAIVDLLFKTNRKVTVKQLKED
YFKKIECFDSVEISGVEDRFNASLGTYHDLLKIIKDKDFLDNEENEDILEDIVLTLTLFEDREMIEERLKTYAH
LFDDKVMKQLKRLRYTGWGRLSRKLINGIRDKQSGKTILDFLKSDGFANRNFMQLIHDDSLTFKEDIQKAQ
VSGQGDSLHEHIANLAGSPAIKKGILQTVKVVDELVKVMGGHKPENIVIEMARENQTTQKGQKNSRERMK
RIEEGIKELGSQILKEHPVENTQLQNEKLYLYYLQNGRDMYVDQELDINRLSDYDVDHIVPQSFLKDDSIDN
KVLTRSDKNRGKSDNVPSEEVVKKMKNYWRQLLNAKLITQRKFDNLTKAERGGLSELDKAGFIKRQLVET
RQITKHVAQILDSRMNTKYDENDKLIREVKVITLKSKLVSDFRKDFQFYKVREINNYHHAHDAYLNAVVGTA
LIKKYPKLESEFVYGDYKVYDVRKMIAKSEQEIGKATAKYFFYSNIMNFFKTEITLANGEIRKRPLIETNGET
GEIVWDKGRDFATVRKVLSMPQVNIVKKTEVQTGGFSKESILPKGNSDKLIARKKDWDPKKYGGFNSPTV
AYSVLVVAKVEKGKSKKLKSVKELLGITIMERSSFEKNPIGFLEAKGYKEVKKDLIIKLPKYSLFELENGRKR
MLASASVLHKGNELALPSKYVNFLYLASHYEKLKGSSEDNKQKQLFVEQHKHYLDEIIEQISEFSKRVILAD
ANLDKVLSAYNKHRDKPIREQAENIIHLFTLTNLGASAAFKYFDTTIGRKLYTSTKEVLDATLIHQSITGLYET
RIDLSQLGGDSGGSKRTADGSEFEPKKKRKV

Start	End	Feature Description
1	19	BP NLS
20	185	Engineered TadA* variant 8e
186	217	(SGGS)2 – XTEN – (SGGS)2 linker
218	1584	Cas9(D10T) Sp-NRTH PAM variant
1585	1605	Linker + SV40 BP NLS

ABE9 – Cas9 (D10T) – NRTH variant
Plasmid ID: pHS0412

DNA sequence

GACATTGATTATTGACTAGTTATTAATAGTAATCAATTACGGGGTCATTAGTTCATAGCCCATATATGGAG
TTCCGCGTTACATAACTTACGGTAAATGGCCCGCCTGGCTGACCGCCCAACGACCCCCGCCCATTGA
CGTCAATAATGACGTATGTTCCCATAGTAACGCCAATAGGGACTTTCCATTGACGTCAATGGGTGGAGT
ATTTACGGTAAACTGCCCACTTGGCAGTACATCAAGTGTATCATATGCCAAGTACGCCCCCTATTGACG
TCAATGACGGTAAATGGCCCGCCTGGCATTATGCCCAGTACATGACCTTATGGGACTTTCCTACTTGG
CAGTACATCTACGTATTAGTCATCGCTATTACCATGGTGATGCGGTTTTGGCAGTACATCAATGGGCGT
GGATAGCGGTTTGACTCACGGGGATTTCCAAGTCTCCACCCCATTGACGTCAATGGGAGTTTGTTTTG
GCACCAAAATCAACGGGACTTTCCAAAATGTCGTAACAACTCCGCCCCATTGACGCAAATGGGCGGT
AGGCGTGTACGGTGGGAGGTCTATATAAGCAGAGCTGGTTTAGTGAACCGTCAGATCTCGAGCTCGG
TACCTAATACGACACACTATAAGGAAATAAGAGAGAAAAGAAGAGTAAGAAGAAATATAAGAGCCACCA
TGAAACGGACAGCCGACGGAAGCGAGTTCGAGTCACCAAAGAAGAAGCGGAAAGTCTCTGAGGTG
GAGTTTTCCCACGAGTACTGGATGAGACATGCCCTGACCCTGGCCAAGAGGGCACGGGATGAGAGG
GAGGTGCCTGTGGGAGCCGTGCTGGTGCTGAACAATAGAGTGATCGGCGAGGGCTGGAACAGAGC
CATCGGCCTGCACGACCCAACAGCCCATGCCGAAATTATGGCCCTGAGACAGGGCGGCCTGGTCAT
GCAGAACTACAGACTGATTGACGCCACCCTGTACGTGACATTCGAGCCTTGCGTGATGTGCGCCGGC
GCCATGATCCACTCTAGGATCGGCCGCGTGGTGTTTGGCGTGAGGCAGTCAAAAAGAGGCGCCGCA
GGCTCCCTGATGAACGTGCTGAACTACCCCGGCATGAATCACCGCGTCGAAATTACCGAGGGAATCC
TGGCAGATGAATGTGCCGCCCTGACCTGCGATTTCTATCGGATGCCTAGACAGGTGTTCAATGCTCAG
AAGAAGGCCCAGAGCTCCATCAACTCCGGAGGATCTAGCGGAGGCTCCTCTGGCTCTGAGACACCT
GGCACAAGCGAGAGCGCAACACCTGAAAGCAGCGGGGGCAGCAGCGGGGGGTCAGACAAGAAGT
ACAGCATCGGCCTGACCATCGGCACCAACTCTGTGGGCTGGGCCGTGATCACCGACGAGTACAAGG
TGCCCAGCAAGAAATTCAAGGTGCTGGGCAACACCGACCGGCACAGCATCAAGAAGAACCTGATCG
GAGCCCTGCTGTTCGACAGCGGCGAAACAGCCGAGGCCACCCGGCTGAAGAGAACCGCCAGAAGA
AGATACACCAGACGGAAGAACCGGATCTGCTATCTGCAAGAGATCTTCAGCAACGAGATGGCCAAGG
TGGACGACAGCTTCTTCCACAGACTGGAAGAGTCCTTCCTGGTGGAAGAGGATAAGAAGCACGAGC
GGCACCCCATCTTCGGCAACATCGTGGACGAGGTGGCCTACCACGAGAAGTACCCCACCATCTACCA
CCTGAGAAAGAAACTGGTGGACAGCACCGACAAGGCCGACCTGCGGCTGATCTATCTGGCCCTGGC
CCACATGATCAAGTTCCGGGGCCACTTCCTGATCGAGGGCGACCTGAACCCCGACAACAGCGACGT
GGACAAGCTGTTCATCCAGCTGGTGCAGACCTACAACCAGCTGTTCGAGGAAAACCCCATCAACGCC
AGCGGCGTGGACGCCAAGGCCATCCTGTCTGCCAGACTGAGCAAGAGCAGACGGCTGGAAAATCTG
ATCGCCCAGCTGCCCGGCGAGAAGAAGAATGGCCTGTTCGGAAACCTGATTGCCCTGAGCCTGGGC
CTGACCCCCAACTTCAAGAGCAACTTCGACCTGGCCGAGGATGCCAAACTGCAGCTGAGCAAGGAC
ACCTACGACGACGACCTGGACAACCTGCTGGCCCAGATCGGCGACCAGTACGCCGACCTGTTTCTG
GCCGCCAAGAACCTGTCCGACGCCATCCTGCTGAGCGACATCCTGAGAGTGAACACCGAGATCACC
AAGGCCCCCCTGAGCGCCTCTATGGTGAAGAGATACGACGAGCACCACCAGGACCTGACCCTGCTG
AAAGCTCTCGTGCGGCAGCAGCTGCCTGAGAAGTACAAAGAGATTTTCTTCGACCAGAGCAAGAACG
GCTACGCCGGCTACATTGACGGCGGAGCCAGCCAGGAAGAGTTCTACAAGTTCATCAAGCCCATCCT
GGAAAAGATGGACGGCACCGAGGAACTGCTCGTGAAGCTGAACAGAGAGGACCTGCTGCGGAAGC
AGCGGACCTTCGACAACGGCATTATCCCCCACCAGATCCACCTGGGAGAGCTGCACGCCATTCTGC
GGCGGCAGGGCGATTTTTACCCATTCCTGAAGGACAACCGGGAAAAGATCGAGAAGATCCTGACCTT
CCGCATCCCCTACTACGTGGGCCCTCTGGCCAGGGGAAACAGCAGATTCGCCTGGATGACCAGAAA
GAGCGAGGAAACCATCACCCCCTGGAACTTCGAGGAAGTGGTGGACAAGGGCGCTTCCGCCCAGA
GCTTCATCGAGCGGATGACCAACTTCGATAAGAACCTGCCCAACGAGAAGGTGCTGCCCAAGCACA
GCCTGCTGTACGAGTACTTCACCGTGTATAACGAGCTGACCAAAGTGAAATACGTGACCGAGGGAAT
GAGAAAGCCCGCCTTCCTGAGCGGCGAGCAGAAAAAGGCCATCGTGGACCTGCTGTTCAAGACCAA
CCGGAAAGTGACCGTGAAGCAGCTGAAAGAGGACTACTTCAAGAAAATCGAGTGCTTCGACTCCGTG
GAAATCTCCGGCGTGGAAGATCGGTTCAACGCCTCCCTGGGCACATACCACGATCTGCTGAAAATTAT
CAAGGACAAGGACTTCCTGGACAATGAGGAAAACGAGGACATTCTGGAAGATATCGTGCTGACCCTG
ACACTGTTTGAGGACAGAGAGATGATCGAGGAACGGCTGAAAACCTATGCCCACCTGTTCGACGACA
AAGTGATGAAGCAGCTGAAGCGGCTGAGATACACCGGCTGGGGCAGGCTGAGCCGGAAGCTGATCA
ACGGCATCCGGGACAAGCAGTCCGGCAAGACAATCCTGGATTTCCTGAAGTCCGACGGCTTCGCCA
ACAGAAACTTCATGCAGCTGATCCACGACGACAGCCTGACCTTTAAAGAGGACATCCAGAAAGCCCA

GGTGTCCGGCCAGGGCGATAGCCTGCACGAGCACATTGCCAATCTGGCCGGCAGCCCCGCCATTAA
GAAGGGCATCCTGCAGACAGTGAAGGTGGTGGACGAGCTCGTGAAAGTGATGGGCGGCCACAAGC
CCGAGAACATCGTGATCGAAATGGCCAGAGAGAACCAGACCACCCAGAAGGGACAGAAGAACAGCC
GCGAGAGAATGAAGCGGATCGAAGAGGGCATCAAAGAGCTGGGCAGCCAGATCCTGAAAGAACACC
CCGTGGAAAACACCCAGCTGCAGAACGAGAAGCTGTACCTGTACTACCTGCAGAATGGGCGGGATAT
GTACGTGGACCAGGAACTGGACATCAACCGGCTGTCCGACTACGATGTGGACCATATCGTGCCTCAG
AGCTTTCTGAAGGACGACTCCATCGACAACAAGGTGCTGACCAGAAGCGACAAGAACCGGGGCAAG
AGCGACAACGTGCCCTCCGAAGAGGTCGTGAAGAAGATGAAGAACTACTGGCGGCAGCTGCTGAAC
GCCAAGCTGATTACCCAGAGAAAGTTCGACAATCTGACCAAGGCCGAAAGAGGCGGCCTGAGCGAA
CTGGATAAGGCCGGCTTCATCAAGAGACAGCTGGTGGAAACCCGGCAGATCACAAAGCACGTGGCA
CAGATCCTGGACTCCCGGATGAACACTAAGTACGACGAGAATGACAAGCTGATCCGGGAAGTGAAAG
TGATCACCCTGAAGTCCAAGCTGGTGTCCGATTTCCGGAAGGATTTCCAGTTTTACAAAGTGCGCGA
GATCAACAACTACCACCACGCCCACGACGCCTACCTGAACGCCGTCGTGGGAACCGCCCTGATCAA
AAAGTACCCTAAGCTGGAAAGCGAGTTCGTGTACGGCGACTACAAGGTGTACGACGTGCGGAAGATG
ATCGCCAAGAGCGAGCAGGAAATCGGCAAGGCTACCGCCAAGTACTTCTTCTACAGCAACATCATGA
ACTTTTTCAAGACCGAGATTACCCTGGCCAACGGCGAGATCCGGAAGCGGCCTCTGATCGAGACAAA
CGGCGAAACCGGGGAGATCGTGTGGGATAAGGGCCGGGATTTTGCCACCGTGCGGAAAGTGCTGA
GCATGCCCCAAGTGAATATCGTGAAAAAGACCGAGGTGCAGACAGGCGGCTTCAGCAAAGAGTCTAT
CCTGCCCAAGGGCAACAGCGATAAGCTGATCGCCAGAAAGAAGGACTGGGACCCTAAGAAGTACGG
CGGCTTCAACAGCCCCACCGTGGCCTATTCTGTGCTGGTGGTGGCCAAAGTGGAAAAGGGCAAGTC
CAAGAAACTGAAGAGTGTGAAAGAGCTGCTGGGGATCACCATCATGGAAAGAAGCAGCTTCGAGAA
GAATCCCATCGGCTTTCTGGAAGCCAAGGGCTACAAAGAAGTGAAAAAGGACCTGATCATCAAGCTG
CCTAAGTACTCCCTGTTCGAGCTGGAAAACGGCCGGAAGAGAATGCTGGCCTCTGCCAGCGTGCTG
CATAAGGGAAACGAACTGGCCCTGCCCTCCAAATATGTGAACTTCCTGTACCTGGCCAGCCACTATGA
GAAGCTGAAGGGCTCCAGCGAGGATAATAAACAGAAACAGCTGTTTGTGGAACAGCACAAGCACTAC
CTGGACGAGATCATCGAGCAGATCAGCGAGTTCTCCAAGAGAGTGATCCTGGCCGACGCTAATCTGG
ACAAAGTGCTGTCCGCCTACAACAAGCACCGGGATAAGCCCATCAGAGAGCAGGCCGAGAATATCAT
CCACCTGTTTACCCTGACCAATCTGGGAGCCAGCGCCGCCTTCAAGTACTTTGACACCACCATCGGC
CGGAAGCTGTACACCAGCACCAAAGAGGTGCTGGACGCCACCCTGATCCACCAGAGCATCACCGGC
CTGTACGAGACACGGATCGACCTGTCTCAGCTGGGAGGTGACTCTGGCGGCTCAAAAAGAACCGCC
GACGGCAGCGAATTCGAGCCCAAGAAGAAGAGGAAAGTCTAATTAATTAAGCTGCCTTCTGCGGGGC
TTGCCTTCTGGCCATGCCCTTCTTCTCTCCCTTGCACCTGTACCTCTTGGTCTTTGAATAAAGCCTGA
GTAGGAAGCGACTGTGCCTTCTAGTTGCCAGCCATCTGTTGTTTGCCCCTCCCCCGTGCCTTCCTTG
ACCCTGGAAGGTGCCACTCCCACTGTCCTTTCCTAATAAAATGAGAAATTGCATCGCATTGTCTGAG
TAGGTGTCATTCTATTCTGGGGGGTGGGGTGGGGCAGGACAGCAAGGGGGAGGATTGGGAAGACAA
TAGCAGGCATGCTGGGGATGCGGTGGGCTCTATGG

Start	End	Feature Description
1	380	CMV enhancer
381	619	CMV promoter
620	636	T7 promoter with mismatch
637	683	5' UTR
684	740	BP NLS
741	1238	Engineered TadA* variant ABE9 (TadA-8e N108Q L145T)
1239	1334	(SGGS)2 – XTEN – (SGGS)2 linker
1335	5435	Cas9(D10T) Sp-NRTH PAM variant
5436	5501	Linker + SV40 BP NLS

5502	5605	3’ UTR
5606	5830	bGH poly(A) signal

Amino acid sequence
MKRTADGSEFESPKKKRKVSEVEFSHEYWMRHALTLAKRARDEREVPVGAVLVLNNRVIGEGWNRAIGL
HDPTAHAEIMALRQGGLVMQNYRLIDATLYVTFEPCVMCAGAMIHSRIGRVVFGVRQSKRGAAGSLMNVL
NYPGMNHRVEITEGILADECAALTCDFYRMPRQVFNAQKKAQSSINSGGSSGGSSGSETPGTSESATPE
SSGGSSGGSDKKYSIGLTIGTNSVGWAVITDEYKVPSKKFKVLGNTDRHSIKKNLIGALLFDSGETAEATRL
KRTARRRYTRRKNRICYLQEIFSNEMAKVDDSFFHRLEESFLVEEDKKHERHPIFGNIVDEVAYHEKYPTIY
HLRKKLVDSTDKADLRLIYLALAHMIKFRGHFLIEGDLNPDNSDVDKLFIQLVQTYNQLFEENPINASGVDA
KAILSARLSKSRRLENLIAQLPGEKKNGLFGNLIALSLGLTPNFKSNFDLAEDAKLQLSKDTYDDDLDNLLA
QIGDQYADLFLAAKNLSDAILLSDILRVNTEITKAPLSASMVKRYDEHHQDLTLLKALVRQQLPEKYKEIFFD
QSKNGYAGYIDGGASQEEFYKFIKPILEKMDGTEELLVKLNREDLLRKQRTFDNGIIPHQIHLGELHAILRR
QGDFYPFLKDNREKIEKILTFRIPYYVGPLARGNSRFAWMTRKSEETITPWNFEEVVDKGASAQSFIERMT
NFDKNLPNEKVLPKHSLLYEYFTVYNELTKVKYVTEGMRKPAFLSGEQKKAIVDLLFKTNRKVTVKQLKED
YFKKIECFDSVEISGVEDRFNASLGTYHDLLKIIKDKDFLDNEENEDILEDIVLTLTLFEDREMIEERLKTYAH
LFDDKVMKQLKRLRYTGWGRLSRKLINGIRDKQSGKTILDFLKSDGFANRNFMQLIHDDSLTFKEDIQKAQ
VSGQGDSLHEHIANLAGSPAIKKGILQTVKVVDELVKVMGGHKPENIVIEMARENQTTQKGQKNSRERMK
RIEEGIKELGSQILKEHPVENTQLQNEKLYLYYLQNGRDMYVDQELDINRLSDYDVDHIVPQSFLKDDSIDN
KVLTRSDKNRGKSDNVPSEEVVKKMKNYWRQLLNAKLITQRKFDNLTKAERGGLSELDKAGFIKRQLVET
RQITKHVAQILDSRMNTKYDENDKLIREVKVITLKSKLVSDFRKDFQFYKVREINNYHHAHDAYLNAVVGTA
LIKKYPKLESEFVYGDYKVYDVRKMIAKSEQEIGKATAKYFFYSNIMNFFKTEITLANGEIRKRPLIETNGET
GEIVWDKGRDFATVRKVLSMPQVNIVKKTEVQTGGFSKESILPKGNSDKLIARKKDWDPKKYGGFNSPTV
AYSVLVVAKVEKGKSKKLKSVKELLGITIMERSSFEKNPIGFLEAKGYKEVKKDLIIKLPKYSLFELENGRKR
MLASASVLHKGNELALPSKYVNFLYLASHYEKLKGSSEDNKQKQLFVEQHKHYLDEIIEQISEFSKRVILAD
ANLDKVLSAYNKHRDKPIREQAENIIHLFTLTNLGASAAFKYFDTTIGRKLYTSTKEVLDATLIHQSITGLYET
RIDLSQLGGDSGGSKRTADGSEFEPKKKRKV

Start	End	Feature Description
1	19	BP NLS
20	185	Engineered TadA* variant 8e
186	217	(SGGS)2 – XTEN – (SGGS)2 linker
218	1584	Cas9(D10T) Sp-NRTH PAM variant
1585	1605	Linker + SV40 BP NLS

ABE7.10 – SpCas9 (D10A)
Plasmid ID: pHS0228

DNA sequence
GACATTGATTATTGACTAGTTATTAATAGTAATCAATTACGGGGTCATTAGTTCATAGCCCATATATGGAG
TTCCGCGTTACATAACTTACGGTAAATGGCCCGCCTGGCTGACCGCCCAACGACCCCCGCCCATTGA
CGTCAATAATGACGTATGTTCCCATAGTAACGCCAATAGGGACTTTCCATTGACGTCAATGGGTGGAGT
ATTTACGGTAAACTGCCCACTTGGCAGTACATCAAGTGTATCATATGCCAAGTACGCCCCCTATTGACG
TCAATGACGGTAAATGGCCCGCCTGGCATTATGCCCAGTACATGACCTTATGGGACTTTCCTACTTGG
CAGTACATCTACGTATTAGTCATCGCTATTACCATGGTGATGCGGTTTTGGCAGTACATCAATGGGCGT
GGATAGCGGTTTGACTCACGGGGATTTCCAAGTCTCCACCCCATTGACGTCAATGGGAGTTTGTTTTG
GCACCAAAATCAACGGGACTTTCCAAAATGTCGTAACAACTCCGCCCCATTGACGCAAATGGGCGGT
AGGCGTGTACGGTGGGAGGTCTATATAAGCAGAGCTGGTTTAGTGAACCGTCAGATCTCGAGCTCGG
TACCTAATACGACACACTATAAGGAAATAAGAGAGAAAAGAAGAGTAAGAAGAAATATAAGAGCCACCA
TGAAACGGACAGCCGACGGAAGCGAGTTCGAGTCACCAAAGAAGAAGCGGAAAGTCTCTGAAGTCG
AGTTTAGCCACGAGTATTGGATGAGGCACGCACTGACCCTGGCAAAGCGAGCATGGGATGAAAGAGA
AGTCCCCGTGGGCGCCGTGCTGGTGCACAACAATAGAGTGATCGGAGAGGGATGGAACAGGCCAAT
CGGCCGCCACGACCCTACCGCACACGCAGAGATCATGGCACTGAGGCAGGGAGGCCTGGTCATGC
AGAATTACCGCCTGATCGATGCCACCCTGTATGTGACACTGGAGCCATGCGTGATGTGCGCAGGAGC
AATGATCCACAGCAGGATCGGAAGAGTGGTGTTCGGAGCACGGGACGCCAAGACCGGCGCAGCAG
GCTCCCTGATGGATGTGCTGCACCACCCCGGCATGAACCACCGGGTGGAGATCACAGAGGGAATCC
TGGCAGACGAGTGCGCCGCCCTGCTGAGCGATTTCTTTAGAATGCGGAGACAGGAGATCAAGGCCC
AGAAGAAGGCACAGAGCTCCACCGACTCTGGAGGATCTAGCGGAGGATCCTCTGGAAGCGAGACAC
CAGGCACAAGCGAGTCCGCCACACCAGAGAGCTCCGGCGGCTCCTCCGGAGGATCCTCTGAGGTG
GAGTTTTCCCACGAGTACTGGATGAGACATGCCCTGACCCTGGCCAAGAGGGCACGCGATGAGAGG
GAGGTGCCTGTGGGAGCCGTGCTGGTGCTGAACAATAGAGTGATCGGCGAGGGCTGGAACAGAGC
CATCGGCCTGCACGACCCAACAGCCCATGCCGAAATTATGGCCCTGAGACAGGGCGGCCTGGTCAT
GCAGAACTACAGACTGATTGACGCCACCCTGTACGTGACATTCGAGCCTTGCGTGATGTGCGCCGGC
GCCATGATCCACTCTAGGATCGGCCGCGTGGTGTTTGGCGTGAGGAACGCAAAAACCGGCGCCGCA
GGCTCCCTGATGGACGTGCTGCACTACCCCGGCATGAATCACCGCGTCGAAATTACCGAGGGAATCC
TGGCAGATGAATGTGCCGCCCTGCTGTGCTATTTCTTTCGGATGCCTAGACAGGTGTTCAATGCTCAG
AAGAAGGCCCAGAGCTCCACCGACTCCGGAGGATCTAGCGGAGGCTCCTCTGGCTCTGAGACACCT
GGCACAAGCGAGAGCGCAACACCTGAAAGCAGCGGGGGCAGCAGCGGGGGGTCAGACAAGAAGT
ACAGCATCGGCCTGGCCATCGGCACCAACTCTGTGGGCTGGGCCGTGATCACCGACGAGTACAAGG
TGCCCAGCAAGAAATTCAAGGTGCTGGGCAACACCGACCGGCACAGCATCAAGAAGAACCTGATCG
GAGCCCTGCTGTTCGACAGCGGCGAAACAGCCGAGGCCACCCGGCTGAAGAGAACCGCCAGAAGA
AGATACACCAGACGGAAGAACCGGATCTGCTATCTGCAAGAGATCTTCAGCAACGAGATGGCCAAGG
TGGACGACAGCTTCTTCCACAGACTGGAAGAGTCCTTCCTGGTGGAAGAGGATAAGAAGCACGAGC
GGCACCCCATCTTCGGCAACATCGTGGACGAGGTGGCCTACCACGAGAAGTACCCCACCATCTACCA
CCTGAGAAAGAAACTGGTGGACAGCACCGACAAGGCCGACCTGCGGCTGATCTATCTGGCCCTGGC
CCACATGATCAAGTTCCGGGGCCACTTCCTGATCGAGGGCGACCTGAACCCCGACAACAGCGACGT
GGACAAGCTGTTCATCCAGCTGGTGCAGACCTACAACCAGCTGTTCGAGGAAAACCCCATCAACGCC
AGCGGCGTGGACGCCAAGGCCATCCTGTCTGCCAGACTGAGCAAGAGCAGACGGCTGGAAAATCTG
ATCGCCCAGCTGCCCGGCGAGAAGAAGAATGGCCTGTTCGGAAACCTGATTGCCCTGAGCCTGGGC
CTGACCCCCAACTTCAAGAGCAACTTCGACCTGGCCGAGGATGCCAAACTGCAGCTGAGCAAGGAC
ACCTACGACGACGACCTGGACAACCTGCTGGCCCAGATCGGCGACCAGTACGCCGACCTGTTTCTG
GCCGCCAAGAACCTGTCCGACGCCATCCTGCTGAGCGACATCCTGAGAGTGAACACCGAGATCACC
AAGGCCCCCCTGAGCGCCTCTATGATCAAGAGATACGACGAGCACCACCAGGACCTGACCCTGCTG
AAAGCTCTCGTGCGGCAGCAGCTGCCTGAGAAGTACAAAGAGATTTTCTTCGACCAGAGCAAGAACG
GCTACGCCGGCTACATTGACGGCGGAGCCAGCCAGGAAGAGTTCTACAAGTTCATCAAGCCCATCCT
GGAAAAGATGGACGGCACCGAGGAACTGCTCGTGAAGCTGAACAGAGAGGACCTGCTGCGGAAGC
AGCGGACCTTCGACAACGGCAGCATCCCCCACCAGATCCACCTGGGAGAGCTGCACGCCATTCTGC
GGCGGCAGGAAGATTTTTACCCATTCCTGAAGGACAACCGGGAAAAGATCGAGAAGATCCTGACCTT
CCGCATCCCCTACTACGTGGGCCCTCTGGCCAGGGGAAACAGCAGATTCGCCTGGATGACCAGAAA
GAGCGAGGAAACCATCACCCCCTGGAACTTCGAGGAAGTGGTGGACAAGGGCGCTTCCGCCCAGA
GCTTCATCGAGCGGATGACCAACTTCGATAAGAACCTGCCCAACGAGAAGGTGCTGCCCAAGCACA

GCCTGCTGTACGAGTACTTCACCGTGTATAACGAGCTGACCAAAGTGAAATACGTGACCGAGGGAAT
GAGAAAGCCCGCCTTCCTGAGCGGCGAGCAGAAAAAGGCCATCGTGGACCTGCTGTTCAAGACCAA
CCGGAAAGTGACCGTGAAGCAGCTGAAAGAGGACTACTTCAAGAAAATCGAGTGCTTCGACTCCGTG
GAAATCTCCGGCGTGGAAGATCGGTTCAACGCCTCCCTGGGCACATACCACGATCTGCTGAAAATTAT
CAAGGACAAGGACTTCCTGGACAATGAGGAAAACGAGGACATTCTGGAAGATATCGTGCTGACCCTG
ACACTGTTTGAGGACAGAGAGATGATCGAGGAACGGCTGAAAACCTATGCCCACCTGTTCGACGACA
AAGTGATGAAGCAGCTGAAGCGGCGGAGATACACCGGCTGGGGCAGGCTGAGCCGGAAGCTGATC
AACGGCATCCGGGACAAGCAGTCCGGCAAGACAATCCTGGATTTCCTGAAGTCCGACGGCTTCGCC
AACAGAAACTTCATGCAGCTGATCCACGACGACAGCCTGACCTTTAAAGAGGACATCCAGAAAGCCC
AGGTGTCCGGCCAGGGCGATAGCCTGCACGAGCACATTGCCAATCTGGCCGGCAGCCCCGCCATTA
AGAAGGGCATCCTGCAGACAGTGAAGGTGGTGGACGAGCTCGTGAAAGTGATGGGCCGGCACAAG
CCCGAGAACATCGTGATCGAAATGGCCAGAGAGAACCAGACCACCCAGAAGGGACAGAAGAACAGC
CGCGAGAGAATGAAGCGGATCGAAGAGGGCATCAAAGAGCTGGGCAGCCAGATCCTGAAAGAACAC
CCCGTGGAAAACACCCAGCTGCAGAACGAGAAGCTGTACCTGTACTACCTGCAGAATGGGCGGGATA
TGTACGTGGACCAGGAACTGGACATCAACCGGCTGTCCGACTACGATGTGGACCATATCGTGCCTCA
GAGCTTTCTGAAGGACGACTCCATCGACAACAAGGTGCTGACCAGAAGCGACAAGAACCGGGGCAA
GAGCGACAACGTGCCCTCCGAAGAGGTCGTGAAGAAGATGAAGAACTACTGGCGGCAGCTGCTGAA
CGCCAAGCTGATTACCCAGAGAAAGTTCGACAATCTGACCAAGGCCGAGAGAGGCGGCCTGAGCGA
ACTGGATAAGGCCGGCTTCATCAAGAGACAGCTGGTGGAAACCCGGCAGATCACAAAGCACGTGGC
ACAGATCCTGGACTCCCGGATGAACACTAAGTACGACGAGAATGACAAGCTGATCCGGGAAGTGAAA
GTGATCACCCTGAAGTCCAAGCTGGTGTCCGATTTCCGGAAGGATTTCCAGTTTTACAAAGTGCGCG
AGATCAACAACTACCACCACGCCCACGACGCCTACCTGAACGCCGTCGTGGGAACCGCCCTGATCA
AAAAGTACCCTAAGCTGGAAAGCGAGTTCGTGTACGGCGACTACAAGGTGTACGACGTGCGGAAGAT
GATCGCCAAGAGCGAGCAGGAAATCGGCAAGGCTACCGCCAAGTACTTCTTCTACAGCAACATCATG
AACTTTTTCAAGACCGAGATTACCCTGGCCAACGGCGAGATCCGGAAGCGGCCTCTGATCGAGACAA
ACGGCGAAACCGGGGAGATCGTGTGGGATAAGGGCCGGGATTTTGCCACCGTGCGGAAAGTGCTG
AGCATGCCCCAAGTGAATATCGTGAAAAAGACCGAGGTGCAGACAGGCGGCTTCAGCAAAGAGTCTA
TCCTGCCCAAGAGGAACAGCGATAAGCTGATCGCCAGAAAGAAGGACTGGGACCCTAAGAAGTACG
GCGGCTTCGACAGCCCCACCGTGGCCTATTCTGTGCTGGTGGTGGCCAAAGTGGAAAAGGGCAAGT
CCAAGAAACTGAAGAGTGTGAAAGAGCTGCTGGGGATCACCATCATGGAAAGAAGCAGCTTCGAGAA
GAATCCCATCGACTTTCTGGAAGCCAAGGGCTACAAAGAAGTGAAAAAGGACCTGATCATCAAGCTG
CCTAAGTACTCCCTGTTCGAGCTGGAAAACGGCCGGAAGAGAATGCTGGCCTCTGCCGGCGAACTG
CAGAAGGGAAACGAACTGGCCCTGCCCTCCAAATATGTGAACTTCCTGTACCTGGCCAGCCACTATG
AGAAGCTGAAGGGCTCCCCCGAGGATAATGAGCAGAAACAGCTGTTTGTGGAACAGCACAAGCACTA
CCTGGACGAGATCATCGAGCAGATCAGCGAGTTCTCCAAGAGAGTGATCCTGGCCGACGCTAATCTG
GACAAAGTGCTGTCCGCCTACAACAAGCACCGGGATAAGCCCATCAGAGAGCAGGCCGAGAATATCA
TCCACCTGTTTACCCTGACCAATCTGGGAGCCCCTGCCGCCTTCAAGTACTTTGACACCACCATCGA
CCGGAAGAGGTACACCAGCACCAAAGAGGTGCTGGACGCCACCCTGATCCACCAGAGCATCACCGG
CCTGTACGAGACACGGATCGACCTGTCTCAGCTGGGAGGTGACTCTGGCGGCTCAAAAAGAACCGC
CGACGGCAGCGAATTCGAGCCCAAGAAGAAGAGGAAAGTCTAATTAATTAAGCTGCCTTCTGCGGGG
CTTGCCTTCTGGCCATGCCCTTCTTCTCTCCCTTGCACCTGTACCTCTTGGTCTTTGAATAAAGCCTG
AGTAGGAAGCGACTGTGCCTTCTAGTTGCCAGCCATCTGTTGTTTGCCCCTCCCCCGTGCCTTCCTT
GACCCTGGAAGGTGCCACTCCCACTGTCCTTTCCTAATAAAATGAGAAATTGCATCGCATTGTCTGA
GTAGGTGTCATTCTATTCTGGGGGGTGGGGTGGGGCAGGACAGCAAGGGGGAGGATTGGGAAGAC
AATAGCAGGCATGCTGGGATGCGGTGGGCTCTATGG

Start	End	Feature Description
1	380	CMV enhancer
381	619	CMV promoter
620	636	T7 promoter with mismatch
637	683	5’ UTR

684	740	BP NLS
741	1238	TadA wild type
1239	1334	(SGGS)2 – XTEN – (SGGS)2 linker
1335	1832	Engineered TadA* variant 7.10
1833	1928	(SGGS)2 – XTEN – (SGGS)2 linker
1929	6029	Cas9(D10A) SpCas9 wild-type
6030	6095	Linker + SV40 BP NLS
6096	6196	3' UTR
6197	6424	bGH poly(A) signal

Amino acid sequence

MKRTADGSEFESPKKKRKVSEVEFSHEYWMRHALTLAKRAWDEREVPVGAVLVHNNRVIGEGWNRPIG
RHDPTAHAEIMALRQGGLVMQNYRLIDATLYVTLEPCVMCAGAMIHSRIGRVVFGARDAKTGAAGSLMDV
LHHPGMNHRVEITEGILADECAALLSDFFRMRRQEIKAQKKAQSSTDSGGSSGGSSGSETPGTSESATP
ESSGGSSGGSSEVEFSHEYWMRHALTLAKRARDEREVPVGAVLVLNNRVIGEGWNRAIGLHDPTAHAEI
MALRQGGLVMQNYRLIDATLYVTFEPCVMCAGAMIHSRIGRVVFGVRNAKTGAAGSLMDVLHYPGMNHR
VEITEGILADECAALLCYFFRMPRQVFNAQKKAQSSTDSGGSSGGSSGSETPGTSESATPESSGGSSGG
SDKKYSIGLAIGTNSVGWAVITDEYKVPSKKFKVLGNTDRHSIKKNLIGALLFDSGETAEATRLKRTARRRY
TRRKNRICYLQEIFSNEMAKVDDSFFHRLEESFLVEEDKKHERHPIFGNIVDEVAYHEKYPTIYHLRKKLVD
STDKADLRLIYLALAHMIKFRGHFLIEGDLNPDNSDVDKLFIQLVQTYNQLFEENPINASGVDAKAILSARLS
KSRRLENLIAQLPGEKKNGLFGNLIALSLGLTPNFKSNFDLAEDAKLQLSKDTYDDDLDNLLAQIGDQYADL
FLAAKNLSDAILLSDILRVNTEITKAPLSASMIKRYDEHHQDLTLLKALVRQQLPEKYKEIFFDQSKNGYAGYI
DGGASQEEFYKFIKPILEKMDGTEELLVKLNREDLLRKQRTFDNGSIPHQIHLGELHAILRRQEDFYPFLKD
NREKIEKILTFRIPYYVGPLARGNSRFAWMTRKSEETITPWNFEEVVDKGASAQSFIERMTNFDKNLPNEK
VLPKHSLLYEYFTVYNELTKVKYVTEGMRKPAFLSGEQKKAIVDLLFKTNRKVTVKQLKEDYFKKIECFDSV
EISGVEDRFNASLGTYHDLLKIIKDKDFLDNEENEDILEDIVLTLTLFEDREMIEERLKTYAHLFDDKVMKQLK
RRRYTGWGRLSRKLINGIRDKQSGKTILDFLKSDGFANRNFMQLIHDDSLTFKEDIQKAQVSGQGDSLHE
HIANLAGSPAIKKGILQTVKVVDELVKVMGRHKPENIVIEMARENQTTQKGQKNSRERMKRIEEGIKELGS
QILKEHPVENTQLQNEKLYLYYLQNGRDMYVDQELDINRLSDYDVDHIVPQSFLKDDSIDNKVLTRSDKNR
GKSDNVPSEEVVKKMKNYWRQLLNAKLITQRKFDNLTKAERGGLSELDKAGFIKRQLVETRQITKHVAQIL
DSRMNTKYDENDKLIREVKVITLKSKLVSDFRKDFQFYKVREINNYHHAHDAYLNAVVGTALIKKYPKLESE
FVYGDYKVYDVRKMIAKSEQEIGKATAKYFFYSNIMNFFKTEITLANGEIRKRPLIETNGETGEIVWDKGRD
FATVRKVLSMPQVNIVKKTEVQTGGFSKESILPKRNSDKLIARKKDWDPKKYGGFDSPTVAYSVLVVAKVE
KGKSKKLKSVKELLGITIMERSSFEKNPIDFLEAKGYKEVKKDLIIKLPKYSLFELENGRKRMLASAGELQK
GNELALPSKYVNFLYLASHYEKLKGSPEDNEQKQLFVEQHKHYLDEIIEQISEFSKRVILADANLDKVLSAY
NKHRDKPIREQAENIIHLFTLTNLGAPAAFKYFDTTIDRKRYTSTKEVLDATLIHQSITGLYETRIDLSQLGGD
SGGSKRTADGSEFEPKKKRKV

Start	End	Feature Description
1	19	BP NLS
20	185	TadA wild type
186	217	(SGGS)2 – XTEN – (SGGS)2 linker
218	383	Engineered TadA* variant 7.10

384	415	(SGGS)2 – XTEN – (SGGS)2 linker
416	1782	Cas9(D10A) SpCas9 wild-type
1783	1803	Linker + SV40 BP NLS

ABE8e – SpCas9 (D10A)
Plasmid ID: pHS0026

DNA sequence

GACATTGATTATTGACTAGTTATTAATAGTAATCAATTACGGGGTCATTAGTTCATAGCCCATATATGGAG
TTCCGCGTTACATAACTTACGGTAAATGGCCCGCCTGGCTGACCGCCCAACGACCCCCGCCCATTGA
CGTCAATAATGACGTATGTTCCCATAGTAACGCCAATAGGGACTTTCCATTGACGTCAATGGGTGGAGT
ATTTACGGTAAACTGCCCACTTGGCAGTACATCAAGTGTATCATATGCCAAGTACGCCCCCTATTGACG
TCAATGACGGTAAATGGCCCGCCTGGCATTATGCCCAGTACATGACCTTATGGGACTTTCCTACTTGG
CAGTACATCTACGTATTAGTCATCGCTATTACCATGGTGATGCGGTTTTGGCAGTACATCAATGGGCGT
GGATAGCGGTTTGACTCACGGGGATTTCCAAGTCTCCACCCCATTGACGTCAATGGGAGTTTGTTTTG
GCACCAAAATCAACGGGACTTTCCAAAATGTCGTAACAACTCCGCCCCATTGACGCAAATGGGCGGT
AGGCGTGTACGGTGGGAGGTCTATATAAGCAGAGCTGGTTTAGTGAACCGTCAGATCTCGAGCTCGG
TACCTAATACGACACACTATAAGGAAATAAGAGAGAAAAGAAGAGTAAGAAGAAATATAAGAGCCACCA
TGAAACGGACAGCCGACGGAAGCGAGTTCGAGTCACCAAAGAAGAAGCGGAAAGTCTCTGAGGTG
GAGTTTTCCCACGAGTACTGGATGAGACATGCCCTGACCCTGGCCAAGAGGGCACGGGATGAGAGG
GAGGTGCCTGTGGGAGCCGTGCTGGTGCTGAACAATAGAGTGATCGGCGAGGGCTGGAACAGAGC
CATCGGCCTGCACGACCCAACAGCCCATGCCGAAATTATGGCCCTGAGACAGGGCGGCCTGGTCAT
GCAGAACTACAGACTGATTGACGCCACCCTGTACGTGACATTCGAGCCTTGCGTGATGTGCGCCGGC
GCCATGATCCACTCTAGGATCGGCCGCGTGGTGTTTGGCGTGAGGAACTCAAAAAGAGGCGCCGCA
GGCTCCCTGATGAACGTGCTGAACTACCCCGGCATGAATCACCGCGTCGAAATTACCGAGGGAATCC
TGGCAGATGAATGTGCCGCCCTGCTGTGCGATTTCTATCGGATGCCTAGACAGGTGTTCAATGCTCAG
AAGAAGGCCCAGAGCTCCATCAACTCCGGAGGATCTAGCGGAGGCTCCTCTGGCTCTGAGACACCT
GGCACAAGCGAGAGCGCAACACCTGAAAGCAGCGGGGGCAGCAGCGGGGGGTCAGACAAGAAGT
ACAGCATCGGCCTGGCCATCGGCACCAACTCTGTGGGCTGGGCCGTGATCACCGACGAGTACAAGG
TGCCCAGCAAGAAATTCAAGGTGCTGGGCAACACCGACCGGCACAGCATCAAGAAGAACCTGATCG
GAGCCCTGCTGTTCGACAGCGGCGAAACAGCCGAGGCCACCCGGCTGAAGAGAACCGCCAGAAGA
AGATACACCAGACGGAAGAACCGGATCTGCTATCTGCAAGAGATCTTCAGCAACGAGATGGCCAAGG
TGGACGACAGCTTCTTCCACAGACTGGAAGAGTCCTTCCTGGTGGAAGAGGATAAGAAGCACGAGC
GGCACCCCATCTTCGGCAACATCGTGGACGAGGTGGCCTACCACGAGAAGTACCCCACCATCTACCA
CCTGAGAAAGAAACTGGTGGACAGCACCGACAAGGCCGACCTGCGGCTGATCTATCTGGCCCTGGC
CCACATGATCAAGTTCCGGGGCCACTTCCTGATCGAGGGCGACCTGAACCCCGACAACAGCGACGT
GGACAAGCTGTTCATCCAGCTGGTGCAGACCTACAACCAGCTGTTCGAGGAAAACCCCATCAACGCC
AGCGGCGTGGACGCCAAGGCCATCCTGTCTGCCAGACTGAGCAAGAGCAGACGGCTGGAAAATCTG
ATCGCCCAGCTGCCCGGCGAGAAGAAGAATGGCCTGTTCGGAAACCTGATTGCCCTGAGCCTGGGC
CTGACCCCCAACTTCAAGAGCAACTTCGACCTGGCCGAGGATGCCAAACTGCAGCTGAGCAAGGAC
ACCTACGACGACGACCTGGACAACCTGCTGGCCCAGATCGGCGACCAGTACGCCGACCTGTTTCTG
GCCGCCAAGAACCTGTCCGACGCCATCCTGCTGAGCGACATCCTGAGAGTGAACACCGAGATCACC
AAGGCCCCCCTGAGCGCCTCTATGATCAAGAGATACGACGAGCACCACCAGGACCTGACCCTGCTG
AAAGCTCTCGTGCGGCAGCAGCTGCCTGAGAAGTACAAAGAGATTTTCTTCGACCAGAGCAAGAACG
GCTACGCCGGCTACATTGACGGCGGAGCCAGCCAGGAAGAGTTCTACAAGTTCATCAAGCCCATCCT
GGAAAAGATGGACGGCACCGAGGAACTGCTCGTGAAGCTGAACAGAGAGGACCTGCTGCGGAAGC
AGCGGACCTTCGACAACGGCAGCATCCCCCACCAGATCCACCTGGGAGAGCTGCACGCCATTCTGC
GGCGGCAGGAAGATTTTTACCCATTCCTGAAGGACAACCGGGAAAAGATCGAGAAGATCCTGACCTT
CCGCATCCCCTACTACGTGGGCCCTCTGGCCAGGGGAAACAGCAGATTCGCCTGGATGACCAGAAA
GAGCGAGGAAACCATCACCCCCTGGAACTTCGAGGAAGTGGTGGACAAGGGCGCTTCCGCCCAGA
GCTTCATCGAGCGGATGACCAACTTCGATAAGAACCTGCCCAACGAGAAGGTGCTGCCCAAGCACA
GCCTGCTGTACGAGTACTTCACCGTGTATAACGAGCTGACCAAAGTGAAATACGTGACCGAGGGAAT
GAGAAAGCCCGCCTTCCTGAGCGGCGAGCAGAAAAAGGCCATCGTGGACCTGCTGTTCAAGACCAA
CCGGAAAGTGACCGTGAAGCAGCTGAAAGAGGACTACTTCAAGAAAATCGAGTGCTTCGACTCCGTG
GAAATCTCCGGCGTGGAAGATCGGTTCAACGCCTCCCTGGGCACATACCACGATCTGCTGAAAATTAT
CAAGGACAAGGACTTCCTGGACAATGAGGAAAACGAGGACATTCTGGAAGATATCGTGCTGACCCTG
ACACTGTTTGAGGACAGAGAGATGATCGAGGAACGGCTGAAAACCTATGCCCACCTGTTCGACGACA
AAGTGATGAAGCAGCTGAAGCGGCGGAGATACACCGGCTGGGGCAGGCTGAGCCGGAAGCTGATC
AACGGCATCCGGGACAAGCAGTCCGGCAAGACAATCCTGGATTTCCTGAAGTCCGACGGCTTCGCC
AACAGAAACTTCATGCAGCTGATCCACGACGACAGCCTGACCTTTAAAGAGGACATCCAGAAAGCCC

AGGTGTCCGGCCAGGGCGATAGCCTGCACGAGCACATTGCCAATCTGGCCGGCAGCCCCGCCATTA
AGAAGGGCATCCTGCAGACAGTGAAGGTGGTGGACGAGCTCGTGAAAGTGATGGGCCGGCACAAG
CCCGAGAACATCGTGATCGAAATGGCCAGAGAGAACCAGACCACCCAGAAGGGACAGAAGAACAGC
CGCGAGAGAATGAAGCGGATCGAAGAGGGCATCAAAGAGCTGGGCAGCCAGATCCTGAAAGAACAC
CCCGTGGAAAACACCCAGCTGCAGAACGAGAAGCTGTACCTGTACTACCTGCAGAATGGGCGGGATA
TGTACGTGGACCAGGAACTGGACATCAACCGGCTGTCCGACTACGATGTGGACCATATCGTGCCTCA
GAGCTTTCTGAAGGACGACTCCATCGACAACAAGGTGCTGACCAGAAGCGACAAGAACCGGGGCAA
GAGCGACAACGTGCCCTCCGAAGAGGTCGTGAAGAAGATGAAGAACTACTGGCGGCAGCTGCTGAA
CGCCAAGCTGATTACCCAGAGAAAGTTCGACAATCTGACCAAGGCCGAGAGAGGCGGCCTGAGCGA
ACTGGATAAGGCCGGCTTCATCAAGAGACAGCTGGTGGAAACCCGGCAGATCACAAAGCACGTGGC
ACAGATCCTGGACTCCCGGATGAACACTAAGTACGACGAGAATGACAAGCTGATCCGGGAAGTGAAA
GTGATCACCCTGAAGTCCAAGCTGGTGTCCGATTTCCGGAAGGATTTCCAGTTTTACAAAGTGCGCG
AGATCAACAACTACCACCACGCCCACGACGCCTACCTGAACGCCGTCGTGGGAACCGCCCTGATCA
AAAAGTACCCTAAGCTGGAAAGCGAGTTCGTGTACGGCGACTACAAGGTGTACGACGTGCGGAAGAT
GATCGCCAAGAGCGAGCAGGAAATCGGCAAGGCTACCGCCAAGTACTTCTTCTACAGCAACATCATG
AACTTTTTCAAGACCGAGATTACCCTGGCCAACGGCGAGATCCGGAAGCGGCCTCTGATCGAGACAA
ACGGCGAAACCGGGGAGATCGTGTGGGATAAGGGCCGGGATTTTGCCACCGTGCGGAAAGTGCTG
AGCATGCCCCAAGTGAATATCGTGAAAAAGACCGAGGTGCAGACAGGCGGCTTCAGCAAAGAGTCTA
TCCTGCCCAAGAGGAACAGCGATAAGCTGATCGCCAGAAAGAAGGACTGGGACCCTAAGAAGTACG
GCGGCTTCGACAGCCCCACCGTGGCCTATTCTGTGCTGGTGGTGGCCAAAGTGGAAAAGGGCAAGT
CCAAGAAACTGAAGAGTGTGAAAGAGCTGCTGGGGATCACCATCATGGAAAGAAGCAGCTTCGAGAA
GAATCCCATCGACTTTCTGGAAGCCAAGGGCTACAAAGAAGTGAAAAAGGACCTGATCATCAAGCTG
CCTAAGTACTCCCTGTTCGAGCTGGAAAACGGCCGGAAGAGAATGCTGGCCTCTGCCGGCGAACTG
CAGAAGGGAAACGAACTGGCCCTGCCCTCCAAATATGTGAACTTCCTGTACCTGGCCAGCCACTATG
AGAAGCTGAAGGGCTCCCCCGAGGATAATGAGCAGAAACAGCTGTTTGTGGAACAGCACAAGCACTA
CCTGGACGAGATCATCGAGCAGATCAGCGAGTTCTCCAAGAGAGTGATCCTGGCCGACGCTAATCTG
GACAAAGTGCTGTCCGCCTACAACAAGCACCGGGATAAGCCCATCAGAGAGCAGGCCGAGAATATCA
TCCACCTGTTTACCCTGACCAATCTGGGAGCCCCTGCCGCCTTCAAGTACTTTGACACCACCATCGA
CCGGAAGAGGTACACCAGCACCAAAGAGGTGCTGGACGCCACCCTGATCCACCAGAGCATCACCGG
CCTGTACGAGACACGGATCGACCTGTCTCAGCTGGGAGGTGACTCTGGCGGCTCAAAAAGAACCGC
CGACGGCAGCGAATTCGAGCCCAAGAAGAAGAGGAAAGTCTAATTAATTAAGCTGCCTTCTGCGGGG
CTTGCCTTCTGGCCATGCCCTTCTTCTCTCCCTTGCACCTGTACCTCTTGGTCTTTGAATAAAGCCTG
AGTAGGAAGCGACTGTGCCTTCTAGTTGCCAGCCATCTGTTGTTTGCCCCTCCCCCGTGCCTTCCTT
GACCCTGGAAGGTGCCACTCCCACTGTCCTTTCCTAATAAAATGAGAAATTGCATCGCATTGTCTGA
GTAGGTGTCATTCTATTCTGGGGGGTGGGGTGGGGCAGGACAGCAAGGGGGAGGATTGGGAAGAC
AATAGCAGGCATGCTGGGGATGCGGTGGGCTCTATGG

Start	End	Feature Description
1	380	CMV enhancer
381	619	CMV promoter
620	636	T7 promoter with mismatch
637	683	5' UTR
684	740	BP NLS
741	1238	Engineered TadA* variant 8e
1239	1334	(SGGS)2 – XTEN – (SGGS)2 linker
1335	5435	Cas9(D10A) SpCas9 wild-type
5436	5501	Linker + SV40 BP NLS

5502	5605	3' UTR
5606	5830	bGH poly(A) signal

Amino acid sequence

MKRTADGSEFESPKKKRKVSEVEFSHEYWMRHALTLAKRARDEREVPVGAVLVLNNRVIGEGWNRAIGL
HDPTAHAEIMALRQGGLVMQNYRLIDATLYVTFEPCVMCAGAMIHSRIGRVVFGVRNSKRGAAGSLMNVL
NYPGMNHRVEITEGILADECAALLCDFYRMPRQVFNAQKKAQSSINSGGSSGGSSGSETPGTSESATPE
SSGGSSGGSDKKYSIGLAIGTNSVGWAVITDEYKVPSKKFKVLGNTDRHSIKKNLIGALLFDSGETAEATRL
KRTARRRYTRRKNRICYLQEIFSNEMAKVDDSFFHRLEESFLVEEDKKHERHPIFGNIVDEVAYHEKYPTIY
HLRKKLVDSTDKADLRLIYLALAHMIKFRGHFLIEGDLNPDNSDVDKLFIQLVQTYNQLFEENPINASGVDA
KAILSARLSKSRRLENLIAQLPGEKKNGLFGNLIALSLGLTPNFKSNFDLAEDAKLQLSKDTYDDDLDNLLA
QIGDQYADLFLAAKNLSDAILLSDILRVNTEITKAPLSASMIKRYDEHHQDLTLLKALVRQQLPEKYKEIFFD
QSKNGYAGYIDGGASQEEFYKFIKPILEKMDGTEELLVKLNREDLLRKQRTFDNGSIPHQIHLGELHAILRR
QEDFYPFLKDNREKIEKILTFRIPYYVGPLARGNSRFAWMTRKSEETITPWNFEEVVDKGASAQSFIERMT
NFDKNLPNEKVLPKHSLLYEYFTVYNELTKVKYVTEGMRKPAFLSGEQKKAIVDLLFKTNRKVTVKQLKED
YFKKIECFDSVEISGVEDRFNASLGTYHDLLKIIKDKDFLDNEENEDILEDIVLTLTLFEDREMIEERLKTYAH
LFDDKVMKQLKRRRYTGWGRLSRKLINGIRDKQSGKTILDFLKSDGFANRNFMQLIHDDSLTFKEDIQKAQ
VSGQGDSLHEHIANLAGSPAIKKGILQTVKVVDELVKVMGRHKPENIVIEMARENQTTQKGQKNSRERMK
RIEEGIKELGSQILKEHPVENTQLQNEKLYLYYLQNGRDMYVDQELDINRLSDYDVDHIVPQSFLKDDSIDN
KVLTRSDKNRGKSDNVPSEEVVKKMKNYWRQLLNAKLITQRKFDNLTKAERGGLSELDKAGFIKRQLVET
RQITKHVAQILDSRMNTKYDENDKLIREVKVITLKSKLVSDFRKDFQFYKVREINNYHHAHDAYLNAVVGTA
LIKKYPKLESEFVYGDYKVYDVRKMIAKSEQEIGKATAKYFFYSNIMNFFKTEITLANGEIRKRPLIETNGET
GEIVWDKGRDFATVRKVLSMPQVNIVKKTEVQTGGFSKESILPKRNSDKLIARKKDWDPKKYGGFDSPTV
AYSVLVVAKVEKGKSKKLKSVKELLGITIMERSSFEKNPIDFLEAKGYKEVKKDLIIKLPKYSLFELENGRKR
MLASAGELQKGNELALPSKYVNFLYLASHYEKLKGSPEDNEQKQLFVEQHKHYLDEIIEQISEFSKRVILAD
ANLDKVLSAYNKHRDKPIREQAENIIHLFTLTNLGAPAAFKYFDTTIDRKRYTSTKEVLDATLIHQSITGLYET
RIDLSQLGGDSGGSKRTADGSEFEPKKKRKV

Start	End	Feature Description
1	19	BP NLS
20	185	Engineered TadA* variant 8e
186	217	(SGGS)2 – XTEN – (SGGS)2 linker
218	1584	Cas9(D10A) SpCas9 wild-type
1585	1605	Linker + SV40 BP NLS

ABE8e(V106W) – SpCas9 (D10A)
Plasmid ID: pHS0229

DNA sequence

GACATTGATTATTGACTAGTTATTAATAGTAATCAATTACGGGGTCATTAGTTCATAGCCCATATATGGAG
TTCCGCGTTACATAACTTACGGTAAATGGCCCGCCTGGCTGACCGCCCAACGACCCCCGCCCATTGA
CGTCAATAATGACGTATGTTCCCATAGTAACGCCAATAGGGACTTTCCATTGACGTCAATGGGTGGAGT
ATTTACGGTAAACTGCCCACTTGGCAGTACATCAAGTGTATCATATGCCAAGTACGCCCCCTATTGACG
TCAATGACGGTAAATGGCCCGCCTGGCATTATGCCCAGTACATGACCTTATGGGACTTTCCTACTTGG
CAGTACATCTACGTATTAGTCATCGCTATTACCATGGTGATGCGGTTTTGGCAGTACATCAATGGGCGT
GGATAGCGGTTTGACTCACGGGGATTTCCAAGTCTCCACCCCATTGACGTCAATGGGAGTTTGTTTTG
GCACCAAAATCAACGGGACTTTCCAAAATGTCGTAACAACTCCGCCCCATTGACGCAAATGGGCGGT
AGGCGTGTACGGTGGGAGGTCTATATAAGCAGAGCTGGTTTAGTGAACCGTCAGATCTCGAGCTCGG
TACCTAATACGACACACTATAAGGAAATAAGAGAGAAAAGAAGAGTAAGAAGAAATATAAGAGCCACCA
TGAAACGGACAGCCGACGGAAGCGAGTTCGAGTCACCAAAGAAGAAGCGGAAAGTCTCTGAGGTG
GAGTTTTCCCACGAGTACTGGATGAGACATGCCCTGACCCTGGCCAAGAGGGCACGGGATGAGAGG
GAGGTGCCTGTGGGAGCCGTGCTGGTGCTGAACAATAGAGTGATCGGCGAGGGCTGGAACAGAGC
CATCGGCCTGCACGACCCAACAGCCCATGCCGAAATTATGGCCCTGAGACAGGGCGGCCTGGTCAT
GCAGAACTACAGACTGATTGACGCCACCCTGTACGTGACATTCGAGCCTTGCGTGATGTGCGCCGGC
GCCATGATCCACTCTAGGATCGGCCGCGTGGTGTTTGGATGGAGAAATTCTAAAAGAGGCGCCGCAG
GCTCCCTGATGAACGTGCTGAACTACCCCGGCATGAATCACCGCGTCGAAATTACCGAGGGAATCCT
GGCAGATGAATGTGCCGCCCTGCTGTGCGATTTCTATCGGATGCCTAGACAGGTGTTCAATGCTCAG
AAGAAGGCCCAGAGCTCCATCAACTCCGGAGGATCTAGCGGAGGCTCCTCTGGCTCTGAGACACCT
GGCACAAGCGAGAGCGCAACACCTGAAAGCAGCGGGGGCAGCAGCGGGGGGTCAGACAAGAAGT
ACAGCATCGGCCTGGCCATCGGCACCAACTCTGTGGGCTGGGCCGTGATCACCGACGAGTACAAGG
TGCCCAGCAAGAAATTCAAGGTGCTGGGCAACACCGACCGGCACAGCATCAAGAAGAACCTGATCG
GAGCCCTGCTGTTCGACAGCGGCGAAACAGCCGAGGCCACCCGGCTGAAGAGAACCGCCAGAAGA
AGATACACCAGACGGAAGAACCGGATCTGCTATCTGCAAGAGATCTTCAGCAACGAGATGGCCAAGG
TGGACGACAGCTTCTTCCACAGACTGGAAGAGTCCTTCCTGGTGGAAGAGGATAAGAAGCACGAGC
GGCACCCCATCTTCGGCAACATCGTGGACGAGGTGGCCTACCACGAGAAGTACCCCACCATCTACCA
CCTGAGAAAGAAACTGGTGGACAGCACCGACAAGGCCGACCTGCGGCTGATCTATCTGGCCCTGGC
CCACATGATCAAGTTCCGGGGCCACTTCCTGATCGAGGGCGACCTGAACCCCGACAACAGCGACGT
GGACAAGCTGTTCATCCAGCTGGTGCAGACCTACAACCAGCTGTTCGAGGAAAACCCCATCAACGCC
AGCGGCGTGGACGCCAAGGCCATCCTGTCTGCCAGACTGAGCAAGAGCAGACGGCTGGAAAATCTG
ATCGCCCAGCTGCCCGGCGAGAAGAAGAATGGCCTGTTCGGAAACCTGATTGCCCTGAGCCTGGGC
CTGACCCCCAACTTCAAGAGCAACTTCGACCTGGCCGAGGATGCCAAACTGCAGCTGAGCAAGGAC
ACCTACGACGACGACCTGGACAACCTGCTGGCCCAGATCGGCGACCAGTACGCCGACCTGTTTCTG
GCCGCCAAGAACCTGTCCGACGCCATCCTGCTGAGCGACATCCTGAGAGTGAACACCGAGATCACC
AAGGCCCCCCTGAGCGCCTCTATGATCAAGAGATACGACGAGCACCACCAGGACCTGACCCTGCTG
AAAGCTCTCGTGCGGCAGCAGCTGCCTGAGAAGTACAAAGAGATTTTCTTCGACCAGAGCAAGAACG
GCTACGCCGGCTACATTGACGGCGGAGCCAGCCAGGAAGAGTTCTACAAGTTCATCAAGCCCATCCT
GGAAAAGATGGACGGCACCGAGGAACTGCTCGTGAAGCTGAACAGAGAGGACCTGCTGCGGAAGC
AGCGGACCTTCGACAACGGCAGCATCCCCCACCAGATCCACCTGGGAGAGCTGCACGCCATTCTGC
GGCGGCAGGAAGATTTTTACCCATTCCTGAAGGACAACCGGGAAAAGATCGAGAAGATCCTGACCTT
CCGCATCCCCTACTACGTGGGCCCTCTGGCCAGGGGAAACAGCAGATTCGCCTGGATGACCAGAAA
GAGCGAGGAAACCATCACCCCCTGGAACTTCGAGGAAGTGGTGGACAAGGGCGCTTCCGCCCAGA
GCTTCATCGAGCGGATGACCAACTTCGATAAGAACCTGCCCAACGAGAAGGTGCTGCCCAAGCACA
GCCTGCTGTACGAGTACTTCACCGTGTATAACGAGCTGACCAAAGTGAAATACGTGACCGAGGGAAT
GAGAAAGCCCGCCTTCCTGAGCGGCGAGCAGAAAAAGGCCATCGTGGACCTGCTGTTCAAGACCAA
CCGGAAAGTGACCGTGAAGCAGCTGAAAGAGGACTACTTCAAGAAAATCGAGTGCTTCGACTCCGTG
GAAATCTCCGGCGTGGAAGATCGGTTCAACGCCTCCCTGGGCACATACCACGATCTGCTGAAAATTAT
CAAGGACAAGGACTTCCTGGACAATGAGGAAAACGAGGACATTCTGGAAGATATCGTGCTGACCCTG
ACACTGTTTGAGGACAGAGAGATGATCGAGGAACGGCTGAAAACCTATGCCCACCTGTTCGACGACA
AAGTGATGAAGCAGCTGAAGCGGCGGAGATACACCGGCTGGGGCAGGCTGAGCCGGAAGCTGATC
AACGGCATCCGGGACAAGCAGTCCGGCAAGACAATCCTGGATTTCCTGAAGTCCGACGGCTTCGCC
AACAGAAACTTCATGCAGCTGATCCACGACGACAGCCTGACCTTTAAAGAGGACATCCAGAAAGCCC

AGGTGTCCGGCCAGGGCGATAGCCTGCACGAGCACATTGCCAATCTGGCCGGCAGCCCCGCCATTA
AGAAGGGCATCCTGCAGACAGTGAAGGTGGTGGACGAGCTCGTGAAAGTGATGGGCCGGCACAAG
CCCGAGAACATCGTGATCGAAATGGCCAGAGAGAACCAGACCACCCAGAAGGGACAGAAGAACAGC
CGCGAGAGAATGAAGCGGATCGAAGAGGGCATCAAAGAGCTGGGCAGCCAGATCCTGAAAGAACAC
CCCGTGGAAAACACCCAGCTGCAGAACGAGAAGCTGTACCTGTACTACCTGCAGAATGGGCGGGATA
TGTACGTGGACCAGGAACTGGACATCAACCGGCTGTCCGACTACGATGTGGACCATATCGTGCCTCA
GAGCTTTCTGAAGGACGACTCCATCGACAACAAGGTGCTGACCAGAAGCGACAAGAACCGGGGCAA
GAGCGACAACGTGCCCTCCGAAGAGGTCGTGAAGAAGATGAAGAACTACTGGCGGCAGCTGCTGAA
CGCCAAGCTGATTACCCAGAGAAAGTTCGACAATCTGACCAAGGCCGAGAGAGGCGGCCTGAGCGA
ACTGGATAAGGCCGGCTTCATCAAGAGACAGCTGGTGGAAACCCGGCAGATCACAAAGCACGTGGC
ACAGATCCTGGACTCCCGGATGAACACTAAGTACGACGAGAATGACAAGCTGATCCGGGAAGTGAAA
GTGATCACCCTGAAGTCCAAGCTGGTGTCCGATTTCCGGAAGGATTTCCAGTTTTACAAAGTGCGCG
AGATCAACAACTACCACCACGCCCACGACGCCTACCTGAACGCCGTCGTGGGAACCGCCCTGATCA
AAAAGTACCCTAAGCTGGAAAGCGAGTTCGTGTACGGCGACTACAAGGTGTACGACGTGCGGAAGAT
GATCGCCAAGAGCGAGCAGGAAATCGGCAAGGCTACCGCCAAGTACTTCTTCTACAGCAACATCATG
AACTTTTTCAAGACCGAGATTACCCTGGCCAACGGCGAGATCCGGAAGCGGCCTCTGATCGAGACAA
ACGGCGAAACCGGGGAGATCGTGTGGGATAAGGGCCGGGATTTTGCCACCGTGCGGAAAGTGCTG
AGCATGCCCCAAGTGAATATCGTGAAAAAGACCGAGGTGCAGACAGGCGGCTTCAGCAAAGAGTCTA
TCCTGCCCAAGAGGAACAGCGATAAGCTGATCGCCAGAAAGAAGGACTGGGACCCTAAGAAGTACG
GCGGCTTCGACAGCCCCACCGTGGCCTATTCTGTGCTGGTGGTGGCCAAAGTGGAAAAGGGCAAGT
CCAAGAAACTGAAGAGTGTGAAAGAGCTGCTGGGGATCACCATCATGGAAAGAAGCAGCTTCGAGAA
GAATCCCATCGACTTTCTGGAAGCCAAGGGCTACAAAGAAGTGAAAAAGGACCTGATCATCAAGCTG
CCTAAGTACTCCCTGTTCGAGCTGGAAAACGGCCGGAAGAGAATGCTGGCCTCTGCCGGCGAACTG
CAGAAGGGAAACGAACTGGCCCTGCCCTCCAAATATGTGAACTTCCTGTACCTGGCCAGCCACTATG
AGAAGCTGAAGGGCTCCCCCGAGGATAATGAGCAGAAACAGCTGTTTGTGGAACAGCACAAGCACTA
CCTGGACGAGATCATCGAGCAGATCAGCGAGTTCTCCAAGAGAGTGATCCTGGCCGACGCTAATCTG
GACAAAGTGCTGTCCGCCTACAACAAGCACCGGGATAAGCCCATCAGAGAGCAGGCCGAGAATATCA
TCCACCTGTTTACCCTGACCAATCTGGGAGCCCCTGCCGCCTTCAAGTACTTTGACACCACCATCGA
CCGGAAGAGGTACACCAGCACCAAAGAGGTGCTGGACGCCACCCTGATCCACCAGAGCATCACCGG
CCTGTACGAGACACGGATCGACCTGTCTCAGCTGGGAGGTGACTCTGGCGGCTCAAAAAGAACCGC
CGACGGCAGCGAATTCGAGCCCAAGAAGAAGAGGAAAGTCTAATTAATTAAGCTGCCTTCTGCGGGG
CTTGCCTTCTGGCCATGCCCTTCTTCTCTCCCTTGCACCTGTACCTCTTGGTCTTTGAATAAAGCCTG
AGTAGGAAGCGACTGTGCCTTCTAGTTGCCAGCCATCTGTTGTTTGCCCCTCCCCCGTGCCTTCCTT
GACCCTGGAAGGTGCCACTCCCACTGTCCTTTCCTAATAAAATGAGAAATTGCATCGCATTGTCTGA
GTAGGTGTCATTCTATTCTGGGGGGTGGGGTGGGGCAGGACAGCAAGGGGGAGGATTGGGAAGAC
AATAGCAGGCATGCTGGGGATGCGGTGGGCTCTATGG

Start	End	Feature Description
1	380	CMV enhancer
381	619	CMV promoter
620	636	T7 promoter with mismatch
637	683	5' UTR
684	740	BP NLS
741	1238	Engineered TadA* variant 8e(V106W)
1239	1334	(SGGS)2 – XTEN – (SGGS)2 linker
1335	5435	Cas9(D10A) SpCas9 wild-type
5436	5501	Linker + SV40 BP NLS

5502	5605	3' UTR
5606	5830	bGH poly(A) signal

Amino acid sequence
MKRTADGSEFESPKKKRKVSEVEFSHEYWMRHALTLAKRARDEREVPVGAVLVLNNRVIGEGWNRAIGL
HDPTAHAEIMALRQGGLVMQNYRLIDATLYVTFEPCVMCAGAMIHSRIGRVVFGWRNSKRGAAGSLMNV
LNYPGMNHRVEITEGILADECAALLCDFYRMPRQVFNAQKKAQSSINSGGSSGGSSGSETPGTSESATP
ESSGGSSGGSDKKYSIGLAIGTNSVGWAVITDEYKVPSKKFKVLGNTDRHSIKKNLIGALLFDSGETAEAT
RLKRTARRRYTRRKNRICYLQEIFSNEMAKVDDSFFHRLEESFLVEEDKKHERHPIFGNIVDEVAYHEKYP
TIYHLRKKLVDSTDKADLRLIYLALAHMIKFRGHFLIEGDLNPDNSDVDKLFIQLVQTYNQLFEENPINASGV
DAKAILSARLSKSRRLENLIAQLPGEKKNGLFGNLIALSLGLTPNFKSNFDLAEDAKLQLSKDTYDDDLDNL
LAQIGDQYADLFLAAKNLSDAILLSDILRVNTEITKAPLSASMIKRYDEHHQDLTLLKALVRQQLPEKYKEIFF
DQSKNGYAGYIDGGASQEEFYKFIKPILEKMDGTEELLVKLNREDLLRKQRTFDNGSIPHQIHLGELHAILR
RQEDFYPFLKDNREKIEKILTFRIPYYVGPLARGNSRFAWMTRKSEETITPWNFEEVVDKGASAQSFIERM
TNFDKNLPNEKVLPKHSLLYEYFTVYNELTKVKYVTEGMRKPAFLSGEQKKAIVDLLFKTNRKVTVKQLKE
DYFKKIECFDSVEISGVEDRFNASLGTYHDLLKIIKDKDFLDNEENEDILEDIVLTLTLFEDREMIEERLKTYA
HLFDDKVMKQLKRRRYTGWGRLSRKLINGIRDKQSGKTILDFLKSDGFANRNFMQLIHDDSLTFKEDIQKA
QVSGQGDSLHEHIANLAGSPAIKKGILQTVKVVDELVKVMGRHKPENIVIEMARENQTTQKGQKNSRERM
KRIEEGIKELGSQILKEHPVENTQLQNEKLYLYYLQNGRDMYVDQELDINRLSDYDVDHIVPQSFLKDDSID
NKVLTRSDKNRGKSDNVPSEEVVKKMKNYWRQLLNAKLITQRKFDNLTKAERGGLSELDKAGFIKRQLVE
TRQITKHVAQILDSRMNTKYDENDKLIREVKVITLKSKLVSDFRKDFQFYKVREINNYHHAHDAYLNAVVGT
ALIKKYPKLESEFVYGDYKVYDVRKMIAKSEQEIGKATAKYFFYSNIMNFFKTEITLANGEIRKRPLIETNGE
TGEIVWDKGRDFATVRKVLSMPQVNIVKKTEVQTGGFSKESILPKRNSDKLIARKKDWDPKKYGGFDSPT
VAYSVLVVAKVEKGKSKKLKSVKELLGITIMERSSFEKNPIDFLEAKGYKEVKKDLIIKLPKYSLFELENGRK
RMLASAGELQKGNELALPSKYVNFLYLASHYEKLKGSPEDNEQKQLFVEQHKHYLDEIIEQISEFSKRVILA
DANLDKVLSAYNKHRDKPIREQAENIIHLFTLTNLGAPAAFKYFDTTIDRKRYTSTKEVLDATLIHQSITGLYE
TRIDLSQLGGDSGGSKRTADGSEFEPKKKRKV

Start	End	Feature Description
1	19	BP NLS
20	185	Engineered TadA* variant 8e
186	217	(SGGS)2 – XTEN – (SGGS)2 linker
218	1584	Cas9(D10A) SpCas9 wild-type
1585	1605	Linker + SV40 BP NLS

ABE9 – SpCas9 (D10A)
Plasmid ID: pHS0413

DNA sequence
GACATTGATTATTGACTAGTTATTAATAGTAATCAATTACGGGGTCATTAGTTCATAGCCCATATATGGAG
TTCCGCGTTACATAACTTACGGTAAATGGCCCGCCTGGCTGACCGCCCAACGACCCCCGCCCATTGA
CGTCAATAATGACGTATGTTCCCATAGTAACGCCAATAGGGACTTTCCATTGACGTCAATGGGTGGAGT
ATTTACGGTAAACTGCCCACTTGGCAGTACATCAAGTGTATCATATGCCAAGTACGCCCCCTATTGACG
TCAATGACGGTAAATGGCCCGCCTGGCATTATGCCCAGTACATGACCTTATGGGACTTTCCTACTTGG
CAGTACATCTACGTATTAGTCATCGCTATTACCATGGTGATGCGGTTTTGGCAGTACATCAATGGGCGT
GGATAGCGGTTTGACTCACGGGGATTTCCAAGTCTCCACCCCATTGACGTCAATGGGAGTTTGTTTTG
GCACCAAAATCAACGGGACTTTCCAAAATGTCGTAACAACTCCGCCCCATTGACGCAAATGGGCGGT
AGGCGTGTACGGTGGGAGGTCTATATAAGCAGAGCTGGTTTAGTGAACCGTCAGATCTCGAGCTCGG
TACCTAATACGACACACTATAAGGAAATAAGAGAGAAAAGAAGAGTAAGAAGAAATATAAGAGCCACCA
TGAAACGGACAGCCGACGGAAGCGAGTTCGAGTCACCAAAGAAGAAGCGGAAAGTCTCTGAGGTG
GAGTTTTCCCACGAGTACTGGATGAGACATGCCCTGACCCTGGCCAAGAGGGCACGGGATGAGAGG
GAGGTGCCTGTGGGAGCCGTGCTGGTGCTGAACAATAGAGTGATCGGCGAGGGCTGGAACAGAGC
CATCGGCCTGCACGACCCAACAGCCCATGCCGAAATTATGGCCCTGAGACAGGGCGGCCTGGTCAT
GCAGAACTACAGACTGATTGACGCCACCCTGTACGTGACATTCGAGCCTTGCGTGATGTGCGCCGGC
GCCATGATCCACTCTAGGATCGGCCGCGTGGTGTTTGGCGTGAGGCAGTCAAAAAGAGGCGCCGCA
GGCTCCCTGATGAACGTGCTGAACTACCCCGGCATGAATCACCGCGTCGAAATTACCGAGGGAATCC
TGGCAGATGAATGTGCCGCCCTGACCTGCGATTTCTATCGGATGCCTAGACAGGTGTTCAATGCTCAG
AAGAAGGCCCAGAGCTCCATCAACTCCGGAGGATCTAGCGGAGGCTCCTCTGGCTCTGAGACACCT
GGCACAAGCGAGAGCGCAACACCTGAAAGCAGCGGGGGCAGCAGCGGGGGGTCAGACAAGAAGT
ACAGCATCGGCCTGGCCATCGGCACCAACTCTGTGGGCTGGGCCGTGATCACCGACGAGTACAAGG
TGCCCAGCAAGAAATTCAAGGTGCTGGGCAACACCGACCGGCACAGCATCAAGAAGAACCTGATCG
GAGCCCTGCTGTTCGACAGCGGCGAAACAGCCGAGGCCACCCGGCTGAAGAGAACCGCCAGAAGA
AGATACACCAGACGGAAGAACCGGATCTGCTATCTGCAAGAGATCTTCAGCAACGAGATGGCCAAGG
TGGACGACAGCTTCTTCCACAGACTGGAAGAGTCCTTCCTGGTGGAAGAGGATAAGAAGCACGAGC
GGCACCCCATCTTCGGCAACATCGTGGACGAGGTGGCCTACCACGAGAAGTACCCCACCATCTACCA
CCTGAGAAAGAAACTGGTGGACAGCACCGACAAGGCCGACCTGCGGCTGATCTATCTGGCCCTGGC
CCACATGATCAAGTTCCGGGGCCACTTCCTGATCGAGGGCGACCTGAACCCCGACAACAGCGACGT
GGACAAGCTGTTCATCCAGCTGGTGCAGACCTACAACCAGCTGTTCGAGGAAAACCCCATCAACGCC
AGCGGCGTGGACGCCAAGGCCATCCTGTCTGCCAGACTGAGCAAGAGCAGACGGCTGGAAAATCTG
ATCGCCCAGCTGCCCGGCGAGAAGAAGAATGGCCTGTTCGGAAACCTGATTGCCCTGAGCCTGGGC
CTGACCCCCAACTTCAAGAGCAACTTCGACCTGGCCGAGGATGCCAAACTGCAGCTGAGCAAGGAC
ACCTACGACGACGACCTGGACAACCTGCTGGCCCAGATCGGCGACCAGTACGCCGACCTGTTTCTG
GCCGCCAAGAACCTGTCCGACGCCATCCTGCTGAGCGACATCCTGAGAGTGAACACCGAGATCACC
AAGGCCCCCCTGAGCGCCTCTATGATCAAGAGATACGACGAGCACCACCAGGACCTGACCCTGCTG
AAAGCTCTCGTGCGGCAGCAGCTGCCTGAGAAGTACAAAGAGATTTTCTTCGACCAGAGCAAGAACG
GCTACGCCGGCTACATTGACGGCGGAGCCAGCCAGGAAGAGTTCTACAAGTTCATCAAGCCCATCCT
GGAAAAGATGGACGGCACCGAGGAACTGCTCGTGAAGCTGAACAGAGAGGACCTGCTGCGGAAGC
AGCGGACCTTCGACAACGGCAGCATCCCCCACCAGATCCACCTGGGAGAGCTGCACGCCATTCTGC
GGCGGCAGGAAGATTTTTACCCATTCCTGAAGGACAACCGGGAAAAGATCGAGAAGATCCTGACCTT
CCGCATCCCCTACTACGTGGGCCCTCTGGCCAGGGGAAACAGCAGATTCGCCTGGATGACCAGAAA
GAGCGAGGAAACCATCACCCCCTGGAACTTCGAGGAAGTGGTGGACAAGGGCGCTTCCGCCCAGA
GCTTCATCGAGCGGATGACCAACTTCGATAAGAACCTGCCCAACGAGAAGGTGCTGCCCAAGCACA
GCCTGCTGTACGAGTACTTCACCGTGTATAACGAGCTGACCAAAGTGAAATACGTGACCGAGGGAAT
GAGAAAGCCCGCCTTCCTGAGCGGCGAGCAGAAAAAGGCCATCGTGGACCTGCTGTTCAAGACCAA
CCGGAAAGTGACCGTGAAGCAGCTGAAAGAGGACTACTTCAAGAAAATCGAGTGCTTCGACTCCGTG
GAAATCTCCGGCGTGGAAGATCGGTTCAACGCCTCCCTGGGCACATACCACGATCTGCTGAAAATTAT
CAAGGACAAGGACTTCCTGGACAATGAGGAAAACGAGGACATTCTGGAAGATATCGTGCTGACCCTG
ACACTGTTTGAGGACAGAGAGATGATCGAGGAACGGCTGAAAACCTATGCCCACCTGTTCGACGACA
AAGTGATGAAGCAGCTGAAGCGGCGGAGATACACCGGCTGGGGCAGGCTGAGCCGGAAGCTGATC
AACGGCATCCGGGACAAGCAGTCCGGCAAGACAATCCTGGATTTCCTGAAGTCCGACGGCTTCGCC
AACAGAAACTTCATGCAGCTGATCCACGACGACAGCCTGACCTTTAAAGAGGACATCCAGAAAGCCC

AGGTGTCCGGCCAGGGCGATAGCCTGCACGAGCACATTGCCAATCTGGCCGGCAGCCCCGCCATTA
AGAAGGGCATCCTGCAGACAGTGAAGGTGGTGGACGAGCTCGTGAAAGTGATGGGCCGGCACAAG
CCCGAGAACATCGTGATCGAAATGGCCAGAGAGAACCAGACCACCCAGAAGGGACAGAAGAACAGC
CGCGAGAGAATGAAGCGGATCGAAGAGGGCATCAAAGAGCTGGGCAGCCAGATCCTGAAAGAACAC
CCCGTGGAAAACACCCAGCTGCAGAACGAGAAGCTGTACCTGTACTACCTGCAGAATGGGCGGGATA
TGTACGTGGACCAGGAACTGGACATCAACCGGCTGTCCGACTACGATGTGGACCATATCGTGCCTCA
GAGCTTTCTGAAGGACGACTCCATCGACAACAAGGTGCTGACCAGAAGCGACAAGAACCGGGGCAA
GAGCGACAACGTGCCCTCCGAAGAGGTCGTGAAGAAGATGAAGAACTACTGGCGGCAGCTGCTGAA
CGCCAAGCTGATTACCCAGAGAAAGTTCGACAATCTGACCAAGGCCGAGAGAGGCGGCCTGAGCGA
ACTGGATAAGGCCGGCTTCATCAAGAGACAGCTGGTGGAAACCCGGCAGATCACAAAGCACGTGGC
ACAGATCCTGGACTCCCGGATGAACACTAAGTACGACGAGAATGACAAGCTGATCCGGGAAGTGAAA
GTGATCACCCTGAAGTCCAAGCTGGTGTCCGATTTCCGGAAGGATTTCCAGTTTTACAAAGTGCGCG
AGATCAACAACTACCACCACGCCCACGACGCCTACCTGAACGCCGTCGTGGGAACCGCCCTGATCA
AAAAGTACCCTAAGCTGGAAAGCGAGTTCGTGTACGGCGACTACAAGGTGTACGACGTGCGGAAGAT
GATCGCCAAGAGCGAGCAGGAAATCGGCAAGGCTACCGCCAAGTACTTCTTCTACAGCAACATCATG
AACTTTTTCAAGACCGAGATTACCCTGGCCAACGGCGAGATCCGGAAGCGGCCTCTGATCGAGACAA
ACGGCGAAACCGGGGAGATCGTGTGGGATAAGGGCCGGGATTTTGCCACCGTGCGGAAAGTGCTG
AGCATGCCCCAAGTGAATATCGTGAAAAAGACCGAGGTGCAGACAGGCGGCTTCAGCAAAGAGTCTA
TCCTGCCCAAGAGGAACAGCGATAAGCTGATCGCCAGAAAGAAGGACTGGGACCCTAAGAAGTACG
GCGGCTTCGACAGCCCCACCGTGGCCTATTCTGTGCTGGTGGTGGCCAAAGTGGAAAAGGGCAAGT
CCAAGAAACTGAAGAGTGTGAAAGAGCTGCTGGGGATCACCATCATGGAAAGAAGCAGCTTCGAGAA
GAATCCCATCGACTTTCTGGAAGCCAAGGGCTACAAAGAAGTGAAAAAGGACCTGATCATCAAGCTG
CCTAAGTACTCCCTGTTCGAGCTGGAAAACGGCCGGAAGAGAATGCTGGCCTCTGCCGGCGAACTG
CAGAAGGGAAACGAACTGGCCCTGCCCTCCAAATATGTGAACTTCCTGTACCTGGCCAGCCACTATG
AGAAGCTGAAGGGCTCCCCCGAGGATAATGAGCAGAAACAGCTGTTTGTGGAACAGCACAAGCACTA
CCTGGACGAGATCATCGAGCAGATCAGCGAGTTCTCCAAGAGAGTGATCCTGGCCGACGCTAATCTG
GACAAAGTGCTGTCCGCCTACAACAAGCACCGGGATAAGCCCATCAGAGAGCAGGCCGAGAATATCA
TCCACCTGTTTACCCTGACCAATCTGGGAGCCCCTGCCGCCTTCAAGTACTTTGACACCACCATCGA
CCGGAAGAGGTACACCAGCACCAAAGAGGTGCTGGACGCCACCCTGATCCACCAGAGCATCACCGG
CCTGTACGAGACACGGATCGACCTGTCTCAGCTGGGAGGTGACTCTGGCGGCTCAAAAAGAACCGC
CGACGGCAGCGAATTCGAGCCCAAGAAGAAGAGGAAAGTCTAATTAATTAAGCTGCCTTCTGCGGGG
CTTGCCTTCTGGCCATGCCCTTCTTCTCTCCCTTGCACCTGTACCTCTTGGTCTTTGAATAAAGCCTG
AGTAGGAAGCGACTGTGCCTTCTAGTTGCCAGCCATCTGTTGTTTGCCCCTCCCCCGTGCCTTCCTT
GACCCTGGAAGGTGCCACTCCCACTGTCCTTTCCTAATAAAATGAGAAATTGCATCGCATTGTCTGA
GTAGGTGTCATTCTATTCTGGGGGGTGGGGTGGGGCAGGACAGCAAGGGGGAGGATTGGGAAGAC
AATAGCAGGCATGCTGGGGATGCGGTGGGCTCTATGG

Start	End	Feature Description
1	380	CMV enhancer
381	619	CMV promoter
620	636	T7 promoter with mismatch
637	683	5' UTR
684	740	BP NLS
741	1238	Engineered TadA* variant ABE9 (TadA-8e N108Q L145T)
1239	1334	(SGGS)2 – XTEN – (SGGS)2 linker
1335	5435	Cas9(D10A) SpCas9 wild-type
5436	5501	Linker + SV40 BP NLS

5502	5605	3' UTR
5606	5830	bGH poly(A) signal

Amino acid sequence

MKRTADGSEFESPKKKRKVSEVEFSHEYWMRHALTLAKRARDEREVPVGAVLVLNNRVIGEGWNRAIGL
HDPTAHAEIMALRQGGLVMQNYRLIDATLYVTFEPCVMCAGAMIHSRIGRVVFGVRQSKRGAAGSLMNVL
NYPGMNHRVEITEGILADECAALTCDFYRMPRQVFNAQKKAQSSINSGGSSGGSSGSETPGTSESATPE
SSGGSSGGSDKKYSIGLAIGTNSVGWAVITDEYKVPSKKFKVLGNTDRHSIKKNLIGALLFDSGETAEATRL
KRTARRRYTRRKNRICYLQEIFSNEMAKVDDSFFHRLEESFLVEEDKKHERHPIFGNIVDEVAYHEKYPTIY
HLRKKLVDSTDKADLRLIYLALAHMIKFRGHFLIEGDLNPDNSDVDKLFIQLVQTYNQLFEENPINASGVDA
KAILSARLSKSRRLENLIAQLPGEKKNGLFGNLIALSLGLTPNFKSNFDLAEDAKLQLSKDTYDDDLDNLLA
QIGDQYADLFLAAKNLSDAILLSDILRVNTEITKAPLSASMIKRYDEHHQDLTLLKALVRQQLPEKYKEIFFD
QSKNGYAGYIDGGASQEEFYKFIKPILEKMDGTEELLVKLNREDLLRKQRTFDNGSIPHQIHLGELHAILRR
QEDFYPFLKDNREKIEKILTFRIPYYVGPLARGNSRFAWMTRKSEETITPWNFEEVVDKGASAQSFIERMT
NFDKNLPNEKVLPKHSLLYEYFTVYNELTKVKYVTEGMRKPAFLSGEQKKAIVDLLFKTNRKVTVKQLKED
YFKKIECFDSVEISGVEDRFNASLGTYHDLLKIIKDKDFLDNEENEDILEDIVLTLTLFEDREMIEERLKTYAH
LFDDKVMKQLKRRRYTGWGRLSRKLINGIRDKQSGKTILDFLKSDGFANRNFMQLIHDDSLTFKEDIQKAQ
VSGQGDSLHEHIANLAGSPAIKKGILQTVKVVDELVKVMGRHKPENIVIEMARENQTTQKGQKNSRERMK
RIEEGIKELGSQILKEHPVENTQLQNEKLYLYYLQNGRDMYVDQELDINRLSDYDVDHIVPQSFLKDDSIDN
KVLTRSDKNRGKSDNVPSEEVVKKMKNYWRQLLNAKLITQRKFDNLTKAERGGLSELDKAGFIKRQLVET
RQITKHVAQILDSRMNTKYDENDKLIREVKVITLKSKLVSDFRKDFQFYKVREINNYHHAHDAYLNAVVGTA
LIKKYPKLESEFVYGDYKVYDVRKMIAKSEQEIGKATAKYFFYSNIMNFFKTEITLANGEIRKRPLIETNGET
GEIVWDKGRDFATVRKVLSMPQVNIVKKTEVQTGGFSKESILPKRNSDKLIARKKDWDPKKYGGFDSPTV
AYSVLVVAKVEKGKSKKLKSVKELLGITIMERSSFEKNPIDFLEAKGYKEVKKDLIIKLPKYSLFELENGRKR
MLASAGELQKGNELALPSKYVNFLYLASHYEKLKGSPEDNEQKQLFVEQHKHYLDEIIEQISEFSKRVILAD
ANLDKVLSAYNKHRDKPIREQAENIIHLFTLTNLGAPAAFKYFDTTIDRKRYTSTKEVLDATLIHQSITGLYET
RIDLSQLGGDSGGSKRTADGSEFEPKKKRKV

Start	End	Feature Description
1	19	BP NLS
20	185	Engineered TadA* variant 8e
186	217	(SGGS)2 – XTEN – (SGGS)2 linker
218	1584	Cas9(D10A) SpCas9 wild-type
1585	1605	Linker + SV40 BP NLS

BE4max – Cas9 (D10A) – SpRY variant – UGI
Plasmid ID: pHS0422

DNA sequence

GACATTGATTATTGACTAGTTATTAATAGTAATCAATTACGGGGTCATTAGTTCATAGCCCATATATGGAG
TTCCGCGTTACATAACTTACGGTAAATGGCCCGCCTGGCTGACCGCCCAACGACCCCCGCCCATTGA
CGTCAATAATGACGTATGTTCCCATAGTAACGCCAATAGGGACTTTCCATTGACGTCAATGGGTGGAGT
ATTTACGGTAAACTGCCCACTTGGCAGTACATCAAGTGTATCATATGCCAAGTACGCCCCCTATTGACG
TCAATGACGGTAAATGGCCCGCCTGGCATTATGCCCAGTACATGACCTTATGGGACTTTCCTACTTGG
CAGTACATCTACGTATTAGTCATCGCTATTACCATGGTGATGCGGTTTTGGCAGTACATCAATGGGCGT
GGATAGCGGTTTGACTCACGGGGATTTCCAAGTCTCCACCCCATTGACGTCAATGGGAGTTTGTTTTG
GCACCAAAATCAACGGGACTTTCCAAAATGTCGTAACAACTCCGCCCCATTGACGCAAATGGGCGGT
AGGCGTGTACGGTGGGAGGTCTATATAAGCAGAGCTGGTTTAGTGAACCGTCAGATCTCGAGCTCGG
TACCTAATACGACACACTATAAGGAAATAAGAGAGAAAAGAAGAGTAAGAAGAAATATAAGAGCCACCA
TGAAACGGACAGCCGACGGAAGCGAGTTCGAGTCACCAAAGAAGAAGCGGAAAGTCAGTTCAGAGA
CTGGGCCTGTCGCCGTCGATCCAACCCTGCGCCGCCGGATTGAACCTCACGAGTTTGAAGTGTTCTT
TGACCCCCGGGAGCTGAGAAAGGAGACATGCCTGCTGTACGAGATCAACTGGGGAGGCAGGCACTC
CATCTGGAGGCACACCTCTCAGAACACAAATAAGCACGTGGAGGTGAACTTCATCGAGAAGTTTACC
ACAGAGCGGTACTTCTGCCCCAATACCAGATGTAGCATCACATGGTTTCTGAGCTGGTCCCCTTGCG
GAGAGTGTAGCAGGGCCATCACCGAGTTCCTGTCCAGATATCCACACGTGACACTGTTTATCTACATC
GCCAGGCTGTATCACCACGCAGACCCAAGGAATAGGCAGGGCCTGCGCGATCTGATCAGCTCCGGC
GTGACCATCCAGATCATGACAGAGCAGGAGTCCGGCTACTGCTGGCGGAACTTCGTGAATTATTCTC
CTAGCAACGAGGCCCACTGGCCTAGGTACCCACACCTGTGGGTGCGCCTGTACGTGCTGGAGCTGT
ATTGCATCATCCTGGGCCTGCCCCCTTGTCTGAATATCCTGCGGAGAAAGCAGCCCCAGCTGACCTT
CTTTACAATCGCCCTGCAGTCTTGTCACTATCAGAGGCTGCCACCCCACATCCTGTGGGCCACAGGC
CTGAAGTCTGGCGGATCTAGCGGAGGATCCTCTGGCAGCGAGACACCAGGAACAAGCGAGTCAGCA
ACACCAGAGAGCAGTGGCGGCAGCAGCGGCGGCAGCGACAAGAAGTACAGCATCGGCCTGGCCAT
CGGCACCAACTCTGTGGGCTGGGCCGTGATCACCGACGAGTACAAGGTGCCCAGCAAGAAATTCAA
GGTGCTGGGCAACACCGACCGGCACAGCATCAAGAAGAACCTGATCGGAGCCCTGCTGTTCGACAG
CGGCGAAACAGCCGAGAGAACCCGGCTGAAGAGAACCGCCAGAAGAAGATACACCAGACGGAAGA
ACCGGATCTGCTATCTGCAAGAGATCTTCAGCAACGAGATGGCCAAGGTGGACGACAGCTTCTTCCA
CAGACTGGAAGAGTCCTTCCTGGTGGAAGAGGATAAGAAGCACGAGCGGCACCCCATCTTCGGCAA
CATCGTGGACGAGGTGGCCTACCACGAGAAGTACCCCACCATCTACCACCTGAGAAAGAAACTGGTG
GACAGCACCGACAAGGCCGACCTGCGGCTGATCTATCTGGCCCTGGCCCACATGATCAAGTTCCGG
GGCCACTTCCTGATCGAGGGCGACCTGAACCCCGACAACAGCGACGTGGACAAGCTGTTCATCCAG
CTGGTGCAGACCTACAACCAGCTGTTCGAGGAAAACCCCATCAACGCCAGCGGCGTGGACGCCAAG
GCCATCCTGTCTGCCAGACTGAGCAAGAGCAGACGGCTGGAAAATCTGATCGCCCAGCTGCCCGGC
GAGAAGAAGAATGGCCTGTTCGGAAACCTGATTGCCCTGAGCCTGGGCCTGACCCCCAACTTCAAG
AGCAACTTCGACCTGGCCGAGGATGCCAAACTGCAGCTGAGCAAGGACACCTACGACGACGACCTG
GACAACCTGCTGGCCCAGATCGGCGACCAGTACGCCGACCTGTTTCTGGCCGCCAAGAACCTGTCC
GACGCCATCCTGCTGAGCGACATCCTGAGAGTGAACACCGAGATCACCAAGGCCCCCCTGAGCGCC
TCTATGATCAAGAGATACGACGAGCACCACCAGGACCTGACCCTGCTGAAAGCTCTCGTGCGGCAGC
AGCTGCCTGAGAAGTACAAAGAGATTTTCTTCGACCAGAGCAAGAACGGCTACGCCGGCTACATTGA
CGGCGGAGCCAGCCAGGAAGAGTTCTACAAGTTCATCAAGCCCATCCTGGAAAAGATGGACGGCAC
CGAGGAACTGCTCGTGAAGCTGAACAGAGAGGACCTGCTGCGGAAGCAGCGGACCTTCGACAACG
GCAGCATCCCCCACCAGATCCACCTGGGAGAGCTGCACGCCATTCTGCGGCGGCAGGAAGATTTTT
ACCCATTCCTGAAGGACAACCGGGAAAAGATCGAGAAGATCCTGACCTTCCGCATCCCCTACTACGT
GGGCCCTCTGGCCAGGGGAAACAGCAGATTCGCCTGGATGACCAGAAAGAGCGAGGAAACCATCAC
CCCCTGGAACTTCGAGGAAGTGGTGGACAAGGGCGCTTCCGCCCAGAGCTTCATCGAGCGGATGAC
CAACTTCGATAAGAACCTGCCCAACGAGAAGGTGCTGCCCAAGCACAGCCTGCTGTACGAGTACTTC
ACCGTGTATAACGAGCTGACCAAAGTGAAATACGTGACCGAGGGAATGAGAAAGCCCGCCTTCCTGA
GCGGCGAGCAGAAAAAGGCCATCGTGGACCTGCTGTTCAAGACCAACCGGAAAGTGACCGTGAAGC
AGCTGAAAGAGGACTACTTCAAGAAAATCGAGTGCTTCGACTCCGTGGAAATCTCCGGCGTGGAAGA
TCGGTTCAACGCCTCCCTGGGCACATACCACGATCTGCTGAAAATTATCAAGGACAAGGACTTCCTG
GACAATGAGGAAAACGAGGACATTCTGGAAGATATCGTGCTGACCCTGACACTGTTTGAGGACAGAG
AGATGATCGAGGAACGGCTGAAAACCTATGCCCACCTGTTCGACGACAAAGTGATGAAGCAGCTGAA

GCGGCGGAGATACACCGGCTGGGGCAGGCTGAGCCGGAAGCTGATCAACGGCATCCGGGACAAGC
AGTCCGGCAAGACAATCCTGGATTTCCTGAAGTCCGACGGCTTCGCCAACAGAAACTTCATGCAGCT
GATCCACGACGACAGCCTGACCTTTAAAGAGGACATCCAGAAAGCCCAGGTGTCCGGCCAGGGCGA
TAGCCTGCACGAGCACATTGCCAATCTGGCCGGCAGCCCCGCCATTAAGAAGGGCATCCTGCAGAC
AGTGAAGGTGGTGGACGAGCTCGTGAAAGTGATGGGCCGGCACAAGCCCGAGAACATCGTGATCGA
AATGGCCAGAGAGAACCAGACCACCCAGAAGGGACAGAAGAACAGCCGCGAGAGAATGAAGCGGAT
CGAAGAGGGCATCAAAGAGCTGGGCAGCCAGATCCTGAAAGAACACCCCGTGGAAAACACCCAGCT
GCAGAACGAGAAGCTGTACCTGTACTACCTGCAGAATGGGCGGGATATGTACGTGGACCAGGAACTG
GACATCAACCGGCTGTCCGACTACGATGTGGACCATATCGTGCCTCAGAGCTTTCTGAAGGACGACT
CCATCGACAACAAGGTGCTGACCAGAAGCGACAAGAACCGGGGCAAGAGCGACAACGTGCCCTCC
GAAGAGGTCGTGAAGAAGATGAAGAACTACTGGCGGCAGCTGCTGAACGCCAAGCTGATTACCCAG
AGAAAGTTCGACAATCTGACCAAGGCCGAGAGAGGCGGCCTGAGCGAACTGGATAAGGCCGGCTTC
ATCAAGAGACAGCTGGTGGAAACCCGGCAGATCACAAAGCACGTGGCACAGATCCTGGACTCCCGG
ATGAACACTAAGTACGACGAGAATGACAAGCTGATCCGGGAAGTGAAAGTGATCACCCTGAAGTCCA
AGCTGGTGTCCGATTTCCGGAAGGATTTCCAGTTTTACAAAGTGCGCGAGATCAACAACTACCACCAC
GCCCACGACGCCTACCTGAACGCCGTCGTGGGAACCGCCCTGATCAAAAAGTACCCTAAGCTGGAA
AGCGAGTTCGTGTACGGCGACTACAAGGTGTACGACGTGCGGAAGATGATCGCCAAGAGCGAGCAG
GAAATCGGCAAGGCTACCGCCAAGTACTTCTTCTACAGCAACATCATGAACTTTTTCAAGACCGAGAT
TACCCTGGCCAACGGCGAGATCCGGAAGCGGCCTCTGATCGAGACAAACGGCGAAACCGGGGAGA
TCGTGTGGGATAAGGGCCGGGATTTTGCCACCGTGCGGAAAGTGCTGAGCATGCCCCAAGTGAATAT
CGTGAAAAAGACCGAGGTGCAGACAGGCGGCTTCAGCAAAGAGTCTATCAGACCCAAGAGGAACAG
CGATAAGCTGATCGCCAGAAAGAAGGACTGGGACCCTAAGAAGTACGGCGGCTTCCTGTGGCCCAC
CGTGGCCTATTCTGTGCTGGTGGTGGCCAAAGTGGAAAAGGGCAAGTCCAAGAAACTGAAGAGTGT
GAAAGAGCTGCTGGGGATCACCATCATGGAAAGAAGCAGCTTCGAGAAGAATCCCATCGACTTTCTG
GAAGCCAAGGGCTACAAAGAAGTGAAAAAGGACCTGATCATCAAGCTGCCTAAGTACTCCCTGTTCG
AGCTGGAAAACGGCCGGAAGAGAATGCTGGCCTCTGCCAAGCAGCTGCAGAAGGGAAACGAACTG
GCCCTGCCCTCCAAATATGTGAACTTCCTGTACCTGGCCAGCCACTATGAGAAGCTGAAGGGCTCCC
CCGAGGATAATGAGCAGAAACAGCTGTTTGTGGAACAGCACAAGCACTACCTGGACGAGATCATCGA
GCAGATCAGCGAGTTCTCCAAGAGAGTGATCCTGGCCGACGCTAATCTGGACAAAGTGCTGTCCGC
CTACAACAAGCACCGGGATAAGCCCATCAGAGAGCAGGCCGAGAATATCATCCACCTGTTTACCCTGA
CCAGACTGGGAGCCCCTAGAGCCTTCAAGTACTTTGACACCACCATCGACCCCAAGCAGTACAGAAG
CACCAAAGAGGTGCTGGACGCCACCCTGATCCACCAGAGCATCACCGGCCTGTACGAGACACGGAT
CGACCTGTCTCAGCTGGGAGGTGACAGCGGCGGGAGCGGCGGGAGCGGGGGGAGCACTAATCTG
AGCGACATCATTGAGAAGGAGACTGGGAAACAGCTGGTCATTCAGGAGTCCATCCTGATGCTGCCTG
AGGAGGTGGAGGAAGTGATCGGCAACAAGCCAGAGTCTGACATCCTGGTGCACACCGCCTACGACG
AGTCCACAGATGAGAATGTGATGCTGCTGACCTCTGACGCCCCCGAGTATAAGCCTTGGGCCCTGGT
CATCCAGGATTCTAACGGCGAGAATAAGATCAAGATGCTGAGCGGAGGATCCGGAGGATCTGGAGGC
AGC_ACCAACCTGTCTGACATCATCGAGAAGGAGACAGGCAAGCAGCTGGTCATCCAGGAGAGCATC_
CTGATGCTGCCCGAAGAAGTCGAAGAAGTGATCGGAAACAAGCCTGAGAGCGATATCCTGGTCCATA
CCGCCTACGACGAGAGTACCGACGAAAATGTGATGCTGCTGACATCCGACGCCCCAGAGTATAAGCC
CTGGGCTCTGGTCATCCAGGATTCCAACGGAGAGAACAAAATCAAAATGCTG**TCTGGCGGCTCAAAA**
AGAACCGCCGACGGCAGCGAATTCGAGCCCAAGAAGAAGAGGAAAGTCTAATTAATTAAGCTGCC
TTCTGCGGGGCTTGCCTTCTGGCCATGCCCTTCTTCTCTCCCTTGCACCTGTACCTCTTGGTCTTTGA
ATAAAGCCTGAGTAGGAAGCGACTGTGCCTTCTAGTTGCCAGCCATCTGTTGTTTGCCCCTCCCCCG
TGCCTTCCTTGACCCTGGAAGGTGCCACTCCCACTGTCCTTTCCTAATAAAATGAGAAATTGCATCG
CATTGTCTGAGTAGGTGTCATTCTATTCTGGGGGGTGGGGTGGGGCAGGACAGCAAGGGGGAGGAT
TGGGAAGACAATAGCAGGCATGCTGGGGATGCGGTGGGCTCTATGG

Start	End	Feature Description
1	380	CMV enhancer
381	619	CMV promoter
620	636	T7 promoter with mismatch

637	683	5' UTR
684	740	BP NLS
741	1424	Engineered BE4max deaminase
1425	1520	BE4max linker
1521	5621	Cas9(D10A) Sp-RY PAM variant
5622	5651	Linker
5652	5900	uracil glycosylase inhibitor
5901	5930	Linker
5931	*6179*	*uracil glycosylase inhibitor*
**6180**	**6245**	**linker + SV40 BP NLS**
6246	6346	3' UTR
6347	6574	bGH poly(A) signal

Amino acid sequence
MKRTADGSEFESPKKKRKVSSETGPVAVDPTLRRRIEPHEFEVFFDPRELRKETCLLYEINWGGRHSIWR
HTSQNTNKHVEVNFIEKFTTERYFCPNTRCSITWFLSWSPCGECSRAITEFLSRYPHVTLFIYIARLYHHAD
PRNRQGLRDLISSGVTIQIMTEQESGYCWRNFVNYSPSNEAHWPRYPHLWVRLYVLELYCIILGLPPCLNI
LRRKQPQLTFFTIALQSCHYQRLPPHILWATGLKSGGSSGGSSGSETPGTSESATPESSGGSSGGSDKKY
SIGLAIGTNSVGWAVITDEYKVPSKKFKVLGNTDRHSIKKNLIGALLFDSGETAERTRLKRTARRRYTRRKN
RICYLQEIFSNEMAKVDDSFFHRLEESFLVEEDKKHERHPIFGNIVDEVAYHEKYPTIYHLRKKLVDSTDKA
DLRLIYLALAHMIKFRGHFLIEGDLNPDNSDVDKLFIQLVQTYNQLFEENPINASGVDAKAILSARLSKSRRL
ENLIAQLPGEKKNGLFGNLIALSLGLTPNFKSNFDLAEDAKLQLSKDTYDDDLDNLLAQIGDQYADLFLAAK
NLSDAILLSDILRVNTEITKAPLSASMIKRYDEHHQDLTLLKALVRQQLPEKYKEIFFDQSKNGYAGYIDGGA
SQEEFYKFIKPILEKMDGTEELLVKLNREDLLRKQRTFDNGSIPHQIHLGELHAILRRQEDFYPFLKDNREKI
EKILTFRIPYYVGPLARGNSRFAWMTRKSEETITPWNFEEVVDKGASAQSFIERMTNFDKNLPNEKVLPKH
SLLYEYFTVYNELTKVKYVTEGMRKPAFLSGEQKKAIVDLLFKTNRKVTVKQLKEDYFKKIECFDSVEISGV
EDRFNASLGTYHDLLKIIKDKDFLDNEENEDILEDIVLTLTLFEDREMIEERLKTYAHLFDDKVMKQLKRRRY
TGWGRLSRKLINGIRDKQSGKTILDFLKSDGFANRNFMQLIHDDSLTFKEDIQKAQVSGQGDSLHEHIANL
AGSPAIKKGILQTVKVVDELVKVMGRHKPENIVIEMARENQTTQKGQKNSRERMKRIEEGIKELGSQILKE
HPVENTQLQNEKLYLYYLQNGRDMYVDQELDINRLSDYDVDHIVPQSFLKDDSIDNKVLTRSDKNRGKSD
NVPSEEVVKKMKNYWRQLLNAKLITQRKFDNLTKAERGGLSELDKAGFIKRQLVETRQITKHVAQILDSRM
NTKYDENDKLIREVKVITLKSKLVSDFRKDFQFYKVREINNYHHAHDAYLNAVVGTALIKKYPKLESEFVYG
DYKVYDVRKMIAKSEQEIGKATAKYFFYSNIMNFFKTEITLANGEIRKRPLIETNGETGEIVWDKGRDFATV
RKVLSMPQVNIVKKTEVQTGGFSKESIRPKRNSDKLIARKKDWDPKKYGGFLWPTVAYSVLVVAKVEKGK
SKKLKSVKELLGITIMERSSFEKNPIDFLEAKGYKEVKKDLIIKLPKYSLFELENGRKRMLASAKQLQKGNEL
ALPSKYVNFLYLASHYEKLKGSPEDNEQKQLFVEQHKHYLDEIIEQISEFSKRVILADANLDKVLSAYNKHR
DKPIREQAENIIHLFTLTRLGAPRAFKYFDTTIDPKQYRSTKEVLDATLIHQSITGLYETRIDLSQLGGDSGGS
GGSGGSTNLSDIIEKETGKQLVIQESILMLPEEVEEVIGNKPESDILVHTAYDESTDENVMLLTSDAPEYKP
WALVIQDSNGENKIKMLSGGSGGSGGS*TNLSDIIEKETGKQLVIQESILMLPEEVEEVIGNKPESDILVHTAY*
*DESTDENVMLLTSDAPEYKPWALVIQDSNGENKIKML***SGGSKRTADGSEFEPKKKRKV**.

Start	End	Feature Description
1	19	BP NLS
20	247	Engineered BE4max deaminase

248	279	BE4max linker
280	1646	Cas9(D10A) Sp-RY PAM variant
1647	1656	Linker
1657	1739	uracil glycosylase inhibitor
1740	1749	Linker
1750	*1832*	*uracil glycosylase inhibitor*
**1833**	**1853**	**linker + SV40 BP NLS**

YE1-BE3 – Cas9 (D10A) – SpRY variant – UGI
Plasmid ID: pHS0421

DNA sequence

GACATTGATTATTGACTAGTTATTAATAGTAATCAATTACGGGGTCATTAGTTCATAGCCCATATATGGAG
TTCCGCGTTACATAACTTACGGTAAATGGCCCGCCTGGCTGACCGCCCAACGACCCCCGCCCATTGA
CGTCAATAATGACGTATGTTCCCATAGTAACGCCAATAGGGACTTTCCATTGACGTCAATGGGTGGAGT
ATTTACGGTAAACTGCCCACTTGGCAGTACATCAAGTGTATCATATGCCAAGTACGCCCCCTATTGACG
TCAATGACGGTAAATGGCCCGCCTGGCATTATGCCCAGTACATGACCTTATGGGACTTTCCTACTTGG
CAGTACATCTACGTATTAGTCATCGCTATTACCATGGTGATGCGGTTTTGGCAGTACATCAATGGGCGT
GGATAGCGGTTTGACTCACGGGGATTTCCAAGTCTCCACCCCATTGACGTCAATGGGAGTTTGTTTTG
GCACCAAAATCAACGGGACTTTCCAAAATGTCGTAACAACTCCGCCCCATTGACGCAAATGGGCGGT
AGGCGTGTACGGTGGGAGGTCTATATAAGCAGAGCTGGTTTAGTGAACCGTCAGATCTCGAGCTCGG
TACCTAATACGACACACTATAAGGAAATAAGAGAGAAAAGAAGAGTAAGAAGAAATATAAGAGCCACCA
TGAAACGGACAGCCGACGGAAGCGAGTTCGAGTCACCAAAGAAGAAGCGGAAAGTCTCCTCAGAGA
CTGGGCCTGTCGCCGTCGATCCAACCCTGCGCCGCCGGATTGAACCTCACGAGTTTGAAGTGTTCTT
TGACCCCCGGGAGCTGAGAAAGGAGACATGCCTGCTGTACGAGATCAACTGGGGAGGCAGGCACTC
CATCTGGAGGCACACCTCTCAGAACACAAATAAGCACGTGGAGGTGAACTTCATCGAGAAGTTTACC
ACAGAGCGGTACTTCTGCCCCAATACCAGATGTAGCATCACATGGTTTCTGAGCTATTCCCCTTGCGG
AGAGTGTAGCAGGGCCATCACCGAGTTCCTGTCCAGATATCCACACGTGACACTGTTTATCTACATCG
CCAGGCTGTATCACCACGCAGACCCAGAGAATAGGCAGGGCCTGCGCGATCTGATCAGCTCCGGCG
TGACCATCCAGATCATGACAGAGCAGGAGTCCGGCTACTGCTGGCGGAACTTCGTGAATTATTCTCCT
AGCAACGAGGCCCACTGGCCTAGGTACCCACACCTGTGGGTGCGCCTGTACGTGCTGGAGCTGTAT
TGCATCATCCTGGGCCTGCCCCCTTGTCTGAATATCCTGCGGAGAAAGCAGCCCCAGCTGACCTTCT
TTACAATCGCCCTGCAGTCTTGTCACTATCAGAGGCTGCCACCCCACATCCTGTGGGCCACAGGCCT
GAAGTCTGGAGGATCTAGCGGAGGATCCTCTGGCAGCGAGACACCAGGAACAAGCGAGTCAGCAAC
ACCAGAGAGCAGTGGCGGCAGCAGCGGCGGCAGCGACAAGAAGTACAGCATCGGCCTGGCCATCG
GCACCAACTCTGTGGGCTGGGCCGTGATCACCGACGAGTACAAGGTGCCCAGCAAGAAATTCAAGG
TGCTGGGCAACACCGACCGGCACAGCATCAAGAAGAACCTGATCGGAGCCCTGCTGTTCGACAGCG
GCGAAACAGCCGAGAGAACCCGGCTGAAGAGAACCGCCAGAAGAAGATACACCAGACGGAAGAAC
CGGATCTGCTATCTGCAAGAGATCTTCAGCAACGAGATGGCCAAGGTGGACGACAGCTTCTTCCACA
GACTGGAAGAGTCCTTCCTGGTGGAAGAGGATAAGAAGCACGAGCGGCACCCCATCTTCGGCAACA
TCGTGGACGAGGTGGCCTACCACGAGAAGTACCCCACCATCTACCACCTGAGAAAGAAACTGGTGG
ACAGCACCGACAAGGCCGACCTGCGGCTGATCTATCTGGCCCTGGCCCACATGATCAAGTTCCGGG
GCCACTTCCTGATCGAGGGCGACCTGAACCCCGACAACAGCGACGTGGACAAGCTGTTCATCCAGC
TGGTGCAGACCTACAACCAGCTGTTCGAGGAAAACCCCATCAACGCCAGCGGCGTGGACGCCAAGG
CCATCCTGTCTGCCAGACTGAGCAAGAGCAGACGGCTGGAAAATCTGATCGCCCAGCTGCCCGGCG
AGAAGAAGAATGGCCTGTTCGGAAACCTGATTGCCCTGAGCCTGGGCCTGACCCCCAACTTCAAGA
GCAACTTCGACCTGGCCGAGGATGCCAAACTGCAGCTGAGCAAGGACACCTACGACGACGACCTGG
ACAACCTGCTGGCCCAGATCGGCGACCAGTACGCCGACCTGTTTCTGGCCGCCAAGAACCTGTCCG
ACGCCATCCTGCTGAGCGACATCCTGAGAGTGAACACCGAGATCACCAAGGCCCCCCTGAGCGCCT
CTATGATCAAGAGATACGACGAGCACCACCAGGACCTGACCCTGCTGAAAGCTCTCGTGCGGCAGCA
GCTGCCTGAGAAGTACAAAGAGATTTTCTTCGACCAGAGCAAGAACGGCTACGCCGGCTACATTGAC
GGCGGAGCCAGCCAGGAAGAGTTCTACAAGTTCATCAAGCCCATCCTGGAAAAGATGGACGGCACC
GAGGAACTGCTCGTGAAGCTGAACAGAGAGGACCTGCTGCGGAAGCAGCGGACCTTCGACAACGG
CAGCATCCCCCACCAGATCCACCTGGGAGAGCTGCACGCCATTCTGCGGCGGCAGGAAGATTTTTAC
CCATTCCTGAAGGACAACCGGGAAAAGATCGAGAAGATCCTGACCTTCCGCATCCCCTACTACGTGG
GCCCTCTGGCCAGGGGAAACAGCAGATTCGCCTGGATGACCAGAAAGAGCGAGGAAACCATCACCC
CCTGGAACTTCGAGGAAGTGGTGGACAAGGGCGCTTCCGCCCAGAGCTTCATCGAGCGGATGACCA
ACTTCGATAAGAACCTGCCCAACGAGAAGGTGCTGCCCAAGCACAGCCTGCTGTACGAGTACTTCAC
CGTGTATAACGAGCTGACCAAAGTGAAATACGTGACCGAGGGAATGAGAAAGCCCGCCTTCCTGAGC
GGCGAGCAGAAAAAGGCCATCGTGGACCTGCTGTTCAAGACCAACCGGAAAGTGACCGTGAAGCAG
CTGAAAGAGGACTACTTCAAGAAAATCGAGTGCTTCGACTCCGTGGAAATCTCCGGCGTGGAAGATC
GGTTCAACGCCTCCCTGGGCACATACCACGATCTGCTGAAAATTATCAAGGACAAGGACTTCCTGGA
CAATGAGGAAAACGAGGACATTCTGGAAGATATCGTGCTGACCCTGACACTGTTTGAGGACAGAGAG
ATGATCGAGGAACGGCTGAAAACCTATGCCCACCTGTTCGACGACAAAGTGATGAAGCAGCTGAAGC

GGCGGAGATACACCGGCTGGGGCAGGCTGAGCCGGAAGCTGATCAACGGCATCCGGGACAAGCAG
TCCGGCAAGACAATCCTGGATTTCCTGAAGTCCGACGGCTTCGCCAACAGAAACTTCATGCAGCTGA
TCCACGACGACAGCCTGACCTTTAAAGAGGACATCCAGAAAGCCCAGGTGTCCGGCCAGGGCGATA
GCCTGCACGAGCACATTGCCAATCTGGCCGGCAGCCCCGCCATTAAGAAGGGCATCCTGCAGACAG
TGAAGGTGGTGGACGAGCTCGTGAAAGTGATGGGCCGGCACAAGCCCGAGAACATCGTGATCGAAA
TGGCCAGAGAGAACCAGACCACCCAGAAGGGACAGAAGAACAGCCGCGAGAGAATGAAGCGGATC
GAAGAGGGCATCAAAGAGCTGGGCAGCCAGATCCTGAAAGAACACCCCGTGGAAAACACCCAGCTG
CAGAACGAGAAGCTGTACCTGTACTACCTGCAGAATGGGCGGGATATGTACGTGGACCAGGAACTGG
ACATCAACCGGCTGTCCGACTACGATGTGGACCATATCGTGCCTCAGAGCTTTCTGAAGGACGACTC
CATCGACAACAAGGTGCTGACCAGAAGCGACAAGAACCGGGGCAAGAGCGACAACGTGCCCTCCG
AAGAGGTCGTGAAGAAGATGAAGAACTACTGGCGGCAGCTGCTGAACGCCAAGCTGATTACCCAGA
GAAAGTTCGACAATCTGACCAAGGCCGAGAGAGGCGGCCTGAGCGAACTGGATAAGGCCGGCTTCA
TCAAGAGACAGCTGGTGGAAACCCGGCAGATCACAAAGCACGTGGCACAGATCCTGGACTCCCGGA
TGAACACTAAGTACGACGAGAATGACAAGCTGATCCGGGAAGTGAAAGTGATCACCCTGAAGTCCAA
GCTGGTGTCCGATTTCCGGAAGGATTTCCAGTTTTACAAAGTGCGCGAGATCAACAACTACCACCAC
GCCCACGACGCCTACCTGAACGCCGTCGTGGGAACCGCCCTGATCAAAAAGTACCCTAAGCTGGAA
AGCGAGTTCGTGTACGGCGACTACAAGGTGTACGACGTGCGGAAGATGATCGCCAAGAGCGAGCAG
GAAATCGGCAAGGCTACCGCCAAGTACTTCTTCTACAGCAACATCATGAACTTTTTCAAGACCGAGAT
TACCCTGGCCAACGGCGAGATCCGGAAGCGGCCTCTGATCGAGACAAACGGCGAAACCGGGGAGA
TCGTGTGGGATAAGGGCCGGGATTTTGCCACCGTGCGGAAAGTGCTGAGCATGCCCCAAGTGAATAT
CGTGAAAAAGACCGAGGTGCAGACAGGCGGCTTCAGCAAAGAGTCTATCAGACCCAAGAGGAACAG
CGATAAGCTGATCGCCAGAAAGAAGGACTGGGACCCTAAGAAGTACGGCGGCTTCCTGTGGCCCAC
CGTGGCCTATTCTGTGCTGGTGGTGGCCAAAGTGGAAAAGGGCAAGTCCAAGAAACTGAAGAGTGT
GAAAGAGCTGCTGGGGATCACCATCATGGAAAGAAGCAGCTTCGAGAAGAATCCCATCGACTTTCTG
GAAGCCAAGGGCTACAAAGAAGTGAAAAAGGACCTGATCATCAAGCTGCCTAAGTACTCCCTGTTCG
AGCTGGAAAACGGCCGGAAGAGAATGCTGGCCTCTGCCAAGCAGCTGCAGAAGGGAAACGAACTG
GCCCTGCCCTCCAAATATGTGAACTTCCTGTACCTGGCCAGCCACTATGAGAAGCTGAAGGGCTCCC
CCGAGGATAATGAGCAGAAACAGCTGTTTGTGGAACAGCACAAGCACTACCTGGACGAGATCATCGA
GCAGATCAGCGAGTTCTCCAAGAGAGTGATCCTGGCCGACGCTAATCTGGACAAAGTGCTGTCCGC
CTACAACAAGCACCGGGATAAGCCCATCAGAGAGCAGGCCGAGAATATCATCCACCTGTTTACCCTGA
CCAGACTGGGAGCCCCTAGAGCCTTCAAGTACTTTGACACCACCATCGACCCCAAGCAGTACAGAAG
CACCAAAGAGGTGCTGGACGCCACCCTGATCCACCAGAGCATCACCGGCCTGTACGAGACACGGAT
CGACCTGTCTCAGCTGGGAGGTGACAGCGGCGGGAGCGGCGGGAGCGGGGGGAGCACTAATCTG
AGCGACATCATTGAGAAGGAGACTGGGAAACAGCTGGTCATTCAGGAGTCCATCCTGATGCTGCCTG
AGGAGGTGGAGGAAGTGATCGGCAACAAGCCAGAGTCTGACATCCTGGTGCACACCGCCTACGACG
AGTCCACAGATGAGAATGTGATGCTGCTGACCTCTGACGCCCCCGAGTATAAGCCTTGGGCCCTGGT
CATCCAGGATTCTAACGGCGAGAATAAGATCAAGATGCTGAGCGGAGGATCCGGAGGATCTGGAGGC
AGC*ACCAACCTGTCTGACATCATCGAGAAGGAGACAGGCAAGCAGCTGGTCATCCAGGAGAGCATC*
CTGATGCTGCCCGAAGAAGTCGAAGAAGTGATCGGAAACAAGCCTGAGAGCGATATCCTGGTCCATA
CCGCCTACGACGAGAGTACCGACGAAAATGTGATGCTGCTGACATCCGACGCCCCAGAGTATAAGCC
*CTGGGCTCTGGTCATCCAGGATTCCAACGGAGAGAACAAAATCAAAATGCTG***TCTGGCGGCTCAAAA**
AGAACCGCCGACGGCAGCGAATTCGAGCCCAAGAAGAAGAGGAAAGTCTAATTAATTAAGCTGCC
TTCTGCGGGGCTTGCCTTCTGGCCATGCCCTTCTTCTCTCCCTTGCACCTGTACCTCTTGGTCTTTGA
ATAAAGCCTGAGTAGGAAGCGACTGTGCCTTCTAGTTGCCAGCCATCTGTTGTTTGCCCCTCCCCCG
TGCCTTCCTTGACCCTGGAAGGTGCCACTCCCACTGTCCTTTCCTAATAAAATGAGAAATTGCATCG
CATTGTCTGAGTAGGTGTCATTCTATTCTGGGGGGTGGGGTGGGGCAGGACAGCAAGGGGGAGGAT
TGGGAAGACAATAGCAGGCATGCTGGGGATGCGGTGGGCTCTATGG

Start	End	Feature Description
1	380	CMV enhancer
381	619	CMV promoter
620	636	T7 promoter with mismatch

637	683	5’ UTR
684	740	BP NLS
741	1424	YE1 deaminase
1425	1520	BE4max linker
1521	5621	Cas9(D10A) Sp-RY PAM variant
5622	5651	Linker
5652	5900	uracil glycosylase inhibitor
5901	5930	Linker
5931	*6179*	*uracil glycosylase inhibitor*
**6180**	**6245**	**linker + SV40 BP NLS**
6246	6346	3’ UTR
6347	6574	bGH poly(A) signal

Amino acid sequence
MKRTADGSEFESPKKKRKVSSETGPVAVDPTLRRRIEPHEFEVFFDPRELRKETCLLYEINWGGRHSIWR
HTSQNTNKHVEVNFIEKFTTERYFCPNTRCSITWFLSYSPCGECSRAITEFLSRYPHVTLFIYIARLYHHAD
PENRQGLRDLISSGVTIQIMTEQESGYCWRNFVNYSPSNEAHWPRYPHLWVRLYVLELYCIILGLPPCLNI
LRRKQPQLTFFTIALQSCHYQRLPPHILWATGLKSGGSSGGSSGSETPGTSESATPESSGGSSGGSDKKY
SIGLAIGTNSVGWAVITDEYKVPSKKFKVLGNTDRHSIKKNLIGALLFDSGETAERTRLKRTARRRYTRRKN
RICYLQEIFSNEMAKVDDSFFHRLEESFLVEEDKKHERHPIFGNIVDEVAYHEKYPTIYHLRKKLVDSTDKA
DLRLIYLALAHMIKFRGHFLIEGDLNPDNSDVDKLFIQLVQTYNQLFEENPINASGVDAKAILSARLSKSRRL
ENLIAQLPGEKKNGLFGNLIALSLGLTPNFKSNFDLAEDAKLQLSKDTYDDDLDNLLAQIGDQYADLFLAAK
NLSDAILLSDILRVNTEITKAPLSASMIKRYDEHHQDLTLLKALVRQQLPEKYKEIFFDQSKNGYAGYIDGGA
SQEEFYKFIKPILEKMDGTEELLVKLNREDLLRKQRTFDNGSIPHQIHLGELHAILRRQEDFYPFLKDNREKI
EKILTFRIPYYVGPLARGNSRFAWMTRKSEETITPWNFEEVVDKGASAQSFIERMTNFDKNLPNEKVLPKH
SLLYEYFTVYNELTKVKYVTEGMRKPAFLSGEQKKAIVDLLFKTNRKVTVKQLKEDYFKKIECFDSVEISGV
EDRFNASLGTYHDLLKIIKDKDFLDNEENEDILEDIVLTLTLFEDREMIEERLKTYAHLFDDKVMKQLKRRRY
TGWGRLSRKLINGIRDKQSGKTILDFLKSDGFANRNFMQLIHDDSLTFKEDIQKAQVSGQGDSLHEHIANL
AGSPAIKKGILQTVKVVDELVKVMGRHKPENIVIEMARENQTTQKGQKNSRERMKRIEEGIKELGSQILKE
HPVENTQLQNEKLYLYYLQNGRDMYVDQELDINRLSDYDVDHIVPQSFLKDDSIDNKVLTRSDKNRGKSD
NVPSEEVVKKMKNYWRQLLNAKLITQRKFDNLTKAERGGLSELDKAGFIKRQLVETRQITKHVAQILDSRM
NTKYDENDKLIREVKVITLKSKLVSDFRKDFQFYKVREINNYHHAHDAYLNAVVGTALIKKYPKLESEFVYG
DYKVYDVRKMIAKSEQEIGKATAKYFFYSNIMNFFKTEITLANGEIRKRPLIETNGETGEIVWDKGRDFATV
RKVLSMPQVNIVKKTEVQTGGFSKESIRPKRNSDKLIARKKDWDPKKYGGFLWPTVAYSVLVVAKVEKGK
SKKLKSVKELLGITIMERSSFEKNPIDFLEAKGYKEVKKDLIIKLPKYSLFELENGRKRMLASAKQLQKGNEL
ALPSKYVNFLYLASHYEKLKGSPEDNEQKQLFVEQHKHYLDEIIEQISEFSKRVILADANLDKVLSAYNKHR
DKPIREQAENIIHLFTLTRLGAPRAFKYFDTTIDPKQYRSTKEVLDATLIHQSITGLYETRIDLSQLGGDSGGS
GGSGGSTNLSDIIEKETGKQLVIQESILMLPEEVEEVIGNKPESDILVHTAYDESTDENVMLLTSDAPEYKP
WALVIQDSNGENKIKMLSGGSGGSGGS*TNLSDIIEKETGKQLVIQESILMLPEEVEEVIGNKPESDILVHTAY*
*DESTDENVMLLTSDAPEYKPWALVIQDSNGENKIKML***SGGSKRTADGSEFEPKKKRKV**.

Start	End	Feature Description
1	19	BP NLS
20	247	YE1 deaminase

248	279	BE4max linker
280	1646	Cas9(D10A) Sp-RY PAM variant
1647	1656	Linker
1657	1739	uracil glycosylase inhibitor
1740	1749	Linker
1750	*1832*	*uracil glycosylase inhibitor*
**1833**	**1853**	**linker + SV40 BP NLS**

TadCBEd – Cas9 (D10A) – SpRY variant – UGI
Plasmid ID: pHS0423

DNA sequence

GACATTGATTATTGACTAGTTATTAATAGTAATCAATTACGGGGTCATTAGTTCATAGCCCATATATGGAG
TTCCGCGTTACATAACTTACGGTAAATGGCCCGCCTGGCTGACCGCCCAACGACCCCCGCCCATTGA
CGTCAATAATGACGTATGTTCCCATAGTAACGCCAATAGGGACTTTCCATTGACGTCAATGGGTGGAGT
ATTTACGGTAAACTGCCCACTTGGCAGTACATCAAGTGTATCATATGCCAAGTACGCCCCCTATTGACG
TCAATGACGGTAAATGGCCCGCCTGGCATTATGCCCAGTACATGACCTTATGGGACTTTCCTACTTGG
CAGTACATCTACGTATTAGTCATCGCTATTACCATGGTGATGCGGTTTTGGCAGTACATCAATGGGCGT
GGATAGCGGTTTGACTCACGGGGATTTCCAAGTCTCCACCCCATTGACGTCAATGGGAGTTTGTTTTG
GCACCAAAATCAACGGGACTTTCCAAAATGTCGTAACAACTCCGCCCCATTGACGCAAATGGGCGGT
AGGCGTGTACGGTGGGAGGTCTATATAAGCAGAGCTGGTTTAGTGAACCGTCAGATCTCGAGCTCGG
TACCTAATACGACACACTATAAGGAAATAAGAGAGAAAAGAAGAGTAAGAAGAAATATAAGAGCCACCA
TGAAACGGACAGCCGACGGAAGCGAGTTCGAGTCACCAAAGAAGAAGCGGAAAGTCAGTTCTGAGG
TGGAGTTTTCCCACGAGTACTGGATGAGACATGCCCTGACCCTGGCCAAGAGGGCACGGGATGAGA
GGAAGGCGCCTGTGGGAGCCGTGCTGGTGCTGAACAATAGAGTGATCGGCGAGGGCTGGAACAGA
GCCATCGGCCTGCACGACCCAACAGCCCATGCCGAAATTATAGCCCTGAGACAGGGCGGCCTGGTC
ATGCAGAACTACAGACTGATTGACGCCACCCTGTACGTGACATTCGAGCCTTGCGTGATGTGCGCCG
GCGCCATGATCAACTCTAGGATCGGCCGCGTGGTGTTTGGCGTGAGGAACTCAAAAAGAGGCGCCG
CAGGCTCCCTGATGAACGTGCTGAACTACCCCGGAATGAATCACCGCGTCGAAATTACCGAGGGAAT
CCTGGCAGATGAATGTGCCGCCCTGCTGTGCGATTTCTATCGGATGCCTAGACAGGTGTTCAATGCTC
AGAAGAAGGCCCAGAGCTCCATCAACTCTGGCGGATCTAGCGGAGGATCCTCTGGCAGCGAGACAC
CAGGAACAAGCGAGTCAGCAACACCAGAGAGCAGTGGCGGCAGCAGCGGCGGCAGCGACAAGAA
GTACAGCATCGGCCTGGCCATCGGCACCAACTCTGTGGGCTGGGCCGTGATCACCGACGAGTACAA
GGTGCCCAGCAAGAAATTCAAGGTGCTGGGCAACACCGACCGGCACAGCATCAAGAAGAACCTGAT
CGGAGCCCTGCTGTTCGACAGCGGCGAAACAGCCGAGAGAACCCGGCTGAAGAGAACCGCCAGAA
GAAGATACACCAGACGGAAGAACCGGATCTGCTATCTGCAAGAGATCTTCAGCAACGAGATGGCCAA
GGTGGACGACAGCTTCTTCCACAGACTGGAAGAGTCCTTCCTGGTGGAAGAGGATAAGAAGCACGA
GCGGCACCCCATCTTCGGCAACATCGTGGACGAGGTGGCCTACCACGAGAAGTACCCCACCATCTA
CCACCTGAGAAAGAAACTGGTGGACAGCACCGACAAGGCCGACCTGCGGCTGATCTATCTGGCCCT
GGCCCACATGATCAAGTTCCGGGGCCACTTCCTGATCGAGGGCGACCTGAACCCCGACAACAGCGA
CGTGGACAAGCTGTTCATCCAGCTGGTGCAGACCTACAACCAGCTGTTCGAGGAAAACCCCATCAAC
GCCAGCGGCGTGGACGCCAAGGCCATCCTGTCTGCCAGACTGAGCAAGAGCAGACGGCTGGAAAA
TCTGATCGCCCAGCTGCCCGGCGAGAAGAAGAATGGCCTGTTCGGAAACCTGATTGCCCTGAGCCT
GGGCCTGACCCCCAACTTCAAGAGCAACTTCGACCTGGCCGAGGATGCCAAACTGCAGCTGAGCAA
GGACACCTACGACGACGACCTGGACAACCTGCTGGCCCAGATCGGCGACCAGTACGCCGACCTGTT
TCTGGCCGCCAAGAACCTGTCCGACGCCATCCTGCTGAGCGACATCCTGAGAGTGAACACCGAGAT
CACCAAGGCCCCCCTGAGCGCCTCTATGATCAAGAGATACGACGAGCACCACCAGGACCTGACCCT
GCTGAAAGCTCTCGTGCGGCAGCAGCTGCCTGAGAAGTACAAAGAGATTTTCTTCGACCAGAGCAAG
AACGGCTACGCCGGCTACATTGACGGCGGAGCCAGCCAGGAAGAGTTCTACAAGTTCATCAAGCCC
ATCCTGGAAAAGATGGACGGCACCGAGGAACTGCTCGTGAAGCTGAACAGAGAGGACCTGCTGCGG
AAGCAGCGGACCTTCGACAACGGCAGCATCCCCCACCAGATCCACCTGGGAGAGCTGCACGCCATT
CTGCGGCGGCAGGAAGATTTTTACCCATTCCTGAAGGACAACCGGGAAAAGATCGAGAAGATCCTGA
CCTTCCGCATCCCCTACTACGTGGGCCCTCTGGCCAGGGGAAACAGCAGATTCGCCTGGATGACCA
GAAAGAGCGAGGAAACCATCACCCCCTGGAACTTCGAGGAAGTGGTGGACAAGGGCGCTTCCGCC
CAGAGCTTCATCGAGCGGATGACCAACTTCGATAAGAACCTGCCCAACGAGAAGGTGCTGCCCAAG
CACAGCCTGCTGTACGAGTACTTCACCGTGTATAACGAGCTGACCAAAGTGAAATACGTGACCGAGG
GAATGAGAAAGCCCGCCTTCCTGAGCGGCGAGCAGAAAAAGGCCATCGTGGACCTGCTGTTCAAGA
CCAACCGGAAAGTGACCGTGAAGCAGCTGAAAGAGGACTACTTCAAGAAAATCGAGTGCTTCGACTC
CGTGGAAATCTCCGGCGTGGAAGATCGGTTCAACGCCTCCCTGGGCACATACCACGATCTGCTGAAA
ATTATCAAGGACAAGGACTTCCTGGACAATGAGGAAAACGAGGACATTCTGGAAGATATCGTGCTGAC
CCTGACACTGTTTGAGGACAGAGAGATGATCGAGGAACGGCTGAAAACCTATGCCCACCTGTTCGAC
GACAAAGTGATGAAGCAGCTGAAGCGGCGGAGATACACCGGCTGGGGCAGGCTGAGCCGGAAGCT
GATCAACGGCATCCGGGACAAGCAGTCCGGCAAGACAATCCTGGATTTCCTGAAGTCCGACGGCTT
CGCCAACAGAAACTTCATGCAGCTGATCCACGACGACAGCCTGACCTTTAAAGAGGACATCCAGAAA

GCCCAGGTGTCCGGCCAGGGCGATAGCCTGCACGAGCACATTGCCAATCTGGCCGGCAGCCCCGC
CATTAAGAAGGGCATCCTGCAGACAGTGAAGGTGGTGGACGAGCTCGTGAAAGTGATGGGCCGGCA
CAAGCCCGAGAACATCGTGATCGAAATGGCCAGAGAGAACCAGACCACCCAGAAGGGACAGAAGAA
CAGCCGCGAGAGAATGAAGCGGATCGAAGAGGGCATCAAAGAGCTGGGCAGCCAGATCCTGAAAGA
ACACCCCGTGGAAAACACCCAGCTGCAGAACGAGAAGCTGTACCTGTACTACCTGCAGAATGGGCG
GGATATGTACGTGGACCAGGAACTGGACATCAACCGGCTGTCCGACTACGATGTGGACCATATCGTG
CCTCAGAGCTTTCTGAAGGACGACTCCATCGACAACAAGGTGCTGACCAGAAGCGACAAGAACCGG
GGCAAGAGCGACAACGTGCCCTCCGAAGAGGTCGTGAAGAAGATGAAGAACTACTGGCGGCAGCT
GCTGAACGCCAAGCTGATTACCCAGAGAAAGTTCGACAATCTGACCAAGGCCGAGAGAGGCGGCCT
GAGCGAACTGGATAAGGCCGGCTTCATCAAGAGACAGCTGGTGGAAACCCGGCAGATCACAAAGCA
CGTGGCACAGATCCTGGACTCCCGGATGAACACTAAGTACGACGAGAATGACAAGCTGATCCGGGAA
GTGAAAGTGATCACCCTGAAGTCCAAGCTGGTGTCCGATTTCCGGAAGGATTTCCAGTTTTACAAAGT
GCGCGAGATCAACAACTACCACCACGCCCACGACGCCTACCTGAACGCCGTCGTGGGAACCGCCCT
GATCAAAAAGTACCCTAAGCTGGAAAGCGAGTTCGTGTACGGCGACTACAAGGTGTACGACGTGCGG
AAGATGATCGCCAAGAGCGAGCAGGAAATCGGCAAGGCTACCGCCAAGTACTTCTTCTACAGCAACA
TCATGAACTTTTTCAAGACCGAGATTACCCTGGCCAACGGCGAGATCCGGAAGCGGCCTCTGATCGA
GACAAACGGCGAAACCGGGGAGATCGTGTGGGATAAGGGCCGGGATTTTGCCACCGTGCGGAAAGT
GCTGAGCATGCCCCAAGTGAATATCGTGAAAAAGACCGAGGTGCAGACAGGCGGCTTCAGCAAAGA
GTCTATCAGACCCAAGAGGAACAGCGATAAGCTGATCGCCAGAAAGAAGGACTGGGACCCTAAGAAG
TACGGCGGCTTCCTGTGGCCCACCGTGGCCTATTCTGTGCTGGTGGTGGCCAAAGTGGAAAAGGGC
AAGTCCAAGAAACTGAAGAGTGTGAAAGAGCTGCTGGGGATCACCATCATGGAAAGAAGCAGCTTCG
AGAAGAATCCCATCGACTTTCTGGAAGCCAAGGGCTACAAAGAAGTGAAAAAGGACCTGATCATCAA
GCTGCCTAAGTACTCCCTGTTCGAGCTGGAAAACGGCCGGAAGAGAATGCTGGCCTCTGCCAAGCA
GCTGCAGAAGGGAAACGAACTGGCCCTGCCCTCCAAATATGTGAACTTCCTGTACCTGGCCAGCCAC
TATGAGAAGCTGAAGGGCTCCCCCGAGGATAATGAGCAGAAACAGCTGTTTGTGGAACAGCACAAGC
ACTACCTGGACGAGATCATCGAGCAGATCAGCGAGTTCTCCAAGAGAGTGATCCTGGCCGACGCTAA
TCTGGACAAAGTGCTGTCCGCCTACAACAAGCACCGGGATAAGCCCATCAGAGAGCAGGCCGAGAA
TATCATCCACCTGTTTACCCTGACCAGACTGGGAGCCCCTAGAGCCTTCAAGTACTTTGACACCACCA
TCGACCCCAAGCAGTACAGAAGCACCAAAGAGGTGCTGGACGCCACCCTGATCCACCAGAGCATCA
CCGGCCTGTACGAGACACGGATCGACCTGTCTCAGCTGGGAGGTGACAGCGGCGGGAGCGGCGG
GAGCGGGGGGAGCACTAATCTGAGCGACATCATTGAGAAGGAGACTGGGAAACAGCTGGTCATTCA
GGAGTCCATCCTGATGCTGCCTGAGGAGGTGGAGGAAGTGATCGGCAACAAGCCAGAGTCTGACAT
CCTGGTGCACACCGCCTACGACGAGTCCACAGATGAGAATGTGATGCTGCTGACCTCTGACGCCCC
CGAGTATAAGCCTTGGGCCCTGGTCATCCAGGATTCTAACGGCGAGAATAAGATCAAGATGCTGAGC
GGAGGATCCGGAGGATCTGGAGGCAGC*ACCAACCTGTCTGACATCATCGAGAAGGAGACAGGCAAG*
CAGCTGGTCATCCAGGAGAGCATCCTGATGCTGCCCGAAGAAGTCGAAGAAGTGATCGGAAACAAG
CCTGAGAGCGATATCCTGGTCCATACCGCCTACGACGAGAGTACCGACGAAAATGTGATGCTGCTGA
CATCCGACGCCCCAGAGTATAAGCCCTGGGCTCTGGTCATCCAGGATTCCAACGGAGAGAACAAAAT
*CAAAATGCTG***TCTGGCGGCTCAAAAAGAACCGCCGACGGCAGCGAATTCGAGCCCAAGAAGAAGA**
GGAAAGTCTAATTAATTAAGCTGCCTTCTGCGGGGCTTGCCTTCTGGCCATGCCCTTCTTCTCTCCCT
TGCACCTGTACCTCTTGGTCTTTGAATAAAGCCTGAGTAGGAAGCGACTGTGCCTTCTAGTTGCCAGC
CATCTGTTGTTTGCCCCTCCCCCGTGCCTTCCTTGACCCTGGAAGGTGCCACTCCCACTGTCCTTTC
CTAATAAAATGAGAAAATTGCATCGCATTGTCTGAGTAGGTGTCATTCTATTCTGGGGGGTGGGGTGG
GGCAGGACAGCAAGGGGGAGGATTGGGAAGACAATAGCAGGCATGCTGGGGATGCGGTGGGCTCT
ATGG

Start	End	Feature Description
1	380	CMV enhancer
381	619	CMV promoter
620	636	T7 promoter with mismatch
637	683	5’ UTR

684	740	BP NLS
741	1241	TadCBEd deaminase
1242	1337	linker
1338	5438	Cas9(D10A) Sp-RY PAM variant
5439	5468	Linker
5469	5717	uracil glycosylase inhibitor
5718	5747	Linker
5748	*5996*	*uracil glycosylase inhibitor*
**5997**	**6062**	**linker + SV40 BP NLS**
6063	6163	3’ UTR
6164	6391	bGH poly(A) signal

Amino acid sequence

MKRTADGSEFESPKKKRKVSSEVEFSHEYWMRHALTLAKRARDERKAPVGAVLVLNNRVIGEGWNRAIG
LHDPTAHAEIIALRQGGLVMQNYRLIDATLYVTFEPCVMCAGAMINSRIGRVVFGVRNSKRGAAGSLMNVL
NYPGMNHRVEITEGILADECAALLCDFYRMPRQVFNAQKKAQSSINSGGSSGGSSGSETPGTSESATPE
SSGGSSGGSDKKYSIGLAIGTNSVGWAVITDEYKVPSKKFKVLGNTDRHSIKKNLIGALLFDSGETAERTR
LKRTARRRYTRRKNRICYLQEIFSNEMAKVDDSFFHRLEESFLVEEDKKHERHPIFGNIVDEVAYHEKYPTI
YHLRKKLVDSTDKADLRLIYLALAHMIKFRGHFLIEGDLNPDNSDVDKLFIQLVQTYNQLFEENPINASGVD
AKAILSARLSKSRRLENLIAQLPGEKKNGLFGNLIALSLGLTPNFKSNFDLAEDAKLQLSKDTYDDDLDNLL
AQIGDQYADLFLAAKNLSDAILLSDILRVNTEITKAPLSASMIKRYDEHHQDLTLLKALVRQQLPEKYKEIFFD
QSKNGYAGYIDGGASQEEFYKFIKPILEKMDGTEELLVKLNREDLLRKQRTFDNGSIPHQIHLGELHAILRR
QEDFYPFLKDNREKIEKILTFRIPYYVGPLARGNSRFAWMTRKSEETITPWNFEEVVDKGASAQSFIERMT
NFDKNLPNEKVLPKHSLLYEYFTVYNELTKVKYVTEGMRKPAFLSGEQKKAIVDLLFKTNRKVTVKQLKED
YFKKIECFDSVEISGVEDRFNASLGTYHDLLKIIKDKDFLDNEENEDILEDIVLTLTLFEDREMIEERLKTYAH
LFDDKVMKQLKRRRYTGWGRLSRKLINGIRDKQSGKTILDFLKSDGFANRNFMQLIHDDSLTFKEDIQKAQ
VSGQGDSLHEHIANLAGSPAIKKGILQTVKVVDELVKVMGRHKPENIVIEMARENQTTQKGQKNSRERMK
RIEEGIKELGSQILKEHPVENTQLQNEKLYLYYLQNGRDMYVDQELDINRLSDYDVDHIVPQSFLKDDSIDN
KVLTRSDKNRGKSDNVPSEEVVKKMKNYWRQLLNAKLITQRKFDNLTKAERGGLSELDKAGFIKRQLVET
RQITKHVAQILDSRMNTKYDENDKLIREVKVITLKSKLVSDFRKDFQFYKVREINNYHHAHDAYLNAVVGTA
LIKKYPKLESEFVYGDYKVYDVRKMIAKSEQEIGKATAKYFFYSNIMNFFKTEITLANGEIRKRPLIETNGET
GEIVWDKGRDFATVRKVLSMPQVNIVKKTEVQTGGFSKESIRPKRNSDKLIARKKDWDPKKYGGFLWPTV
AYSVLVVAKVEKGKSKKLKSVKELLGITIMERSSFEKNPIDFLEAKGYKEVKKDLIIKLPKYSLFELENGRKR
MLASAKQLQKGNELALPSKYVNFLYLASHYEKLKGSPEDNEQKQLFVEQHKHYLDEIIEQISEFSKRVILAD
ANLDKVLSAYNKHRDKPIREQAENIIHLFTLTRLGAPRAFKYFDTTIDPKQYRSTKEVLDATLIHQSITGLYET
RIDLSQLGGDSGGSGGSGGSTNLSDIIEKETGKQLVIQESILMLPEEVEEVIGNKPESDILVHTAYDESTDE
NVMLLTSDAPEYKPWALVIQDSNGENKIKMLSGGSGGSGGS*TNLSDIIEKETGKQLVIQESILMLPEEVEEV*
*IGNKPESDILVHTAYDESTDENVMLLTSDAPEYKPWALVIQDSNGENKIKML***SGGSKRTADGSEFEPKKK**
RKV

Start	End	Feature Description
1	19	BP NLS
20	186	TadCBEd deaminase
187	218	BE4max linker

219	1585	Cas9(D10A) Sp-RY PAM variant
1586	1595	Linker
1596	1678	uracil glycosylase inhibitor
1679	1688	Linker
1689	*1771*	*uracil glycosylase inhibitor*
**1772**	**1792**	**linker + SV40 BP NLS**

CBE6b – Cas9 (D10A) – SpRY variant – UGI
Plasmid ID: pHS0424

DNA sequence

GACATTGATTATTGACTAGTTATTAATAGTAATCAATTACGGGGTCATTAGTTCATAGCCCATATATGGAG
TTCCGCGTTACATAACTTACGGTAAATGGCCCGCCTGGCTGACCGCCCAACGACCCCCGCCCATTGA
CGTCAATAATGACGTATGTTCCCATAGTAACGCCAATAGGGACTTTCCATTGACGTCAATGGGTGGAGT
ATTTACGGTAAACTGCCCACTTGGCAGTACATCAAGTGTATCATATGCCAAGTACGCCCCCTATTGACG
TCAATGACGGTAAATGGCCCGCCTGGCATTATGCCCAGTACATGACCTTATGGGACTTTCCTACTTGG
CAGTACATCTACGTATTAGTCATCGCTATTACCATGGTGATGCGGTTTTGGCAGTACATCAATGGGCGT
GGATAGCGGTTTGACTCACGGGGATTTCCAAGTCTCCACCCCATTGACGTCAATGGGAGTTTGTTTTG
GCACCAAAATCAACGGGACTTTCCAAAATGTCGTAACAACTCCGCCCCATTGACGCAAATGGGCGGT
AGGCGTGTACGGTGGGAGGTCTATATAAGCAGAGCTGGTTTAGTGAACCGTCAGATCTCGAGCTCGG
TACCTAATACGACACACTATAAGGAAATAAGAGAGAAAAGAAGAGTAAGAAGAAATATAAGAGCCACCA
TGAAACGGACAGCCGACGGAAGCGAGTTCGAGTCACCAAAGAAGAAGCGGAAAGTCAGTTCTGAGG
TGGAGTTTTCCCACGAGTACTGGATGAGACATGCCCTGACCCTGGCCAAGAGGGCACGGATGAGG
GAGAGGCGCCTGTGGGAGCCGTGCTGGTGCTGAACAATAGAGTGATCGGCGAGGGCTGGGTGAGA
CGTATCGGCCTGCACGACCCAACAGCCCATGCCGAAATTATGGCCCTGAGACAGGGCGGCCTGGTC
ATGCAGAACCCCAGACTGATTGACGCCACCCTGTACGTGACATTCGAGCCTTGCGTGATGTGCGCCG
GCGCCATGATCAACTCTAGGATCGGCCGCGTGGTGTTTGGCGTGAGGAACTCAAAAAGAGGCGCCG
CAGGCTCCCTGATGAACGTGCTGAACTACCCCGGCATGAATCACCGCGTCGAAATTACCGAGGGAAT
CCTGGCAGATGAATGTGCCGCCCTGCTGTGCGATTTCTATCGGATGCCTAGACAGGTGTTCAATGCTC
AGAAGAAGGCCCAGAGCTCCATCAACTCTGGCGGATCTAGCGGAGGATCCTCTGGCAGCGAGACAC
CAGGAACAAGCGAGTCAGCAACACCAGAGAGCAGTGGCGGCAGCAGCGGCGGCAGCGACAAGAA
GTACAGCATCGGCCTGGCCATCGGCACCAACTCTGTGGGCTGGGCCGTGATCACCGACGAGTACAA
GGTGCCCAGCAAGAAATTCAAGGTGCTGGGCAACACCGACCGGCACAGCATCAAGAAGAACCTGAT
CGGAGCCCTGCTGTTCGACAGCGGCGAAACAGCCGAGAGAACCCGGCTGAAGAGAACCGCCAGAA
GAAGATACACCAGACGGAAGAACCGGATCTGCTATCTGCAAGAGATCTTCAGCAACGAGATGGCCAA
GGTGGACGACAGCTTCTTCCACAGACTGGAAGAGTCCTTCCTGGTGGAAGAGGATAAGAAGCACGA
GCGGCACCCCATCTTCGGCAACATCGTGGACGAGGTGGCCTACCACGAGAAGTACCCCACCATCTA
CCACCTGAGAAAGAAACTGGTGGACAGCACCGACAAGGCCGACCTGCGGCTGATCTATCTGGCCCT
GGCCCACATGATCAAGTTCCGGGGCCACTTCCTGATCGAGGGCGACCTGAACCCCGACAACAGCGA
CGTGGACAAGCTGTTCATCCAGCTGGTGCAGACCTACAACCAGCTGTTCGAGGAAAACCCCATCAAC
GCCAGCGGCGTGGACGCCAAGGCCATCCTGTCTGCCAGACTGAGCAAGAGCAGACGGCTGGAAAA
TCTGATCGCCCAGCTGCCCGGCGAGAAGAAGAATGGCCTGTTCGGAAACCTGATTGCCCTGAGCCT
GGGCCTGACCCCCAACTTCAAGAGCAACTTCGACCTGGCCGAGGATGCCAAACTGCAGCTGAGCAA
GGACACCTACGACGACGACCTGGACAACCTGCTGGCCCAGATCGGCGACCAGTACGCCGACCTGTT
TCTGGCCGCCAAGAACCTGTCCGACGCCATCCTGCTGAGCGACATCCTGAGAGTGAACACCGAGAT
CACCAAGGCCCCCCTGAGCGCCTCTATGATCAAGAGATACGACGAGCACCACCAGGACCTGACCCT
GCTGAAAGCTCTCGTGCGGCAGCAGCTGCCTGAGAAGTACAAAGAGATTTTCTTCGACCAGAGCAAG
AACGGCTACGCCGGCTACATTGACGGCGGAGCCAGCCAGGAAGAGTTCTACAAGTTCATCAAGCCC
ATCCTGGAAAAGATGGACGGCACCGAGGAACTGCTCGTGAAGCTGAACAGAGAGGACCTGCTGCGG
AAGCAGCGGACCTTCGACAACGGCAGCATCCCCCACCAGATCCACCTGGGAGAGCTGCACGCCATT
CTGCGGCGGCAGGAAGATTTTTACCCATTCCTGAAGGACAACCGGGAAAAGATCGAGAAGATCCTGA
CCTTCCGCATCCCCTACTACGTGGGCCCTCTGGCCAGGGGAAACAGCAGATTCGCCTGGATGACCA
GAAAGAGCGAGGAAACCATCACCCCCTGGAACTTCGAGGAAGTGGTGGACAAGGGCGCTTCCGCC
CAGAGCTTCATCGAGCGGATGACCAACTTCGATAAGAACCTGCCCAACGAGAAGGTGCTGCCCAAG
CACAGCCTGCTGTACGAGTACTTCACCGTGTATAACGAGCTGACCAAAGTGAAATACGTGACCGAGG
GAATGAGAAAGCCCGCCTTCCTGAGCGGCGAGCAGAAAAAGGCCATCGTGGACCTGCTGTTCAAGA
CCAACCGGAAAGTGACCGTGAAGCAGCTGAAAGAGGACTACTTCAAGAAAATCGAGTGCTTCGACTC
CGTGGAAATCTCCGGCGTGGAAGATCGGTTCAACGCCTCCCTGGGCACATACCACGATCTGCTGAAA
ATTATCAAGGACAAGGACTTCCTGGACAATGAGGAAAACGAGGACATTCTGGAAGATATCGTGCTGAC
CCTGACACTGTTTGAGGACAGAGAGATGATCGAGGAACGGCTGAAAACCTATGCCCACCTGTTCGAC
GACAAAGTGATGAAGCAGCTGAAGCGGCGGAGATACACCGGCTGGGGCAGGCTGAGCCGGAAGCT
GATCAACGGCATCCGGGACAAGCAGTCCGGCAAGACAATCCTGGATTTCCTGAAGTCCGACGGCTT
CGCCAACAGAAACTTCATGCAGCTGATCCACGACGACAGCCTGACCTTTAAAGAGGACATCCAGAAA

GCCCAGGTGTCCGGCCAGGGCGATAGCCTGCACGAGCACATTGCCAATCTGGCCGGCAGCCCCGC
CATTAAGAAGGGCATCCTGCAGACAGTGAAGGTGGTGGACGAGCTCGTGAAAGTGATGGGCCGGCA
CAAGCCCGAGAACATCGTGATCGAAATGGCCAGAGAGAACCAGACCACCCAGAAGGGACAGAAGAA
CAGCCGCGAGAGAATGAAGCGGATCGAAGAGGGCATCAAAGAGCTGGGCAGCCAGATCCTGAAAGA
ACACCCCGTGGAAAACACCCAGCTGCAGAACGAGAAGCTGTACCTGTACTACCTGCAGAATGGGCG
GGATATGTACGTGGACCAGGAACTGGACATCAACCGGCTGTCCGACTACGATGTGGACCATATCGTG
CCTCAGAGCTTTCTGAAGGACGACTCCATCGACAACAAGGTGCTGACCAGAAGCGACAAGAACCGG
GGCAAGAGCGACAACGTGCCCTCCGAAGAGGTCGTGAAGAAGATGAAGAACTACTGGCGGCAGCT
GCTGAACGCCAAGCTGATTACCCAGAGAAAGTTCGACAATCTGACCAAGGCCGAGAGAGGCGGCCT
GAGCGAACTGGATAAGGCCGGCTTCATCAAGAGACAGCTGGTGGAAACCCGGCAGATCACAAAGCA
CGTGGCACAGATCCTGGACTCCCGGATGAACACTAAGTACGACGAGAATGACAAGCTGATCCGGGAA
GTGAAAGTGATCACCCTGAAGTCCAAGCTGGTGTCCGATTTCCGGAAGGATTTCCAGTTTTACAAAGT
GCGCGAGATCAACAACTACCACCACGCCCACGACGCCTACCTGAACGCCGTCGTGGGAACCGCCCT
GATCAAAAAGTACCCTAAGCTGGAAAGCGAGTTCGTGTACGGCGACTACAAGGTGTACGACGTGCGG
AAGATGATCGCCAAGAGCGAGCAGGAAATCGGCAAGGCTACCGCCAAGTACTTCTTCTACAGCAACA
TCATGAACTTTTTCAAGACCGAGATTACCCTGGCCAACGGCGAGATCCGGAAGCGGCCTCTGATCGA
GACAAACGGCGAAACCGGGGAGATCGTGTGGGATAAGGGCCGGGATTTTGCCACCGTGCGGAAAGT
GCTGAGCATGCCCCAAGTGAATATCGTGAAAAAGACCGAGGTGCAGACAGGCGGCTTCAGCAAAGA
GTCTATCAGACCCAAGAGGAACAGCGATAAGCTGATCGCCAGAAAGAAGGACTGGGACCCTAAGAAG
TACGGCGGCTTCCTGTGGCCCACCGTGGCCTATTCTGTGCTGGTGGTGGCCAAAGTGGAAAAGGGC
AAGTCCAAGAAACTGAAGAGTGTGAAAGAGCTGCTGGGGATCACCATCATGGAAAGAAGCAGCTTCG
AGAAGAATCCCATCGACTTTCTGGAAGCCAAGGGCTACAAAGAAGTGAAAAAGGACCTGATCATCAA
GCTGCCTAAGTACTCCCTGTTCGAGCTGGAAAACGGCCGGAAGAGAATGCTGGCCTCTGCCAAGCA
GCTGCAGAAGGGAAACGAACTGGCCCTGCCCTCCAAATATGTGAACTTCCTGTACCTGGCCAGCCAC
TATGAGAAGCTGAAGGGCTCCCCCGAGGATAATGAGCAGAAACAGCTGTTTGTGGAACAGCACAAGC
ACTACCTGGACGAGATCATCGAGCAGATCAGCGAGTTCTCCAAGAGAGTGATCCTGGCCGACGCTAA
TCTGGACAAAGTGCTGTCCGCCTACAACAAGCACCGGGATAAGCCCATCAGAGAGCAGGCCGAGAA
TATCATCCACCTGTTTACCCTGACCAGACTGGGAGCCCCTAGAGCCTTCAAGTACTTTGACACCACCA
TCGACCCCAAGCAGTACAGAAGCACCAAAGAGGTGCTGGACGCCACCCTGATCCACCAGAGCATCA
CCGGCCTGTACGAGACACGGATCGACCTGTCTCAGCTGGGAGGTGACAGCGGCGGGAGCGGCGG
GAGCGGGGGGAGCACTAATCTGAGCGACATCATTGAGAAGGAGACTGGGAAACAGCTGGTCATTCA
GGAGTCCATCCTGATGCTGCCTGAGGAGGTGGAGGAAGTGATCGGCAACAAGCCAGAGTCTGACAT
CCTGGTGCACACCGCCTACGACGAGTCCACAGATGAGAATGTGATGCTGCTGACCTCTGACGCCCC
CGAGTATAAGCCTTGGGCCCTGGTCATCCAGGATTCTAACGGCGAGAATAAGATCAAGATGCTGAGC
GGAGGATCCGGAGGATCTGGAGGCAGC*ACCAACCTGTCTGACATCATCGAGAAGGAGACAGGCAAG*
CAGCTGGTCATCCAGGAGAGCATCCTGATGCTGCCCGAAGAAGTCGAAGAAGTGATCGGAAACAAG
CCTGAGAGCGATATCCTGGTCCATACCGCCTACGACGAGAGTACCGACGAAAATGTGATGCTGCTGA
CATCCGACGCCCCAGAGTATAAGCCCTGGGCTCTGGTCATCCAGGATTCCAACGGAGAGAACAAAAT
*CAAAATGCTG***TCTGGCGGCTCAAAAAGAACCGCCGACGGCAGCGAATTCGAGCCCAAGAAGAAGA**
GGAAAGTCTAATTAATTAAGCTGCCTTCTGCGGGGCTTGCCTTCTGGCCATGCCCTTCTTCTCTCCCT
TGCACCTGTACCTCTTGGTCTTTGAATAAAGCCTGAGTAGGAAGCGACTGTGCCTTCTAGTTGCCAGC
CATCTGTTGTTTGCCCCTCCCCCGTGCCTTCCTTGACCCTGGAAGGTGCCACTCCCACTGTCCTTTC
CTAATAAAATGAGAAAATTGCATCGCATTGTCTGAGTAGGTGTCATTCTATTCTGGGGGGTGGGGTGG
GGCAGGACAGCAAGGGGGAGGATTGGGAAGACAATAGCAGGCATGCTGGGGATGCGGTGGGCTCT
ATGG

Start	End	Feature Description
1	380	CMV enhancer
381	619	CMV promoter
620	636	T7 promoter with mismatch
637	683	5’ UTR

684	740	BP NLS
741	1241	CBE6b deaminase
1242	1337	Linker
1338	5438	Cas9(D10A) Sp-RY PAM variant
5439	5468	Linker
5469	5717	uracil glycosylase inhibitor
5718	5747	Linker
5748	*5996*	*uracil glycosylase inhibitor*
**5997**	**6062**	**linker + SV40 BP NLS**
6063	6163	3' UTR
6164	6391	bGH poly(A) signal

Amino acid sequence
MKRTADGSEFESPKKKRKVSSEVEFSHEYWMRHALTLAKRARDEGEAPVGAVLVLNNRVIGEGWVRRIG
LHDPTAHAEIMALRQGGLVMQNPRLIDATLYVTFEPCVMCAGAMINSRIGRVVFGVRNSKRGAAGSLMNV
LNYPGMNHRVEITEGILADECAALLCDFYRMPRQVFNAQKKAQSSINSGGSSGGSSGSETPGTSESATP
ESSGGSSGGSDKKYSIGLAIGTNSVGWAVITDEYKVPSKKFKVLGNTDRHSIKKNLIGALLFDSGETAERT
RLKRTARRRYTRRKNRICYLQEIFSNEMAKVDDSFFHRLEESFLVEEDKKHERHPIFGNIVDEVAYHEKYP
TIYHLRKKLVDSTDKADLRLIYLALAHMIKFRGHFLIEGDLNPDNSDVDKLFIQLVQTYNQLFEENPINASGV
DAKAILSARLSKSRRLENLIAQLPGEKKNGLFGNLIALSLGLTPNFKSNFDLAEDAKLQLSKDTYDDDLDNL
LAQIGDQYADLFLAAKNLSDAILLSDILRVNTEITKAPLSASMIKRYDEHHQDLTLLKALVRQQLPEKYKEIFF
DQSKNGYAGYIDGGASQEEFYKFIKPILEKMDGTEELLVKLNREDLLRKQRTFDNGSIPHQIHLGELHAILR
RQEDFYPFLKDNREKIEKILTFRIPYYVGPLARGNSRFAWMTRKSEETITPWNFEEVVDKGASAQSFIERM
TNFDKNLPNEKVLPKHSLLYEYFTVYNELTKVKYVTEGMRKPAFLSGEQKKAIVDLLFKTNRKVTVKQLKE
DYFKKIECFDSVEISGVEDRFNASLGTYHDLLKIIKDKDFLDNEENEDILEDIVLTLTLFEDREMIEERLKTYA
HLFDDKVMKQLKRRRYTGWGRLSRKLINGIRDKQSGKTILDFLKSDGFANRNFMQLIHDDSLTFKEDIQKA
QVSGQGDSLHEHIANLAGSPAIKKGILQTVKVVDELVKVMGRHKPENIVIEMARENQTTQKGQKNSRERM
KRIEEGIKELGSQILKEHPVENTQLQNEKLYLYYLQNGRDMYVDQELDINRLSDYDVDHIVPQSFLKDDSID
NKVLTRSDKNRGKSDNVPSEEVVKKMKNYWRQLLNAKLITQRKFDNLTKAERGGLSELDKAGFIKRQLVE
TRQITKHVAQILDSRMNTKYDENDKLIREVKVITLKSKLVSDFRKDFQFYKVREINNYHHAHDAYLNAVVGT
ALIKKYPKLESEFVYGDYKVYDVRKMIAKSEQEIGKATAKYFFYSNIMNFFKTEITLANGEIRKRPLIETNGE
TGEIVWDKGRDFATVRKVLSMPQVNIVKKTEVQTGGFSKESIRPKRNSDKLIARKKDWDPKKYGGFLWPT
VAYSVLVVAKVEKGKSKKLKSVKELLGITIMERSSFEKNPIDFLEAKGYKEVKKDLIIKLPKYSLFELENGRK
RMLASAKQLQKGNELALPSKYVNFLYLASHYEKLKGSPEDNEQKQLFVEQHKHYLDEIIEQISEFSKRVILA
DANLDKVLSAYNKHRDKPIREQAENIIHLFTLTRLGAPRAFKYFDTTIDPKQYRSTKEVLDATLIHQSITGLYE
TRIDLSQLGGDSGGSGGSGGSTNLSDIIEKETGKQLVIQESILMLPEEVEEVIGNKPESDILVHTAYDESTDE
NVMLLTSDAPEYKPWALVIQDSNGENKIKMLSGGSGGSGGS*TNLSDIIEKETGKQLVIQESILMLPEEVEEV*
*IGNKPESDILVHTAYDESTDENVMLLTSDAPEYKPWALVIQDSNGENKIKML***SGGSKRTADGSEFEPKKK**
RKV

Start	End	Feature Description
1	19	BP NLS
20	186	TadCBEd deaminase
187	218	Linker

219	1585	Cas9(D10A) Sp-RY PAM variant
1586	1595	Linker
1596	1678	uracil glycosylase inhibitor
1679	1688	Linker
1689	*1771*	*uracil glycosylase inhibitor*
**1772**	**1792**	**linker + SV40 BP NLS**

BE4max – Cas9 (D10A) – SpNG variant – UGI
Plasmid ID: pHS0418

DNA sequence

GACATTGATTATTGACTAGTTATTAATAGTAATCAATTACGGGGTCATTAGTTCATAGCCCATATATGGAG
TTCCGCGTTACATAACTTACGGTAAATGGCCCGCCTGGCTGACCGCCCAACGACCCCCGCCCATTGA
CGTCAATAATGACGTATGTTCCCATAGTAACGCCAATAGGGACTTTCCATTGACGTCAATGGGTGGAGT
ATTTACGGTAAACTGCCCACTTGGCAGTACATCAAGTGTATCATATGCCAAGTACGCCCCCTATTGACG
TCAATGACGGTAAATGGCCCGCCTGGCATTATGCCCAGTACATGACCTTATGGGACTTTCCTACTTGG
CAGTACATCTACGTATTAGTCATCGCTATTACCATGGTGATGCGGTTTTGGCAGTACATCAATGGGCGT
GGATAGCGGTTTGACTCACGGGGATTTCCAAGTCTCCACCCCATTGACGTCAATGGGAGTTTGTTTTG
GCACCAAAATCAACGGGACTTTCCAAAATGTCGTAACAACTCCGCCCCATTGACGCAAATGGGCGGT
AGGCGTGTACGGTGGGAGGTCTATATAAGCAGAGCTGGTTTAGTGAACCGTCAGATCTCGAGCTCGG
TACCTAATACGACACACTATAAGGAAATAAGAGAGAAAAGAAGAGTAAGAAGAAATATAAGAGCCACCA
TGAAACGGACAGCCGACGGAAGCGAGTTCGAGTCACCAAAGAAGAAGCGGAAAGTCAGTTCAGAGA
CTGGGCCTGTCGCCGTCGATCCAACCCTGCGCCGCCGGATTGAACCTCACGAGTTTGAAGTGTTCTT
TGACCCCCGGGAGCTGAGAAAGGAGACATGCCTGCTGTACGAGATCAACTGGGGAGGCAGGCACTC
CATCTGGAGGCACACCTCTCAGAACACAAATAAGCACGTGGAGGTGAACTTCATCGAGAAGTTTACC
ACAGAGCGGTACTTCTGCCCCAATACCAGATGTAGCATCACATGGTTTCTGAGCTGGTCCCCTTGCG
GAGAGTGTAGCAGGGCCATCACCGAGTTCCTGTCCAGATATCCACACGTGACACTGTTTATCTACATC
GCCAGGCTGTATCACCACGCAGACCCAAGGAATAGGCAGGGCCTGCGCGATCTGATCAGCTCCGGC
GTGACCATCCAGATCATGACAGAGCAGGAGTCCGGCTACTGCTGGCGGAACTTCGTGAATTATTCTC
CTAGCAACGAGGCCCACTGGCCTAGGTACCCACACCTGTGGGTGCGCCTGTACGTGCTGGAGCTGT
ATTGCATCATCCTGGGCCTGCCCCCTTGTCTGAATATCCTGCGGAGAAAGCAGCCCCAGCTGACCTT
CTTTACAATCGCCCTGCAGTCTTGTCACTATCAGAGGCTGCCACCCCACATCCTGTGGGCCACAGGC
CTGAAGTCTGGCGGATCTAGCGGAGGATCCTCTGGCAGCGAGACACCAGGAACAAGCGAGTCAGCA
ACACCAGAGAGCAGTGGCGGCAGCAGCGGCGGCAGCGACAAGAAGTACAGCATCGGCCTGGCCAT
CGGCACCAACTCTGTGGGCTGGGCCGTGATCACCGACGAGTACAAGGTGCCCAGCAAGAAATTCAA
GGTGCTGGGCAACACCGACCGGCACAGCATCAAGAAGAACCTGATCGGAGCCCTGCTGTTCGACAG
CGGCGAAACAGCCGAGGCCACCCGGCTGAAGAGAACCGCCAGAAGAAGATACACCAGACGGAAGA
ACCGGATCTGCTATCTGCAAGAGATCTTCAGCAACGAGATGGCCAAGGTGGACGACAGCTTCTTCCA
CAGACTGGAAGAGTCCTTCCTGGTGGAAGAGGATAAGAAGCACGAGCGGCACCCCATCTTCGGCAA
CATCGTGGACGAGGTGGCCTACCACGAGAAGTACCCCACCATCTACCACCTGAGAAAGAAACTGGTG
GACAGCACCGACAAGGCCGACCTGCGGCTGATCTATCTGGCCCTGGCCCACATGATCAAGTTCCGG
GGCCACTTCCTGATCGAGGGCGACCTGAACCCCGACAACAGCGACGTGGACAAGCTGTTCATCCAG
CTGGTGCAGACCTACAACCAGCTGTTCGAGGAAAACCCCATCAACGCCAGCGGCGTGGACGCCAAG
GCCATCCTGTCTGCCAGACTGAGCAAGAGCAGACGGCTGGAAAATCTGATCGCCCAGCTGCCCGGC
GAGAAGAAGAATGGCCTGTTCGGAAACCTGATTGCCCTGAGCCTGGGCCTGACCCCCAACTTCAAG
AGCAACTTCGACCTGGCCGAGGATGCCAAACTGCAGCTGAGCAAGGACACCTACGACGACGACCTG
GACAACCTGCTGGCCCAGATCGGCGACCAGTACGCCGACCTGTTTCTGGCCGCCAAGAACCTGTCC
GACGCCATCCTGCTGAGCGACATCCTGAGAGTGAACACCGAGATCACCAAGGCCCCCCTGAGCGCC
TCTATGATCAAGAGATACGACGAGCACCACCAGGACCTGACCCTGCTGAAAGCTCTCGTGCGGCAGC
AGCTGCCTGAGAAGTACAAAGAGATTTTCTTCGACCAGAGCAAGAACGGCTACGCCGGCTACATTGA
CGGCGGAGCCAGCCAGGAAGAGTTCTACAAGTTCATCAAGCCCATCCTGGAAAAGATGGACGGCAC
CGAGGAACTGCTCGTGAAGCTGAACAGAGAGGACCTGCTGCGGAAGCAGCGGACCTTCGACAACG
GCAGCATCCCCCACCAGATCCACCTGGGAGAGCTGCACGCCATTCTGCGGCGGCAGGAAGATTTTT
ACCCATTCCTGAAGGACAACCGGGAAAAGATCGAGAAGATCCTGACCTTCCGCATCCCCTACTACGT
GGGCCCTCTGGCCAGGGGAAACAGCAGATTCGCCTGGATGACCAGAAAGAGCGAGGAAACCATCAC
CCCCTGGAACTTCGAGGAAGTGGTGGACAAGGGCGCTTCCGCCCAGAGCTTCATCGAGCGGATGAC
CAACTTCGATAAGAACCTGCCCAACGAGAAGGTGCTGCCCAAGCACAGCCTGCTGTACGAGTACTTC
ACCGTGTATAACGAGCTGACCAAAGTGAAATACGTGACCGAGGGAATGAGAAAGCCCGCCTTCCTGA
GCGGCGAGCAGAAAAAGGCCATCGTGGACCTGCTGTTCAAGACCAACCGGAAAGTGACCGTGAAGC
AGCTGAAAGAGGACTACTTCAAGAAAATCGAGTGCTTCGACTCCGTGGAAATCTCCGGCGTGGAAGA
TCGGTTCAACGCCTCCCTGGGCACATACCACGATCTGCTGAAAATTATCAAGGACAAGGACTTCCTG
GACAATGAGGAAAACGAGGACATTCTGGAAGATATCGTGCTGACCCTGACACTGTTTGAGGACAGAG
AGATGATCGAGGAACGGCTGAAAACCTATGCCCACCTGTTCGACGACAAAGTGATGAAGCAGCTGAA

GCGGCGGAGATACACCGGCTGGGGCAGGCTGAGCCGGAAGCTGATCAACGGCATCCGGGACAAGC
AGTCCGGCAAGACAATCCTGGATTTCCTGAAGTCCGACGGCTTCGCCAACAGAAACTTCATGCAGCT
GATCCACGACGACAGCCTGACCTTTAAAGAGGACATCCAGAAAGCCCAGGTGTCCGGCCAGGGCGA
TAGCCTGCACGAGCACATTGCCAATCTGGCCGGCAGCCCCGCCATTAAGAAGGGCATCCTGCAGAC
AGTGAAGGTGGTGGACGAGCTCGTGAAAGTGATGGGCCGGCACAAGCCCGAGAACATCGTGATCGA
AATGGCCAGAGAGAACCAGACCACCCAGAAGGGACAGAAGAACAGCCGCGAGAGAATGAAGCGGAT
CGAAGAGGGCATCAAAGAGCTGGGCAGCCAGATCCTGAAAGAACACCCCGTGGAAAACACCCAGCT
GCAGAACGAGAAGCTGTACCTGTACTACCTGCAGAATGGGCGGGATATGTACGTGGACCAGGAACTG
GACATCAACCGGCTGTCCGACTACGATGTGGACCATATCGTGCCTCAGAGCTTTCTGAAGGACGACT
CCATCGACAACAAGGTGCTGACCAGAAGCGACAAGAACCGGGGCAAGAGCGACAACGTGCCCTCC
GAAGAGGTCGTGAAGAAGATGAAGAACTACTGGCGGCAGCTGCTGAACGCCAAGCTGATTACCCAG
AGAAAGTTCGACAATCTGACCAAGGCCGAGAGAGGCGGCCTGAGCGAACTGGATAAGGCCGGCTTC
ATCAAGAGACAGCTGGTGGAAACCCGGCAGATCACAAAGCACGTGGCACAGATCCTGGACTCCCGG
ATGAACACTAAGTACGACGAGAATGACAAGCTGATCCGGGAAGTGAAAGTGATCACCCTGAAGTCCA
AGCTGGTGTCCGATTTCCGGAAGGATTTCCAGTTTTACAAAGTGCGCGAGATCAACAACTACCACCAC
GCCCACGACGCCTACCTGAACGCCGTCGTGGGAACCGCCCTGATCAAAAAGTACCCTAAGCTGGAA
AGCGAGTTCGTGTACGGCGACTACAAGGTGTACGACGTGCGGAAGATGATCGCCAAGAGCGAGCAG
GAAATCGGCAAGGCTACCGCCAAGTACTTCTTCTACAGCAACATCATGAACTTTTTCAAGACCGAGAT
TACCCTGGCCAACGGCGAGATCCGGAAGCGGCCTCTGATCGAGACAAACGGCGAAACCGGGGAGA
TCGTGTGGGATAAGGGCCGGGATTTTGCCACCGTGCGGAAAGTGCTGAGCATGCCCCAAGTGAATAT
CGTGAAAAAGACCGAGGTGCAGACAGGCGGCTTCAGCAAAGAGTCTATCAGGCCCAAGAGGAACAG
CGATAAGCTGATCGCCAGAAAGAAGGACTGGGACCCTAAGAAGTACGGCGGCTTCGTCAGCCCCAC
CGTGGCCTATTCTGTGCTGGTGGTGGCCAAAGTGGAAAAGGGCAAGTCCAAGAAACTGAAGAGTGT
GAAAGAGCTGCTGGGGATCACCATCATGGAAAGAAGCAGCTTCGAGAAGAATCCCATCGACTTTCTG
GAAGCCAAGGGCTACAAAGAAGTGAAAAAGGACCTGATCATCAAGCTGCCTAAGTACTCCCTGTTCG
AGCTGGAAAACGGCCGGAAGAGAATGCTGGCCTCTGCCAGATTCCTGCAGAAGGGAAACGAACTGG
CCCTGCCCTCCAAATATGTGAACTTCCTGTACCTGGCCAGCCACTATGAGAAGCTGAAGGGCTCCCC
CGAGGATAATGAGCAGAAACAGCTGTTTGTGGAACAGCACAAGCACTACCTGGACGAGATCATCGAG
CAGATCAGCGAGTTCTCCAAGAGAGTGATCCTGGCCGACGCTAATCTGGACAAAGTGCTGTCCGCCT
ACAACAAGCACCGGGATAAGCCCATCAGAGAGCAGGCCGAGAATATCATCCACCTGTTTACCCTGAC
CAATCTGGGAGCCCCTAGGGCCTTCAAGTACTTTGACACCACCATCGACCGGAAGGTGTACAGGAGC
ACCAAAGAGGTGCTGGACGCCACCCTGATCCACCAGAGCATCACCGGCCTGTACGAGACACGGATC
GACCTGTCTCAGCTGGGAGGTGACAGCGGCGGGAGCGGCGGGAGCGGGGGGAGCACTAATCTGA
GCGACATCATTGAGAAGGAGACTGGGAAACAGCTGGTCATTCAGGAGTCCATCCTGATGCTGCCTGA
GGAGGTGGAGGAAGTGATCGGCAACAAGCCAGAGTCTGACATCCTGGTGCACACCGCCTACGACGA
GTCCACAGATGAGAATGTGATGCTGCTGACCTCTGACGCCCCCGAGTATAAGCCTTGGGCCCTGGTC
ATCCAGGATTCTAACGGCGAGAATAAGATCAAGATGCTGAGCGGAGGATCCGGAGGATCTGGAGGCA
GC*ACCAACCTGTCTGACATCATCGAGAAGGAGACAGGCAAGCAGCTGGTCATCCAGGAGAGCATCC*
TGATGCTGCCCGAAGAAGTCGAAGAAGTGATCGGAAACAAGCCTGAGAGCGATATCCTGGTCCATAC
CGCCTACGACGAGAGTACCGACGAAAATGTGATGCTGCTGACATCCGACGCCCCAGAGTATAAGCCC
*TGGGCTCTGGTCATCCAGGATTCCAACGGAGAGAACAAAATCAAAATGCTG***TCTGGCGGCTCAAAAA**
GAACCGCCGACGGCAGCGAATTCGAGCCCAAGAAGAAGAGGAAAGTCTAATTAATTAAGCTGCCTT
CTGCGGGGCTTGCCTTCTGGCCATGCCCTTCTTCTCTCCCTTGCACCTGTACCTCTTGGTCTTTGAAT
AAAGCCTGAGTAGGAAGCGACTGTGCCTTCTAGTTGCCAGCCATCTGTTGTTTGCCCCTCCCCCGTG
CCTTCCTTGACCCTGGAAGGTGCCACTCCCACTGTCCTTTCCTAATAAAATGAGAAATTGCATCGCA
TTGTCTGAGTAGGTGTCATTCTATTCTGGGGGGTGGGGTGGGGCAGGACAGCAAGGGGGAGGATTG
GGAAGACAATAGCAGGCATGCTGGGGATGCGGTGGGCTCTATGG

Start	End	Feature Description
1	380	CMV enhancer
381	619	CMV promoter
620	636	T7 promoter with mismatch

637	683	5’ UTR
684	740	BP NLS
741	1424	Engineered BE4max deaminase
1425	1520	BE4max linker
1521	5621	Cas9(D10A) Sp-NG PAM variant
5622	5651	Linker
5652	5900	uracil glycosylase inhibitor
5901	5930	Linker
5931	*6179*	*uracil glycosylase inhibitor*
**6180**	**6245**	**linker + SV40 BP NLS**
6246	6346	3’ UTR
6347	6574	bGH poly(A) signal

Amino acid sequence

MKRTADGSEFESPKKKRKVSSETGPVAVDPTLRRRIEPHEFEVFFDPRELRKETCLLYEINWGGRHSIWR
HTSQNTNKHVEVNFIEKFTTERYFCPNTRCSITWFLSWSPCGECSRAITEFLSRYPHVTLFIYIARLYHHAD
PRNRQGLRDLISSGVTIQIMTEQESGYCWRNFVNYSPSNEAHWPRYPHLWVRLYVLELYCIILGLPPCLNI
LRRKQPQLTFFTIALQSCHYQRLPPHILWATGLKSGGSSGGSSGSETPGTSESATPESSGGSSGGSDKKY
SIGLAIGTNSVGWAVITDEYKVPSKKFKVLGNTDRHSIKKNLIGALLFDSGETAEATRLKRTARRRYTRRKN
RICYLQEIFSNEMAKVDDSFFHRLEESFLVEEDKKHERHPIFGNIVDEVAYHEKYPTIYHLRKKLVDSTDKA
DLRLIYLALAHMIKFRGHFLIEGDLNPDNSDVDKLFIQLVQTYNQLFEENPINASGVDAKAILSARLSKSRRL
ENLIAQLPGEKKNGLFGNLIALSLGLTPNFKSNFDLAEDAKLQLSKDTYDDDLDNLLAQIGDQYADLFLAAK
NLSDAILLSDILRVNTEITKAPLSASMIKRYDEHHQDLTLLKALVRQQLPEKYKEIFFDQSKNGYAGYIDGGA
SQEEFYKFIKPILEKMDGTEELLVKLNREDLLRKQRTFDNGSIPHQIHLGELHAILRRQEDFYPFLKDNREKI
EKILTFRIPYYVGPLARGNSRFAWMTRKSEETITPWNFEEVVDKGASAQSFIERMTNFDKNLPNEKVLPKH
SLLYEYFTVYNELTKVKYVTEGMRKPAFLSGEQKKAIVDLLFKTNRKVTVKQLKEDYFKKIECFDSVEISGV
EDRFNASLGTYHDLLKIIKDKDFLDNEENEDILEDIVLTLTLFEDREMIEERLKTYAHLFDDKVMKQLKRRRY
TGWGRLSRKLINGIRDKQSGKTILDFLKSDGFANRNFMQLIHDDSLTFKEDIQKAQVSGQGDSLHEHIANL
AGSPAIKKGILQTVKVVDELVKVMGRHKPENIVIEMARENQTTQKGQKNSRERMKRIEEGIKELGSQILKE
HPVENTQLQNEKLYLYYLQNGRDMYVDQELDINRLSDYDVDHIVPQSFLKDDSIDNKVLTRSDKNRGKSD
NVPSEEVVKKMKNYWRQLLNAKLITQRKFDNLTKAERGGLSELDKAGFIKRQLVETRQITKHVAQILDSRM
NTKYDENDKLIREVKVITLKSKLVSDFRKDFQFYKVREINNYHHAHDAYLNAVVGTALIKKYPKLESEFVYG
DYKVYDVRKMIAKSEQEIGKATAKYFFYSNIMNFFKTEITLANGEIRKRPLIETNGETGEIVWDKGRDFATV
RKVLSMPQVNIVKKTEVQTGGFSKESIRPKRNSDKLIARKKDWDPKKYGGFVSPTVAYSVLVVAKVEKGK
SKKLKSVKELLGITIMERSSFEKNPIDFLEAKGYKEVKKDLIIKLPKYSLFELENGRKRMLASARFLQKGNEL
ALPSKYVNFLYLASHYEKLKGSPEDNEQKQLFVEQHKHYLDEIIEQISEFSKRVILADANLDKVLSAYNKHR
DKPIREQAENIIHLFTLTNLGAPRAFKYFDTTIDRKVYRSTKEVLDATLIHQSITGLYETRIDLSQLGGDSGGS
GGSGGSTNLSDIIEKETGKQLVIQESILMLPEEVEEVIGNKPESDILVHTAYDESTDENVMLLTSDAPEYKP
WALVIQDSNGENKIKMLSGGSGGSGGS*TNLSDIIEKETGKQLVIQESILMLPEEVEEVIGNKPESDILVHTAY
DESTDENVMLLTSDAPEYKPWALVIQDSNGENKIKML***SGGSKRTADGSEFEPKKKRKV**.

Start	End	Feature Description
1	19	BP NLS
20	247	Engineered BE4max deaminase

248	279	BE4max linker
280	1646	Cas9(D10A) Sp-NG PAM variant
1647	1656	Linker
1657	1739	uracil glycosylase inhibitor
1740	1749	Linker
1750	*1832*	*uracil glycosylase inhibitor*
**1833**	**1853**	**linker + SV40 BP NLS**

YE1-BE3 – Cas9 (D10A) – SpNG variant – UGI
Plasmid ID: pHS0417

DNA sequence
GACATTGATTATTGACTAGTTATTAATAGTAATCAATTACGGGGTCATTAGTTCATAGCCCATATATGGAG
TTCCGCGTTACATAACTTACGGTAAATGGCCCGCCTGGCTGACCGCCCAACGACCCCCGCCCATTGA
CGTCAATAATGACGTATGTTCCCATAGTAACGCCAATAGGGACTTTCCATTGACGTCAATGGGTGGAGT
ATTTACGGTAAACTGCCCACTTGGCAGTACATCAAGTGTATCATATGCCAAGTACGCCCCCTATTGACG
TCAATGACGGTAAATGGCCCGCCTGGCATTATGCCCAGTACATGACCTTATGGGACTTTCCTACTTGG
CAGTACATCTACGTATTAGTCATCGCTATTACCATGGTGATGCGGTTTTGGCAGTACATCAATGGGCGT
GGATAGCGGTTTGACTCACGGGGATTTCCAAGTCTCCACCCCATTGACGTCAATGGGAGTTTGTTTTG
GCACCAAAATCAACGGGACTTTCCAAAATGTCGTAACAACTCCGCCCCATTGACGCAAATGGGCGGT
AGGCGTGTACGGTGGGAGGTCTATATAAGCAGAGCTGGTTTAGTGAACCGTCAGATCTCGAGCTCGG
TACCTAATACGACACACTATAAGGAAATAAGAGAGAAAAGAAGAGTAAGAAGAAATATAAGAGCCACCA
TGAAACGGACAGCCGACGGAAGCGAGTTCGAGTCACCAAAGAAGAAGCGGAAAGTCTCCTCAGAGA
CTGGGCCTGTCGCCGTCGATCCAACCCTGCGCCGCCGGATTGAACCTCACGAGTTTGAAGTGTTCTT
TGACCCCCGGGAGCTGAGAAAGGAGACATGCCTGCTGTACGAGATCAACTGGGGAGGCAGGCACTC
CATCTGGAGGCACACCTCTCAGAACACAAATAAGCACGTGGAGGTGAACTTCATCGAGAAGTTTACC
ACAGAGCGGTACTTCTGCCCCAATACCAGATGTAGCATCACATGGTTTCTGAGCTATTCCCCTTGCGG
AGAGTGTAGCAGGGCCATCACCGAGTTCCTGTCCAGATATCCACACGTGACACTGTTTATCTACATCG
CCAGGCTGTATCACCACGCAGACCCAGAGAATAGGCAGGGCCTGCGCGATCTGATCAGCTCCGGCG
TGACCATCCAGATCATGACAGAGCAGGAGTCCGGCTACTGCTGGCGGAACTTCGTGAATTATTCTCCT
AGCAACGAGGCCCACTGGCCTAGGTACCCACACCTGTGGGTGCGCCTGTACGTGCTGGAGCTGTAT
TGCATCATCCTGGGCCTGCCCCCTTGTCTGAATATCCTGCGGAGAAAGCAGCCCCAGCTGACCTTCT
TTACAATCGCCCTGCAGTCTTGTCACTATCAGAGGCTGCCACCCCACATCCTGTGGGCCACAGGCCT
GAAGTCTGGAGGATCTAGCGGAGGATCCTCTGGCAGCGAGACACCAGGAACAAGCGAGTCAGCAAC
ACCAGAGAGCAGTGGCGGCAGCAGCGGCGGCAGCGACAAGAAGTACAGCATCGGCCTGGCCATCG
GCACCAACTCTGTGGGCTGGGCCGTGATCACCGACGAGTACAAGGTGCCCAGCAAGAAATTCAAGG
TGCTGGGCAACACCGACCGGCACAGCATCAAGAAGAACCTGATCGGAGCCCTGCTGTTCGACAGCG
GCGAAACAGCCGAGGCCACCCGGCTGAAGAGAACCGCCAGAAGAAGATACACCAGACGGAAGAAC
CGGATCTGCTATCTGCAAGAGATCTTCAGCAACGAGATGGCCAAGGTGGACGACAGCTTCTTCCACA
GACTGGAAGAGTCCTTCCTGGTGGAAGAGGATAAGAAGCACGAGCGGCACCCCATCTTCGGCAACA
TCGTGGACGAGGTGGCCTACCACGAGAAGTACCCCACCATCTACCACCTGAGAAAGAAACTGGTGG
ACAGCACCGACAAGGCCGACCTGCGGCTGATCTATCTGGCCCTGGCCCACATGATCAAGTTCCGGG
GCCACTTCCTGATCGAGGGCGACCTGAACCCCGACAACAGCGACGTGGACAAGCTGTTCATCCAGC
TGGTGCAGACCTACAACCAGCTGTTCGAGGAAAACCCCATCAACGCCAGCGGCGTGGACGCCAAGG
CCATCCTGTCTGCCAGACTGAGCAAGAGCAGACGGCTGGAAAATCTGATCGCCCAGCTGCCCGGCG
AGAAGAAGAATGGCCTGTTCGGAAACCTGATTGCCCTGAGCCTGGGCCTGACCCCCAACTTCAAGA
GCAACTTCGACCTGGCCGAGGATGCCAAACTGCAGCTGAGCAAGGACACCTACGACGACGACCTGG
ACAACCTGCTGGCCCAGATCGGCGACCAGTACGCCGACCTGTTTCTGGCCGCCAAGAACCTGTCCG
ACGCCATCCTGCTGAGCGACATCCTGAGAGTGAACACCGAGATCACCAAGGCCCCCCTGAGCGCCT
CTATGATCAAGAGATACGACGAGCACCACCAGGACCTGACCCTGCTGAAAGCTCTCGTGCGGCAGCA
GCTGCCTGAGAAGTACAAAGAGATTTTCTTCGACCAGAGCAAGAACGGCTACGCCGGCTACATTGAC
GGCGGAGCCAGCCAGGAAGAGTTCTACAAGTTCATCAAGCCCATCCTGGAAAAGATGGACGGCACC
GAGGAACTGCTCGTGAAGCTGAACAGAGAGGACCTGCTGCGGAAGCAGCGGACCTTCGACAACGG
CAGCATCCCCCACCAGATCCACCTGGGAGAGCTGCACGCCATTCTGCGGCGGCAGGAAGATTTTTAC
CCATTCCTGAAGGACAACCGGGAAAAGATCGAGAAGATCCTGACCTTCCGCATCCCCTACTACGTGG
GCCCTCTGGCCAGGGGAAACAGCAGATTCGCCTGGATGACCAGAAAGAGCGAGGAAACCATCACCC
CCTGGAACTTCGAGGAAGTGGTGGACAAGGGCGCTTCCGCCCAGAGCTTCATCGAGCGGATGACCA
ACTTCGATAAGAACCTGCCCAACGAGAAGGTGCTGCCCAAGCACAGCCTGCTGTACGAGTACTTCAC
CGTGTATAACGAGCTGACCAAAGTGAAATACGTGACCGAGGGAATGAGAAAGCCCGCCTTCCTGAGC
GGCGAGCAGAAAAAGGCCATCGTGGACCTGCTGTTCAAGACCAACCGGAAAGTGACCGTGAAGCAG
CTGAAAGAGGACTACTTCAAGAAAATCGAGTGCTTCGACTCCGTGGAAATCTCCGGCGTGGAAGATC
GGTTCAACGCCTCCCTGGGCACATACCACGATCTGCTGAAAATTATCAAGGACAAGGACTTCCTGGA
CAATGAGGAAAACGAGGACATTCTGGAAGATATCGTGCTGACCCTGACACTGTTTGAGGACAGAGAG
ATGATCGAGGAACGGCTGAAAACCTATGCCCACCTGTTCGACGACAAAGTGATGAAGCAGCTGAAGC

GGCGGAGATACACCGGCTGGGGCAGGCTGAGCCGGAAGCTGATCAACGGCATCCGGGACAAGCAG
TCCGGCAAGACAATCCTGGATTTCCTGAAGTCCGACGGCTTCGCCAACAGAAACTTCATGCAGCTGA
TCCACGACGACAGCCTGACCTTTAAAGAGGACATCCAGAAAGCCCAGGTGTCCGGCCAGGGCGATA
GCCTGCACGAGCACATTGCCAATCTGGCCGGCAGCCCCGCCATTAAGAAGGGCATCCTGCAGACAG
TGAAGGTGGTGGACGAGCTCGTGAAAGTGATGGGCCGGCACAAGCCCGAGAACATCGTGATCGAAA
TGGCCAGAGAGAACCAGACCACCCAGAAGGGACAGAAGAACAGCCGCGAGAGAATGAAGCGGATC
GAAGAGGGCATCAAAGAGCTGGGCAGCCAGATCCTGAAAGAACACCCCGTGGAAAACACCCAGCTG
CAGAACGAGAAGCTGTACCTGTACTACCTGCAGAATGGGCGGGATATGTACGTGGACCAGGAACTGG
ACATCAACCGGCTGTCCGACTACGATGTGGACCATATCGTGCCTCAGAGCTTTCTGAAGGACGACTC
CATCGACAACAAGGTGCTGACCAGAAGCGACAAGAACCGGGGCAAGAGCGACAACGTGCCCTCCG
AAGAGGTCGTGAAGAAGATGAAGAACTACTGGCGGCAGCTGCTGAACGCCAAGCTGATTACCCAGA
GAAAGTTCGACAATCTGACCAAGGCCGAGAGAGGCGGCCTGAGCGAACTGGATAAGGCCGGCTTCA
TCAAGAGACAGCTGGTGGAAACCCGGCAGATCACAAAGCACGTGGCACAGATCCTGGACTCCCGGA
TGAACACTAAGTACGACGAGAATGACAAGCTGATCCGGGAAGTGAAAGTGATCACCCTGAAGTCCAA
GCTGGTGTCCGATTTCCGGAAGGATTTCCAGTTTTACAAAGTGCGCGAGATCAACAACTACCACCAC
GCCCACGACGCCTACCTGAACGCCGTCGTGGGAACCGCCCTGATCAAAAAGTACCCTAAGCTGGAA
AGCGAGTTCGTGTACGGCGACTACAAGGTGTACGACGTGCGGAAGATGATCGCCAAGAGCGAGCAG
GAAATCGGCAAGGCTACCGCCAAGTACTTCTTCTACAGCAACATCATGAACTTTTTCAAGACCGAGAT
TACCCTGGCCAACGGCGAGATCCGGAAGCGGCCTCTGATCGAGACAAACGGCGAAACCGGGGAGA
TCGTGTGGGATAAGGGCCGGGATTTTGCCACCGTGCGGAAAGTGCTGAGCATGCCCCAAGTGAATAT
CGTGAAAAAGACCGAGGTGCAGACAGGCGGCTTCAGCAAAGAGTCTATCAGGCCCAAGAGGAACAG
CGATAAGCTGATCGCCAGAAAGAAGGACTGGGACCCTAAGAAGTACGGCGGCTTCGTCAGCCCCAC
CGTGGCCTATTCTGTGCTGGTGGTGGCCAAAGTGGAAAAGGGCAAGTCCAAGAAACTGAAGAGTGT
GAAAGAGCTGCTGGGGATCACCATCATGGAAAGAAGCAGCTTCGAGAAGAATCCCATCGACTTTCTG
GAAGCCAAGGGCTACAAAGAAGTGAAAAAGGACCTGATCATCAAGCTGCCTAAGTACTCCCTGTTCG
AGCTGGAAAACGGCCGGAAGAGAATGCTGGCCTCTGCCAGATTCCTGCAGAAGGGAAACGAACTGG
CCCTGCCCTCCAAATATGTGAACTTCCTGTACCTGGCCAGCCACTATGAGAAGCTGAAGGGCTCCCC
CGAGGATAATGAGCAGAAACAGCTGTTTGTGGAACAGCACAAGCACTACCTGGACGAGATCATCGAG
CAGATCAGCGAGTTCTCCAAGAGAGTGATCCTGGCCGACGCTAATCTGGACAAAGTGCTGTCCGCCT
ACAACAAGCACCGGGATAAGCCCATCAGAGAGCAGGCCGAGAATATCATCCACCTGTTTACCCTGAC
CAATCTGGGAGCCCCTAGGGCCTTCAAGTACTTTGACACCACCATCGACCGGAAGGTGTACAGGAGC
ACCAAAGAGGTGCTGGACGCCACCCTGATCCACCAGAGCATCACCGGCCTGTACGAGACACGGATC
GACCTGTCTCAGCTGGGAGGTGACAGCGGCGGGAGCGGCGGGAGCGGGGGGAGCACTAATCTGA
GCGACATCATTGAGAAGGAGACTGGGAAACAGCTGGTCATTCAGGAGTCCATCCTGATGCTGCCTGA
GGAGGTGGAGGAAGTGATCGGCAACAAGCCAGAGTCTGACATCCTGGTGCACACCGCCTACGACGA
GTCCACAGATGAGAATGTGATGCTGCTGACCTCTGACGCCCCCGAGTATAAGCCTTGGGCCCTGGTC
ATCCAGGATTCTAACGGCGAGAATAAGATCAAGATGCTGAGCGGAGGATCCGGAGGATCTGGAGGCA
GC*ACCAACCTGTCTGACATCATCGAGAAGGAGACAGGCAAGCAGCTGGTCATCCAGGAGAGCATCC*
TGATGCTGCCCGAAGAAGTCGAAGAAGTGATCGGAAACAAGCCTGAGAGCGATATCCTGGTCCATAC
CGCCTACGACGAGAGTACCGACGAAAATGTGATGCTGCTGACATCCGACGCCCCAGAGTATAAGCCC
*TGGGCTCTGGTCATCCAGGATTCCAACGGAGAGAACAAAATCAAAATGCTG***TCTGGCGGCTCAAAAA**
GAACCGCCGACGGCAGCGAATTCGAGCCCAAGAAGAAGAGGAAAGTCTAATTAATTAAGCTGCCTT
CTGCGGGGCTTGCCTTCTGGCCATGCCCTTCTTCTCTCCCTTGCACCTGTACCTCTTGGTCTTTGAAT
AAAGCCTGAGTAGGAAGCGACTGTGCCTTCTAGTTGCCAGCCATCTGTTGTTTGCCCCTCCCCCGTG
CCTTCCTTGACCCTGGAAGGTGCCACTCCCACTGTCCTTTCCTAATAAAATGAGAAATTGCATCGCA
TTGTCTGAGTAGGTGTCATTCTATTCTGGGGGGTGGGGTGGGGCAGGACAGCAAGGGGGAGGATTG
GGAAGACAATAGCAGGCATGCTGGGGATGCGGTGGGCTCTATGG

Start	End	Feature Description
1	380	CMV enhancer
381	619	CMV promoter
620	636	T7 promoter with mismatch

637	683	5' UTR
684	740	BP NLS
741	1424	YE1 deaminase
1425	1520	BE4max linker
1521	5621	Cas9(D10A) Sp-NG PAM variant
5622	5651	Linker
5652	5900	uracil glycosylase inhibitor
5901	5930	Linker
5931	*6179*	*uracil glycosylase inhibitor*
**6180**	**6245**	**linker + SV40 BP NLS**
6246	6346	3' UTR
6347	6574	bGH poly(A) signal

Amino acid sequence

MKRTADGSEFESPKKKRKVSSETGPVAVDPTLRRRIEPHEFEVFFDPRELRKETCLLYEINWGGRHSIWR
HTSQNTNKHVEVNFIEKFTTERYFCPNTRCSITWFLSYSPCGECSRAITEFLSRYPHVTLFIYIARLYHHAD
PENRQGLRDLISSGVTIQIMTEQESGYCWRNFVNYSPSNEAHWPRYPHLWVRLYVLELYCIILGLPPCLNI
LRRKQPQLTFFTIALQSCHYQRLPPHILWATGLKSGGSSGGSSGSETPGTSESATPESSGGSSGGSDKKY
SIGLAIGTNSVGWAVITDEYKVPSKKFKVLGNTDRHSIKKNLIGALLFDSGETAEATRLKRTARRRYTRRKN
RICYLQEIFSNEMAKVDDSFFHRLEESFLVEEDKKHERHPIFGNIVDEVAYHEKYPTIYHLRKKLVDSTDKA
DLRLIYLALAHMIKFRGHFLIEGDLNPDNSDVDKLFIQLVQTYNQLFEENPINASGVDAKAILSARLSKSRRL
ENLIAQLPGEKKNGLFGNLIALSLGLTPNFKSNFDLAEDAKLQLSKDTYDDDLDNLLAQIGDQYADLFLAAK
NLSDAILLSDILRVNTEITKAPLSASMIKRYDEHHQDLTLLKALVRQQLPEKYKEIFFDQSKNGYAGYIDGGA
SQEEFYKFIKPILEKMDGTEELLVKLNREDLLRKQRTFDNGSIPHQIHLGELHAILRRQEDFYPFLKDNREKI
EKILTFRIPYYVGPLARGNSRFAWMTRKSEETITPWNFEEVVDKGASAQSFIERMTNFDKNLPNEKVLPKH
SLLYEYFTVYNELTKVKYVTEGMRKPAFLSGEQKKAIVDLLFKTNRKVTVKQLKEDYFKKIECFDSVEISGV
EDRFNASLGTYHDLLKIIKDKDFLDNEENEDILEDIVLTLTLFEDREMIEERLKTYAHLFDDKVMKQLKRRRY
TGWGRLSRKLINGIRDKQSGKTILDFLKSDGFANRNFMQLIHDDSLTFKEDIQKAQVSGQGDSLHEHIANL
AGSPAIKKGILQTVKVVDELVKVMGRHKPENIVIEMARENQTTQKGQKNSRERMKRIEEGIKELGSQILKE
HPVENTQLQNEKLYLYYLQNGRDMYVDQELDINRLSDYDVDHIVPQSFLKDDSIDNKVLTRSDKNRGKSD
NVPSEEVVKKMKNYWRQLLNAKLITQRKFDNLTKAERGGLSELDKAGFIKRQLVETRQITKHVAQILDSRM
NTKYDENDKLIREVKVITLKSKLVSDFRKDFQFYKVREINNYHHAHDAYLNAVVGTALIKKYPKLESEFVYG
DYKVYDVRKMIAKSEQEIGKATAKYFFYSNIMNFFKTEITLANGEIRKRPLIETNGETGEIVWDKGRDFATV
RKVLSMPQVNIVKKTEVQTGGFSKESIRPKRNSDKLIARKKDWDPKKYGGFVSPTVAYSVLVVAKVEKGK
SKKLKSVKELLGITIMERSSFEKNPIDFLEAKGYKEVKKDLIIKLPKYSLFELENGRKRMLASARFLQKGNEL
ALPSKYVNFLYLASHYEKLKGSPEDNEQKQLFVEQHKHYLDEIIEQISEFSKRVILADANLDKVLSAYNKHR
DKPIREQAENIIHLFTLTNLGAPRAFKYFDTTIDRKVYRSTKEVLDATLIHQSITGLYETRIDLSQLGGDSGGS
GGSGGSTNLSDIIEKETGKQLVIQESILMLPEEVEEVIGNKPESDILVHTAYDESTDENVMLLTSDAPEYKP
WALVIQDSNGENKIKMLSGGSGGSGGS*TNLSDIIEKETGKQLVIQESILMLPEEVEEVIGNKPESDILVHTAY*
*DESTDENVMLLTSDAPEYKPWALVIQDSNGENKIKML***SGGSKRTADGSEFEPKKKRKV**.

Start	End	Feature Description
1	19	BP NLS
20	247	YE1 deaminase

248	279	BE4max linker
280	1646	Cas9(D10A) Sp-NG PAM variant
1647	1656	Linker
1657	1739	uracil glycosylase inhibitor
1740	1749	Linker
1750	*1832*	*uracil glycosylase inhibitor*
**1833**	**1853**	**linker + SV40 BP NLS**

TadCBEd – Cas9 (D10A) – SpNG variant – UGI
Plasmid ID: pHS0419

DNA sequence
GACATTGATTATTGACTAGTTATTAATAGTAATCAATTACGGGGTCATTAGTTCATAGCCCATATATGGAG
TTCCGCGTTACATAACTTACGGTAAATGGCCCGCCTGGCTGACCGCCCAACGACCCCCGCCCATTGA
CGTCAATAATGACGTATGTTCCCATAGTAACGCCAATAGGGACTTTCCATTGACGTCAATGGGTGGAGT
ATTTACGGTAAACTGCCCACTTGGCAGTACATCAAGTGTATCATATGCCAAGTACGCCCCCTATTGACG
TCAATGACGGTAAATGGCCCGCCTGGCATTATGCCCAGTACATGACCTTATGGGACTTTCCTACTTGG
CAGTACATCTACGTATTAGTCATCGCTATTACCATGGTGATGCGGTTTTGGCAGTACATCAATGGGCGT
GGATAGCGGTTTGACTCACGGGGATTTCCAAGTCTCCACCCCATTGACGTCAATGGGAGTTTGTTTTG
GCACCAAAATCAACGGGACTTTCCAAAATGTCGTAACAACTCCGCCCCATTGACGCAAATGGGCGGT
AGGCGTGTACGGTGGGAGGTCTATATAAGCAGAGCTGGTTTAGTGAACCGTCAGATCTCGAGCTCGG
TACCTAATACGACACACTATAAGGAAATAAGAGAGAAAAGAAGAGTAAGAAGAAATATAAGAGCCACCA
TGAAACGGACAGCCGACGGAAGCGAGTTCGAGTCACCAAAGAAGAAGCGGAAAGTCAGTTCTGAGG
TGGAGTTTTCCCACGAGTACTGGATGAGACATGCCCTGACCCTGGCCAAGAGGGCACGGGATGAGA
GGAAGGCGCCTGTGGGAGCCGTGCTGGTGCTGAACAATAGAGTGATCGGCGAGGGCTGGAACAGA
GCCATCGGCCTGCACGACCCAACAGCCCATGCCGAAATTATAGCCCTGAGACAGGGCGGCCTGGTC
ATGCAGAACTACAGACTGATTGACGCCACCCTGTACGTGACATTCGAGCCTTGCGTGATGTGCGCCG
GCGCCATGATCAACTCTAGGATCGGCCGCGTGGTGTTTGGCGTGAGGAACTCAAAAAGAGGCGCCG
CAGGCTCCCTGATGAACGTGCTGAACTACCCCGGAATGAATCACCGCGTCGAAATTACCGAGGGAAT
CCTGGCAGATGAATGTGCCGCCCTGCTGTGCGATTTCTATCGGATGCCTAGACAGGTGTTCAATGCTC
AGAAGAAGGCCCAGAGCTCCATCAACTCTGGCGGATCTAGCGGAGGATCCTCTGGCAGCGAGACAC
CAGGAACAAGCGAGTCAGCAACACCAGAGAGCAGTGGCGGCAGCAGCGGCGGCAGCGACAAGAA
GTACAGCATCGGCCTGGCCATCGGCACCAACTCTGTGGGCTGGGCCGTGATCACCGACGAGTACAA
GGTGCCCAGCAAGAAATTCAAGGTGCTGGGCAACACCGACCGGCACAGCATCAAGAAGAACCTGAT
CGGAGCCCTGCTGTTCGACAGCGGCGAAACAGCCGAGGCCACCCGGCTGAAGAGAACCGCCAGAA
GAAGATACACCAGACGGAAGAACCGGATCTGCTATCTGCAAGAGATCTTCAGCAACGAGATGGCCAA
GGTGGACGACAGCTTCTTCCACAGACTGGAAGAGTCCTTCCTGGTGGAAGAGGATAAGAAGCACGA
GCGGCACCCCATCTTCGGCAACATCGTGGACGAGGTGGCCTACCACGAGAAGTACCCCACCATCTA
CCACCTGAGAAAGAAACTGGTGGACAGCACCGACAAGGCCGACCTGCGGCTGATCTATCTGGCCCT
GGCCCACATGATCAAGTTCCGGGGCCACTTCCTGATCGAGGGCGACCTGAACCCCGACAACAGCGA
CGTGGACAAGCTGTTCATCCAGCTGGTGCAGACCTACAACCAGCTGTTCGAGGAAAACCCCATCAAC
GCCAGCGGCGTGGACGCCAAGGCCATCCTGTCTGCCAGACTGAGCAAGAGCAGACGGCTGGAAAA
TCTGATCGCCCAGCTGCCCGGCGAGAAGAAGAATGGCCTGTTCGGAAACCTGATTGCCCTGAGCCT
GGGCCTGACCCCCAACTTCAAGAGCAACTTCGACCTGGCCGAGGATGCCAAACTGCAGCTGAGCAA
GGACACCTACGACGACGACCTGGACAACCTGCTGGCCCAGATCGGCGACCAGTACGCCGACCTGTT
TCTGGCCGCCAAGAACCTGTCCGACGCCATCCTGCTGAGCGACATCCTGAGAGTGAACACCGAGAT
CACCAAGGCCCCCCTGAGCGCCTCTATGATCAAGAGATACGACGAGCACCACCAGGACCTGACCCT
GCTGAAAGCTCTCGTGCGGCAGCAGCTGCCTGAGAAGTACAAAGAGATTTTCTTCGACCAGAGCAAG
AACGGCTACGCCGGCTACATTGACGGCGGAGCCAGCCAGGAAGAGTTCTACAAGTTCATCAAGCCC
ATCCTGGAAAAGATGGACGGCACCGAGGAACTGCTCGTGAAGCTGAACAGAGAGGACCTGCTGCGG
AAGCAGCGGACCTTCGACAACGGCAGCATCCCCCACCAGATCCACCTGGGAGAGCTGCACGCCATT
CTGCGGCGGCAGGAAGATTTTTACCCATTCCTGAAGGACAACCGGGAAAAGATCGAGAAGATCCTGA
CCTTCCGCATCCCCTACTACGTGGGCCCTCTGGCCAGGGGAAACAGCAGATTCGCCTGGATGACCA
GAAAGAGCGAGGAAACCATCACCCCCTGGAACTTCGAGGAAGTGGTGGACAAGGGCGCTTCCGCC
CAGAGCTTCATCGAGCGGATGACCAACTTCGATAAGAACCTGCCCAACGAGAAGGTGCTGCCCAAG
CACAGCCTGCTGTACGAGTACTTCACCGTGTATAACGAGCTGACCAAAGTGAAATACGTGACCGAGG
GAATGAGAAAGCCCGCCTTCCTGAGCGGCGAGCAGAAAAAGGCCATCGTGGACCTGCTGTTCAAGA
CCAACCGGAAAGTGACCGTGAAGCAGCTGAAAGAGGACTACTTCAAGAAAATCGAGTGCTTCGACTC
CGTGGAAATCTCCGGCGTGGAAGATCGGTTCAACGCCTCCCTGGGCACATACCACGATCTGCTGAAA
ATTATCAAGGACAAGGACTTCCTGGACAATGAGGAAAACGAGGACATTCTGGAAGATATCGTGCTGAC
CCTGACACTGTTTGAGGACAGAGAGATGATCGAGGAACGGCTGAAAACCTATGCCCACCTGTTCGAC
GACAAAGTGATGAAGCAGCTGAAGCGGCGGAGATACACCGGCTGGGGCAGGCTGAGCCGGAAGCT
GATCAACGGCATCCGGGACAAGCAGTCCGGCAAGACAATCCTGGATTTCCTGAAGTCCGACGGCTT
CGCCAACAGAAACTTCATGCAGCTGATCCACGACGACAGCCTGACCTTTAAAGAGGACATCCAGAAA

GCCCAGGTGTCCGGCCAGGGCGATAGCCTGCACGAGCACATTGCCAATCTGGCCGGCAGCCCCGC
CATTAAGAAGGGCATCCTGCAGACAGTGAAGGTGGTGGACGAGCTCGTGAAAGTGATGGGCCGGCA
CAAGCCCGAGAACATCGTGATCGAAATGGCCAGAGAGAACCAGACCACCCAGAAGGGACAGAAGAA
CAGCCGCGAGAGAATGAAGCGGATCGAAGAGGGCATCAAAGAGCTGGGCAGCCAGATCCTGAAAGA
ACACCCCGTGGAAAACACCCAGCTGCAGAACGAGAAGCTGTACCTGTACTACCTGCAGAATGGGCG
GGATATGTACGTGGACCAGGAACTGGACATCAACCGGCTGTCCGACTACGATGTGGACCATATCGTG
CCTCAGAGCTTTCTGAAGGACGACTCCATCGACAACAAGGTGCTGACCAGAAGCGACAAGAACCGG
GGCAAGAGCGACAACGTGCCCTCCGAAGAGGTCGTGAAGAAGATGAAGAACTACTGGCGGCAGCT
GCTGAACGCCAAGCTGATTACCCAGAGAAAGTTCGACAATCTGACCAAGGCCGAGAGAGGCGGCCT
GAGCGAACTGGATAAGGCCGGCTTCATCAAGAGACAGCTGGTGGAAACCCGGCAGATCACAAAGCA
CGTGGCACAGATCCTGGACTCCCGGATGAACACTAAGTACGACGAGAATGACAAGCTGATCCGGGAA
GTGAAAGTGATCACCCTGAAGTCCAAGCTGGTGTCCGATTTCCGGAAGGATTTCCAGTTTTACAAAGT
GCGCGAGATCAACAACTACCACCACGCCCACGACGCCTACCTGAACGCCGTCGTGGGAACCGCCCT
GATCAAAAAGTACCCTAAGCTGGAAAGCGAGTTCGTGTACGGCGACTACAAGGTGTACGACGTGCGG
AAGATGATCGCCAAGAGCGAGCAGGAAATCGGCAAGGCTACCGCCAAGTACTTCTTCTACAGCAACA
TCATGAACTTTTTCAAGACCGAGATTACCCTGGCCAACGGCGAGATCCGGAAGCGGCCTCTGATCGA
GACAAACGGCGAAACCGGGGAGATCGTGTGGGATAAGGGCCGGGATTTTGCCACCGTGCGGAAAGT
GCTGAGCATGCCCCAAGTGAATATCGTGAAAAAGACCGAGGTGCAGACAGGCGGCTTCAGCAAAGA
GTCTATCAGGCCCAAGAGGAACAGCGATAAGCTGATCGCCAGAAAGAAGGACTGGGACCCTAAGAA
GTACGGCGGCTTCGTCAGCCCCACCGTGGCCTATTCTGTGCTGGTGGTGGCCAAAGTGGAAAAGGG
CAAGTCCAAGAAACTGAAGAGTGTGAAAGAGCTGCTGGGGATCACCATCATGGAAAGAAGCAGCTTC
GAGAAGAATCCCATCGACTTTCTGGAAGCCAAGGGCTACAAAGAAGTGAAAAAGGACCTGATCATCA
AGCTGCCTAAGTACTCCCTGTTCGAGCTGGAAAACGGCCGGAAGAGAATGCTGGCCTCTGCCAGATT
CCTGCAGAAGGGAAACGAACTGGCCCTGCCCTCCAAATATGTGAACTTCCTGTACCTGGCCAGCCAC
TATGAGAAGCTGAAGGGCTCCCCCGAGGATAATGAGCAGAAACAGCTGTTTGTGGAACAGCACAAGC
ACTACCTGGACGAGATCATCGAGCAGATCAGCGAGTTCTCCAAGAGAGTGATCCTGGCCGACGCTAA
TCTGGACAAAGTGCTGTCCGCCTACAACAAGCACCGGGATAAGCCCATCAGAGAGCAGGCCGAGAA
TATCATCCACCTGTTTACCCTGACCAATCTGGGAGCCCCTAGGGCCTTCAAGTACTTTGACACCACCA
TCGACCGGAAGGTGTACAGGAGCACCAAAGAGGTGCTGGACGCCACCCTGATCCACCAGAGCATCA
CCGGCCTGTACGAGACACGGATCGACCTGTCTCAGCTGGGAGGTGACAGCGGCGGGAGCGGCGG
GAGCGGGGGGAGCACTAATCTGAGCGACATCATTGAGAAGGAGACTGGGAAACAGCTGGTCATTCA
GGAGTCCATCCTGATGCTGCCTGAGGAGGTGGAGGAAGTGATCGGCAACAAGCCAGAGTCTGACAT
CCTGGTGCACACCGCCTACGACGAGTCCACAGATGAGAATGTGATGCTGCTGACCTCTGACGCCCC
CGAGTATAAGCCTTGGGCCCTGGTCATCCAGGATTCTAACGGCGAGAATAAGATCAAGATGCTGAGC
GGAGGATCCGGAGGATCTGGAGGCAGC*ACCAACCTGTCTGACATCATCGAGAAGGAGACAGGCAAG*
CAGCTGGTCATCCAGGAGAGCATCCTGATGCTGCCCGAAGAAGTCGAAGAAGTGATCGGAAACAAG
CCTGAGAGCGATATCCTGGTCCATACCGCCTACGACGAGAGTACCGACGAAAATGTGATGCTGCTGA
CATCCGACGCCCCAGAGTATAAGCCCTGGGCTCTGGTCATCCAGGATTCCAACGGAGAGAACAAAAT
*CAAAATGCTG***TCTGGCGGCTCAAAAAGAACCGCCGACGGCAGCGAATTCGAGCCCAAGAAGAAGA**
GGAAAGTCTAATTAATTAAGCTGCCTTCTGCGGGGCTTGCCTTCTGGCCATGCCCTTCTTCTCTCCCT
TGCACCTGTACCTCTTGGTCTTTGAATAAAGCCTGAGTAGGAAGCGACTGTGCCTTCTAGTTGCCAGC
CATCTGTTGTTTGCCCCTCCCCCGTGCCTTCCTTGACCCTGGAAGGTGCCACTCCCACTGTCCTTTC
CTAATAAAATGAGAAAATTGCATCGCATTGTCTGAGTAGGTGTCATTCTATTCTGGGGGGTGGGGTGG
GGCAGGACAGCAAGGGGGAGGATTGGGAAGACAATAGCAGGCATGCTGGGGATGCGGTGGGCTCT
ATGG

Start	End	Feature Description
1	380	CMV enhancer
381	619	CMV promoter
620	636	T7 promoter with mismatch
637	683	5' UTR

684	740	BP NLS
741	1241	TadCBEd deaminase
1242	1337	linker
1338	5438	Cas9(D10A) Sp-NG PAM variant
5439	5468	Linker
5469	5717	uracil glycosylase inhibitor
5718	5747	Linker
5748	*5996*	*uracil glycosylase inhibitor*
**5997**	**6062**	**linker + SV40 BP NLS**
6063	6163	3' UTR
6164	6391	bGH poly(A) signal

Amino acid sequence
MKRTADGSEFESPKKKRKVSSEVEFSHEYWMRHALTLAKRARDERKAPVGAVLVLNNRVIGEGWNRAIG
LHDPTAHAEIIALRQGGLVMQNYRLIDATLYVTFEPCVMCAGAMINSRIGRVVFGVRNSKRGAAGSLMNVL
NYPGMNHRVEITEGILADECAALLCDFYRMPRQVFNAQKKAQSSINSGGSSGGSSGSETPGTSESATPE
SSGGSSGGSDKKYSIGLAIGTNSVGWAVITDEYKVPSKKFKVLGNTDRHSIKKNLIGALLFDSGETAEATRL
KRTARRRYTRRKNRICYLQEIFSNEMAKVDDSFFHRLEESFLVEEDKKHERHPIFGNIVDEVAYHEKYPTIY
HLRKKLVDSTDKADLRLIYLALAHMIKFRGHFLIEGDLNPDNSDVDKLFIQLVQTYNQLFEENPINASGVDA
KAILSARLSKSRRLENLIAQLPGEKKNGLFGNLIALSLGLTPNFKSNFDLAEDAKLQLSKDTYDDDLDNLLA
QIGDQYADLFLAAKNLSDAILLSDILRVNTEITKAPLSASMIKRYDEHHQDLTLLKALVRQQLPEKYKEIFFD
QSKNGYAGYIDGGASQEEFYKFIKPILEKMDGTEELLVKLNREDLLRKQRTFDNGSIPHQIHLGELHAILRR
QEDFYPFLKDNREKIEKILTFRIPYYVGPLARGNSRFAWMTRKSEETITPWNFEEVVDKGASAQSFIERMT
NFDKNLPNEKVLPKHSLLYEYFTVYNELTKVKYVTEGMRKPAFLSGEQKKAIVDLLFKTNRKVTVKQLKED
YFKKIECFDSVEISGVEDRFNASLGTYHDLLKIIKDKDFLDNEENEDILEDIVLTLTLFEDREMIEERLKTYAH
LFDDKVMKQLKRRRYTGWGRLSRKLINGIRDKQSGKTILDFLKSDGFANRNFMQLIHDDSLTFKEDIQKAQ
VSGQGDSLHEHIANLAGSPAIKKGILQTVKVVDELVKVMGRHKPENIVIEMARENQTTQKGQKNSRERMK
RIEEGIKELGSQILKEHPVENTQLQNEKLYLYYLQNGRDMYVDQELDINRLSDYDVDHIVPQSFLKDDSIDN
KVLTRSDKNRGKSDNVPSEEVVKKMKNYWRQLLNAKLITQRKFDNLTKAERGGLSELDKAGFIKRQLVET
RQITKHVAQILDSRMNTKYDENDKLIREVKVITLKSKLVSDFRKDFQFYKVREINNYHHAHDAYLNAVVGTA
LIKKYPKLESEFVYGDYKVYDVRKMIAKSEQEIGKATAKYFFYSNIMNFFKTEITLANGEIRKRPLIETNGET
GEIVWDKGRDFATVRKVLSMPQVNIVKKTEVQTGGFSKESIRPKRNSDKLIARKKDWDPKKYGGFVSPTV
AYSVLVVAKVEKGKSKKLKSVKELLGITIMERSSFEKNPIDFLEAKGYKEVKKDLIIKLPKYSLFELENGRKR
MLASARFLQKGNELALPSKYVNFLYLASHYEKLKGSPEDNEQKQLFVEQHKHYLDEIIEQISEFSKRVILAD
ANLDKVLSAYNKHRDKPIREQAENIIHLFTLTNLGAPRAFKYFDTTIDRKVYRSTKEVLDATLIHQSITGLYET
RIDLSQLGGDSGGSGGSGGSTNLSDIIEKETGKQLVIQESILMLPEEVEEVIGNKPESDILVHTAYDESTDE
NVMLLTSDAPEYKPWALVIQDSNGENKIKMLSGGSGGSGGS*TNLSDIIEKETGKQLVIQESILMLPEEVEEV*
*IGNKPESDILVHTAYDESTDENVMLLTSDAPEYKPWALVIQDSNGENKIKML***SGGSKRTADGSEFEPKKK**
RKV

Start	End	Feature Description
1	19	BP NLS
20	186	TadCBEd deaminase
187	218	BE4max linker

219	1585	Cas9(D10A) Sp-NG PAM variant
1586	1595	Linker
1596	1678	uracil glycosylase inhibitor
1679	1688	Linker
1689	*1771*	*uracil glycosylase inhibitor*
**1772**	**1792**	**linker + SV40 BP NLS**

CBE6b – Cas9 (D10A) – SpNG variant – UGI
Plasmid ID: pHS0420

DNA sequence

GACATTGATTATTGACTAGTTATTAATAGTAATCAATTACGGGGTCATTAGTTCATAGCCCATATATGGAG
TTCCGCGTTACATAACTTACGGTAAATGGCCCGCCTGGCTGACCGCCCAACGACCCCCGCCCATTGA
CGTCAATAATGACGTATGTTCCCATAGTAACGCCAATAGGGACTTTCCATTGACGTCAATGGGTGGAGT
ATTTACGGTAAACTGCCCACTTGGCAGTACATCAAGTGTATCATATGCCAAGTACGCCCCCTATTGACG
TCAATGACGGTAAATGGCCCGCCTGGCATTATGCCCAGTACATGACCTTATGGGACTTTCCTACTTGG
CAGTACATCTACGTATTAGTCATCGCTATTACCATGGTGATGCGGTTTTGGCAGTACATCAATGGGCGT
GGATAGCGGTTTGACTCACGGGGATTTCCAAGTCTCCACCCCATTGACGTCAATGGGAGTTTGTTTTG
GCACCAAAATCAACGGGACTTTCCAAAATGTCGTAACAACTCCGCCCCATTGACGCAAATGGGCGGT
AGGCGTGTACGGTGGGAGGTCTATATAAGCAGAGCTGGTTTAGTGAACCGTCAGATCTCGAGCTCGG
TACCTAATACGACACACTATAAGGAAATAAGAGAGAAAAGAAGAGTAAGAAGAAATATAAGAGCCACCA
TGAAACGGACAGCCGACGGAAGCGAGTTCGAGTCACCAAAGAAGAAGCGGAAAGTCAGTTCTGAGG
TGGAGTTTTCCCACGAGTACTGGATGAGACATGCCCTGACCCTGGCCAAGAGGGCACGGGATGAGG
GAGAGGCGCCTGTGGGAGCCGTGCTGGTGCTGAACAATAGAGTGATCGGCGAGGGCTGGGTGAGA
CGTATCGGCCTGCACGACCCAACAGCCCATGCCGAAATTATGGCCCTGAGACAGGGCGGCCTGGTC
ATGCAGAACCCCAGACTGATTGACGCCACCCTGTACGTGACATTCGAGCCTTGCGTGATGTGCGCCG
GCGCCATGATCAACTCTAGGATCGGCCGCGTGGTGTTTGGCGTGAGGAACTCAAAAAGAGGCGCCG
CAGGCTCCCTGATGAACGTGCTGAACTACCCCGGCATGAATCACCGCGTCGAAATTACCGAGGGAAT
CCTGGCAGATGAATGTGCCGCCCTGCTGTGCGATTTCTATCGGATGCCTAGACAGGTGTTCAATGCTC
AGAAGAAGGCCCAGAGCTCCATCAACTCTGGCGGATCTAGCGGAGGATCCTCTGGCAGCGAGACAC
CAGGAACAAGCGAGTCAGCAACACCAGAGAGCAGTGGCGGCAGCAGCGGCGGCAGCGACAAGAA
GTACAGCATCGGCCTGGCCATCGGCACCAACTCTGTGGGCTGGGCCGTGATCACCGACGAGTACAA
GGTGCCCAGCAAGAAATTCAAGGTGCTGGGCAACACCGACCGGCACAGCATCAAGAAGAACCTGAT
CGGAGCCCTGCTGTTCGACAGCGGCGAAACAGCCGAGGCCACCCGGCTGAAGAGAACCGCCAGAA
GAAGATACACCAGACGGAAGAACCGGATCTGCTATCTGCAAGAGATCTTCAGCAACGAGATGGCCAA
GGTGGACGACAGCTTCTTCCACAGACTGGAAGAGTCCTTCCTGGTGGAAGAGGATAAGAAGCACGA
GCGGCACCCCATCTTCGGCAACATCGTGGACGAGGTGGCCTACCACGAGAAGTACCCCACCATCTA
CCACCTGAGAAAGAAACTGGTGGACAGCACCGACAAGGCCGACCTGCGGCTGATCTATCTGGCCCT
GGCCCACATGATCAAGTTCCGGGGCCACTTCCTGATCGAGGGCGACCTGAACCCCGACAACAGCGA
CGTGGACAAGCTGTTCATCCAGCTGGTGCAGACCTACAACCAGCTGTTCGAGGAAAACCCCATCAAC
GCCAGCGGCGTGGACGCCAAGGCCATCCTGTCTGCCAGACTGAGCAAGAGCAGACGGCTGGAAAA
TCTGATCGCCCAGCTGCCCGGCGAGAAGAAGAATGGCCTGTTCGGAAACCTGATTGCCCTGAGCCT
GGGCCTGACCCCCAACTTCAAGAGCAACTTCGACCTGGCCGAGGATGCCAAACTGCAGCTGAGCAA
GGACACCTACGACGACGACCTGGACAACCTGCTGGCCCAGATCGGCGACCAGTACGCCGACCTGTT
TCTGGCCGCCAAGAACCTGTCCGACGCCATCCTGCTGAGCGACATCCTGAGAGTGAACACCGAGAT
CACCAAGGCCCCCCTGAGCGCCTCTATGATCAAGAGATACGACGAGCACCACCAGGACCTGACCCT
GCTGAAAGCTCTCGTGCGGCAGCAGCTGCCTGAGAAGTACAAAGAGATTTTCTTCGACCAGAGCAAG
AACGGCTACGCCGGCTACATTGACGGCGGAGCCAGCCAGGAAGAGTTCTACAAGTTCATCAAGCCC
ATCCTGGAAAAGATGGACGGCACCGAGGAACTGCTCGTGAAGCTGAACAGAGAGGACCTGCTGCGG
AAGCAGCGGACCTTCGACAACGGCAGCATCCCCCACCAGATCCACCTGGGAGAGCTGCACGCCATT
CTGCGGCGGCAGGAAGATTTTTACCCATTCCTGAAGGACAACCGGGAAAAGATCGAGAAGATCCTGA
CCTTCCGCATCCCCTACTACGTGGGCCCTCTGGCCAGGGGAAACAGCAGATTCGCCTGGATGACCA
GAAAGAGCGAGGAAACCATCACCCCCTGGAACTTCGAGGAAGTGGTGGACAAGGGCGCTTCCGCC
CAGAGCTTCATCGAGCGGATGACCAACTTCGATAAGAACCTGCCCAACGAGAAGGTGCTGCCCAAG
CACAGCCTGCTGTACGAGTACTTCACCGTGTATAACGAGCTGACCAAAGTGAAATACGTGACCGAGG
GAATGAGAAAGCCCGCCTTCCTGAGCGGCGAGCAGAAAAAGGCCATCGTGGACCTGCTGTTCAAGA
CCAACCGGAAAGTGACCGTGAAGCAGCTGAAAGAGGACTACTTCAAGAAAATCGAGTGCTTCGACTC
CGTGGAAATCTCCGGCGTGGAAGATCGGTTCAACGCCTCCCTGGGCACATACCACGATCTGCTGAAA
ATTATCAAGGACAAGGACTTCCTGGACAATGAGGAAAACGAGGACATTCTGGAAGATATCGTGCTGAC
CCTGACACTGTTTGAGGACAGAGAGATGATCGAGGAACGGCTGAAAACCTATGCCCACCTGTTCGAC
GACAAAGTGATGAAGCAGCTGAAGCGGCGGAGATACACCGGCTGGGGCAGGCTGAGCCGGAAGCT
GATCAACGGCATCCGGGACAAGCAGTCCGGCAAGACAATCCTGGATTTCCTGAAGTCCGACGGCTT
CGCCAACAGAAACTTCATGCAGCTGATCCACGACGACAGCCTGACCTTTAAAGAGGACATCCAGAAA

GCCCAGGTGTCCGGCCAGGGCGATAGCCTGCACGAGCACATTGCCAATCTGGCCGGCAGCCCCGC
CATTAAGAAGGGCATCCTGCAGACAGTGAAGGTGGTGGACGAGCTCGTGAAAGTGATGGGCCGGCA
CAAGCCCGAGAACATCGTGATCGAAATGGCCAGAGAGAACCAGACCACCCAGAAGGGACAGAAGAA
CAGCCGCGAGAGAATGAAGCGGATCGAAGAGGGCATCAAAGAGCTGGGCAGCCAGATCCTGAAAGA
ACACCCCGTGGAAAACACCCAGCTGCAGAACGAGAAGCTGTACCTGTACTACCTGCAGAATGGGCG
GGATATGTACGTGGACCAGGAACTGGACATCAACCGGCTGTCCGACTACGATGTGGACCATATCGTG
CCTCAGAGCTTTCTGAAGGACGACTCCATCGACAACAAGGTGCTGACCAGAAGCGACAAGAACCGG
GGCAAGAGCGACAACGTGCCCTCCGAAGAGGTCGTGAAGAAGATGAAGAACTACTGGCGGCAGCT
GCTGAACGCCAAGCTGATTACCCAGAGAAAGTTCGACAATCTGACCAAGGCCGAGAGAGGCGGCCT
GAGCGAACTGGATAAGGCCGGCTTCATCAAGAGACAGCTGGTGGAAACCCGGCAGATCACAAAGCA
CGTGGCACAGATCCTGGACTCCCGGATGAACACTAAGTACGACGAGAATGACAAGCTGATCCGGGAA
GTGAAAGTGATCACCCTGAAGTCCAAGCTGGTGTCCGATTTCCGGAAGGATTTCCAGTTTTACAAAGT
GCGCGAGATCAACAACTACCACCACGCCCACGACGCCTACCTGAACGCCGTCGTGGGAACCGCCCT
GATCAAAAAGTACCCTAAGCTGGAAAGCGAGTTCGTGTACGGCGACTACAAGGTGTACGACGTGCGG
AAGATGATCGCCAAGAGCGAGCAGGAAATCGGCAAGGCTACCGCCAAGTACTTCTTCTACAGCAACA
TCATGAACTTTTTCAAGACCGAGATTACCCTGGCCAACGGCGAGATCCGGAAGCGGCCTCTGATCGA
GACAAACGGCGAAACCGGGGAGATCGTGTGGGATAAGGGCCGGGATTTTGCCACCGTGCGGAAAGT
GCTGAGCATGCCCCAAGTGAATATCGTGAAAAAGACCGAGGTGCAGACAGGCGGCTTCAGCAAAGA
GTCTATCAGGCCCAAGAGGAACAGCGATAAGCTGATCGCCAGAAAGAAGGACTGGGACCCTAAGAA
GTACGGCGGCTTCGTCAGCCCCACCGTGGCCTATTCTGTGCTGGTGGTGGCCAAAGTGGAAAAGGG
CAAGTCCAAGAAACTGAAGAGTGTGAAAGAGCTGCTGGGGATCACCATCATGGAAAGAAGCAGCTTC
GAGAAGAATCCCATCGACTTTCTGGAAGCCAAGGGCTACAAAGAAGTGAAAAAGGACCTGATCATCA
AGCTGCCTAAGTACTCCCTGTTCGAGCTGGAAAACGGCCGGAAGAGAATGCTGGCCTCTGCCAGATT
CCTGCAGAAGGGAAACGAACTGGCCCTGCCCTCCAAATATGTGAACTTCCTGTACCTGGCCAGCCAC
TATGAGAAGCTGAAGGGCTCCCCCGAGGATAATGAGCAGAAACAGCTGTTTGTGGAACAGCACAAGC
ACTACCTGGACGAGATCATCGAGCAGATCAGCGAGTTCTCCAAGAGAGTGATCCTGGCCGACGCTAA
TCTGGACAAAGTGCTGTCCGCCTACAACAAGCACCGGGATAAGCCCATCAGAGAGCAGGCCGAGAA
TATCATCCACCTGTTTACCCTGACCAATCTGGGAGCCCCTAGGGCCTTCAAGTACTTTGACACCACCA
TCGACCGGAAGGTGTACAGGAGCACCAAAGAGGTGCTGGACGCCACCCTGATCCACCAGAGCATCA
CCGGCCTGTACGAGACACGGATCGACCTGTCTCAGCTGGGAGGTGACAGCGGCGGGAGCGGCGG
GAGCGGGGGGAGCACTAATCTGAGCGACATCATTGAGAAGGAGACTGGGAAACAGCTGGTCATTCA
GGAGTCCATCCTGATGCTGCCTGAGGAGGTGGAGGAAGTGATCGGCAACAAGCCAGAGTCTGACAT
CCTGGTGCACACCGCCTACGACGAGTCCACAGATGAGAATGTGATGCTGCTGACCTCTGACGCCCC
CGAGTATAAGCCTTGGGCCCTGGTCATCCAGGATTCTAACGGCGAGAATAAGATCAAGATGCTGAGC
GGAGGATCCGGAGGATCTGGAGGCAGC*ACCAACCTGTCTGACATCATCGAGAAGGAGACAGGCAAG*
CAGCTGGTCATCCAGGAGAGCATCCTGATGCTGCCCGAAGAAGTCGAAGAAGTGATCGGAAACAAG
CCTGAGAGCGATATCCTGGTCCATACCGCCTACGACGAGAGTACCGACGAAAATGTGATGCTGCTGA
CATCCGACGCCCCAGAGTATAAGCCCTGGGCTCTGGTCATCCAGGATTCCAACGGAGAGAACAAAAT
*CAAAATGCTG***TCTGGCGGCTCAAAAAGAACCGCCGACGGCAGCGAATTCGAGCCCAAGAAGAAGA**
GGAAAGTCTAATTAATTAAGCTGCCTTCTGCGGGGCTTGCCTTCTGGCCATGCCCTTCTTCTCTCCCT
TGCACCTGTACCTCTTGGTCTTTGAATAAAGCCTGAGTAGGAAGCGACTGTGCCTTCTAGTTGCCAGC
CATCTGTTGTTTGCCCCTCCCCCGTGCCTTCCTTGACCCTGGAAGGTGCCACTCCCACTGTCCTTTC
CTAATAAAATGAGAAAATTGCATCGCATTGTCTGAGTAGGTGTCATTCTATTCTGGGGGGTGGGGTGG
GGCAGGACAGCAAGGGGGAGGATTGGGAAGACAATAGCAGGCATGCTGGGGATGCGGTGGGCTCT
ATGG

Start	End	Feature Description
1	380	CMV enhancer
381	619	CMV promoter
620	636	T7 promoter with mismatch
637	683	5’ UTR

684	740	BP NLS
741	1241	CBE6b deaminase
1242	1337	Linker
1338	5438	Cas9(D10A) Sp-NG PAM variant
5439	5468	Linker
5469	5717	uracil glycosylase inhibitor
5718	5747	Linker
5748	*5996*	*uracil glycosylase inhibitor*
**5997**	**6062**	**linker + SV40 BP NLS**
6063	6163	3' UTR
6164	6391	bGH poly(A) signal

Amino acid sequence
MKRTADGSEFESPKKKRKVSSEVEFSHEYWMRHALTLAKRARDEGEAPVGAVLVLNNRVIGEGWVRRIG
LHDPTAHAEIMALRQGGLVMQNPRLIDATLYVTFEPCVMCAGAMINSRIGRVVFGVRNSKRGAAGSLMNV
LNYPGMNHRVEITEGILADECAALLCDFYRMPRQVFNAQKKAQSSINSGGSSGGSSGSETPGTSESATP
ESSGGSSGGSDKKYSIGLAIGTNSVGWAVITDEYKVPSKKFKVLGNTDRHSIKKNLIGALLFDSGETAEAT
RLKRTARRRYTRRKNRICYLQEIFSNEMAKVDDSFFHRLEESFLVEEDKKHERHPIFGNIVDEVAYHEKYP
TIYHLRKKLVDSTDKADLRLIYLALAHMIKFRGHFLIEGDLNPDNSDVDKLFIQLVQTYNQLFEENPINASGV
DAKAILSARLSKSRRLENLIAQLPGEKKNGLFGNLIALSLGLTPNFKSNFDLAEDAKLQLSKDTYDDDLDNL
LAQIGDQYADLFLAAKNLSDAILLSDILRVNTEITKAPLSASMIKRYDEHHQDLTLLKALVRQQLPEKYKEIFF
DQSKNGYAGYIDGGASQEEFYKFIKPILEKMDGTEELLVKLNREDLLRKQRTFDNGSIPHQIHLGELHAILR
RQEDFYPFLKDNREKIEKILTFRIPYYVGPLARGNSRFAWMTRKSEETITPWNFEEVVDKGASAQSFIERM
TNFDKNLPNEKVLPKHSLLYEYFTVYNELTKVKYVTEGMRKPAFLSGEQKKAIVDLLFKTNRKVTVKQLKE
DYFKKIECFDSVEISGVEDRFNASLGTYHDLLKIIKDKDFLDNEENEDILEDIVLTLTLFEDREMIEERLKTYA
HLFDDKVMKQLKRRRYTGWGRLSRKLINGIRDKQSGKTILDFLKSDGFANRNFMQLIHDDSLTFKEDIQKA
QVSGQGDSLHEHIANLAGSPAIKKGILQTVKVVDELVKVMGRHKPENIVIEMARENQTTQKGQKNSRERM
KRIEEGIKELGSQILKEHPVENTQLQNEKLYLYYLQNGRDMYVDQELDINRLSDYDVDHIVPQSFLKDDSID
NKVLTRSDKNRGKSDNVPSEEVVKKMKNYWRQLLNAKLITQRKFDNLTKAERGGLSELDKAGFIKRQLVE
TRQITKHVAQILDSRMNTKYDENDKLIREVKVITLKSKLVSDFRKDFQFYKVREINNYHHAHDAYLNAVVGT
ALIKKYPKLESEFVYGDYKVYDVRKMIAKSEQEIGKATAKYFFYSNIMNFFKTEITLANGEIRKRPLIETNGE
TGEIVWDKGRDFATVRKVLSMPQVNIVKKTEVQTGGFSKESIRPKRNSDKLIARKKDWDPKKYGGFVSPT
VAYSVLVVAKVEKGKSKKLKSVKELLGITIMERSSFEKNPIDFLEAKGYKEVKKDLIIKLPKYSLFELENGRK
RMLASARFLQKGNELALPSKYVNFLYLASHYEKLKGSPEDNEQKQLFVEQHKHYLDEIIEQISEFSKRVILA
DANLDKVLSAYNKHRDKPIREQAENIIHLFTLTNLGAPRAFKYFDTTIDRKVYRSTKEVLDATLIHQSITGLYE
TRIDLSQLGGDSGGSGGSGGSTNLSDIIEKETGKQLVIQESILMLPEEVEEVIGNKPESDILVHTAYDESTDE
NVMLLTSDAPEYKPWALVIQDSNGENKIKMLSGGSGGSGGS*TNLSDIIEKETGKQLVIQESILMLPEEVEEV*
*IGNKPESDILVHTAYDESTDENVMLLTSDAPEYKPWALVIQDSNGENKIKML***SGGSKRTADGSEFEPKKK**
RKV

Start	End	Feature Description
1	19	BP NLS
20	186	TadCBEd deaminase
187	218	Linker

219	1585	Cas9(D10A) Sp-NG PAM variant
1586	1595	Linker
1596	1678	uracil glycosylase inhibitor
1679	1688	Linker
1689	*1771*	*uracil glycosylase inhibitor*
**1772**	**1792**	**linker + SV40 BP NLS**

BE4max – Cas9 (D10A) – SpCas9 (WT) – UGI
Plasmid ID: pHS0388

DNA sequence

GACATTGATTATTGACTAGTTATTAATAGTAATCAATTACGGGGTCATTAGTTCATAGCCCATATATGGAG
TTCCGCGTTACATAACTTACGGTAAATGGCCCGCCTGGCTGACCGCCCAACGACCCCCGCCCATTGA
CGTCAATAATGACGTATGTTCCCATAGTAACGCCAATAGGGACTTTCCATTGACGTCAATGGGTGGAGT
ATTTACGGTAAACTGCCCACTTGGCAGTACATCAAGTGTATCATATGCCAAGTACGCCCCCTATTGACG
TCAATGACGGTAAATGGCCCGCCTGGCATTATGCCCAGTACATGACCTTATGGGACTTTCCTACTTGG
CAGTACATCTACGTATTAGTCATCGCTATTACCATGGTGATGCGGTTTTGGCAGTACATCAATGGGCGT
GGATAGCGGTTTGACTCACGGGGATTTCCAAGTCTCCACCCCATTGACGTCAATGGGAGTTTGTTTTG
GCACCAAAATCAACGGGACTTTCCAAAATGTCGTAACAACTCCGCCCCATTGACGCAAATGGGCGGT
AGGCGTGTACGGTGGGAGGTCTATATAAGCAGAGCTGGTTTAGTGAACCGTCAGATCTCGAGCTCGG
TACCTAATACGACACACTATAAGGAAATAAGAGAGAAAAGAAGAGTAAGAAGAAATATAAGAGCCACCA
TGAAACGGACAGCCGACGGAAGCGAGTTCGAGTCACCAAAGAAGAAGCGGAAAGTCAGTTCAGAGA
CTGGGCCTGTCGCCGTCGATCCAACCCTGCGCCGCCGGATTGAACCTCACGAGTTTGAAGTGTTCTT
TGACCCCCGGGAGCTGAGAAAGGAGACATGCCTGCTGTACGAGATCAACTGGGGAGGCAGGCACTC
CATCTGGAGGCACACCTCTCAGAACACAAATAAGCACGTGGAGGTGAACTTCATCGAGAAGTTTACC
ACAGAGCGGTACTTCTGCCCCAATACCAGATGTAGCATCACATGGTTTCTGAGCTGGTCCCCTTGCG
GAGAGTGTAGCAGGGCCATCACCGAGTTCCTGTCCAGATATCCACACGTGACACTGTTTATCTACATC
GCCAGGCTGTATCACCACGCAGACCCAAGGAATAGGCAGGGCCTGCGCGATCTGATCAGCTCCGGC
GTGACCATCCAGATCATGACAGAGCAGGAGTCCGGCTACTGCTGGCGGAACTTCGTGAATTATTCTC
CTAGCAACGAGGCCCACTGGCCTAGGTACCCACACCTGTGGGTGCGCCTGTACGTGCTGGAGCTGT
ATTGCATCATCCTGGGCCTGCCCCCTTGTCTGAATATCCTGCGGAGAAAGCAGCCCCAGCTGACCTT
CTTTACAATCGCCCTGCAGTCTTGTCACTATCAGAGGCTGCCACCCCACATCCTGTGGGCCACAGGC
CTGAAGTCTGGCGGATCTAGCGGAGGATCCTCTGGCAGCGAGACACCAGGAACAAGCGAGTCAGCA
ACACCAGAGAGCAGTGGCGGCAGCAGCGGCGGCAGCGACAAGAAGTACAGCATCGGCCTGGCCAT
CGGCACCAACTCTGTGGGCTGGGCCGTGATCACCGACGAGTACAAGGTGCCCAGCAAGAAATTCAA
GGTGCTGGGCAACACCGACCGGCACAGCATCAAGAAGAACCTGATCGGAGCCCTGCTGTTCGACAG
CGGCGAAACAGCCGAGGCCACCCGGCTGAAGAGAACCGCCAGAAGAAGATACACCAGACGGAAGA
ACCGGATCTGCTATCTGCAAGAGATCTTCAGCAACGAGATGGCCAAGGTGGACGACAGCTTCTTCCA
CAGACTGGAAGAGTCCTTCCTGGTGGAAGAGGATAAGAAGCACGAGCGGCACCCCATCTTCGGCAA
CATCGTGGACGAGGTGGCCTACCACGAGAAGTACCCCACCATCTACCACCTGAGAAAGAAACTGGTG
GACAGCACCGACAAGGCCGACCTGCGGCTGATCTATCTGGCCCTGGCCCACATGATCAAGTTCCGG
GGCCACTTCCTGATCGAGGGCGACCTGAACCCCGACAACAGCGACGTGGACAAGCTGTTCATCCAG
CTGGTGCAGACCTACAACCAGCTGTTCGAGGAAAACCCCATCAACGCCAGCGGCGTGGACGCCAAG
GCCATCCTGTCTGCCAGACTGAGCAAGAGCAGACGGCTGGAAAATCTGATCGCCCAGCTGCCCGGC
GAGAAGAAGAATGGCCTGTTCGGAAACCTGATTGCCCTGAGCCTGGGCCTGACCCCCAACTTCAAG
AGCAACTTCGACCTGGCCGAGGATGCCAAACTGCAGCTGAGCAAGGACACCTACGACGACGACCTG
GACAACCTGCTGGCCCAGATCGGCGACCAGTACGCCGACCTGTTTCTGGCCGCCAAGAACCTGTCC
GACGCCATCCTGCTGAGCGACATCCTGAGAGTGAACACCGAGATCACCAAGGCCCCCCTGAGCGCC
TCTATGATCAAGAGATACGACGAGCACCACCAGGACCTGACCCTGCTGAAAGCTCTCGTGCGGCAGC
AGCTGCCTGAGAAGTACAAAGAGATTTTCTTCGACCAGAGCAAGAACGGCTACGCCGGCTACATTGA
CGGCGGAGCCAGCCAGGAAGAGTTCTACAAGTTCATCAAGCCCATCCTGGAAAAGATGGACGGCAC
CGAGGAACTGCTCGTGAAGCTGAACAGAGAGGACCTGCTGCGGAAGCAGCGGACCTTCGACAACG
GCAGCATCCCCCACCAGATCCACCTGGGAGAGCTGCACGCCATTCTGCGGCGGCAGGAAGATTTTT
ACCCATTCCTGAAGGACAACCGGGAAAAGATCGAGAAGATCCTGACCTTCCGCATCCCCTACTACGT
GGGCCCTCTGGCCAGGGGAAACAGCAGATTCGCCTGGATGACCAGAAAGAGCGAGGAAACCATCAC
CCCCTGGAACTTCGAGGAAGTGGTGGACAAGGGCGCTTCCGCCCAGAGCTTCATCGAGCGGATGAC
CAACTTCGATAAGAACCTGCCCAACGAGAAGGTGCTGCCCAAGCACAGCCTGCTGTACGAGTACTTC
ACCGTGTATAACGAGCTGACCAAAGTGAAATACGTGACCGAGGGAATGAGAAAGCCCGCCTTCCTGA
GCGGCGAGCAGAAAAAGGCCATCGTGGACCTGCTGTTCAAGACCAACCGGAAAGTGACCGTGAAGC
AGCTGAAAGAGGACTACTTCAAGAAAATCGAGTGCTTCGACTCCGTGGAAATCTCCGGCGTGGAAGA
TCGGTTCAACGCCTCCCTGGGCACATACCACGATCTGCTGAAAATTATCAAGGACAAGGACTTCCTG
GACAATGAGGAAAACGAGGACATTCTGGAAGATATCGTGCTGACCCTGACACTGTTTGAGGACAGAG
AGATGATCGAGGAACGGCTGAAAACCTATGCCCACCTGTTCGACGACAAAGTGATGAAGCAGCTGAA

GCGGCGGAGATACACCGGCTGGGGCAGGCTGAGCCGGAAGCTGATCAACGGCATCCGGGACAAGC
AGTCCGGCAAGACAATCCTGGATTTCCTGAAGTCCGACGGCTTCGCCAACAGAAACTTCATGCAGCT
GATCCACGACGACAGCCTGACCTTTAAAGAGGACATCCAGAAAGCCCAGGTGTCCGGCCAGGGCGA
TAGCCTGCACGAGCACATTGCCAATCTGGCCGGCAGCCCCGCCATTAAGAAGGGCATCCTGCAGAC
AGTGAAGGTGGTGGACGAGCTCGTGAAAGTGATGGGCCGGCACAAGCCCGAGAACATCGTGATCGA
AATGGCCAGAGAGAACCAGACCACCCAGAAGGGACAGAAGAACAGCCGCGAGAGAATGAAGCGGAT
CGAAGAGGGCATCAAAGAGCTGGGCAGCCAGATCCTGAAAGAACACCCCGTGGAAAACACCCAGCT
GCAGAACGAGAAGCTGTACCTGTACTACCTGCAGAATGGGCGGGATATGTACGTGGACCAGGAACTG
GACATCAACCGGCTGTCCGACTACGATGTGGACCATATCGTGCCTCAGAGCTTTCTGAAGGACGACT
CCATCGACAACAAGGTGCTGACCAGAAGCGACAAGAACCGGGGCAAGAGCGACAACGTGCCCTCC
GAAGAGGTCGTGAAGAAGATGAAGAACTACTGGCGGCAGCTGCTGAACGCCAAGCTGATTACCCAG
AGAAAGTTCGACAATCTGACCAAGGCCGAGAGAGGCGGCCTGAGCGAACTGGATAAGGCCGGCTTC
ATCAAGAGACAGCTGGTGGAAACCCGGCAGATCACAAAGCACGTGGCACAGATCCTGGACTCCCGG
ATGAACACTAAGTACGACGAGAATGACAAGCTGATCCGGGAAGTGAAAGTGATCACCCTGAAGTCCA
AGCTGGTGTCCGATTTCCGGAAGGATTTCCAGTTTTACAAAGTGCGCGAGATCAACAACTACCACCAC
GCCCACGACGCCTACCTGAACGCCGTCGTGGGAACCGCCCTGATCAAAAAGTACCCTAAGCTGGAA
AGCGAGTTCGTGTACGGCGACTACAAGGTGTACGACGTGCGGAAGATGATCGCCAAGAGCGAGCAG
GAAATCGGCAAGGCTACCGCCAAGTACTTCTTCTACAGCAACATCATGAACTTTTTCAAGACCGAGAT
TACCCTGGCCAACGGCGAGATCCGGAAGCGGCCTCTGATCGAGACAAACGGCGAAACCGGGGAGA
TCGTGTGGGATAAGGGCCGGGATTTTGCCACCGTGCGGAAAGTGCTGAGCATGCCCCAAGTGAATAT
CGTGAAAAAGACCGAGGTGCAGACAGGCGGCTTCAGCAAAGAGTCTATCCTGCCCAAGAGGAACAG
CGATAAGCTGATCGCCAGAAAGAAGGACTGGGACCCTAAGAAGTACGGCGGCTTCGACAGCCCCAC
CGTGGCCTATTCTGTGCTGGTGGTGGCCAAAGTGGAAAAGGGCAAGTCCAAGAAACTGAAGAGTGT
GAAAGAGCTGCTGGGGATCACCATCATGGAAAGAAGCAGCTTCGAGAAGAATCCCATCGACTTTCTG
GAAGCCAAGGGCTACAAAGAAGTGAAAAAGGACCTGATCATCAAGCTGCCTAAGTACTCCCTGTTCG
AGCTGGAAAACGGCCGGAAGAGAATGCTGGCCTCTGCCGGCGAACTGCAGAAGGGAAACGAACTG
GCCCTGCCCTCCAAATATGTGAACTTCCTGTACCTGGCCAGCCACTATGAGAAGCTGAAGGGCTCCC
CCGAGGATAATGAGCAGAAACAGCTGTTTGTGGAACAGCACAAGCACTACCTGGACGAGATCATCGA
GCAGATCAGCGAGTTCTCCAAGAGAGTGATCCTGGCCGACGCTAATCTGGACAAAGTGCTGTCCGC
CTACAACAAGCACCGGGATAAGCCCATCAGAGAGCAGGCCGAGAATATCATCCACCTGTTTACCCTGA
CCAATCTGGGAGCCCCTGCCGCCTTCAAGTACTTTGACACCACCATCGACCGGAAGAGGTACACCAG
CACCAAAGAGGTGCTGGACGCCACCCTGATCCACCAGAGCATCACCGGCCTGTACGAGACACGGAT
CGACCTGTCTCAGCTGGGAGGTGACAGCGGCGGGAGCGGCGGGAGCGGGGGGAGCACTAATCTG
AGCGACATCATTGAGAAGGAGACTGGGAAACAGCTGGTCATTCAGGAGTCCATCCTGATGCTGCCTG
AGGAGGTGGAGGAAGTGATCGGCAACAAGCCAGAGTCTGACATCCTGGTGCACACCGCCTACGACG
AGTCCACAGATGAGAATGTGATGCTGCTGACCTCTGACGCCCCCGAGTATAAGCCTTGGGCCCTGGT
CATCCAGGATTCTAACGGCGAGAATAAGATCAAGATGCTGAGCGGAGGATCCGGAGGATCTGGAGGC
AGC*ACCAACCTGTCTGACATCATCGAGAAGGAGACAGGCAAGCAGCTGGTCATCCAGGAGAGCATC*
CTGATGCTGCCCGAAGAAGTCGAAGAAGTGATCGGAAACAAGCCTGAGAGCGATATCCTGGTCCATA
CCGCCTACGACGAGAGTACCGACGAAAATGTGATGCTGCTGACATCCGACGCCCCAGAGTATAAGCC
*CTGGGCTCTGGTCATCCAGGATTCCAACGGAGAGAACAAAATCAAAATGCTG***TCTGGCGGCTCAAAA**
AGAACCGCCGACGGCAGCGAATTCGAGCCCAAGAAGAAGAGGAAAGTCTAATTAATTAAGCTGCC
TTCTGCGGGGCTTGCCTTCTGGCCATGCCCTTCTTCTCTCCCTTGCACCTGTACCTCTTGGTCTTTGA
ATAAAGCCTGAGTAGGAAGCGACTGTGCCTTCTAGTTGCCAGCCATCTGTTGTTTGCCCCTCCCCCG
TGCCTTCCTTGACCCTGGAAGGTGCCACTCCCACTGTCCTTTCCTAATAAAATGAGAAATTGCATCG
CATTGTCTGAGTAGGTGTCATTCTATTCTGGGGGGTGGGGTGGGGCAGGACAGCAAGGGGGAGGAT
TGGGAAGACAATAGCAGGCATGCTGGGGATGCGGTGGGCTCTATGG

Start	End	Feature Description
1	380	CMV enhancer
381	619	CMV promoter
620	636	T7 promoter with mismatch

637	683	5' UTR
684	740	BP NLS
741	1424	Engineered BE4max deaminase
1425	1520	BE4max linker
1521	5621	Cas9(D10A) SpCas9 wild-type
5622	5651	Linker
5652	5900	uracil glycosylase inhibitor
5901	5930	Linker
5931	*6179*	*uracil glycosylase inhibitor*
**6180**	**6245**	**linker + SV40 BP NLS**
6246	6346	3' UTR
6347	6574	bGH poly(A) signal

Amino acid sequence
MKRTADGSEFESPKKKRKVSSETGPVAVDPTLRRRIEPHEFEVFFDPRELRKETCLLYEINWGGRHSIWR
HTSQNTNKHVEVNFIEKFTTERYFCPNTRCSITWFLSWSPCGECSRAITEFLSRYPHVTLFIYIARLYHHAD
PRNRQGLRDLISSGVTIQIMTEQESGYCWRNFVNYSPSNEAHWPRYPHLWVRLYVLELYCIILGLPPCLNI
LRRKQPQLTFFTIALQSCHYQRLPPHILWATGLKSGGSSGGSSGSETPGTSESATPESSGGSSGGSDKKY
SIGLAIGTNSVGWAVITDEYKVPSKKFKVLGNTDRHSIKKNLIGALLFDSGETAEATRLKRTARRRYTRRKN
RICYLQEIFSNEMAKVDDSFFHRLEESFLVEEDKKHERHPIFGNIVDEVAYHEKYPTIYHLRKKLVDSTDKA
DLRLIYLALAHMIKFRGHFLIEGDLNPDNSDVDKLFIQLVQTYNQLFEENPINASGVDAKAILSARLSKSRRL
ENLIAQLPGEKKNGLFGNLIALSLGLTPNFKSNFDLAEDAKLQLSKDTYDDDLDNLLAQIGDQYADLFLAAK
NLSDAILLSDILRVNTEITKAPLSASMIKRYDEHHQDLTLLKALVRQQLPEKYKEIFFDQSKNGYAGYIDGGA
SQEEFYKFIKPILEKMDGTEELLVKLNREDLLRKQRTFDNGSIPHQIHLGELHAILRRQEDFYPFLKDNREKI
EKILTFRIPYYVGPLARGNSRFAWMTRKSEETITPWNFEEVVDKGASAQSFIERMTNFDKNLPNEKVLPKH
SLLYEYFTVYNELTKVKYVTEGMRKPAFLSGEQKKAIVDLLFKTNRKVTVKQLKEDYFKKIECFDSVEISGV
EDRFNASLGTYHDLLKIIKDKDFLDNEENEDILEDIVLTLTLFEDREMIEERLKTYAHLFDDKVMKQLKRRRY
TGWGRLSRKLINGIRDKQSGKTILDFLKSDGFANRNFMQLIHDDSLTFKEDIQKAQVSGQGDSLHEHIANL
AGSPAIKKGILQTVKVVDELVKVMGRHKPENIVIEMARENQTTQKGQKNSRERMKRIEEGIKELGSQILKE
HPVENTQLQNEKLYLYYLQNGRDMYVDQELDINRLSDYDVDHIVPQSFLKDDSIDNKVLTRSDKNRGKSD
NVPSEEVVKKMKNYWRQLLNAKLITQRKFDNLTKAERGGLSELDKAGFIKRQLVETRQITKHVAQILDSRM
NTKYDENDKLIREVKVITLKSKLVSDFRKDFQFYKVREINNYHHAHDAYLNAVVGTALIKKYPKLESEFVYG
DYKVYDVRKMIAKSEQEIGKATAKYFFYSNIMNFFKTEITLANGEIRKRPLIETNGETGEIVWDKGRDFATV
RKVLSMPQVNIVKKTEVQTGGFSKESILPKRNSDKLIARKKDWDPKKYGGFDSPTVAYSVLVVAKVEKGK
SKKLKSVKELLGITIMERSSFEKNPIDFLEAKGYKEVKKDLIIKLPKYSLFELENGRKRMLASAGELQKGNEL
ALPSKYVNFLYLASHYEKLKGSPEDNEQKQLFVEQHKHYLDEIIEQISEFSKRVILADANLDKVLSAYNKHR
DKPIREQAENIIHLFTLTNLGAPAAFKYFDTTIDRKRYTSTKEVLDATLIHQSITGLYETRIDLSQLGGDSGGS
GGSGGSTNLSDIIEKETGKQLVIQESILMLPEEVEEVIGNKPESDILVHTAYDESTDENVMLLTSDAPEYKP
WALVIQDSNGENKIKMLSGGSGGSGGS*TNLSDIIEKETGKQLVIQESILMLPEEVEEVIGNKPESDILVHTAY*
*DESTDENVMLLTSDAPEYKPWALVIQDSNGENKIKML***SGGSKRTADGSEFEPKKKRKV**.

Start	End	Feature Description
1	19	BP NLS
20	247	Engineered BE4max deaminase

248	279	BE4max linker
280	1646	Cas9(D10A) SpCas9 wild-type
1647	1656	Linker
1657	1739	uracil glycosylase inhibitor
1740	1749	Linker
1750	*1832*	*uracil glycosylase inhibitor*
**1833**	**1853**	**linker + SV40 BP NLS**

YE1-BE3 – Cas9 (D10A) – SpCas9 (WT) – UGI
Plasmid ID: pHS0389

DNA sequence

GACATTGATTATTGACTAGTTATTAATAGTAATCAATTACGGGGTCATTAGTTCATAGCCCATATATGGAG
TTCCGCGTTACATAACTTACGGTAAATGGCCCGCCTGGCTGACCGCCCAACGACCCCCGCCCATTGA
CGTCAATAATGACGTATGTTCCCATAGTAACGCCAATAGGGACTTTCCATTGACGTCAATGGGTGGAGT
ATTTACGGTAAACTGCCCACTTGGCAGTACATCAAGTGTATCATATGCCAAGTACGCCCCCTATTGACG
TCAATGACGGTAAATGGCCCGCCTGGCATTATGCCCAGTACATGACCTTATGGGACTTTCCTACTTGG
CAGTACATCTACGTATTAGTCATCGCTATTACCATGGTGATGCGGTTTTGGCAGTACATCAATGGGCGT
GGATAGCGGTTTGACTCACGGGGATTTCCAAGTCTCCACCCCATTGACGTCAATGGGAGTTTGTTTTG
GCACCAAAATCAACGGGACTTTCCAAAATGTCGTAACAACTCCGCCCCATTGACGCAAATGGGCGGT
AGGCGTGTACGGTGGGAGGTCTATATAAGCAGAGCTGGTTTAGTGAACCGTCAGATCTCGAGCTCGG
TACCTAATACGACACACTATAAGGAAATAAGAGAGAAAAGAAGAGTAAGAAGAAATATAAGAGCCACCA
TGAAACGGACAGCCGACGGAAGCGAGTTCGAGTCACCAAAGAAGAAGCGGAAAGTCTCCTCAGAGA
CTGGGCCTGTCGCCGTCGATCCAACCCTGCGCCGCCGGATTGAACCTCACGAGTTTGAAGTGTTCTT
TGACCCCCGGGAGCTGAGAAAGGAGACATGCCTGCTGTACGAGATCAACTGGGGAGGCAGGCACTC
CATCTGGAGGCACACCTCTCAGAACACAAATAAGCACGTGGAGGTGAACTTCATCGAGAAGTTTACC
ACAGAGCGGTACTTCTGCCCCAATACCAGATGTAGCATCACATGGTTTCTGAGCTATTCCCCTTGCGG
AGAGTGTAGCAGGGCCATCACCGAGTTCCTGTCCAGATATCCACACGTGACACTGTTTATCTACATCG
CCAGGCTGTATCACCACGCAGACCCAGAGAATAGGCAGGGCCTGCGCGATCTGATCAGCTCCGGCG
TGACCATCCAGATCATGACAGAGCAGGAGTCCGGCTACTGCTGGCGGAACTTCGTGAATTATTCTCCT
AGCAACGAGGCCCACTGGCCTAGGTACCCACACCTGTGGGTGCGCCTGTACGTGCTGGAGCTGTAT
TGCATCATCCTGGGCCTGCCCCCTTGTCTGAATATCCTGCGGAGAAAGCAGCCCCAGCTGACCTTCT
TTACAATCGCCCTGCAGTCTTGTCACTATCAGAGGCTGCCACCCCACATCCTGTGGGCCACAGGCCT
GAAGTCTGGAGGATCTAGCGGAGGATCCTCTGGCAGCGAGACACCAGGAACAAGCGAGTCAGCAAC
ACCAGAGAGCAGTGGCGGCAGCAGCGGCGGCAGCGACAAGAAGTACAGCATCGGCCTGGCCATCG
GCACCAACTCTGTGGGCTGGGCCGTGATCACCGACGAGTACAAGGTGCCCAGCAAGAAATTCAAGG
TGCTGGGCAACACCGACCGGCACAGCATCAAGAAGAACCTGATCGGAGCCCTGCTGTTCGACAGCG
GCGAAACAGCCGAGGCCACCCGGCTGAAGAGAACCGCCAGAAGAAGATACACCAGACGGAAGAAC
CGGATCTGCTATCTGCAAGAGATCTTCAGCAACGAGATGGCCAAGGTGGACGACAGCTTCTTCCACA
GACTGGAAGAGTCCTTCCTGGTGGAAGAGGATAAGAAGCACGAGCGGCACCCCATCTTCGGCAACA
TCGTGGACGAGGTGGCCTACCACGAGAAGTACCCCACCATCTACCACCTGAGAAAGAAACTGGTGG
ACAGCACCGACAAGGCCGACCTGCGGCTGATCTATCTGGCCCTGGCCCACATGATCAAGTTCCGGG
GCCACTTCCTGATCGAGGGCGACCTGAACCCCGACAACAGCGACGTGGACAAGCTGTTCATCCAGC
TGGTGCAGACCTACAACCAGCTGTTCGAGGAAAACCCCATCAACGCCAGCGGCGTGGACGCCAAGG
CCATCCTGTCTGCCAGACTGAGCAAGAGCAGACGGCTGGAAAATCTGATCGCCCAGCTGCCCGGCG
AGAAGAAGAATGGCCTGTTCGGAAACCTGATTGCCCTGAGCCTGGGCCTGACCCCCAACTTCAAGA
GCAACTTCGACCTGGCCGAGGATGCCAAACTGCAGCTGAGCAAGGACACCTACGACGACGACCTGG
ACAACCTGCTGGCCCAGATCGGCGACCAGTACGCCGACCTGTTTCTGGCCGCCAAGAACCTGTCCG
ACGCCATCCTGCTGAGCGACATCCTGAGAGTGAACACCGAAATCACCAAGGCCCCCCTGAGCGCCT
CTATGATCAAGAGATACGACGAGCACCACCAGGACCTGACCCTGCTGAAAGCTCTCGTGCGGCAGCA
GCTGCCTGAGAAGTACAAAGAGATTTTCTTCGACCAGAGCAAGAACGGCTACGCCGGCTACATTGAC
GGCGGAGCCAGCCAGGAAGAGTTCTACAAGTTCATCAAGCCCATCCTGGAAAAGATGGACGGCACC
GAGGAACTGCTCGTGAAGCTGAACAGAGAGGACCTGCTGCGGAAGCAGCGGACCTTCGACAACGG
CAGCATCCCCCACCAGATCCACCTGGGAGAGCTGCACGCCATTCTGCGGCGGCAGGAAGATTTTTAC
CCATTCCTGAAGGACAACCGGGAAAAGATCGAGAAGATCCTGACCTTCCGCATTCCCTACTACGTGG
GCCCTCTGGCCAGGGGAAACAGCAGATTCGCCTGGATGACCAGAAAGAGCGAGGAAACCATCACCC
CCTGGAACTTCGAGGAAGTGGTGGACAAGGGCGCTTCCGCCCAGAGCTTCATCGAGCGGATGACCA
ACTTCGATAAGAACCTGCCCAACGAGAAGGTGCTGCCCAAGCACAGCCTGCTGTACGAGTACTTCAC
CGTGTATAACGAGCTGACCAAAGTGAAATACGTGACCGAGGGAATGAGAAAGCCCGCCTTCCTGAGC
GGCGAGCAGAAAAAGGCCATCGTGGACCTGCTGTTCAAGACCAACCGGAAAGTGACCGTGAAGCAG
CTGAAAGAGGACTACTTCAAGAAAATCGAGTGCTTCGACTCCGTGGAAATCTCCGGCGTGGAAGATC
GGTTCAACGCCTCCCTGGGCACATACCACGATCTGCTGAAAATTATCAAGGACAAGGACTTCCTGGA
CAATGAGGAAAACGAGGACATTCTGGAAGATATCGTGCTGACCCTGACACTGTTTGAGGACAGAGAG
ATGATCGAGGAACGGCTGAAAACCTATGCCCACCTGTTCGACGACAAAGTGATGAAGCAGCTGAAGC

GGCGGAGATACACCGGCTGGGGCAGGCTGAGCCGGAAGCTGATCAACGGCATCCGGGACAAGCAG
TCCGGCAAGACAATCCTGGATTTCCTGAAGTCCGACGGCTTCGCCAACAGAAACTTCATGCAGCTGA
TCCACGACGACAGCCTGACCTTTAAAGAGGACATCCAGAAAGCCCAGGTGTCCGGCCAGGGCGATA
GCCTGCACGAGCACATTGCCAATCTGGCCGGCAGCCCCGCCATTAAGAAGGGCATCCTGCAGACAG
TGAAGGTGGTGGACGAGCTCGTGAAAGTGATGGGCCGGCACAAGCCCGAGAACATCGTGATCGAAA
TGGCCAGAGAGAACCAGACCACCCAGAAGGGACAGAAGAACAGCCGCGAGAGAATGAAGCGGATC
GAAGAGGGCATCAAAGAGCTGGGCAGCCAGATCCTGAAAGAACACCCCGTGGAAAACACCCAGCTG
CAGAACGAGAAGCTGTACCTGTACTACCTGCAGAATGGGCGGGATATGTACGTGGACCAGGAACTGG
ACATCAACCGGCTGTCCGACTACGATGTGGACCATATCGTGCCTCAGAGCTTTCTGAAGGACGACTC
CATCGACAACAAGGTGCTGACCAGAAGCGACAAGAACCGGGGCAAGAGCGACAACGTGCCCTCCG
AAGAGGTCGTGAAGAAGATGAAGAACTACTGGCGGCAGCTGCTGAACGCCAAGCTGATTACCCAGA
GAAAGTTCGACAATCTGACCAAGGCCGAGAGAGGCGGCCTGAGCGAACTGGATAAGGCCGGCTTCA
TCAAGAGACAGCTGGTGGAAACCCGGCAGATCACAAAGCACGTGGCACAGATCCTGGACTCCCGGA
TGAACACTAAGTACGACGAGAATGACAAGCTGATCCGGGAAGTGAAAGTGATCACCCTGAAGTCCAA
GCTGGTGTCCGATTTCCGGAAGGATTTCCAGTTTTACAAAGTGCGCGAGATCAACAACTACCACCAC
GCCCACGACGCCTACCTGAACGCCGTCGTGGGAACCGCCCTGATCAAAAAGTACCCTAAGCTGGAA
AGCGAGTTCGTGTACGGCGACTACAAGGTGTACGACGTGCGGAAGATGATCGCCAAGAGCGAGCAG
GAAATCGGCAAGGCTACCGCCAAGTACTTCTTCTACAGCAACATCATGAACTTTTTCAAGACCGAGAT
TACCCTGGCCAACGGCGAGATCCGGAAGCGGCCTCTGATCGAGACAAACGGCGAAACCGGGGAGA
TCGTGTGGGATAAGGGCCGGGATTTTGCCACCGTGCGGAAAGTGCTGAGCATGCCCCAAGTGAATAT
CGTGAAAAAGACCGAGGTGCAGACAGGCGGCTTCAGCAAAGAGTCTATCCTGCCCAAGAGGAACAG
CGATAAGCTGATCGCCAGAAAGAAGGACTGGGACCCTAAGAAGTACGGCGGCTTCGACAGCCCCAC
CGTGGCCTATTCTGTGCTGGTGGTGGCCAAAGTGGAAAAGGGCAAGTCCAAGAAACTGAAGAGTGT
GAAAGAGCTGCTGGGGATCACCATCATGGAAAGAAGCAGCTTCGAGAAGAATCCCATCGACTTTCTG
GAAGCCAAGGGCTACAAAGAAGTGAAAAAGGACCTGATCATCAAGCTGCCTAAGTACTCCCTGTTCG
AGCTGGAAAACGGCCGGAAGAGAATGCTGGCCTCTGCCGGCGAACTGCAGAAGGGAAACGAACTG
GCCCTGCCCTCCAAATATGTGAACTTCCTGTACCTGGCCAGCCACTATGAGAAGCTGAAGGGCTCCC
CCGAGGATAATGAGCAGAAACAGCTGTTTGTGGAACAGCACAAGCACTACCTGGACGAGATCATCGA
GCAGATCAGCGAGTTCTCCAAGAGAGTGATCCTGGCCGACGCTAATCTGGACAAAGTGCTGTCCGC
CTACAACAAGCACCGGGATAAGCCCATCAGAGAGCAGGCCGAGAATATCATCCACCTGTTTACCCTGA
CCAATCTGGGAGCCCCTGCCGCCTTCAAGTACTTTGACACCACCATCGACCGGAAGAGGTACACCAG
CACCAAAGAGGTGCTGGACGCCACCCTGATCCACCAGAGCATCACCGGCCTGTACGAGACACGGAT
CGACCTGTCTCAGCTGGGAGGTGACAGCGGCGGGAGCGGCGGGAGCGGGGGGAGCACTAATCTG
AGCGACATCATTGAGAAGGAGACTGGGAAACAGCTGGTCATTCAGGAGTCCATCCTGATGCTGCCTG
AGGAGGTGGAGGAAGTGATCGGCAACAAGCCAGAGTCTGACATCCTGGTGCACACCGCCTACGACG
AGTCCACAGATGAGAATGTGATGCTGCTGACCTCTGACGCCCCCGAGTATAAGCCTTGGGCCCTGGT
CATCCAGGATTCTAACGGCGAGAATAAGATCAAGATGCTGAGCGGAGGATCCGGAGGATCTGGAGGC
AGC*ACCAACCTGTCTGACATCATCGAGAAGGAGACAGGCAAGCAGCTGGTCATCCAGGAGAGCATC*
CTGATGCTGCCCGAAGAAGTCGAAGAAGTGATCGGAAACAAGCCTGAGAGCGATATCCTGGTCCATA
CCGCCTACGACGAGAGTACCGACGAAAATGTGATGCTGCTGACATCCGACGCCCCAGAGTATAAGCC
*CTGGGCTCTGGTCATCCAGGATTCCAACGGAGAGAACAAAATCAAAATGCTG***TCTGGCGGCTCAAAA**
AGAACCGCCGACGGCAGCGAATTCGAGCCCAAGAAGAAGAGGAAAGTCTAATTAATTAAGCTGCC
TTCTGCGGGGCTTGCCTTCTGGCCATGCCCTTCTTCTCTCCCTTGCACCTGTACCTCTTGGTCTTTGA
ATAAAGCCTGAGTAGGAAGCGACTGTGCCTTCTAGTTGCCAGCCATCTGTTGTTTGCCCCTCCCCCG
TGCCTTCCTTGACCCTGGAAGGTGCCACTCCCACTGTCCTTTCCTAATAAAATGAGAAATTGCATCG
CATTGTCTGAGTAGGTGTCATTCTATTCTGGGGGGTGGGGTGGGGCAGGACAGCAAGGGGGAGGAT
TGGGAAGACAATAGCAGGCATGCTGGGGATGCGGTGGGCTCTATGG

Start	End	Feature Description
1	380	CMV enhancer
381	619	CMV promoter
620	636	T7 promoter with mismatch

637	683	5' UTR
684	740	BP NLS
741	1424	YE1 deaminase
1425	1520	BE4max linker
1521	5621	Cas9(D10A) SpCas9 wild-type
5622	5651	Linker
5652	5900	uracil glycosylase inhibitor
5901	5930	Linker
5931	*6179*	*uracil glycosylase inhibitor*
**6180**	**6245**	**linker + SV40 BP NLS**
6246	6346	3' UTR
6347	6574	bGH poly(A) signal

Amino acid sequence
MKRTADGSEFESPKKKRKVSSETGPVAVDPTLRRRIEPHEFEVFFDPRELRKETCLLYEINWGGRHSIWR
HTSQNTNKHVEVNFIEKFTTERYFCPNTRCSITWFLSYSPCGECSRAITEFLSRYPHVTLFIYIARLYHHAD
PENRQGLRDLISSGVTIQIMTEQESGYCWRNFVNYSPSNEAHWPRYPHLWVRLYVLELYCIILGLPPCLNI
LRRKQPQLTFFTIALQSCHYQRLPPHILWATGLKSGGSSGGSSGSETPGTSESATPESSGGSSGGSDKKY
SIGLAIGTNSVGWAVITDEYKVPSKKFKVLGNTDRHSIKKNLIGALLFDSGETAEATRLKRTARRRYTRRKN
RICYLQEIFSNEMAKVDDSFFHRLEESFLVEEDKKHERHPIFGNIVDEVAYHEKYPTIYHLRKKLVDSTDKA
DLRLIYLALAHMIKFRGHFLIEGDLNPDNSDVDKLFIQLVQTYNQLFEENPINASGVDAKAILSARLSKSRRL
ENLIAQLPGEKKNGLFGNLIALSLGLTPNFKSNFDLAEDAKLQLSKDTYDDDLDNLLAQIGDQYADLFLAAK
NLSDAILLSDILRVNTEITKAPLSASMIKRYDEHHQDLTLLKALVRQQLPEKYKEIFFDQSKNGYAGYIDGGA
SQEEFYKFIKPILEKMDGTEELLVKLNREDLLRKQRTFDNGSIPHQIHLGELHAILRRQEDFYPFLKDNREKI
EKILTFRIPYYVGPLARGNSRFAWMTRKSEETITPWNFEEVVDKGASAQSFIERMTNFDKNLPNEKVLPKH
SLLYEYFTVYNELTKVKYVTEGMRKPAFLSGEQKKAIVDLLFKTNRKVTVKQLKEDYFKKIECFDSVEISGV
EDRFNASLGTYHDLLKIIKDKDFLDNEENEDILEDIVLTLTLFEDREMIEERLKTYAHLFDDKVMKQLKRRRY
TGWGRLSRKLINGIRDKQSGKTILDFLKSDGFANRNFMQLIHDDSLTFKEDIQKAQVSGQGDSLHEHIANL
AGSPAIKKGILQTVKVVDELVKVMGRHKPENIVIEMARENQTTQKGQKNSRERMKRIEEGIKELGSQILKE
HPVENTQLQNEKLYLYYLQNGRDMYVDQELDINRLSDYDVDHIVPQSFLKDDSIDNKVLTRSDKNRGKSD
NVPSEEVVKKMKNYWRQLLNAKLITQRKFDNLTKAERGGLSELDKAGFIKRQLVETRQITKHVAQILDSRM
NTKYDENDKLIREVKVITLKSKLVSDFRKDFQFYKVREINNYHHAHDAYLNAVVGTALIKKYPKLESEFVYG
DYKVYDVRKMIAKSEQEIGKATAKYFFYSNIMNFFKTEITLANGEIRKRPLIETNGETGEIVWDKGRDFATV
RKVLSMPQVNIVKKTEVQTGGFSKESILPKRNSDKLIARKKDWDPKKYGGFDSPTVAYSVLVVAKVEKGK
SKKLKSVKELLGITIMERSSFEKNPIDFLEAKGYKEVKKDLIIKLPKYSLFELENGRKRMLASAGELQKGNEL
ALPSKYVNFLYLASHYEKLKGSPEDNEQKQLFVEQHKHYLDEIIEQISEFSKRVILADANLDKVLSAYNKHR
DKPIREQAENIIHLFTLTNLGAPAAFKYFDTTIDRKRYTSTKEVLDATLIHQSITGLYETRIDLSQLGGDSGGS
GGSGGSTNLSDIIEKETGKQLVIQESILMLPEEVEEVIGNKPESDILVHTAYDESTDENVMLLTSDAPEYKP
WALVIQDSNGENKIKMLSGGSGGSGGS*TNLSDIIEKETGKQLVIQESILMLPEEVEEVIGNKPESDILVHTAY*
*DESTDENVMLLTSDAPEYKPWALVIQDSNGENKIKML***SGGSKRTADGSEFEPKKKRKV**.

Start	End	Feature Description
1	19	BP NLS
20	247	YE1 deaminase

248	279	BE4max linker
280	1646	Cas9(D10A) SpCas9 wild-type
1647	1656	Linker
1657	1739	uracil glycosylase inhibitor
1740	1749	Linker
1750	*1832*	*uracil glycosylase inhibitor*
**1833**	**1853**	**linker + SV40 BP NLS**

TadCBEd – Cas9 (D10A) – SpCas9 (WT) – UGI
Plasmid ID: pHS0387

DNA sequence
GACATTGATTATTGACTAGTTATTAATAGTAATCAATTACGGGGTCATTAGTTCATAGCCCATATATGGAG
TTCCGCGTTACATAACTTACGGTAAATGGCCCGCCTGGCTGACCGCCCAACGACCCCCGCCCATTGA
CGTCAATAATGACGTATGTTCCCATAGTAACGCCAATAGGGACTTTCCATTGACGTCAATGGGTGGAGT
ATTTACGGTAAACTGCCCACTTGGCAGTACATCAAGTGTATCATATGCCAAGTACGCCCCCTATTGACG
TCAATGACGGTAAATGGCCCGCCTGGCATTATGCCCAGTACATGACCTTATGGGACTTTCCTACTTGG
CAGTACATCTACGTATTAGTCATCGCTATTACCATGGTGATGCGGTTTTGGCAGTACATCAATGGGCGT
GGATAGCGGTTTGACTCACGGGGATTTCCAAGTCTCCACCCCATTGACGTCAATGGGAGTTTGTTTTG
GCACCAAAATCAACGGGACTTTCCAAAATGTCGTAACAACTCCGCCCCATTGACGCAAATGGGCGGT
AGGCGTGTACGGTGGGAGGTCTATATAAGCAGAGCTGGTTTAGTGAACCGTCAGATCTCGAGCTCGG
TACCTAATACGACACACTATAAGGAAATAAGAGAGAAAAGAAGAGTAAGAAGAAATATAAGAGCCACCA
TGAAACGGACAGCCGACGGAAGCGAGTTCGAGTCACCAAAGAAGAAGCGGAAAGTCAGTTCTGAGG
TGGAGTTTTCCCACGAGTACTGGATGAGACATGCCCTGACCCTGGCCAAGAGGGCACGGGATGAGA
GGAAGGCGCCTGTGGGAGCCGTGCTGGTGCTGAACAATAGAGTGATCGGCGAGGGCTGGAACAGA
GCCATCGGCCTGCACGACCCAACAGCCCATGCCGAAATTATAGCCCTGAGACAGGGCGGCCTGGTC
ATGCAGAACTACAGACTGATTGACGCCACCCTGTACGTGACATTCGAGCCTTGCGTGATGTGCGCCG
GCGCCATGATCAACTCTAGGATCGGCCGCGTGGTGTTTGGCGTGAGGAACTCAAAAAGAGGCGCCG
CAGGCTCCCTGATGAACGTGCTGAACTACCCCGGAATGAATCACCGCGTCGAAATTACCGAGGGAAT
CCTGGCAGATGAATGTGCCGCCCTGCTGTGCGATTTCTATCGGATGCCTAGACAGGTGTTCAATGCTC
AGAAGAAGGCCCAGAGCTCCATCAACTCTGGCGGATCTAGCGGAGGATCCTCTGGCAGCGAGACAC
CAGGAACAAGCGAGTCAGCAACACCAGAGAGCAGTGGCGGCAGCAGCGGCGGCAGCGACAAGAA
GTACAGCATCGGCCTGGCCATCGGCACCAACTCTGTGGGCTGGGCCGTGATCACCGACGAGTACAA
GGTGCCCAGCAAGAAATTCAAGGTGCTGGGCAACACCGACCGGCACAGCATCAAGAAGAACCTGAT
CGGAGCCCTGCTGTTCGACAGCGGCGAAACAGCCGAGGCCACCCGGCTGAAGAGAACCGCCAGAA
GAAGATACACCAGACGGAAGAACCGGATCTGCTATCTGCAAGAGATCTTCAGCAACGAGATGGCCAA
GGTGGACGACAGCTTCTTCCACAGACTGGAAGAGTCCTTCCTGGTGGAAGAGGATAAGAAGCACGA
GCGGCACCCCATCTTCGGCAACATCGTGGACGAGGTGGCCTACCACGAGAAGTACCCCACCATCTA
CCACCTGAGAAAGAAACTGGTGGACAGCACCGACAAGGCCGACCTGCGGCTGATCTATCTGGCCCT
GGCCCACATGATCAAGTTCCGGGGCCACTTCCTGATCGAGGGCGACCTGAACCCCGACAACAGCGA
CGTGGACAAGCTGTTCATCCAGCTGGTGCAGACCTACAACCAGCTGTTCGAGGAAAACCCCATCAAC
GCCAGCGGCGTGGACGCCAAGGCCATCCTGTCTGCCAGACTGAGCAAGAGCAGACGGCTGGAAAA
TCTGATCGCCCAGCTGCCCGGCGAGAAGAAGAATGGCCTGTTCGGAAACCTGATTGCCCTGAGCCT
GGGCCTGACCCCCAACTTCAAGAGCAACTTCGACCTGGCCGAGGATGCCAAACTGCAGCTGAGCAA
GGACACCTACGACGACGACCTGGACAACCTGCTGGCCCAGATCGGCGACCAGTACGCCGACCTGTT
TCTGGCCGCCAAGAACCTGTCCGACGCCATCCTGCTGAGCGACATCCTGAGAGTGAACACCGAGAT
CACCAAGGCCCCCCTGAGCGCCTCTATGATCAAGAGATACGACGAGCACCACCAGGACCTGACCCT
GCTGAAAGCTCTCGTGCGGCAGCAGCTGCCTGAGAAGTACAAAGAGATTTTCTTCGACCAGAGCAAG
AACGGCTACGCCGGCTACATTGACGGCGGAGCCAGCCAGGAAGAGTTCTACAAGTTCATCAAGCCC
ATCCTGGAAAAGATGGACGGCACCGAGGAACTGCTCGTGAAGCTGAACAGAGAGGACCTGCTGCGG
AAGCAGCGGACCTTCGACAACGGCAGCATCCCCCACCAGATCCACCTGGGAGAGCTGCACGCCATT
CTGCGGCGGCAGGAAGATTTTTACCCATTCCTGAAGGACAACCGGGAAAAGATCGAGAAGATCCTGA
CCTTCCGCATCCCCTACTACGTGGGCCCTCTGGCCAGGGGAAACAGCAGATTCGCCTGGATGACCA
GAAAGAGCGAGGAAACCATCACCCCCTGGAACTTCGAGGAAGTGGTGGACAAGGGCGCTTCCGCC
CAGAGCTTCATCGAGCGGATGACCAACTTCGATAAGAACCTGCCCAACGAGAAGGTGCTGCCCAAG
CACAGCCTGCTGTACGAGTACTTCACCGTGTATAACGAGCTGACCAAAGTGAAATACGTGACCGAGG
GAATGAGAAAGCCCGCCTTCCTGAGCGGCGAGCAGAAAAAGGCCATCGTGGACCTGCTGTTCAAGA
CCAACCGGAAAGTGACCGTGAAGCAGCTGAAAGAGGACTACTTCAAGAAAATCGAGTGCTTCGACTC
CGTGGAAATCTCCGGCGTGGAAGATCGGTTCAACGCCTCCCTGGGCACATACCACGATCTGCTGAAA
ATTATCAAGGACAAGGACTTCCTGGACAATGAGGAAAACGAGGACATTCTGGAAGATATCGTGCTGAC
CCTGACACTGTTTGAGGACAGAGAGATGATCGAGGAACGGCTGAAAACCTATGCCCACCTGTTCGAC
GACAAAGTGATGAAGCAGCTGAAGCGGCGGAGATACACCGGCTGGGGCAGGCTGAGCCGGAAGCT
GATCAACGGCATCCGGGACAAGCAGTCCGGCAAGACAATCCTGGATTTCCTGAAGTCCGACGGCTT
CGCCAACAGAAACTTCATGCAGCTGATCCACGACGACAGCCTGACCTTTAAAGAGGACATCCAGAAA

GCCCAGGTGTCCGGCCAGGGCGATAGCCTGCACGAGCACATTGCCAATCTGGCCGGCAGCCCCGC
CATTAAGAAGGGCATCCTGCAGACAGTGAAGGTGGTGGACGAGCTCGTGAAAGTGATGGGCCGGCA
CAAGCCCGAGAACATCGTGATCGAAATGGCCAGAGAGAACCAGACCACCCAGAAGGGACAGAAGAA
CAGCCGCGAGAGAATGAAGCGGATCGAAGAGGGCATCAAAGAGCTGGGCAGCCAGATCCTGAAAGA
ACACCCCGTGGAAAACACCCAGCTGCAGAACGAGAAGCTGTACCTGTACTACCTGCAGAATGGGCG
GGATATGTACGTGGACCAGGAACTGGACATCAACCGGCTGTCCGACTACGATGTGGACCATATCGTG
CCTCAGAGCTTTCTGAAGGACGACTCCATCGACAACAAGGTGCTGACCAGAAGCGACAAGAACCGG
GGCAAGAGCGACAACGTGCCCTCCGAAGAGGTCGTGAAGAAGATGAAGAACTACTGGCGGCAGCT
GCTGAACGCCAAGCTGATTACCCAGAGAAAGTTCGACAATCTGACCAAGGCCGAGAGAGGCGGCCT
GAGCGAACTGGATAAGGCCGGCTTCATCAAGAGACAGCTGGTGGAAACCCGGCAGATCACAAAGCA
CGTGGCACAGATCCTGGACTCCCGGATGAACACTAAGTACGACGAGAATGACAAGCTGATCCGGGAA
GTGAAAGTGATCACCCTGAAGTCCAAGCTGGTGTCCGATTTCCGGAAGGATTTCCAGTTTTACAAAGT
GCGCGAGATCAACAACTACCACCACGCCCACGACGCCTACCTGAACGCCGTCGTGGGAACCGCCCT
GATCAAAAAGTACCCTAAGCTGGAAAGCGAGTTCGTGTACGGCGACTACAAGGTGTACGACGTGCGG
AAGATGATCGCCAAGAGCGAGCAGGAAATCGGCAAGGCTACCGCCAAGTACTTCTTCTACAGCAACA
TCATGAACTTTTTCAAGACCGAGATTACCCTGGCCAACGGCGAGATCCGGAAGCGGCCTCTGATCGA
GACAAACGGCGAAACCGGGGAGATCGTGTGGGATAAGGGCCGGGATTTTGCCACCGTGCGGAAAGT
GCTGAGCATGCCCCAAGTGAATATCGTGAAAAAGACCGAGGTGCAGACAGGCGGCTTCAGCAAAGA
GTCTATCCTGCCCAAGAGGAACAGCGATAAGCTGATCGCCAGAAAGAAGGACTGGGACCCTAAGAAG
TACGGCGGCTTCGACAGCCCCACCGTGGCCTATTCTGTGCTGGTGGTGGCCAAAGTGGAAAAGGGC
AAGTCCAAGAAACTGAAGAGTGTGAAAGAGCTGCTGGGGATCACCATCATGGAAAGAAGCAGCTTCG
AGAAGAATCCCATCGACTTTCTGGAAGCCAAGGGCTACAAAGAAGTGAAAAAGGACCTGATCATCAA
GCTGCCTAAGTACTCCCTGTTCGAGCTGGAAAACGGCCGGAAGAGAATGCTGGCCTCTGCCGGCGA
ACTGCAGAAGGGAAACGAACTGGCCCTGCCCTCCAAATATGTGAACTTCCTGTACCTGGCCAGCCAC
TATGAGAAGCTGAAGGGCTCCCCCGAGGATAATGAGCAGAAACAGCTGTTTGTGGAACAGCACAAGC
ACTACCTGGACGAGATCATCGAGCAGATCAGCGAGTTCTCCAAGAGAGTGATCCTGGCCGACGCTAA
TCTGGACAAAGTGCTGTCCGCCTACAACAAGCACCGGGATAAGCCCATCAGAGAGCAGGCCGAGAA
TATCATCCACCTGTTTACCCTGACCAATCTGGGAGCCCCTGCCGCCTTCAAGTACTTTGACACCACCA
TCGACCGGAAGAGGTACACCAGCACCAAAGAGGTGCTGGACGCCACCCTGATCCACCAGAGCATCA
CCGGCCTGTACGAGACACGGATCGACCTGTCTCAGCTGGGAGGTGACAGCGGCGGGAGCGGCGG
GAGCGGGGGGAGCACTAATCTGAGCGACATCATTGAGAAGGAGACTGGGAAACAGCTGGTCATTCA
GGAGTCCATCCTGATGCTGCCTGAGGAGGTGGAGGAAGTGATCGGCAACAAGCCAGAGTCTGACAT
CCTGGTGCACACCGCCTACGACGAGTCCACAGATGAGAATGTGATGCTGCTGACCTCTGACGCCCC
CGAGTATAAGCCTTGGGCCCTGGTCATCCAGGATTCTAACGGCGAGAATAAGATCAAGATGCTGAGC
GGAGGATCCGGAGGATCTGGAGGCAGC*ACCAACCTGTCTGACATCATCGAGAAGGAGACAGGCAAG*
CAGCTGGTCATCCAGGAGAGCATCCTGATGCTGCCCGAAGAAGTCGAAGAAGTGATCGGAAACAAG
CCTGAGAGCGATATCCTGGTCCATACCGCCTACGACGAGAGTACCGACGAAAATGTGATGCTGCTGA
CATCCGACGCCCCAGAGTATAAGCCCTGGGCTCTGGTCATCCAGGATTCCAACGGAGAGAACAAAAT
*CAAAATGCTG***TCTGGCGGCTCAAAAAGAACCGCCGACGGCAGCGAATTCGAGCCCAAGAAGAAGA**
GGAAAGTCTAATTAATTAAGCTGCCTTCTGCGGGGCTTGCCTTCTGGCCATGCCCTTCTTCTCTCCCT
TGCACCTGTACCTCTTGGTCTTTGAATAAAGCCTGAGTAGGAAGCGACTGTGCCTTCTAGTTGCCAGC
CATCTGTTGTTTGCCCCTCCCCCGTGCCTTCCTTGACCCTGGAAGGTGCCACTCCCACTGTCCTTTC
CTAATAAAATGAGAAAATTGCATCGCATTGTCTGAGTAGGTGTCATTCTATTCTGGGGGGTGGGGTGG
GGCAGGACAGCAAGGGGGAGGATTGGGAAGACAATAGCAGGCATGCTGGGGATGCGGTGGGCTCT
ATGG

Start	End	Feature Description
1	380	CMV enhancer
381	619	CMV promoter
620	636	T7 promoter with mismatch
637	683	5' UTR

684	740	BP NLS
741	1241	TadCBEd deaminase
1242	1337	linker
1338	5438	Cas9(D10A) SpCas9 wild-type
5439	5468	Linker
5469	5717	uracil glycosylase inhibitor
5718	5747	Linker
5748	*5996*	*uracil glycosylase inhibitor*
**5997**	**6062**	**linker + SV40 BP NLS**
6063	6163	3’ UTR
6164	6391	bGH poly(A) signal

Amino acid sequence
MKRTADGSEFESPKKKRKVSSEVEFSHEYWMRHALTLAKRARDERKAPVGAVLVLNNRVIGEGWNRAIG
LHDPTAHAEIIALRQGGLVMQNYRLIDATLYVTFEPCVMCAGAMINSRIGRVVFGVRNSKRGAAGSLMNVL
NYPGMNHRVEITEGILADECAALLCDFYRMPRQVFNAQKKAQSSINSGGSSGGSSGSETPGTSESATPE
SSGGSSGGSDKKYSIGLAIGTNSVGWAVITDEYKVPSKKFKVLGNTDRHSIKKNLIGALLFDSGETAEATRL
KRTARRRYTRRKNRICYLQEIFSNEMAKVDDSFFHRLEESFLVEEDKKHERHPIFGNIVDEVAYHEKYPTIY
HLRKKLVDSTDKADLRLIYLALAHMIKFRGHFLIEGDLNPDNSDVDKLFIQLVQTYNQLFEENPINASGVDA
KAILSARLSKSRRLENLIAQLPGEKKNGLFGNLIALSLGLTPNFKSNFDLAEDAKLQLSKDTYDDDLDNLLA
QIGDQYADLFLAAKNLSDAILLSDILRVNTEITKAPLSASMIKRYDEHHQDLTLLKALVRQQLPEKYKEIFFD
QSKNGYAGYIDGGASQEEFYKFIKPILEKMDGTEELLVKLNREDLLRKQRTFDNGSIPHQIHLGELHAILRR
QEDFYPFLKDNREKIEKILTFRIPYYVGPLARGNSRFAWMTRKSEETITPWNFEEVVDKGASAQSFIERMT
NFDKNLPNEKVLPKHSLLYEYFTVYNELTKVKYVTEGMRKPAFLSGEQKKAIVDLLFKTNRKVTVKQLKED
YFKKIECFDSVEISGVEDRFNASLGTYHDLLKIIKDKDFLDNEENEDILEDIVLTLTLFEDREMIEERLKTYAH
LFDDKVMKQLKRRRYTGWGRLSRKLINGIRDKQSGKTILDFLKSDGFANRNFMQLIHDDSLTFKEDIQKAQ
VSGQGDSLHEHIANLAGSPAIKKGILQTVKVVDELVKVMGRHKPENIVIEMARENQTTQKGQKNSRERMK
RIEEGIKELGSQILKEHPVENTQLQNEKLYLYYLQNGRDMYVDQELDINRLSDYDVDHIVPQSFLKDDSIDN
KVLTRSDKNRGKSDNVPSEEVVKKMKNYWRQLLNAKLITQRKFDNLTKAERGGLSELDKAGFIKRQLVET
RQITKHVAQILDSRMNTKYDENDKLIREVKVITLKSKLVSDFRKDFQFYKVREINNYHHAHDAYLNAVVGTA
LIKKYPKLESEFVYGDYKVYDVRKMIAKSEQEIGKATAKYFFYSNIMNFFKTEITLANGEIRKRPLIETNGET
GEIVWDKGRDFATVRKVLSMPQVNIVKKTEVQTGGFSKESILPKRNSDKLIARKKDWDPKKYGGFDSPTV
AYSVLVVAKVEKGKSKKLKSVKELLGITIMERSSFEKNPIDFLEAKGYKEVKKDLIIKLPKYSLFELENGRKR
MLASAGELQKGNELALPSKYVNFLYLASHYEKLKGSPEDNEQKQLFVEQHKHYLDEIIEQISEFSKRVILAD
ANLDKVLSAYNKHRDKPIREQAENIIHLFTLTNLGAPAAFKYFDTTIDRKRYTSTKEVLDATLIHQSITGLYET
RIDLSQLGGDSGGSGGSGGSTNLSDIIEKETGKQLVIQESILMLPEEVEEVIGNKPESDILVHTAYDESTDE
NVMLLTSDAPEYKPWALVIQDSNGENKIKMLSGGSGGSGGS*TNLSDIIEKETGKQLVIQESILMLPEEVEEV*
*IGNKPESDILVHTAYDESTDENVMLLTSDAPEYKPWALVIQDSNGENKIKML***SGGSKRTADGSEFEPKKK**
RKV

Start	End	Feature Description
1	19	BP NLS
20	186	TadCBEd deaminase
187	218	BE4max linker

219	1585	Cas9(D10A) SpCas9 wild-type
1586	1595	Linker
1596	1678	uracil glycosylase inhibitor
1679	1688	Linker
1689	*1771*	*uracil glycosylase inhibitor*
**1772**	**1792**	**linker + SV40 BP NLS**

CBE6b – Cas9 (D10A) – SpCas9 (WT) – UGI
Plasmid ID: pHS0386

DNA sequence

GACATTGATTATTGACTAGTTATTAATAGTAATCAATTACGGGGTCATTAGTTCATAGCCCATATATGGAG
TTCCGCGTTACATAACTTACGGTAAATGGCCCGCCTGGCTGACCGCCCAACGACCCCCGCCCATTGA
CGTCAATAATGACGTATGTTCCCATAGTAACGCCAATAGGGACTTTCCATTGACGTCAATGGGTGGAGT
ATTTACGGTAAACTGCCCACTTGGCAGTACATCAAGTGTATCATATGCCAAGTACGCCCCCTATTGACG
TCAATGACGGTAAATGGCCCGCCTGGCATTATGCCCAGTACATGACCTTATGGGACTTTCCTACTTGG
CAGTACATCTACGTATTAGTCATCGCTATTACCATGGTGATGCGGTTTTGGCAGTACATCAATGGGCGT
GGATAGCGGTTTGACTCACGGGGATTTCCAAGTCTCCACCCCATTGACGTCAATGGGAGTTTGTTTTG
GCACCAAAATCAACGGGACTTTCCAAAATGTCGTAACAACTCCGCCCCATTGACGCAAATGGGCGGT
AGGCGTGTACGGTGGGAGGTCTATATAAGCAGAGCTGGTTTAGTGAACCGTCAGATCTCGAGCTCGG
TACCTAATACGACACACTATAAGGAAATAAGAGAGAAAAGAAGAGTAAGAAGAAATATAAGAGCCACCA
TGAAACGGACAGCCGACGGAAGCGAGTTCGAGTCACCAAAGAAGAAGCGGAAAGTCAGTTCTGAGG
TGGAGTTTTCCCACGAGTACTGGATGAGACATGCCCTGACCCTGGCCAAGAGGGCACGGGATGAGG
GAGAGGCGCCTGTGGGAGCCGTGCTGGTGCTGAACAATAGAGTGATCGGCGAGGGCTGGGTGAGA
CGTATCGGCCTGCACGACCCAACAGCCCATGCCGAAATTATGGCCCTGAGACAGGGCGGCCTGGTC
ATGCAGAACCCCAGACTGATTGACGCCACCCTGTACGTGACATTCGAGCCTTGCGTGATGTGCGCCG
GCGCCATGATCAACTCTAGGATCGGCCGCGTGGTGTTTGGCGTGAGGAACTCAAAAAGAGGCGCCG
CAGGCTCCCTGATGAACGTGCTGAACTACCCCGGCATGAATCACCGCGTCGAAATTACCGAGGGAAT
CCTGGCAGATGAATGTGCCGCCCTGCTGTGCGATTTCTATCGGATGCCTAGACAGGTGTTCAATGCTC
AGAAGAAGGCCCAGAGCTCCATCAACTCTGGCGGATCTAGCGGAGGATCCTCTGGCAGCGAGACAC
CAGGAACAAGCGAGTCAGCAACACCAGAGAGCAGTGGCGGCAGCAGCGGCGGCAGCGACAAGAA
GTACAGCATCGGCCTGGCCATCGGCACCAACTCTGTGGGCTGGGCCGTGATCACCGACGAGTACAA
GGTGCCCAGCAAGAAATTCAAGGTGCTGGGCAACACCGACCGGCACAGCATCAAGAAGAACCTGAT
CGGAGCCCTGCTGTTCGACAGCGGCGAAACAGCCGAGGCCACCCGGCTGAAGAGAACCGCCAGAA
GAAGATACACCAGACGGAAGAACCGGATCTGCTATCTGCAAGAGATCTTCAGCAACGAGATGGCCAA
GGTGGACGACAGCTTCTTCCACAGACTGGAAGAGTCCTTCCTGGTGGAAGAGGATAAGAAGCACGA
GCGGCACCCCATCTTCGGCAACATCGTGGACGAGGTGGCCTACCACGAGAAGTACCCCACCATCTA
CCACCTGAGAAAGAAACTGGTGGACAGCACCGACAAGGCCGACCTGCGGCTGATCTATCTGGCCCT
GGCCCACATGATCAAGTTCCGGGGCCACTTCCTGATCGAGGGCGACCTGAACCCCGACAACAGCGA
CGTGGACAAGCTGTTCATCCAGCTGGTGCAGACCTACAACCAGCTGTTCGAGGAAAACCCCATCAAC
GCCAGCGGCGTGGACGCCAAGGCCATCCTGTCTGCCAGACTGAGCAAGAGCAGACGGCTGGAAAA
TCTGATCGCCCAGCTGCCCGGCGAGAAGAAGAATGGCCTGTTCGGAAACCTGATTGCCCTGAGCCT
GGGCCTGACCCCCAACTTCAAGAGCAACTTCGACCTGGCCGAGGATGCCAAACTGCAGCTGAGCAA
GGACACCTACGACGACGACCTGGACAACCTGCTGGCCCAGATCGGCGACCAGTACGCCGACCTGTT
TCTGGCCGCCAAGAACCTGTCCGACGCCATCCTGCTGAGCGACATCCTGAGAGTGAACACCGAGAT
CACCAAGGCCCCCCTGAGCGCCTCTATGATCAAGAGATACGACGAGCACCACCAGGACCTGACCCT
GCTGAAAGCTCTCGTGCGGCAGCAGCTGCCTGAGAAGTACAAAGAGATTTTCTTCGACCAGAGCAAG
AACGGCTACGCCGGCTACATTGACGGCGGAGCCAGCCAGGAAGAGTTCTACAAGTTCATCAAGCCC
ATCCTGGAAAAGATGGACGGCACCGAGGAACTGCTCGTGAAGCTGAACAGAGAGGACCTGCTGCGG
AAGCAGCGGACCTTCGACAACGGCAGCATCCCCCACCAGATCCACCTGGGAGAGCTGCACGCCATT
CTGCGGCGGCAGGAAGATTTTTACCCATTCCTGAAGGACAACCGGGAAAAGATCGAGAAGATCCTGA
CCTTCCGCATCCCCTACTACGTGGGCCCTCTGGCCAGGGGAAACAGCAGATTCGCCTGGATGACCA
GAAAGAGCGAGGAAACCATCACCCCCTGGAACTTCGAGGAAGTGGTGGACAAGGGCGCTTCCGCC
CAGAGCTTCATCGAGCGGATGACCAACTTCGATAAGAACCTGCCCAACGAGAAGGTGCTGCCCAAG
CACAGCCTGCTGTACGAGTACTTCACCGTGTATAACGAGCTGACCAAAGTGAAATACGTGACCGAGG
GAATGAGAAAGCCCGCCTTCCTGAGCGGCGAGCAGAAAAAGGCCATCGTGGACCTGCTGTTCAAGA
CCAACCGGAAAGTGACCGTGAAGCAGCTGAAAGAGGACTACTTCAAGAAAATCGAGTGCTTCGACTC
CGTGGAAATCTCCGGCGTGGAAGATCGGTTCAACGCCTCCCTGGGCACATACCACGATCTGCTGAAA
ATTATCAAGGACAAGGACTTCCTGGACAATGAGGAAAACGAGGACATTCTGGAAGATATCGTGCTGAC
CCTGACACTGTTTGAGGACAGAGAGATGATCGAGGAACGGCTGAAAACCTATGCCCACCTGTTCGAC
GACAAAGTGATGAAGCAGCTGAAGCGGCGGAGATACACCGGCTGGGGCAGGCTGAGCCGGAAGCT
GATCAACGGCATCCGGGACAAGCAGTCCGGCAAGACAATCCTGGATTTCCTGAAGTCCGACGGCTT
CGCCAACAGAAACTTCATGCAGCTGATCCACGACGACAGCCTGACCTTTAAAGAGGACATCCAGAAA

GCCCAGGTGTCCGGCCAGGGCGATAGCCTGCACGAGCACATTGCCAATCTGGCCGGCAGCCCCGC
CATTAAGAAGGGCATCCTGCAGACAGTGAAGGTGGTGGACGAGCTCGTGAAAGTGATGGGCCGGCA
CAAGCCCGAGAACATCGTGATCGAAATGGCCAGAGAGAACCAGACCACCCAGAAGGGACAGAAGAA
CAGCCGCGAGAGAATGAAGCGGATCGAAGAGGGCATCAAAGAGCTGGGCAGCCAGATCCTGAAAGA
ACACCCCGTGGAAAACACCCAGCTGCAGAACGAGAAGCTGTACCTGTACTACCTGCAGAATGGGCG
GGATATGTACGTGGACCAGGAACTGGACATCAACCGGCTGTCCGACTACGATGTGGACCATATCGTG
CCTCAGAGCTTTCTGAAGGACGACTCCATCGACAACAAGGTGCTGACCAGAAGCGACAAGAACCGG
GGCAAGAGCGACAACGTGCCCTCCGAAGAGGTCGTGAAGAAGATGAAGAACTACTGGCGGCAGCT
GCTGAACGCCAAGCTGATTACCCAGAGAAAGTTCGACAATCTGACCAAGGCCGAGAGAGGCGGCCT
GAGCGAACTGGATAAGGCCGGCTTCATCAAGAGACAGCTGGTGGAAACCCGGCAGATCACAAAGCA
CGTGGCACAGATCCTGGACTCCCGGATGAACACTAAGTACGACGAGAATGACAAGCTGATCCGGGAA
GTGAAAGTGATCACCCTGAAGTCCAAGCTGGTGTCCGATTTCCGGAAGGATTTCCAGTTTTACAAAGT
GCGCGAGATCAACAACTACCACCACGCCCACGACGCCTACCTGAACGCCGTCGTGGGAACCGCCCT
GATCAAAAAGTACCCTAAGCTGGAAAGCGAGTTCGTGTACGGCGACTACAAGGTGTACGACGTGCGG
AAGATGATCGCCAAGAGCGAGCAGGAAATCGGCAAGGCTACCGCCAAGTACTTCTTCTACAGCAACA
TCATGAACTTTTTCAAGACCGAGATTACCCTGGCCAACGGCGAGATCCGGAAGCGGCCTCTGATCGA
GACAAACGGCGAAACCGGGGAGATCGTGTGGGATAAGGGCCGGGATTTTGCCACCGTGCGGAAAGT
GCTGAGCATGCCCCAAGTGAATATCGTGAAAAAGACCGAGGTGCAGACAGGCGGCTTCAGCAAAGA
GTCTATCCTGCCCAAGAGGAACAGCGATAAGCTGATCGCCAGAAAGAAGGACTGGGACCCTAAGAAG
TACGGCGGCTTCGACAGCCCCACCGTGGCCTATTCTGTGCTGGTGGTGGCCAAAGTGGAAAAGGGC
AAGTCCAAGAAACTGAAGAGTGTGAAAGAGCTGCTGGGGATCACCATCATGGAAAGAAGCAGCTTCG
AGAAGAATCCCATCGACTTTCTGGAAGCCAAGGGCTACAAAGAAGTGAAAAAGGACCTGATCATCAA
GCTGCCTAAGTACTCCCTGTTCGAGCTGGAAAACGGCCGGAAGAGAATGCTGGCCTCTGCCGGCGA
ACTGCAGAAGGGAAACGAACTGGCCCTGCCCTCCAAATATGTGAACTTCCTGTACCTGGCCAGCCAC
TATGAGAAGCTGAAGGGCTCCCCCGAGGATAATGAGCAGAAACAGCTGTTTGTGGAACAGCACAAGC
ACTACCTGGACGAGATCATCGAGCAGATCAGCGAGTTCTCCAAGAGAGTGATCCTGGCCGACGCTAA
TCTGGACAAAGTGCTGTCCGCCTACAACAAGCACCGGGATAAGCCCATCAGAGAGCAGGCCGAGAA
TATCATCCACCTGTTTACCCTGACCAATCTGGGAGCCCCTGCCGCCTTCAAGTACTTTGACACCACCA
TCGACCGGAAGAGGTACACCAGCACCAAAGAGGTGCTGGACGCCACCCTGATCCACCAGAGCATCA
CCGGCCTGTACGAGACACGGATCGACCTGTCTCAGCTGGGAGGTGACAGCGGCGGGAGCGGCGG
GAGCGGGGGGAGCACTAATCTGAGCGACATCATTGAGAAGGAGACTGGGAAACAGCTGGTCATTCA
GGAGTCCATCCTGATGCTGCCTGAGGAGGTGGAGGAAGTGATCGGCAACAAGCCAGAGTCTGACAT
CCTGGTGCACACCGCCTACGACGAGTCCACAGATGAGAATGTGATGCTGCTGACCTCTGACGCCCC
CGAGTATAAGCCTTGGGCCCTGGTCATCCAGGATTCTAACGGCGAGAATAAGATCAAGATGCTGAGC
GGAGGATCCGGAGGATCTGGAGGCAGC*ACCAACCTGTCTGACATCATCGAGAAGGAGACAGGCAAG*
CAGCTGGTCATCCAGGAGAGCATCCTGATGCTGCCCGAAGAAGTCGAAGAAGTGATCGGAAACAAG
CCTGAGAGCGATATCCTGGTCCATACCGCCTACGACGAGAGTACCGACGAAAATGTGATGCTGCTGA
CATCCGACGCCCCAGAGTATAAGCCCTGGGCTCTGGTCATCCAGGATTCCAACGGAGAGAACAAAAT
*CAAAATGCTG***TCTGGCGGCTCAAAAAGAACCGCCGACGGCAGCGAATTCGAGCCCAAGAAGAAGA**
GGAAAGTCTAATTAATTAAGCTGCCTTCTGCGGGGCTTGCCTTCTGGCCATGCCCTTCTTCTCTCCCT
TGCACCTGTACCTCTTGGTCTTTGAATAAAGCCTGAGTAGGAAGCGACTGTGCCTTCTAGTTGCCAGC
CATCTGTTGTTTGCCCCTCCCCCGTGCCTTCCTTGACCCTGGAAGGTGCCACTCCCACTGTCCTTTC
CTAATAAAATGAGAAAATTGCATCGCATTGTCTGAGTAGGTGTCATTCTATTCTGGGGGGTGGGGTGG
GGCAGGACAGCAAGGGGGAGGATTGGGAAGACAATAGCAGGCATGCTGGGGATGCGGTGGGCTCT
ATGG

Start	End	Feature Description
1	380	CMV enhancer
381	619	CMV promoter
620	636	T7 promoter with mismatch
637	683	5' UTR

684	740	BP NLS
741	1241	CBE6b deaminase
1242	1337	Linker
1338	5438	Cas9(D10A) SpCas9 wild-type
5439	5468	Linker
5469	5717	uracil glycosylase inhibitor
5718	5747	Linker
5748	*5996*	*uracil glycosylase inhibitor*
**5997**	**6062**	**linker + SV40 BP NLS**
6063	6163	3' UTR
6164	6391	bGH poly(A) signal

Amino acid sequence

MKRTADGSEFESPKKKRKVSSEVEFSHEYWMRHALTLAKRARDEGEAPVGAVLVLNNRVIGEGWVRRIG
LHDPTAHAEIMALRQGGLVMQNPRLIDATLYVTFEPCVMCAGAMINSRIGRVVFGVRNSKRGAAGSLMNV
LNYPGMNHRVEITEGILADECAALLCDFYRMPRQVFNAQKKAQSSINSGGSSGGSSGSETPGTSESATP
ESSGGSSGGSDKKYSIGLAIGTNSVGWAVITDEYKVPSKKFKVLGNTDRHSIKKNLIGALLFDSGETAEAT
RLKRTARRRYTRRKNRICYLQEIFSNEMAKVDDSFFHRLEESFLVEEDKKHERHPIFGNIVDEVAYHEKYP
TIYHLRKKLVDSTDKADLRLIYLALAHMIKFRGHFLIEGDLNPDNSDVDKLFIQLVQTYNQLFEENPINASGV
DAKAILSARLSKSRRLENLIAQLPGEKKNGLFGNLIALSLGLTPNFKSNFDLAEDAKLQLSKDTYDDDLDNL
LAQIGDQYADLFLAAKNLSDAILLSDILRVNTEITKAPLSASMIKRYDEHHQDLTLLKALVRQQLPEKYKEIFF
DQSKNGYAGYIDGGASQEEFYKFIKPILEKMDGTEELLVKLNREDLLRKQRTFDNGSIPHQIHLGELHAILR
RQEDFYPFLKDNREKIEKILTFRIPYYVGPLARGNSRFAWMTRKSEETITPWNFEEVVDKGASAQSFIERM
TNFDKNLPNEKVLPKHSLLYEYFTVYNELTKVKYVTEGMRKPAFLSGEQKKAIVDLLFKTNRKVTVKQLKE
DYFKKIECFDSVEISGVEDRFNASLGTYHDLLKIIKDKDFLDNEENEDILEDIVLTLTLFEDREMIEERLKTYA
HLFDDKVMKQLKRRRYTGWGRLSRKLINGIRDKQSGKTILDFLKSDGFANRNFMQLIHDDSLTFKEDIQKA
QVSGQGDSLHEHIANLAGSPAIKKGILQTVKVVDELVKVMGRHKPENIVIEMARENQTTQKGQKNSRERM
KRIEEGIKELGSQILKEHPVENTQLQNEKLYLYYLQNGRDMYVDQELDINRLSDYDVDHIVPQSFLKDDSID
NKVLTRSDKNRGKSDNVPSEEVVKKMKNYWRQLLNAKLITQRKFDNLTKAERGGLSELDKAGFIKRQLVE
TRQITKHVAQILDSRMNTKYDENDKLIREVKVITLKSKLVSDFRKDFQFYKVREINNYHHAHDAYLNAVVGT
ALIKKYPKLESEFVYGDYKVYDVRKMIAKSEQEIGKATAKYFFYSNIMNFFKTEITLANGEIRKRPLIETNGE
TGEIVWDKGRDFATVRKVLSMPQVNIVKKTEVQTGGFSKESILPKRNSDKLIARKKDWDPKKYGGFDSPT
VAYSVLVVAKVEKGKSKKLKSVKELLGITIMERSSFEKNPIDFLEAKGYKEVKKDLIIKLPKYSLFELENGRK
RMLASAGELQKGNELALPSKYVNFLYLASHYEKLKGSPEDNEQKQLFVEQHKHYLDEIIEQISEFSKRVILA
DANLDKVLSAYNKHRDKPIREQAENIIHLFTLTNLGAPAAFKYFDTTIDRKRYTSTKEVLDATLIHQSITGLYE
TRIDLSQLGGDSGGSGGSGGSTNLSDIIEKETGKQLVIQESILMLPEEVEEVIGNKPESDILVHTAYDESTDE
NVMLLTSDAPEYKPWALVIQDSNGENKIKMLSGGSGGSGGS*TNLSDIIEKETGKQLVIQESILMLPEEVEEV*
*IGNKPESDILVHTAYDESTDENVMLLTSDAPEYKPWALVIQDSNGENKIKML***SGGSKRTADGSEFEPKKK**
RKV

Start	End	Feature Description
1	19	BP NLS
20	186	TadCBEd deaminase
187	218	Linker

219	1585	Cas9(D10A) SpCas9 wild-type
1586	1595	Linker
1596	1678	uracil glycosylase inhibitor
1679	1688	Linker
1689	*1771*	*uracil glycosylase inhibitor*
**1772**	**1792**	**linker + SV40 BP NLS**

SpCas9 – VRQR variant – PEmax
Plasmid ID: pHS0234

DNA sequence

GACATTGATTATTGACTAGTTATTAATAGTAATCAATTACGGGGTCATTAGTTCATAGCCCATATATGGAG
TTCCGCGTTACATAACTTACGGTAAATGGCCCGCCTGGCTGACCGCCCAACGACCCCCGCCCATTGA
CGTCAATAATGACGTATGTTCCCATAGTAACGCCAATAGGGACTTTCCATTGACGTCAATGGGTGGAGT
ATTTACGGTAAACTGCCCACTTGGCAGTACATCAAGTGTATCATATGCCAAGTACGCCCCCTATTGACG
TCAATGACGGTAAATGGCCCGCCTGGCATTATGCCCAGTACATGACCTTATGGGACTTTCCTACTTGG
CAGTACATCTACGTATTAGTCATCGCTATTACCATGGTGATGCGGTTTTGGCAGTACATCAATGGGCGT
GGATAGCGGTTTGACTCACGGGGATTTCCAAGTCTCCACCCCATTGACGTCAATGGGAGTTTGTTTTG
GCACCAAAATCAACGGGACTTTCCAAAATGTCGTAACAACTCCGCCCCATTGACGCAAATGGGCGGT
AGGCGTGTACGGTGGGAGGTCTATATAAGCAGAGCTGGTTTAGTGAACCGTCAGATCCGCTAGAGAT
CCGCGGCCGCTAATACGACTCACTATAGGGAGAGCCGCCACCATGAAACGGACAGCCGACGGAAGC
GAGTTCGAGTCACCAAAGAAGAAGCGGAAAGTCGACAAGAAGTACAGCATCGGCCTGGACATCGGC
ACCAACTCTGTGGGCTGGGCCGTGATCACCGACGAGTACAAGGTGCCCAGCAAGAAATTCAAGGTG
CTGGGCAACACCGACCGGCACAGCATCAAGAAGAACCTGATCGGAGCCCTGCTGTTCGACAGCGGC
GAAACAGCCGAGGCCACCCGGCTGAAGAGAACCGCCAGAAGAAGATACACCAGACGGAAGAACCG
GATCTGCTATCTGCAAGAGATCTTCAGCAACGAGATGGCCAAGGTGGACGACAGCTTCTTCCACAGA
CTGGAAGAGTCCTTCCTGGTGGAAGAGGATAAGAAGCACGAGCGGCACCCCATCTTCGGCAACATC
GTGGACGAGGTGGCCTACCACGAGAAGTACCCCACCATCTACCACCTGAGAAAGAAACTGGTGGAC
AGCACCGACAAGGCCGACCTGCGGCTGATCTATCTGGCCCTGGCCCACATGATCAAGTTCCGGGGC
CACTTCCTGATCGAGGGCGACCTGAACCCCGACAACAGCGACGTGGACAAGCTGTTCATCCAGCTG
GTGCAGACCTACAACCAGCTGTTCGAGGAAAACCCCATCAACGCCAGCGGCGTGGACGCCAAGGCC
ATCCTGTCTGCCAGACTGAGCAAGAGCAGAAAGCTGGAAAATCTGATCGCCCAGCTGCCCGGCGAG
AAGAAGAATGGCCTGTTCGGAAACCTGATTGCCCTGAGCCTGGGCCTGACCCCCAACTTCAAGAGC
AACTTCGACCTGGCCGAGGATGCCAAACTGCAGCTGAGCAAGGACACCTACGACGACGACCTGGAC
AACCTGCTGGCCCAGATCGGCGACCAGTACGCCGACCTGTTTCTGGCCGCCAAGAACCTGTCCGAC
GCCATCCTGCTGAGCGACATCCTGAGAGTGAACACCGAGATCACCAAGGCCCCCCTGAGCGCCTCT
ATGATCAAGAGATACGACGAGCACCACCAGGACCTGACCCTGCTGAAAGCTCTCGTGCGGCAGCAG
CTGCCTGAGAAGTACAAAGAGATTTTCTTCGACCAGAGCAAGAACGGCTACGCCGGCTACATTGACG
GCGGAGCCAGCCAGGAAGAGTTCTACAAGTTCATCAAGCCCATCCTGGAAAAGATGGACGGCACCG
AGGAACTGCTCGTGAAGCTGAAGAGAGAGGACCTGCTGCGGAAGCAGCGGACCTTCGACAACGGC
AGCATCCCCCACCAGATCCACCTGGGAGAGCTGCACGCCATTCTGCGGCGGCAGGAAGATTTTTACC
CATTCCTGAAGGACAACCGGGAAAAGATCGAGAAGATCCTGACCTTCCGCATCCCCTACTACGTGGG
CCCTCTGGCCAGGGGAAACAGCAGATTCGCCTGGATGACCAGAAAGAGCGAGGAAACCATCACCCC
CTGGAACTTCGAGGAAGTGGTGGACAAGGGCGCTTCCGCCCAGAGCTTCATCGAGCGGATGACCAA
CTTCGATAAGAACCTGCCCAACGAGAAGGTGCTGCCCAAGCACAGCCTGCTGTACGAGTACTTCACC
GTGTATAACGAGCTGACCAAAGTGAAATACGTGACCGAGGGAATGAGAAAGCCCGCCTTCCTGAGCG
GCGAGCAGAAAAAGGCCATCGTGGACCTGCTGTTCAAGACCAACCGGAAAGTGACCGTGAAGCAGC
TGAAAGAGGACTACTTCAAGAAAATCGAGTGCTTCGACTCCGTGGAAATCTCCGGCGTGGAAGATCG
GTTCAACGCCTCCCTGGGCACATACCACGATCTGCTGAAAATTATCAAGGACAAGGACTTCCTGGACA
ATGAGGAAAACGAGGACATTCTGGAAGATATCGTGCTGACCCTGACACTGTTTGAGGACAGAGAGAT
GATCGAGGAACGGCTGAAAACCTATGCCCACCTGTTCGACGACAAAGTGATGAAGCAGCTGAAGCG
GCGGAGATACACCGGCTGGGGCAGGCTGAGCCGGAAGCTGATCAACGGCATCCGGGACAAGCAGT
CCGGCAAGACAATCCTGGATTTCCTGAAGTCCGACGGCTTCGCCAACAGAAACTTCATGCAGCTGAT
CCACGACGACAGCCTGACCTTTAAAGAGGACATCCAGAAAGCCCAGGTGTCCGGCCAGGGCGATAG
CCTGCACGAGCACATTGCCAATCTGGCCGGCAGCCCCGCCATTAAGAAGGGCATCCTGCAGACAGT
GAAGGTGGTGGACGAGCTCGTGAAAGTGATGGGCCGGCACAAGCCCGAGAACATCGTGATCGAAAT
GGCCAGAGAGAACCAGACCACCCAGAAGGGACAGAAGAACAGCCGCGAGAGAATGAAGCGGATCG
AAGAGGGCATCAAAGAGCTGGGCAGCCAGATCCTGAAAGAACACCCCGTGGAAAACACCCAGCTGC
AGAACGAGAAGCTGTACCTGTACTACCTGCAGAATGGGCGGGATATGTACGTGGACCAGGAACTGGA
CATCAACCGGCTGTCCGACTACGATGTGGACGCTATCGTGCCTCAGAGCTTTCTGAAGGACGACTCC
ATCGACAACAAGGTGCTGACCAGAAGCGACAAGAACCGGGGCAAGAGCGACAACGTGCCCTCCGA
AGAGGTCGTGAAGAAGATGAAGAACTACTGGCGGCAGCTGCTGAACGCCAAGCTGATTACCCAGAG
AAAGTTCGACAATCTGACCAAGGCCGAGAGAGGCGGCCTGAGCGAACTGGATAAGGCCGGCTTCAT

CAAGAGACAGCTGGTGGAAACCCGGCAGATCACAAAGCACGTGGCACAGATCCTGGACTCCCGGAT
GAACACTAAGTACGACGAGAATGACAAGCTGATCCGGGAAGTGAAAGTGATCACCCTGAAGTCCAAG
CTGGTGTCCGATTTCCGGAAGGATTTCCAGTTTTACAAAGTGCGCGAGATCAACAACTACCACCACGC
CCACGACGCCTACCTGAACGCCGTCGTGGGAACCGCCCTGATCAAAAAGTACCCTAAGCTGGAAAG
CGAGTTCGTGTACGGCGACTACAAGGTGTACGACGTGCGGAAGATGATCGCCAAGAGCGAGCAGGA
AATCGGCAAGGCTACCGCCAAGTACTTCTTCTACAGCAACATCATGAACTTTTTCAAGACCGAGATTAC
CCTGGCCAACGGCGAGATCCGGAAGCGGCCTCTGATCGAGACAAACGGCGAAACCGGGGAGATCG
TGTGGGATAAGGGCCGGGATTTTGCCACCGTGCGGAAAGTGCTGAGCATGCCCCAAGTGAATATCGT
GAAAAAGACCGAGGTGCAGACAGGCGGCTTCAGCAAAGAGTCTATCCTGCCCAAGAGGAACAGCGA
TAAGCTGATCGCCAGAAAGAAGGACTGGGACCCTAAGAAGTACGGCGGCTTCGTGAGCCCCACCGT
GGCCTATTCTGTGCTGGTGGTGGCCAAAGTGGAAAAGGGCAAGTCCAAGAAACTGAAGAGTGTGAA
AGAGCTGCTGGGGATCACCATCATGGAAAGAAGCAGCTTCGAGAAGAATCCCATCGACTTTCTGGAA
GCCAAGGGCTACAAAGAAGTGAAAAAGGACCTGATCATCAAGCTGCCTAAGTACTCCCTGTTCGAGC
TGGAAAACGGCCGGAAGAGAATGCTGGCCTCAGCCAGAGAACTGCAGAAGGGAAACGAACTGGCC
CTGCCCTCCAAATATGTGAACTTCCTGTACCTGGCCAGCCACTATGAGAAGCTGAAGGGCTCCCCCG
AGGATAATGAGCAGAAACAGCTGTTTGTGGAACAGCACAAGCACTACCTGGACGAGATCATCGAGCA
GATCAGCGAGTTCTCCAAGAGAGTGATCCTGGCCGACGCTAATCTGGACAAAGTGCTGTCCGCCTAC
AACAAGCACCGGGATAAGCCCATCAGAGAGCAGGCCGAGAATATCATCCACCTGTTTACCCTGACCA
ATCTGGGAGCCCCTGCCGCCTTCAAGTACTTTGACACCACCATCGACCGGAAGCAGTACAGAAGCAC
CAAAGAGGTGCTGGACGCCACCCTGATCCACCAGAGCATCACCGGCCTGTACGAGACACGGATCGA
CCTGTCTCAGCTGGGAGGTGACTCCGGCGGAAGCTCTGGTGGCAGCAAGCGGACCGCCGACGGCT
CTGAATTCGAGAGCCCTAAGAAGAAAAGAAAGGTGAGCGGAGGCTCTAGCGGCGGAAGCACCCTGA
ACATTGAAGACGAGTATAGACTGCATGAAACAAGCAAGGAACCCGACGTGTCCCTGGGCTCCACCTG
GCTGTCCGACTTTCCCCAGGCCTGGGCCGAGACAGGAGGAATGGGCCTGGCCGTGCGGCAGGCAC
CCCTGATCATCCCTCTGAAGGCCACCTCTACACCCGTGAGCATCAAGCAGTACCCTATGTCTCAGGA
GGCCAGACTGGGCATCAAGCCTCACATCCAGAGGCTGCTGGACCAGGGCATCCTGGTGCCATGCCA
GAGCCCCTGGAACACACCACTGCTGCCCGTGAAGAAGCCAGGCACCAATGACTATAGACCCGTGCA
GGATCTGAGAGAGGTGAACAAGAGGGTGGAGGATATCCACCCCACCGTGCCCAACCCTTACAATCTG
CTGTCCGGCCTGCCCCCTTCTCACCAGTGGTATACAGTGCTGGACCTGAAGGATGCCTTCTTTTGTC
TGAGACTGCACCCTACCAGCCAGCCACTGTTCGCCTTTGAGTGGAGGGACCCTGAGATGGGCATCT
CTGGCCAGCTGACCTGGACACGCCTGCCTCAGGGCTTCAAGAATAGCCCAACACTGTTTAACGAGG
CCCTGCACCGCGACCTGGCAGATTTCCGGATCCAGCACCCAGATCTGATCCTGCTGCAGTACGTGGA
CGATCTGCTGCTGGCCGCCACCAGCGAGCTGGATTGCCAGCAGGGAACACGCGCCCTGCTGCAGA
CCCTGGGAAACCTGGGATATAGGGCATCCGCCAAGAAGGCCCAGATCTGTCAGAAGCAGGTGAAGT
ACCTGGGCTATCTGCTGAAGGAGGGCCAGAGATGGCTGACAGAGGCCAGGAAGGAGACAGTGATG
GGCCAGCCAACACCCAAGACCCCAAGACAGCTGAGGGAGTTCCTGGGCAAAGCAGGATTTTGCAGG
CTGTTCATCCCAGGATTCGCAGAGATGGCAGCACCTCTGTACCCACTGACCAAGCCGGGCACCCTGT
TTAATTGGGGCCCTGACCAGCAGAAGGCCTATCAGGAGATCAAGCAGGCCCTGCTGACAGCACCAG
CCCTGGGCCTGCCAGACCTGACCAAGCCTTTCGAGCTGTTTGTGGATGAGAAGCAGGGCTACGCCA
AGGGCGTGCTGACCCAGAAGCTGGGACCATGGAGACGGCCCGTGGCCTATCTGTCCAAGAAGCTG
GACCCAGTGGCAGCAGGATGGCCACCATGCCTGAGGATGGTGGCAGCAATCGCCGTGCTGACAAAG
GATGCCGGCAAGCTGACCATGGGACAGCCACTGGTCATCCTGGCACCACACGCAGTGGAGGCCCT
GGTGAAGCAGCCTCCAGATCGCTGGCTGTCTAACGCCCGGATGACACACTACCAGGCCCTGCTGCT
GGACACCGATCGCGTGCAGTTTGGCCCTGTGGTGGCCCTGAATCCAGCCACCCTGCTGCCTCTGCC
AGAGGAGGGCCTGCAGCACAACTGTCTGGACATCCTGGCAGAGGCACACGGAACAAGGCCAGACC
TGACCGATCAGCCCCTGCCTGACGCCGATCACACATGGTATACCGATGGAAGCTCCCTGCTGCAGGA
GGGCCAGAGGAAGGCAGGAGCAGCAGTGACCACAGAGACAGAAGTGATCTGGGCCAAGGCCCTGC
CAGCAGGCACATCCGCCCAGCGGGCCGAGCTGATCGCCCTGACCCAGGCCCTGAAGATGGCCGAG
GGCAAGAAGCTGAACGTGTACACAGACTCCAGATATGCCTTCGCCACCGCACACATCCACGGAGAGA
TCTACAGGCGCCGGGGCTGGCTGACCTCTGAGGGCAAGGAGATCAAGAACAAGGATGAGATCCTGG
CCCTGCTGAAGGCCCTGTTTCTGCCCAAGCGGCTGAGCATCATCCACTGTCCTGGACACCAGAAGG
GACACTCCGCCGAGGCAAGGGGCAATCGGATGGCCGACCAGGCCGCCAGAAAGGCTGCTATTACTG
AAACTCCCGACACTTCCACTCTGCTGATTGAAAACTCCTCCCCTTCTGGCGGCTCAAAAAGAACCGC
CGACGGCAGCGAATTCGAGTCTCCCAAGAAGAAGAGGAAAGTCGGCTCTGGCCCTGCCGCTAAGAG
AGTGAAGCTGGACTAACCGGTCATCATCACCATCACCATTGAGTTTAAACCCGCTGATCAGCCTCGAC
TGTGCCTTCTAGTTGCCAGCCATCTGTTGTTTGCCCCTCCCCCGTGCCTTCCTTGACCCTGGAAGGT

GCCACTCCCACTGTCCTTTCCTAATAAAATGAGAAATTGCATCGCATTGTCTGAGTAGGTGTCATTCT
ATTCTGGGGGGTGGGGTGGGGCAGGACAGCAAGGGGGAGGATTGGGAAGACAATAGCAGGCATGC
TGGGGATGCGGTGGGCTCTATGG

Start	End	Feature Description
1	380	CMV enhancer
381	597	CMV promoter
598	657	5' UTR (contains T7 promoter)
658	714	SV40 BP NLS
715	4815	SpCas9 (R221K, N394K, H840A) - VRQR PAM variant
4816	4917	(SGGS)2 – SV40 NLS – SGGS2 optimized flexible linker
4918	6948	MMLV engineered reverse transcriptase
6949	7053	Linker and SV40 BP NLS
7054	7104	3' UTR
7105	7329	bGH poly(A) signal

Amino acid sequence
MKRTADGSEFESPKKKRKVDKKYSIGLDIGTNSVGWAVITDEYKVPSKKFKVLGNTDRHSIKKNLIGALLFD
SGETAEATRLKRTARRRYTRRKNRICYLQEIFSNEMAKVDDSFFHRLEESFLVEEDKKHERHPIFGNIVDE
VAYHEKYPTIYHLRKKLVDSTDKADLRLIYLALAHMIKFRGHFLIEGDLNPDNSDVDKLFIQLVQTYNQLFEE
NPINASGVDAKAILSARLSKSRRLENLIAQLPGEKKNGLFGNLIALSLGLTPNFKSNFDLAEDAKLQLSKDT
YDDDLDNLLAQIGDQYADLFLAAKNLSDAILLSDILRVNTEITKAPLSASMIKRYDEHHQDLTLLKALVRQQL
PEKYKEIFFDQSKNGYAGYIDGGASQEEFYKFIKPILEKMDGTEELLVKLKREDLLRKQRTFDNGSIPHQIH
LGELHAILRRQEDFYPFLKDNREKIEKILTFRIPYYVGPLARGNSRFAWMTRKSEETITPWNFEEVVDKGAS
AQSFIERMTNFDKNLPNEKVLPKHSLLYEYFTVYNELTKVKYVTEGMRKPAFLSGEQKKAIVDLLFKTNRK
VTVKQLKEDYFKKIECFDSVEISGVEDRFNASLGTYHDLLKIIKDKDFLDNEENEDILEDIVLTLTLFEDREMI
EERLKTYAHLFDDKVMKQLKRRRYTGWGRLSRKLINGIRDKQSGKTILDFLKSDGFANRNFMQLIHDDSLT
FKEDIQKAQVSGQGDSLHEHIANLAGSPAIKKGILQTVKVVDELVKVMGRHKPENIVIEMARENQTTQKGQ
KNSRERMKRIEEGIKELGSQILKEHPVENTQLQNEKLYLYYLQNGRDMYVDQELDINRLSDYDVDAIVPQS
FLKDDSIDNKVLTRSDKNRGKSDNVPSEEVVKKMKNYWRQLLNAKLITQRKFDNLTKAERGGLSELDKAG
FIKRQLVETRQITKHVAQILDSRMNTKYDENDKLIREVKVITLKSKLVSDFRKDFQFYKVREINNYHHAHDAY
LNAVVGTALIKKYPKLESEFVYGDYKVYDVRKMIAKSEQEIGKATAKYFFYSNIMNFFKTEITLANGEIRKRP
LIETNGETGEIVWDKGRDFATVRKVLSMPQVNIVKKTEVQTGGFSKESILPKRNSDKLIARKKDWDPKKYG
GFVSPTVAYSVLVVAKVEKGKSKKLKSVKELLGITIMERSSFEKNPIDFLEAKGYKEVKKDLIIKLPKYSLFEL
ENGRKRMLASARELQKGNELALPSKYVNFLYLASHYEKLKGSPEDNEQKQLFVEQHKHYLDEIIEQISEFS
KRVILADANLDKVLSAYNKHRDKPIREQAENIIHLFTLTNLGAPAAFKYFDTTIDRKQYRSTKEVLDATLIHQS
ITGLYETRIDLSQLGGDSGGSSGGSKRTADGSEFESPKKKRKVSGGSSGGSTLNIEDEYRLHETSKEPDV
SLGSTWLSDFPQAWAETGGMGLAVRQAPLIIPLKATSTPVSIKQYPMSQEARLGIKPHIQRLLDQGILVPC
QSPWNTPLLPVKKPGTNDYRPVQDLREVNKRVEDIHPTVPNPYNLLSGLPPSHQWYTVLDLKDAFFCLR
LHPTSQPLFAFEWRDPEMGISGQLTWTRLPQGFKNSPTLFNEALHRDLADFRIQHPDLILLQYVDDLLLAA
TSELDCQQGTRALLQTLGNLGYRASAKKAQICQKQVKYLGYLLKEGQRWLTEARKETVMGQPTPKTPRQ
LREFLGKAGFCRLFIPGFAEMAAPLYPLTKPGTLFNWGPDQQKAYQEIKQALLTAPALGLPDLTKPFELFVD
EKQGYAKGVLTQKLGPWRRPVAYLSKKLDPVAAGWPPCLRMVAAIAVLTKDAGKLTMGQPLVILAPHAVE
ALVKQPPDRWLSNARMTHYQALLLDTDRVQFGPVVALNPATLLPLPEEGLQHNCLDILAEAHGTRPDLTD
QPLPDADHTWYTDGSSLLQEGQRKAGAAVTTETEVIWAKALPAGTSAQRAELIALTQALKMAEGKKLNVY

TDSRYAFATAHIHGEIYRRRGWLTSEGKEIKNKDEILALLKALFLPKRLSIIHCPGHQKGHSAEARGNRMAD
QAARKAAITETPDTSTLLIENSSPSGGSKRTADGSEFESPKKKRKVGSGPAAKRVKLD

Start	End	Feature Description
1	19	SV40 BP NLS
20	1386	SpCas9 (R221K, N394K, H840A) - VRQR PAM variant
1387	1420	(SGGS)2 – SV40 NLS – SGGS2 optimized flexible linker
1421	2097	Engineered MMLV reverse transcriptase
2098	2131	Linker and SV40 BP NLS

SpCas9 – VRQR variant – PE6b
Plasmid ID: pHS0241

DNA sequence

GACATTGATTATTGACTAGTTATTAATAGTAATCAATTACGGGGTCATTAGTTCATAGCCCATATATGGAG
TTCCGCGTTACATAACTTACGGTAAATGGCCCGCCTGGCTGACCGCCCAACGACCCCCGCCCATTGA
CGTCAATAATGACGTATGTTCCCATAGTAACGCCAATAGGGACTTTCCATTGACGTCAATGGGTGGAGT
ATTTACGGTAAACTGCCCACTTGGCAGTACATCAAGTGTATCATATGCCAAGTACGCCCCCTATTGACG
TCAATGACGGTAAATGGCCCGCCTGGCATTATGCCCAGTACATGACCTTATGGGACTTTCCTACTTGG
CAGTACATCTACGTATTAGTCATCGCTATTACCATGGTGATGCGGTTTTGGCAGTACATCAATGGGCGT
GGATAGCGGTTTGACTCACGGGGATTTCCAAGTCTCCACCCCATTGACGTCAATGGGAGTTTGTTTTG
GCACCAAAATCAACGGGACTTTCCAAAATGTCGTAACAACTCCGCCCCATTGACGCAAATGGGCGGT
AGGCGTGTACGGTGGGAGGTCTATATAAGCAGAGCTGGTTTAGTGAACCGTCAGATCCGCTAGAGAT
CCGCGGCCGCTAATACGACTCACTATAGGGAGAGCCGCCACCATGAAACGGACAGCCGACGGAAGC
GAGTTCGAGTCACCAAAGAAGAAGCGGAAAGTCGACAAGAAGTACAGCATCGGCCTGGACATCGGC
ACCAACTCTGTGGGCTGGGCCGTGATCACCGACGAGTACAAGGTGCCCAGCAAGAAATTCAAGGTG
CTGGGCAACACCGACCGGCACAGCATCAAGAAGAACCTGATCGGAGCCCTGCTGTTCGACAGCGGC
GAAACAGCCGAGGCCACCCGGCTGAAGAGAACCGCCAGAAGAAGATACACCAGACGGAAGAACCG
GATCTGCTATCTGCAAGAGATCTTCAGCAACGAGATGGCCAAGGTGGACGACAGCTTCTTCCACAGA
CTGGAAGAGTCCTTCCTGGTGGAAGAGGATAAGAAGCACGAGCGGCACCCCATCTTCGGCAACATC
GTGGACGAGGTGGCCTACCACGAGAAGTACCCCACCATCTACCACCTGAGAAAGAAACTGGTGGAC
AGCACCGACAAGGCCGACCTGCGGCTGATCTATCTGGCCCTGGCCCACATGATCAAGTTCCGGGGC
CACTTCCTGATCGAGGGCGACCTGAACCCCGACAACAGCGACGTGGACAAGCTGTTCATCCAGCTG
GTGCAGACCTACAACCAGCTGTTCGAGGAAAACCCCATCAACGCCAGCGGCGTGGACGCCAAGGCC
ATCCTGTCTGCCAGACTGAGCAAGAGCAGAAAGCTGGAAAATCTGATCGCCCAGCTGCCCGGCGAG
AAGAAGAATGGCCTGTTCGGAAACCTGATTGCCCTGAGCCTGGGCCTGACCCCCAACTTCAAGAGC
AACTTCGACCTGGCCGAGGATGCCAAACTGCAGCTGAGCAAGGACACCTACGACGACGACCTGGAC
AACCTGCTGGCCCAGATCGGCGACCAGTACGCCGACCTGTTTCTGGCCGCCAAGAACCTGTCCGAC
GCCATCCTGCTGAGCGACATCCTGAGAGTGAACACCGAGATCACCAAGGCCCCCCTGAGCGCCTCT
ATGATCAAGAGATACGACGAGCACCACCAGGACCTGACCCTGCTGAAAGCTCTCGTGCGGCAGCAG
CTGCCTGAGAAGTACAAAGAGATTTTCTTCGACCAGAGCAAGAACGGCTACGCCGGCTACATTGACG
GCGGAGCCAGCCAGGAAGAGTTCTACAAGTTCATCAAGCCCATCCTGGAAAAGATGGACGGCACCG
AGGAACTGCTCGTGAAGCTGAAGAGAGAGGACCTGCTGCGGAAGCAGCGGACCTTCGACAACGGC
AGCATCCCCCACCAGATCCACCTGGGAGAGCTGCACGCCATTCTGCGGCGGCAGGAAGATTTTTACC
CATTCCTGAAGGACAACCGGGAAAAGATCGAGAAGATCCTGACCTTCCGCATCCCCTACTACGTGGG
CCCTCTGGCCAGGGGAAACAGCAGATTCGCCTGGATGACCAGAAAGAGCGAGGAAACCATCACCCC
CTGGAACTTCGAGGAAGTGGTGGACAAGGGCGCTTCCGCCCAGAGCTTCATCGAGCGGATGACCAA
CTTCGATAAGAACCTGCCCAACGAGAAGGTGCTGCCCAAGCACAGCCTGCTGTACGAGTACTTCACC
GTGTATAACGAGCTGACCAAAGTGAAATACGTGACCGAGGGAATGAGAAAGCCCGCCTTCCTGAGCG
GCGAGCAGAAAAAGGCCATCGTGGACCTGCTGTTCAAGACCAACCGGAAAGTGACCGTGAAGCAGC
TGAAAGAGGACTACTTCAAGAAAATCGAGTGCTTCGACTCCGTGGAAATCTCCGGCGTGGAAGATCG
GTTCAACGCCTCCCTGGGCACATACCACGATCTGCTGAAAATTATCAAGGACAAGGACTTCCTGGACA
ATGAGGAAAACGAGGACATTCTGGAAGATATCGTGCTGACCCTGACACTGTTTGAGGACAGAGAGAT
GATCGAGGAACGGCTGAAAACCTATGCCCACCTGTTCGACGACAAAGTGATGAAGCAGCTGAAGCG
GCGGAGATACACCGGCTGGGGCAGGCTGAGCCGGAAGCTGATCAACGGCATCCGGGACAAGCAGT
CCGGCAAGACAATCCTGGATTTCCTGAAGTCCGACGGCTTCGCCAACAGAAACTTCATGCAGCTGAT
CCACGACGACAGCCTGACCTTTAAAGAGGACATCCAGAAAGCCCAGGTGTCCGGCCAGGGCGATAG
CCTGCACGAGCACATTGCCAATCTGGCCGGCAGCCCCGCCATTAAGAAGGGCATCCTGCAGACAGT
GAAGGTGGTGGACGAGCTCGTGAAAGTGATGGGCCGGCACAAGCCCGAGAACATCGTGATCGAAAT
GGCCAGAGAGAACCAGACCACCCAGAAGGGACAGAAGAACAGCCGCGAGAGAATGAAGCGGATCG
AAGAGGGCATCAAAGAGCTGGGCAGCCAGATCCTGAAAGAACACCCCGTGGAAAACACCCAGCTGC
AGAACGAGAAGCTGTACCTGTACTACCTGCAGAATGGGCGGGATATGTACGTGGACCAGGAACTGGA
CATCAACCGGCTGTCCGACTACGATGTGGACGCTATCGTGCCTCAGAGCTTTCTGAAGGACGACTCC
ATCGACAACAAGGTGCTGACCAGAAGCGACAAGAACCGGGGCAAGAGCGACAACGTGCCCTCCGA
AGAGGTCGTGAAGAAGATGAAGAACTACTGGCGGCAGCTGCTGAACGCCAAGCTGATTACCCAGAG
AAAGTTCGACAATCTGACCAAGGCCGAGAGAGGCGGCCTGAGCGAACTGGATAAGGCCGGCTTCAT

CAAGAGACAGCTGGTGGAAACCCGGCAGATCACAAAGCACGTGGCACAGATCCTGGACTCCCGGAT
GAACACTAAGTACGACGAGAATGACAAGCTGATCCGGGAAGTGAAAGTGATCACCCTGAAGTCCAAG
CTGGTGTCCGATTTCCGGAAGGATTTCCAGTTTTACAAAGTGCGCGAGATCAACAACTACCACCACGC
CCACGACGCCTACCTGAACGCCGTCGTGGGAACCGCCCTGATCAAAAAGTACCCTAAGCTGGAAAG
CGAGTTCGTGTACGGCGACTACAAGGTGTACGACGTGCGGAAGATGATCGCCAAGAGCGAGCAGGA
AATCGGCAAGGCTACCGCCAAGTACTTCTTCTACAGCAACATCATGAACTTTTTCAAGACCGAGATTAC
CCTGGCCAACGGCGAGATCCGGAAGCGGCCTCTGATCGAGACAAACGGCGAAACCGGGGAGATCG
TGTGGGATAAGGGCCGGGATTTTGCCACCGTGCGGAAAGTGCTGAGCATGCCCCAAGTGAATATCGT
GAAAAAGACCGAGGTGCAGACAGGCGGCTTCAGCAAAGAGTCTATCCTGCCCAAGAGGAACAGCGA
TAAGCTGATCGCCAGAAAGAAGGACTGGGACCCTAAGAAGTACGGCGGCTTCGTGAGCCCCACCGT
GGCCTATTCTGTGCTGGTGGTGGCCAAAGTGGAAAAGGGCAAGTCCAAGAAACTGAAGAGTGTGAA
AGAGCTGCTGGGGATCACCATCATGGAAAGAAGCAGCTTCGAGAAGAATCCCATCGACTTTCTGGAA
GCCAAGGGCTACAAAGAAGTGAAAAAGGACCTGATCATCAAGCTGCCTAAGTACTCCCTGTTCGAGC
TGGAAAACGGCCGGAAGAGAATGCTGGCCTCAGCCAGAGAACTGCAGAAGGGAAACGAACTGGCC
CTGCCCTCCAAATATGTGAACTTCCTGTACCTGGCCAGCCACTATGAGAAGCTGAAGGGCTCCCCCG
AGGATAATGAGCAGAAACAGCTGTTTGTGGAACAGCACAAGCACTACCTGGACGAGATCATCGAGCA
GATCAGCGAGTTCTCCAAGAGAGTGATCCTGGCCGACGCTAATCTGGACAAAGTGCTGTCCGCCTAC
AACAAGCACCGGGATAAGCCCATCAGAGAGCAGGCCGAGAATATCATCCACCTGTTTACCCTGACCA
ATCTGGGAGCCCCTGCCGCCTTCAAGTACTTTGACACCACCATCGACCGGAAGCAGTACAGAAGCAC
CAAAGAGGTGCTGGACGCCACCCTGATCCACCAGAGCATCACCGGCCTGTACGAGACACGGATCGA
CCTGTCTCAGCTGGGAGGTGACTCCGGCGGAAGCTCTGGTGGCAGCAAGCGGACCGCCGACGGCT
CTGAATTCGAGAGCCCTAAGAAGAAAAGAAAGGTGAGCGGAGGCTCTAGCGGCGGAAGCATCAGCA
GCTCTAAGCACACCCTGAGCCAGATGAACAAGGTGAGCAACATCGTGAAGGAACCCGAGCTGCCCG
ACATCTACAAGGAATTTAAGGACATCACCGCCGACACCAATACCGAGAAGCTGCCTAAGCCTATTAAG
GGCCTGGAATTTGAAGTGGAACTGACACAGGAGAACTACAGACTGCCTATCCGGAACTATCCTCTGA
CTCCAGTCAAGATGCAGGCCATGAACGACGAGATCAATCAAGGCCTGAAAGGCGGCATCATCAGAGA
GAGCAAGGCCATCAACGCCTGCCCTGTTATATTCGTGCCCAGAAAGGAAGGCACACTGCGCATGGTG
GTCGACTACAGGCCCCTGAACAAGTACGTGAAGCCCAACGTCTACCCCCTGCCACTGATTGAACAAC
TGCTGGCCAAGATCCAGGGCAGCACCATTTTCACCAAGCTGGACCTGAAAAGCGCCTACCACCAGAT
CAGAGTGCGAAAGGGAGATGAGCACAAGCTGGCCTTCCGGTGCCCCAGAGGAGTCTTCGAGTACCT
GGTGATGCCTTACGGCATCAGCACAGCCCCTGCCCACTTTCAGTACTTCATCAACACAATCCTGGGC
GAGGCCAAGGAAAGCCACGTGGTGTGCTACATGGACGACATCCTGATCCATTCCAAGTCCGAGTCCG
AACACGTGAAACATGTGAAGGACGTGCTGCAAAAGCTGAAGAACGCTAATCTGATCATCAACCAGGC
CAAATGCGAGTTTCACCAGAGCCAAGTGAAGTTCATCGGCTACCACATCAGCGAGAAGGGCTTAACC
CCATGTCAGGAGAACATCGACAAGGTGCTGCAGTGGAAACAGCCTAAAAACCGGAAGGAACTGAGA
CAGTTCCTGGGCAGCGTGAACTACCTGAGAAAATTCATCCCCAAAACCAGCCAGTTGACCCACCCTC
TGAACAAACTCCTGAAAAAGGATGTCAGATGGAAATGGACCCCTACCCAGACCCAGGCTATCGAGAA
TATCAAGCAATGTCTGGTGTCTCCTCCTGTGCTGAGGCACTTCGACTTCAGCAAGAAGATCCTGCTTG
AGACAGACGTTTCTGATGTGGCCGTGGGAGCCGTGCTGAGCCAGAAGCATGATGATGATAAGTACTA
CCCTGTGGGCTATTACAGCGCTAAAATGAGCAAAGCCCAGCTGAATTATTCTGTGTCCGACAAGGAGA
TGCTGGCTATCATCAAAAGCCTGGAGCACTGGCGGCACTACCTGGAATCTACAATCGAGCCCTTCAA
GATCCTGACCGACCACAGAAACCTGATTGGAAGAATCACAAACGAGAGCGAACCAGAGAACAAGCG
GCTGGCCAGATGGCAGCTGTTCCTGCAGGACTTCAACTTCGAGATCAACTACAGACCTGGCTCTGCA
AATCACATCGCCGATGCCCTGTCTAGAATCGTGGACGAGACTGAGCCTATCCCTAAGGACAACGAAG
ATAACAGCATCAACTTCGTGAACCAGATCAGCATCTCTGGCGGCTCAAAAAGAACCGCCGACGGCAG
CGAATTCGAGTCTCCCAAGAAGAAGAGGAAAGTCGGCTCTGGCCCTGCCGCTAAGAGAGTGAAGCT
GGACTAACCGGTCATCATCACCATCACCATTGAGTTTAAACCCGCTGATCAGCCTCGACTGTGCCTTC
TAGTTGCCAGCCATCTGTTGTTTGCCCCTCCCCCGTGCCTTCCTTGACCCTGGAAGGTGCCACTCCC
ACTGTCCTTTCCTAATAAAATGAGAAAATTGCATCGCATTGTCTGAGTAGGTGTCATTCTATTCTGGGG
GGTGGGGTGGGGCAGGACAGCAAGGGGGAGGATTGGGAAGACAATAGCAGGCATGCTGGGGATGC
GGTGGGCTCTATGG

Start	End	Feature Description
1	380	CMV enhancer

381	597	CMV promoter
598	657	5’ UTR (contains T7 promoter)
658	714	SV40 BP NLS
715	4815	SpCas9 (R221K, N394K, H840A) - VRQR PAM variant
4816	4917	(SGGS)2 – SV40 NLS – SGGS2 optimized flexible linker
4918	6432	PE6b evolved reverse transcriptase from Tf1
6433	6537	Linker and SV40 BP NLS
6538	6588	3’ UTR
6589	6813	bGH poly(A) signal

<u>Amino acid sequence</u>
MKRTADGSEFESPKKKRKVDKKYSIGLDIGTNSVGWAVITDEYKVPSKKFKVLGNTDRHSIKKNLIGALLFD
SGETAEATRLKRTARRRYTRRKNRICYLQEIFSNEMAKVDDSFFHRLEESFLVEEDKKHERHPIFGNIVDE
VAYHEKYPTIYHLRKKLVDSTDKADLRLIYLALAHMIKFRGHFLIEGDLNPDNSDVDKLFIQLVQTYNQLFEE
NPINASGVDAKAILSARLSKSRKLENLIAQLPGEKKNGLFGNLIALSLGLTPNFKSNFDLAEDAKLQLSKDT
YDDDLDNLLAQIGDQYADLFLAAKNLSDAILLSDILRVNTEITKAPLSASMIKRYDEHHQDLTLLKALVRQQL
PEKYKEIFFDQSKNGYAGYIDGGASQEEFYKFIKPILEKMDGTEELLVKLKREDLLRKQRTFDNGSIPHQIH
LGELHAILRRQEDFYPFLKDNREKIEKILTFRIPYYVGPLARGNSRFAWMTRKSEETITPWNFEEVVDKGAS
AQSFIERMTNFDKNLPNEKVLPKHSLLYEYFTVYNELTKVKYVTEGMRKPAFLSGEQKKAIVDLLFKTNRK
VTVKQLKEDYFKKIECFDSVEISGVEDRFNASLGTYHDLLKIIKDKDFLDNEENEDILEDIVLTLTLFEDREMI
EERLKTYAHLFDDKVMKQLKRRRYTGWGRLSRKLINGIRDKQSGKTILDFLKSDGFANRNFMQLIHDDSLT
FKEDIQKAQVSGQGDSLHEHIANLAGSPAIKKGILQTVKVVDELVKVMGRHKPENIVIEMARENQTTQKGQ
KNSRERMKRIEEGIKELGSQILKEHPVENTQLQNEKLYLYYLQNGRDMYVDQELDINRLSDYDVDAIVPQS
FLKDDSIDNKVLTRSDKNRGKSDNVPSEEVVKKMKNYWRQLLNAKLITQRKFDNLTKAERGGLSELDKAG
FIKRQLVETRQITKHVAQILDSRMNTKYDENDKLIREVKVITLKSKLVSDFRKDFQFYKVREINNYHHAHDAY
LNAVVGTALIKKYPKLESEFVYGDYKVYDVRKMIAKSEQEIGKATAKYFFYSNIMNFFKTEITLANGEIRKRP
LIETNGETGEIVWDKGRDFATVRKVLSMPQVNIVKKTEVQTGGFSKESILPKRNSDKLIARKKDWDPKKYG
GFVSPTVAYSVLVVAKVEKGKSKKLKSVKELLGITIMERSSFEKNPIDFLEAKGYKEVKKDLIIKLPKYSLFEL
ENGRKRMLASARELQKGNELALPSKYVNFLYLASHYEKLKGSPEDNEQKQLFVEQHKHYLDEIIEQISEFS
KRVILADANLDKVLSAYNKHRDKPIREQAENIIHLFTLTNLGAPAAFKYFDTTIDRKQYRSTKEVLDATLIHQS
ITGLYETRIDLSQLGGDSGGSSGGSKRTADGSEFESPKKKRKVSGGSSGGSISSSKHTLSQMNKVSNIVK
EPELPDIYKEFKDITADTNTEKLPKPIKGLEFEVELTQENYRLPIRNYPLTPVKMQAMNDEINQGLKGGIIRE
SKAINACPVIFVPRKEGTLRMVVDYRPLNKYVKPNVYPLPLIEQLLAKIQGSTIFTKLDLKSAYHQIRVRKGD
EHKLAFRCPRGVFEYLVMPYGISTAPAHFQYFINTILGEAKESHVVCYMDDILIHSKSESEHVKHVKDVLQK
LKNANLIINQAKCEFHQSQVKFIGYHISEKGLTPCQENIDKVLQWKQPKNRKELRQFLGSVNYLRKFIPKTS
QLTHPLNKLLKKDVRWKWTPTQTQAIENIKQCLVSPPVLRHFDFSKKILLETDVSDVAVGAVLSQKHDDDK
YYPVGYYSAKMSKAQLNYSVSDKEMLAIIKSLEHWRHYLESTIEPFKILTDHRNLIGRITNESEPENKRLAR
WQLFLQDFNFEINYRPGSANHIADALSRIVDETEPIPKDNEDNSINFVNQISISGGSKRTADGSEFESPKKK
RKVGSGPAAKRVKLD

Start	End	Feature Description
1	19	SV40 BP NLS
20	1386	SpCas9 (R221K, N394K, H840A) - VRQR PAM variant
1387	1420	(SGGS)2 – SV40 NLS – SGGS2 optimized flexible linker

1421	1925	PE6b evolved reverse transcriptase from Tf1
1926	1959	Linker and SV40 BP NLS

SpCas9 – VRQR variant – PE6c
Plasmid ID: pHS0242

DNA sequence

GACATTGATTATTGACTAGTTATTAATAGTAATCAATTACGGGGTCATTAGTTCATAGCCCATATATGGAG
TTCCGCGTTACATAACTTACGGTAAATGGCCCGCCTGGCTGACCGCCCAACGACCCCCGCCCATTGA
CGTCAATAATGACGTATGTTCCCATAGTAACGCCAATAGGGACTTTCCATTGACGTCAATGGGTGGAGT
ATTTACGGTAAACTGCCCACTTGGCAGTACATCAAGTGTATCATATGCCAAGTACGCCCCCTATTGACG
TCAATGACGGTAAATGGCCCGCCTGGCATTATGCCCAGTACATGACCTTATGGGACTTTCCTACTTGG
CAGTACATCTACGTATTAGTCATCGCTATTACCATGGTGATGCGGTTTTGGCAGTACATCAATGGGCGT
GGATAGCGGTTTGACTCACGGGGATTTCCAAGTCTCCACCCCATTGACGTCAATGGGAGTTTGTTTTG
GCACCAAAATCAACGGGACTTTCCAAAATGTCGTAACAACTCCGCCCCATTGACGCAAATGGGCGGT
AGGCGTGTACGGTGGGAGGTCTATATAAGCAGAGCTGGTTTAGTGAACCGTCAGATCCGCTAGAGAT
CCGCGGCCGCTAATACGACTCACTATAGGGAGAGCCGCCACCATGAAACGGACAGCCGACGGAAGC
GAGTTCGAGTCACCAAAGAAGAAGCGGAAAGTCGACAAGAAGTACAGCATCGGCCTGGACATCGGC
ACCAACTCTGTGGGCTGGGCCGTGATCACCGACGAGTACAAGGTGCCCAGCAAGAAATTCAAGGTG
CTGGGCAACACCGACCGGCACAGCATCAAGAAGAACCTGATCGGAGCCCTGCTGTTCGACAGCGGC
GAAACAGCCGAGGCCACCCGGCTGAAGAGAACCGCCAGAAGAAGATACACCAGACGGAAGAACCG
GATCTGCTATCTGCAAGAGATCTTCAGCAACGAGATGGCCAAGGTGGACGACAGCTTCTTCCACAGA
CTGGAAGAGTCCTTCCTGGTGGAAGAGGATAAGAAGCACGAGCGGCACCCCATCTTCGGCAACATC
GTGGACGAGGTGGCCTACCACGAGAAGTACCCCACCATCTACCACCTGAGAAAGAAACTGGTGGAC
AGCACCGACAAGGCCGACCTGCGGCTGATCTATCTGGCCCTGGCCCACATGATCAAGTTCCGGGGC
CACTTCCTGATCGAGGGCGACCTGAACCCCGACAACAGCGACGTGGACAAGCTGTTCATCCAGCTG
GTGCAGACCTACAACCAGCTGTTCGAGGAAAACCCCATCAACGCCAGCGGCGTGGACGCCAAGGCC
ATCCTGTCTGCCAGACTGAGCAAGAGCAGAAAGCTGGAAAATCTGATCGCCCAGCTGCCCGGCGAG
AAGAAGAATGGCCTGTTCGGAAACCTGATTGCCCTGAGCCTGGGCCTGACCCCCAACTTCAAGAGC
AACTTCGACCTGGCCGAGGATGCCAAACTGCAGCTGAGCAAGGACACCTACGACGACGACCTGGAC
AACCTGCTGGCCCAGATCGGCGACCAGTACGCCGACCTGTTTCTGGCCGCCAAGAACCTGTCCGAC
GCCATCCTGCTGAGCGACATCCTGAGAGTGAACACCGAGATCACCAAGGCCCCCCTGAGCGCCTCT
ATGATCAAGAGATACGACGAGCACCACCAGGACCTGACCCTGCTGAAAGCTCTCGTGCGGCAGCAG
CTGCCTGAGAAGTACAAAGAGATTTTCTTCGACCAGAGCAAGAACGGCTACGCCGGCTACATTGACG
GCGGAGCCAGCCAGGAAGAGTTCTACAAGTTCATCAAGCCCATCCTGGAAAAGATGGACGGCACCG
AGGAACTGCTCGTGAAGCTGAAGAGAGAGGACCTGCTGCGGAAGCAGCGGACCTTCGACAACGGC
AGCATCCCCCACCAGATCCACCTGGGAGAGCTGCACGCCATTCTGCGGCGGCAGGAAGATTTTTACC
CATTCCTGAAGGACAACCGGGAAAAGATCGAGAAGATCCTGACCTTCCGCATCCCCTACTACGTGGG
CCCTCTGGCCAGGGGAAACAGCAGATTCGCCTGGATGACCAGAAAGAGCGAGGAAACCATCACCCC
CTGGAACTTCGAGGAAGTGGTGGACAAGGGCGCTTCCGCCCAGAGCTTCATCGAGCGGATGACCAA
CTTCGATAAGAACCTGCCCAACGAGAAGGTGCTGCCCAAGCACAGCCTGCTGTACGAGTACTTCACC
GTGTATAACGAGCTGACCAAAGTGAAATACGTGACCGAGGGAATGAGAAAGCCCGCCTTCCTGAGCG
GCGAGCAGAAAAAGGCCATCGTGGACCTGCTGTTCAAGACCAACCGGAAAGTGACCGTGAAGCAGC
TGAAAGAGGACTACTTCAAGAAAATCGAGTGCTTCGACTCCGTGGAAATCTCCGGCGTGGAAGATCG
GTTCAACGCCTCCCTGGGCACATACCACGATCTGCTGAAAATTATCAAGGACAAGGACTTCCTGGACA
ATGAGGAAAACGAGGACATTCTGGAAGATATCGTGCTGACCCTGACACTGTTTGAGGACAGAGAGAT
GATCGAGGAACGGCTGAAAACCTATGCCCACCTGTTCGACGACAAAGTGATGAAGCAGCTGAAGCG
GCGGAGATACACCGGCTGGGGCAGGCTGAGCCGGAAGCTGATCAACGGCATCCGGGACAAGCAGT
CCGGCAAGACAATCCTGGATTTCCTGAAGTCCGACGGCTTCGCCAACAGAAACTTCATGCAGCTGAT
CCACGACGACAGCCTGACCTTTAAAGAGGACATCCAGAAAGCCCAGGTGTCCGGCCAGGGCGATAG
CCTGCACGAGCACATTGCCAATCTGGCCGGCAGCCCCGCCATTAAGAAGGGCATCCTGCAGACAGT
GAAGGTGGTGGACGAGCTCGTGAAAGTGATGGGCCGGCACAAGCCCGAGAACATCGTGATCGAAAT
GGCCAGAGAGAACCAGACCACCCAGAAGGGACAGAAGAACAGCCGCGAGAGAATGAAGCGGATCG
AAGAGGGCATCAAAGAGCTGGGCAGCCAGATCCTGAAAGAACACCCCGTGGAAAACACCCAGCTGC
AGAACGAGAAGCTGTACCTGTACTACCTGCAGAATGGGCGGGATATGTACGTGGACCAGGAACTGGA
CATCAACCGGCTGTCCGACTACGATGTGGACGCTATCGTGCCTCAGAGCTTTCTGAAGGACGACTCC
ATCGACAACAAGGTGCTGACCAGAAGCGACAAGAACCGGGGCAAGAGCGACAACGTGCCCTCCGA
AGAGGTCGTGAAGAAGATGAAGAACTACTGGCGGCAGCTGCTGAACGCCAAGCTGATTACCCAGAG
AAAGTTCGACAATCTGACCAAGGCCGAGAGAGGCGGCCTGAGCGAACTGGATAAGGCCGGCTTCAT

CAAGAGACAGCTGGTGGAAACCCGGCAGATCACAAAGCACGTGGCACAGATCCTGGACTCCCGGAT
GAACACTAAGTACGACGAGAATGACAAGCTGATCCGGGAAGTGAAAGTGATCACCCTGAAGTCCAAG
CTGGTGTCCGATTTCCGGAAGGATTTCCAGTTTTACAAAGTGCGCGAGATCAACAACTACCACCACGC
CCACGACGCCTACCTGAACGCCGTCGTGGGAACCGCCCTGATCAAAAAGTACCCTAAGCTGGAAAG
CGAGTTCGTGTACGGCGACTACAAGGTGTACGACGTGCGGAAGATGATCGCCAAGAGCGAGCAGGA
AATCGGCAAGGCTACCGCCAAGTACTTCTTCTACAGCAACATCATGAACTTTTTCAAGACCGAGATTAC
CCTGGCCAACGGCGAGATCCGGAAGCGGCCTCTGATCGAGACAAACGGCGAAACCGGGGAGATCG
TGTGGGATAAGGGCCGGGATTTTGCCACCGTGCGGAAAGTGCTGAGCATGCCCCAAGTGAATATCGT
GAAAAAGACCGAGGTGCAGACAGGCGGCTTCAGCAAAGAGTCTATCCTGCCCAAGAGGAACAGCGA
TAAGCTGATCGCCAGAAAGAAGGACTGGGACCCTAAGAAGTACGGCGGCTTCGTGAGCCCCACCGT
GGCCTATTCTGTGCTGGTGGTGGCCAAAGTGGAAAAGGGCAAGTCCAAGAAACTGAAGAGTGTGAA
AGAGCTGCTGGGGATCACCATCATGGAAAGAAGCAGCTTCGAGAAGAATCCCATCGACTTTCTGGAA
GCCAAGGGCTACAAAGAAGTGAAAAAGGACCTGATCATCAAGCTGCCTAAGTACTCCCTGTTCGAGC
TGGAAAACGGCCGGAAGAGAATGCTGGCCTCAGCCAGAGAACTGCAGAAGGGAAACGAACTGGCC
CTGCCCTCCAAATATGTGAACTTCCTGTACCTGGCCAGCCACTATGAGAAGCTGAAGGGCTCCCCCG
AGGATAATGAGCAGAAACAGCTGTTTGTGGAACAGCACAAGCACTACCTGGACGAGATCATCGAGCA
GATCAGCGAGTTCTCCAAGAGAGTGATCCTGGCCGACGCTAATCTGGACAAAGTGCTGTCCGCCTAC
AACAAGCACCGGGATAAGCCCATCAGAGAGCAGGCCGAGAATATCATCCACCTGTTTACCCTGACCA
ATCTGGGAGCCCCTGCCGCCTTCAAGTACTTTGACACCACCATCGACCGGAAGCAGTACAGAAGCAC
CAAAGAGGTGCTGGACGCCACCCTGATCCACCAGAGCATCACCGGCCTGTACGAGACACGGATCGA
CCTGTCTCAGCTGGGAGGTGACTCCGGCGGAAGCTCTGGTGGCAGCAAGCGGACCGCCGACGGCT
CTGAATTCGAGAGCCCTAAGAAGAAAAGAAAGGTGAGCGGAGGCTCTAGCGGCGGAAGCATCAGCA
GCTCTAAGCACACCCTGAGCCAGATGAACAAGGTGAGCAACATCGTGAAGGAACCCGAGCTGCCCG
ACATCTACAAGGAATTTAAGGACATCACCGCCGACACCAATACCGAGAAGCTGCCTAAGCCTATTAAG
GGCCTGGAATTTGAAGTGGAACTGACACAGGAGAACTACAGACTGCCTATCCGGAACTATCCTCTGA
CTCCAGTCAAGATGCAGGCCATGAACGACGAGATCAATCAAGGCCTGAAAGGCGGCATCATCAGAGA
GAGCAAGGCCATCAACGCCTGCCCTGTTATATTCGTGCCCAGAAAGGAAGGCACACTGCGCATGGTG
GTCGACTACAGGCCCCTGAACAAGTACGTGAAGCCCAACGTCTACCCCCTGCCACTGATTGAACAAC
TGCTGGCCAAGATCCAGGGCAGCACCATTTTCACCAAGCTGGACCTGAAAAGCGCCTACCACCAGAT
CAGAGTGCGAAAGGGAGATGAGCACAAGCTGGCCTTCCGGTGCCCCAGAGGAGTCTTCGAGTACCT
GGTGATGCCTTACGGCATCAAGACAGCCCCTGCCCACTTTCAGTACTTCATCAACACAATCCTGGGC
GAGGCCAAGGAAAGCCACGTGGTGTGCTACATGGACGACATCCTGATCCATTCCAAGTCCGAGTCCG
AACACGTGAAACATGTGAAGGACGTGCTGCAAAAGCTGAAGAACGCTAATCTGATCATCAACCAGGC
CAAATGCGAGTTTCACCAGAGCCAAGTGAAGTTCCTGGGCTACCACATCAGCGAGAAGGGCTTAACC
CCATGTCAGGAGAACATCGACAAGGTGCTGCAGTGGAAACAGCCTAAAAACCAGAAGGAACTGAGA
CAGTTCCTGGGCCAGGTGAACTACCTGAGAAAATTCATCCCCAAAACCAGCCAGTTGACCCACCCTC
TGAACAAACTCCTGAAAAAGGATGTCAGATGGAAATGGACCCCTACCCAGACCCAGGCTATCGAGAA
TATCAAGCAATGTCTGGTGTCTCCTCCTGTGCTGAGGCACTTCGACTTCAGCAAGAAGATCCTGCTTG
AGACAGACGTTTCTGATGTGGCCGTGGGAGCCGTGCTGAGCCAGAAGCATGATGATGATAAGTACTA
CCCTGTGGGCTATTACAGCGCTAAAATGAGCAAAGCCCAGCTGAATTATTCTGTGTCCGACAAGGAGA
TGCTGGCTATCATCAAAAGCCTGGAGCACTGGCGGCACTACCTGGAATCTACAATCGAGCCCTTCAA
GATCCTGACCGACCACAGAAACCTGATTGGAAGAATCACAAACGAGAGCGAACCAGAGAACAAGCG
GCTGGCCAGATGGCAGCTGTTCCTGCAGGACTTCAACTTCGAGATCAACTACAGACCTGGCTCTGCA
AATCACATCGCCGATGCCCTGTCTAGAATCGTGGACGAGACTGAGCCTATCCCTAAGGACAACGAAG
ATAACAGCATCAACTTCGTGAACCAGATCAGCATCTCTGGCGGCTCAAAAAGAACCGCCGACGGCAG
CGAATTCGAGTCTCCCAAGAAGAAGAGGAAAGTCGGCTCTGGCCCTGCCGCTAAGAGAGTGAAGCT
GGACTAACCGGTCATCATCACCATCACCATTGAGTTTAAACCCGCTGATCAGCCTCGACTGTGCCTTC
TAGTTGCCAGCCATCTGTTGTTTGCCCCTCCCCCGTGCCTTCCTTGACCCTGGAAGGTGCCACTCCC
ACTGTCCTTTCCTAATAAAATGAGAAAATTGCATCGCATTGTCTGAGTAGGTGTCATTCTATTCTGGGG
GGTGGGGTGGGGCAGGACAGCAAGGGGGAGGATTGGGAAGACAATAGCAGGCATGCTGGGGATGC
GGTGGGCTCTATGG

Start	End	Feature Description
1	380	CMV enhancer

381	597	CMV promoter
598	657	5’ UTR (contains T7 promoter)
658	714	SV40 BP NLS
715	4815	SpCas9 (R221K, N394K, H840A) - VRQR PAM variant
4816	4917	(SGGS)2 – SV40 NLS – SGGS2 optimized flexible linker
4918	6432	PE6c engineered & evolved reverse transcriptase from Tf1
6433	6537	Linker and SV40 BP NLS
6538	6588	3’ UTR
6589	6813	bGH poly(A) signal

Amino acid sequence

MKRTADGSEFESPKKKRKVDKKYSIGLDIGTNSVGWAVITDEYKVPSKKFKVLGNTDRHSIKKNLIGALLFD
SGETAEATRLKRTARRRYTRRKNRICYLQEIFSNEMAKVDDSFFHRLEESFLVEEDKKHERHPIFGNIVDE
VAYHEKYPTIYHLRKKLVDSTDKADLRLIYLALAHMIKFRGHFLIEGDLNPDNSDVDKLFIQLVQTYNQLFEE
NPINASGVDAKAILSARLSKSRKLENLIAQLPGEKKNGLFGNLIALSLGLTPNFKSNFDLAEDAKLQLSKDT
YDDDLDNLLAQIGDQYADLFLAAKNLSDAILLSDILRVNTEITKAPLSASMIKRYDEHHQDLTLLKALVRQQL
PEKYKEIFFDQSKNGYAGYIDGGASQEEFYKFIKPILEKMDGTEELLVKLKREDLLRKQRTFDNGSIPHQIH
LGELHAILRRQEDFYPFLKDNREKIEKILTFRIPYYVGPLARGNSRFAWMTRKSEETITPWNFEEVVDKGAS
AQSFIERMTNFDKNLPNEKVLPKHSLLYEYFTVYNELTKVKYVTEGMRKPAFLSGEQKKAIVDLLFKTNRK
VTVKQLKEDYFKKIECFDSVEISGVEDRFNASLGTYHDLLKIIKDKDFLDNEENEDILEDIVLTLTLFEDREMI
EERLKTYAHLFDDKVMKQLKRRRYTGWGRLSRKLINGIRDKQSGKTILDFLKSDGFANRNFMQLIHDDSLT
FKEDIQKAQVSGQGDSLHEHIANLAGSPAIKKGILQTVKVVDELVKVMGRHKPENIVIEMARENQTTQKGQ
KNSRERMKRIEEGIKELGSQILKEHPVENTQLQNEKLYLYYLQNGRDMYVDQELDINRLSDYDVDAIVPQS
FLKDDSIDNKVLTRSDKNRGKSDNVPSEEVVKKMKNYWRQLLNAKLITQRKFDNLTKAERGGLSELDKAG
FIKRQLVETRQITKHVAQILDSRMNTKYDENDKLIREVKVITLKSKLVSDFRKDFQFYKVREINNYHHAHDAY
LNAVVGTALIKKYPKLESEFVYGDYKVYDVRKMIAKSEQEIGKATAKYFFYSNIMNFFKTEITLANGEIRKRP
LIETNGETGEIVWDKGRDFATVRKVLSMPQVNIVKKTEVQTGGFSKESILPKRNSDKLIARKKDWDPKKYG
GFVSPTVAYSVLVVAKVEKGKSKKLKSVKELLGITIMERSSFEKNPIDFLEAKGYKEVKKDLIIKLPKYSLFEL
ENGRKRMLASARELQKGNELALPSKYVNFLYLASHYEKLKGSPEDNEQKQLFVEQHKHYLDEIIEQISEFS
KRVILADANLDKVLSAYNKHRDKPIREQAENIIHLFTLTNLGAPAAFKYFDTTIDRKQYRSTKEVLDATLIHQS
ITGLYETRIDLSQLGGDSGGSSGGSKRTADGSEFESPKKKRKVSGGSSGGSISSSKHTLSQMNKVSNIVK
EPELPDIYKEFKDITADTNTEKLPKPIKGLEFEVELTQENYRLPIRNYPLTPVKMQAMNDEINQGLKGGIIRE
SKAINACPVIFVPRKEGTLRMVVDYRPLNKYVKPNVYPLPLIEQLLAKIQGSTIFTKLDLKSAYHQIRVRKGD
EHKLAFRCPRGVFEYLVMPYGIKTAPAHFQYFINTILGEAKESHVVCYMDDILIHSKSESEHVKHVKDVLQK
LKNANLIINQAKCEFHQSQVKFLGYHISEKGLTPCQENIDKVLQWKQPKNQKELRQFLGQVNYLRKFIPKT
SQLTHPLNKLLKKDVRWKWTPTQTQAIENIKQCLVSPPVLRHFDFSKKILLETDVSDVAVGAVLSQKHDDD
KYYPVGYYSAKMSKAQLNYSVSDKEMLAIIKSLEHWRHYLESTIEPFKILTDHRNLIGRITNESEPENKRLA
RWQLFLQDFNFEINYRPGSANHIADALSRIVDETEPIPKDNEDNSINFVNQISISGGSKRTADGSEFESPKK
KRKVGSGPAAKRVKLD

Start	End	Feature Description
1	19	SV40 BP NLS
20	1386	SpCas9 (R221K, N394K, H840A) - VRQR PAM variant
1387	1420	(SGGS)2 – SV40 NLS – SGGS2 optimized flexible linker

1421	1925	PE6c & evolved reverse transcriptase from Tf1
1926	1959	Linker and SV40 BP NLS

SpCas9 – VRQR variant – PE6d
Plasmid ID: pHS0243

DNA sequence

GACATTGATTATTGACTAGTTATTAATAGTAATCAATTACGGGGTCATTAGTTCATAGCCCATATATGGAG
TTCCGCGTTACATAACTTACGGTAAATGGCCCGCCTGGCTGACCGCCCAACGACCCCCGCCCATTGA
CGTCAATAATGACGTATGTTCCCATAGTAACGCCAATAGGGACTTTCCATTGACGTCAATGGGTGGAGT
ATTTACGGTAAACTGCCCACTTGGCAGTACATCAAGTGTATCATATGCCAAGTACGCCCCCTATTGACG
TCAATGACGGTAAATGGCCCGCCTGGCATTATGCCCAGTACATGACCTTATGGGACTTTCCTACTTGG
CAGTACATCTACGTATTAGTCATCGCTATTACCATGGTGATGCGGTTTTGGCAGTACATCAATGGGCGT
GGATAGCGGTTTGACTCACGGGGATTTCCAAGTCTCCACCCCATTGACGTCAATGGGAGTTTGTTTTG
GCACCAAAATCAACGGGACTTTCCAAAATGTCGTAACAACTCCGCCCCATTGACGCAAATGGGCGGT
AGGCGTGTACGGTGGGAGGTCTATATAAGCAGAGCTGGTTTAGTGAACCGTCAGATCCGCTAGAGAT
CCGCGGCCGCTAATACGACTCACTATAGGGAGAGCCGCCACCATGAAACGGACAGCCGACGGAAGC
GAGTTCGAGTCACCAAAGAAGAAGCGGAAAGTCGACAAGAAGTACAGCATCGGCCTGGACATCGGC
ACCAACTCTGTGGGCTGGGCCGTGATCACCGACGAGTACAAGGTGCCCAGCAAGAAATTCAAGGTG
CTGGGCAACACCGACCGGCACAGCATCAAGAAGAACCTGATCGGAGCCCTGCTGTTCGACAGCGGC
GAAACAGCCGAGGCCACCCGGCTGAAGAGAACCGCCAGAAGAAGATACACCAGACGGAAGAACCG
GATCTGCTATCTGCAAGAGATCTTCAGCAACGAGATGGCCAAGGTGGACGACAGCTTCTTCCACAGA
CTGGAAGAGTCCTTCCTGGTGGAAGAGGATAAGAAGCACGAGCGGCACCCCATCTTCGGCAACATC
GTGGACGAGGTGGCCTACCACGAGAAGTACCCCACCATCTACCACCTGAGAAAGAAACTGGTGGAC
AGCACCGACAAGGCCGACCTGCGGCTGATCTATCTGGCCCTGGCCCACATGATCAAGTTCCGGGGC
CACTTCCTGATCGAGGGCGACCTGAACCCCGACAACAGCGACGTGGACAAGCTGTTCATCCAGCTG
GTGCAGACCTACAACCAGCTGTTCGAGGAAAACCCCATCAACGCCAGCGGCGTGGACGCCAAGGCC
ATCCTGTCTGCCAGACTGAGCAAGAGCAGAAAGCTGGAAAATCTGATCGCCCAGCTGCCCGGCGAG
AAGAAGAATGGCCTGTTCGGAAACCTGATTGCCCTGAGCCTGGGCCTGACCCCCAACTTCAAGAGC
AACTTCGACCTGGCCGAGGATGCCAAACTGCAGCTGAGCAAGGACACCTACGACGACGACCTGGAC
AACCTGCTGGCCCAGATCGGCGACCAGTACGCCGACCTGTTTCTGGCCGCCAAGAACCTGTCCGAC
GCCATCCTGCTGAGCGACATCCTGAGAGTGAACACCGAGATCACCAAGGCCCCCCTGAGCGCCTCT
ATGATCAAGAGATACGACGAGCACCACCAGGACCTGACCCTGCTGAAAGCTCTCGTGCGGCAGCAG
CTGCCTGAGAAGTACAAAGAGATTTTCTTCGACCAGAGCAAGAACGGCTACGCCGGCTACATTGACG
GCGGAGCCAGCCAGGAAGAGTTCTACAAGTTCATCAAGCCCATCCTGGAAAAGATGGACGGCACCG
AGGAACTGCTCGTGAAGCTGAAGAGAGAGGACCTGCTGCGGAAGCAGCGGACCTTCGACAACGGC
AGCATCCCCCACCAGATCCACCTGGGAGAGCTGCACGCCATTCTGCGGCGGCAGGAAGATTTTTACC
CATTCCTGAAGGACAACCGGGAAAAGATCGAGAAGATCCTGACCTTCCGCATCCCCTACTACGTGGG
CCCTCTGGCCAGGGGAAACAGCAGATTCGCCTGGATGACCAGAAAGAGCGAGGAAACCATCACCCC
CTGGAACTTCGAGGAAGTGGTGGACAAGGGCGCTTCCGCCCAGAGCTTCATCGAGCGGATGACCAA
CTTCGATAAGAACCTGCCCAACGAGAAGGTGCTGCCCAAGCACAGCCTGCTGTACGAGTACTTCACC
GTGTATAACGAGCTGACCAAAGTGAAATACGTGACCGAGGGAATGAGAAAGCCCGCCTTCCTGAGCG
GCGAGCAGAAAAAGGCCATCGTGGACCTGCTGTTCAAGACCAACCGGAAAGTGACCGTGAAGCAGC
TGAAAGAGGACTACTTCAAGAAAATCGAGTGCTTCGACTCCGTGGAAATCTCCGGCGTGGAAGATCG
GTTCAACGCCTCCCTGGGCACATACCACGATCTGCTGAAAATTATCAAGGACAAGGACTTCCTGGACA
ATGAGGAAAACGAGGACATTCTGGAAGATATCGTGCTGACCCTGACACTGTTTGAGGACAGAGAGAT
GATCGAGGAACGGCTGAAAACCTATGCCCACCTGTTCGACGACAAAGTGATGAAGCAGCTGAAGCG
GCGGAGATACACCGGCTGGGGCAGGCTGAGCCGGAAGCTGATCAACGGCATCCGGGACAAGCAGT
CCGGCAAGACAATCCTGGATTTCCTGAAGTCCGACGGCTTCGCCAACAGAAACTTCATGCAGCTGAT
CCACGACGACAGCCTGACCTTTAAAGAGGACATCCAGAAAGCCCAGGTGTCCGGCCAGGGCGATAG
CCTGCACGAGCACATTGCCAATCTGGCCGGCAGCCCCGCCATTAAGAAGGGCATCCTGCAGACAGT
GAAGGTGGTGGACGAGCTCGTGAAAGTGATGGGCCGGCACAAGCCCGAGAACATCGTGATCGAAAT
GGCCAGAGAGAACCAGACCACCCAGAAGGGACAGAAGAACAGCCGCGAGAGAATGAAGCGGATCG
AAGAGGGCATCAAAGAGCTGGGCAGCCAGATCCTGAAAGAACACCCCGTGGAAAACACCCAGCTGC
AGAACGAGAAGCTGTACCTGTACTACCTGCAGAATGGGCGGGATATGTACGTGGACCAGGAACTGGA
CATCAACCGGCTGTCCGACTACGATGTGGACGCTATCGTGCCTCAGAGCTTTCTGAAGGACGACTCC
ATCGACAACAAGGTGCTGACCAGAAGCGACAAGAACCGGGGCAAGAGCGACAACGTGCCCTCCGA
AGAGGTCGTGAAGAAGATGAAGAACTACTGGCGGCAGCTGCTGAACGCCAAGCTGATTACCCAGAG
AAAGTTCGACAATCTGACCAAGGCCGAGAGAGGCGGCCTGAGCGAACTGGATAAGGCCGGCTTCAT

CAAGAGACAGCTGGTGGAAACCCGGCAGATCACAAAGCACGTGGCACAGATCCTGGACTCCCGGAT
GAACACTAAGTACGACGAGAATGACAAGCTGATCCGGGAAGTGAAAGTGATCACCCTGAAGTCCAAG
CTGGTGTCCGATTTCCGGAAGGATTTCCAGTTTTACAAAGTGCGCGAGATCAACAACTACCACCACGC
CCACGACGCCTACCTGAACGCCGTCGTGGGAACCGCCCTGATCAAAAAGTACCCTAAGCTGGAAAG
CGAGTTCGTGTACGGCGACTACAAGGTGTACGACGTGCGGAAGATGATCGCCAAGAGCGAGCAGGA
AATCGGCAAGGCTACCGCCAAGTACTTCTTCTACAGCAACATCATGAACTTTTTCAAGACCGAGATTAC
CCTGGCCAACGGCGAGATCCGGAAGCGGCCTCTGATCGAGACAAACGGCGAAACCGGGGAGATCG
TGTGGGATAAGGGCCGGGATTTTGCCACCGTGCGGAAAGTGCTGAGCATGCCCCAAGTGAATATCGT
GAAAAAGACCGAGGTGCAGACAGGCGGCTTCAGCAAAGAGTCTATCCTGCCCAAGAGGAACAGCGA
TAAGCTGATCGCCAGAAAGAAGGACTGGGACCCTAAGAAGTACGGCGGCTTCGTGAGCCCCACCGT
GGCCTATTCTGTGCTGGTGGTGGCCAAAGTGGAAAAGGGCAAGTCCAAGAAACTGAAGAGTGTGAA
AGAGCTGCTGGGGATCACCATCATGGAAAGAAGCAGCTTCGAGAAGAATCCCATCGACTTTCTGGAA
GCCAAGGGCTACAAAGAAGTGAAAAAGGACCTGATCATCAAGCTGCCTAAGTACTCCCTGTTCGAGC
TGGAAAACGGCCGGAAGAGAATGCTGGCCTCAGCCAGAGAACTGCAGAAGGGAAACGAACTGGCC
CTGCCCTCCAAATATGTGAACTTCCTGTACCTGGCCAGCCACTATGAGAAGCTGAAGGGCTCCCCCG
AGGATAATGAGCAGAAACAGCTGTTTGTGGAACAGCACAAGCACTACCTGGACGAGATCATCGAGCA
GATCAGCGAGTTCTCCAAGAGAGTGATCCTGGCCGACGCTAATCTGGACAAAGTGCTGTCCGCCTAC
AACAAGCACCGGGATAAGCCCATCAGAGAGCAGGCCGAGAATATCATCCACCTGTTTACCCTGACCA
ATCTGGGAGCCCCTGCCGCCTTCAAGTACTTTGACACCACCATCGACCGGAAGCAGTACAGAAGCAC
CAAAGAGGTGCTGGACGCCACCCTGATCCACCAGAGCATCACCGGCCTGTACGAGACACGGATCGA
CCTGTCTCAGCTGGGAGGTGACTCCGGCGGAAGCTCTGGTGGCAGCAAGCGGACCGCCGACGGCT
CTGAATTCGAGAGCCCTAAGAAGAAAAGAAAGGTGAGCGGAGGCTCTAGCGGCGGAAGCACCCTGA
ACATTGAAGACGAGTATAGACTGCATGAAACAAGCAAGGAACCCGACGTGTCCCTGGGCTCCACCTG
GCTGTCCGACTTTCCCCAGGCCTGGGCCGAGACAGGAGGAATGGGCCTGGCCGTGCGGCAGGCAC
CCCTGATCATCCCTCTGAAGGCCACCTCTACACCCGTGAGCATCAAGCAGTACCCTATGTCTCAGGA
GGCCAGACTGGGCATCAAGCCTCACATCCAGAGGCTGCTGGACCAGGGCATCCTGGTGCCATGCCA
GAGCCCCTGGAACACACCACTGCTGCCCGTGAAGAAGCCAGGCACCAATGACTATAGACCCGTGCA
GGATCTGAGAGAGGTGAACAAGAGGGTGGAGGATATCCACCCCAACGTGCCCAACCCTTACAATCTG
CTGTCCGGCCTGCCCCCTTCTCACCAGTGGTATACAGTGCTGGACCTGAAGGATGCCTTCTTTTGTC
TGAGACTGCACCCTACCAGCCAGCCACTGTTCGCCTTTGAGTGGAGGGACCCTGAGATGGGCATCT
CTGGCCAGCTGACCTGGACACGCCTGCCTCAGGGCTTCAAGAATAGCCCAACACTGTTTTGCGAGG
CCCTGCACCGCGACCTGGCAGATTTCCGGATCCAGCACCCAGATCTGATCCTGCTGCAGTACTACGA
CGATCTGCTGCTGGCCGCCACCAGCGAGCTGGATTGCCAGCAGGGAACACGCGCCCTGCTGCAGA
CCCTGGGAAACCTGGGATATAGGGCATCCGCCAAGAAGGCCCAGATCTGTCAGAAGCAGGTGAAGT
ACCTGGGCTATCTGCTGAAGGAGGGCCAGAGATGGCTGACAGAGGCCAGGAAGGAGACAGTGATG
GGCCAGCCAACACCCAAGACCCCAAGACAGCTGAGGGAGTTCCTGGGCAAAGCAGGATTTTGCAGG
CTGTTCATCCCAGGATTCGCAGAGATGGCAGCACCTCTGTACCCACTGACCAAGCCGGGCACCCTGT
TTAATTGGGGCCCTGACCAGCAGAAGGCCTATCAGGAGATCAAGCAGGCCCTGCTGACAGCACCAG
CCCTGGGCCTGCCAGACCTGACCAAGCCTTTCGAGCTGTTTGTGGATGAGAAGCAGGGCTACGCCA
AGGGCGTGCTGACCCAGAAGCTGGGACCATGGAGACGGCCCGTGGCCTATCTGTCCAAGAAGCTG
GACCCAGTGGCAGCAGGATGGCCACCATGCCTGAGGATGGTGGCAGCAATCGCCGTGCTGACAAAG
GATGCCGGCAAGCTGACCATGGGACAGCCACTGGTCATCCTGGCACCACACGCAGTGGAGGCCCT
GGTGAAGCAGCCTCCAGATCGCTGGCTGTCTAACGCCCGGATGACACACTACCAGGCCCTGCTGCT
GGACACCGATCGCGTGCAGTTTGGCCCTGTGGTGGCCCTGAATCCAGCCACCCTGCTGCCTCTGCC
AGAGGAGGGCCTGCAGCACAACTGTCTGGACTCTGGCGGCTCAAAAAGAACCGCCGACGGCAGCG
AATTCGAGTCTCCCAAGAAGAAGAGGAAAGTCGGCTCTGGCCCTGCCGCTAAGAGAGTGAAGCTGG
ACTAACCGGTCATCATCACCATCACCATTGAGTTTAAACCCGCTGATCAGCCTCGACTGTGCCTTCTA
GTTGCCAGCCATCTGTTGTTTGCCCCTCCCCCGTGCCTTCCTTGACCCTGGAAGGTGCCACTCCCAC
TGTCCTTTCCTAATAAAATGAGAAATTGCATCGCATTGTCTGAGTAGGTGTCATTCTATTCTGGGGGG
TGGGGTGGGGCAGGACAGCAAGGGGGAGGATTGGGAAGACAATAGCAGGCATGCTGGGGATGCGG
TGGGCTCTATGG

Start	End	Feature Description
1	380	CMV enhancer

381	597	CMV promoter
598	657	5’ UTR (contains T7 promoter)
658	714	SV40 BP NLS
715	4815	SpCas9 (R221K, N394K, H840A) - VRQR PAM variant
4816	4917	(SGGS)2 – SV40 NLS – SGGS2 optimized flexible linker
4918	6408	PE6d evolved MMLV reverse transcriptase
6409	6513	Linker and SV40 BP NLS
6514	6564	3’ UTR
6565	6789	bGH poly(A) signal

Amino acid sequence

MKRTADGSEFESPKKKRKVDKKYSIGLDIGTNSVGWAVITDEYKVPSKKFKVLGNTDRHSIKKNLIGALLFD
SGETAEATRLKRTARRRYTRRKNRICYLQEIFSNEMAKVDDSFFHRLEESFLVEEDKKHERHPIFGNIVDE
VAYHEKYPTIYHLRKKLVDSTDKADLRLIYLALAHMIKFRGHFLIEGDLNPDNSDVDKLFIQLVQTYNQLFEE
NPINASGVDAKAILSARLSKSRKLENLIAQLPGEKKNGLFGNLIALSLGLTPNFKSNFDLAEDAKLQLSKDT
YDDDLDNLLAQIGDQYADLFLAAKNLSDAILLSDILRVNTEITKAPLSASMIKRYDEHHQDLTLLKALVRQQL
PEKYKEIFFDQSKNGYAGYIDGGASQEEFYKFIKPILEKMDGTEELLVKLKREDLLRKQRTFDNGSIPHQIH
LGELHAILRRQEDFYPFLKDNREKIEKILTFRIPYYVGPLARGNSRFAWMTRKSEETITPWNFEEVVDKGAS
AQSFIERMTNFDKNLPNEKVLPKHSLLYEYFTVYNELTKVKYVTEGMRKPAFLSGEQKKAIVDLLFKTNRK
VTVKQLKEDYFKKIECFDSVEISGVEDRFNASLGTYHDLLKIIKDKDFLDNEENEDILEDIVLTLTLFEDREMI
EERLKTYAHLFDDKVMKQLKRRRYTGWGRLSRKLINGIRDKQSGKTILDFLKSDGFANRNFMQLIHDDSLT
FKEDIQKAQVSGQGDSLHEHIANLAGSPAIKKGILQTVKVVDELVKVMGRHKPENIVIEMARENQTTQKGQ
KNSRERMKRIEEGIKELGSQILKEHPVENTQLQNEKLYLYYLQNGRDMYVDQELDINRLSDYDVDAIVPQS
FLKDDSIDNKVLTRSDKNRGKSDNVPSEEVVKKMKNYWRQLLNAKLITQRKFDNLTKAERGGLSELDKAG
FIKRQLVETRQITKHVAQILDSRMNTKYDENDKLIREVKVITLKSKLVSDFRKDFQFYKVREINNYHHAHDAY
LNAVVGTALIKKYPKLESEFVYGDYKVYDVRKMIAKSEQEIGKATAKYFFYSNIMNFFKTEITLANGEIRKRP
LIETNGETGEIVWDKGRDFATVRKVLSMPQVNIVKKTEVQTGGFSKESILPKRNSDKLIARKKDWDPKKYG
GFVSPTVAYSVLVVAKVEKGKSKKLKSVKELLGITIMERSSFEKNPIDFLEAKGYKEVKKDLIIKLPKYSLFEL
ENGRKRMLASARELQKGNELALPSKYVNFLYLASHYEKLKGSPEDNEQKQLFVEQHKHYLDEIIEQISEFS
KRVILADANLDKVLSAYNKHRDKPIREQAENIIHLFTLTNLGAPAAFKYFDTTIDRKQYRSTKEVLDATLIHQS
ITGLYETRIDLSQLGGDSGGSSGGSKRTADGSEFESPKKKRKVSGGSSGGSTLNIEDEYRLHETSKEPDV
SLGSTWLSDFPQAWAETGGMGLAVRQAPLIIPLKATSTPVSIKQYPMSQEARLGIKPHIQRLLDQGILVPC
QSPWNTPLLPVKKPGTNDYRPVQDLREVNKRVEDIHPNVPNPYNLLSGLPPSHQWYTVLDLKDAFFCLR
LHPTSQPLFAFEWRDPEMGISGQLTWTRLPQGFKNSPTLFCEALHRDLADFRIQHPDLILLQYYDDLLLAA
TSELDCQQGTRALLQTLGNLGYRASAKKAQICQKQVKYLGYLLKEGQRWLTEARKETVMGQPTPKTPRQ
LREFLGKAGFCRLFIPGFAEMAAPLYPLTKPGTLFNWGPDQQKAYQEIKQALLTAPALGLPDLTKPFELFVD
EKQGYAKGVLTQKLGPWRRPVAYLSKKLDPVAAGWPPCLRMVAAIAVLTKDAGKLTMGQPLVILAPHAVE
ALVKQPPDRWLSNARMTHYQALLLDTDRVQFGPVVALNPATLLPLPEEGLQHNCLDSGGSKRTADGSEF
ESPKKKRKVGSGPAAKRVKLD

Start	End	Feature Description
1	19	SV40 BP NLS
20	1386	SpCas9 (R221K, N394K, H840A) - VRQR PAM variant
1387	1420	(SGGS)2 – SV40 NLS – SGGS2 optimized flexible linker

1421	1917	PE6d evolved MMLV reverse transcriptase
1918	1951	Linker and SV40 BP NLS
