# Supplementary material for: In vivo prime editing rescues alternating hemiplegia of childhood in mice
Source: Cell. Author manuscript; Available in PMC 2025 Dec 14. (PMC12702498; doi:10.1016/j.cell.2025.06.038)
Supplement: 1 [file NIHMS2127386-supplement-1.pdf]

Supplemental figures

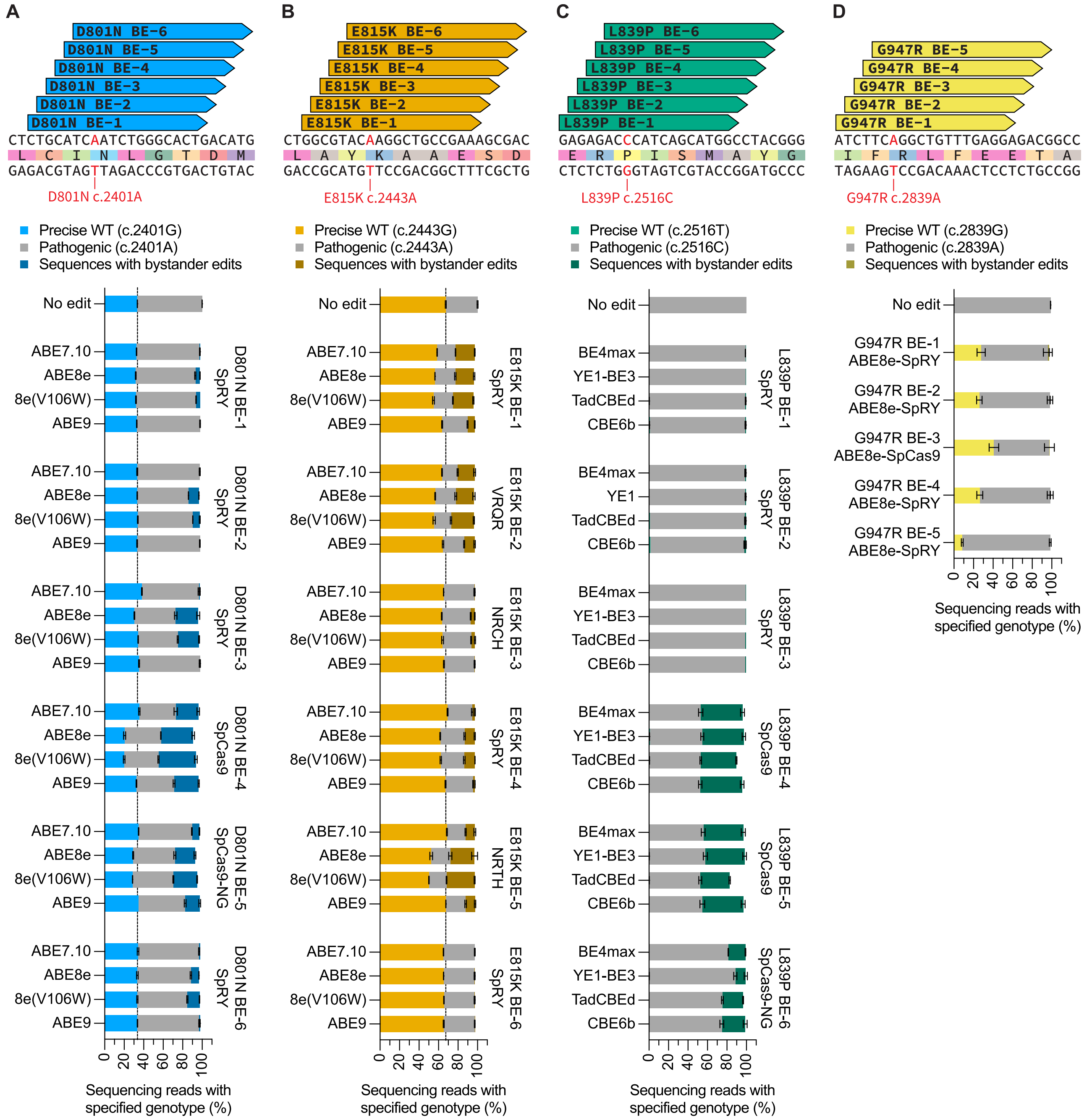

Figure S1. BE of AHC-causing ATP1A3 mutations, related to Figure 1

(A–D) Top: Schematic of *ATP1A3* mutations with annotated protospacers of sgRNAs for ABE or CBE correction. Bottom: Base editing correction with Cas9(D10A) DNA binding domain variants SpRY,[111] SpCas9,[161] and SpCas9-NG,[162] VRQR,[92] NRCH, and NRTH[163] and with ABE deaminases ABE7.10,[42] ABE8e, ABE8e (V106W)[85] (listed as 8e[V106W]), and ABE9[164] or with CBE deaminases BE4max,[165] YE1-BE3,[166] TadCBEd,[167] and CBE6b[168] used in the indicated combinations for each protospacer.

(legend continued on next page)

(A) ABE correction of *ATP1A3* D801N c.2401A.
(B) ABE correction of *ATP1A3* E815K c.2443A.
(C) CBE correction of *ATP1A3* L839P c.2516C.
(D) ABE correction of *ATP1A3* G947R c.2839A.
Data and error bars represent the mean ± SD of n = 3 independent biological replicates. HEK293T cell lines are presumed triploid at the *ATP1A3* locus, and the baseline unedited genotype (“No edit”) is indicated with a dashed line where applicable in (A) and (B).

CellPress
OPEN ACCESS

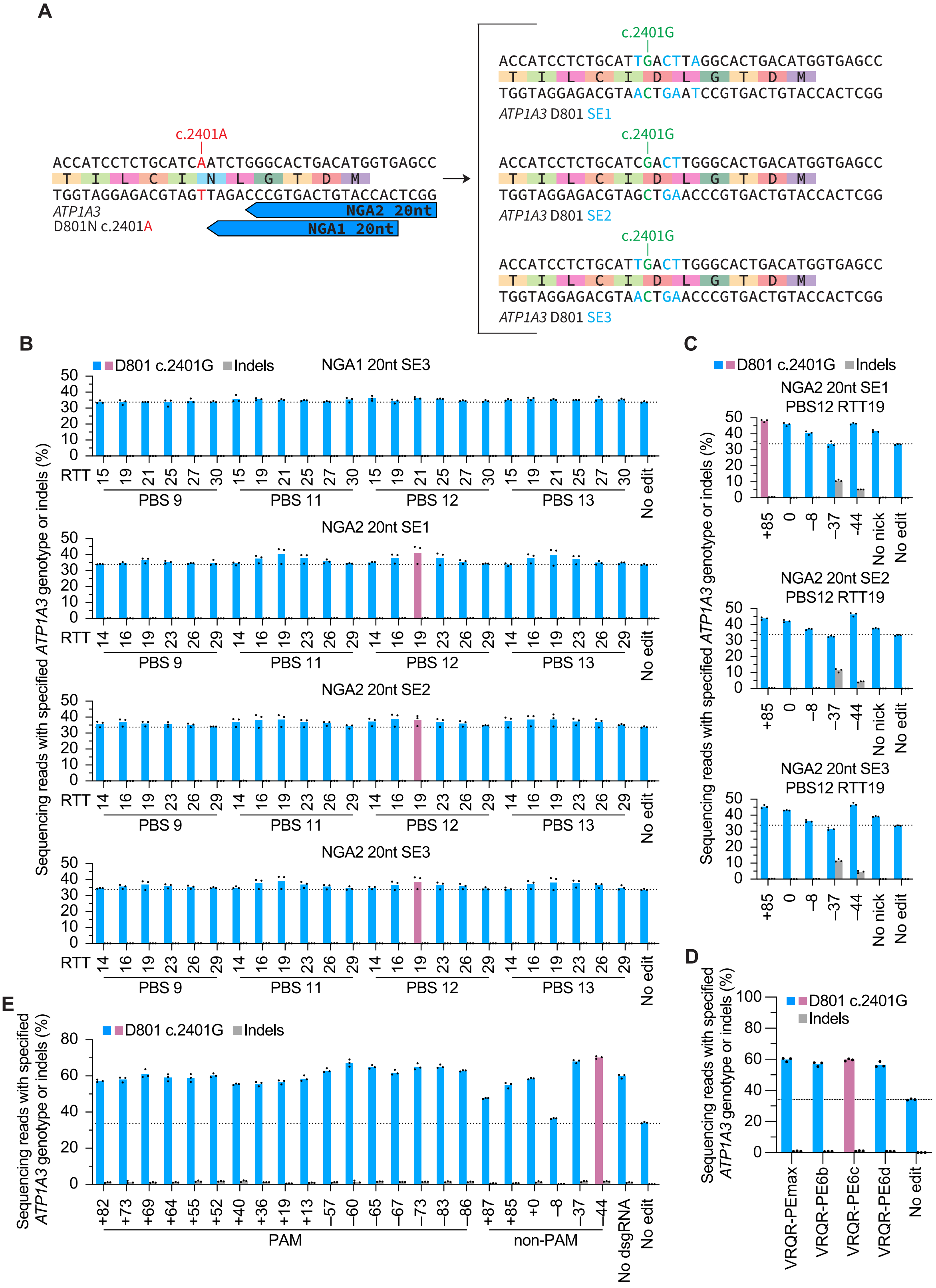

(legend on next page)

Figure S2. Optimization of PE strategies to correct *ATP1A3* D801N c.2401A, related to Figure 1

(A) Left: The pathogenic D801N mutation targeted for PE correction (red) and the two epegRNA protospacers (NGA1 20-nt and NGA2 20-nt) used for PE. Right: The corrective edit (green) with alternate silent editing strategies 1–3 (SE1–SE3, blue).

(B–E) HEK293T cells with the D801N mutation were transfected with plasmid reagents to iteratively optimize each editing component for D801N correction.

(B) Optimization of epegRNA spacer selection, silent edit strategies, and PBS and RTT lengths with SpCas9(VRQR)-PEmax, identifying three top-performing epegRNAs (purple) for subsequent experiments.

(C) Optimization of ngRNA spacer selection using previously optimized epegRNAs, identifying a top performing epegRNA-ngRNA combination (purple) for subsequent experiments.

(D) Optimization of SpCas9(VRQR)-PE variant using the previously optimized epegRNA and ngRNA, identifying SpCas9(VRQR)-PE6c (purple) for subsequent experiments.

(E) Optimization of dsgRNA spacer selection using previously optimized editor, epegRNA, and ngRNA, identifying a top performing dsgRNA (purple) for subsequent experiments.

For (B)–(E), unedited HEK293T samples (“No edit”) start at approximately 33% wild-type D801 c.2401G genotype, marked by a dotted line on each plot. See STAR Methods for details. Data represent the mean of n = 3 independent biological replicates, and dots show individual replicates.

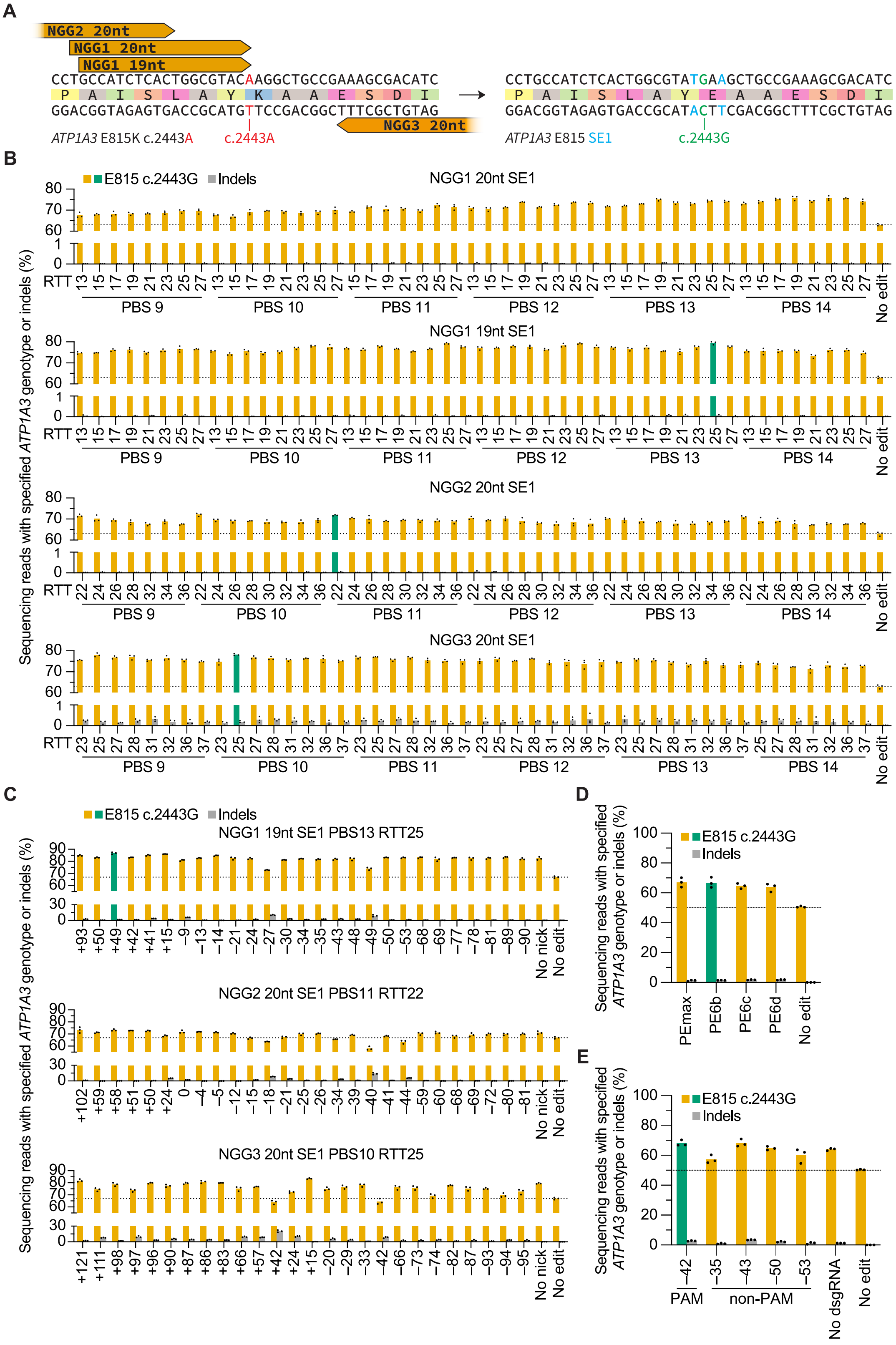

(legend on next page)

Figure S3. Optimization of PE strategies to correct *ATP1A3* E815K c.2443A, related to Figure 1

(A) Left: The pathogenic E815K mutation targeted for PE correction (red) and the four epegRNA protospacers (NGG1 20-nt, NGG1 19-nt, NGG2 20-nt, and NGG3 20-nt) used for PE. Right: The corrective edit (green) with the concomitantly installed silent editing strategy 1 (SE1, blue).

(B–E) HEK293T cells or patient-derived iPSCs with the E815K mutation were transfected with PE plasmid reagents (B and C) or electroporated with RNA reagents (D and E), respectively, to iteratively optimize each editing component for E815K correction.

(B) Optimization of epegRNA spacer selection, PBS lengths, and RTT lengths with PEmax, identifying three top performing epegRNAs (green) for subsequent experiments.

(C) Optimization of ngRNA spacer selection using previously optimized epegRNAs, identifying a top performing epegRNA-ngRNA combination (green) for subsequent experiments.

(D) Optimization of prime editor variant using previously optimized pegRNA and ngRNA, identifying PE6b (green) for subsequent experiments.

(E) Optimization of dsgRNA spacer selection using previously optimized editor, pegRNA, and ngRNA, identifying a top performing dsgRNA (green) for subsequent editing experiments.

For (B)–(E), unedited HEK293T and iPSC samples (“No edit”) start at approximately 66% and 50% wild-type E815 c.2443G genotype, respectively, marked by a dotted line on each plot. See STAR Methods for details. Data represent the mean of $n = 3$ independent biological replicates, and dots show individual replicates.

CellPress
OPEN ACCESS

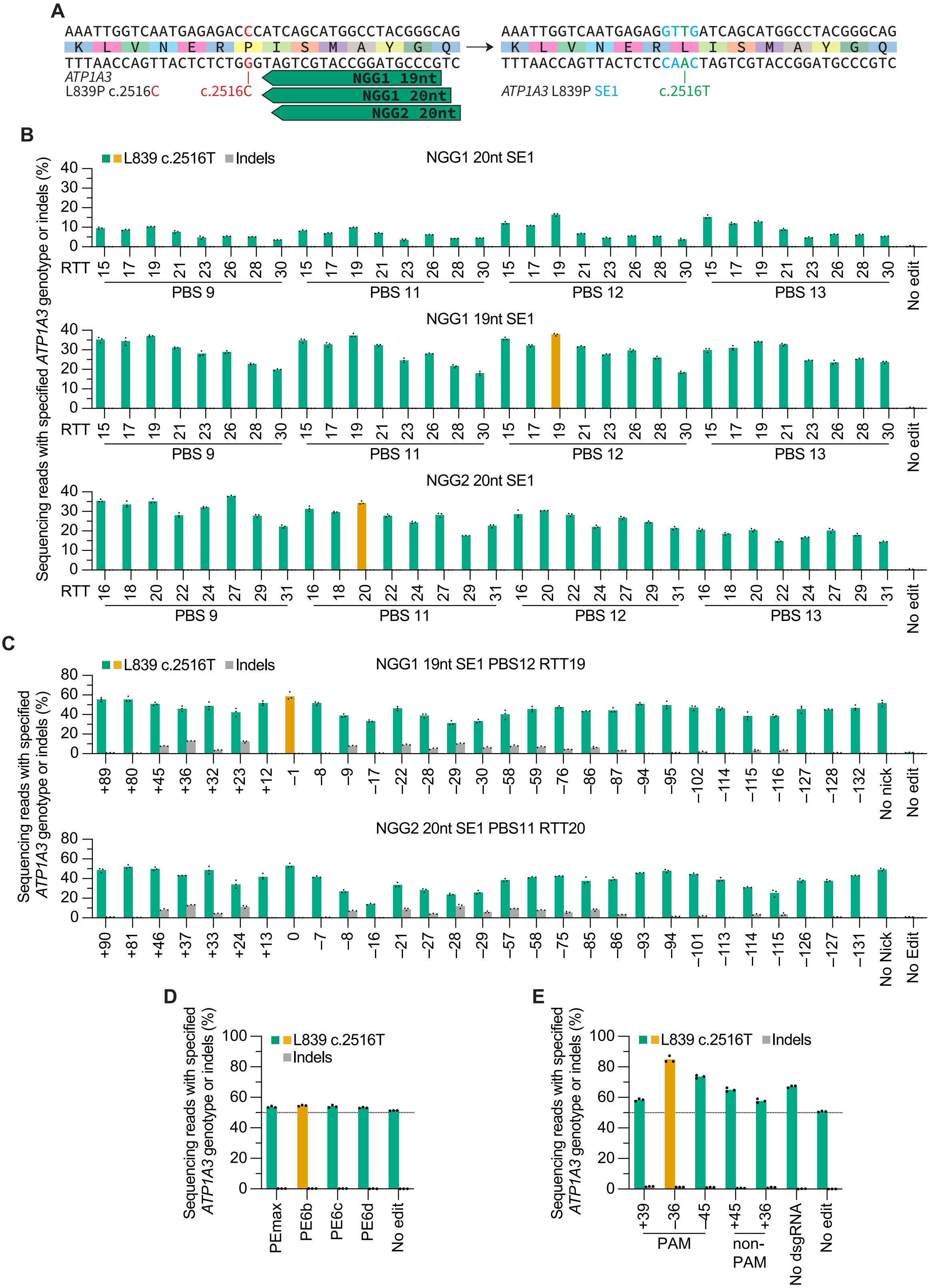

(legend on next page)

Figure S4. Optimization of PE strategies to correct *ATP1A3* L839P c.2516C, related to Figure 1

(A) Left: The pathogenic L839P mutation targeted for PE correction (red) and the three epegRNA protospacers (NGG1 19-nt, NGG1 20-nt, and NGG2 20-nt) used for PE. Right: The corrective edit (green) with the concomitantly installed silent editing strategy 1 (SE1, blue).

(B–E) HEK293T cells or patient-derived iPSCs with the L839P mutation were transfected with PE plasmid reagents (B and C) or electroporated with RNA reagents (D and E), respectively, to iteratively optimize each editing component for L839P correction.

(B) Optimization of epegRNA spacer selection, PBS lengths, and RTT lengths with PEmax, identifying two top-performing epegRNAs (orange) for subsequent experiments.

(C) Optimization of ngRNA spacer selection using previously optimized epegRNAs, identifying a top performing epegRNA-ngRNA combination (orange) for subsequent experiments.

(D) Optimization of prime editor variant using previously optimized pegRNA and ngRNA, identifying PE6b (orange) for subsequent experiments.

(E) Optimization of dsgRNA spacer selection using previously optimized editor, epegRNA, and ngRNA, identifying a top performing dsgRNA (orange) for subsequent experiments.

For (B)–(E), unedited HEK293T and iPSC samples (“No edit”) start at approximately 0% and 50% wild-type L839 c.2516T genotype, respectively, marked by a dotted line on each plot. See STAR Methods for details. Data represent the mean of n = 3 independent biological replicates, and dots show individual replicates.

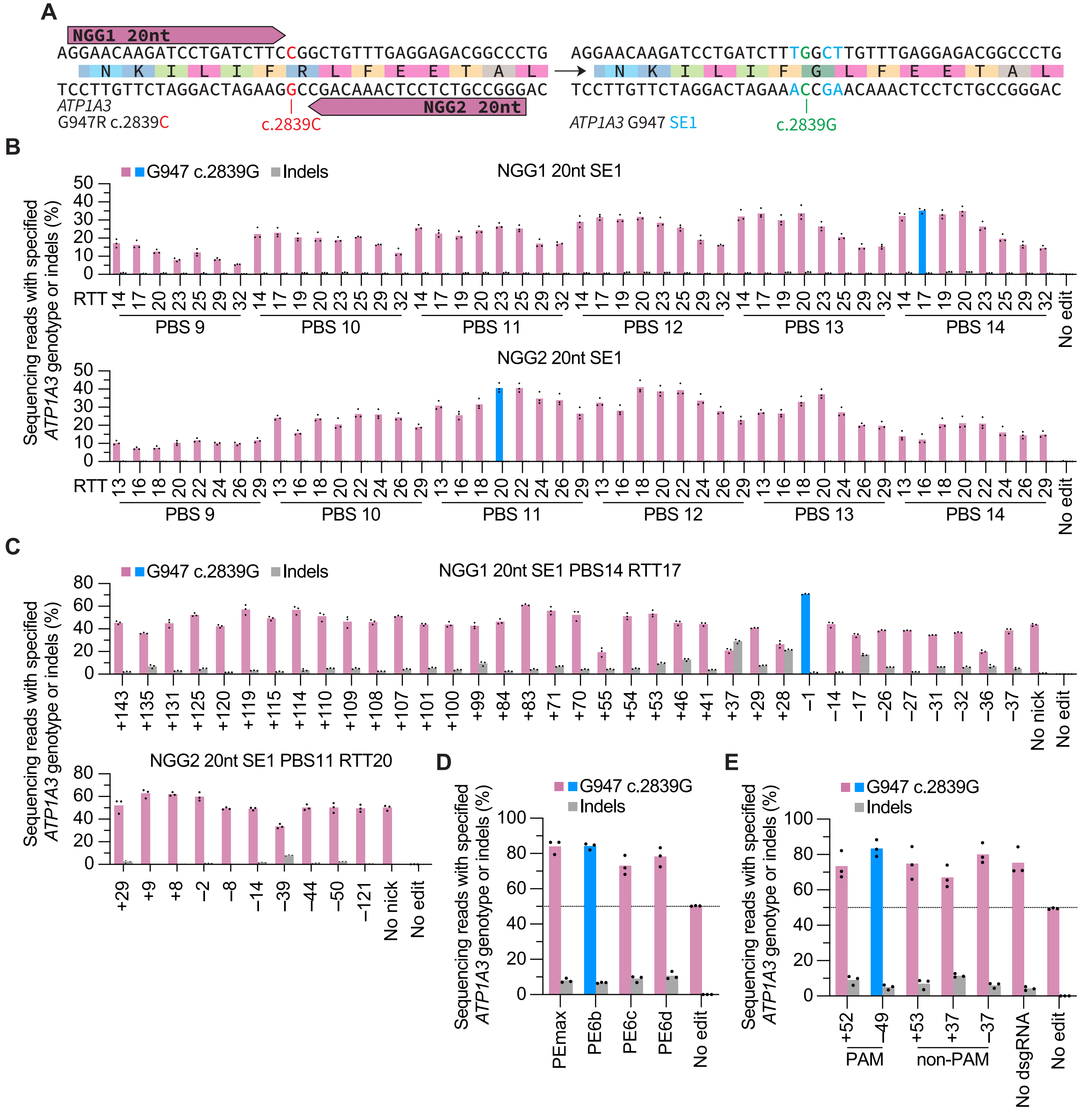

Figure S5. Optimization of PE strategies to correct *ATP1A3* G947R c.2839C, related to Figure 1

(A) Left: The pathogenic G947R-C mutation targeted for PE correction (red) and the two epegRNA protospacers (NGG1 20-nt and NGG2 20-nt) used for PE. Right: The corrective edit (green) with the concomitantly installed silent editing strategy 1 (SE1, in blue).

(B–E) HEK293T cells or patient-derived iPSCs with the G947R-C mutation were transfected with PE plasmid reagents (B and C) or electroporated with RNA reagents (D and E), respectively, to iteratively optimize each editing component for L839P correction.

(B) Optimization of epegRNA spacer selection, PBS lengths, and RTT lengths with PEmax, identifying two top-performing epegRNAs (blue) for subsequent experiments.

(C) Optimization of ngRNA spacer selection using previously optimized epegRNAs, identifying a top performing epegRNA-ngRNA combination (blue) for subsequent experiments.

(D) Optimization of prime editor variant using previously optimized pegRNA and ngRNA, identifying PE6b (blue) for subsequent experiments.

(legend continued on next page)

(E) Optimization of dsgRNA spacer selection using previously optimized editor, pegRNA, and ngRNA, identifying a top performing dsgRNA (blue) for subsequent experiments.

For (B)–(E), unedited HEK293T cells and iPSC samples (“No edit”) start at approximately 0% and 50% wild-type G947 c.2839C genotype, respectively, marked by a dotted line on each plot. See STAR Methods for details. Data represent the mean of n = 3 independent biological replicates, and dots show individual replicates.

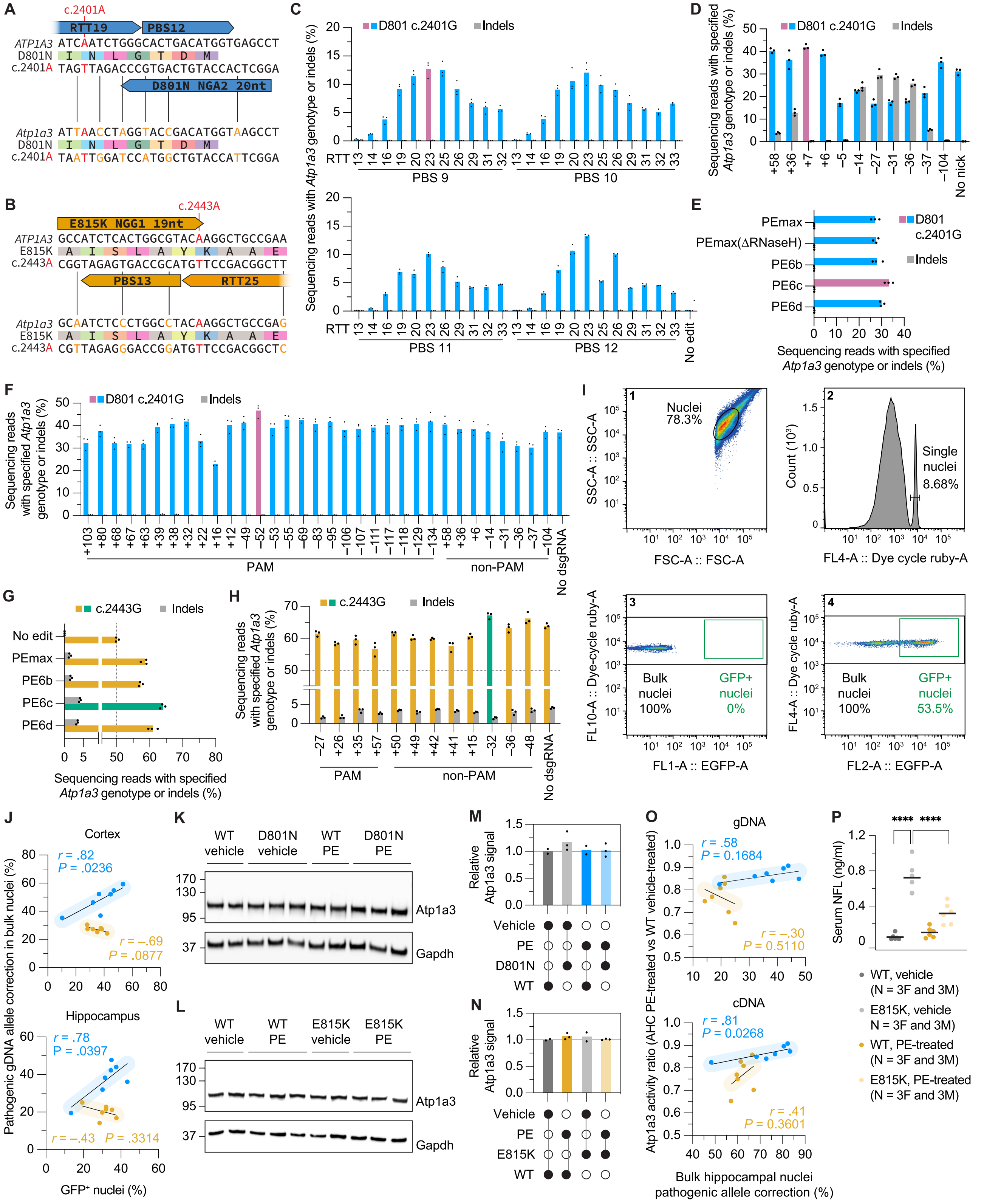

(legend on next page)

Figure S6. Optimization of PE strategies to correct *Atp1a3* D801N c.2401A and *Atp1a3* E815K c.2443A, related to Figure 3

(A and B) Comparison of human *ATP1A3* and mouse *Atp1a3* targets for (A) D801N c.2401A and (B) E815K c.2443A near the pathogenic mutation (red). Sequence differences in *Atp1a3* (orange) are linked to corresponding human base pairs (black lines). Optimized epegRNA sequence elements from Figure 1 are annotated on the *ATP1A3* sequence; silent edits have been omitted from RTT annotations for clarity.

(C–F) Monoclonal N2a cells homozygous for the *Atp1a3* D801N c.2401A mutation were transfected with prime editing plasmid reagents to iteratively optimize each editing component. Unedited N2a cells (“No edit”) start at approximately 0% wild-type D801 c.2401G genotype. See STAR Methods for details.

(C) Optimization of epegRNA PBS lengths and RTT lengths with PEmax, identifying a top performing epegRNA (purple) for subsequent experiments.

(D) Optimization of ngRNA spacer selection using previously optimized epegRNA, identifying a top performing ngRNA (purple) for subsequent experiments.

(E) Optimization of prime editor variant using previously optimized epegRNA and ngRNA, identifying PE6c (purple) for subsequent experiments.

(F) Optimization of dsgRNA spacer selection using previously optimized editor, epegRNA, and ngRNA, identifying a top performing dsgRNA (purple) for experiments.

(G and H) E815K mouse primary fibroblasts were electroporated with prime editing RNA reagents to iteratively optimize (G) prime editor variant and (H) dsgRNA spacer selection using previously identified pegRNA and ngRNA components adapted from the human E815K correction strategy, identifying a top-performing editor PE6 (G, green) and dsgRNA (H, green) for subsequent experiments. Unedited mouse primary fibroblasts (“No edit”) start at approximately 50% wild-type E815 c.2443G genotype. See Methods for details.

(I) Representative fluorescence-activated cell sorting (FACS) gating strategy for brain nuclei. Panels show isolated nuclei (1), singlets (2), and GFP gating for vehicle-treated D801N mice (3) and D801N-PE-AAV9-treated D801N mice (4).

(J) GFP^{+} nuclei versus percent pathogenic allele correction from gDNA HTS of bulk nuclei from cortex (top) or hippocampus (bottom). Each point represents a matched data pair from a single mouse. Pearson’s correlation coefficient (r) is shown for D801N (blue) and E815K (orange).

(K–N) Hippocampal Atp1a3 western blot from WT and (K) D801N mice and (L) E815K mice treated with D801N-PE-AAV9 and E815K-PE-AAV9, respectively, (“PE”) or phosphate-buffered saline (PBS, “Vehicle”), and accompanying densitometry (M and N) to assess Atp1a3 signal intensity relative to WT vehicle-treated samples, normalized to GAPDH signal intensity. Each gel lane represents a single mouse, and plotted data represent the mean of at least $n = 2$ mice, with dots showing individual mice.

(O) Percent pathogenic allele correction from gDNA (top) or cDNA (bottom) HTS of bulk hippocampal nuclei versus the Atp1a3 activity ratio of PE-treated D801N (blue) or PE-treated E815K (orange) mice and vehicle-treated WT mice. Each point represents a matched data pair from a single mouse. Pearson’s correlation coefficient (r) is shown.

(P) Serum levels of neurofilament light chain (NFL) were analyzed in vehicle- or PE-treated E815K mice (30 weeks). One-way ANOVA followed by Tukey’s multiple comparisons test. ns, not significant; $^{*}p < 0.05$, $^{**}p < 0.01$, $^{***}p < 0.001$, and $^{****}p < 0.0001$.

For (C)–(H), data represent the mean of $n = 3$ independent biological replicates, and dots show individual replicate values.
